# Highly Selective Adsorption of Fluorinated Gases by Porous Organic Cages – Effects of Fluorinated Side‐Chains

**DOI:** 10.1002/adma.202516358

**Published:** 2025-11-21

**Authors:** Ke Tian, Wen‐Shan Zhang, Anjana Kunhumbadukka Othayoth, Moritz Philip Schuldt, Francesco Walenszus, Frank Rominger, Rasmus R. Schröder, Sven Michael Elbert, Michael Mastalerz

**Affiliations:** ^1^ Organisch‐Chemisches Institut Ruprecht‐Karls‐Universität Heidelberg Im Neuenheimer Feld 272 69120 Heidelberg Germany; ^2^ Bioquant, Ruprecht‐Karls‐Universität Heidelberg Im Neuenheimer Feld 267 69120 Heidelberg Germany; ^3^ 3P Instruments GmbH & Co. KG Bitterfelder Str. 1‐5 04129 Leipzig Germany

**Keywords:** F‐gases, nitrogen trifluoride, perfluoro carbons, PFAS, porous materials, porous organic cages, sulfur hexafluoride

## Abstract

Perfluorocarbons (PFCs) as well as related gases such as sulfur hexafluoride (SF_6_) or nitrogen trifluoride (NF_3_) are examples of F‐gases, a subclass of per‐ and polyfluoroalkyl substances (PFAS). F‐gases are anthropogenic greenhouse gases with the highest global warming potentials known and thus contribute significantly to global warming. To reduce their amount in the atmosphere, selectively adsorbing materials would be beneficial, but such materials are rare. A homologous series of porous organic cages with fluorinated side‐chains is synthesized and depending on the targeted gas as well as operating temperature, different cages have high selectivities for certain F‐gases against N_2_, CO_2_, and O_2_. Within this series, new benchmark selectivities against, e.g., perfluoro propane (PFC‐218) and perfluoro cyclobutane (PFC‐318) have been achieved based on attractive fluorine‐fluorine interactions. Furthermore, these interactions are exploited for the first time in the adsorption of sulfur hexafluoride and nitrogen trifluoride.

## Graphical abstract

A systematic study of a series of porous organic cages with perfluorinated alkyl side‐chains of different lengths is presented. The structure‐property relationship of the various cages on gas sorption of highly potent greenhouse gases, such as perfluorocarbons (PFCs), sulfur hexafluoride (SF_6_), and nitrogen trifluoride (NF_3_) is investigated. Very high selectivities have been obtained especially for PFC‐218 and PFC‐318.

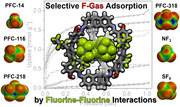

## 1 Introduction

Per‐ and polyfluoroalkyl substances (short; PFAS) have one of the strongest single bonds in chemistry, the C─F bond, making the compounds robust against chemical degradation.^[^ ^1^, ^2^ ^]^ This property in combination with others makes PFAS unique for various applications such as gaseous insulators in electrical equipment,^[^ ^3^ ^]^ for machinery manufacturing^[^ ^3^ ^]^ or as refrigerants.^[^ ^3^, ^4^ ^]^ While the resulting chemical longevity of such materials is beneficial for most of the corresponding applications, the strength of this bond also makes PFAS very persistent in the environment, up to an extent that they have been referred to as “forever chemicals”.^[^ ^5^ ^]^ This is due to their slow decomposition in nature^[^ ^6^ ^]^ which makes PFAS in general hazardous pollutants^[^ ^7^ ^]^ with a huge variety of negative consequences for humans,^[^ ^8^, ^9^, ^10^ ^]^ wildlife,^[^ ^11^, ^12^ ^]^ plants^[^ ^13^, ^14^, ^15^ ^]^ or the climate.^[^ ^16^, ^17^ ^]^ Some PFAS subclasses such as perfluorosulfonic acids (PFSAs, e.g. perfluorooctanesulfonic acid (PFOS))^[^ ^18^, ^19^, ^20^, ^21^, ^22^ ^]^ or perfluoroalkyl carboxylic acids (PFCAs, such as perfluorooctane acid (PFOA))^[^ ^18^, ^19^, ^20^, ^21^, ^22^ ^]^ have early been identified as serious pollutants due to their harming influence on the human body and thus were added to Annex A (elimination)^[^ ^23^ ^]^ or B (restriction)^[^ ^24^ ^]^ of the Stockholm Convention on Persistent Organic Pollutants in 2009 and 2019, respectively.^[^ ^25^ ^]^

Another prominent subclass of PFAS are fluorinated gases or F‐Gases. These are anthropogenic well‐mixed greenhouse gases (WMGHGs) and thus of major concern due to their impact to global warming,^[^ ^26^, ^27^, ^28^ ^]^ which is ≈2–3% of the overall contribution.^[^ ^17^, ^29^, ^30^ ^]^ Some of these gases have atmospheric lifetimes up to 50 000 years (tetrafluoromethane, PFC‐14).^[^ ^16^ ^]^ Based on updated data of the Sixth Assessment Report (AR6) of the Intergovernmental Panel on Climate Change (IPCC), the corresponding global warming potentials (GWPs), (which are also referred to as CO_2_‐equivalents) reach values up to 12 400 (for hexafluoroethane, PFC‐116) and even 24 300 for sulfur hexafluoride (SF_6_), meaning one ton of SF_6_ has the same effect on global warming over 100 years as 24 300 tons of CO_2_.^[^ ^16^ ^]^

Several F‐gas subclasses have been banned under the Kigali Amendment to the Montreal Protocol^[^ ^31^, ^32^ ^]^ for their use, such as the hydrofluorocarbons (HFC). While the import of HFCs has significantly decreased in the European Union (EU), the import of perfluorocarbons (PFCs) on the other hand is constantly increasing as data of the European Environment Agency (EEA) from 2013 to 2023 shows.^[^ ^33^ ^]^

**Figure 1:**
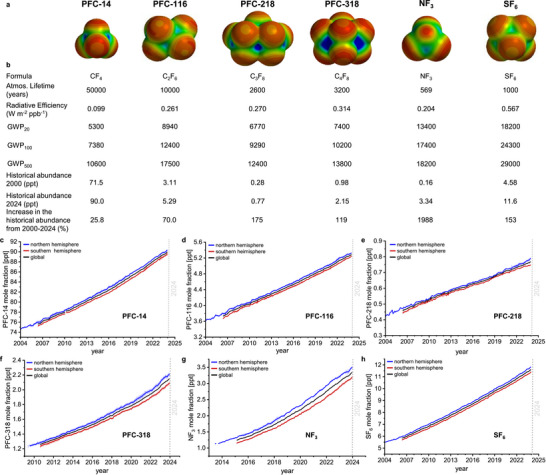
a) Space filling models of the WMGHGs tetrafluoromethane (PFC‐14), hexafluoroethane (PFC‐116), octafluoropropane (PFC‐218), octafluorocyclobutane (PFC‐318), nitrogen trifluoride (NF_3_), and sulfur hexafluoride (SF_6_) with corresponding electrostatic potential maps (depicted from 280 kJ mol^−1^ (blue) to −120 kJ mol^−1^ (red) in 20 bands), and b) table with selected physical properties of the WMGHGs based on updated values of the IPCCs AR6.^[^ ^16^, ^17^ ^]^ c–h) Atmospheric concentrations of the WMGHGs over time (2004–2024) based on data of the Advanced Global Atmospheric Gases Experiment (AGAGE).^[^ ^34^, ^35^ ^]^

Alarmingly, also the atmospheric abundancies of perfluorocarbons such as tetrafluoromethane (PFC‐14), hexafluoroethane (PFC‐116), octafluoropropane (PFC‐218) or octafluorocyclobutane (PFC‐318) are steadily increasing as indicated by recent data of the Advanced Global Atmospheric Gases Experiment (AGAGE) of NASA's Atmospheric Composition Focus Area in Earth Science (**Figure** **1**c–g).^[^ ^34^, ^35^ ^]^

Sources for PFC‐14 and PFC‐116 are mainly based on by‐product emission from metal smelting (mainly aluminium by the Hall‐Héroult process)^[^ ^36^, ^37^, ^38^, ^39^ ^]^ as well as from emissions of unused gas in plasma etching in the semiconductor industry.^[^ ^36^, ^37^, ^38^, ^39^ ^]^ PFC‐218 is also used for the latter,^[^ ^40^ ^]^ but in addition to that used in medical treatments like as an ultrasound contrast agent^[^ ^41^ ^]^ or in vitrectomy eye surgeries.^[^ ^42^ ^]^ In the case of PFC‐318, the semiconductor and microelectronics industry significantly decreased its emission in recent years, but it was found that the main source of PFC‐318 is evolved during the production of tetrafluoroethene (TFE), a monomer in polytetrafluoroethylene (PTFE) production.^[^ ^43^, ^44^ ^]^ These sources led to an increase of atmospheric abundancies for PFC‐218 and PFC‐318 of 175% and 119%, respectively between the years 2000 and 2024 (Figure 1b).^[^ ^34^, ^35^ ^]^

Besides PFCs, other fluorinated gases contribute to global warming as well. For instance, SF_6_ shows a similar increase in atmospheric abundance of 153%, which is mainly due to its use as insulator gas in high‐voltage switchgears.^[^ ^45^, ^46^ ^]^ The most dramatic growth with 1988%(!) is reported for nitrogen trifluoride (NF_3_) due its emissions during the production and use in the semiconductor industry.^[^ ^47^, ^48^ ^]^

Since F‐gases are crucial for some of the applications listed above and difficult to substitute, selective adsorbents would be beneficial to reduce their emissions on‐site.

In 2022, we developed a fluoroalkyl‐chain containing porous organic cage (POC) compound (“**F‐cage”**; here named as **C_4_F_9_‐Cage**), which is to the best of our knowledge among the benchmark materials for the adsorption of C_3_F_8_ (PFC‐218) and *c*‐C_4_F_8_ (PFC‐318).^[^ ^49^ ^]^ By comparison of cages with different degree of side‐chain fluorination it was concluded, that especially the second CF_2_‐unit of the side‐chains played a major role in the adsorption processes.^[^ ^49^ ^]^ POCs in general are ideal platforms to study structure‐property‐relationships even in respect of small differences of the molecular scaffolds due to their monodisperse nature, the lack of defects and the widened analytical toolbox in comparison to polymeric or network materials.^[^ ^50^, ^51^, ^52^, ^53^, ^54^, ^55^, ^56^, ^57^, ^58^, ^59^, ^60^, ^61^, ^62^ ^]^ The interaction of the fluorinated side‐chains of the highly selective cage compound with the corresponding gas by weak fluorine‐fluorine interaction was found to be a key factor for the good adsorption properties of PFCs.^[^ ^63^, ^64^, ^65^, ^66^, ^67^, ^68^ ^]^ With the knowledge gained in our first study, we were interested in the effect that perfluorinated side‐chains of different lengths will have for the adsorption behavior for PFCs and other fluorinated gases (NF_3_ and SF_6_) and thus their potential to selectively remove these from mixtures with N_2_.

## 2 Results and Discussion

**Figure 2:**
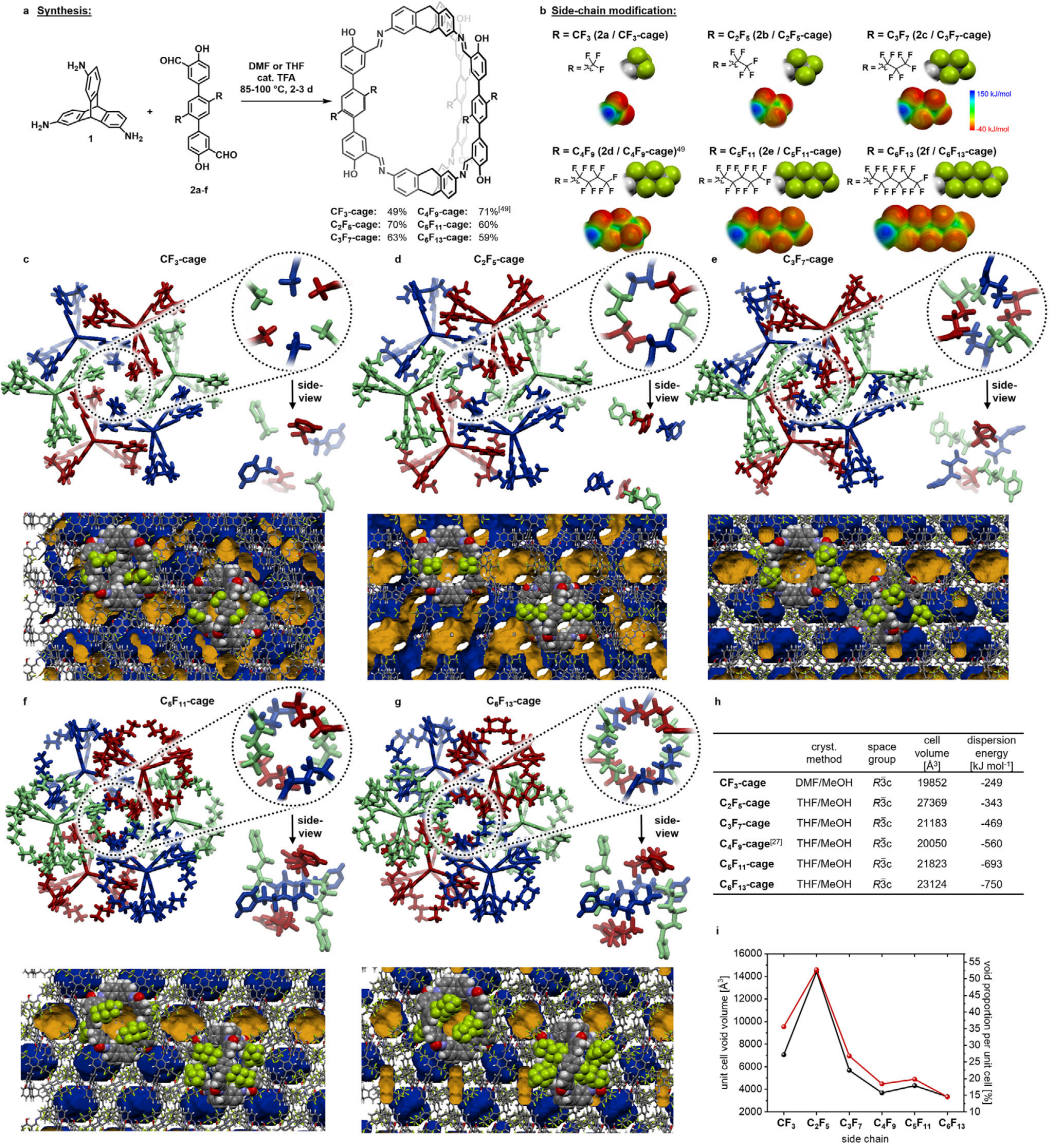
a) Synthesis of **CF_3_‐** to **C_6_F_13_‐cage** by imine condensation reactions of triamino triptycene **1** ^[^ ^69^ ^]^ and terphenyl bis‐salicylaldehydes **2a**–**2f** (for synthetic details and characterization, see Supporting Information). b) Side‐chain modifications of the terphenyl bis‐salicylaldehydes **2a**–**2f** and corresponding organic cage compounds as structural formulas, space‐filling models, and electrostatic potential maps (the electrostatic potentials are depicted from 150 kJ mol^−1^ (blue) to −40 kJ mol^−1^ (red) in 20 bands) calculated on the B3LYP‐6‐311G^**^ level of theory. c–g) Crystalline packing of **CF_3_‐** (c), **C_2_F_5_‐** (d), **C_3_F_7_‐** (e), **C_5_F_11_‐** (f) and **C_6_F_13_‐cage** (g): top: Hexameric alignment of cages along the crystallographic *c*‐axis. Cages depicted in the same color are equal by inversion symmetry. Zoom‐in to the hexameric side‐chain alignments as top view. Side view on the side‐chain alignments. bottom: Crystalline voids represented as contact surfaces (blue outer surface, yellow inner surface, created by Mercury 2020.1 with a probe radius of 1.82 Å and a grid spacing of 0.5 Å). For **C_4_F_9_‐ cage** see CCDC 2 150 481 and Ref.[49] Dispersion interaction energy of the hexameric side‐chain alignment depicted in (c)–(g) is calculated by XDM methods (B3LYP/aug‐cc‐pVDZ) and summarized in Table 2h.^[^ ^79^ ^]^ h) Summary of crystal parameters. i) Void analysis in respect of the corresponding side chains. Black: Unit cell void volume; red: void proportion per unit cell.

**Figure 3:**
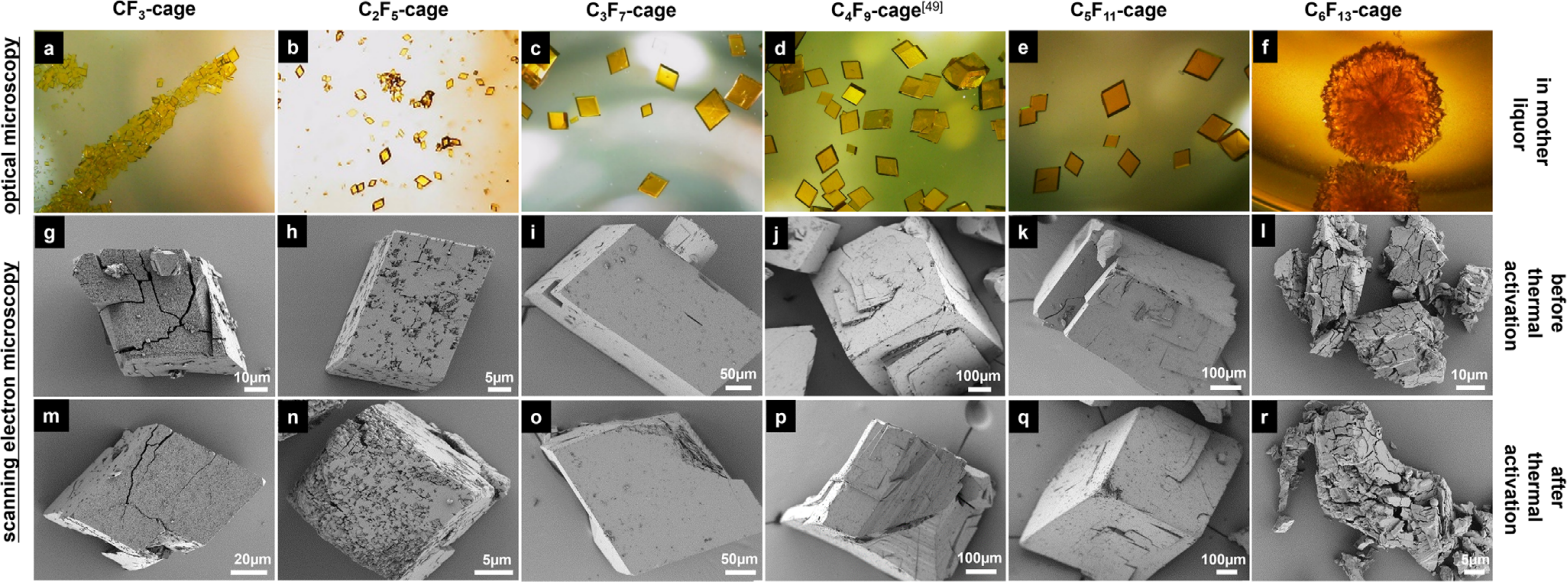
Crystallinity and morphology of **CF_3_‐cage** to **C_6_F_13_‐cage** investigated by optical microscopy images: a–f) in the THF/MeOH or DMF/MeOH mother liquors (for details see Figure 2) and g–r) by scanning electron microscopy images before and after activation.

The cage compounds studied in this report differ in length of their perfluorinated alkyl side‐chains (C1 to C6, see **Figure** **2**a). The condensations of triamino triptycene **1** ^[^ ^69^ ^]^ with the corresponding salicyl aldehyde building blocks **2a‐f** gave the cage compounds in yields between 49% and 70% under slightly varying conditions (Figure 2a; for details and full analytical data see Chapter S2, Supporting Information). To fully understand the nature of a change in property by a structural modification, the key challenge for discrete porous materials is to obtain isomorphous solid state structures to exclude variations of properties by different packing motifs and thus different pore structures.^[^ ^70^, ^71^, ^72^, ^73^, ^74^, ^75^, ^76^ ^]^ Crystalline modifications of **CF_3_‐cage**, **C_3_F_7_‐cage**, **C_5_F_11_‐cage** and **C_6_F_13_‐cage** were obtained in the same trigonal space group (*R* $\bar{3}$ c) with *Z* = 6 and with comparable cell volumes (19 852–23124 Å^3^) as the parent **C_4_F_9_‐cage** ^[^ ^49^ ^]^ (20 050 Å^3^; see Table h in Figure 2). In the case of **C_2_F_5_‐cage** a larger cell volume of 27 369 Å^3^ is found due to an elongated crystallographic *c*‐axis (*c *= 77.8 Å). Nevertheless, this difference can be neglected because the main packing motifs of **C_2_F_5_‐cage** correlate well with the ones found for the other five cages of the series (Figure 2). In our initial study, we identified π‐stacking as main driving force for the corresponding packing motifs^[^ ^49^ ^]^ which are similar for all other cages (Figure 2c–g). The comparable crystalline packing of **CF_3_‐cage** to **C_5_F_11_‐cage** is also reflected by almost identical outer appearances as indicated by optical as well as scanning electron microscopy (SEM; **Figure** **3**). An exception are the crystals of **C_6_F_13_‐cage** which are smaller and misshaped (Figure 3f,l,r) but they are still isomorphous.

Thermogravimetric analyses (TGA) revealed that all cages are stable up to 430 °C, enabling thermal activation methods. For mild desolvation, the cages have been treated first in dynamic vacuum (*p* ≈1×10^−3^ mbar) at room temperature for 24 h, followed by thermal activation at 50 °C for 16 h. The successful activation was proven by ^1^H NMR spectroscopy where no traces of enclathrated residual solvents from the crystallization processes were found. Powder X‐ray diffraction analyses (PXRD, see Figures S125 and S126, Supporting Information) revealed that during activation, **CF_3_‐cage** underwent a phase transition and the obtained PXRD pattern is not in correspondence with the one calculated from SCXRD, although proving still a high degree of crystallinity. Also in the case of **C_2_F_5_‐cage** a phase change can be observed, but this time, the resulting PXRD pattern is in good agreement to the ones obtained for **C_3_F_7_‐cage**, **C_4_F_9_‐cage**, **C_5_F_11_‐cage** and **C_6_F_13_‐cage**, which are all the same before and after activation. This indicates, that the slightly different crystalline lattice of activated **C_2_F_5_‐cage** (see discussion above) corresponds to the one of **C_3_F_7_‐cage**, **C_4_F_9_‐cage**, **C_5_F_11_‐cage** and **C_6_F_13_‐cage** after thermal treatment and that the latter have thermally stable crystalline lattices even after desolvation. This difference in behavior can most likely be explained by the increasing contribution of dispersion interactions^[^ ^49^, ^64^ ^]^ to the lattice stability with longer chain lengths (Figure 2h). For the **CF_3_‐cage**, the chains are too short to further stabilize the crystal packing by additional dispersion interactions, explaining the observed phase‐change (Figure 2h). All other activated crystals of **C_2_F_5_‐** to **C_6_F_13_‐cage** show very similar patterns by PXRD, concluding that these five cages are isomorphous in the solid state. As mentioned before, this is ideal to investigate the influence of different perfluoro alkyl chain‐lengths on the resulting gas adsorption behavior.

**Figure 4:**
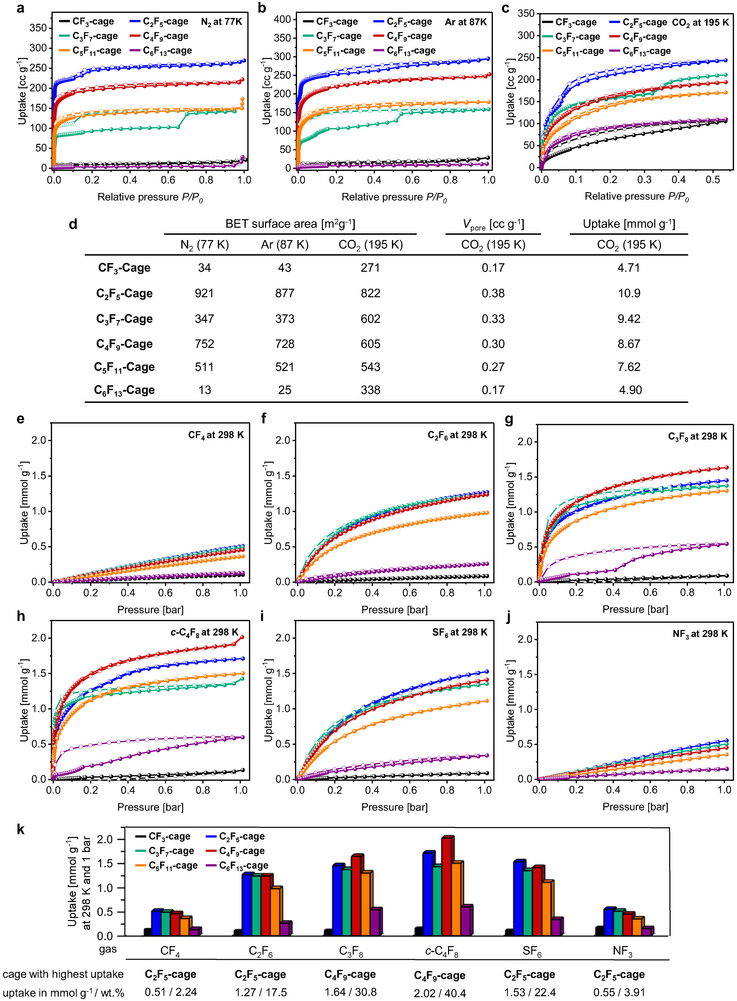
Pore analyses of **CF_3_‐cage** to **C_6_F_13_‐cage** investigated by gas sorption (adsorption: full circles; desorption: empty circles): a) Nitrogen sorption isotherms at 77 K; b) argon sorption isotherms at 87 K; c) carbon dioxide sorption isotherms at 195 K; d) Table comparing the adsorption properties derived from cryogenic measurements. e,f) Gas sorption isotherms of PFC‐14 (e), PFC‐116 (f), PFC‐218 (g), PFC‐318 (h), SF_6_ (i) and NF_3_ (j) at 298 K. k) Column diagram comparing the uptake of the investigated perfluorinated gases at 298 K and 1 bar.

Cryogenic gas sorption measurements with nitrogen at 77 K and argon at 87 K (**Figure** **4**) showed that activated **CF_3_‐cage** was poorly porous with specific surface areas of *SA*_BET_ = 34 m^2^g^−1^ (N_2_, 77 K) and *SA*_BET_ = 43 m^2^g^−1^ (Ar, 87 K). This indicates that the crystalline phase of **CF_3_‐cage**, which is obtained after thermal activation, does not have the same network of pores connected by perfluorinated channels as the initial single crystal X‐ray structure suggests (Figure 2c). In contrast to that, **C_2_F_5_‐** to **C_5_F_11_‐cage** all show accessible pores under these conditions (see Figure 4). Within this series, **C_2_F_5_‐Cage** has the highest specific surface area with *SA*_BET_ = 921 m^2^g^−1^ (N_2_, 77 K) and *SA*_BET_ = 877 m^2^g^−1^ (Ar, 87 K). The specific surface areas of the **C_2_F_5_‐** to **C_5_F_11_‐cage** show an odd‐even effect with **C_3_F_7_‐cage** (347 and 373 m^2^g^−1^) having a substantially lower surface area than **C_2_F_5_‐cage**, followed by a higher surface area in the case of **C_4_F_9_‐cage** (752 and 728 m^2^g^−1^)^[^ ^49^ ^]^ and again a smaller one for **C_5_F_11_‐cage** (511 and 521 m^2^g^−1^). **C_6_F_13_‐cage** has the lowest porosity with *SA*_BET_ = 13 m^2^g^−1^ (N_2_, 77 K) and *SA*_BET_ = 15 m^2^g^−1^ (Ar, 87 K) while showing a similar PXRD pattern and thus solid state structure as **C_2_F_5_‐** to **C_5_F_11_‐cage**. This is most likely caused by a hindered diffusion of the gases through the hexameric side‐chain alignments due to stronger F‐F‐ and dispersion interactions (Figure 2h).^[^ ^49^, ^64^ ^]^ This hypothesis is supported by measurements with carbon dioxide at 195 K where for **C_6_F_13_‐cage** a specific surface area of *SA*_BET_ = 338 m^2^g^−1^ is obtained due to weakened side‐chain to side‐chain interactions at higher temperature and also a less hindered diffusion due to the smaller kinetic diameter of carbon dioxide (3.3 Å).^[^ ^77^, ^78^ ^]^ Based on similar assumptions, the measured surface area for **CF_3_‐cage** (*SA*_BET_ = 271 m^2^g^−1^) using carbon dioxide can be explained, while being non‐porous at 77 and 87 K with N_2_ or Ar. **C_2_F_5_‐cage** has again the highest surface area (*SA*_BET_ = 822 m^2^g^−1^) at 195 K with carbon dioxide and **C_3_F_7_‐** to **C_5_F_11_‐cage** have comparable surface areas of 543–616 m^2^g^−1^ with a general trend toward smaller specific surface areas with longer chains. A similar effect is found for the corresponding pore volumes that reduces from 0.38 to 0.17 cc g^−1^ from **C_2_F_5_‐cage** to **C_6_F_13_‐cage** (Figure 4d). It is worth to mention, that the N_2_‐ and Ar‐isotherms of **C_2_F_5_‐** and the **C_3_F_7_‐cage** each show steps at *P*/*P*_0_ = 0.015 and *P*/*P*_0_ ≈ 0.10–0.15 with **C_3_F_7_‐cage** having a pronounced hysteresis and the other cages with longer chains not.

The deviation in the hysteresis of the **C_3_F_7_‐cage** is more pronounced than for **C_2_F_5_‐cage**. This can be explained by a kind of breathing effect,^[^ ^80^ ^]^ where the F‐F interactions or dispersion interactions need to break by the adsorbed gas and that for the longer chains, this interaction is too strong to occur during equilibration in a manner to be detected. However, this phenomenon needs to be investigated in more detail, which we currently work at. Since these kinetic effects do not affect the thermodynamic behavior, it will not be discussed herein.

The adsorption of F‐gases by all six cages was investigated at four different temperatures (273, 283, 298, and 313 K; see Figure 4 and Chapter S9, Supporting Information). In general, the **CF_3_‐cage** and the **C_6_F_13_‐cage** have the smallest uptakes of all investigated gases, which can be explained by the very small accessible surface areas. At 1 bar and 298 K, the **C_2_F_5_‐cage** with the highest BET‐surface area adsorbs the largest amounts of the smaller PFC‐14 (0.51 mmol g^−1^) and PFC‐116 (1.27 mmol g^−1^). The uptake of PFC‐116 is in the range of the reported adsorption by activated carbon at 303 K (1.38 mmol g^−1^) and lower than reported for zeolite 13X (1.53 mmol g^−1^ at 303 K).^[^ ^81^ ^]^ Decreasing amounts of PFC‐14 are adsorbed in materials with increasing chain lengths for **C_2_F_5_‐** to **C_5_F_11_‐cage**. For PFC‐116, the uptakes of **C_2_F_5_‐** to **C_4_F_9_‐cage** are almost similar with 1.24–1.27  mmol g^−1^ and **C_5_F_11_‐cage** adsorbs smaller amounts of 0.98 mmol g^−1^. For PFC‐218, PFC‐318 and SF_6_ small odd‐even‐effects are found in the overall uptakes (Figure 4k). In the case of the larger gases PFC‐218 and PFC‐318, **C_4_F_9_‐cage** performs better than the **C_2_F_5_‐cage** and adsorbs the highest amounts with 1.64 mmol g^−1^ (PFC‐218) and 2.02 mmol g^−1^ (PFC‐318). For SF_6_ and NF_3_, again **C_2_F_5_‐cage** is the better candidate with 1.14 (at 313 K) to 2.21 mmol g^−1^ (at 273 K) for SF_6._

This is comparable to cage CC3α (2.3 mmol g^−1^ at 273 K) reported by Cooper and co‐workers, but lower than to the significantly larger (but less selective) CC5α (≈3.5 mmol g^−1^).^[^ ^82^ ^]^ To the best of our knowledge, the adsorption of NF_3_ by porous organic cages has not been reported. It is worth mentioning that the adsorbed amounts of 0.31 mmol g^−1^ (at 313 K) to 1.22 mmol g^−1^ (at 273 K) by **C_2_F_5_‐cage** are in the range as described for MOFs like HKUST‐1 (1.48 mmol g^−1^ at 298 K) and Ni(pba)_2_ (1.22 mmol g^−1^) but significantly lower than for ATC‐Cu (3.25 mmol g^−1^).^[^ ^83^, ^84^ ^]^

As expected, all cages adsorb more gas at lower temperatures. For example, **C_2_F_5_‐cage** adsorbs up to 1.11 g mmol^−1^ of PFC‐14 and 1.91 mmol g^−1^ of PFC‐116 at 273 K and 1 bar. For PFC‐218 (2.09 mmol g^−1^) and PFC‐318 (4.41 mmol g^−1^) **C_4_F_9_‐cage** shows the highest adsorption at 273 K (see, Table S15, Supporting Information).

By fitting virial expressions to the isotherms at 273 and 283 K the corresponding isosteric enthalpies of adsorption Δ*H*_ads_ have been calculated (**Table** **1**).^[^ ^85^ ^]^ In the case of the **CF_3_‐** and the **C_6_F_13_‐cage**, the fittings result in large error margins, having insufficient fitting parameters or even non‐converging fits due their low uptakes of the investigated gases and therefore are not reliable to be further discussed.

**Table 1:** Isosteric enthalpies of adsorption Δ*H*_ads_ of **CF_3_‐** to **C_6_F_13_‐cage** in kJ mol^−1^.

cmp.	gas					
	PFC‐14	PFC‐116	PFC‐218	PFC‐318 ^a)^	SF_6_	NF_3_
**CF_3_‐cage**	−31.4±4.8	−23.8±3.8	‐ ^b)^	‐ ^b)^	‐ ^b)^	‐^b)^
**C_2_F_5_‐cage**	−27.1±3.8	−28.1±0.6	−32.0±1.1	−54.6±2.2	−22.9±3.9	−19.5±0.4
**C_3_F_7_‐cage**	−17.6±1.5	−26.1±0.7	−22.9±2.4	−80.1±16.7 ^c)^	−30.7±4.2	−22.9±2.4
**C_4_F_9_‐cage**	−24.1±2.0	−31.6±0.3	−40.1±0.8	−46.2±0.8	−33.2±1.7	‐
**C_5_F_11_‐cage**	−26.4±1.6	−27.2±0.9	−32.5±1.3	−32.7±2.0	−25.1±5.3	−17.6±1.1
**C_6_F_13_‐cage**	−24.1±4.6	−17.4±2.7	‐ ^b)^	‐ ^b)^	‐ ^b)^	‐ ^b)^

^a)^ due to the pore condensation of PFC‐318 at pressures above 0.5 bar only data points > 0.5 bar have been considered.

^b)^ not determined due to insufficient fitting parameters or non‐converging fits.

^c)^ non‐reliable value due to insufficient fitting parameters.

The highest isosteric enthalpies of adsorption of PFC‐14 are found for **C_2_F_5_‐cage**, **C_4_F_9_‐cage** and **C_5_F_11_‐cage** in a similar range of Δ*H*_ads_ = −24.1±2.0 to −27.1±3.8 kJ mol^−1^. These values increase in case of the larger PFC‐116 for all cages with **C_4_F_9_‐cage** having the highest Δ*H*_ads_ = −31.6±0.3 kJ mol^−1^. **C_4_F_9_‐cage** also has the highest Δ*H*_ads_ of −40.1±0.8 kJ mol^−1^ toward the adsorption of PFC‐218 which is 2.9 kJ mol^−1^ higher than the one reported for CPOF‐6 (Δ*H*_ads_ = −37.2 kJ mol^−1^)^[^ ^86^ ^]^ and only slightly lower than the one reported for MOF A520 (Δ*H*_ads_ = −43.6 kJ mol^−1^).^[^ ^87^ ^]^ For PFC‐318, **C_2_F_5_‐cage** was found to have with Δ*H*_ads_ = −54.6±2.2 kJ mol^−1^ the highest isosteric enthalpies of adsorption. This value exceeds the one reported for CPOF‐6 (Δ*H*_ads_ = −33.6 kJ mol^−1^)^[^ ^86^ ^]^ and is the highest Δ*H*_ads_ of the entire study. In general, **C_2_F_5_‐** to **C_5_F_11_‐cages** show increasing isosteric enthalpies of adsorption with increasing PFC size (Table 1), indicating the contribution of fluorine‐fluorine interactions in the binding events (see discussion below). For SF_6_ and NF_3_ smaller Δ*H*_ads_ are observed. Again, **C_4_F_9_‐cage** shows the highest affinity toward SF_6_ with Δ*H*_ads_ = −33.2±1.7 kJ mol^−1^ which is only slightly lower than the one of SF_6_‐selective cage compound CC3α (−35 to −40 kJ mol^−1^).^[^ ^82^ ^]^ NF_3_ being the gas with the least fluorine atoms per molecule, also shows the lowest isosteric enthalpies of adsorption between −17.6±1.1 and −22.9±2.4 kJ mol^−1^.

**Figure 5:**
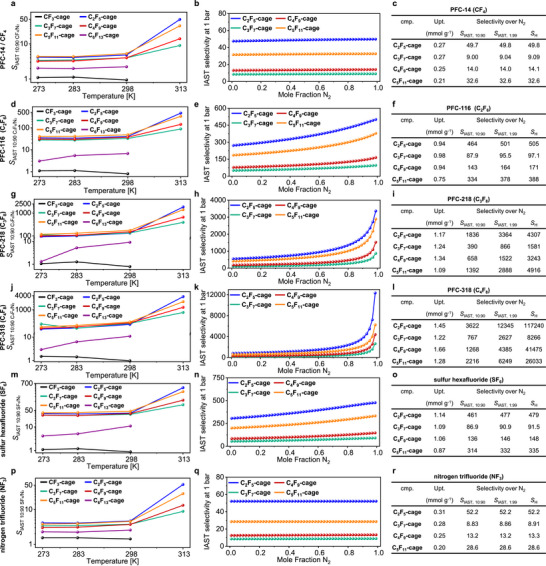
Gas sorption selectivities of **CF_3_‐cage** to **C_6_F_13_‐cage**. First column a,d,g,j,m,p): IAST selectivities of 10:90 mixtures of the corresponding PFCs in nitrogen at 1 bar at various temperatures. Second column b,e,h,k,n,q): IAST selectivities of the PFCs versus nitrogen at 1 bar at various compositions. Third column c,f,i,l,o,r): Uptakes, IAST selectivities and Henry selectivities of the investigated PFCs at 313 K. Note that the data of **C_4_F_9_‐cage** are from our previous work.^[^ ^49^ ^]^

To evaluate the material's potential applications adsorbing small amounts of pollutants, the selectivities for the adsorption of F‐gases in favor to N_2_ were calculated. First Henry selectivities were calculated, but also behavior according to the ideal adsorbed solution theory (IAST) for 10:90‐ and 1:99‐mixtures, mimicking real‐life conditions was examined.^[^ ^88^, ^89^, ^90^ ^]^ In general, selectivities of **CF_3_‐cage** and **C_6_F_13_‐cage** are very small and are not further discussed herein. All other cages show a similar behavior for all gases in the investigated temperature window between 273 and 298 K (**Figure** **5**, first column from left). For example, in the case of PFC‐116 at 298 K Henry selectivities between *S*_H_ = 38.1 (**C_3_F_7_‐cage**) and *S*_H_ = 65.4 (**C_5_F_11_‐cage**) are found. The corresponding IAST selectivities for 10:90 mixtures of PFC‐116 and nitrogen are in a similar range between *S*_IAST, 10:90_ = 32.6 (**C_3_F_7_‐cage**) and *S*_IAST, 10:90_ = 47.8 (**C_5_F_11_‐cage**). This values are significantly lower in comparison to those reported for MOFs like Al‐Fum (*S*_IAST, 10:90_ = 299.6) or MOF‐303 (*S*_IAST, 10:90_ = 149.1) but higher than for MIL‐160 (*S*_IAST, 10:90_ = 29.6).^[^ ^91^ ^]^ Furthermore, the gas composition plays a role, giving higher selectivities of *S*_IAST, 1:99_ = 36.5 (**C_3_F_7_‐cage**) and *S*_IAST, 1:99_ = 58.3 (**C_5_F_11_‐cage**) at PFC contents of 1%.

At 298 K, **C_5_F_11_‐cage** also showed the highest IAST selectivities for the adsorption of PFC‐14 (*S*_IAST, 10:90_ = 5.29), PFC‐218 (*S*_IAST, 10:90_ = 174) and PFC‐318 (*S*_IAST, 10:90_ = 301). However, values for **C_2_F_5_‐** to **C_4_F_9_‐cages** were in a similar regime (Figure 5, first column and Table S16, Supporting Information). The selectivity of PFC‐218 against nitrogen is higher than the one of CPOF‐6 (*S*_IAST, 10:90_ = 148)^[^ ^86^ ^]^ and is to the best of our knowledge only surpassed by that of the recently reported MOF A520 (*S*_IAST, 10:90_ = 6034)^[^ ^87^ ^]^ and the corresponding PFC‐318 selectivity is close the highest reported (*S*_IAST, 10:90_ = 418) of recently reported CPOF‐6.^[^ ^86^ ^]^

At 313 K (40 °C), all selectivities drastically increase for all compounds and for all gases, especially the larger ones, whereas for the smaller gases PFC‐14 and NF_3_ almost no temperature effect is observed. The increasing selectivity with increasing temperature has prior been observed for other gases and is known as N_2_‐phobic effect.^[^ ^92^, ^93^ ^]^ At 313 K, **C_2_F_5_‐cage** shows the highest selectivities for all investigated gases (e.g. *S*_IAST, 1:90_ = 1836 and *S*_IAST, 1:99_ = 3364 for PFC‐218 and *S*_IAST, 1:90_ = 3622 and *S*_IAST, 1:99_ = 12 345 for PFC‐318) with *S*_H _= 4307 (PFC‐218) and *S*_H _= 117 240 (PFC‐318). These values are better than those of **C_4_F_9_‐cage** (PFC‐218: *S*_IAST, 10:90_ = 658; *S*_H _= 3243; PFC‐318: *S*_IAST, 10:90_ = 1268; *S*_H _= 41 475; Figure 5) from our initial study and are benchmark values for these gases.

We also investigated SF_6_ and NF_3_ as other F‐gases than PFCs. **C_2_F_5_‐cage** shows a selectivity of *S*_IAST, 10:90_ = 38.7 at 298 K and 1 bar for SF_6_ over nitrogen. While this is lower compared to materials such as SBMOF‐1 (*S*_IAST, 10:90_ = 325 at 298 K),^[^ ^94^ ^]^ HKUST‐1c (*S*_IAST, 10:90_ = 70.4 at 298 K),^[^ ^95^ ^]^ Zn‐MOF‐74 (*S*_IAST, 10:90_ = 46 at 298 K),^[^ ^89^ ^]^ CPOF‐12 (*S*_IAST, 10:90_ = 149 at 298 K)^[^ ^96^ ^]^ or cage compound CC‐3α (*S*_IAST, 10:90_ = 74 at 298 K, 178 at 273 K),^[^ ^82^ ^]^ the selectivity significantly increases to *S*_IAST, 10:90_ = 461 at 313 K outperforming the above mentioned numbers. It is worth mentioning that most materials show lower selectivities with increasing temperature.^[^ ^82^, ^89^, ^95^ ^]^ This value of 461 is to the best of our knowledge only surpassed by some MOFs such as Ni(NDC)(TED)_0.5_ (*S*_IAST, 10:90_ = 750),^[^ ^97^ ^]^ Ni(adc)(dabco)_0.5_ (*S*_IAST, 10:90_ = 948)^[^ ^98^ ^]^ or Al(fum) and its composites (*S*_IAST, 10:90_ = 28978–50139)^[^ ^99^ ^]^ which were tailor‐made for SF_6_ adsorption.

In the same way, the selectivity of **C_2_F_5_‐cage** toward the adsorption of NF_3_ at 298 K is with *S*_IAST, 10:90_ = 4.67 low compared to benchmark materials such as Ppy‐POF (*S*_IAST, 1:99_ = 48.8 at 298 K)^[^ ^100^ ^]^ or PC‐750 (*S*_IAST, 10:90_  = 61.4 at 298 K),^[^ ^101^ ^]^ and independent from the composition of the binary gas mixture (*S*_IAST, 1:99_ = 4.67), but increases to *S*_IAST, 10:90_ = 52.2 at 313 K although with lower uptakes than the other materials (see discussion above).

All cages with relevant adsorption properties for potential applications (**C_2_F_5_‐** to **C_5_F_11_‐cage**) have been furthermore investigated in respect to their adsorption of F‐gases over other components of air such as oxygen and carbon dioxide. **C_5_F_11_‐cage** has for example Henry selectivities of *S*_H _= 1214 and *S*_H _= 13 896 for PFC‐218 and PFC‐318 over oxygen and *S*_H _= 106 and *S*_H _= 1212 for PFC‐218 and PFC‐318 over carbon dioxide. The corresponding IAST selectivities reach up to *S*_IAST, 1:99_ = 411 (PFC‐218 over oxygen), *S*_IAST, 1:99_ = 1021 (PFC‐318 over oxygen), *S*_IAST, 1:99_ = 19.1 (PFC‐218 over carbon dioxide) and *S*_IAST, 1:99_ = 54.7 (PFC‐318 over carbon dioxide). At 313 K, **C_2_F_5_‐cage** shows *S*_H _= 1520 (PFC‐218) and *S*_H _= 41 379 (PFC‐318) for the PFCs over oxygen and *S*_H _= 24 (PFC‐218) and *S*_H _= 654 (PFC‐318) for the PFCs over carbon dioxide. NF_3_ is the only gas, which is not selectively adsorbed in favor of carbon dioxide for all cages and all temperature, eliminating the application of the cages in processes with high carbon dioxide emissions. The obtained selectivities (see Supporting Information) exclude a blocking of the binding sites by those gases^[^ ^102^, ^103^, ^104^ ^]^ and underline a potential applicability of the cage compounds reported in this study.

**Figure 6:**
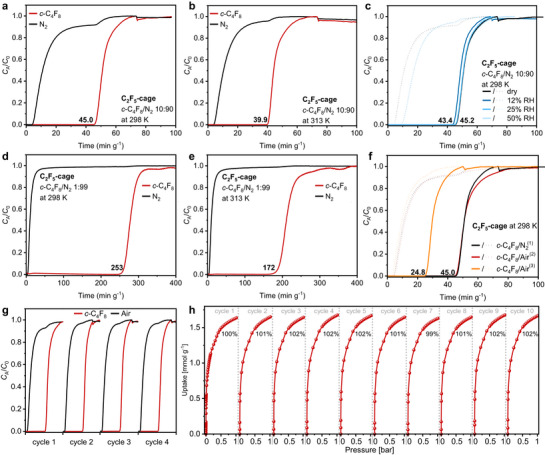
Breakthrough and recyclability of **C_2_F_5_‐cage**. a–g: Experimental breakthrough curves of PFC‐318 (*c*‐C_4_F_8_): (a) *c*‐C_4_F_8_/N_2_ 10:90 at 298 K; (b) *c*‐C_4_F_8_/N_2_ 10:90 at 313 K; () *c*‐C_4_F_8_/N_2_ 10:90 at 298 K at dry conditions (black curve), at 12% relative humidity (RH) (dark blue curve), at 25% RH (blue curve) and at 50% RH (light blue curve); (d) *c*‐C_4_F_8_/N_2_ 1:99 at 298 K; (e) *c*‐C_4_F_8_/N_2_ 1:99 at 313 K; (f) *c*‐C_4_F_8_/N_2_ 10:90 at 298 K (black curve); *c*‐C_4_F_8_/Air 10:90 at 298 K (red curve), *c*‐C_4_F_8_/Air+400 ppm CO_2_ 10:90 at 298 K (orange curve); (g) four cycles of breakthroughs of *c*‐C_4_F_8_/Air 10:90 at 298 K. h) Ten repetitive adsorption isotherms of *c*‐C_4_F_8_ at 298 K. All breakthrough measurements have been obtained at 10 mL min^−1^ in 50% as carrier gas at an overall pressure of 1 bar (if not otherwise mentioned). In Figure 6c,f the solid lines represent the *c*‐C_4_F_8_‐ and the dashed lines the N_2_‐breakthrough curves. In Figure 6f: (1)Breakthrough curve with pure nitrogen as comparison. (2) Synthetic air (79.5% N_2_ + 20.5% O_2_). (3) Synthetic air (79.5% N_2_ + 20.5% O_2_) with 400 ppm CO_2_ in He measured at 20 mL min^−1^ due to the flow rate range of the corresponding CO_2_ mass flow controller.

Crystalline material of **C_2_F_5_‐cage** was further investigated by dynamic breakthrough measurements to evaluate its potential to separate PFC‐318 (*c*‐C_4_F_8_) from binary or ternary gas mixtures (**Figure** **6**). The measurements have been conducted at flow rates of 10 mL min^−1^ in 50% helium as carrier gas at overall pressures of 1 bar. In all measurements an immediate breakthrough of N_2_ was observed. For 10:90 PFC‐318/N_2_ mixtures at 298 K, it was observed that PFC‐318 breaks through at 45.0 min g^−1^, and after 39.9 min g^−1^ at 313 K (Figures 6a,b). The faster breakthrough with higher temperature correlates to the decrease of the corresponding capacities of PFC‐318 of 0.85 mmol g^−1^ at 298 K to 0.69 mmol g^−1^ at 313 K. This temperature dependence of the uptakes is in accordance to the observed trend derived from single gas isotherms (see above). The selectivities increase from *S* = 47.8 at 298 K to *S* = 103.5 at 313 K due to a more pronounced decrease of the nitrogen capacities from 0.16 to 0.06 mmol g^−1^ which clearly supports the discussed N_2_‐phobicity of the investigated cages. The derived capacities are comparable with the ones obtained by the single gas isotherm measurements (≈0.90 mmol g^−1^ at 298 K and ≈0.74 mmol g^−1^ at 313 K at the corresponding partial pressures of ≈0.05 bar) while selectivities are in general lower compared to the ones calculated by IAST (*S*_IAST 10:90_ = 238 at 298 K and *S*_IAST_ _10:90_ = 3622 at 313 K) due to the very small and thus error‐prone determination of the nitrogen capacities. Humidity has a negligible influence on the separation performance of **C_2_F_5_‐cage** as the breakthrough time only varies between 43.4 and 45.2 min comparing dry conditions with up to 50% relative humidity (Figure 6c). For 1:99 PFC‐318/N_2_ mixtures at 298 and 313 K (Figures 6d,e) breakthrough times increased significantly to 253 and 172 min g^−1^. This time the temperature dependence of selectivities (*S* = 231.4 at 298 K and *S* = 189.5 at 313 K) is less pronounced. To be closer to real world conditions, 10:90 mixtures of PFC‐318/synthetic air (79.5% nitrogen + 20.5% oxygen) were investigated, showing identical breakthrough times of 45.0 min g^−1^ as for the corresponding experiments with PFC‐318/N_2_ mixtures (Figure 6f). The addition of 400 ppm carbon dioxide to the synthetic air resulted in a faster breakthrough at 24.8 min g^−1^, because a higher flow rate of 20 mL min^−1^ had to be used due to experimental reasons (see Supporting Information for details). The almost identical capacities of PFC‐318 during the experiments with synthetic air (0.87 mmol g^−1^) and synthetic air and carbon dioxide (0.86 mmol g^−1^) underlines the good separation performance of **C_2_F_5_‐cage** under real‐life conditions.

Next, the recyclability of **C_2_F_5_‐cage** was tested. In four cycles of breakthrough experiments with 10:90 PFC‐318/synthetic air mixtures the breakthrough times all were identical, and showed no loss of performance (Figure 6g). Similar to that, in ten repetitive single gas isotherms comparable gas uptakes between 1.63 and 1.68 mmol g^−1^ were measured at 1 bar, again, showing no hints of materials degradation (Figure 6h).

In our previous study, fluorine‐fluorine interactions have been identified as the driving force for the high selectivities by analyzing the Hirshfeld surface of residual electron density inside the cavity and the closest contacts to atoms of the cage.^[^ ^49^ ^]^

**Figure 7:**
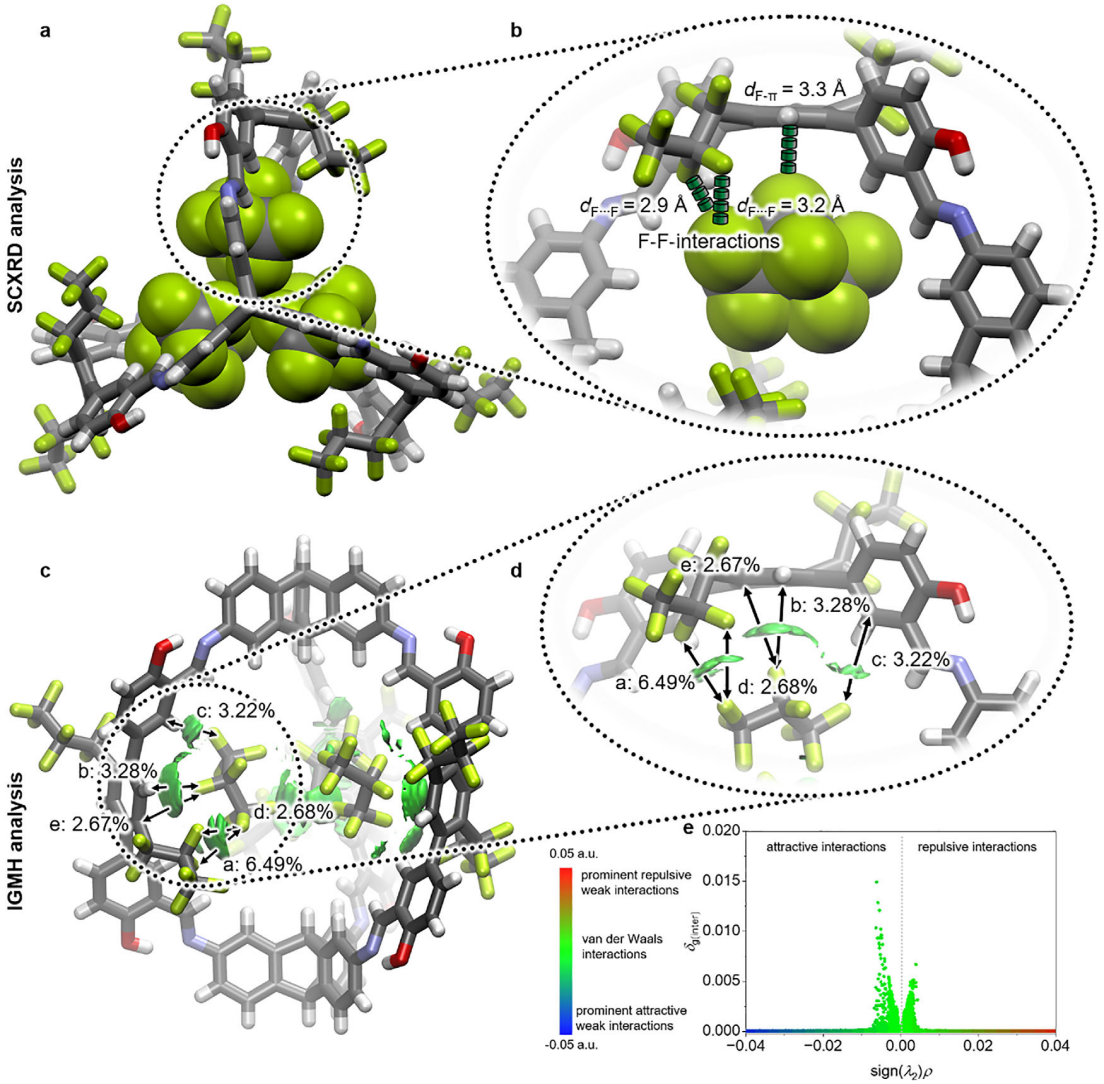
Top: Single crystal X‐ray structure of (C_3_F_8_)_3_⊂**C_3_F_7_‐cage** with the cage depicted as capped‐sticks model and the C_3_F_8_ molecules shown as space‐fill model. a) View along the triptycene bridge‐head atoms. b) Zoom‐in into the interaction of one C_3_F_8_ molecule with the cage. Bottom: IGMH isosurface with an isovalue of 0.002 and the percentage contributions of the single interactions. c) Whole structure with all three guest molecules. d) Zoom‐in into the interaction of one C_3_F_8_ molecule with the cage. e) IGMH scatterplot. Carbon: grey; hydrogen: white; oxygen: red; nitrogen: blue; fluorine: lime green.

This time, adsorbed PFC molecules could be refined, clearly supporting our previous assumption by single crystal X‐ray analysis of **C_3_F_7_‐cage** with three molecules of C_3_F_8_ (PFC‐218) in its cavity (**Figure** **7**). The corresponding crystals where obtained by exposing activated and evacuated single crystals of **C_3_F_7_‐cage** to a C_3_F_8_ atmosphere for several hours and subsequent analysis by SCXRD. Three molecules of C_3_F_8_ were found per **C_3_F_7_‐cage** and the corresponding (C_3_F_8_)_3_⊂**C_3_F_7_‐cage** crystallized in the triclinic space group *R* $\bar{3}$ that is reduced in symmetry to the parent **C_3_F_7_‐cage** crystals (*R* $\bar{3}$ c) due to a slightly different side‐chain alignment. As proposed, the C_3_F_8_ molecules interact with **C_3_F_7_‐cage** by fluorine‐fluorine interactions with *d*_F∙∙∙F_ = 2.9 Å between the terminal CF_3_‐group of the C_3_F_8_ molecules to the second CF_2_‐group of the perfluorinated side chains. This distance is in the range of F–F‐interaction reported before by different methods.^[^ ^105^, ^106^ ^]^ Furthermore, a CF‐π distance of *d*_F‐π_ = 3.3 Å is found to the central phenyl ring, which is slightly larger than reported for close CF‐π‐distances with *d*_F‐π_ = 3.0–3.2 Å.^[^ ^107^, ^108^, ^109^, ^110^ ^]^ To gain a deeper understanding of the interactions, these were visualized using the indepentent gradient model based on Hirshfeld partition (IGMH) analysis (Figure 7 bottom).^[^ ^111^ ^]^ As expected, the largest contribution (interaction a in Figure 6c,d) of 6.49% was detected between the second CF_2_‐group of the perfluoroalkyl side‐chains of the cage and the terminal CF_3_‐group of PFC‐218. This interaction has with *δ*_g(inter)_ = 0.026 a.u. a similar strength as found for the closest contact between two C_3_F_8_ molecules (*δ*_g(inter)_ = 0.028 a.u.) in the centre of (C_3_F_8_)_3_⊂**C_3_F_7_‐cage**, supporting the significance of this position for the binding of the gas. A second fluorine‐fluorine interaction (interaction d in Figure 7c,d) of the same fluorine of the CF_3_‐group with the other fluorine of the cages CF_2_‐group was determined to contribute with additional 2.68% (*δ*_g(inter)_ = 0.011 a.u.) resulting in an overall contribution of 9.17% per second CF_2_‐group not considering the threefold symmetry of the cage. The CF‐π‐interactions b, c and e contribute with 3.28%, 3.22%, and 2.67% to the overall binding. The analysis of the corresponding IGMH scatterplot (Figure 7e) proves predominant attractive over repulsive interactions and furthermore identifies the attractive interactions as van‐der‐Waals‐type.^[^ ^111^ ^]^

## 3 Conclusion

Based on our initial discovery of fluorine‐fluorine interactions as tool to explore F‐gas adsorbing materials, we synthesized a homologous series of porous organic cage compounds with CF_3_‐ to C_6_F_13_‐side‐chains. As a necessity for in‐depth structure‐property‐relationships, all cages were obtained in isomorphic solid‐state configurations with the exception of **CF_3_‐cage** after activation. For **C_2_F_5_‐** to **C_5_F_11_‐cage** specific surface areas up to 921 m^2^ g^−1^ where found surpassing **C_4_F_9_‐cage** (752 m^2^ g^−1^). The cages showed highly selective adsorption of F‐gases. At elevated temperatures of 313 K, **C_2_F_5_‐cage** outperforms all other members of this series with selectivities of *S*_IAST, 10:90_ = 1836 for perfluoropropane (PFC‐218) and *S*_IAST, 10:90_ = 3622 for perfluorocyclobutane (PFC‐318) over nitrogen. Single crystal X‐ray diffraction analysis unambiguously revealed fluorine‐fluorine interactions as the main driving force for the gas sorption process. In addition to PFCs, other F‐gases such as SF_6_ (uptake 1.14 mmol g^−1^, *S*_IAST, 10:90_ = 461 at 313 K) or NF_3_ (uptake 0.31 mmol g^−1^, *S*_IAST, 10:90_ = 52.2 at 313 K) were adsorbed by the fluorinated cages with uptakes and selectivities that can compete with a large number of other porous materials. Single crystal X‐ray as well as IGMH analyses underline the assumption of fluorine‐fluorine interactions playing a major role in the binding of the PFCs. In the future, we will investigate the potential application of the cages in adsorption of other PFAS such as fluorinated anaesthetics.^[^ ^112^ ^]^

## Conflict of Interest

The authors declare no conflict of interest.

## Supporting information



Supporting Information

Supporting Information

## Data Availability

The data that support the findings of this study are available from the corresponding author upon reasonable request.
