# Supplementary material for: Highly Selective Adsorption of Fluorinated Gases by Porous Organic Cages – Effects of Fluorinated Side‐Chains
Source: Adv Mater. 2025 Nov 21;38(7):e16358. doi: 10.1002/adma.202516358 (PMC12862739; doi:10.1002/adma.202516358)
Supplement: Supplementary file 1 — Supporting Information [file ADMA-38-e16358-s002.pdf]

ADVANCED MATERIALS

Supporting Information

for *Adv. Mater.*, DOI 10.1002/adma.202516358

Highly Selective Adsorption of Fluorinated Gases by Porous Organic Cages – Effects of Fluorinated Side-Chains

Ke Tian, Wen-Shan Zhang, Anjana Kunhumbadukka Othayoth, Moritz Philip Schuldt, Francesco Walenszus, Frank Rominger, Rasmus R. Schröder, Sven Michael Elbert and Michael Mastalerz**

Highly Selective Adsorption of Fluorinated Gases by Porous Organic Cages - Effects of Fluorinated Side-Chains

Ke Tian,[a] Wen-Shan Zhang,[a,b] Anjana Kunhumbadukka Othayoth,[a] Moritz P. Schuldt,[a] Francesco Walenszus,[c] Frank Rominger,[a] Rasmus R. Schröder,[b] Sven M. Elbert,*[a] and Michael Mastalerz*[a]

a. Organisch-Chemisches Institut, Ruprecht-Karls-Universität Heidelberg, Im Neuenheimer Feld 272, 69120 Heidelberg (Germany), E-mail: michael.mastalerz@oci.uni-heidelberg.de; sven.elbert@oci.uni-heidelberg.de

b. Bioquant, Ruprecht-Karls-Universität Heidelberg, Im Neuenheimer Feld 267,

69120 Heidelberg Germany

c. 3P Instruments GmbH & Co. KG, Bitterfelder Str. 1-5, 04129 Leipzip (Germany)

Table of Content

1. General Remarks **S2**
2. Synthetic Procedures **S3**
3. NMR Spectra **S17**
DOSY-NMR-Spectroscopy S63
4. Mass Spectrometry **S65**
5. IR Spectroscopy **S72**
6. Single Crystal X-ray Diffraction (SCXRD) **S80**
CF_3-cage S80
C_2F_5-cage S81
C_3F_7-cage S82
C_5F_{11}-cage S83
C_6F_{13}-cage S84
$(C_3F_8)_3 \subset C_3F_7$-cage S85
7. Isomorphism and Structural Details in the Solid State **S86**
8. Powder X-ray Diffraction (PXRD) **S87**
9. Gas Sorption **S89**
Gases S89
Non-ideality factors S89
Selectivity Calculations S92
N_2-Sorption at 77 K S94
Ar-Sorption at 87 K S97
CO_2-Sorption at 195 K S100
Gas Sorption at 273 K S102
Gas Sorption at 283 K S116
Gas Sorption at 298 K S130
Gas Sorption at 313 K S144
Gas Uptake Summary S156
Gas Selectivity Summary S157
IAST Selectivity at 273 K S160
IAST Selectivity Curves at 283 K S169
IAST Selectivity Curves at 298 K S178
IAST Selectivity Curves at 313 K S187
Isosteric Enthalpies of Adsorption S196
Breakthrough Experiments S212
10. Stability Investigations **S214**
11. Quantumchemical Calculations **S216**
12. Literature Comparison **S227**
13. References **S227**

1. General Remarks

Commercially available reagents were obtained from Chempur, Fisher Scientific, abcr, Alfa Aesar, Sigma-Aldrich or VWR and have been used without further purification unless otherwise mentioned. 2,7,14-triaminotriptycene **1**[S1] 2-Hydroxy-5-(4,4,5,5-tetramethyl-1,3,2-dioxaborolan-2-yl)benzaldehyde **S1**,S[S2] **1,4-**dibromo-2,5-bis(trifluoromethyl)benzene **S2**,[S3] 4,4''-dihydroxy-2',5'-bis(perfluorobutyl)-[1,1':4',1''-terphenyl]-3,3''-dicarbaldehyde (**2c**),[S4] **C_4F_9-cage**[S4] and dibromide **S13**[S5] were synthesized according to procedures reported in literature.

Melting points (not corrected) were measured with a Büchi Melting Point B-545. IR-spectra were recorded on a Bruker Tensor 27 spectrometer on a ZnSe ATR crystal. NMR spectra were taken on a Bruker DRX 300 (300 MHz), Bruker Avance 300 III (300 MHz), Bruker Avance III 400 (400 MHz), and Bruker Avance III 600 (600 MHz) spectrometer. NMR spectra were internally referenced to residual solvent peaks in $CDCl_3$ (^{1}H-NMR: δ = 7.26 ppm, ^{13}C-NMR: 77.2 ppm), or THF-d_8 (^{1}H-NMR: δ = 1.72, 3.58 ppm, ^{13}C-NMR: 67.2, 25.3 ppm). HRMS experiments were carried out on a Fourier Transform Ion Cyclotron Resonance (FTICR) mass spectrometer solariX (Bruker Daltonik GmbH, Bremen, Germany) equipped with a 7.0 T superconducting magnet and interfaced to an Apollo II Dual ESI/MALDI source. MALDI-ICR MS experiments of cages were carried out on a Bruker ApexQe FT-ICR instrument with DCTB (trans-2-[3-(4-tert-butylphenyl)-2-methyl-2-propenylidene]malononitrile) as matrix. Electron micrographs were acquired using a Crossbeam 540 field emission scanning electron microscope (Carl Zeiss Microscopy, Germany). TGA was measured on a Mettler-Toledo TGA/DSC1 instrument with a TGA/DSC-Sensor 1100 equipped with a MX1 balance (Mettler-Toledo) and a GC100 gas control box for nitrogen supply. Crystal structure analysis was accomplished on a STOE Stadivari diffractometer with a copper source (λ CuKα = 1.54178 Å). PXRD diffractograms were recorded using a *Rigaku SmartLab* diffractometer (9 kW) containing a *HyPix-3000* detector. For measurements CuK_α radiation (λ =1.54059 Å) in combination with a Debye–Scherrer geometry was used. Samples were filled in glass mark tubes (diameter: 0.6 mm) and attached to a capillary spin stage (60 rpm rotation). Background measurements of empty tubes have been conducted prior to sample measurements and were subtracted from the corresponding measurements. Elemental analysis was performed by the Microanalytical Laboratory of the University of Heidelberg using an Elementar Vario EL machine.

2. Synthetic Procedures

4,4''-Dihydroxy-2',5'-bis(trifluoromethyl)-[1,1':4',1''-terphenyl]-3,3''-dicarbaldehyde (2a)

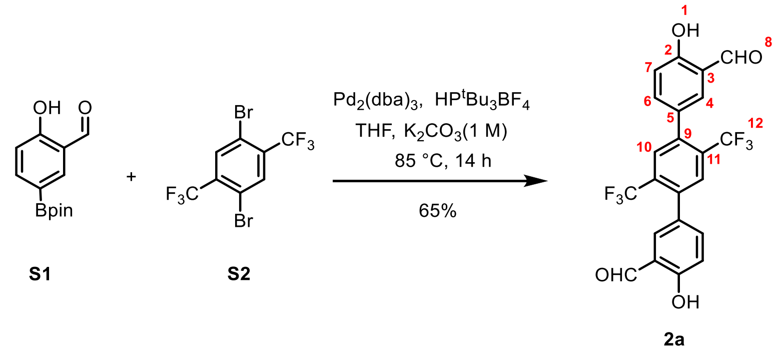

In a screw-capped vessel salicylaldehyde **S1** (2.00 g, 5.38 mmol) and dibromide **S2** (3.20 g, 12.9 mmol) were suspended in THF (32 mL) and an aqueous K_2CO_3 solution (1 M, 8 mL) under argon atmosphere. Tris(dibenzylideneacetone)-dipalladium(0) (246 mg, 269 µmol, 5 mol-%) and tri-*tert*-butylphosphonium tetrafluoroborate (156 mg, 538 µmol, 10 mol-%) were added to the mixture. The resulting suspension was heated to 85 °C and stirred for 14 hours. After cooling to room temperature, THF was removed by rotary evaporation. The residual aqueous suspension was poured on water (40 mL), extracted with CH_2Cl_2 (3 × 30 mL) and the combined organic layer was dried over Na_2SO_4. After removal of solvents under reduced pressure, the obtained crude solid was washed with n-hexane (15 mL) and recrystallized from a DCM/hexane mixture to give **2a** as light yellow solid in 65% yield (1.60 g, 3.52 mmol). Mp = 215 °C. ^{1}H NMR (400 MHz, $CDCl_3$): δ = 11.13 ppm (s, 2H, H-1), 9.95 (s, 2H, H-8), 7.75 (s, 2H, H-10), 7.58 (d, J = 2.3 Hz, 2H, H-4), 7.55 (dd, J = 8.5, 2.4 Hz, 4H, H-6), 7.11 (d, J = 8.5 Hz, 2H, H-7). ^{13}C NMR (101 MHz, $CDCl_3$): δ = 196.4 ppm (C-8), 161.9 (C-2), 139.5 (C-9), 137.4 (C-6), 134.0 (C-4), 131.9 (q, $^2J_{C\text{-}F}$ = 30.6 Hz, C-11), 130.6 (q, $^3J_{C\text{-}F}$ = 5.4 Hz, C-10), 129.7 (C-5), 123.3 (q, $^1J_{C\text{-}F}$ = 274.8 Hz, C-12), 120.3 (C-3), 118.0 (C-7). ^{19}F NMR (283 MHz, $CDCl_3$): δ = -57.5 ppm (F-C12). IR (neat, ATR): $\tilde{\nu}$ = 1684 (w), 1655 (s), 1622 (m), 1591 (m), 1558 (w), 1541 (w), 1518 (w), 1508 (w), 1475 (m), 1458 (w), 1439 (w), 1420 (w), 1400 (w), 1377 (m), 1344 (w), 1310 (m), 1288 (s), 1261 (m), 1238 (m), 1232 (m), 1138 (s), 1090 (s), 1038 (s), 961 (w), 934 (m), 910 (s), 841 (s), 766 (s), 750 (s), 735 (s), 702 (s), 648 (s) cm^{-1}. MS (EI+): [M]$^+$: *m/z* Calcd. For ($C_{22}H_{12}F_6O_4^+$): 454.0640, found 454.0634. Elemental Analysis (%): ($C_{22}H_{12}F_6O_4$) Calcd. C 58.16, H 2.66; found C 58.15, H 2.93.

1,4-Bis(perfluoroethyl)benzene (S5)[S6]

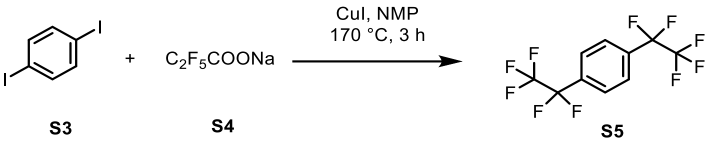

According to a literature known procedure,[S6] a 500 mL flask was charged with 1,4-diiodobenzene **S3** (9.90 g, 30.0 mmol), sodium pentafluoropropionate **S4** (44.6 g, 240 mmol), CuI (22.9 g, 120 mmol) and anhydrous toluene (80 mL) under argon. Toluene and water were removed by distillation at 120 °C under argon flow. The mixture was cooled down to room temperature and anhydrous *N*-methyl-2-pyrrolidone (NMP; 140 mL) was added. The mixture was heated to 170 °C and stirred for 3 h. After cooling down to 55 °C, the product was obtained under reduced pressure to give **S5** as colorless liquid (4.69 g, 14.9 mmol). **S5** was used in the next step without further purification.

1,4-Dibromo-2,5-bis(perfluoroethyl)benzene (S6)

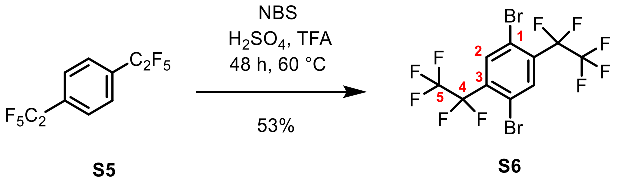

1,4-Bis(perfluoroethyl)benzene (S5) (1.26 g, 4.00 mmol) was added to a stirring mixture of trifluoroacetic acid (10 mL) and concentrated sulfuric acid (3 mL). The mixture was stirred at 60 °C, and *N*-bromosuccinimide (NBS) (2.13 g, 12.0 mmol) was added in portions (6 × 355 mg) over 5 h. After stirring for additional 48 hours at 60 °C, the mixture was cooled to room temperature and ice water (100 mL) was added. The resulting suspension was filtered, washed with water (40 mL × 3) and sublimated to give **S6** as colorless solid in 53 % yield (1.00 g, 2.12 mmol). Mp = 61 °C. ^{1}H NMR (600 MHz, $CDCl_3$): δ = 7.95 ppm (s, 2H, H-2). ^{13}C NMR (151 MHz, $CDCl_3$) δ = 137.1 ppm (t, $^{3}J_{C\text{-}F}$ = 8.8 Hz, C-2), 133.0(t, $^{2}J_{C\text{-}F}$ = 23.3 Hz, C-3), 120.0(t, $^{4}J_{C\text{-}F}$ = 2.4 Hz, C-1), 118.8(qt, $^{1}J_{C\text{-}F}$ = 287.3 Hz, $^{2}J_{C\text{-}F}$ = 37.5 Hz, C-5), 112.1(tq, $^{1}J_{C\text{-}F}$ = 258.8 Hz, $^{2}J_{C\text{-}F}$ = 40.2 Hz, C-4). ^{19}F NMR (471 MHz, $CDCl_3$): δ = -82.8 ppm (6F, F-C5), -111.7 (4F, F-C4). IR (neat, ATR): $\tilde{\nu}$ = 1487 (w), 1348 (m), 1333 (m), 1321 (s), 1277 (m), 1211 (s), 1188 (s), 1146 (s), 1113 (s), 1069 (vs), 1015 (w), 968 (s), 897 (s), 829 (w), 800 (w),

752 (s), 727 (w), 714 (w), 692 (m), 679 (w) cm^{-1}. MS (EI+): $[M]^+$: *m/z* Calcd. For ($C_{10}H_2F_{10}Br_2^+$): 469.8364, found 469.8340. Elemental Analysis (%): ($C_{10}H_2F_{10}Br_2$) Calcd. C 25.45, H 0.43; found C 25.41, H 0.67.

4,4''-Dihydroxy-2',5'-bis(perfluoroethyl)-[1,1':4',1''-terphenyl]-3,3''-dicarbaldehyde (2b)

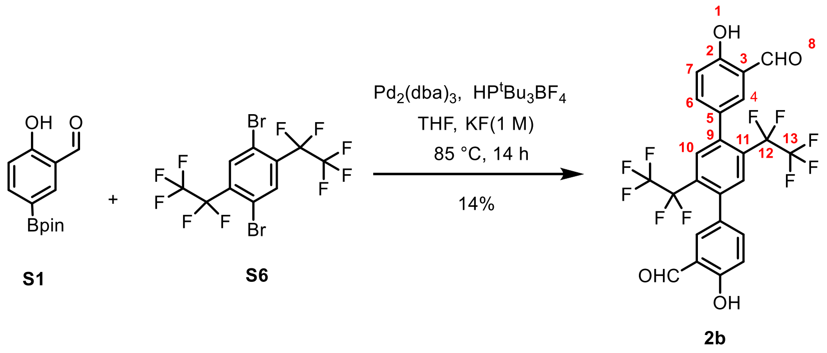

In a screw-capped vessel salicylaldehyde **S1** (472 mg, 2.40 mmol) and dibromide **S6** (572 mg, 1.00 mmol) were suspended in THF (40 mL) and an aqueous KF solution (1 M, 10 mL) under argon. Tris(dibenzylideneacetone)dipalladium(0) (91.6 mg, 10 µmol, 5 mol-%) and tri-*tert*-butylphosphonium tetrafluoroborate (58.0 mg, 20 µmol, 10 mol-%) were added and the mixture stirred at 85 °C for 14 h. After cooling the reaction mixture to room temperature, the organic layer was separated and dried over Na_2SO_4. After removal of the solvents under reduced pressure, the crude product was purified by column chromatography (SiO_2, flushed with THF) and recrystallized further from THF/pentane to give **2b** as colorless solid in 62% yield (342 mg, 617 µmol). Mp = 276 °C. ^{1}H NMR (700 MHz, THF-*d$_8$*): δ = 10.99 ppm (s, 2H, H-1), 10.01 (s, 2H, H-8), 7.73 (s, 2H, H-10), 7.68 (d, *J* = 2.3 Hz, ^{1}H, H-4), 7.51 (dd, *J* = 8.3, 2.3 Hz, 2H, H-6), 7.04 (d, *J* = 8.5 Hz, 2H, H-7). ^{13}C NMR (176 MHz, THF-*d$_8$*): δ = 196.4 ppm (C-8), 162.1 (C-2), 142.0 (C-9), 137.7 (C-6), 134.2 (C-4), 133.7(C-10), 130.4 (C-5), 130.3 (C-11), 121.3 (C-3), 117.5 (C-7), 122.3-112.6 (m, C-12/13). ^{19}F NMR (283 MHz, THF-*d$_8$*): δ = -86.2 ppm (6F, F-13), -109.5(4F, F-C12). IR (neat, ATR): $\tilde{\nu}$ = 1655 (s), 1624 (w), 1589 (w), 1477 (m), 1441 (w), 1389 (w), 1377 (w), 1331 (m), 1308 (m), 1286 (s), 1258 (w), 1194 (s), 1165 (s), 1148 (s), 1097 (s), 1055 (m), 1018 (w), 980 (s), 930 (s), 908 (m), 895 (w), 845 (m), 771 (m), 741 (vs), 706 (s), 646 (s), 613 (m) cm^{-1}. MS (EI+): $[M]^+$: *m/z* Calcd. for ($C_{24}H_{12}F_{10}O_4^+$): 554.0576, found 554.0559. Elemental Analysis (%): ($C_{24}H_{12}F_{10}O_4$) Calcd. C 52.00, H 2.18; found C 52.13, H 2.56.

1,4-Bis(perfluoropropyl)benzene (S8)[S7]

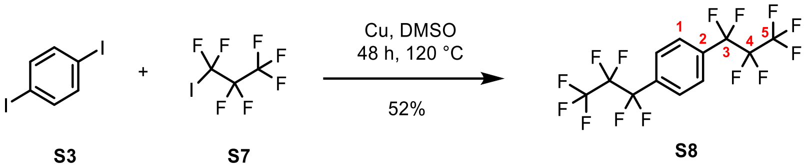

According to a literature known procedure,[S7] in a screw-capped vessel perfluoropropyl iodide **S7** (26.6 g, 90.0 mmol) was added to a mixture of 1,4-diiodobenzene **S3** (9.90 g, 30.0 mmol) and Cu powder (11.4 g, 180 mmol) in anhydrous DMSO (80 mL). The mixture was heated to 120 °C and stirred for 48 hours. After cooling to room temperature, water (50 mL) and diethyl ether (50 mL) were added to the mixture and stirred additional 30 mins. The mixture was filtered and the filtrate was extracted with diethyl ether (3 × 50 mL). The combined extract was washed with water (2 × 40 mL) and dried over $MgSO_4$. After removal of solvent under reduced pressure, the resulting residue was purified by column chromatography (SiO_2, petroleum ether, R_f = 0.68) to give **S8** as colorless solid in 52% yield (6.47 g, 15.6 mmol). Mp = 32 °C. ^{1}H NMR (600 MHz, $CDCl_3$): δ = 7.76 ppm (s, 4H, H-1). ^{13}C NMR (151 MHz, $CDCl_3$): δ = 132.8 ppm (t, $^1J_{C-F}$ = 24.6 Hz, C-2), 127.5 (t, $^2J_{C-F}$ = 6.4 Hz, C-1), 118.1 (qt, $^1J_{C-F}$ = 287.7 Hz, $^2J_{C-F}$ = 34.0 Hz, C-5), 114.9 (tt, $^1J_{C-F}$ = 255.9 Hz, $^2J_{C-F}$ = 31.3 Hz, C-3), 108.9 (m, C-4). ^{19}F NMR (471 MHz, $CDCl_3$): δ = -80.0 ppm (6F, F-C5), -112.3 (4F, F-C3), -126.3 (4F, F-C4). IR (neat, ATR): $\tilde{\nu}$ = 1418 (w), 1346 (s), 1277 (m), 1227 (s), 1194 (s), 1177 (s), 1151 (s), 1105 (s), 1082 (s), 1024 (w), 897 (s), 814 (s), 741 (s), 685 (s), 608 (m) cm^{-1}. MS (EI+): $[M]^+$: *m/z* Calcd. For ($C_{12}H_4F_{14}^+$): 414.0084, found 414.0069. Elemental Analysis (%): ($C_{12}H_4F_{14}$) Calcd. C 34.80, H 0.97; found C 34.81, H 1.44.

1,4-Dibromo-2,5-bis(perfluoropropyl)benzene (S9)[S7]

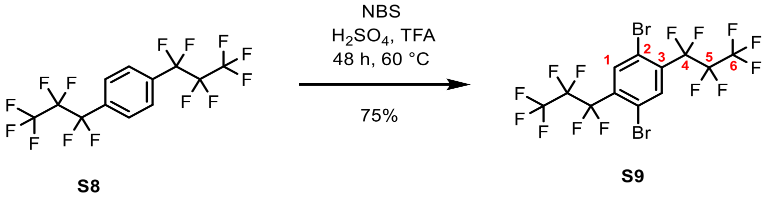

According to a literature known procedure,S[7] 1,4-bis(perfluoropropyl)benzene **(S8)** (9.18g, 22.2 mmol) was added to a mixture of trifluoroacetic acid (85 mL) and

concentrated sulfuric acid (33 mL). The mixture was stirred at 60 °C and *N*-bromosuccinimide (NBS) (11.9 g, 66.6 mmol) was added in portions (6 × 1.98 g) over 5 h. After stirring for additional 48 hours at 60 °C, the mixture was cooled to room temperature and ice water (600 mL) was added. The resulting suspension was filtered, washed with water (3 × 40 mL) and recrystallized from ethanol to give **S9** as colorless solid in 75% yield (9.50 g, 16.6 mmol). Mp = 80 °C. ^{1}H NMR (600 MHz, $CDCl_3$) δ = 7.93 ppm (s, 2H, H-1). ^{13}C NMR (151 MHz, $CDCl_3$): δ = 137.5 ppm (t, $^{1}J_{C\text{-}F}$ = 9.0 Hz, C-1), 133.2 (t, $^{1}J_{C\text{-}F}$ = 23.6 Hz, C-3), 120.3 (s, C-2), 118.0 (qt, $^{1}J_{C\text{-}F}$ = 287.9 Hz, $^{2}J_{C\text{-}F}$ = 33.7 Hz, C-6), 114.3 (tt, $^{1}J_{C\text{-}F}$ = 259.7 Hz, $^{2}J_{C\text{-}F}$ = 33.3 Hz, C-4), 109.3 (m, C-5). ^{19}F NMR (471 MHz, $CDCl_3$) δ = -80.0 ppm (6F, F-C6), -108.5 (4F, F-C4), -123.9 (4F, F-C5). IR (neat, ATR): $\tilde{\nu}$ = 1483 (w), 1352 (m), 1339 (m), 1277 (m), 1221 (s), 1180 (s), 1146 (s), 1119 (s), 1107 (s), 1047 (s), 916 (s), 866 (s), 744 (s), 706 (m), 623 (m) cm^{-1}. MS (EI+): $[M]^+$: *m/z* Calcd. For ($C_{12}H_2Br_2F_{14}^+$): 569.8300, found 569.8312. Elemental Analysis (%): ($C_{12}H_2Br_2F_{14}$) Calcd. C 25.20, H 0.35; found C 25.18, H 0.71.

4,4''-Dihydroxy-2',5'-bis(trifluoromethyl)-[1,1':4',1''-terphenyl]-3,3''-dicarbaldehyde(2c)

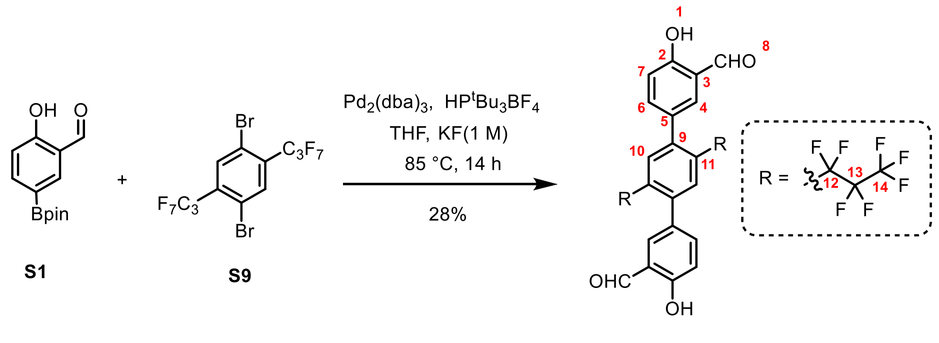

In a screw-capped vessel, salicylaldehyde **S1** (595 mg, 2.40 mmol) and dibromide **S9** (572 mg, 1.00 mmol) were suspended in THF (40 mL) and an aqueous KF solution (1 M, 10 mL) under argon. Tris(dibenzylideneacetone)dipalladium(0) (91.6 mg, 10 µmol, 5 mol-%) and tri-*tert*-butylphosphonium tetrafluoroborate (58.0 mg, 20 µmol, 10 mol-%) were added and the mixture stirred at 85 °C for 14 hours. After cooling the reaction mixture to room temperature, the organic layer was separated and dried over Na_2SO_4. After removal of the solvents under reduced pressure, the crude product was purified by column chromatography (SiO_2, flushed with THF) and recrystallized further from THF/n-pentane to give **2e** as colorless solid in 38% yield (251 mg, 384 µmol). Mp

= 232 °C. ^{1}H NMR (600 MHz, THF-d_8): δ = 10.99 ppm (s, 2H, H-1), 10.02 (s, 2H, H-8), 7.73 (s, 2H, H-10), 7.69 (d, J = 2.3 Hz, ^{1}H, H-4), 7.52 (dd, J = 8.7, 2.5 Hz, 2H, H-6), 7.04 (d, J = 8.5 Hz, 2H, H-7). ^{13}C NMR (126 MHz, THF-d_8): δ = 196.4 ppm (C-8), 162.1 (C-2), 142.1 (C-9), 137.8 (C-6), 134.2 (C-4), 134.1(C-10), 130.4 (C-5), 130.3(C-11), 121.2 (C-3), 117.4 (C-7). *Note*: Due to strong and multiple ^{19}F-^{13}C couplings, the signals of the perfluorinated side chains were not visible in the ^{13}C NMR spectrum but could be extracted from 2D NMR spectra. ^{19}F NMR (471 MHz, THF-d_8): δ = -82.9 ppm (6F), -106.5 (4F), -126.6 (4F). IR (neat, ATR): $\tilde{\nu}$ = 1661 (m), 1593 (w), 1477 (m), 1371 (w), 1344 (m), 1306 (w), 1288 (s), 1258 (w), 1221 (s), 1204 (s), 1173 (s), 1153 (s), 1132 (m), 1115 (vs), 1094 (m), 1047 (w), 947 (m), 916 (m), 880 (s), 839 (m), 770 (m), 752 (s), 733 (s), 712 (m), 663 (w), 633 (m), 608 (w) cm^{-1}. MS (EI+): $[M]^+$: m/z Calcd. for ($C_{26}H_{12}F_{14}O_4^+$): 654.0512, found 654.0478. Elemental Analysis (%): ($C_{26}H_{12}F_{14}O_4$ × 0.6 THF) Calcd. C 47.99, H 2.13; found C 48.19, H 2.19.

1,4-Bis(perfluoropentyl)benzene (S11)

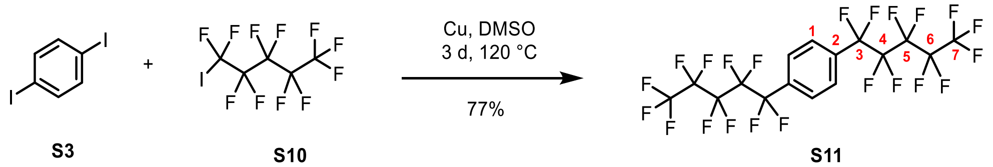

In a screw-capped vessel perfluoropropyl iodide **S10** (22.6 g, 57.2 mmol) was added to a mixture of 1,4-diiodobenzene **S3** (8.58 g, 26.0 mmol) and Cu powder (6.61 g, 104 mmol) in anhydrous DMSO (80 mL). The mixture was heated to 120 °C and stirred for 3 days. After cooling to room temperature, water (60 mL) and diethyl ether (150 mL) were added to the mixture and stirred additional 20 min. The mixture was filtered and the filtrate was extracted with diethyl ether (2 × 40 mL). The combined extract was washed with water (2 × 40 mL) and dried over $MgSO_4$. After removal of the solvent under reduced pressure, the resulting residue was purified by column chromatography (SiO_2, petroleum ether, R_f = 0.77) to give **S11** as colorless solid in 77% (13.5 g, 22.0 mmol). Mp = 51 °C. ^{1}H NMR (400 MHz, $CDCl_3$) δ = 7.76 ppm (s, 4H, H-1). ^{13}C NMR (101 MHz, $CDCl_3$) δ = 133.0 ppm (t, $^{1}J_{C\text{-}F}$ = 24.5 Hz, C-2), 127.6 (t, $^{2}J_{C\text{-}F}$ = 6.6 Hz, C-1) 122.0-105.0 (m, C-3/4/5/6). ^{19}F NMR (283 MHz, $CDCl_3$) δ = -80.9 ppm (6F), -111.4 (4F), -122.0(4F), -122.3(4F), -126.3(4F). IR (neat, ATR): $\tilde{\nu}$ = 1417 (w), 1359 (m), 1297 (m), 1232 (s), 1198 (s), 1186 (s), 1137 (vs), 1104 (s), 1084 (s), 1025 (m), 957 (w), 864

(w), 846 (m), 771 (m), 747 (w), 720 (s), 702 (s), 661 (m), 616 (m) cm^{-1}. MS (EI+): $[M]^+$: *m/z* Calcd. for ($C_{16}H_4F_{22}^+$): 594.9972, found 594.9947. Elemental Analysis (%): ($C_{16}H_4F_{22}$) Calcd. C 31.29, H 0.66; found C 31.76, H 1.12.

1,4-Dibromo-2,5-bis(perfluoropentyl) benzene (S12)

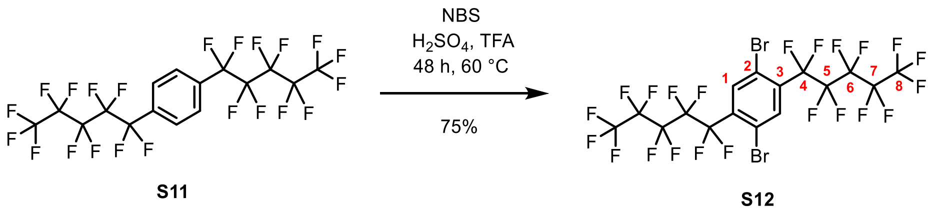

S11 (12.3 g, 20 mmol) was added to a stirred solution of trifluoroacetic acid (100 mL), and concentrated H_2SO_4 (30 mL). The reaction mixture was heated to 60 °C and *N*-bromosuccinimide (10.7 g, 60 mmol) was added in portions (6 × 1.78 g) over 5 hours. After stirring for additional 48 hours at 60 °C, the mixture was cooled to room temperature and ice water (100 mL) was added. The precipitate was collected, washed with water (200 mL), redissolved with dichloromethane (500 mL) and dried over $MgSO_4$. After removal of the solvent under reduced pressure, the resulting residue was recrystallized from ethanol to give **S12** as colorless solid in 75% yield (11.6 g, 15.0 mmol). Mp = 88 °C. ^1H NMR (700 MHz, $CDCl_3$) δ = 7.94 ppm (s, 4H). ^{13}C NMR (176 MHz, $CDCl_3$): δ = 137.5 ppm (t, $^3J_{C-F}$ = 9.1 Hz, C-1), 133.2 (t, $^2J_{C-F}$ = 23.6 Hz, C-3), 120.3 (C-2), 117.4 (qt, $^1J_{C-F}$ = 288.3 Hz, $^2J_{C-F}$ = 33.1 Hz, C-8), 114.9 (tt, $^1J_{C-F}$ = 261.3 Hz, $^2J_{C-F}$ = 33.8 Hz, C-4), 113.0-106.0 (m, C-5/6/7). ^{19}F NMR (283 MHz, $CDCl_3$) δ = -80.7 ppm (6F), -107.7 (4F), -119.5 (4F), -122.4 (4F), -126.1 (4F). IR (neat, ATR): $\tilde{\nu}$ =1481 (w), 1359 (m), 1346 (m), 1309 (w), 1285 (w), 1229 (s), 1197 (s), 1137 (s), 1106 (s), 1052 (s), 975 (w), 957 (m), 901 (m), 864 (w), 840 (w), 805 (m), 783 (s), 746 (m), 723 (s), 671 (s), 651 (w), 635 (w) cm^{-1}. MS (EI+): $[M]^+$: *m/z* Calcd. for ($C_{16}H_2Br_2F_{22}^+$): 769.8167, found 769.8168. Elemental Analysis (%): ($C_{16}H_2Br_2F_{22}$) Calcd. C 24.89, H 0.26; found C 25.39, H 0.80.

4,4''-Dihydroxy-2',5'-bis(perfluoropentyl)-[1,1':4',1''-terphenyl]-3,3''-dicarbaldehyde(2e)

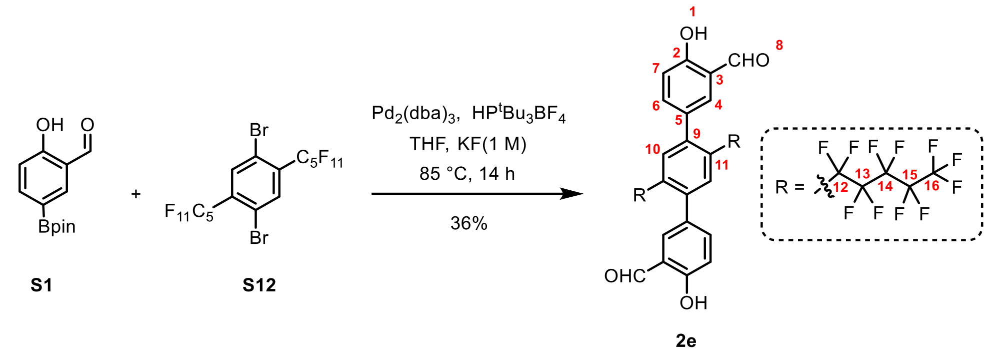

In a screw-capped vessel, salicylaldehyde **S1** (595 mg, 2.40 mmol) and dibromide **S12** (772 mg, 1.00 mmol) were suspended in a mixture of THF (40 mL) and an aqueous KF solution (1 M, 10 mL) under argon. Tris(dibenzylideneacetone)dipalladium(0) (91.6 mg, 10 µmol, 5 mol-%) and tri-*tert*-butylphosphonium tetrafluoroborate (58.0 mg, 20 µmol, 10 mol-%) were added and the mixture was stirred at 85 °C for 14 hours. After cooling the reaction mixture to room temperature, the organic layer was separated and dried over Na_2SO_4. After removal of the solvents under reduced pressure, the crude product was purified by column chromatography (SiO_2, petroleum ether/ethyl acetate = 5:1, R_f = 0.59, flushed with THF) and recrystallized from THF/n-pentane to give **2e** as colorless solid in 36% yield (310 mg, 363 µmol). Mp = 210 °C. ^{1}H NMR (600 MHz, THF-d_8): δ = 11.00 ppm (s, 2H, H-1), 10.03 (s, 2H, H-8), 7.74 (s, 2H, H-10), 7.69 (d, J = 2.5 Hz, 2H, H-4), 7.52 (dd, J = 8.5, 2.5 Hz, 2H, H-6), 7.05 (d, J = 8.5 Hz, 2H, H-7). ^{13}C NMR (151 MHz, THF-d_8): δ = 196.2 ppm (C-8), 162.1 (C-2), 142.2 (C-9), 137.7 (C-6), 134.2 (C-10), 134.1 (C-4), 130.4 (C-5/11), 121.3 (C-3), 117.4 (C-7). *Note*: Due to strong and multiple ^{19}F-^{13}C coupling, the signals of the perfluorinated side chains were not visible in the ^{13}C NMR spectrum and were extracted from 2D NMR data. ^{19}F NMR (283 MHz, THF-d_8): δ = -83.7 ppm (6F), -105.8 (4F), -122.0 (4F), -124.9 (4F), -128.7 (4F). IR (neat, ATR): $\tilde{\nu}$ = 1664 (m), 1623 (w), 1593 (w), 1477 (m), 1371 (w), 1357 (m), 1306 (w), 1287 (m), 1251 (m), 1233 (m), 1192 (s), 1165 (s), 1140 (vs), 1110 (s), 1093 (m), 1049 (w), 936 (w), 909 (w), 883 (w), 861 (w), 836 (m), 822 (w), 789 (m), 770 (m), 746 (m), 719 (s), 693 (m), 678 (m), 656 (m), 638 (w), 604 (m) cm^{-1}. MS (APCI+): [M+H]$^+$: *m/z* Calcd. for ($C_{30}H_{13}F_{22}O_4^+$): 855.0457, found 855.0412. Elemental Analysis (%): ($C_{30}H_{12}F_{22}O_4$) Calcd. C 42.17, H 1.42; found C 42.04, H 1.93.

4,4''-Dihydroxy-2',5'-bis(perfluorohexyl)-[1,1':4',1''-terphenyl]-3,3''-dicarbaldehyde (2f)

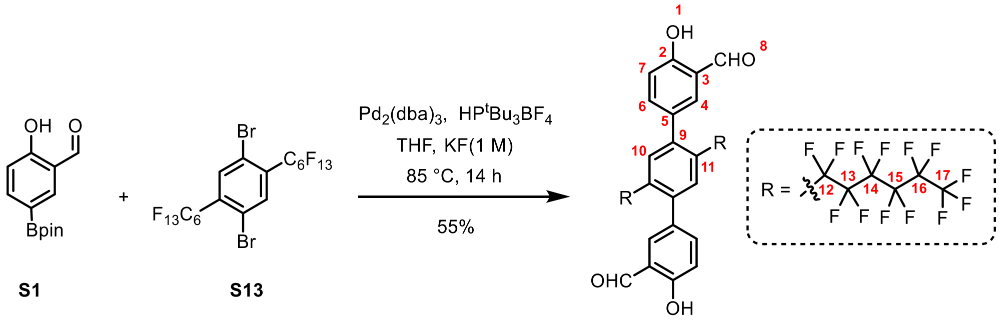

In a screw-capped vessel salicylaldehyde **S1** (1.43 g, 5.78 mmol) and dibromide **S13** (2.10 g, 2.41 mmol) were suspended in THF (40 mL) and an aqueous KF solution (1 M, 10 mL) under argon. Tris(dibenzylideneacetone)dipalladium(0) (111 mg, 121 µmol, 5 mol-%) and tri-*tert*-butylphosphonium tetrafluoroborate (69.9 mg, 241 µmol, 10 mol-%) were added and the mixture stirred at 85 °C for 14 hours. After cooling the reaction mixture to room temperature, the organic layer was separated and dried over Na_2SO_4. After removal of the solvents under reduced pressure, the crude product was purified by column chromatography (SiO_2, petroleum ether/ethyl acetate = 10:1, R_f = 0.32, flushed with THF) and recrystallized further from THF/n-pentane to give **2f** as colorless solid in 55% yield (1.26 g, 1.32 mmol). Mp = 195 °C. ^{1}H NMR (600 MHz, THF-d_8): δ = 11.00 ppm (s, 2H, H-1), 10.02 (s, 2H, H-8), 7.74 (s, 2H, H-10), 7.69 (d, J = 2.5 Hz, 2H, H-4), 7.52 (dd, J = 8.5, 2.6 Hz, 2H, H-6), 7.04 (d, J = 8.5 Hz, 2H, H-7). ^{13}C NMR (151 MHz, THF-d_8): δ = 196.4 ppm (C-8), 162.1 (C-2), 142.2 (C-9), 137.7 (C-6), 134.3 (C-10/4), 130.4 (C-5/11), 121.2 (C-3), 117.5 (C-7). *Note*: Due to strong and multiple ^{19}F-^{13}C coupling, the signals of the perfluorinated side chains were not visible in the ^{13}C NMR spectrum and were extracted from 2D NMR spectra. ^{19}F {^{1}H} NMR (471 MHz, THF-d_8): δ = -81.8 ppm (6F), -103.9 (4F), -120.0 (4F), -122.2 (4F), -123.4 (4F), -126.8 (4F). IR (neat, ATR): $\tilde{\nu}$ = 1661 (m), 1622 (w), 1591 (w), 1477 (m), 1366 (w), 1306 (w), 1286 (s), 1232 (m), 1198 (vs), 1167 (s), 1142 (s), 1123 (s), 1103 (m), 1063 (m), 1032 (m), 1020 (w), 959 (w), 945 (w), 916 (m), 837 (m), 770 (m), 754 (m), 743 (m), 714 (s), 679 (s), 665 (m), 646 (m), 621 (m) cm^{-1}. MS (EI+): [M]$^+$: *m/z* Calcd. For ($C_{32}H_{12}F_{26}O_4^+$): 954.0320, found 954.0336. Elemental Analysis (%): ($C_{32}H_{12}F_{26}O_4$) Calcd. C 40.27, H 1.27 found C 40.15, H 1.67.

CF_3-cage:

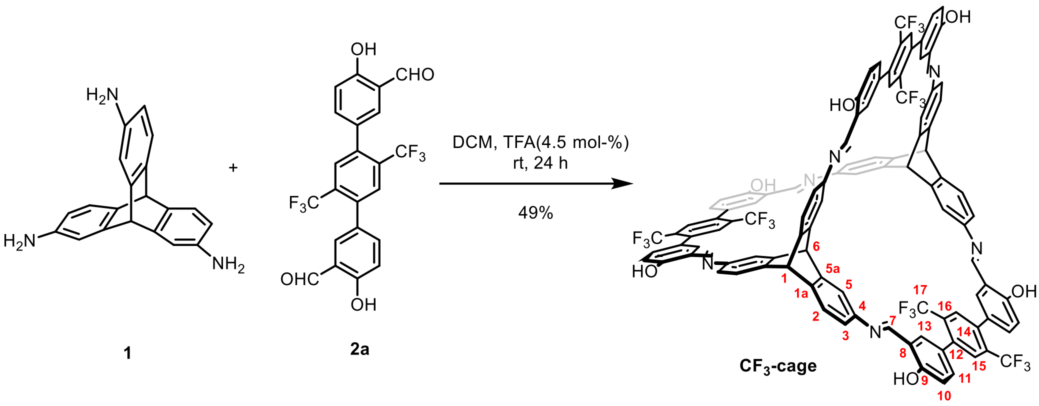

In six individual reaction setups, triamino triptycene **1** (29.9 mg, 100 µmol) and salicylaldehyde **2a** (68.1 mg, 150 µmol) were dissolved in anhydrous DCM (20 mL each) in six 25 mL screw-capped vessels and 4.5 mol-% TFA (0.1 M in anhydrous DCM, 45 µL) was added to each of the solutions. The mixtures were stirred at room temperature for 24 hours. All precipitates was filtered, combined and washed with n-pentane (3 × 3 mL) and diethyl ether (2 × 3 mL), dried on a Kugelrohr oven (100 °C, $7.0 \cdot 10^{-2}$ mbar) for 14 hours to give **CF_3-cage** as light yellow solid in 49% yield (272 mg, 147 µmmol). Mp >350 °C. ^{1}H NMR (600 MHz, THF-d_8): δ = 13.37 ppm (s, 6H,9-OH), 9.16 (s, 6H, imine-H-7), 7.88 (s, 6H, H-15), 7.83 (d, J = 2.1 Hz, 6H, H-5), 7.56 (d, J = 7.8 Hz, 6H, H-2), 7.51 – 7.44 (m, 12H, H-11/13), 7.24 (dd, J = 7.8, 2.1 Hz, 6H, H-3), 7.06 (d, J = 8.1 Hz, 6H, H-10), 5.72 (s, 2H, bridgehead-H-1), 5.63 (s, 2H, bridgehead-H-6). ^{13}C NMR (151 MHz, THF-d_8): δ = 162.3 ppm (C-9), 160.0 (C-7), 146.8 (C-5a), 145.3 (C-1a), 144.3 (C-4), 141.0 (C-14), 133.8 (C-11), 133.7 (C-13), 131.9 (q, $^1J_{C\text{-}F}$ = 30.4 Hz, C-16), 131.3 (C-15), 129.5 (C-12), 125.8 (C-3), 125.5 (C-2), 124.4 (q, $^1J_{C\text{-}F}$ = 274.5 Hz, C-17), 119.8 (C-8), 117.7 (C-10), 111.5 (C-5), 56.0 (bridgehead-C-6), 53.3 (bridgehead-C-1). ^{19}F NMR (471 MHz, THF-d_8): δ = -59.9 ppm (18F, F-C17). IR (neat, ATR): $\tilde{\nu}$ = 1626 (m), 1609 (m), 1582 (m), 1483 (m), 1394 (m), 1364 (w), 1294 (s), 1234 (m), 1167 (s), 1128 (s), 1086 (s), 1040 (s), 959 (m), 920 (w), 887 (m), 866 (m), 852 (m), 827 (m), 793 (m), 777 (m), 744 (m), 733 (w), 687 (w), 662 (m), 615 (w) cm^{-1}. MS (MALDI-FT-ICR): $[M+H]^+$: m/z calcd. for ($C_{106}H_{59}F_{18}N_6O_6^+$): 1853.4203, found 1853.4290. Elemental Analysis (%): ($C_{106}H_{58}F_{18}N_6O_6$) Calcd. C 68.68, H 3.15, N 4.59; found C 68.62, H 3.49, N 4.43.

C_2F_5-cage:

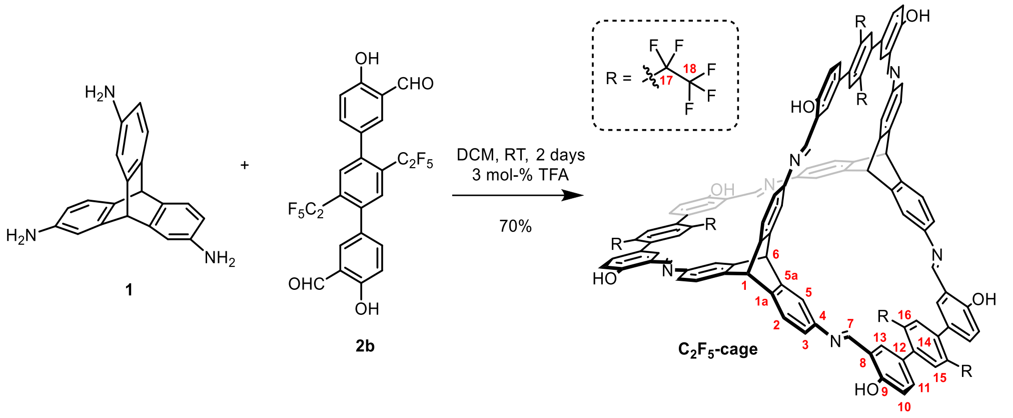

In a 250 mL flask salicylaldehyde **2b** (200 mg, 360 µmol) was stirred in anhydrous DCM (200 mL) until it was dissolved and triamino triptycene **1** (71.9 mg, 240 µmol) was subsequently added to the solution. After complete dissolvation, 3 mol-% TFA (0.1 M in anhydrous DCM, 108 µL) was added. The mixture was stirred at room temperature for 2 days. The precipitate was filtered, washed with n-pentane (10 mL) and diethyl ether (2 × 3 mL) and dried for 6 hours at the Kugelrohr oven (100 °C, $1.0 \cdot 10^{-3}$ mbar) to give **C_2F_5-cage** as light yellow solid in 70% yield (180 mg, 83.6 µmmol). M.p. >350 °C. ^{1}H NMR (600 MHz, THF-d_8): δ = 13.27 ppm (s, 6H, 9-OH), 9.12 (s, 6H, imine-H-7), 7.80 (d, J = 2.2 Hz, 6H, H-5), 7.74 (s, 6H, H-15), 7.54 (d, J = 7.7 Hz, 6H, H-2), 7.38 (d, J = 6.6 Hz, 12H, H-11/13), 7.23 (dd, J = 7.7, 2.5 Hz, 6H, H-3), 7.02 (d, J = 9.1 Hz, 6H, H-10), 5.69(s, 2H, bridgehead-H-1), 5.56(s, 2H, bridgehead-H-6). ^{13}C NMR (151 MHz, THF-d_8): δ = 162.2 ppm (C-9), 159.6 (C-7), 146.7 (C-5a), 145.4 (C-1a), 144.3 (C-4), 142.5 (C-14), 133.9 (C-11), 133.8 (C-15), 133.6 (C-13), 130.4 (t, $^1J_{C\text{-}F}$ = 21.9 Hz, C-16), 129.7 (C-12), 125.9 (C-3), 125.5 (C-2), 119.5 (C-8), 117.3 (C-10), 111.3 (C-5), 56.2 (bridgehead-C-6), 53.5 (bridgehead-C-1). ^{19}F NMR (471 MHz, THF-d_8): δ = -86.1 ppm (18F, F-C18), -109.4 (12F, F-C17). *Note*: Due to strong and multiple ^{19}F-^{13}C-coupling, the signals of the perfluorinated side chains were not visible in the ^{13}C NMR spectrum and have been extracted from 2D NMR spectra. IR (neat, ATR): $\tilde{\nu}$ = 1628 (w), 1609 (w), 1583 (w), 1477 (m), 1385 (w), 1364 (w), 1325 (w), 1279 (m), 1205 (s), 1173 (m), 1151 (m), 1130 (m), 1096 (m), 1084 (s), 1059 (w), 1009 (w), 978 (s), 961 (w), 928 (w), 916 (w), 889 (w), 860 (m), 829 (m), 793 (m), 777 (m), 744 (m), 716 (w), 687 (w), 669 (m), 615 (w) cm^{-1}. MS (MALDI-TOF): $[M+H]^+$: *m/z* calcd. for ($C_{112}H_{59}F_{30}N_6O_6^+$): 2154.4012, found 2154.4022. Elemental Analysis (%): ($C_{112}H_{58}F_{30}N_6O_6$) Calcd. C 62.46, H 2.71, N 3.90; found C 62.13, H 3.12, N 3.70.

C_3F_7-cage:

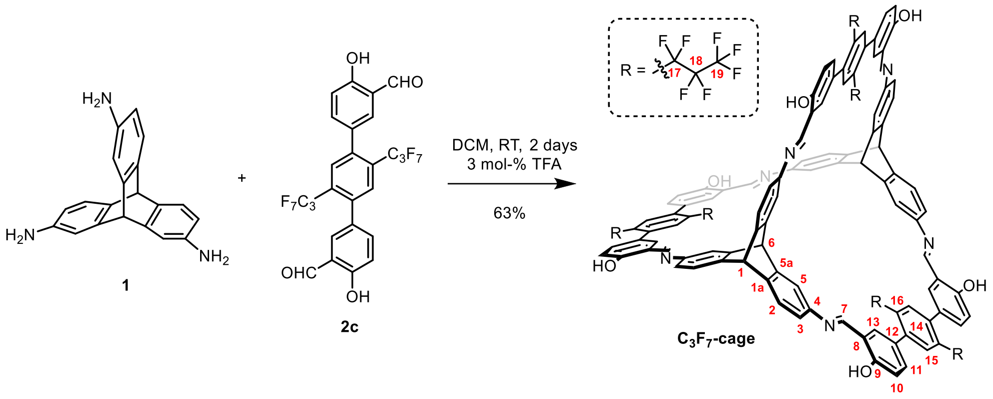

In a 250 mL flask, salicylaldehyde **2c** (232 mg, 360 µmol) was stirred in anhydrous DCM (170 mL) until it dissolved. Triamino triptycene **1** (71.9 mg, 240 µmol) was subsequently added to the solution. After complete dissolution of both compounds, 3 mol-% TFA (0.1 M in anhydrous DCM, 108 µL) was added. The mixture was stirred at room temperature for 2 days. The precipitate was filtered, washed with n-pentane (3 × 3 mL) and diethyl ether (5 × 3 mL) and dried for 4 h at the Kugelrohr oven (100 °C, $1.0 \cdot 10^{-3}$ mbar) to give **C_3F_7-cage** as light yellow solid in 63% yield (185 mg, 75.4 µmmol). Mp >350 °C. ^{1}H NMR (700 MHz, THF-d_8): δ = 13.33 ppm (s, 6H, 9-OH), 9.14 (s, 6H, imine-H-7), 7.83 (d, J = 2.2 Hz, 6H, H-5), 7.75 (s, 6H, H-15), 7.55 (d, J = 7.9 Hz, 6H, H-2), 7.40 (dd, J = 8.0, 2.5 Hz, 6H, H-11), 7.37 (d, J = 2.5 Hz, 6H, H-13), 7.24 (dd, J = 7.9, 2.1 Hz, 6H, H-3), 7.02 (d, J = 8.1 Hz, 6H, H-10), 5.71 (s, 2H, bridgehead-H-1), 5.58 (s, 2H, bridgehead-H-6). ^{13}C NMR (176 MHz, THF-d_8): δ = 162.1 ppm (C-9), 159.7 (C-7), 146.7 (C-5a), 145.3 (C-1a), 144.2 (C-4), 142.6 (C-14), 134.2 (C-15), 134.0 (C-11), 133.7 (C-13), 130.3 (C-16), 129.7 (C-12), 125.9 (C-3), 125.5 (C-2), 119.4 (C-8), 117.2 (C-10), 111.4 (C-5), 56.1 (bridgehead-C-6), 53.4 (bridgehead-C-1). *Note*: Due to strong and multiple ^{19}F-^{13}C-coupling, the signals of the perfluorinated side chains where not visible in ^{13}C-NMR spectra and have been extracted from 2D NMR spectra. ^{19}F NMR (283 MHz, THF-d_8): δ = -83.0 ppm (18F, F-C19), -106.4 (12F, F-C18), -126.4 (12F, F-C17). IR (neat, ATR): $\tilde{\nu}$ = 1629 (m), 1611 (w), 1584 (m), 1480 (m), 1382 (w), 1340 (m), 1279 (m), 1231 (s), 1201 (s), 1182 (s), 1147 (m), 1135 (m), 1129 (m), 1110 (s), 1083 (m), 1061 (w), 1048 (w), 972 (w), 958 (w), 945 (w), 933 (w), 923 (w), 915 (m), 899 (w), 890 (w), 874 (s), 863 (m), 828 (m), 809 (m), 793 (m), 779 (m), 743 (s), 678 (m), 663 (w), 652 (w), 643 (w) cm^{-1}. MS (MALDI-FT-ICR): $[M+H]^+$: *m/z* calcd. for ($C_{118}H_{59}F_{42}N_6O_6^+$): 2453.3820, found 2453.3864. Elemental Analysis (%): ($C_{118}H_{58}F_{42}N_6O_6$) Calcd. C 57.76, H 2.38, N 3.43, found C 57.21, H 2.99, N 3.42.

C_5F_{11}-cage:

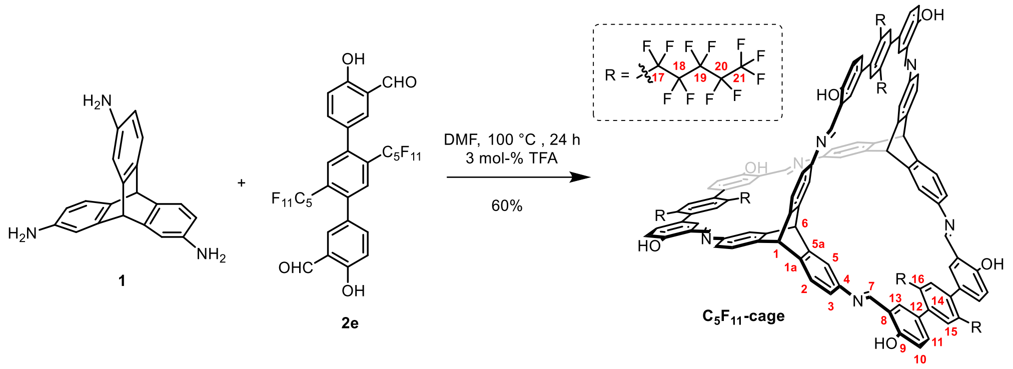

Triamino triptycene **1** (24.0 mg, 80 µmol) and salicylaldehyde **2e** (103 mg, 120 µmol) were suspended in anhydrous DMF (20 mL). After complete dissolution, 3 mol-% TFA (0.1 M in anhydrous DMF, 24 µL) was added. The mixture was heated to 100 °C and stirred for 24 hours. After cooling the reaction mixture to room temperature, the precipitate was filtered, washed with methanol (5 × 3 mL) and dried for 14 hours in high vacuum ($8.9 \cdot 10^{-1}$ mbar) at room temperature to give **C_5F_{11}-cage** as light yellow solid in 60% yield (73 mg, 23.9 µmol). M.p. >350 °C. ^{1}H NMR (600 MHz, THF-d_8)**:** δ = 13.36 ppm (s, 6H, 9-OH), 9.15 (s, 6H, imine-H-7), 7.83 (d, J = 2.2 Hz, 6H, H-5), 7.76 (s, 6H, H-15), 7.56 (d, J = 7.8 Hz, 6H, H-2), 7.43 – 7.38 (m, 12H, H-11/13), 7.25 (dd, J = 7.8, 2.1 Hz, 6H, H-3), 7.03 (d, J = 8.3 Hz, 6H, H-10), 5.72 (s, 2H, bridgehead-H-1), 5.57 (s, 2H, bridgehead-H-6). ^{13}C NMR (151 MHz, THF-d_8)**:** δ = 162.1 ppm (C-9), 159.6 (C-7), 146.6 (C-5a), 145.4 (C-1a), 144.1 (C-4), 142.7 (C-14), 134.4 (C-15), 134.0 (C-11), 133.7 (C-13), 130.4 (C-16), 129.7 (C-12), 126.0 (C-3), 125.6 (C-2), 119.3 (C-8), 117.2 (C-8), 111.3 (C-5), 56.1 (bridgehead-C-6), 53.3 (bridgehead-C-1). *Note***:** Due to strong and multiple ^{19}F-^{13}C-coupling, the signals of the perfluorinated side chains where not visible in the ^{13}C-NMR spectrum and were extracted from 2D NMR spectra. ^{19}F NMR (659 MHz, THF-d_8)**:** δ = -82.0 ppm (18F), -103.7 (12F), -120.0 (12F), -123.1 (12F), -126.9 (12F). IR (neat, ATR): $\tilde{\nu}$ =1628 (w), 1611 (w), 1584 (m), 1481 (m), 1474 (m), 1382 (w), 1359 (w), 1288 (m), 1231 (s), 1198 (s), 1173 (s), 1142 (s), 1134 (s), 1099 (m), 1085 (m), 1056 (w), 1048 (w), 972 (w), 958 (w), 946 (w), 892 (w), 860 (m), 832 (m), 811 (m), 795 (m), 780 (m), 744 (m), 732 (m), 702 (w), 680 (m), 661 (w), 649 (w) cm^{-1}. MS (MALDI-TOF): $[M+H]^+$: *m/z* calcd. for ($C_{130}H_{59}F_{66}N_6O_6^+$): 3053.3437, found 3053.3449. Elemental Analysis (%): ($C_{130}H_{58}F_{66}N_6O_6$) Calcd. C 51.13, H 1.91, N 2.75, found C 50.66 H 2.48, N 2.72.

C_6F_{13}-cage:

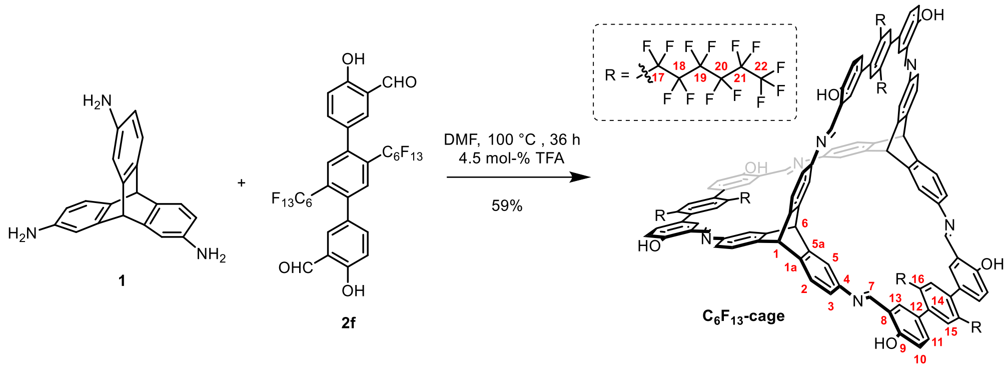

Triamino triptycene **1** (12.0 mg, 40 µmol) and salicylaldehyde **2e** (57.3 mg, 60 µmol) were suspended anhydrous DMF (20 mL). After complete dissolution, 4.5 mol-% TFA (0.1 M in anhydrous DMF, 18 µL) was added. The mixture was heated to 100 °C and stirred for 36 hours. After cooling the reaction mixture to room temperature and additional stirring at this temperature for 12 hours, the precipitate was filtered, washed with methanol (3 × 5 mL) and dried for 8 hours under high vacuum at room temperature to give **C_6F_{13}-cage** as light yellow solid in 59% yield (39.6 mg, 11.8 µmol). Mp >350 °C. ^{1}H NMR (700 MHz, THF-*d*8)**:** δ = 13.36 ppm (s, 6H, 9-OH), 9.15 (s, 6H, imine-H-7), 7.82 (s, 6H, H-5), 7.76 (s, 6H, H-15), 7.56 (d, *J* = 7.7 Hz, 6H, H-2), 7.44 – 7.37 (m, 12H, H-11/13), 7.25 (dd, *J* = 7.6, 1.9 Hz, 6H, H-3), 7.03 (d, *J* = 8.0 Hz, 6H, H-10), 5.72 (s, 2H, bridgehead-H-1), 5.57 (s, 2H, bridgehead-H-6). ^{13}C NMR (176 MHz, THF-*d*8)**:** δ = 162.1 ppm (C-9), 159.6 (C-7), 146.6 (C-5a), 145.3 (C-1a), 144.1 (C-4), 142.7 (C-14), 134.4 (C-15), 134.0 (C-11), 133.7 (C-13), 130.4 (C-16), 129.7 (C-12), 126.0 (C-3), 125.6 (C-2), 119.3 (C-8), 117.2 (C-8), 111.2 (C-5), 56.2 (bridgehead-C-6), 53.3 (bridgehead-C-1). **Note:** Due to strong and multiple ^{19}F-^{13}C-coupling, the signals of the perfluorinated side chains where not visible in the ^{13}C NMR spectrum and have been extracted from 2D NMR spectra. ^{19}F NMR (471 MHz, THF-*d*8)**:** δ = -83.9 (18F), -105.5 (12F), -121.8 (12F), -124.2 (12F), -124.9 (12F), -128.8 (12F). IR (neat, ATR): $\tilde{\nu}$ = 1628 (w), 1610 (w), 1583 (w), 1481 (m), 1383 (w), 1362 (w), 1281 (m), 1232 (s), 1198 (vs), 1171 (s), 1144 (s), 1119 (s), 1092 (m), 1061 (w), 1034 (w), 1018 (w), 959 (w), 945 (w), 924 (w), 889 (w), 860 (m), 829 (m), 795 (m), 775 (m), 744 (m), 735 (m), 721 (m), 700 (m), 677 (m), 658 (m), 631 (w) cm^{-1}. MS (MALDI-TOF): [M+H]$^+$: *m/z* calcd. for ($C_{136}H_{59}F_{78}N_6O_6^+$): 3353.3245, found 3353.3261. Elemental Analysis (%): ($C_{136}H_{58}F_{78}N_6O_6$) Calcd. C 48.70, H 1.74, N 2.51; found C 49.28, H 2.08, N 2.15.

3. NMR Spectra

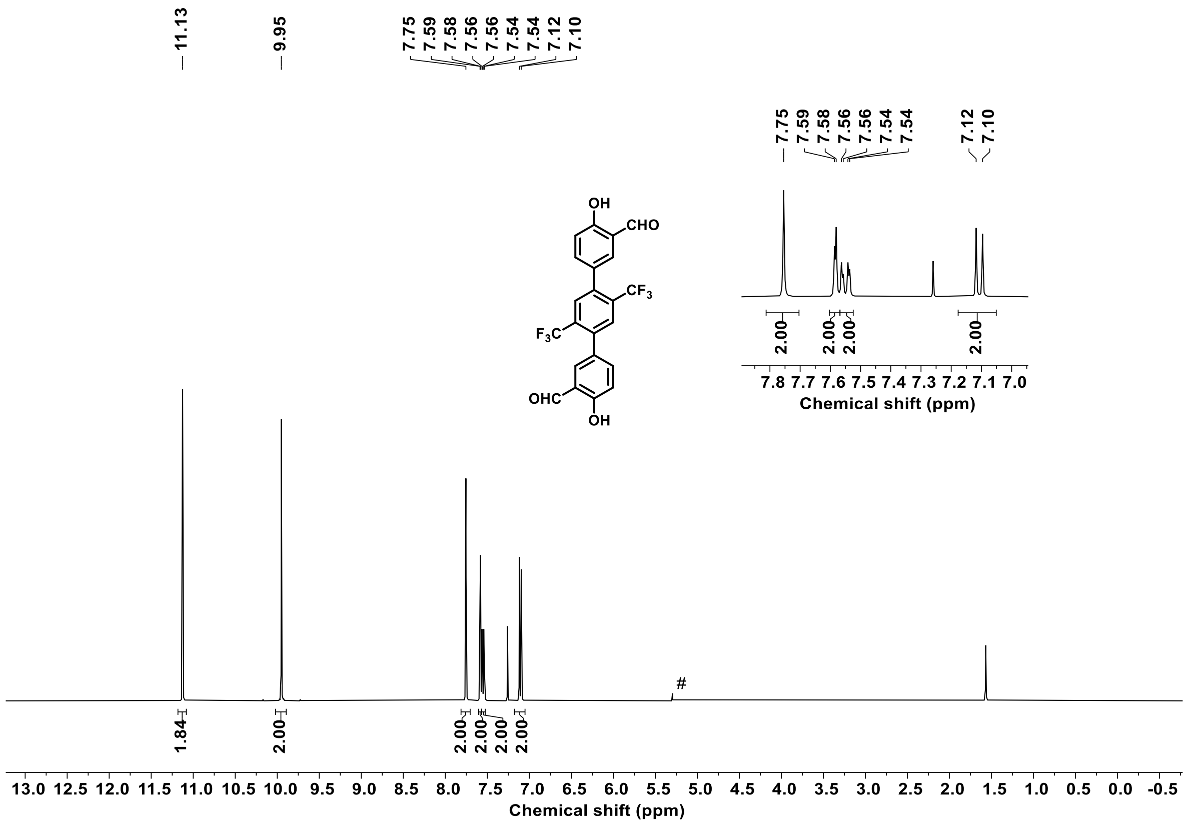

Figure S1. ^{1}H NMR spectrum of **2a** ($CDCl_3$, 400 MHz). The hash-symbol marks residual DCM.

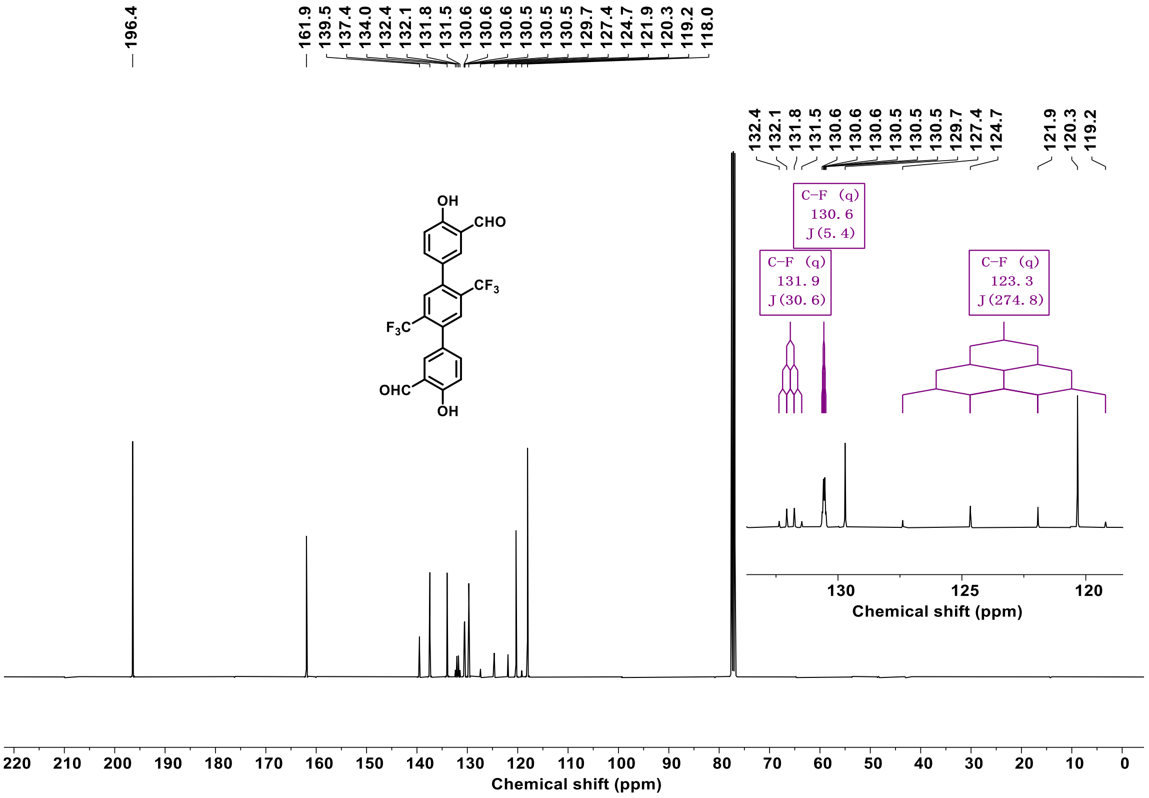

Figure S2. ^{13}C {^{1}H} NMR spectrum of **2a** ($CDCl_3$, 101 MHz).

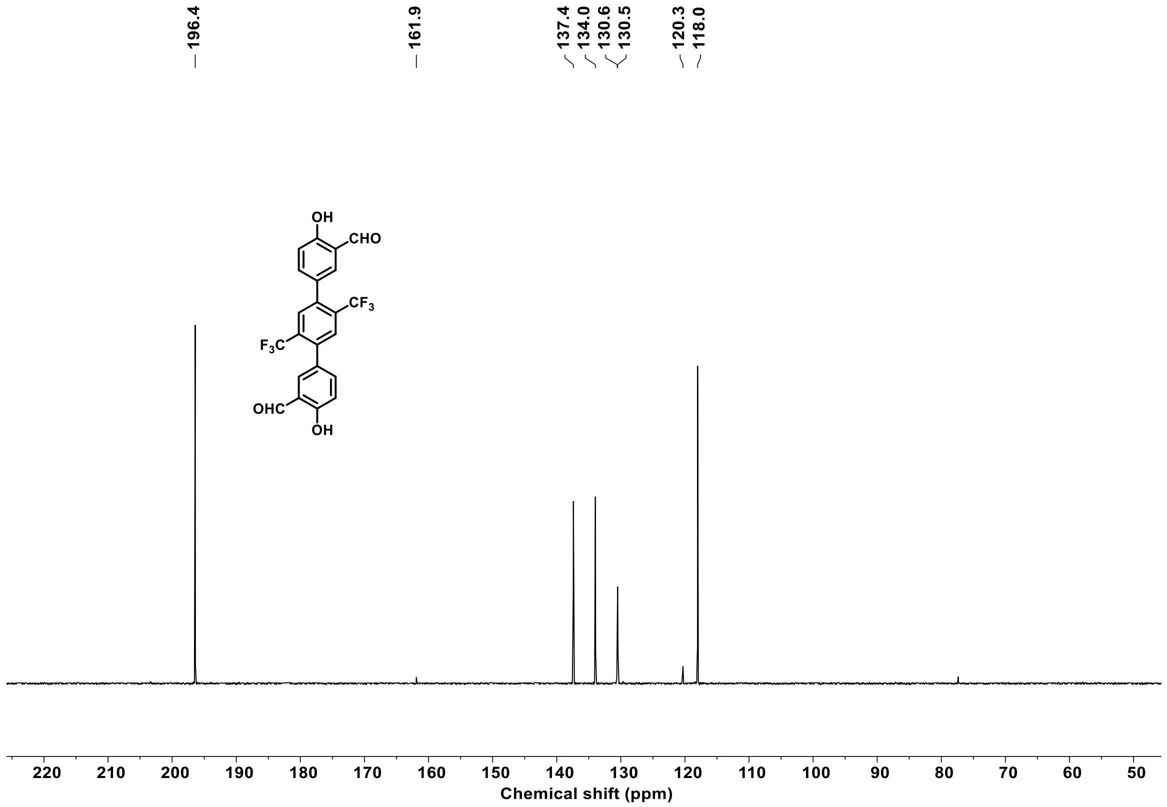

Figure S3. ^{13}C-DEPT135 NMR spectrum of **2a** ($CDCl_3$, 101 MHz).

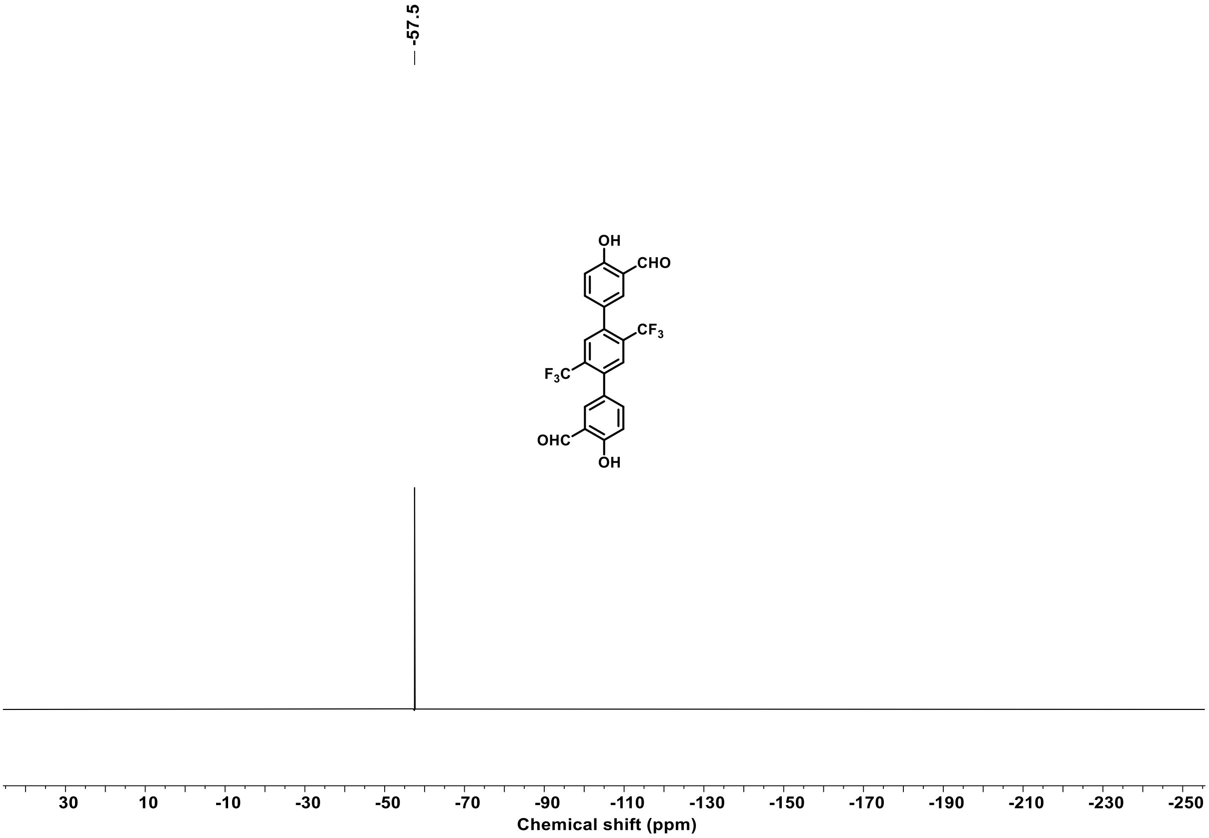

Figure S4. ^{19}F {^{1}H} NMR spectrum of **2a** (THF-d_8, 471 MHz).

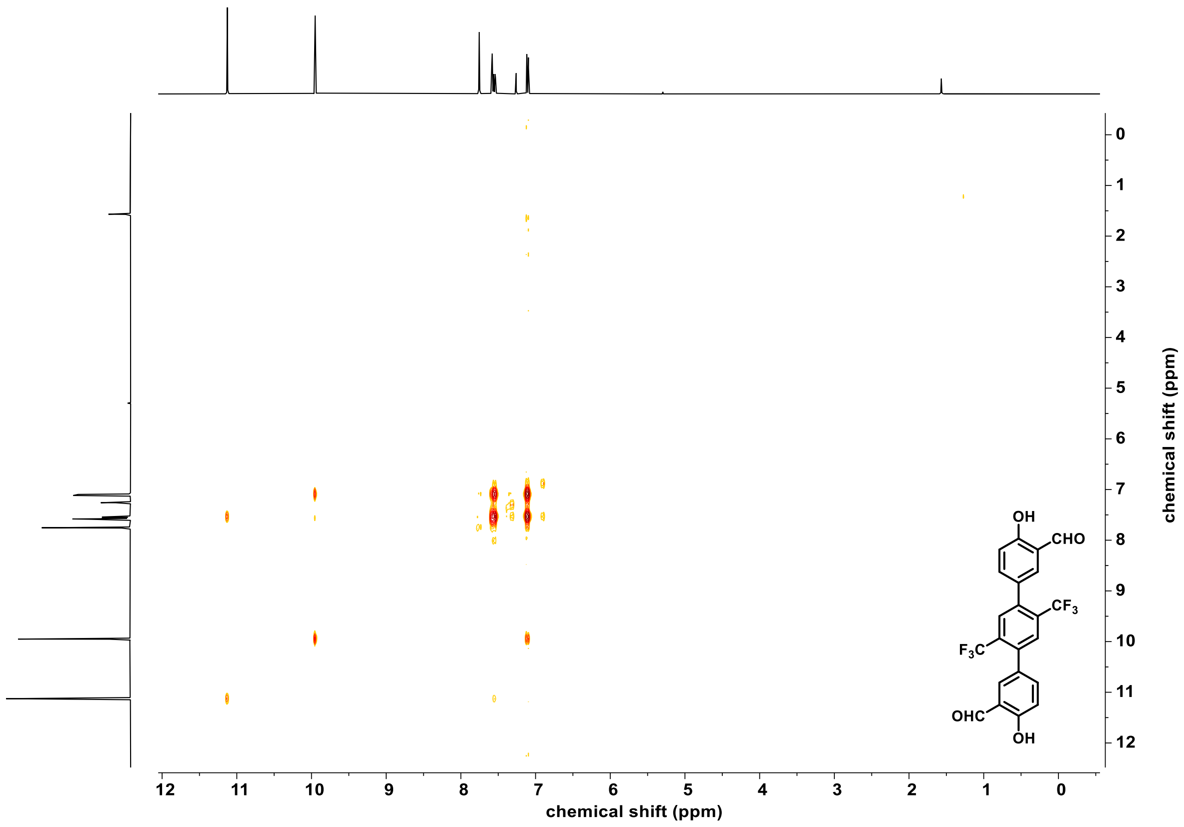

Figure S5. ^{1}H-^{1}H COSY NMR spectrum of **2a** ($CDCl_3$, 400 MHz, 400 MHz).

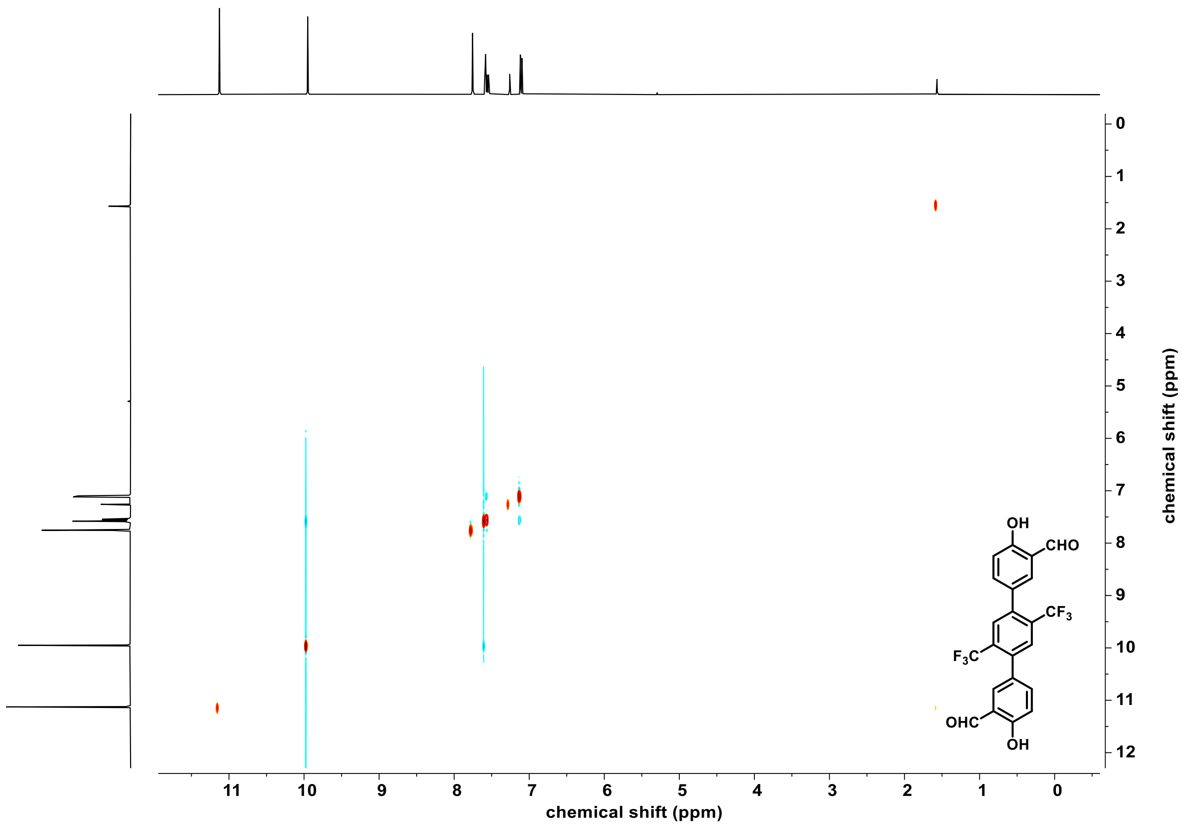

Figure S6. ^{1}H-^{1}H NOSY NMR spectrum of **2a** ($CDCl_3$, 400 MHz, 400 MHz).

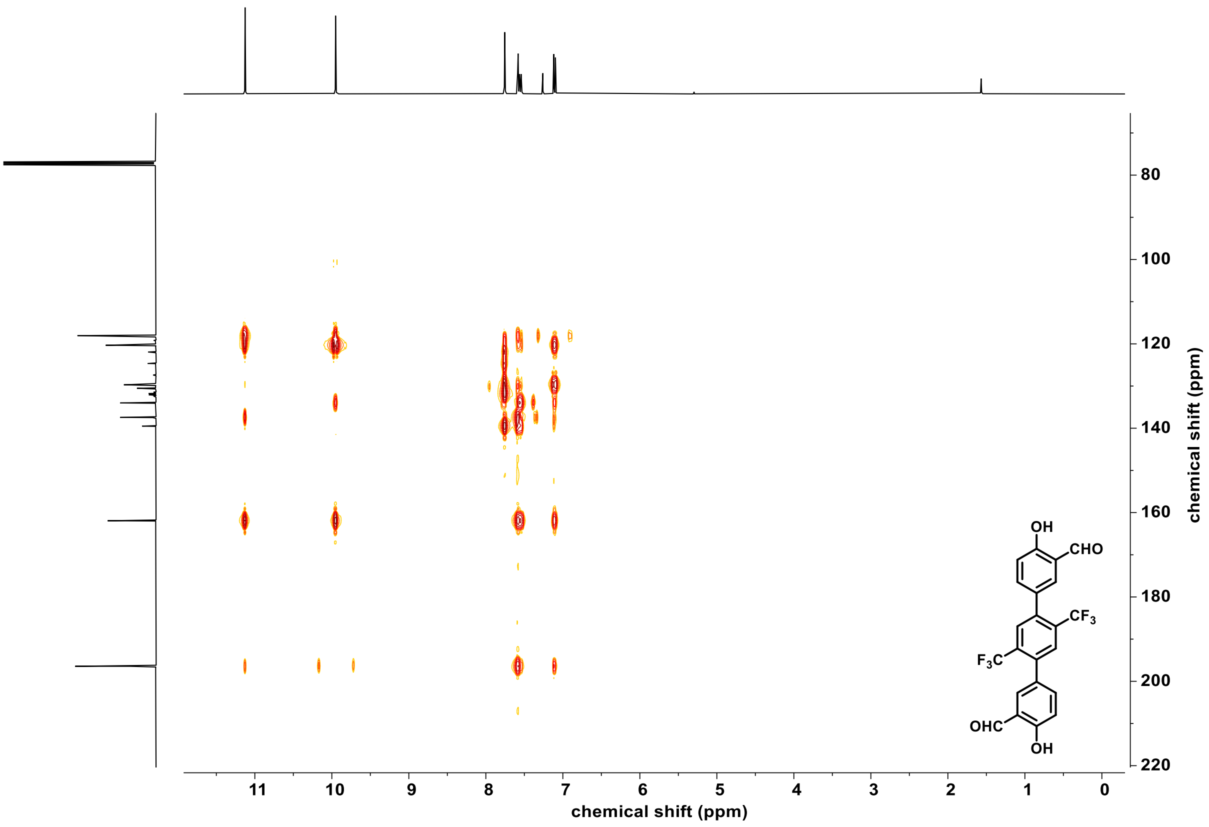

Figure S7. ^{1}H-^{13}C HMBC NMR spectrum of **2a** ($CDCl_3$, 400 MHz, 101 MHz).

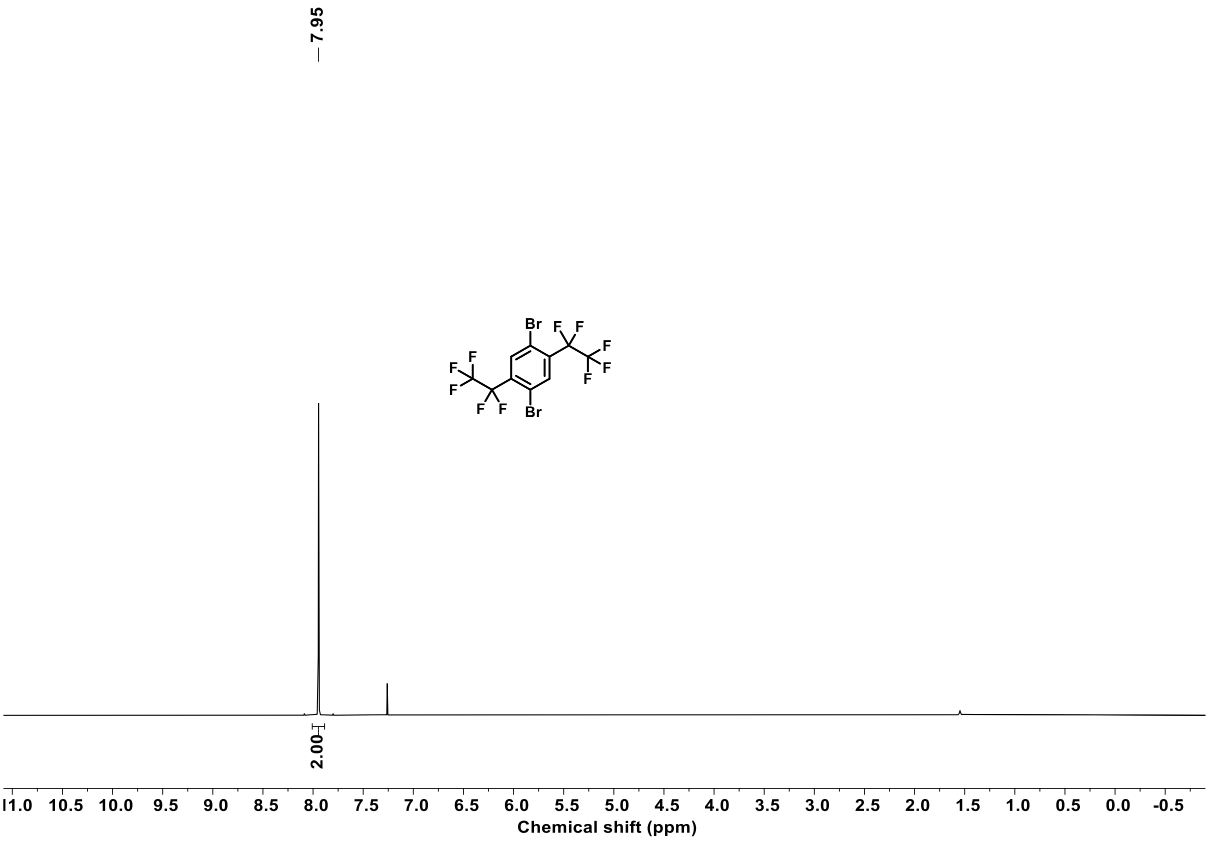

Figure S8. ^{1}H NMR spectrum of **S6** ($CDCl_3$, 600 MHz).

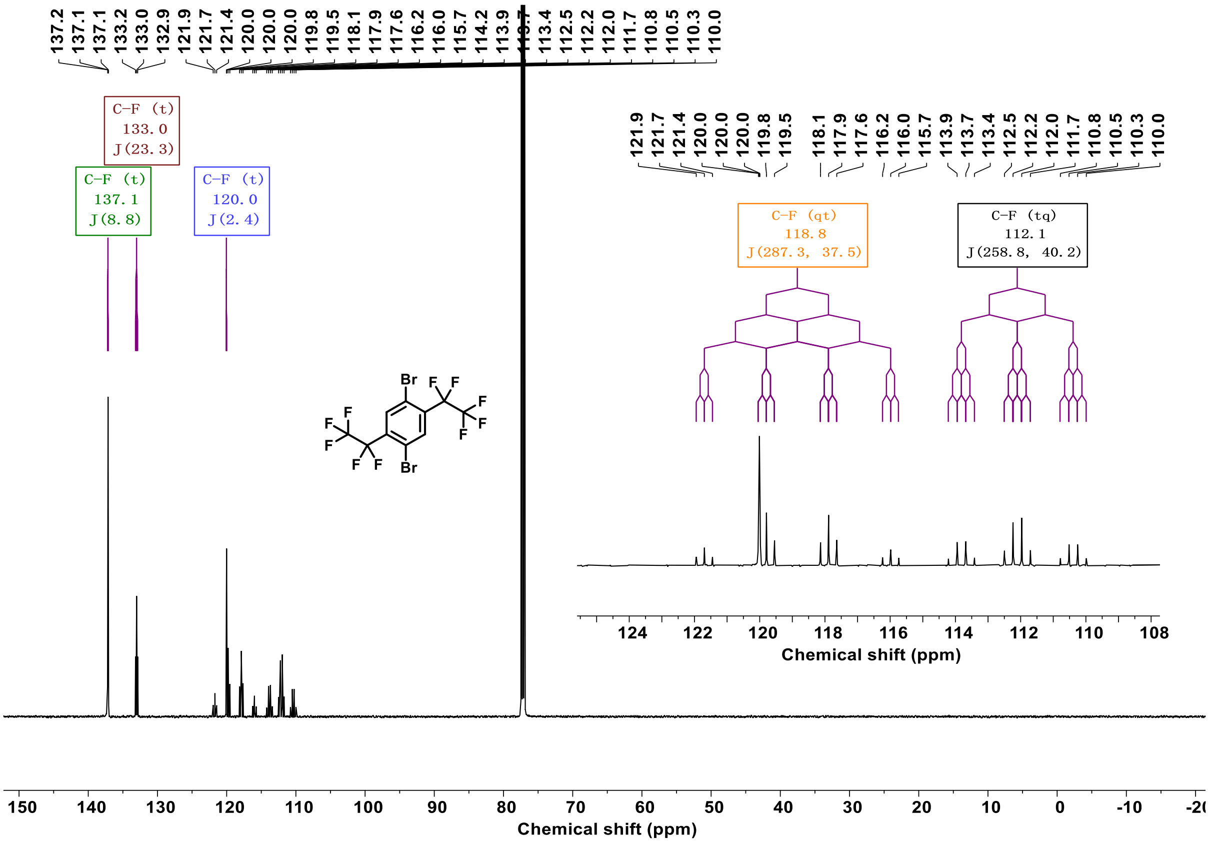

Figure S9. $^{13}C\ \{^1H\}$ NMR spectrum of **S6** ($CDCl_3$, 151 MHz).

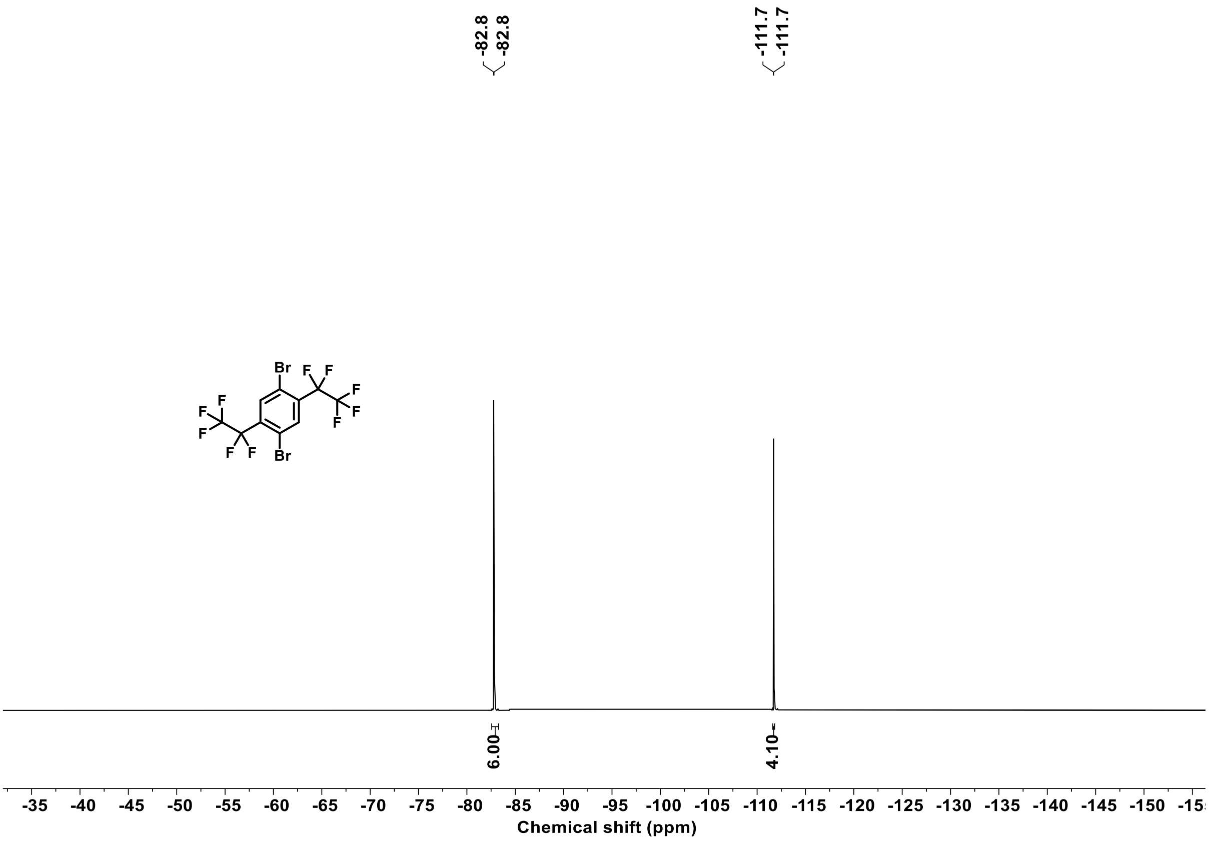

Figure S10. $^{19}F\ \{^1H\}$ NMR spectrum of **S6** ($CDCl_3$, 471 MHz).

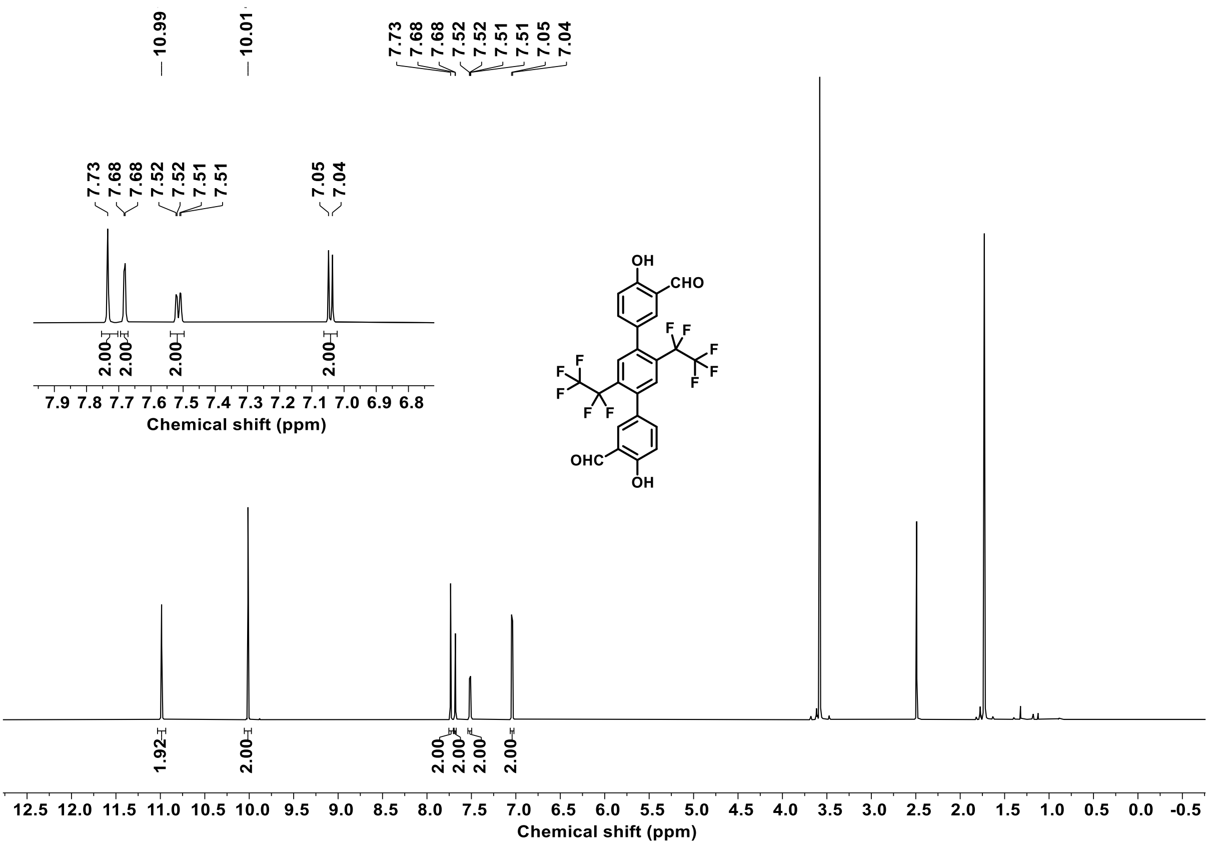

Figure S11. ^{1}H NMR spectrum of **2b** (THF-d$_8$, 700 MHz).

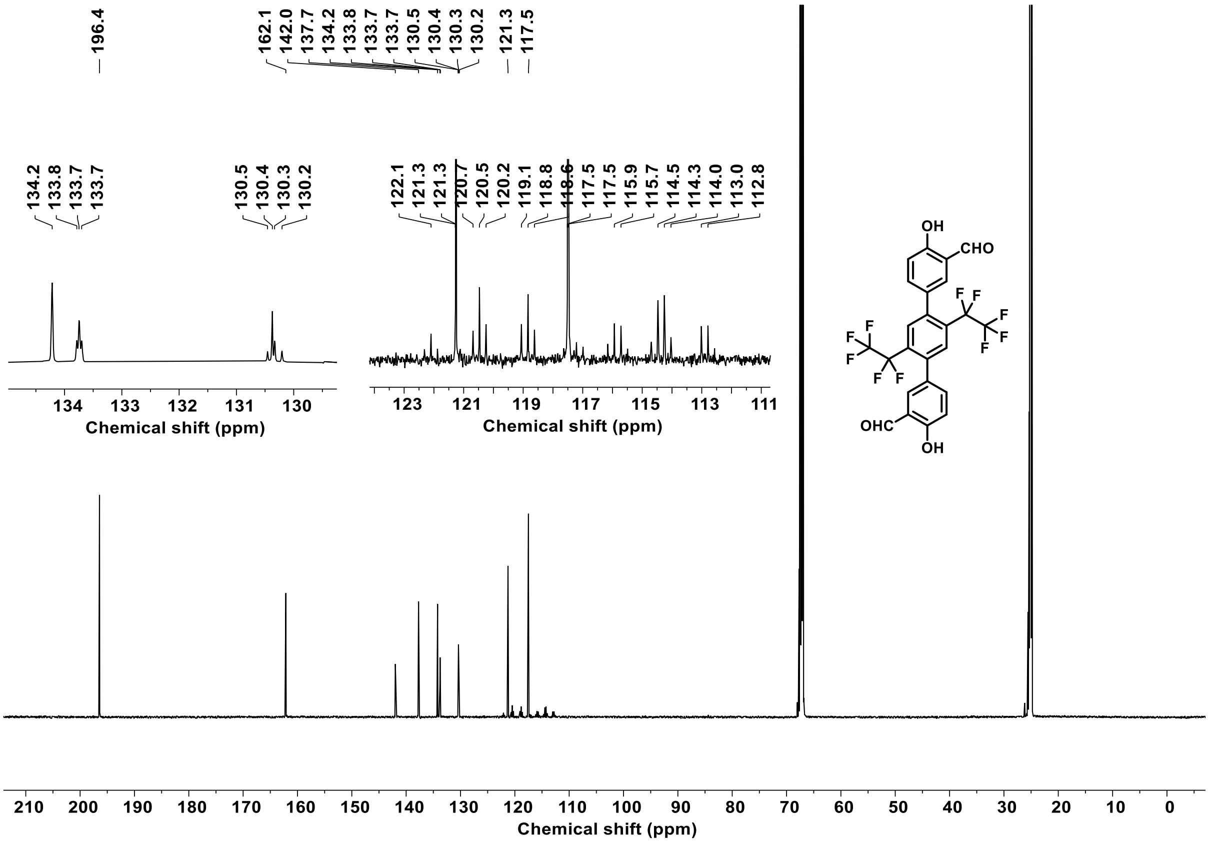

Figure S12. ^{13}C {^{1}H} NMR spectrum of **2b** (THF-d$_8$, 176 MHz).

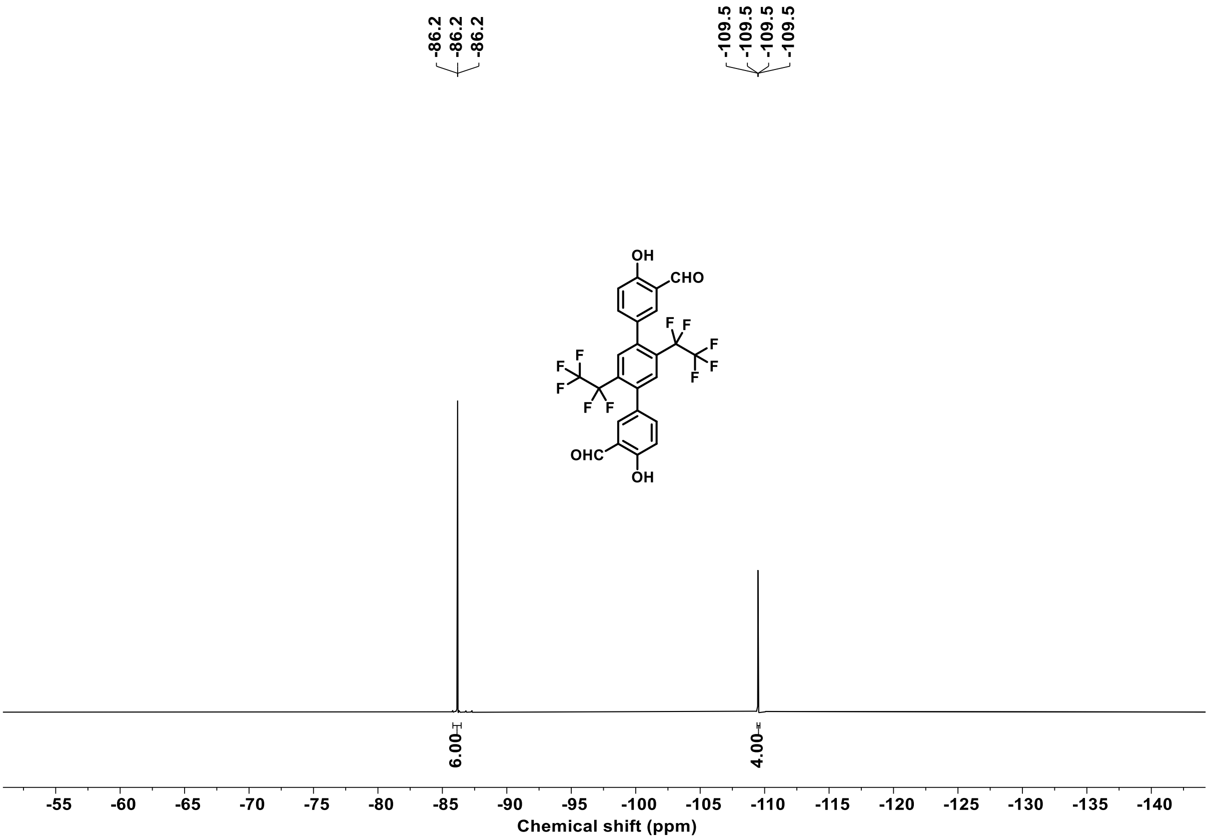

Figure S13. ^{19}F {^{1}H} NMR spectrum of **2b** (THF-d_8, 283 MHz).

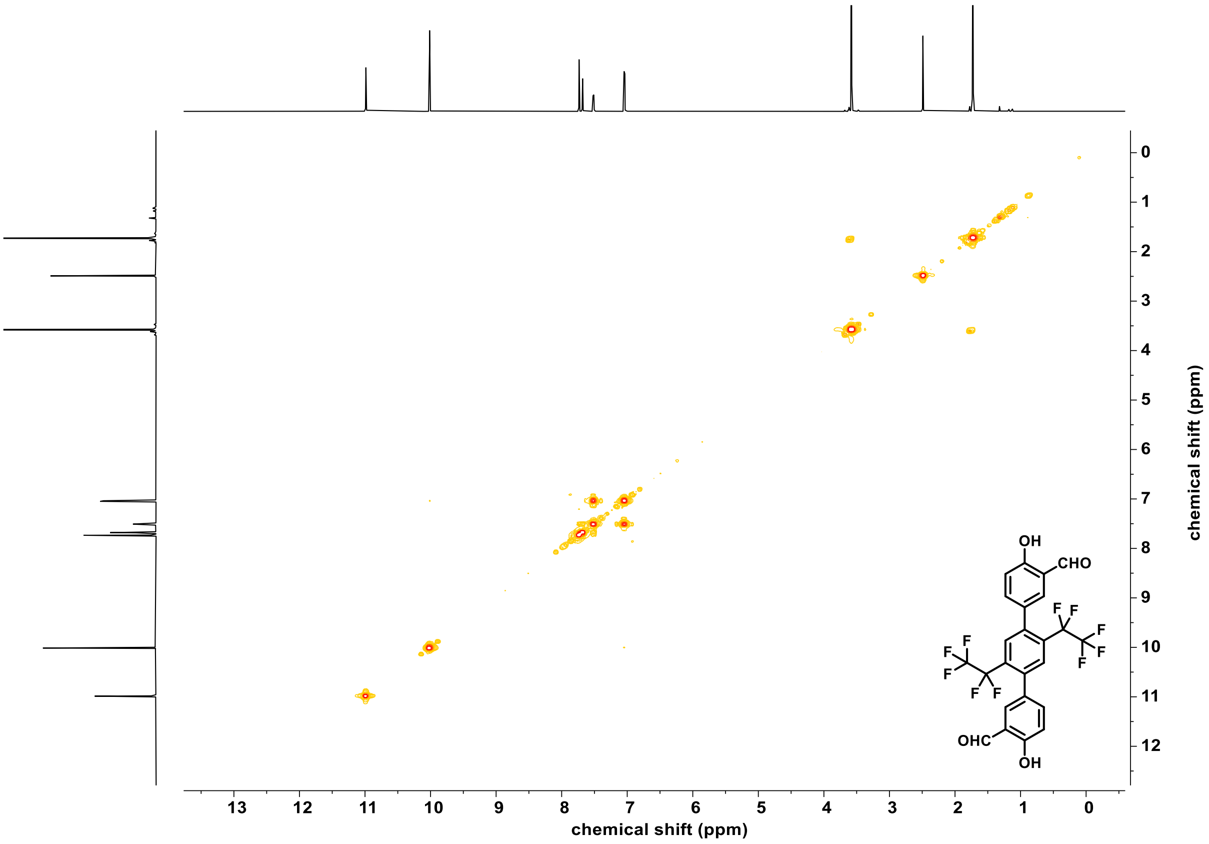

Figure S14. ^{1}H-^{1}H COSY NMR spectrum of **2b** (THF-d_8, 700 MHz, 700 MHz).

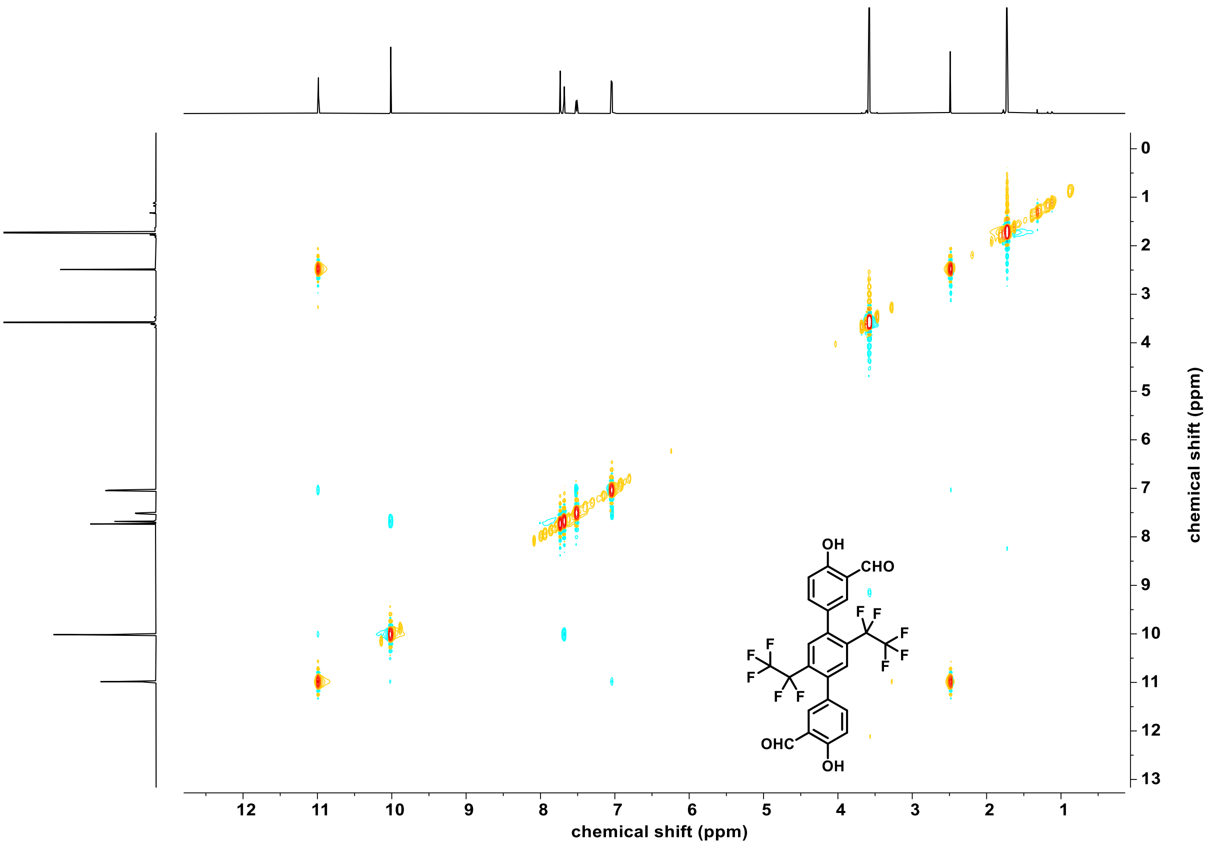

Figure S15. ^{1}H-^{1}H NOSY NMR spectrum of **2b** (THF-d_8, 700 MHz, 700 MHz).

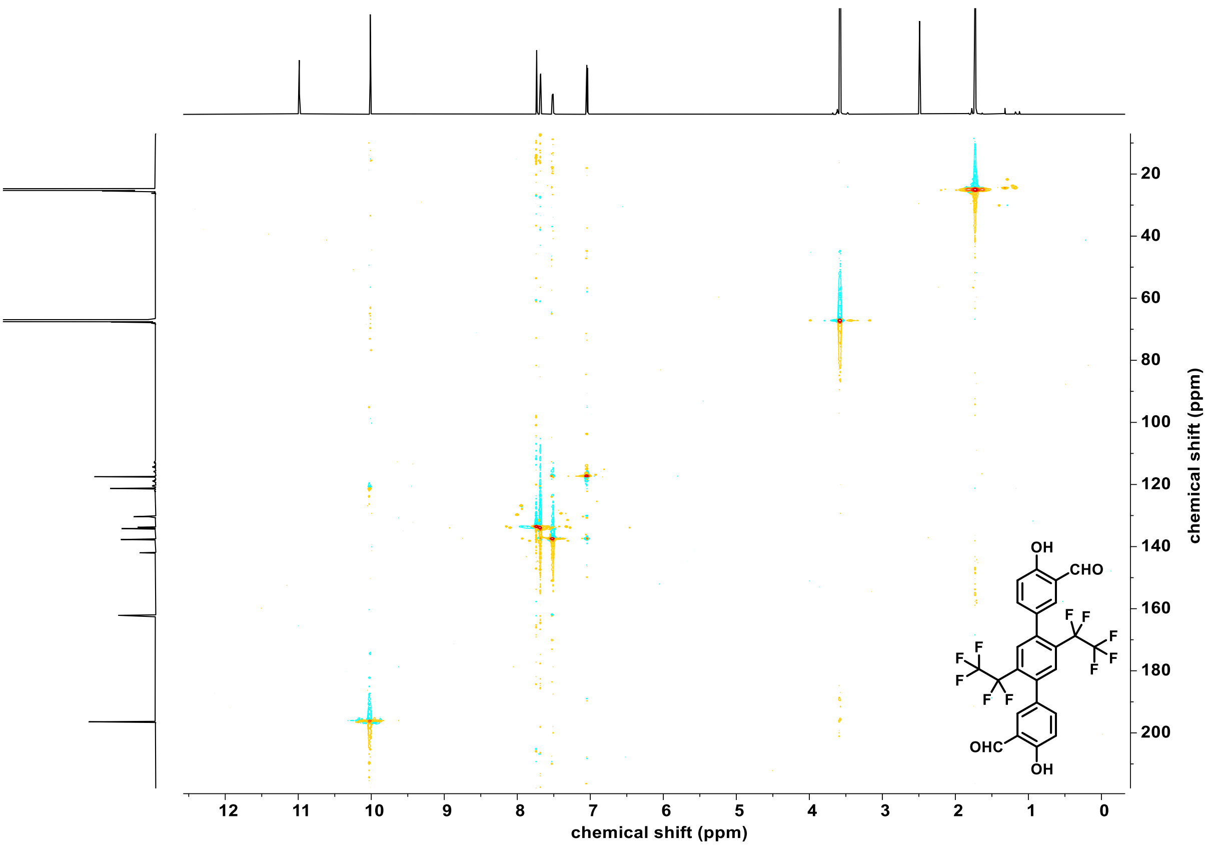

Figure S16. ^{1}H-^{13}C HSQC NMR spectrum of **2b** (THF-d_8, 700 MHz, 176 MHz).

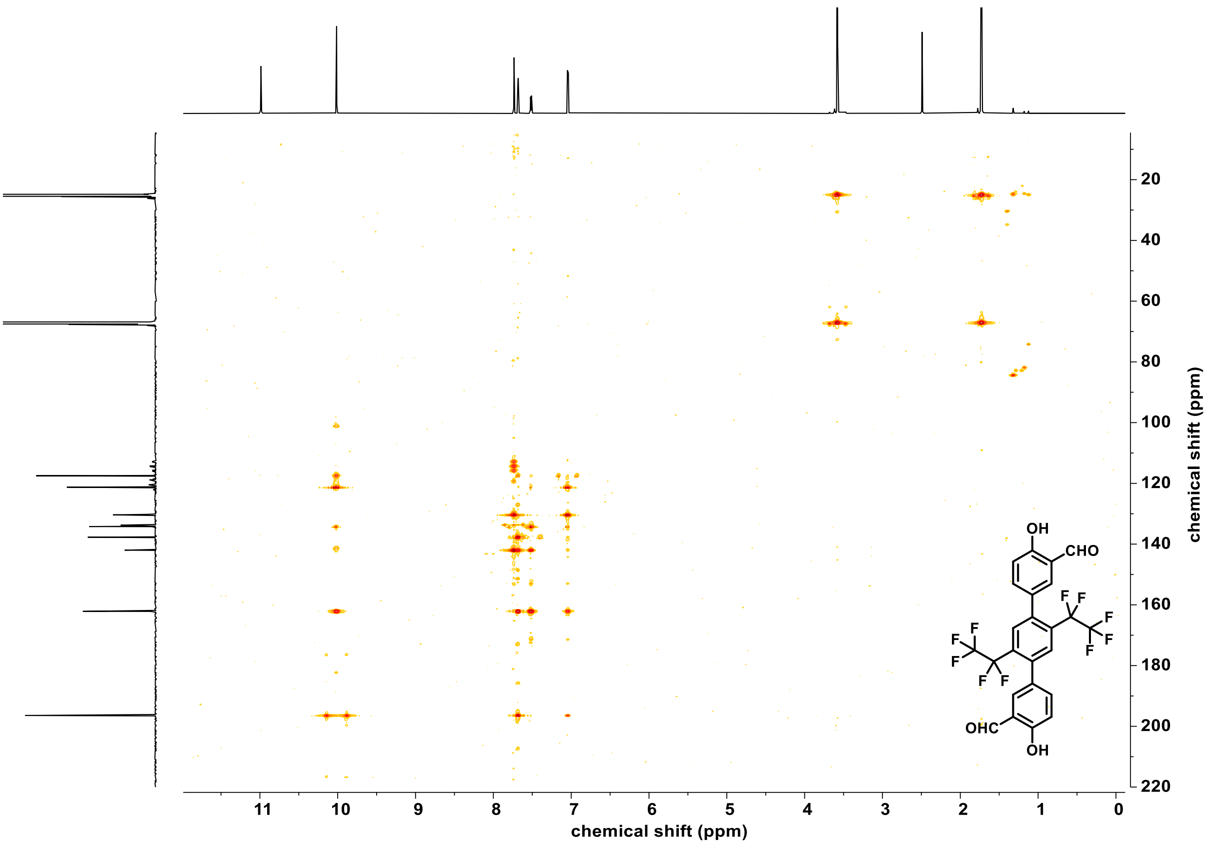

Figure S17. ^{1}H-^{13}C HMBC NMR spectrum of **2b** (THF-d_8, 700 MHz, 176 MHz).

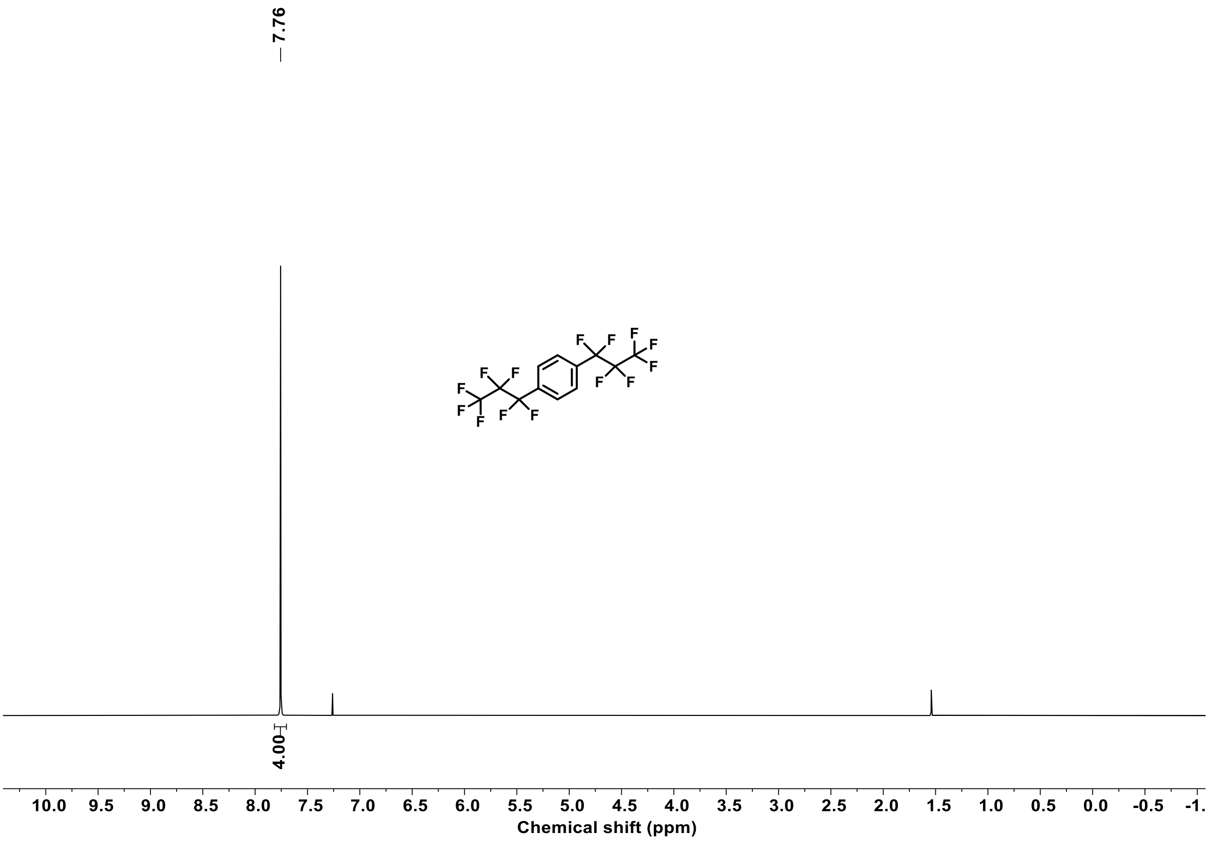

Figure S18. ^{1}H NMR spectrum of **S8** ($CDCl_3$, 600 MHz).

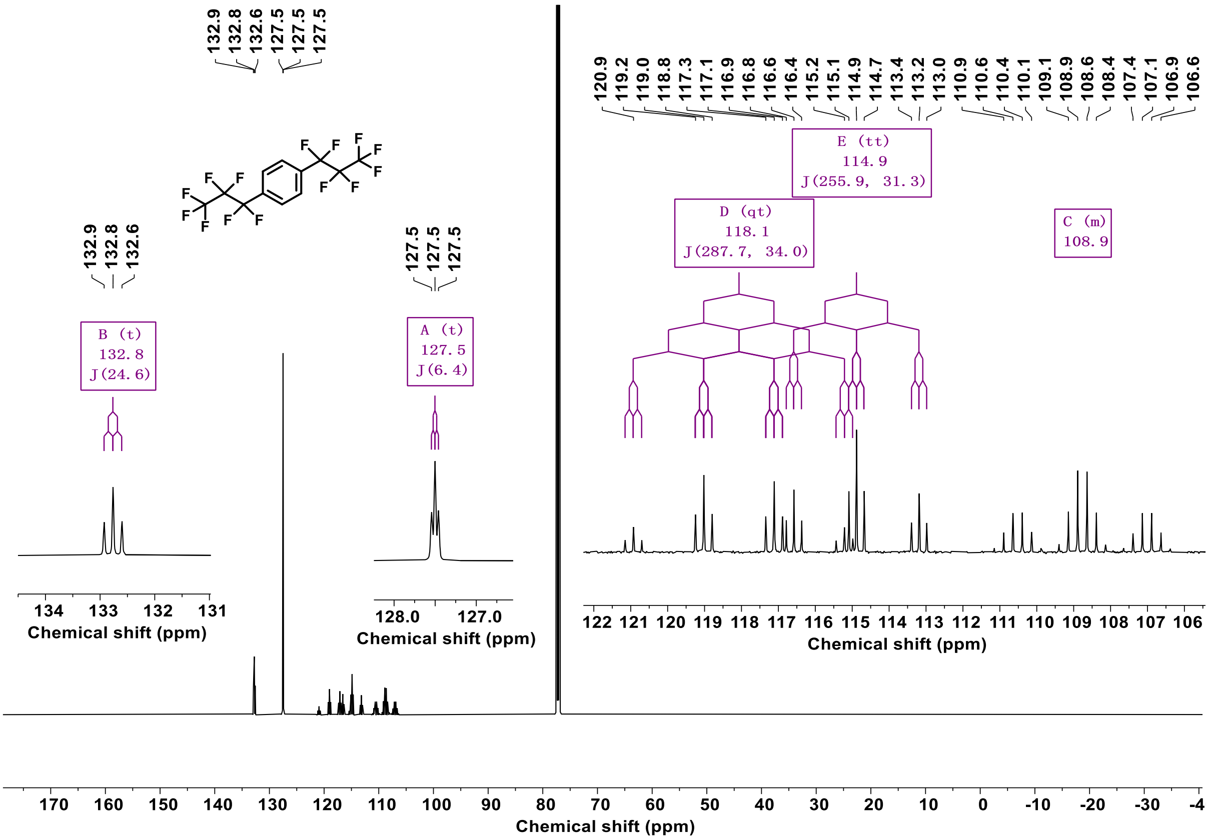

Figure S19. $^{13}C\{^{1}H\}$ NMR spectrum of **S8** ($CDCl_3$, 151 MHz).

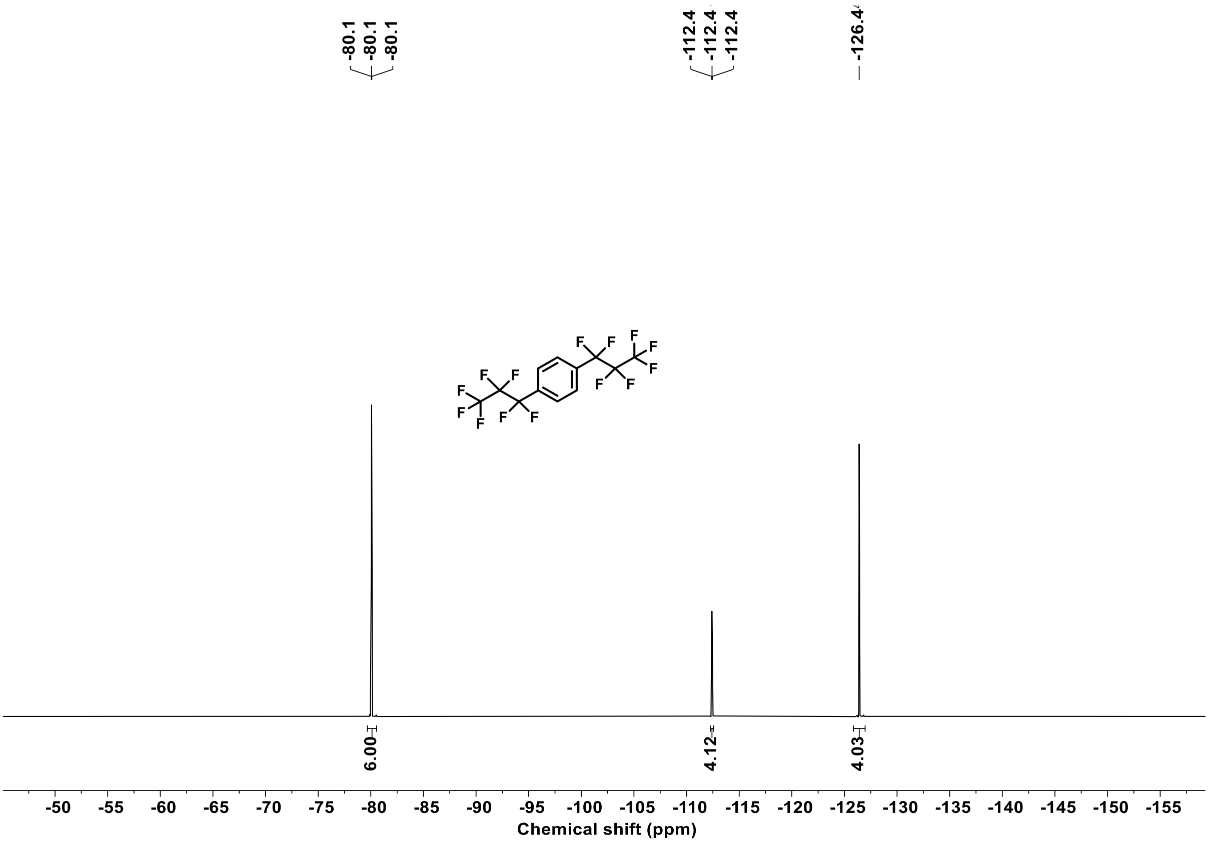

Figure S20. $^{19}F\{^{1}H\}$ NMR spectrum of **S8** ($CDCl_3$, 471 MHz).

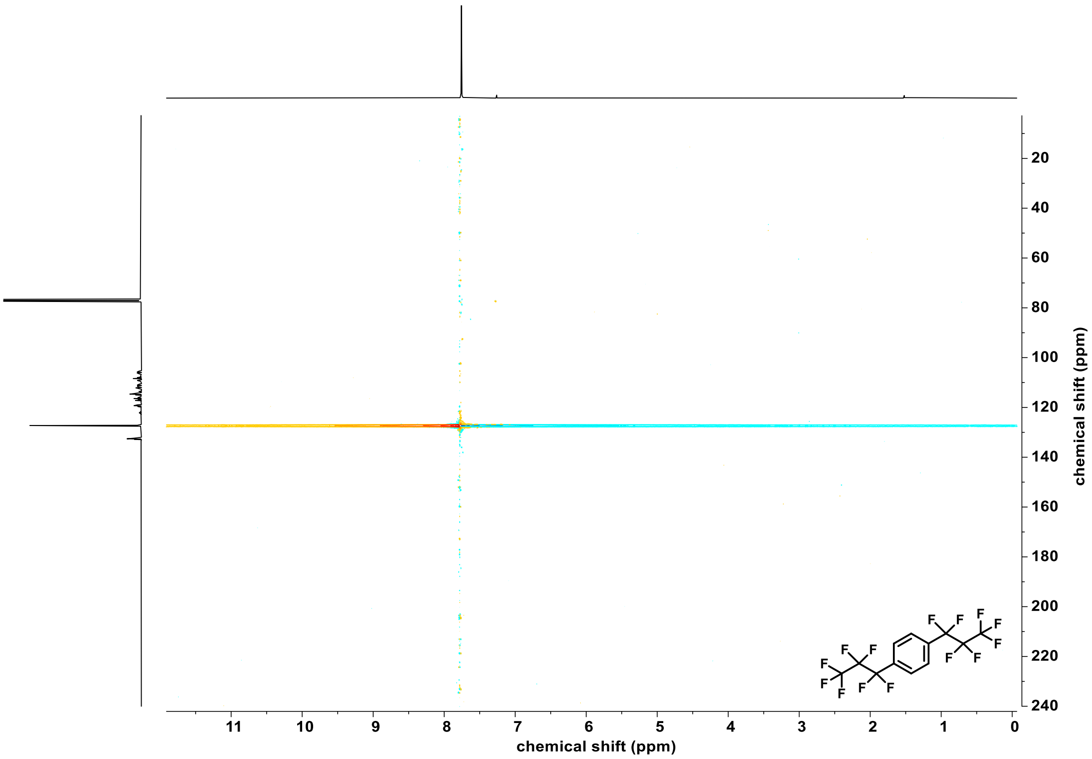

Figure S21. ^{1}H-^{13}C HSQC NMR spectrum of **S8** ($CDCl_3$, 400 MHz, 101 MHz).

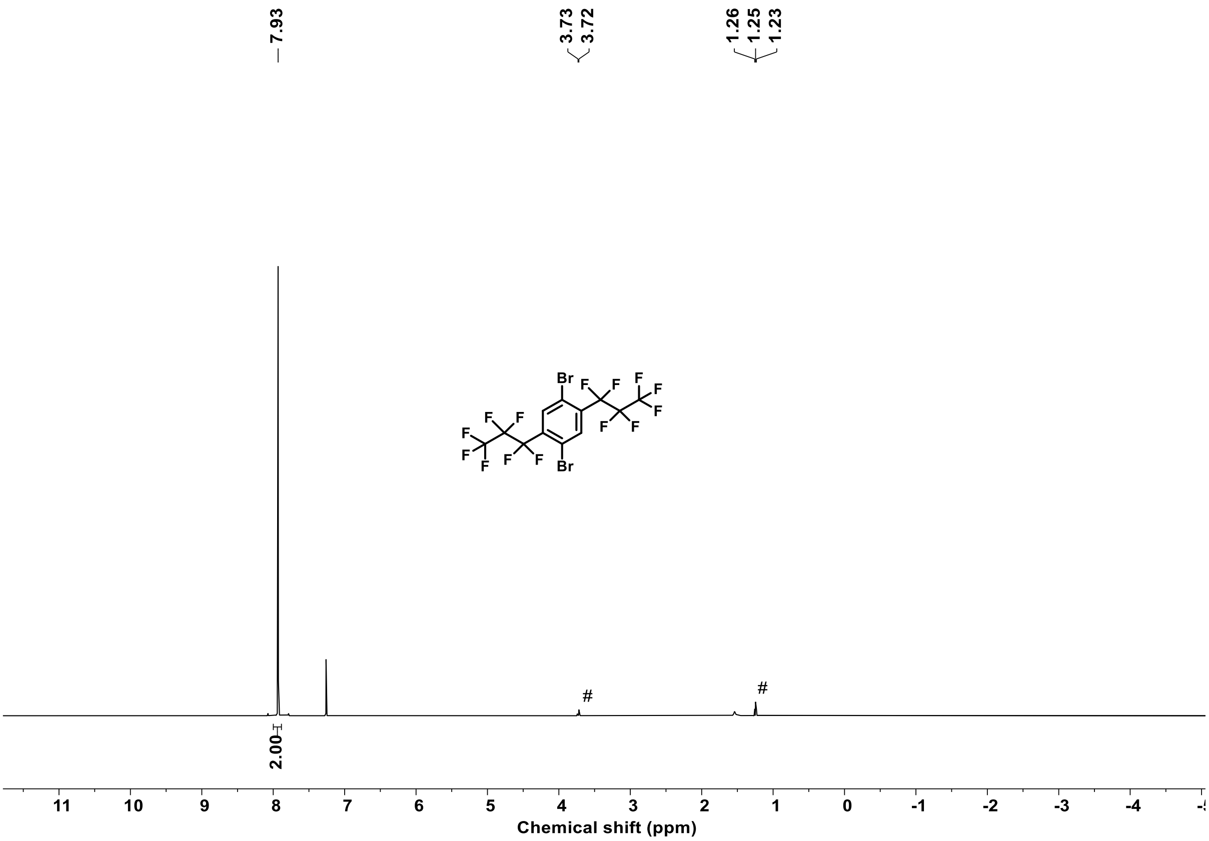

Figure S22. ^{1}H NMR spectrum of **S9** ($CDCl_3$, 600 MHz). The hash symbol marks residual methanol.

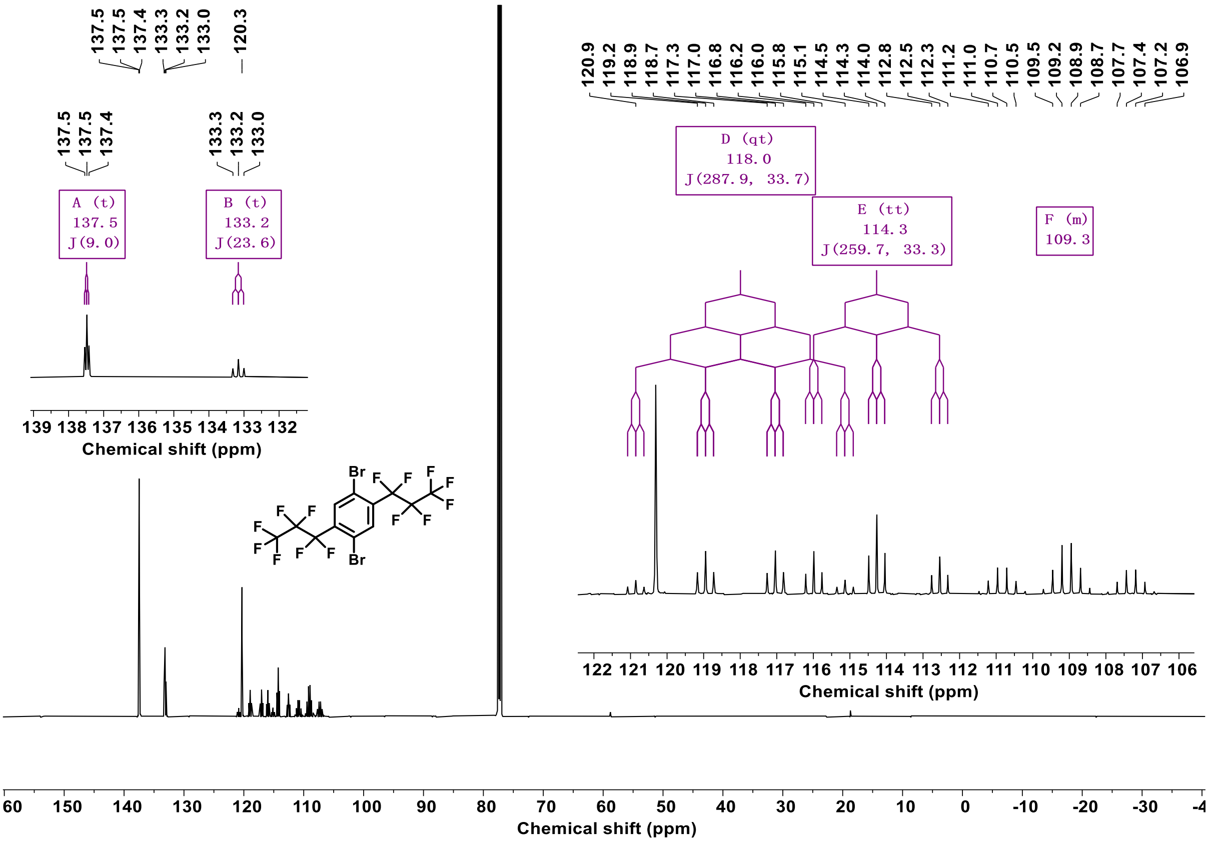

Figure S23. ^{13}C {^{1}H} NMR spectrum of **S9** ($CDCl_3$, 151 MHz).

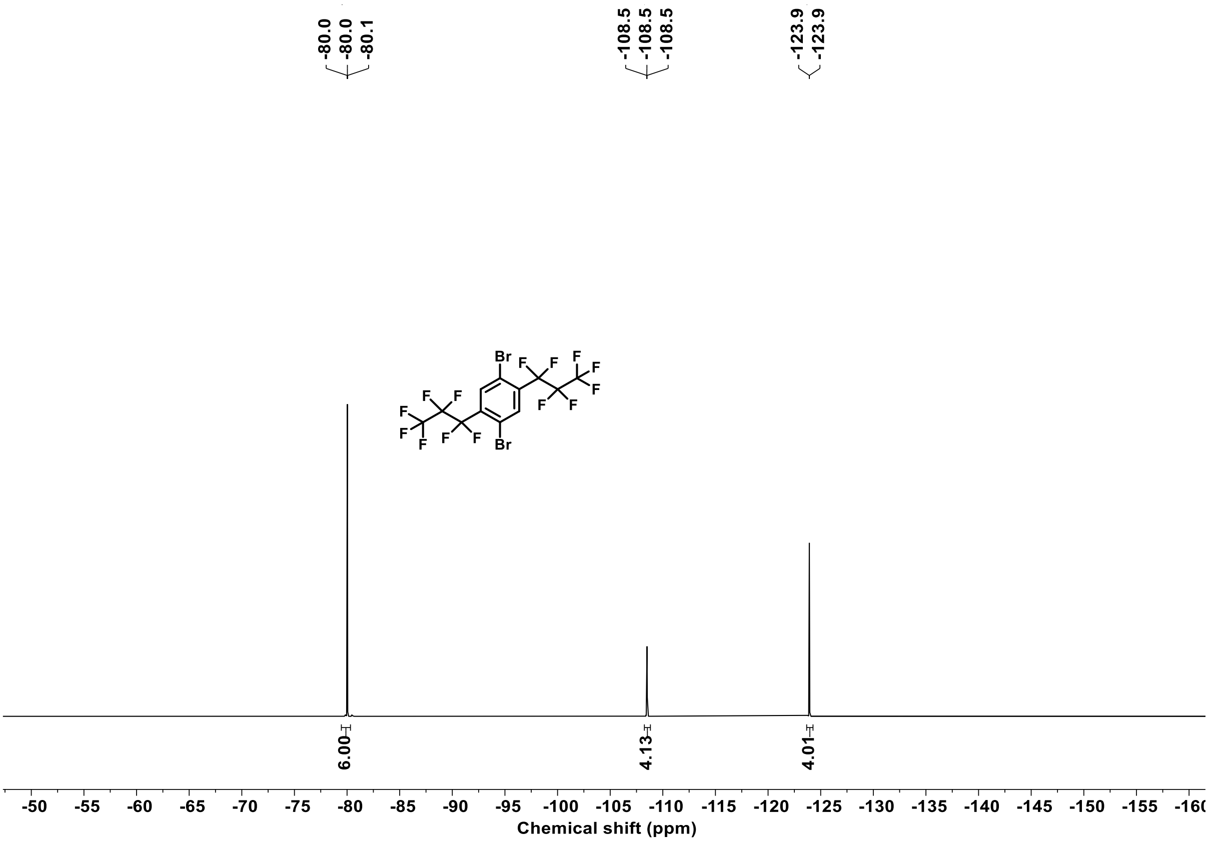

Figure S24. ^{19}F {^{1}H} NMR spectrum of **S9** ($CDCl_3$, 471 MHz).

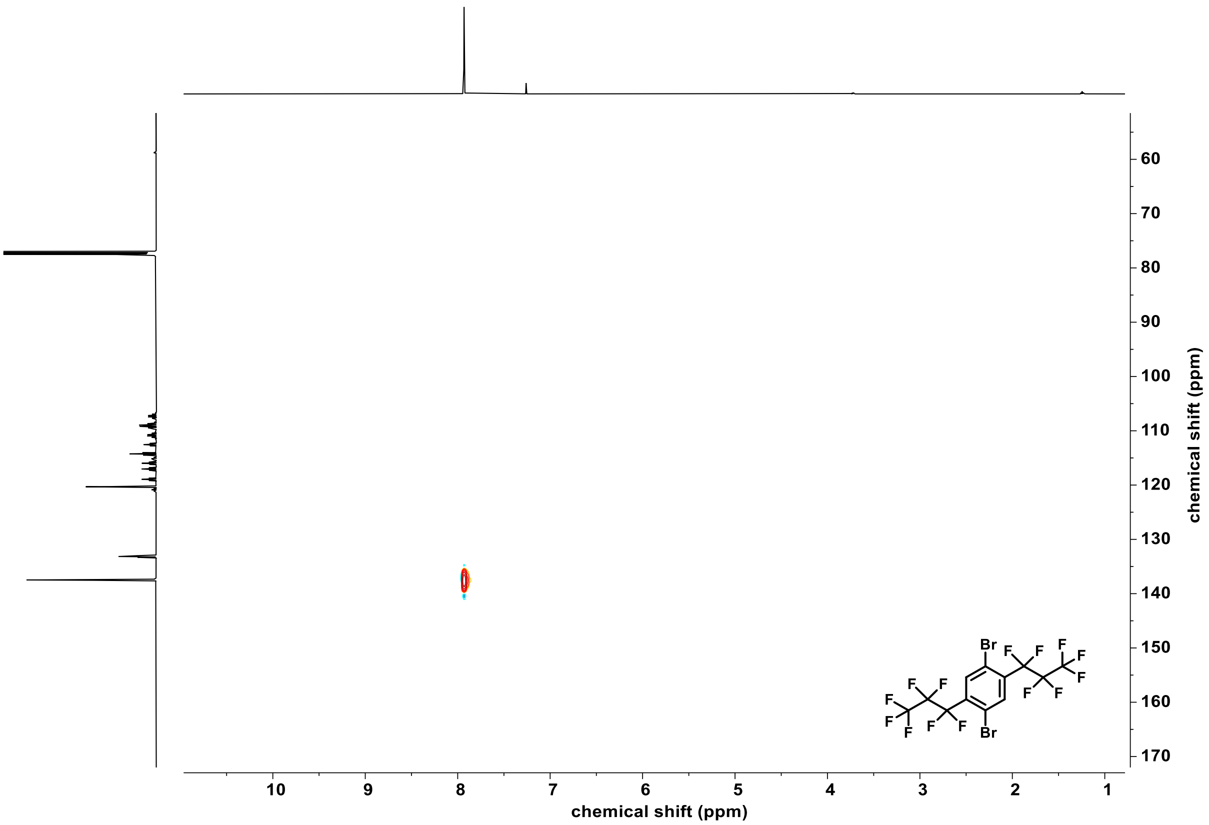

Figure S25. ^{1}H-^{13}C HSQC NMR spectrum of **S9** ($CDCl_3$, 500 MHz, 126 MHz).

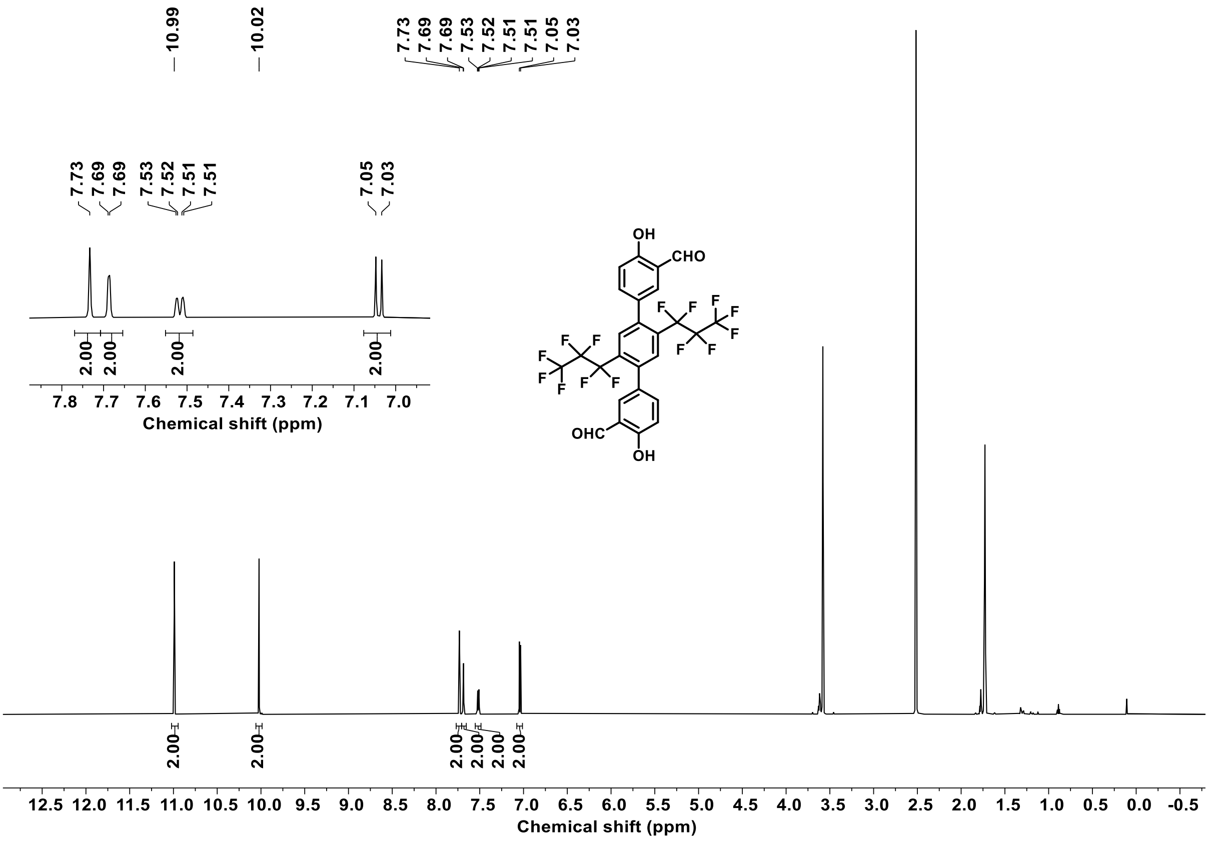

Figure S26. ^{1}H NMR spectrum of **2c** (THF-d_8, 600 MHz).

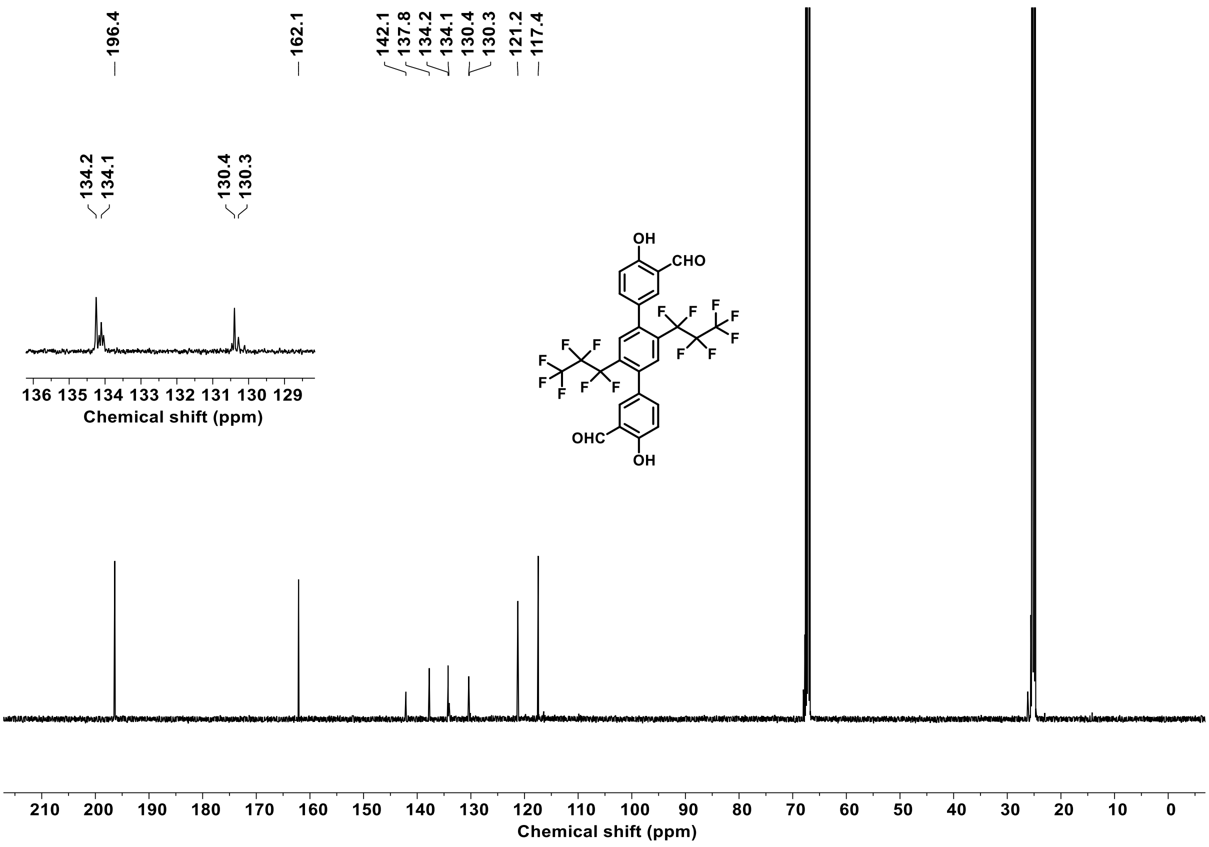

Figure S27. ^{13}C {^{1}H} NMR spectrum of **2c** (THF-d$_8$, 126 MHz).

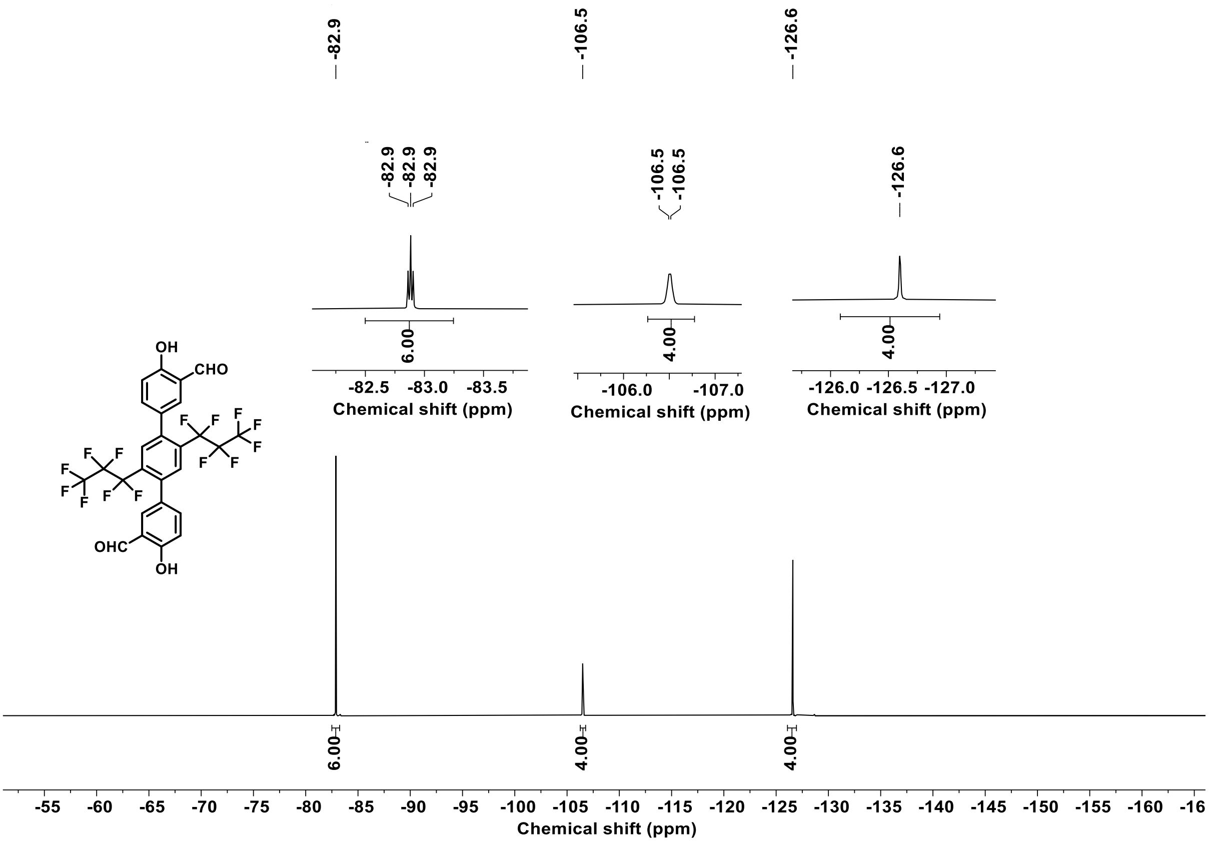

Figure S28. ^{19}F {^{1}H} NMR spectrum of **2c** (THF-d$_8$, 471 MHz).

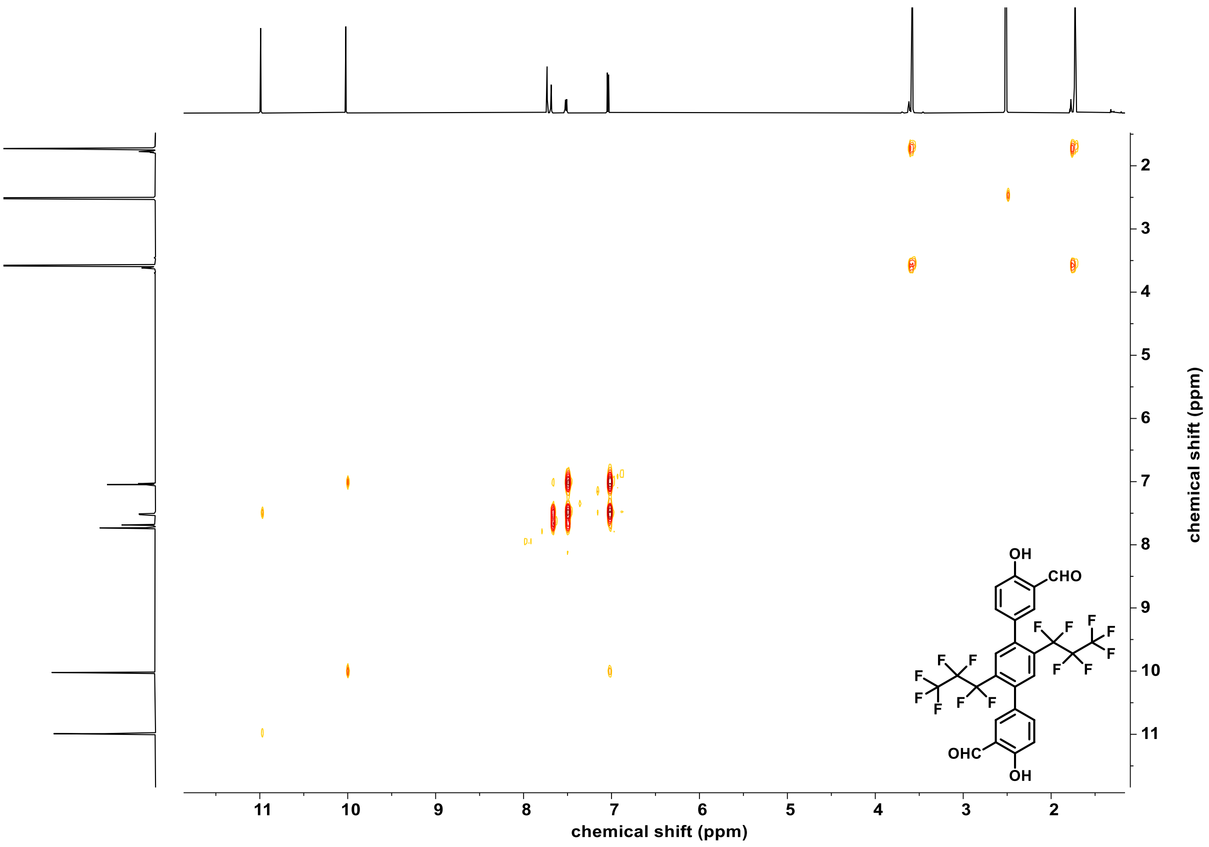

Figure S29. ^{1}H-^{1}H COSY NMR spectrum of **2c** (THF-d8, 600 MHz, 600 MHz).

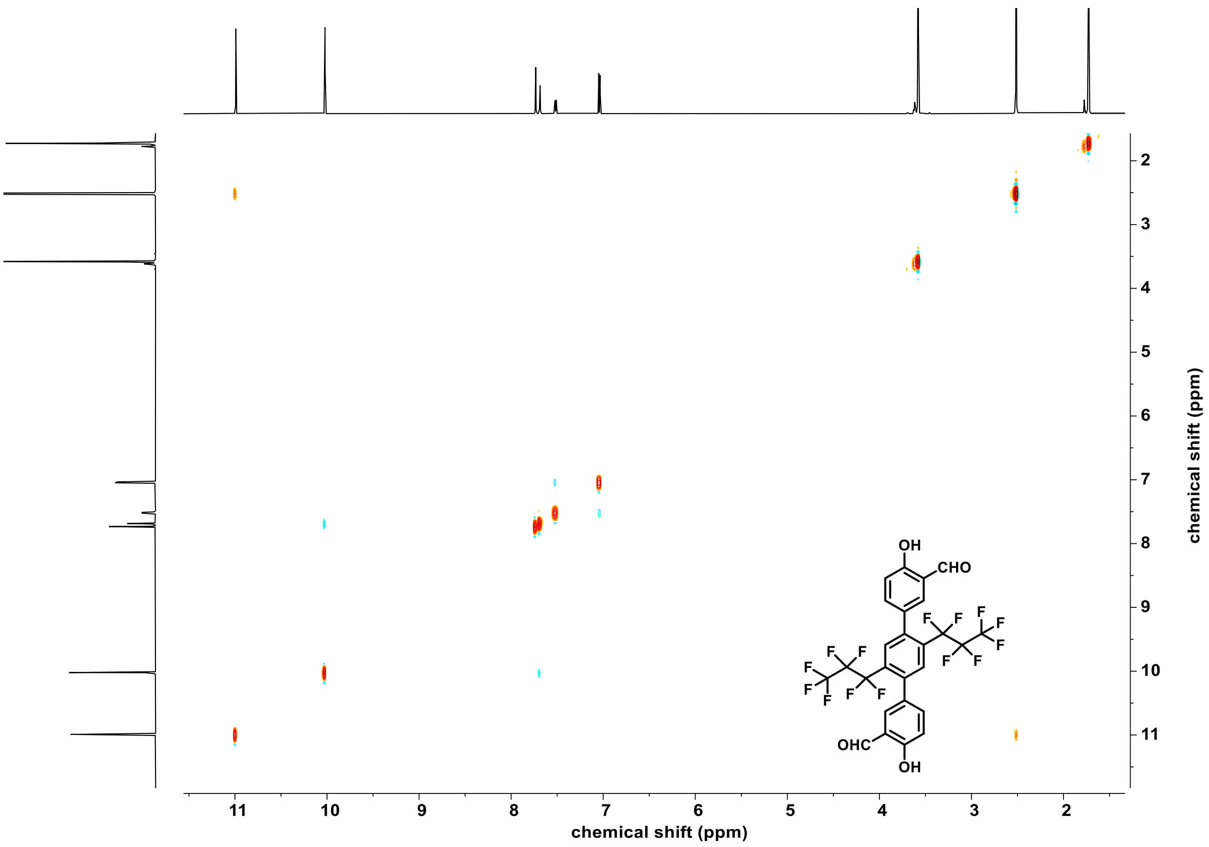

Figure S30. ^{1}H-^{1}H NOSY NMR spectrum of **2c** (THF-d8, 600 MHz, 600 MHz).

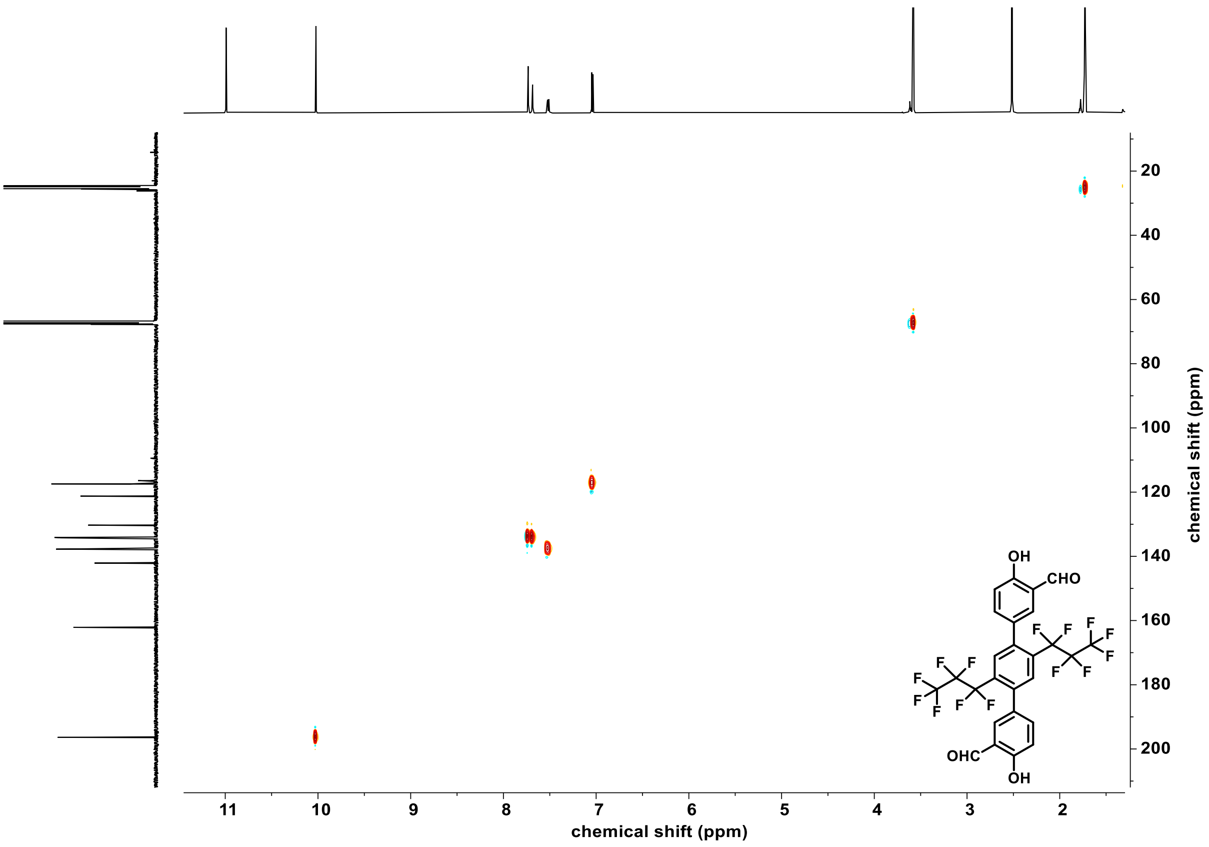

Figure S31. ^{1}H-^{13}C HSQC NMR spectrum of **2c** (THF-d_8, 600 MHz, 126 MHz).

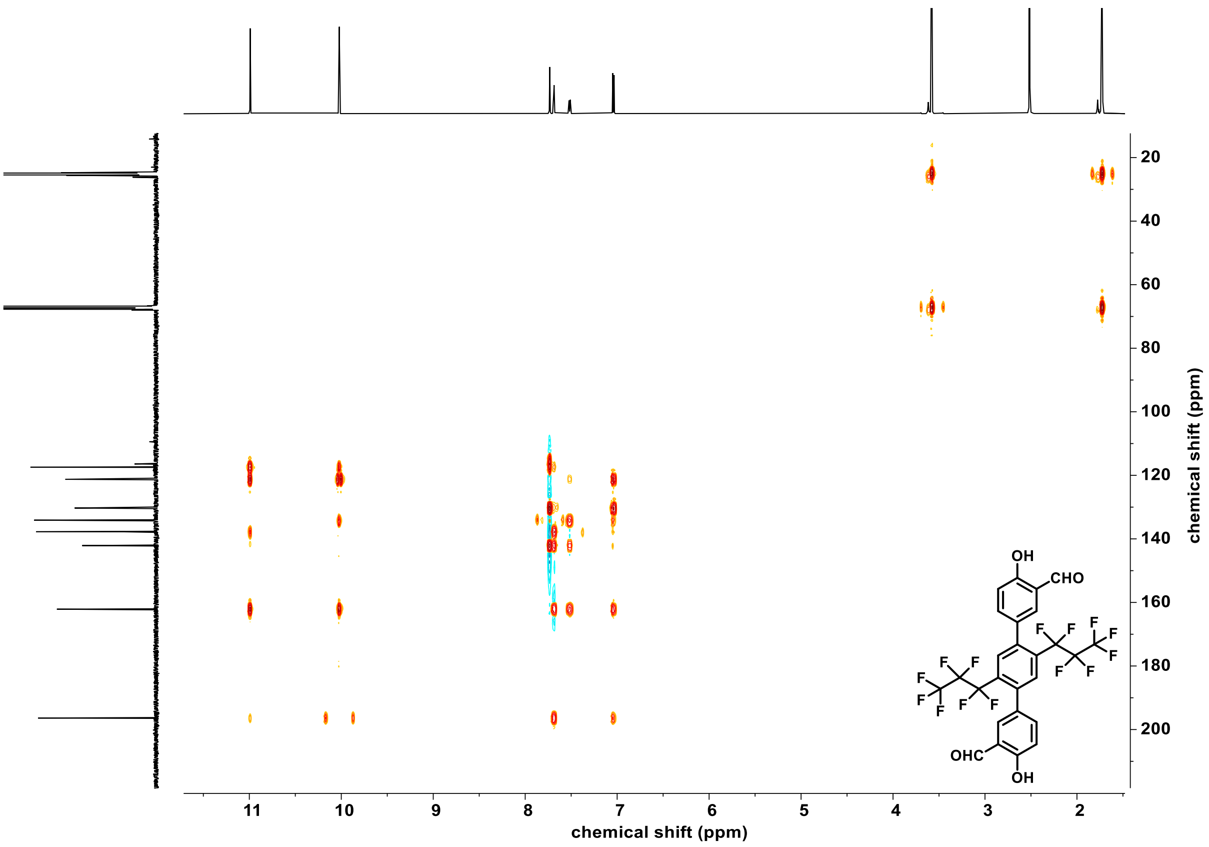

Figure S32. ^{1}H-^{13}C HMBC NMR spectrum of **2c** (THF-d_8, 600 MHz, 126 MHz).

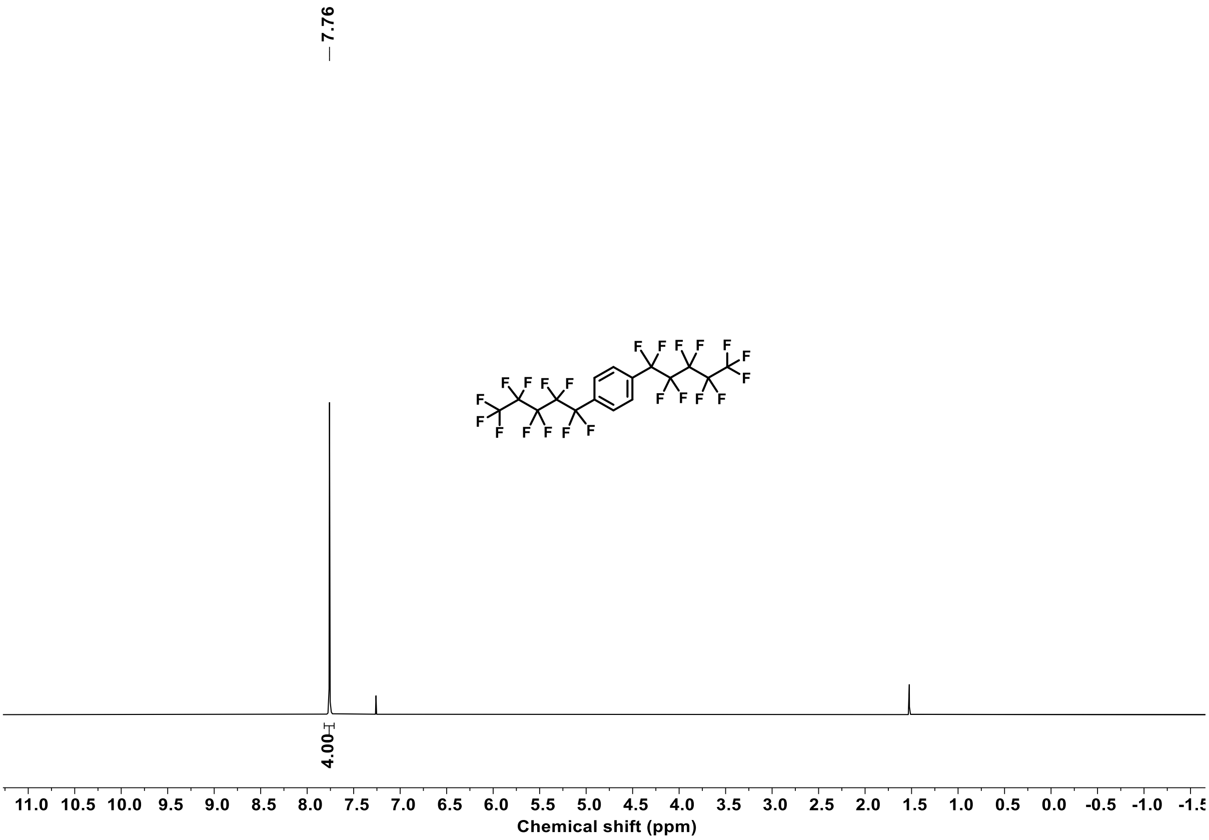

Figure S33. ^{1}H NMR spectrum of **S11** ($CDCl_3$, 400 MHz).

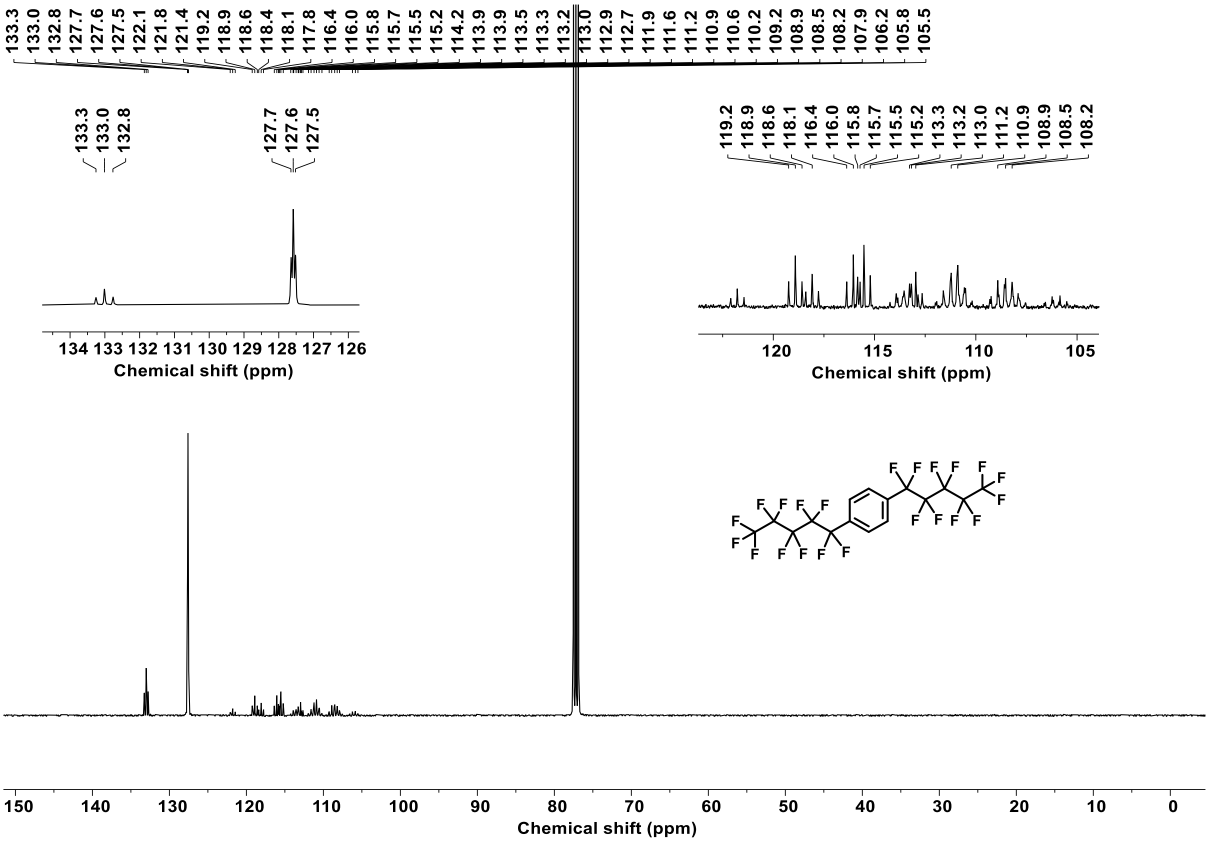

Figure S34. ^{13}C {^{1}H} NMR spectrum of **S11** ($CDCl_3$, 101 MHz).

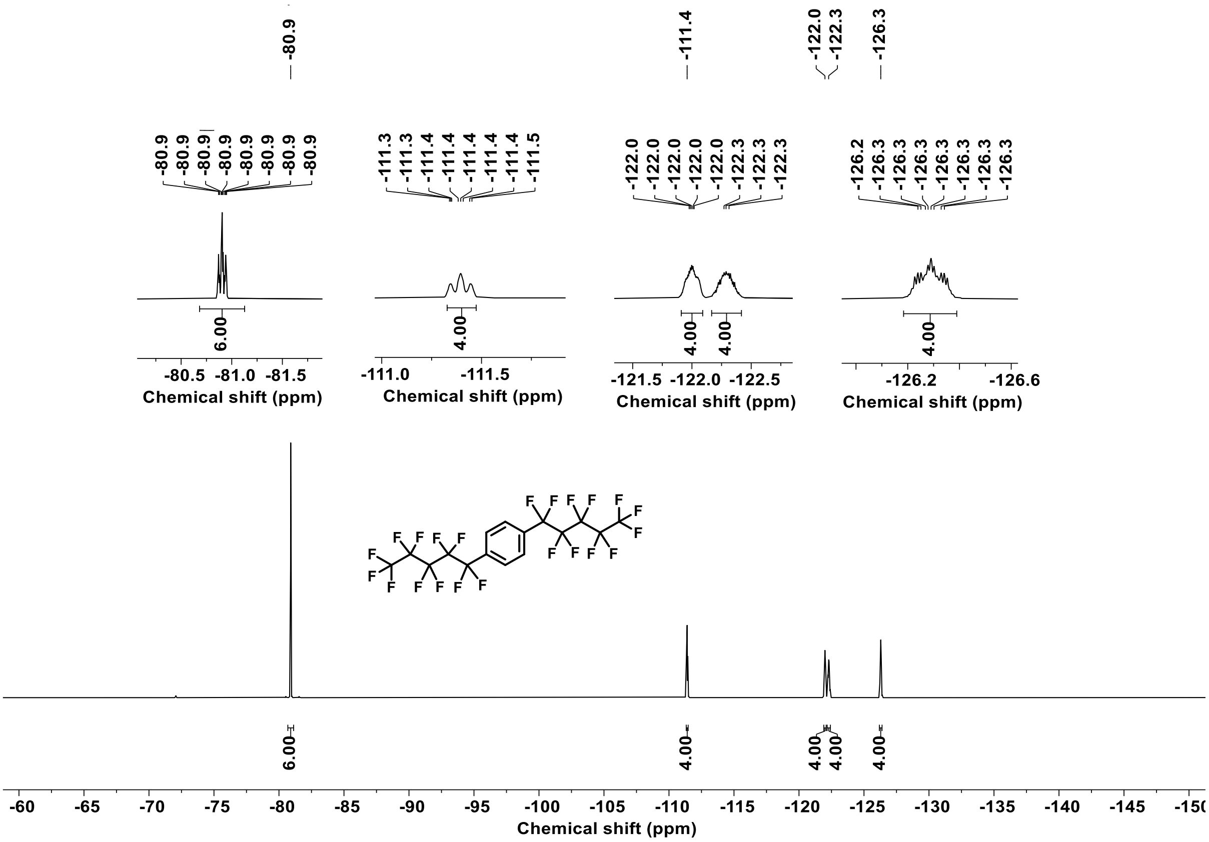

Figure S35. $^{19}F\ \{^{1}H\}$ NMR spectrum of **S11** ($CDCl_3$, 283 MHz).

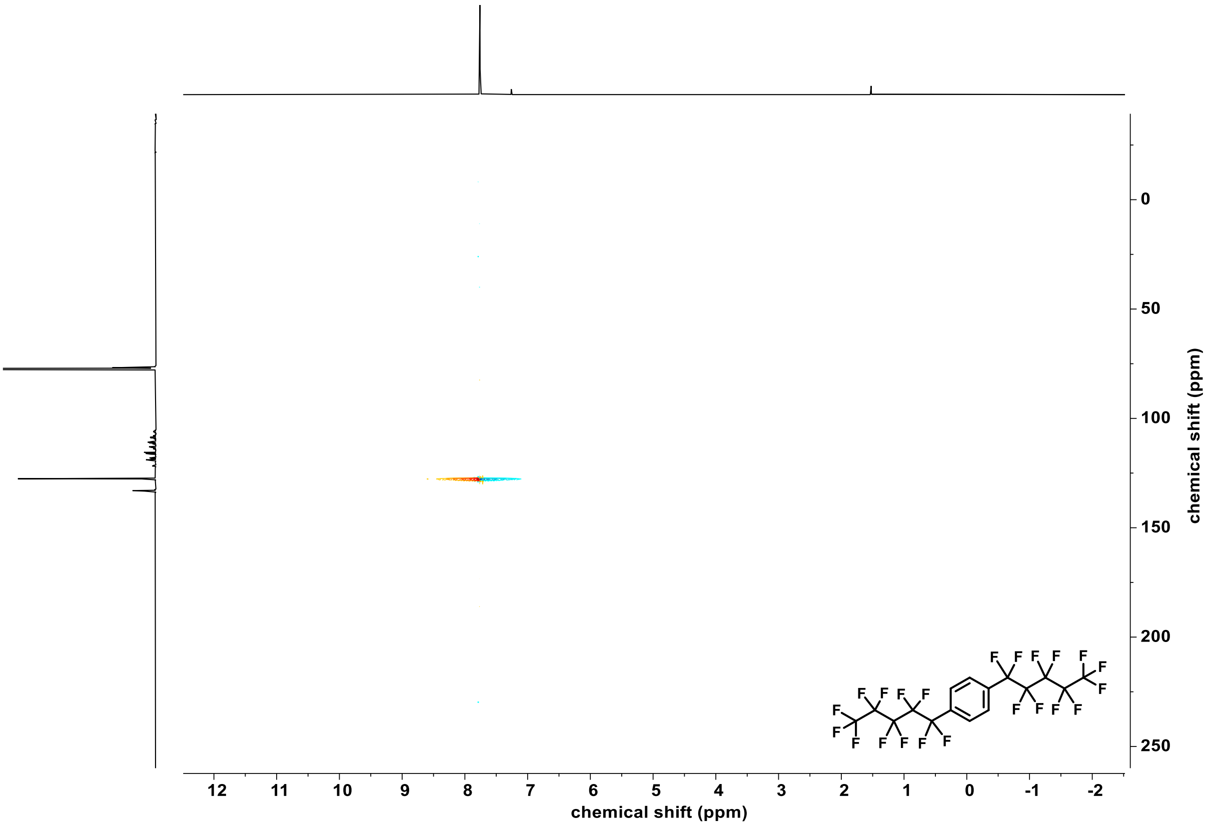

Figure S36. ^{1}H-^{13}C HSQC NMR spectrum of **S11** ($CDCl_3$, 400 MHz, 101 MHz).

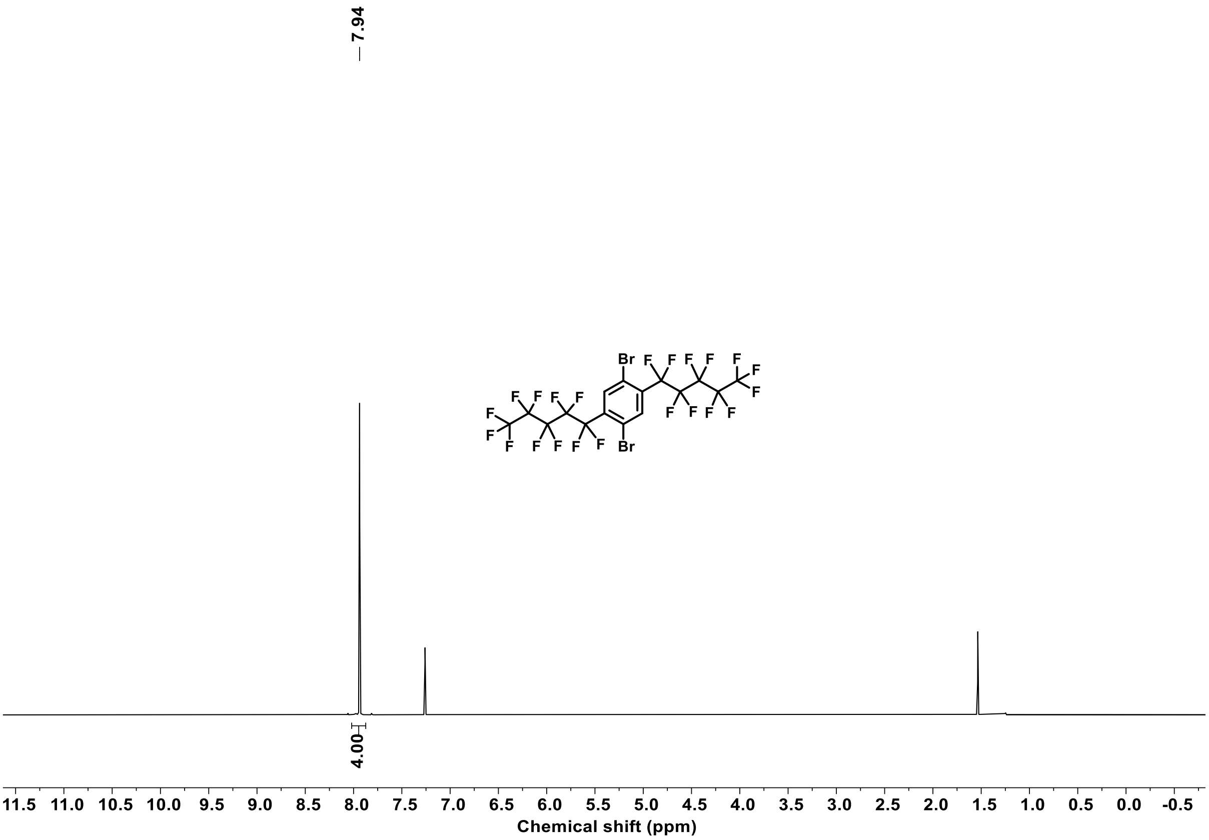

Figure S37. ^{1}H NMR spectrum of **S12** ($CDCl_3$, 700 MHz).

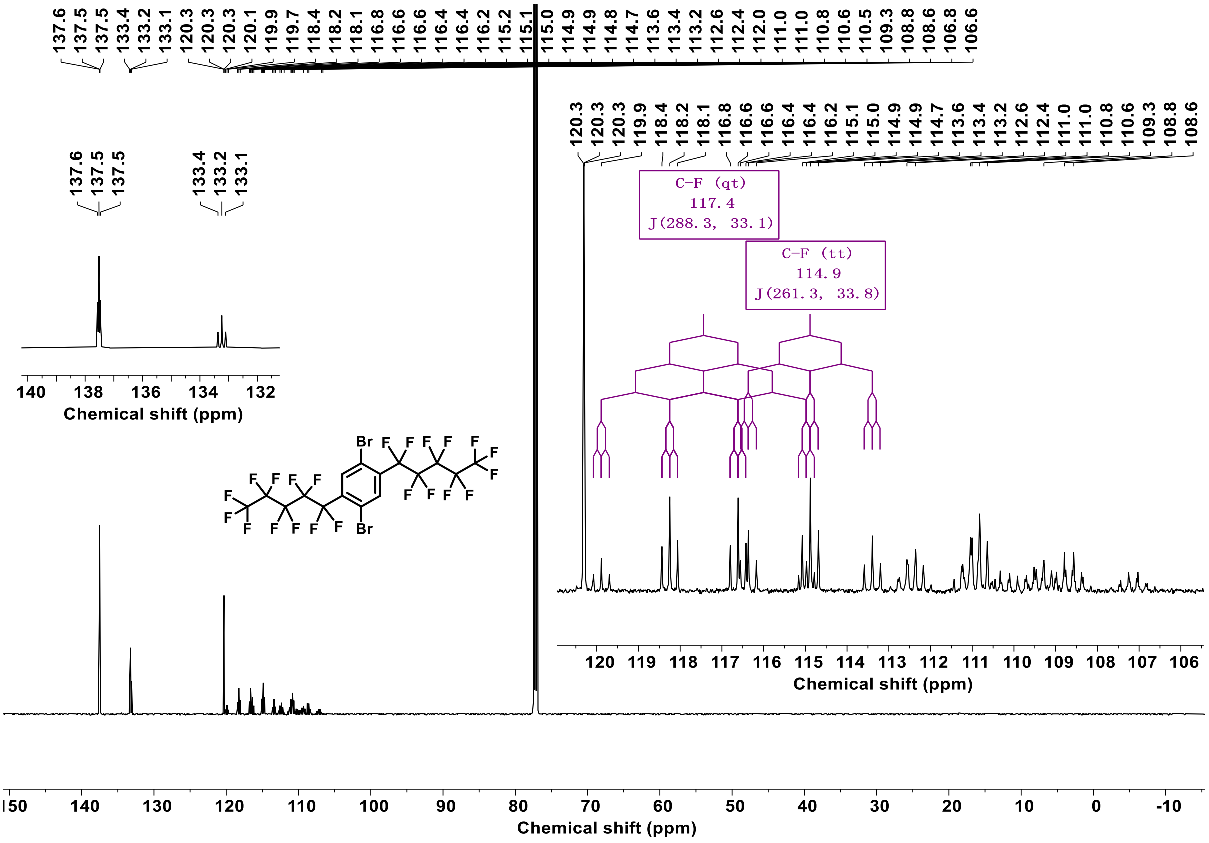

Figure S38. ^{13}C {^{1}H} NMR spectrum of **S12** ($CDCl_3$, 176 MHz).

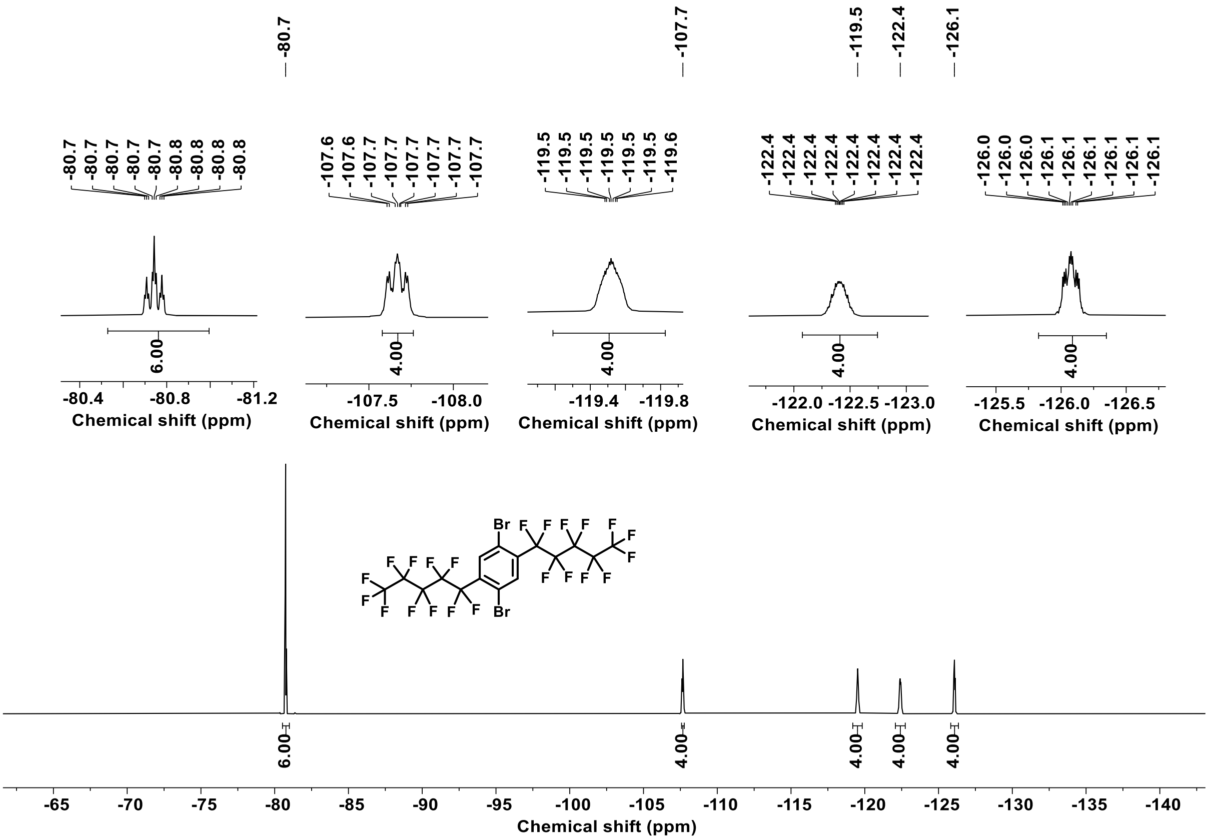

Figure S39. ^{19}F {^{1}H} NMR spectrum of **S12** ($CDCl_3$, 283 MHz).

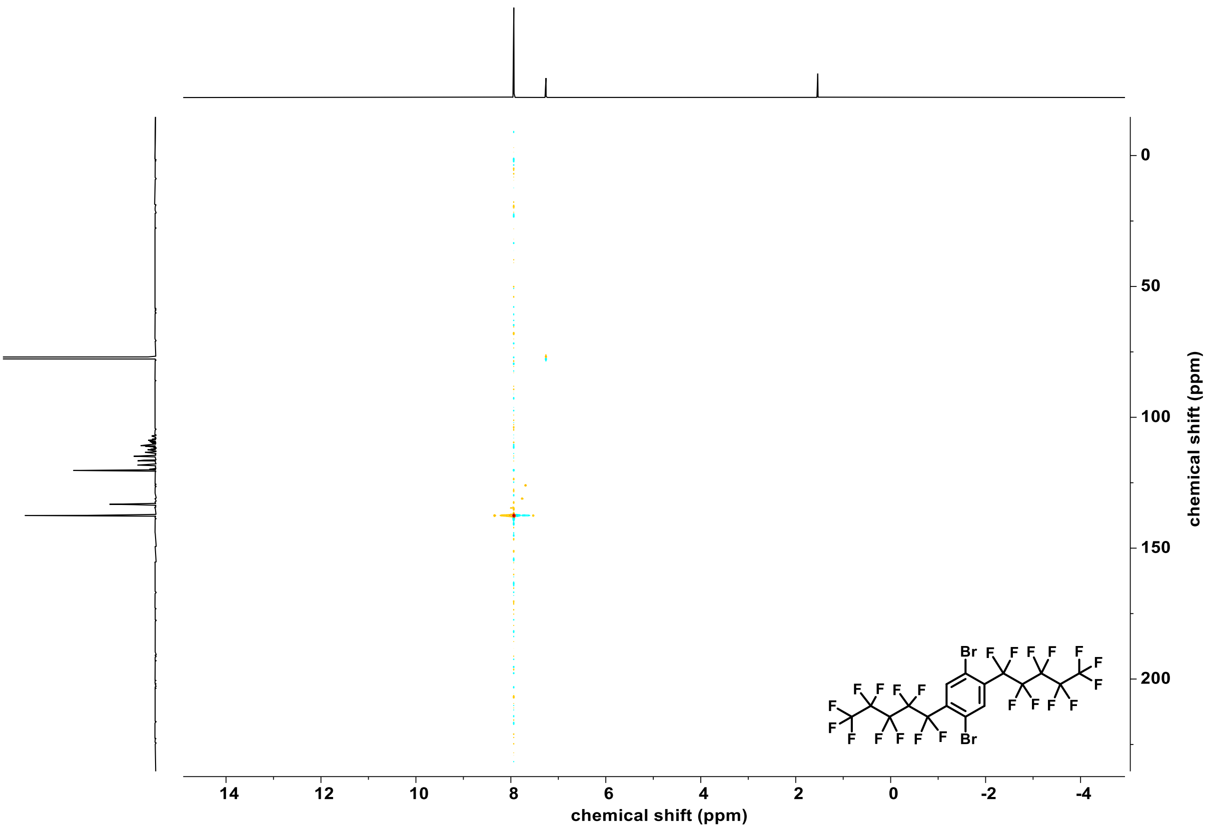

Figure S40. ^{1}H-^{13}C HSQC NMR spectrum of **S12** ($CDCl_3$, 700 MHz, 176 MHz).

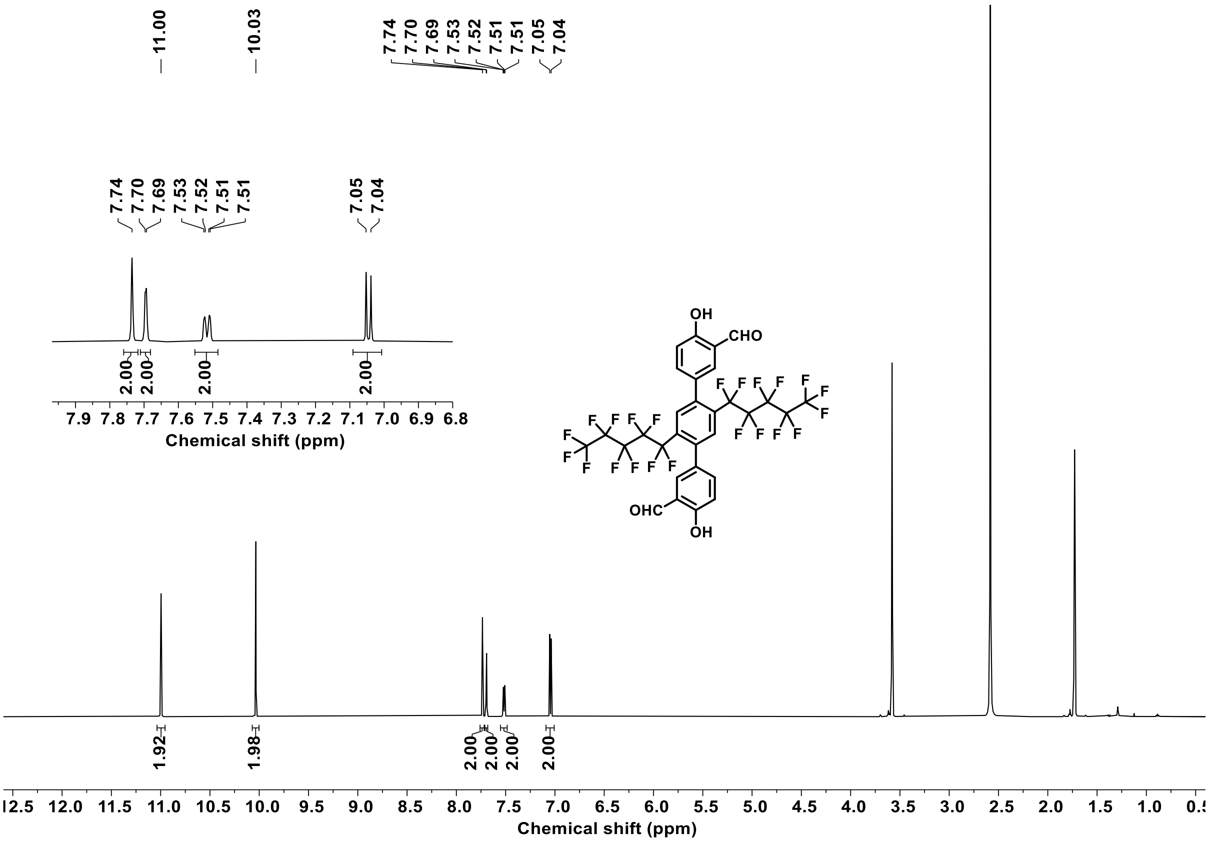

Figure S41. ^{1}H NMR spectrum of **2e** (THF-d$_8$, 600 MHz).

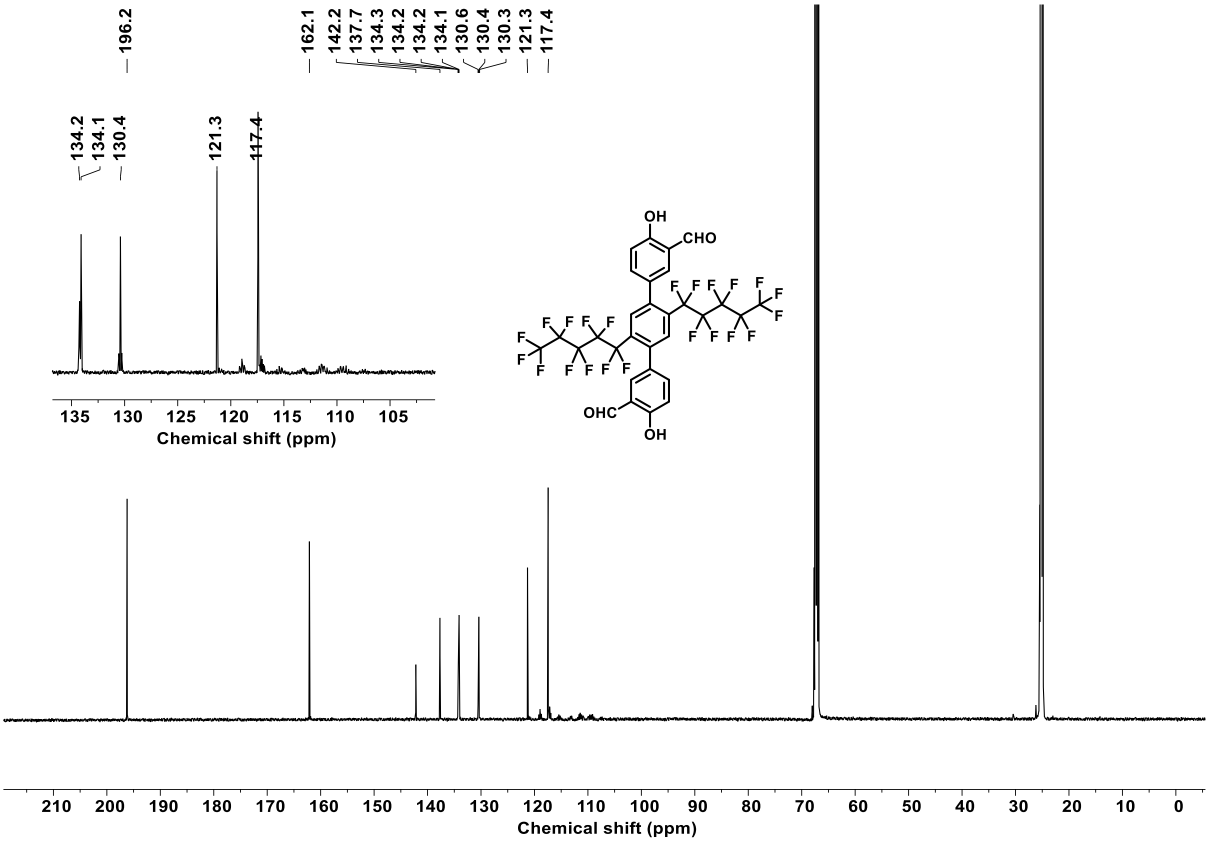

Figure S42. ^{13}C {^{1}H} NMR spectrum of **2e** (THF-d$_8$, 151 MHz).

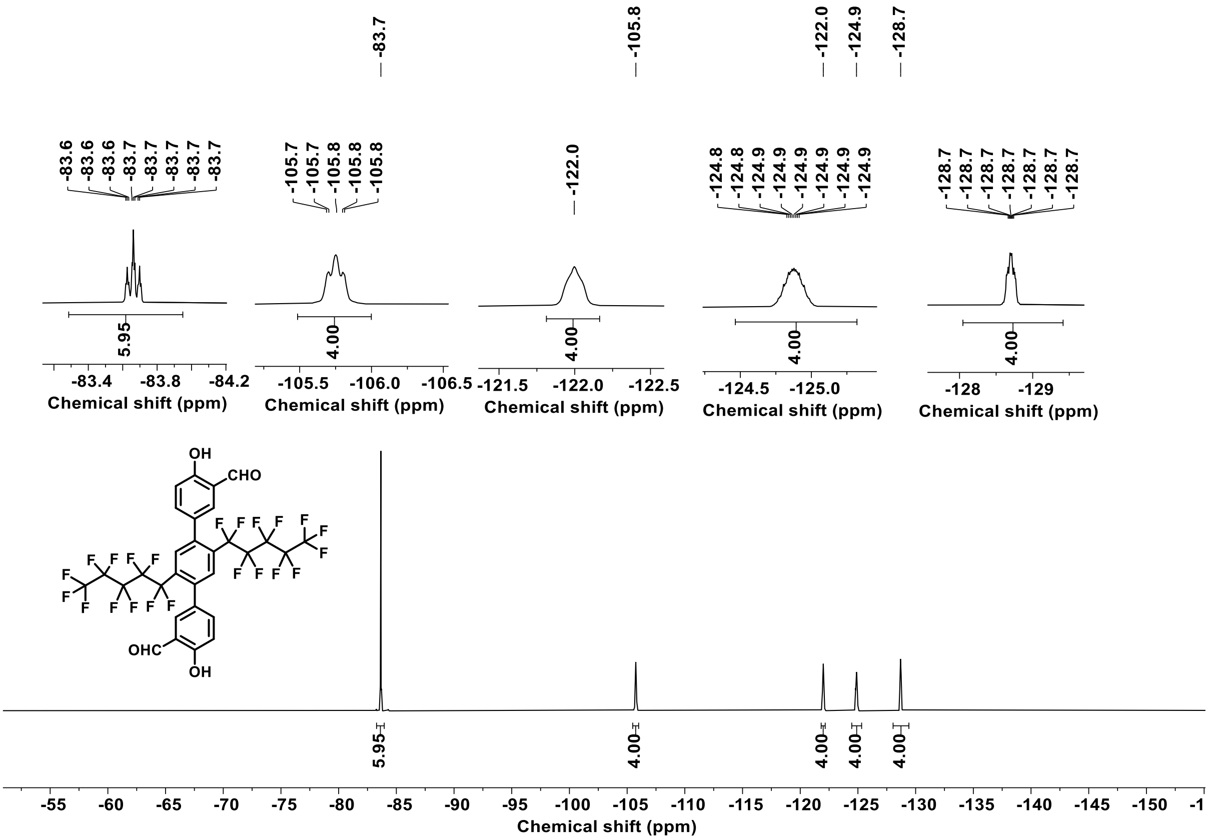

Figure S43. $^{19}F\ \{^1H\}$ NMR spectrum of **2e** (THF-d$_8$, 283 MHz).

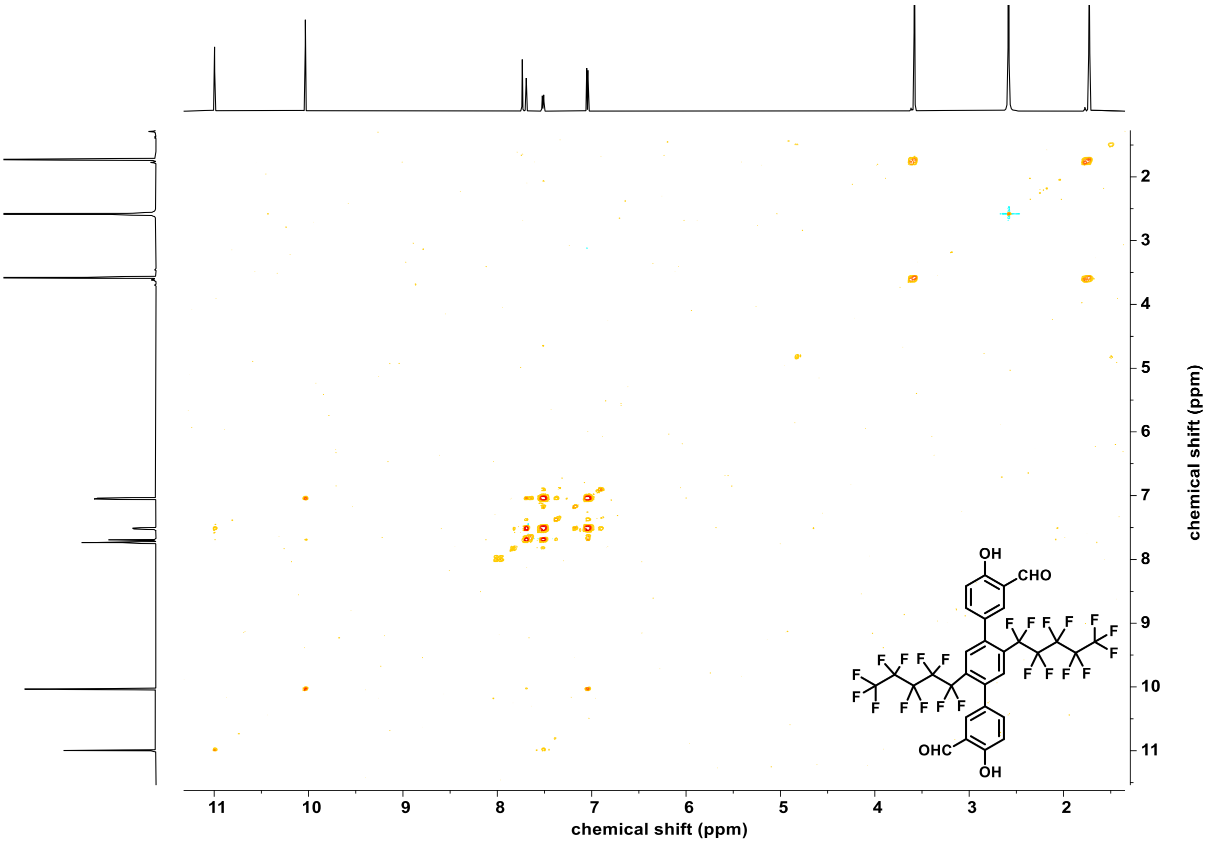

Figure S44. ^1H-^1H COSY NMR spectrum of **2e** (THF-d$_8$, 600 MHz, 600 MHz).

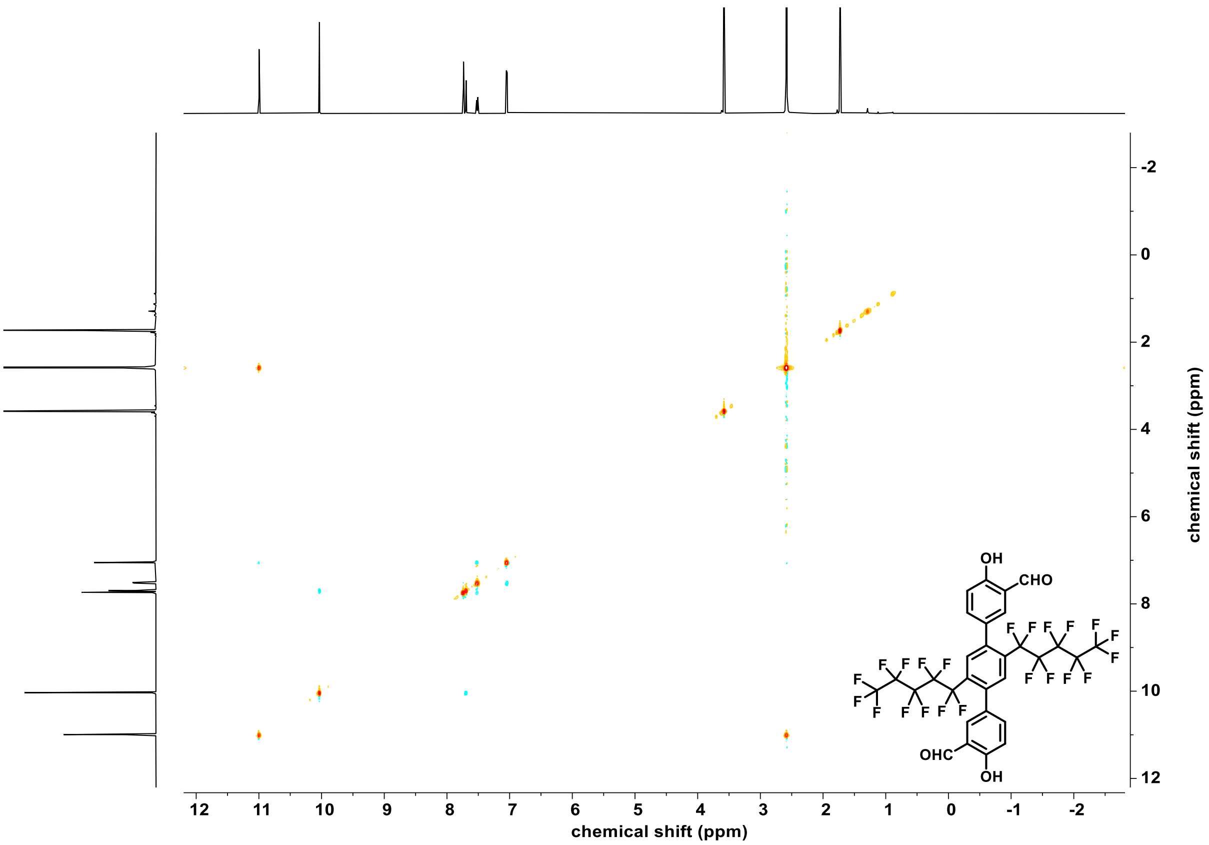

Figure S45. ^{1}H-^{1}H NOSY NMR spectrum of **2e** (THF-d_8, 600 MHz, 600 MHz).

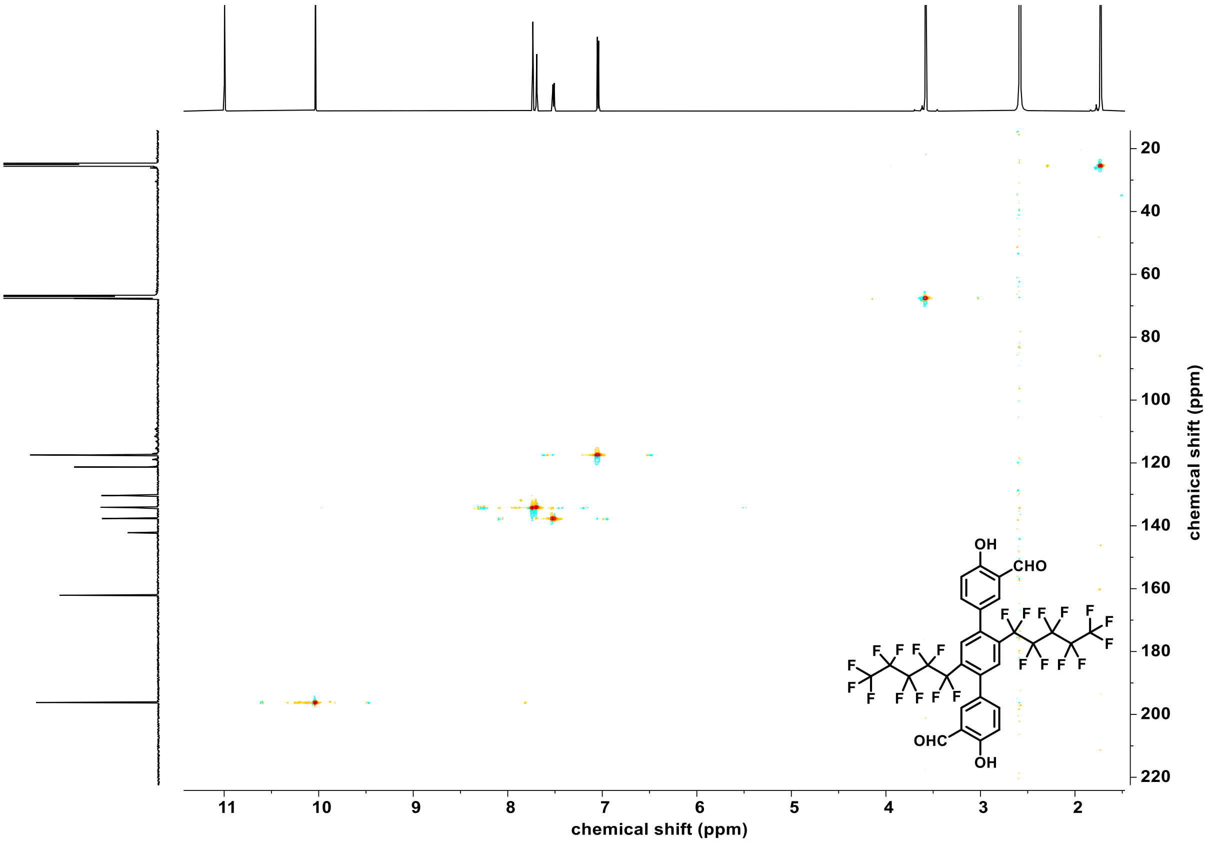

Figure S46. ^{1}H-^{13}C HSQC NMR spectrum of **2e** (THF-d_8, 600 MHz, 151 MHz).

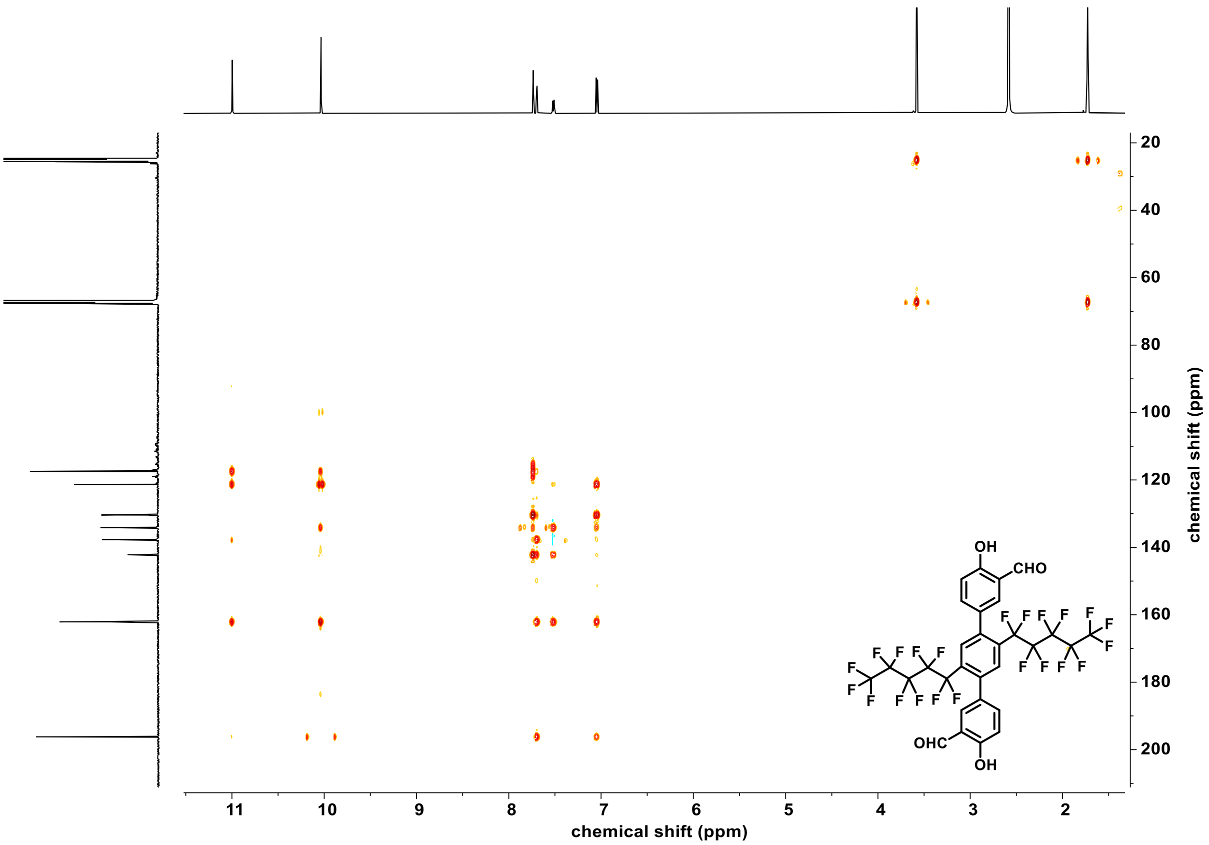

Figure S47. ^{1}H-^{13}C HMBC NMR spectrum of **2e** (THF-d_8, 600 MHz, 151 MHz).

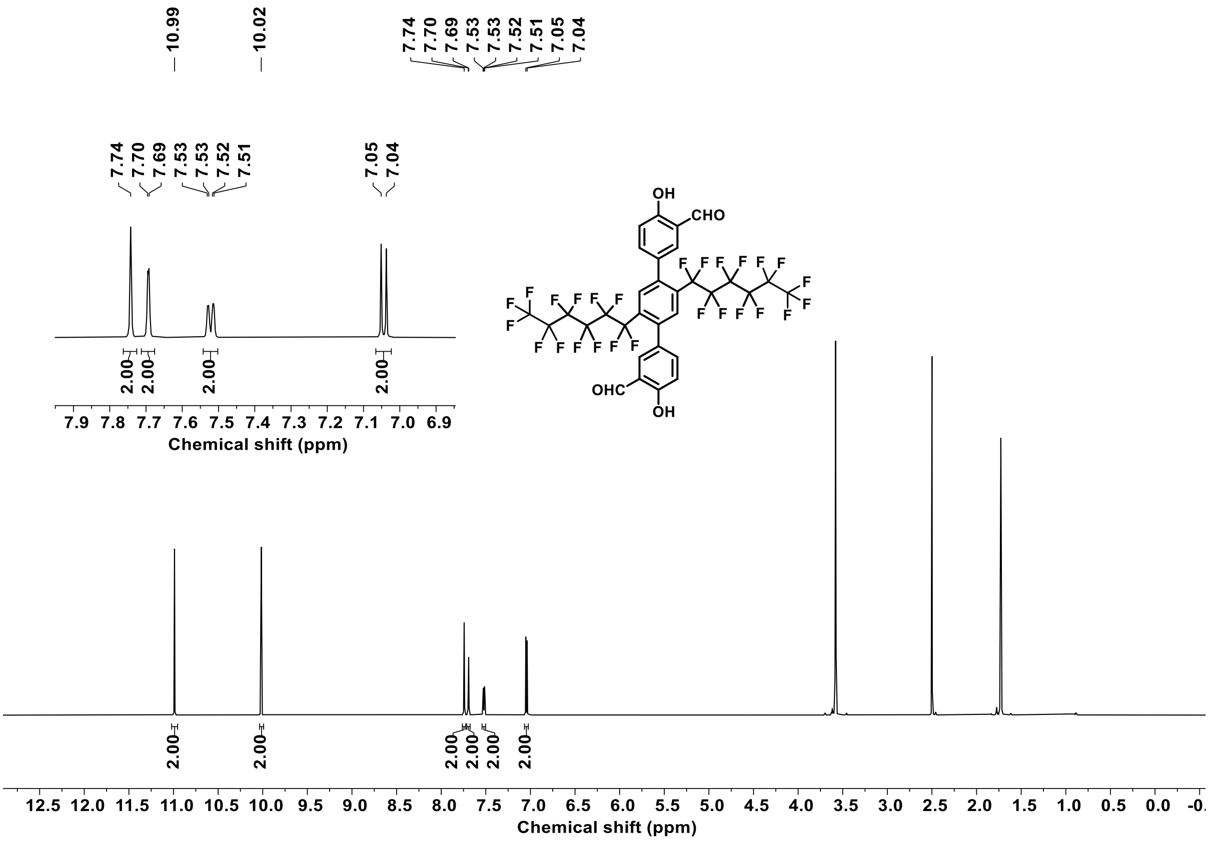

Figure S48. ^{1}H NMR spectrum of **2f** (THF-d_8, 600 MHz).

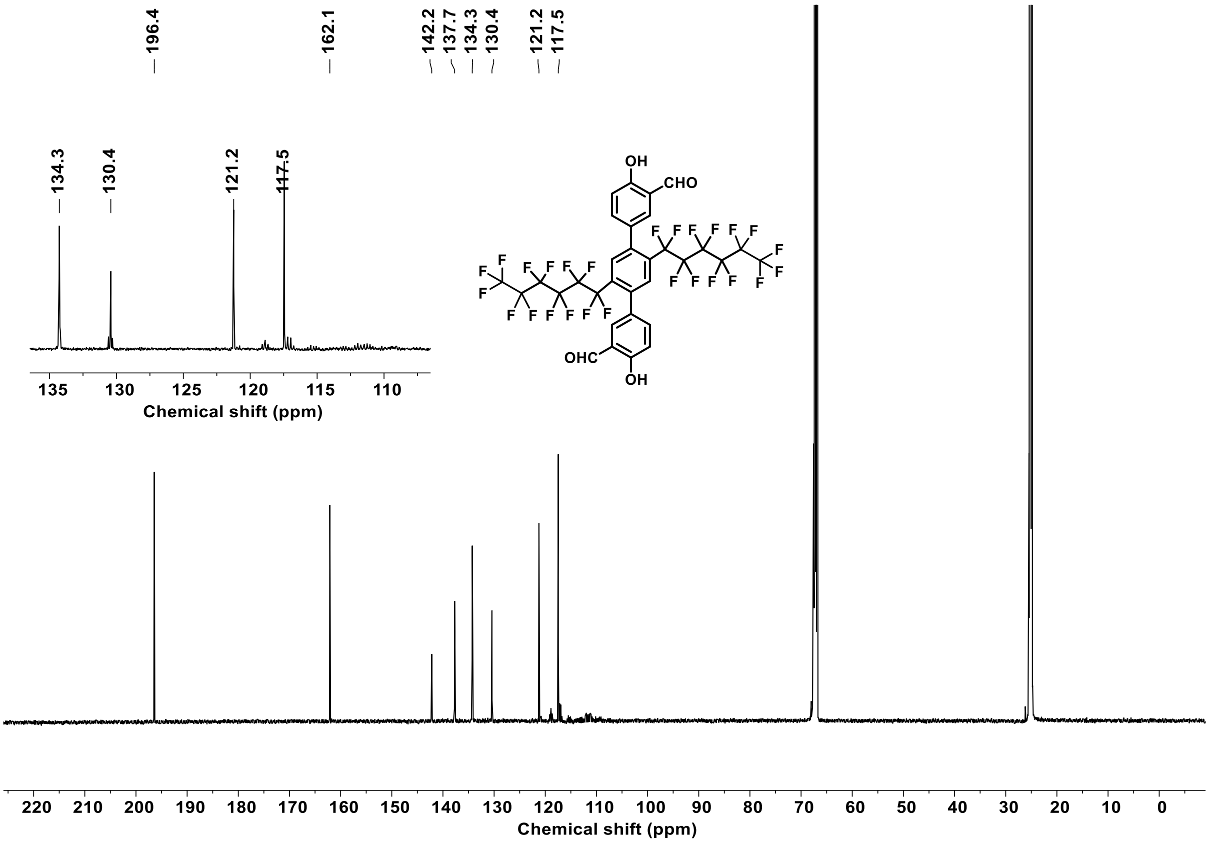

Figure S49. ^{13}C NMR spectrum of **2f** (THF-d_8, 151 MHz).

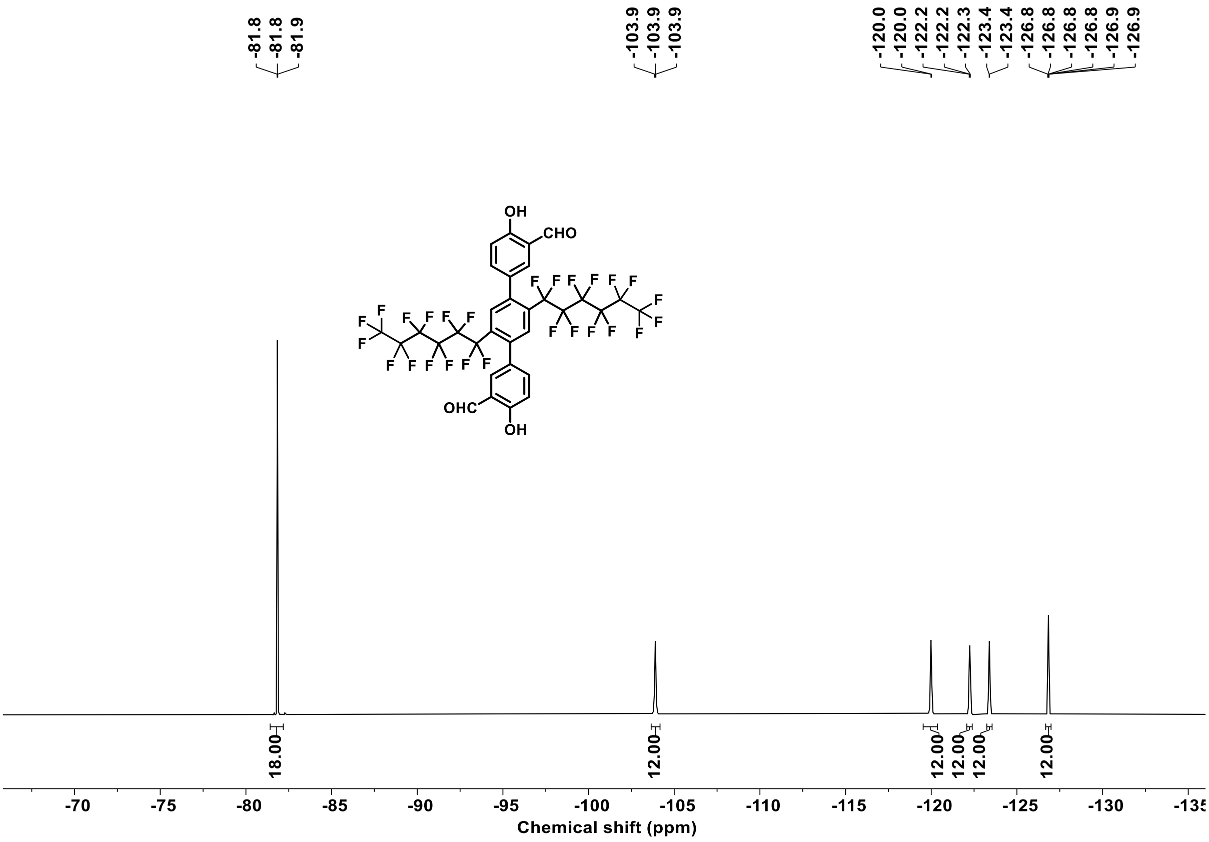

Figure S50. ^{19}F {^1H} NMR spectrum of **2f** (THF-d_8, 471 MHz).

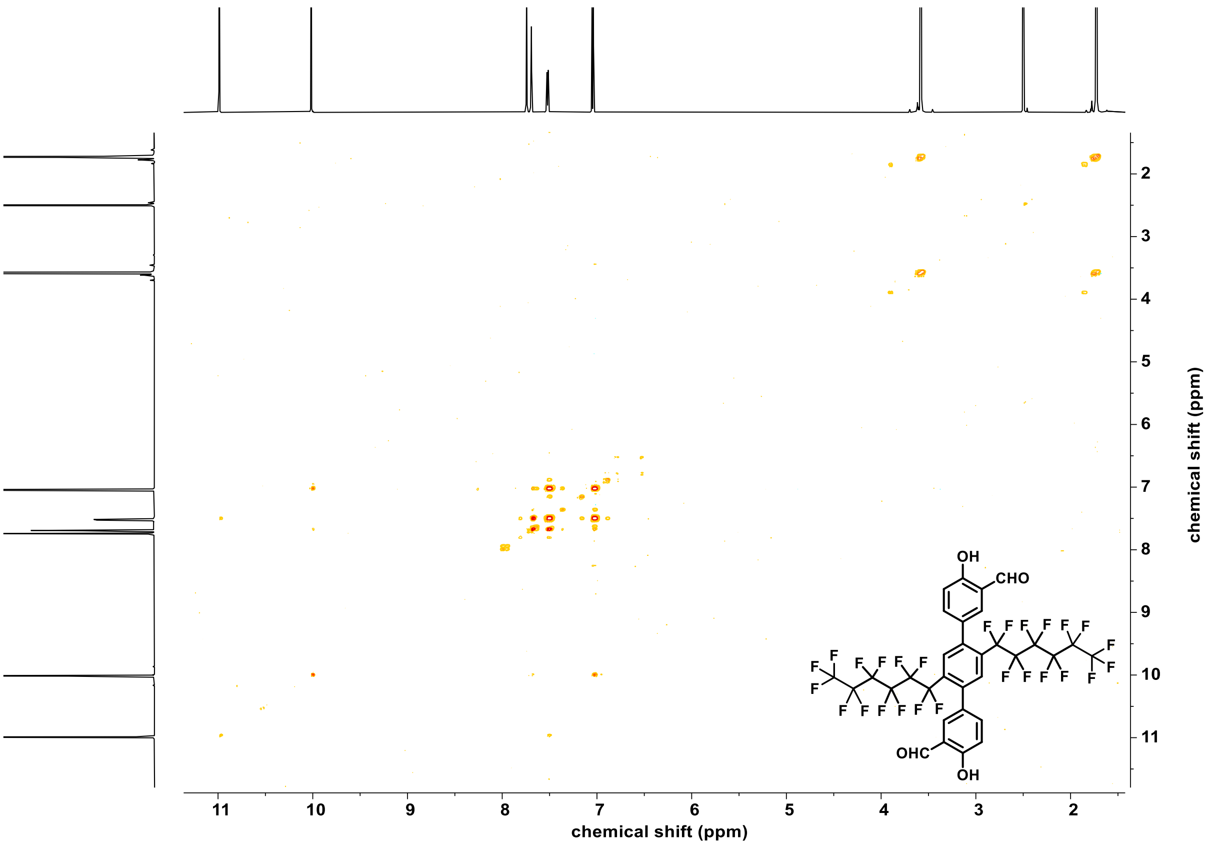

Figure S51. ^{1}H-^{1}H COSY NMR spectrum of **2f** (THF-d$_8$, 600 MHz, 600 MHz).

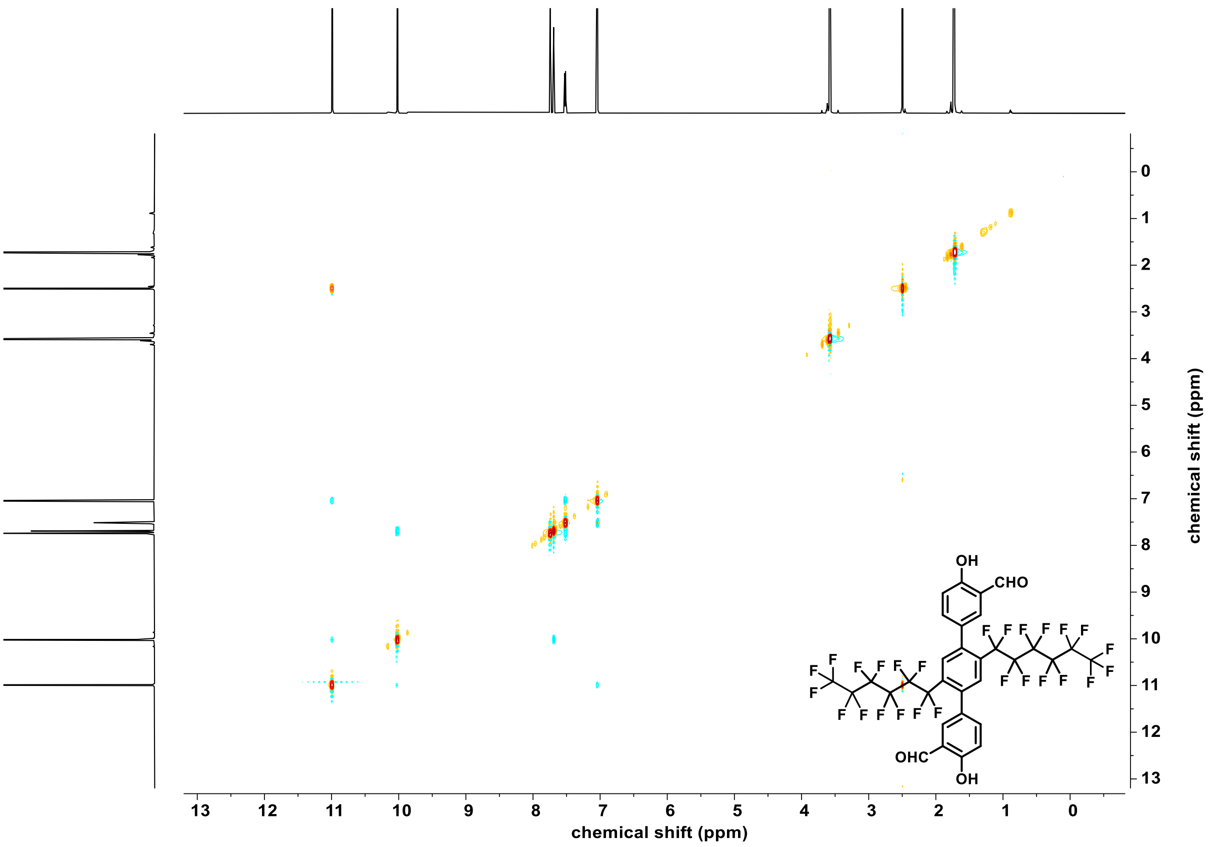

Figure S52. ^{1}H-^{1}H NOSY NMR spectrum of **2f** (THF-d$_8$, 600 MHz, 600 MHz).

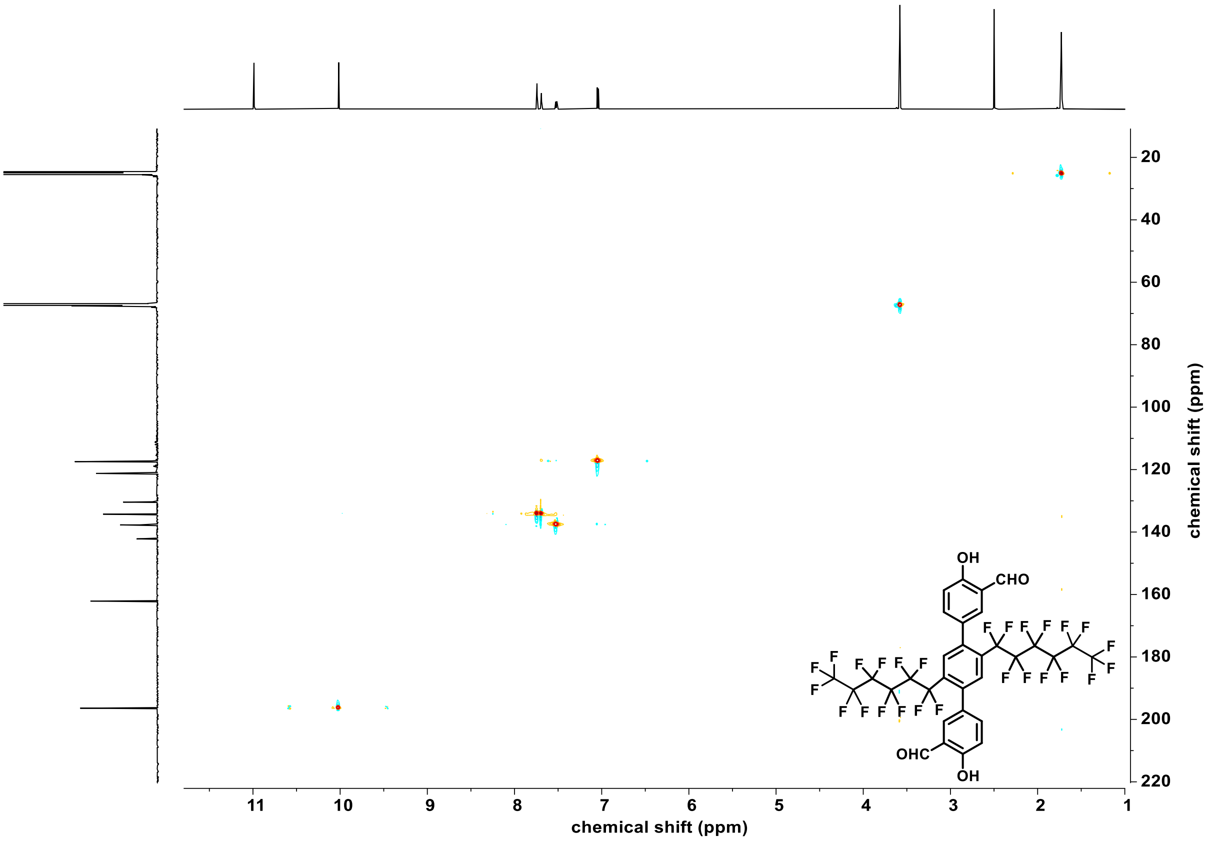

Figure S53. ^{1}H-^{13}C HSQC NMR spectrum of **2f** (THF-d_8, 600 MHz, 151 MHz).

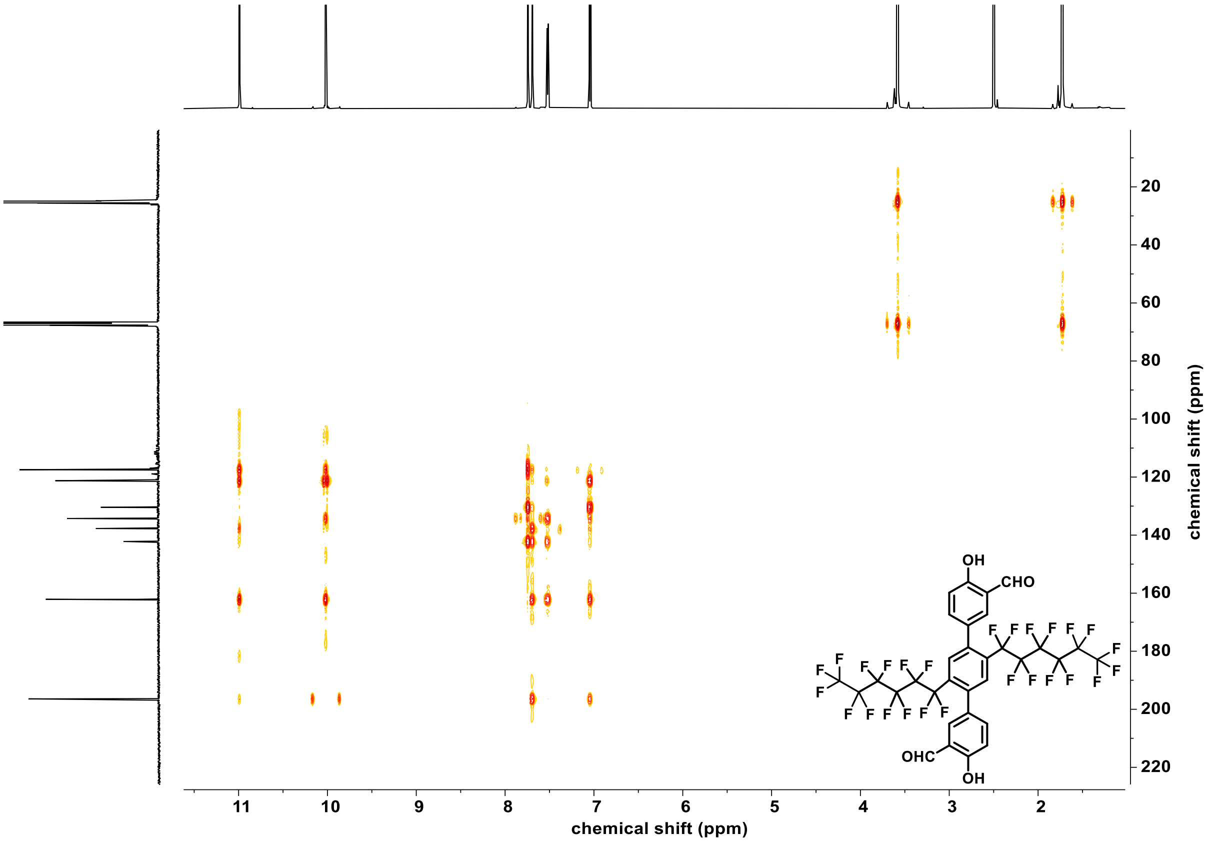

Figure S54. ^{1}H-^{13}C HMBC NMR spectrum of **2f** (THF-d_8, 600 MHz, 151 MHz).

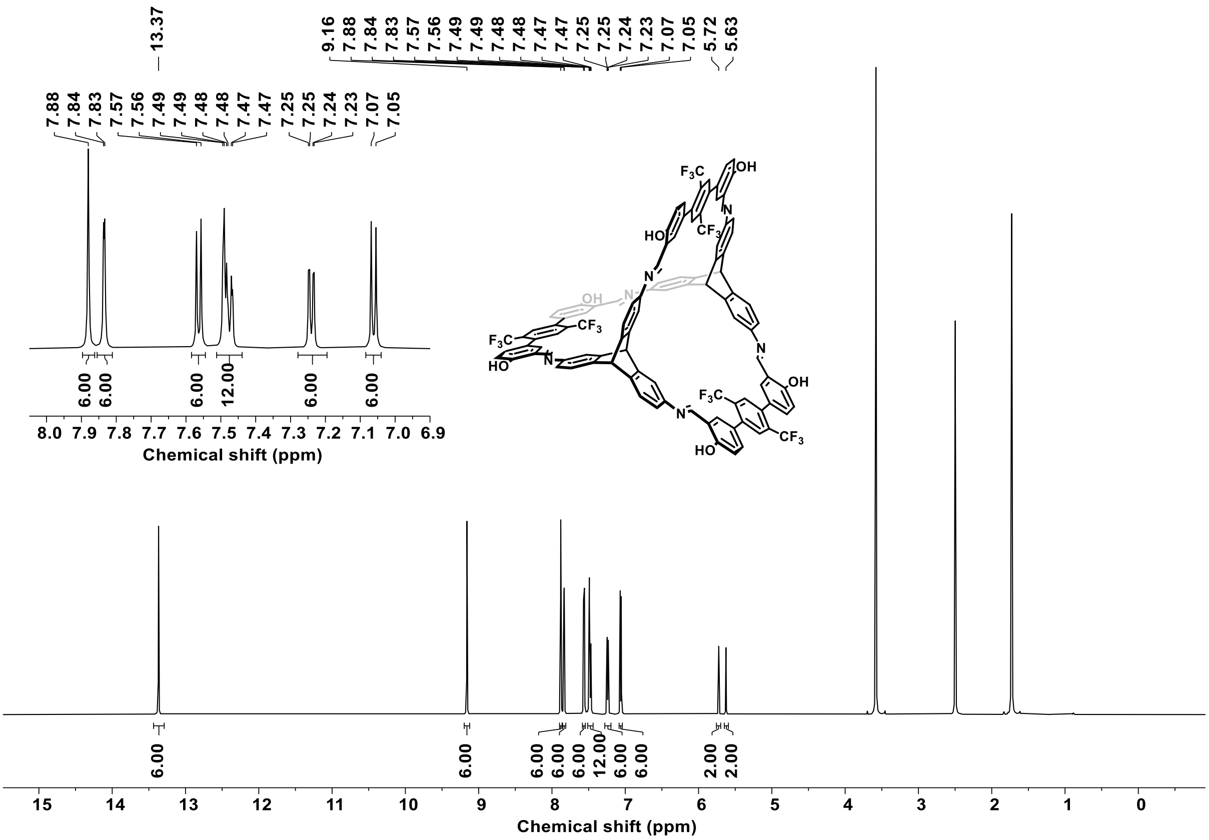

Figure S55. ^{1}H NMR spectrum of **CF_3-cage** (THF-d_8, 600 MHz).

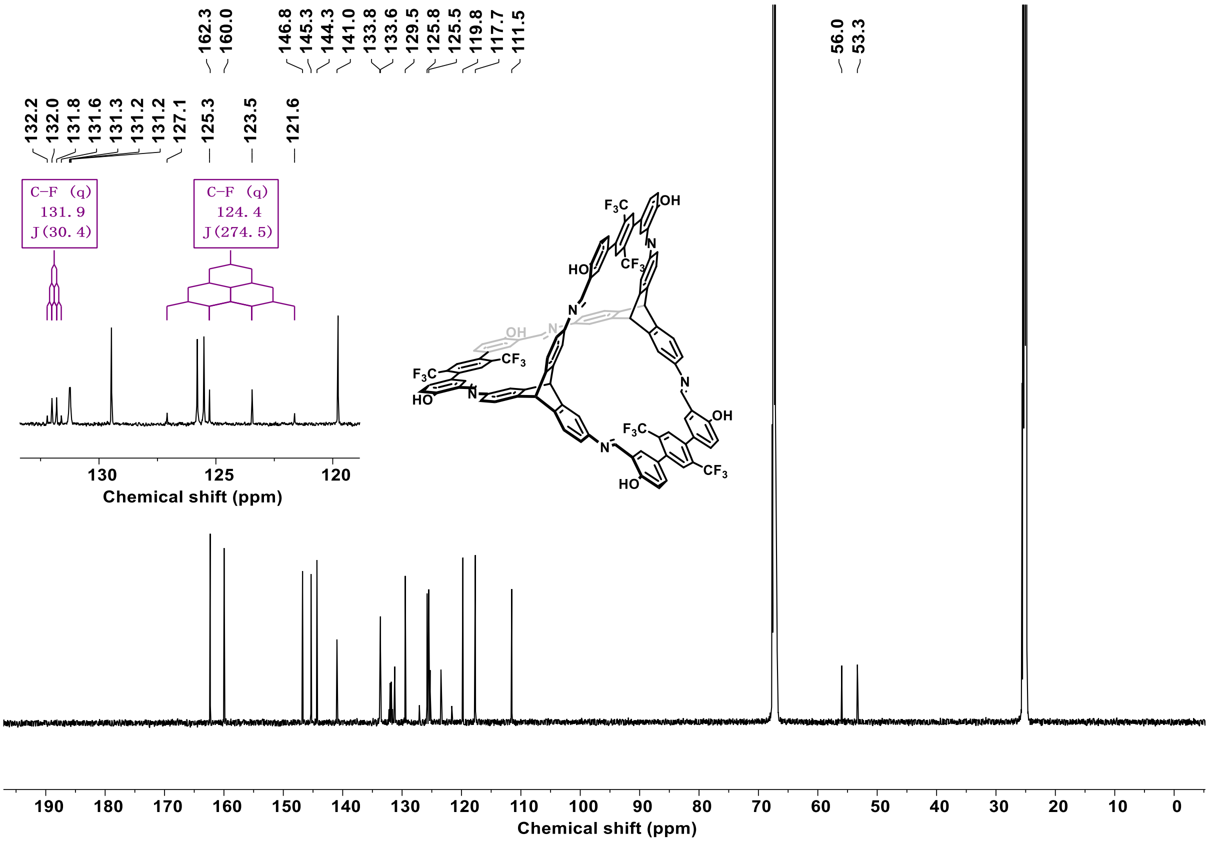

Figure S56. ^{13}C {^{1}H} NMR spectrum of **CF_3-cage** (THF-d_8, 151 MHz).

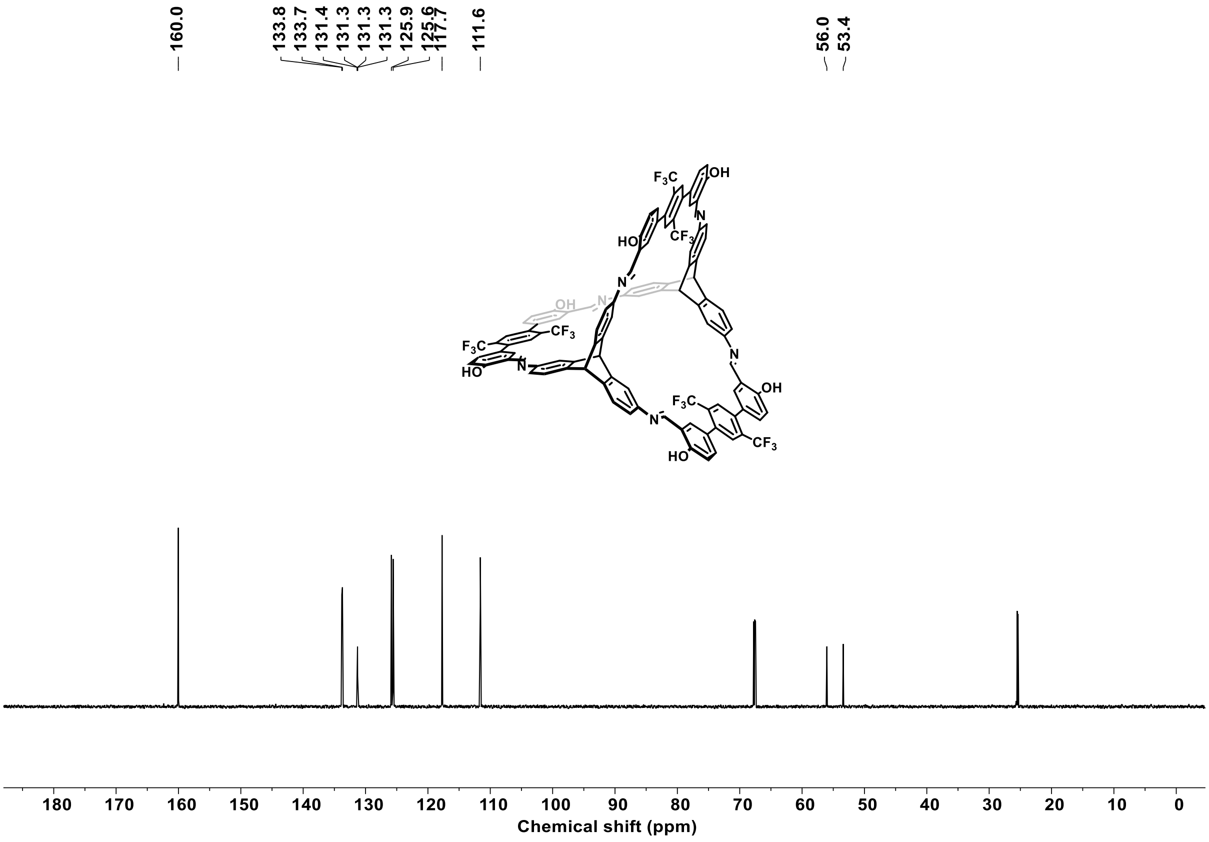

Figure S57. ^{13}C-DEPT135 NMR spectrum of **CF_3-cage** (THF-d_8, 151 MHz).

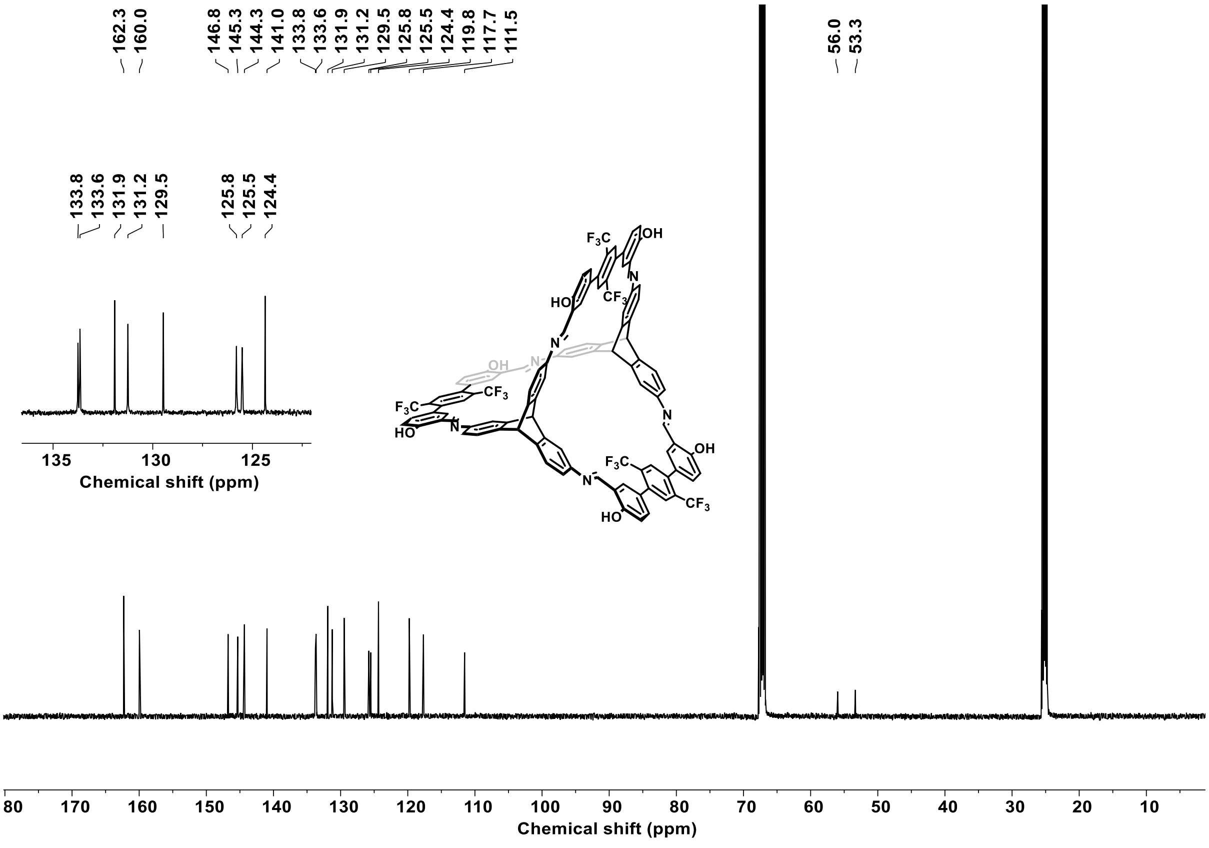

Figure S58. ^{13}C {^{1}H, ^{19}F} NMR spectrum of **CF_3-cage** (THF-d_8, 126 MHz).

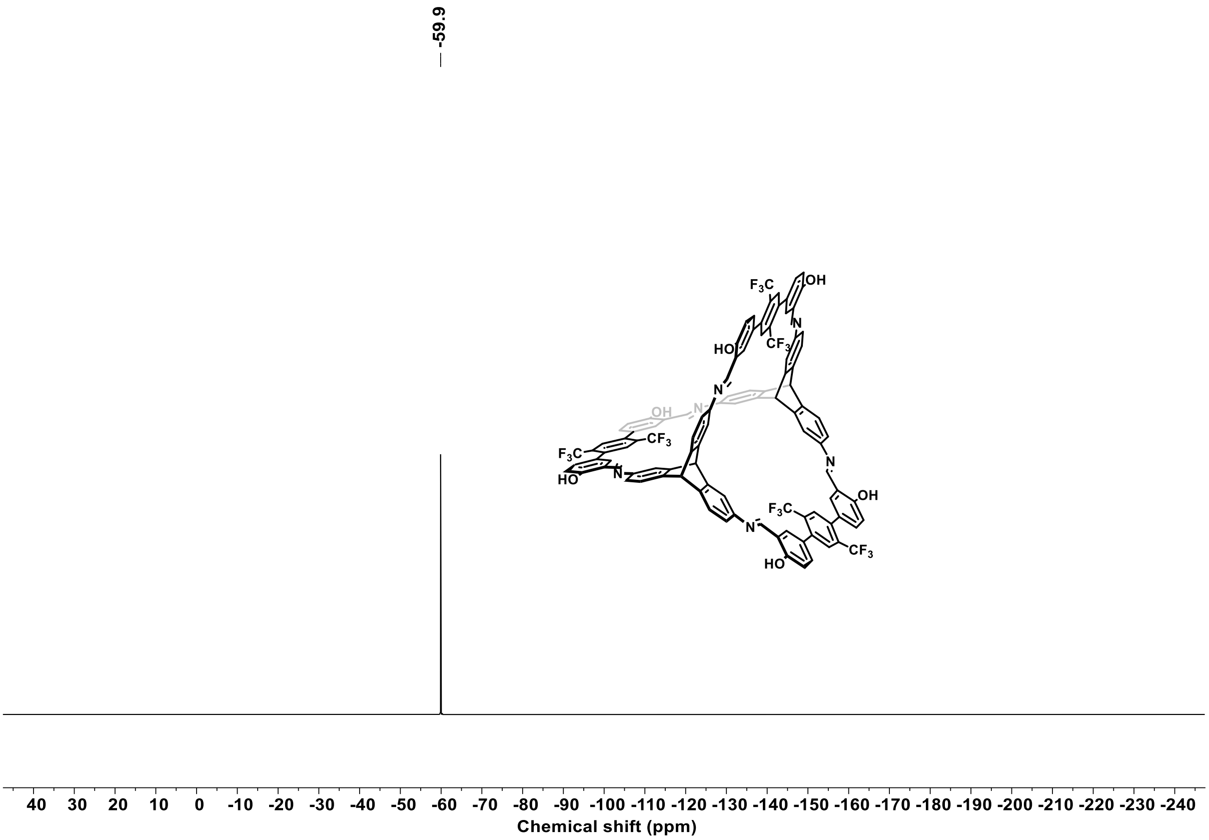

Figure S59. ^{19}F {^{1}H} NMR spectrum of **CF$_3$-cage** (THF-d$_8$, 471 MHz).

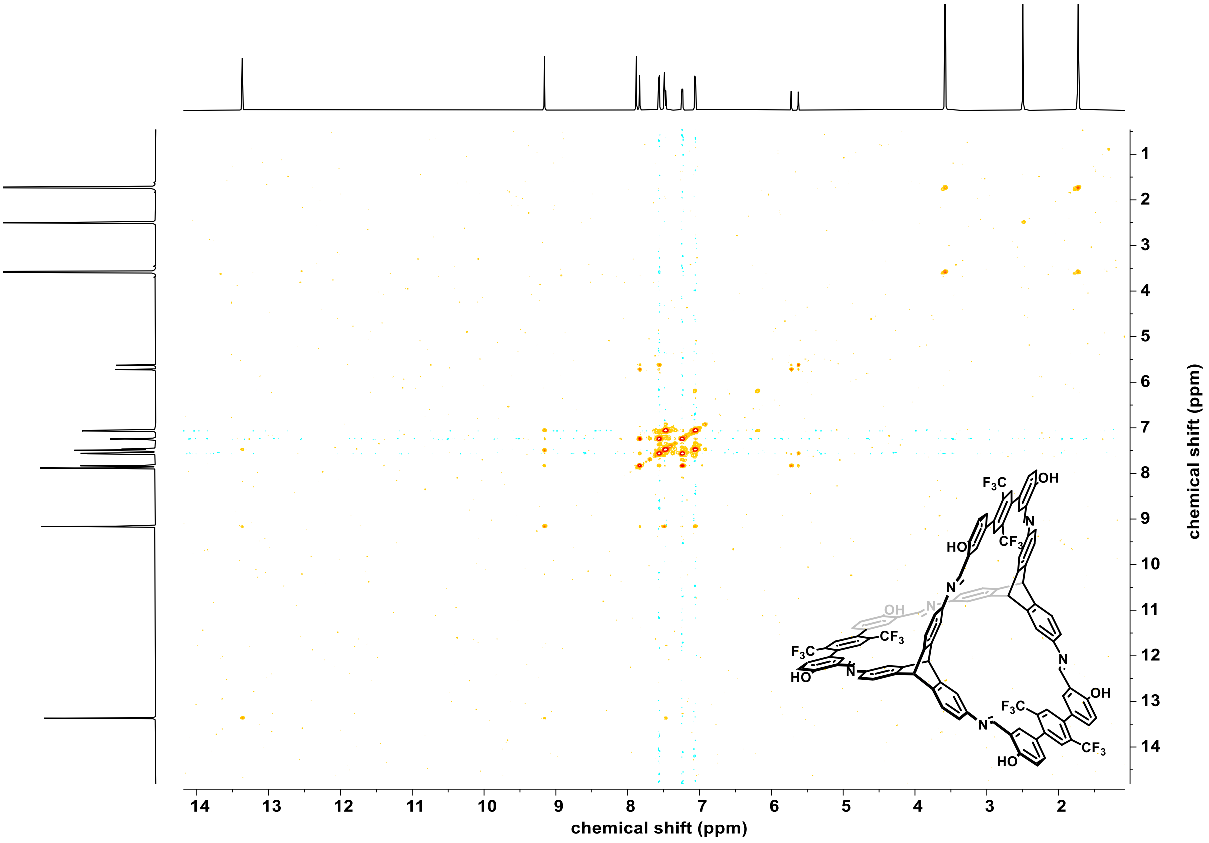

Figure S60. ^{1}H-^{1}H COSY NMR spectrum of **CF$_3$-cage** (THF-d$_8$, 600 MHz, 600 MHz).

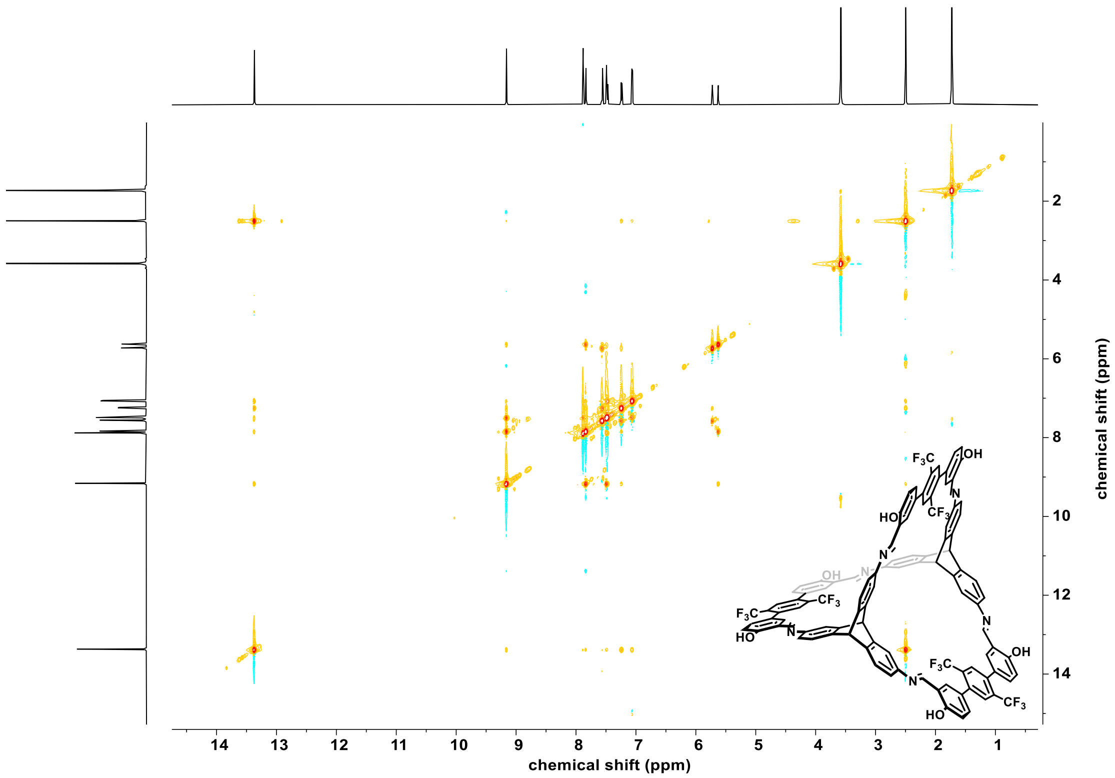

Figure S61. ^{1}H-^{1}H NOESY NMR spectrum of **CF$_3$-cage** (THF-d$_8$, 600 MHz, 600 MHz).

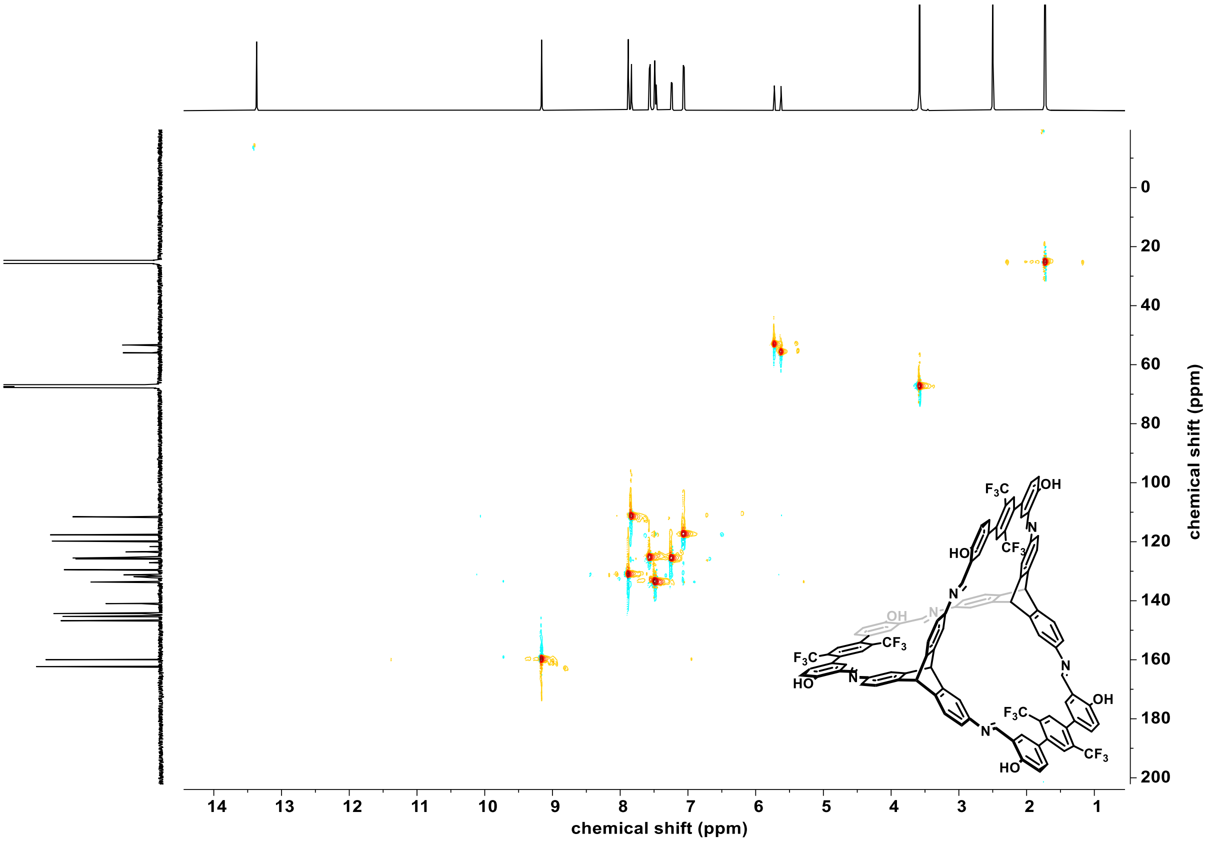

Figure S62. ^{1}H-^{13}C HSQC NMR spectrum of **CF$_3$-cage** (THF-d$_8$, 600 MHz, 151 MHz).

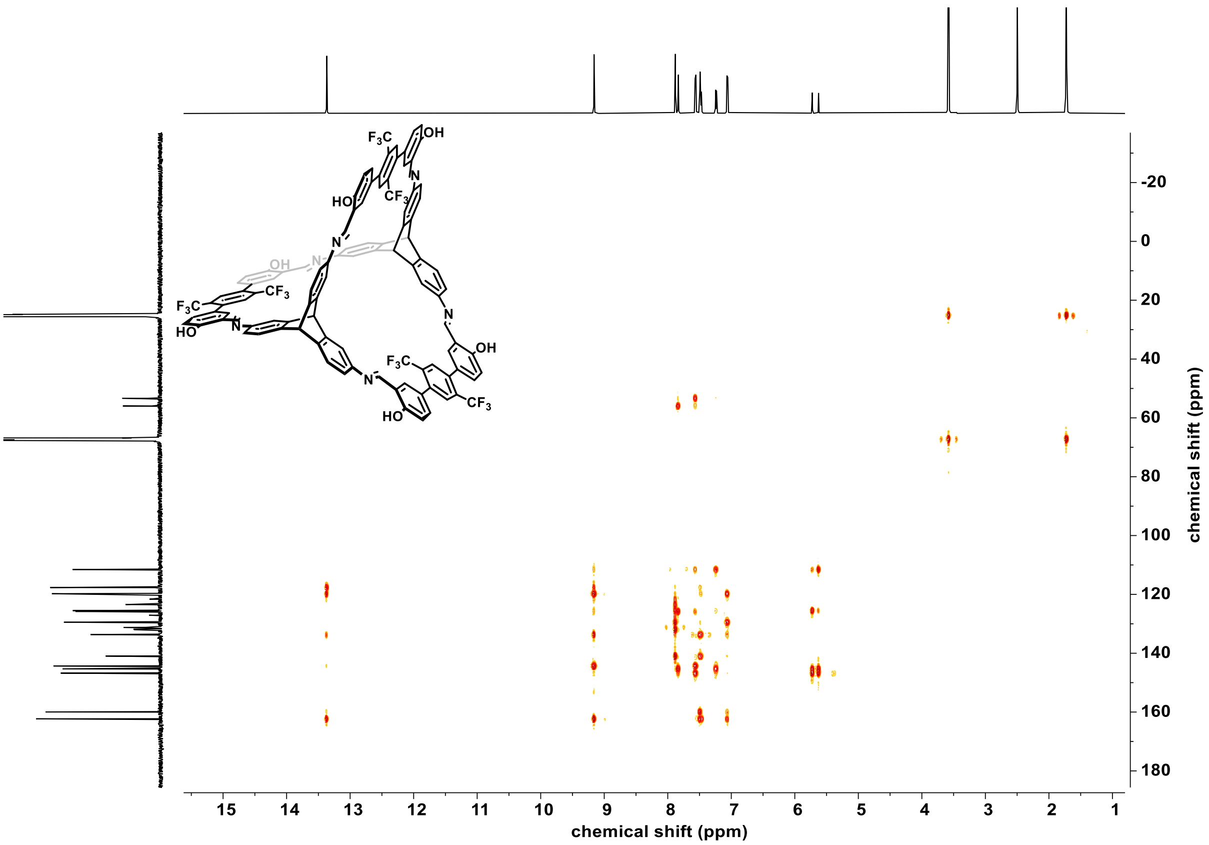

Figure S63. ^{1}H-^{13}C HMBC NMR spectrum of **CF_3-cage** (THF-d_8, 600 MHz, 151 MHz).

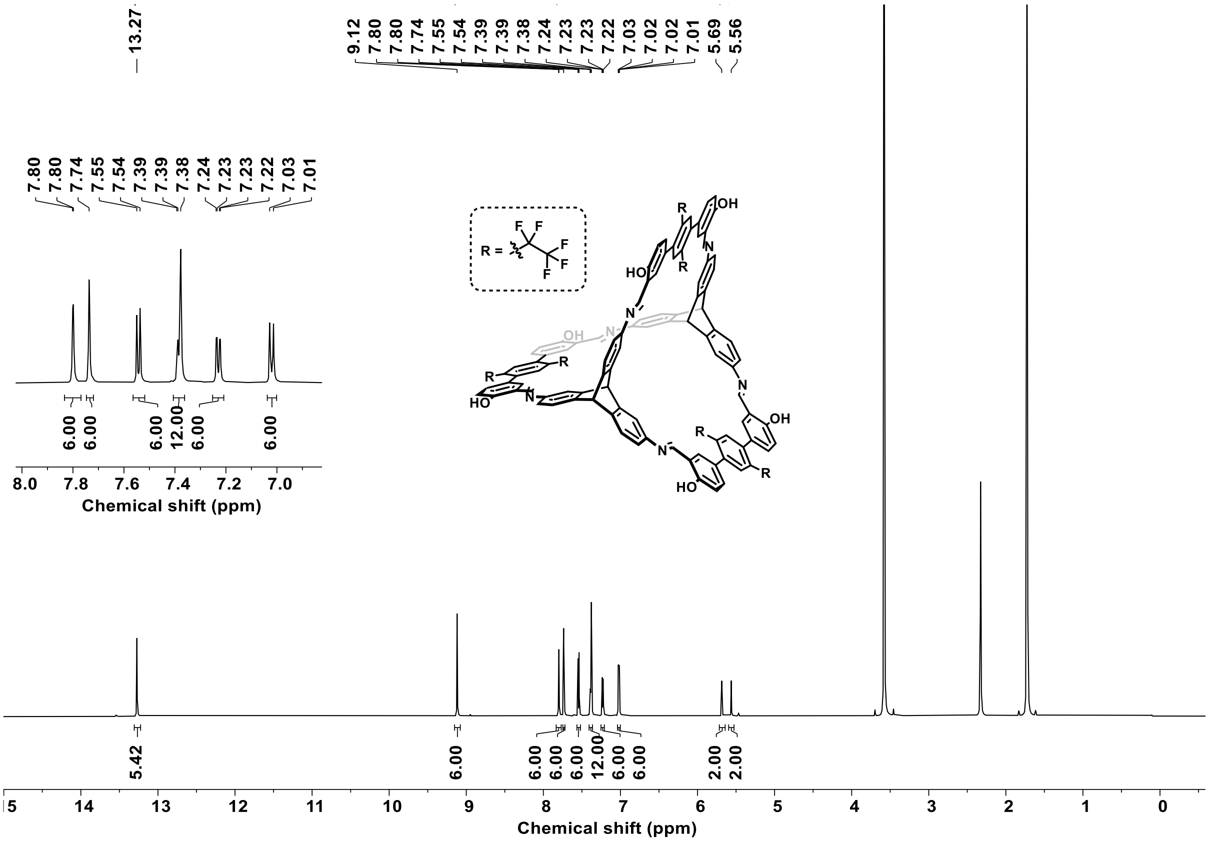

Figure S64. ^{1}H NMR spectrum of **C_2F_5-cage** (THF-d_8, 600 MHz).

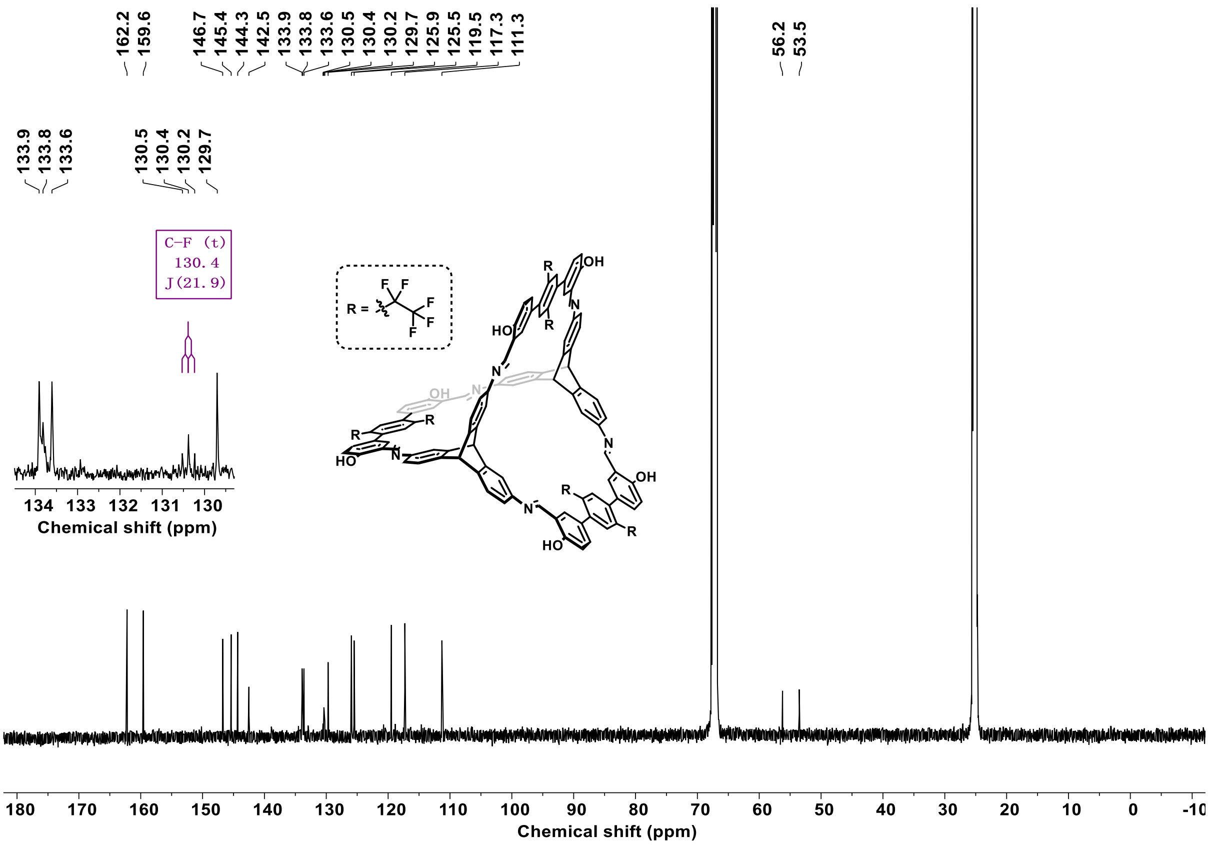

Figure S65. ^{13}C {^1H} NMR spectrum of **C_2F_5-cage** (THF-d_8, 151 MHz).

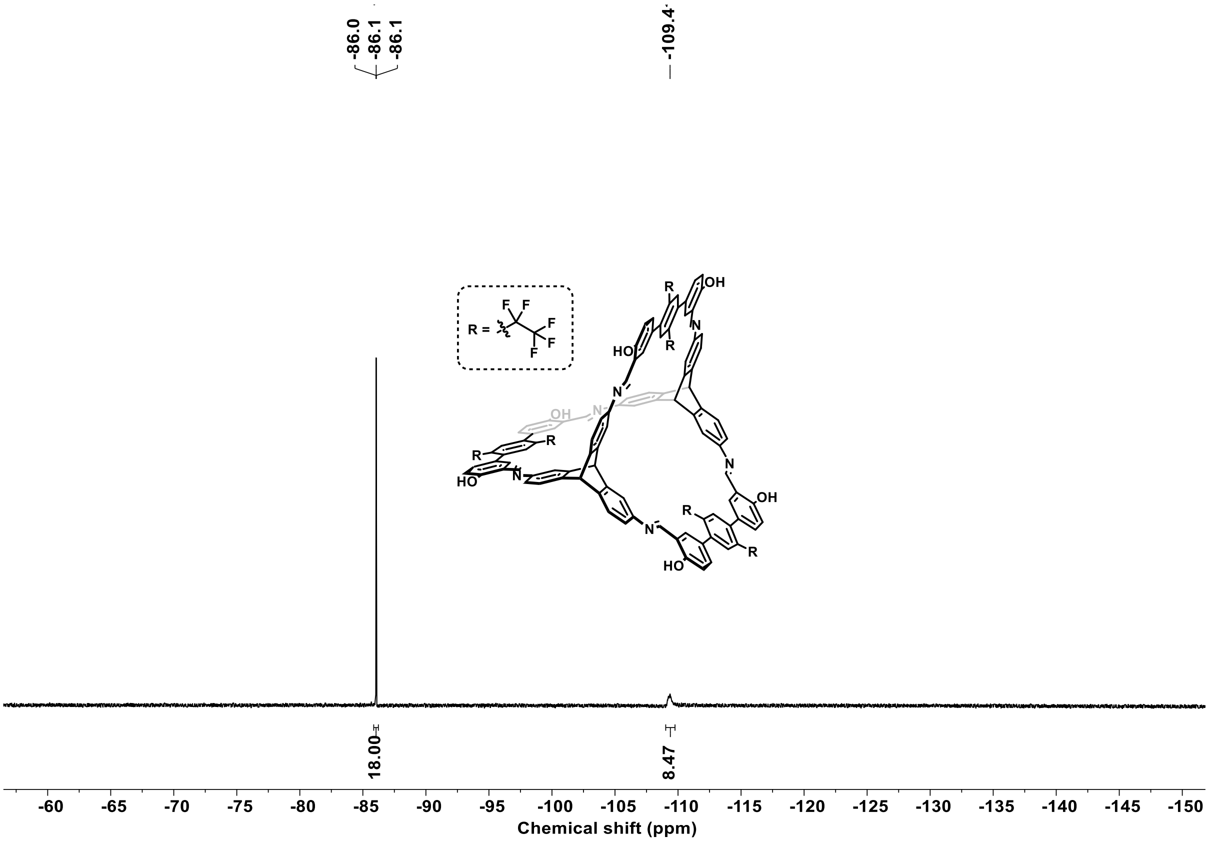

Figure S66. ^{19}F {^1H} NMR spectrum of **C_2F_5-cage** (THF-d_8, 283 MHz).

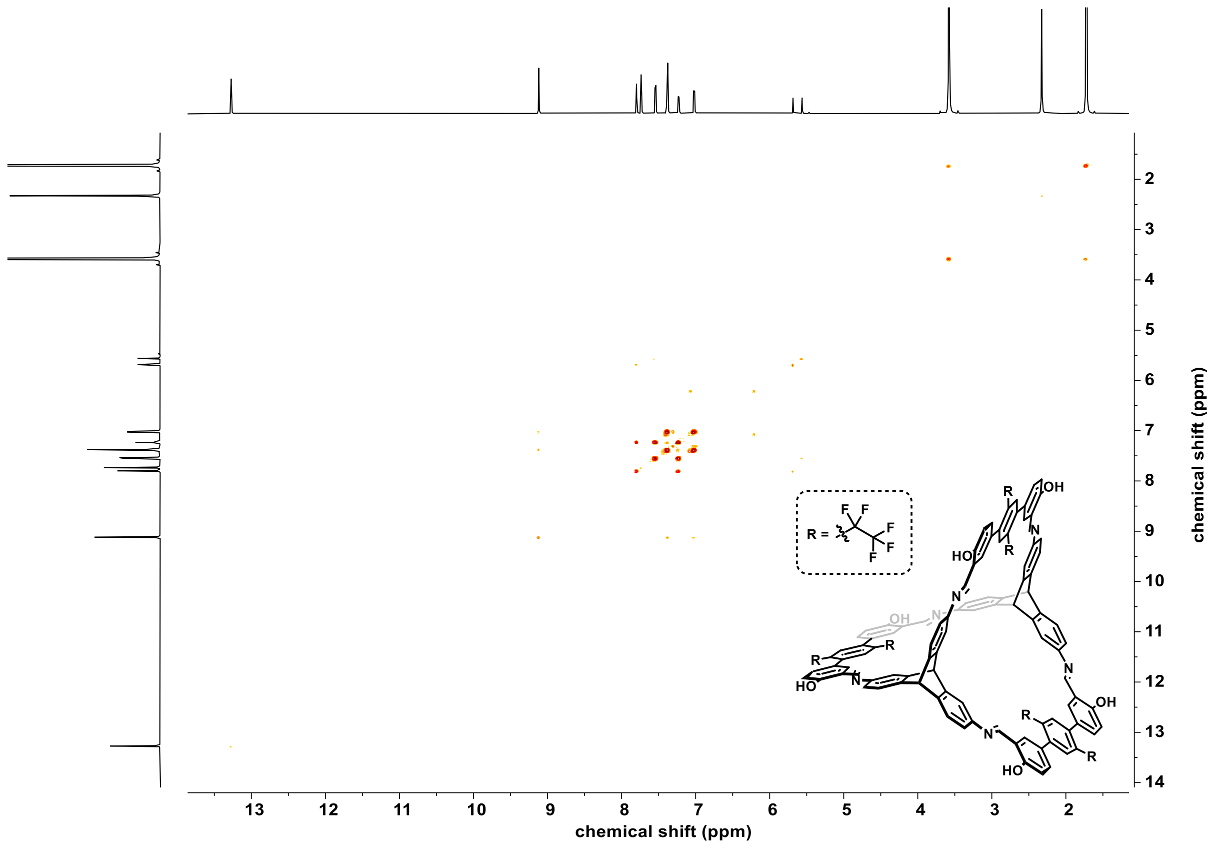

Figure S67. ^1H-^1H COSY NMR spectrum of **C_2F_5-cage** (THF-d_8, 600 MHz, 600 MHz).

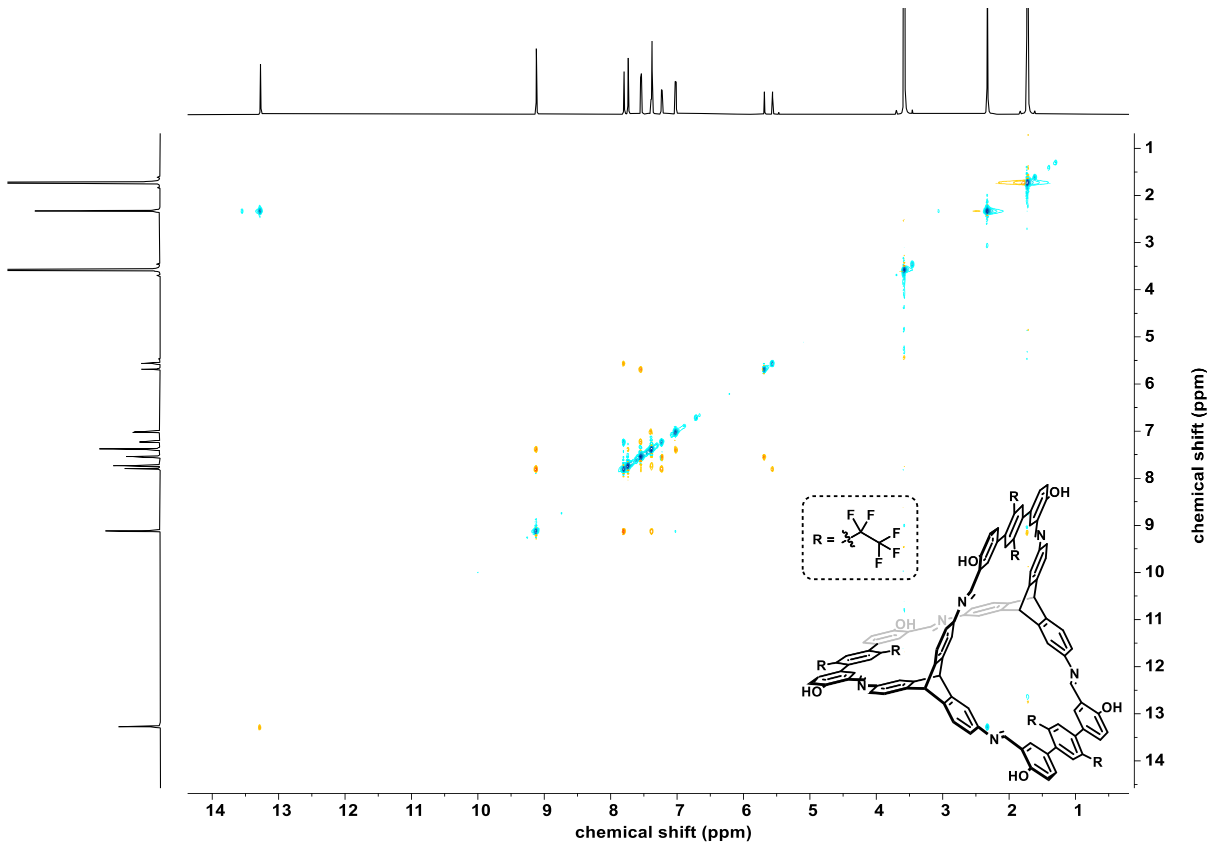

Figure S68. ^1H-^1H ROESY NMR spectrum of **C_2F_5-cage** (THF-d_8, 600 MHz, 600 MHz).

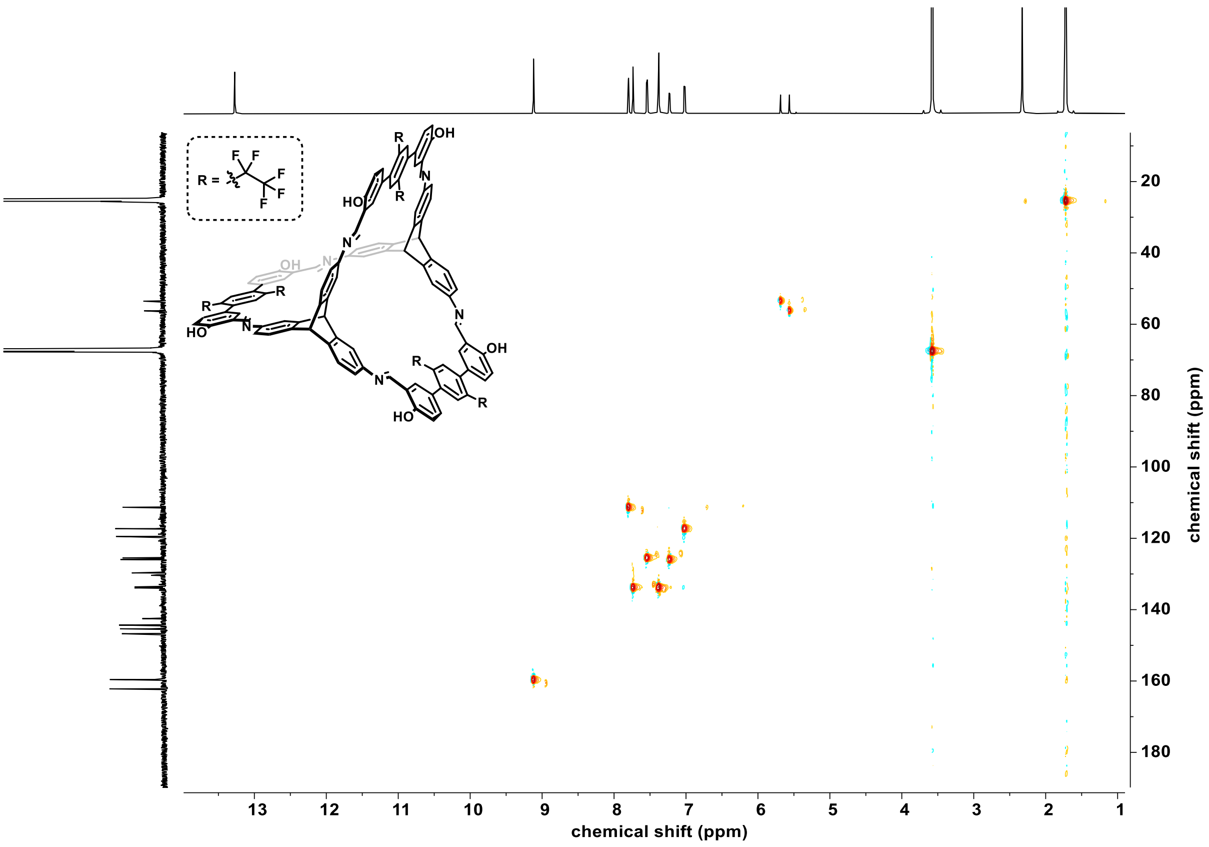

Figure S69. ^{1}H-^{13}C HSQC NMR spectrum of **C_2F_5-cage** (THF-d_8, 600 MHz, 151 MHz).

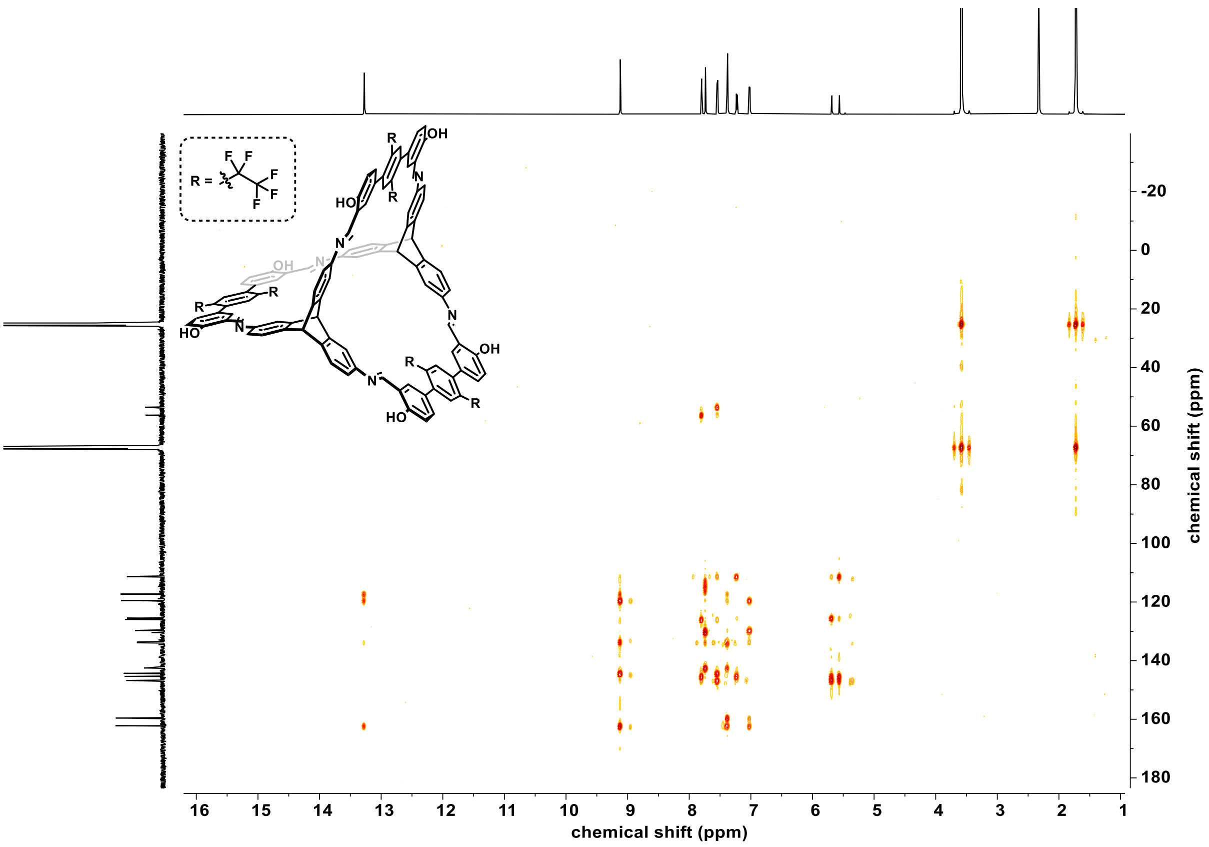

Figure S70. ^{1}H-^{13}C HMBC NMR spectrum of **C_2F_5-cage** (THF-d_8, 600 MHz, 151 MHz).

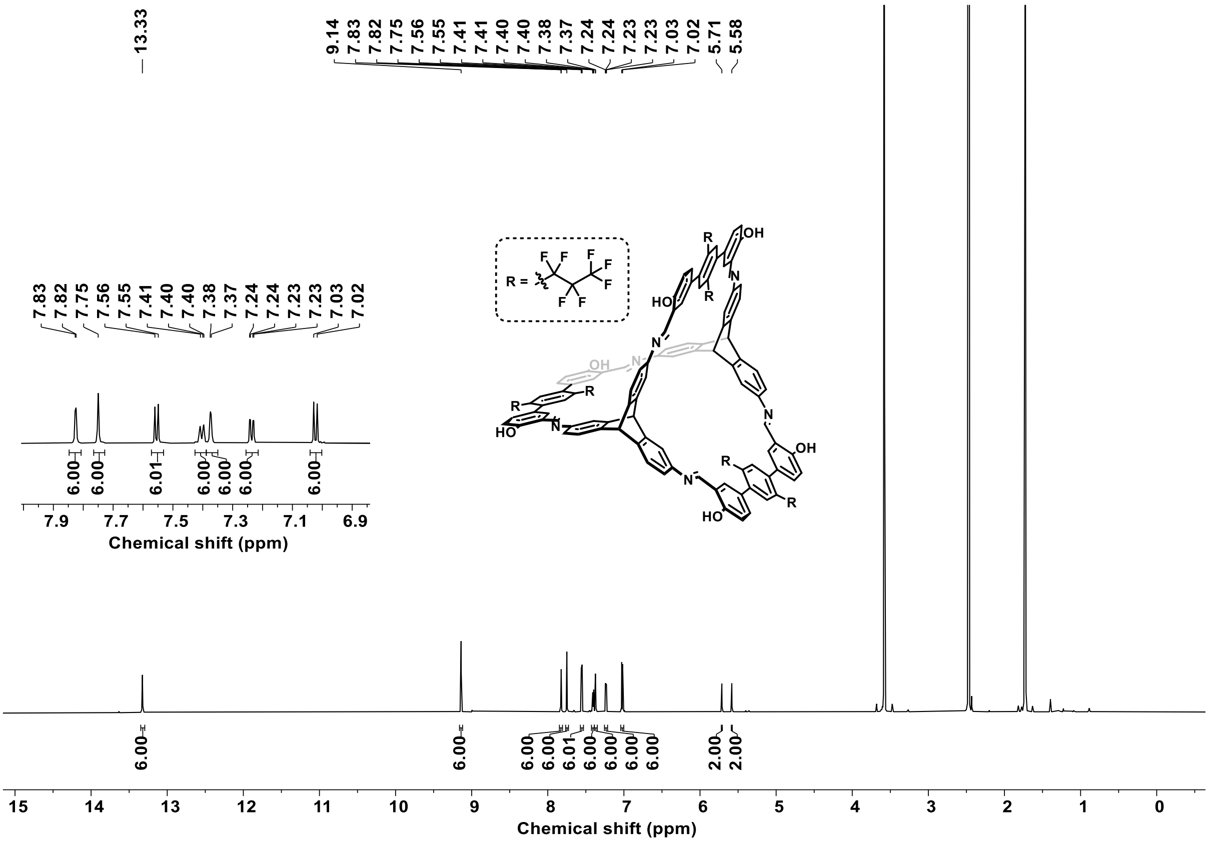

Figure S71. ^{1}H NMR spectrum of **C_3F_7-cage** (THF-d_8, 700 MHz).

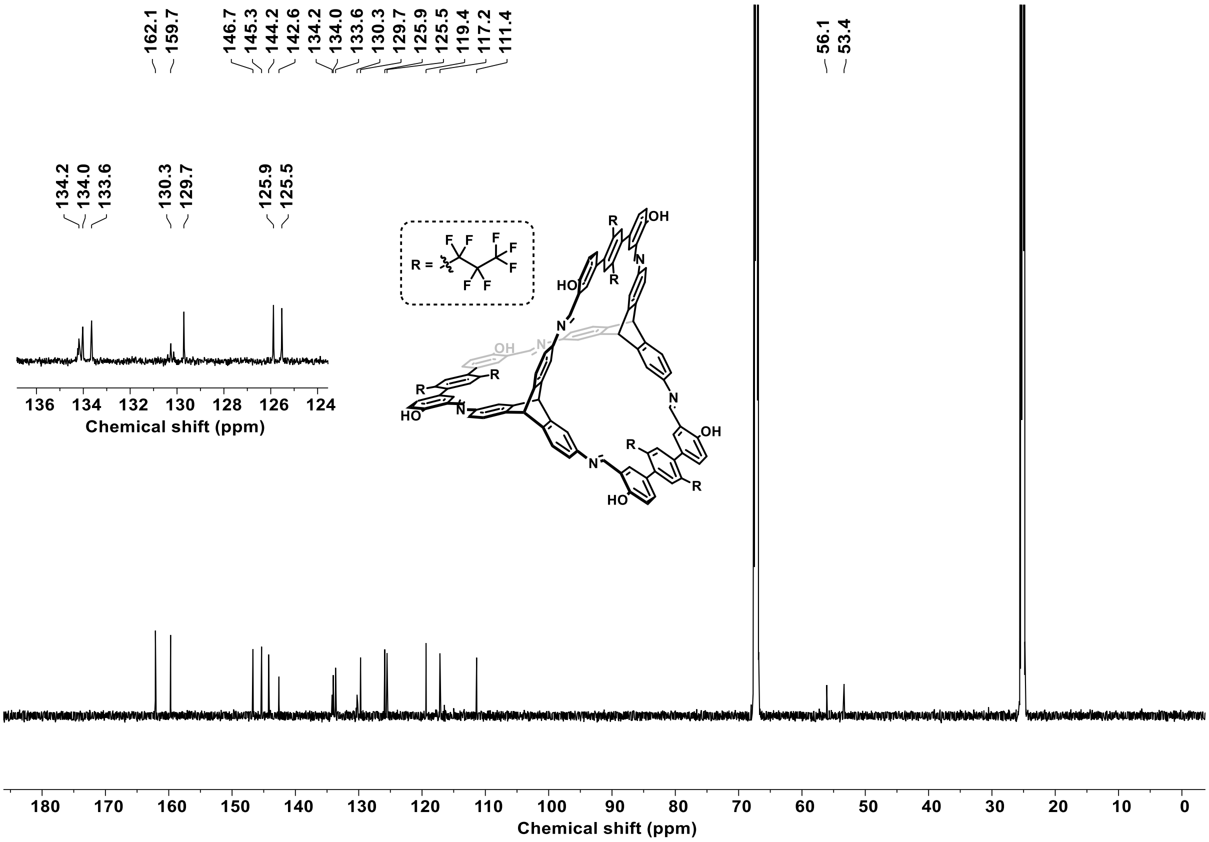

Figure S72. ^{13}C {^{1}H} NMR spectrum of **C_3F_7-cage** (THF-d_8, 176 MHz).

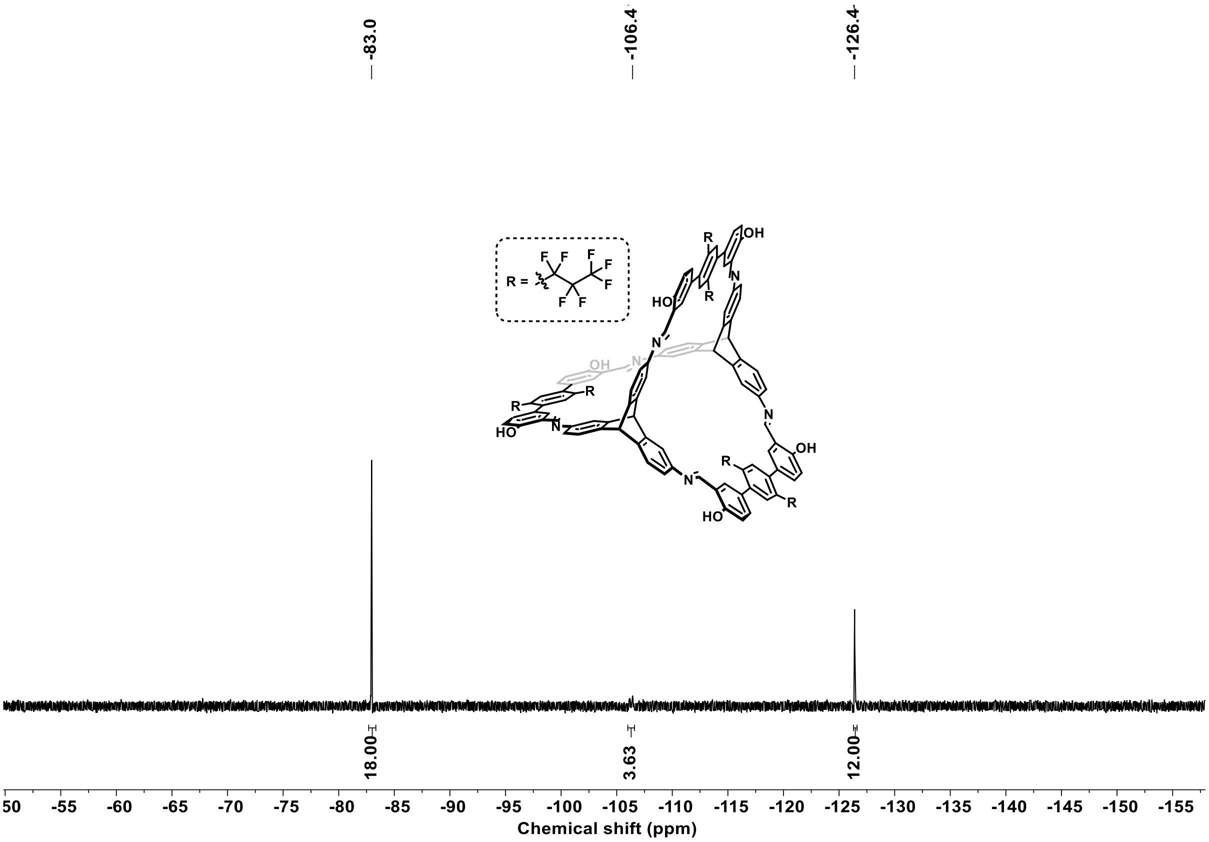

Figure S73. ^{19}F {^{1}H} NMR spectrum of **C_3F_7-cage** (THF-d_8, 283 MHz).

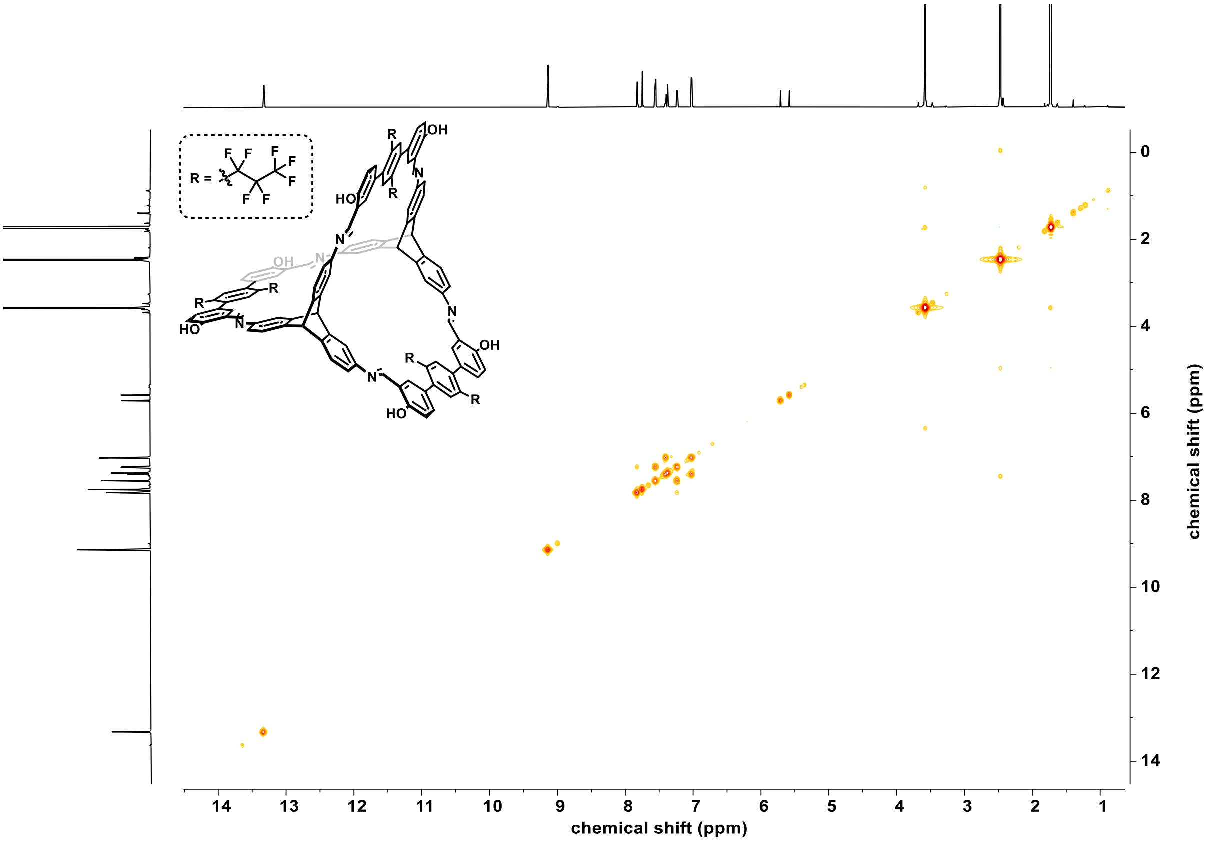

Figure S74. ^{1}H-^{1}H COSY NMR spectrum of **C_3F_7-cage** (THF-d_8, 700 MHz, 700 MHz).

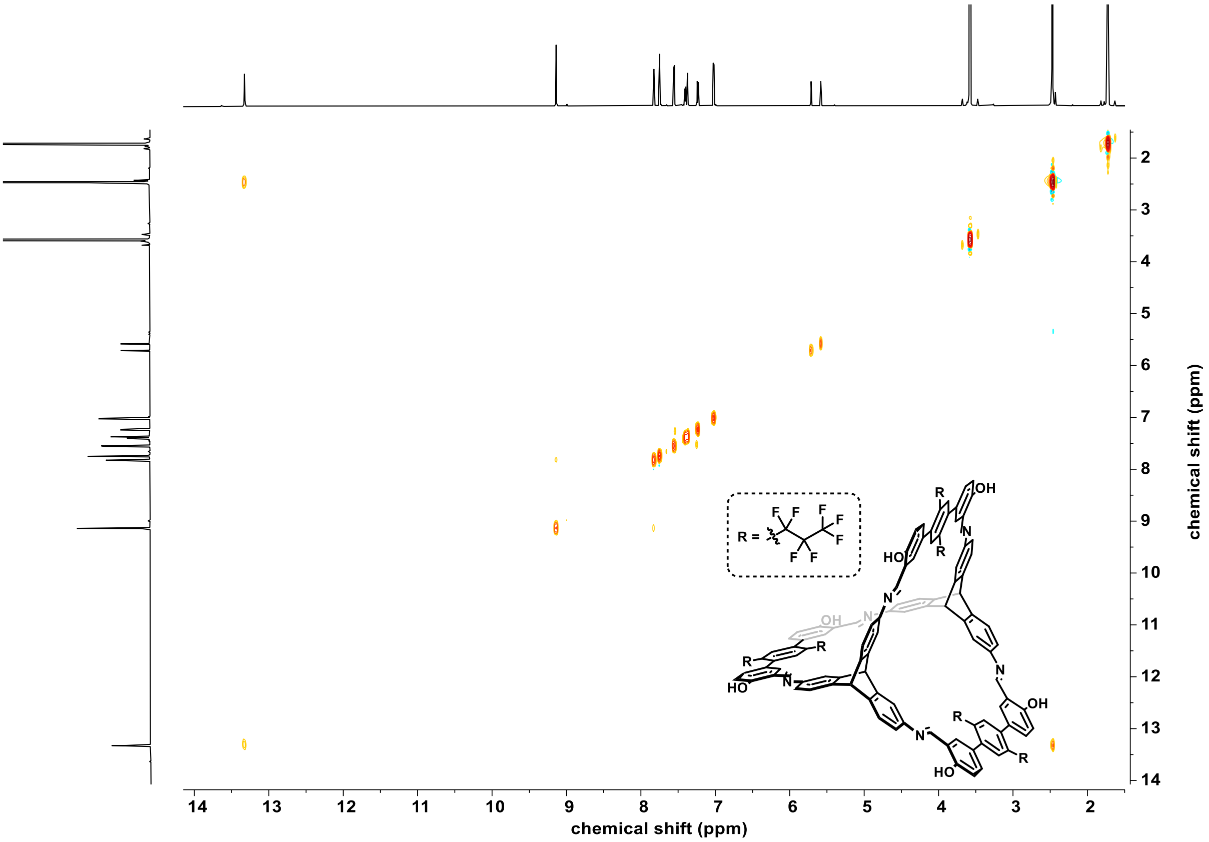

Figure S75. ^{1}H-^{1}H NOESY NMR spectrum of **C_3F_7-cage** (THF-d_8, 700 MHz, 700 MHz).

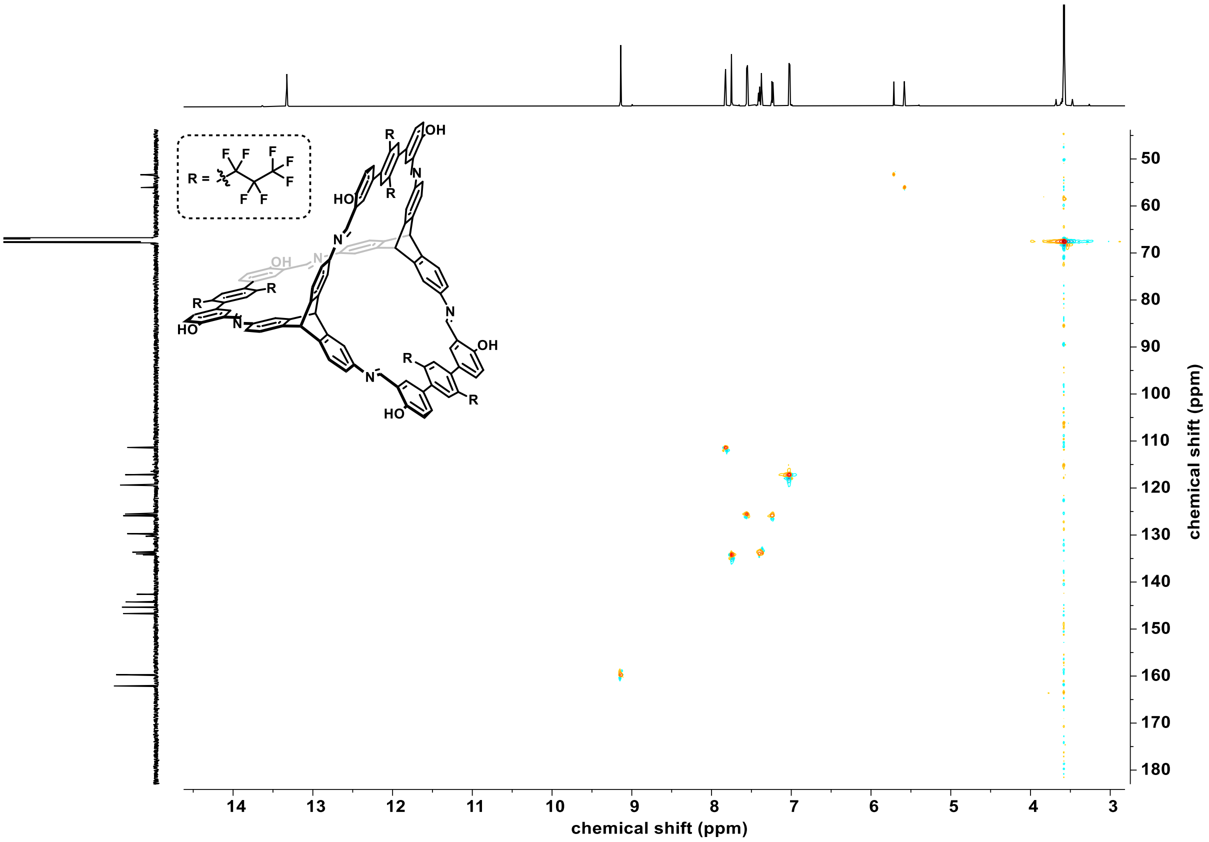

Figure S76. ^{1}H-^{13}C HSQC NMR spectrum of **C_3F_7-cage** (THF-d_8, 700 MHz, 176 MHz).

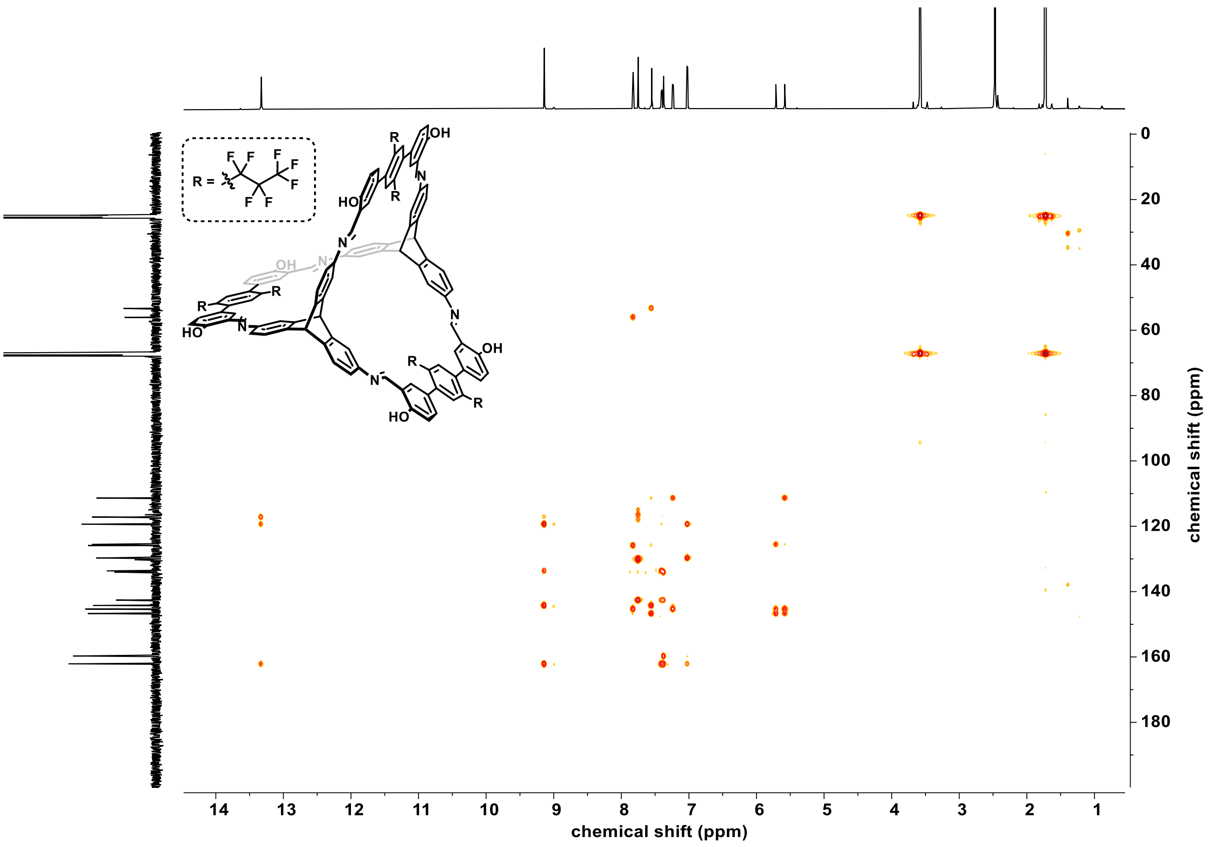

Figure S77. ^{1}H-^{13}C HMBC NMR spectrum of **C_3F_7-cage** (THF-d_8, 700 MHz, 176 MHz).

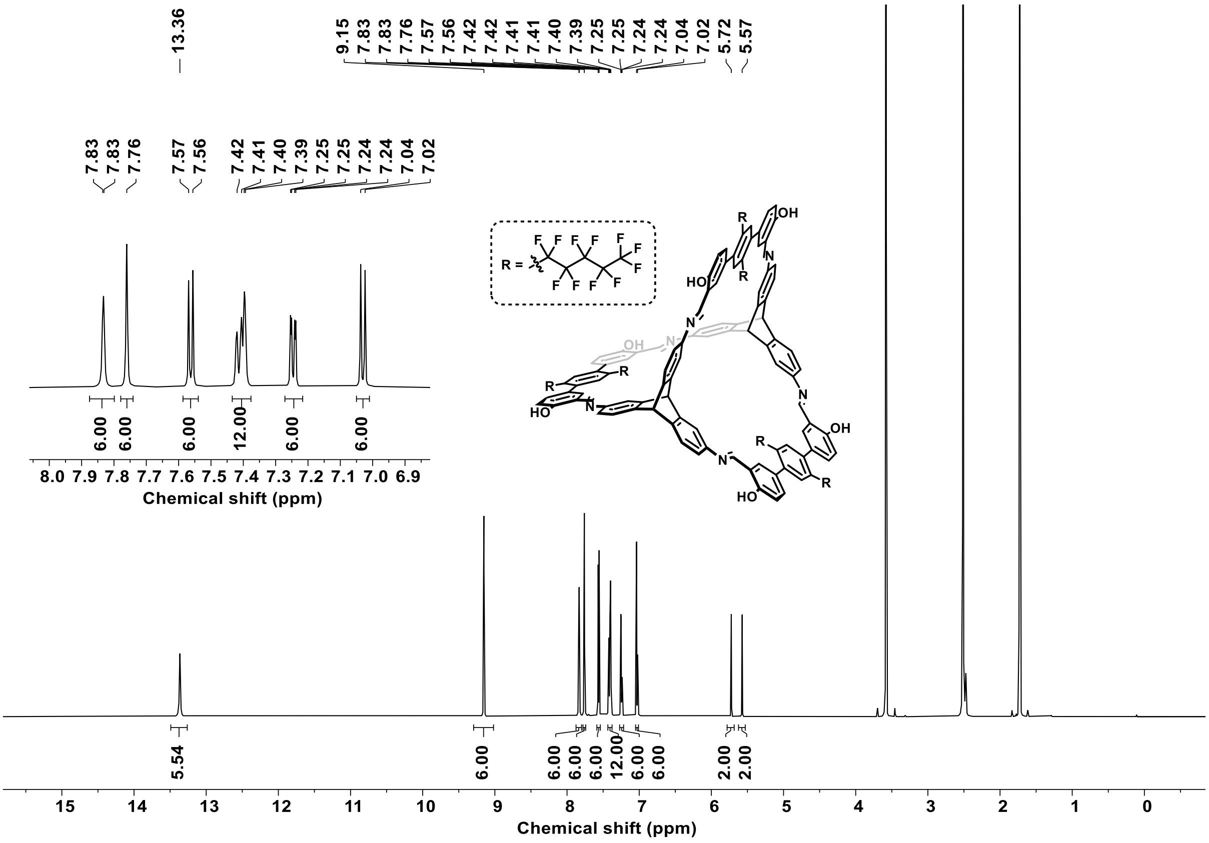

Figure S78. ^{1}H NMR spectrum of **C_5F_{11}-cage** (THF-d_8, 600 MHz).

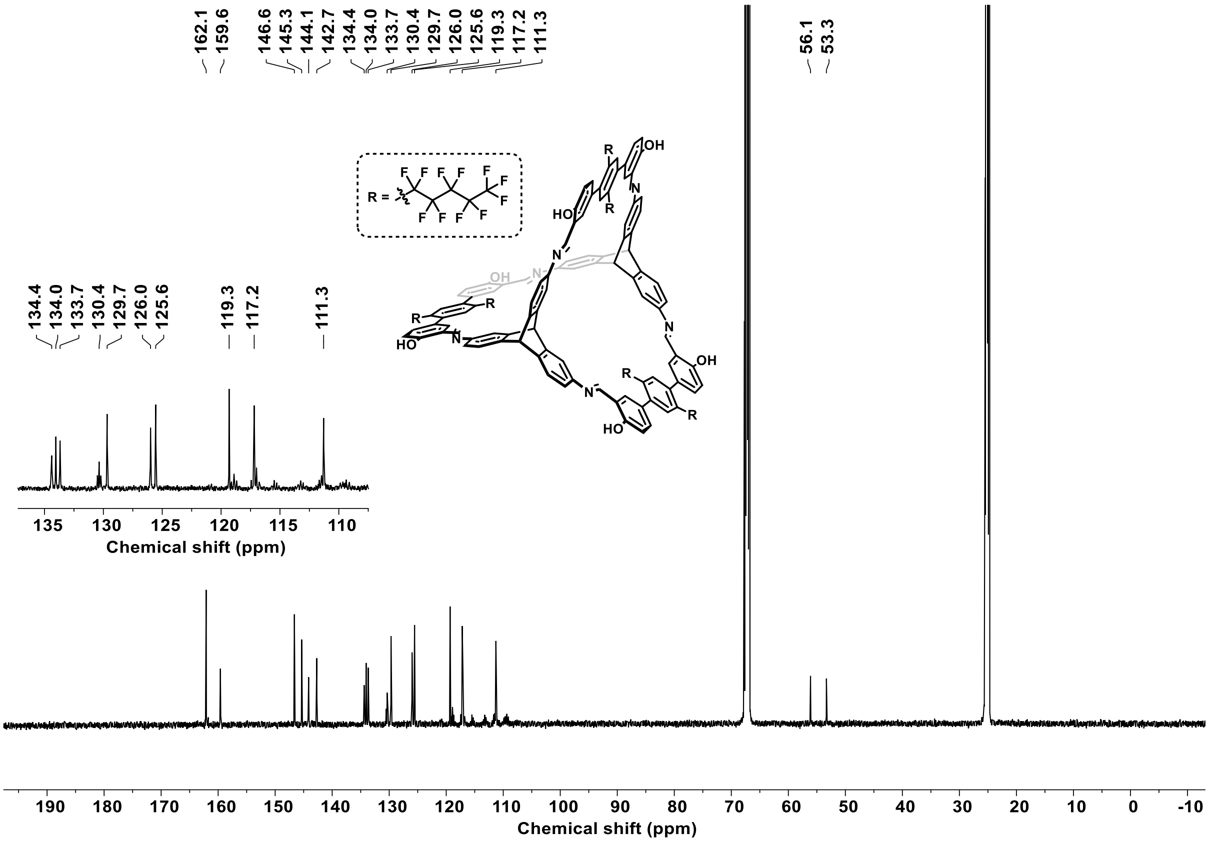

Figure S79. $^{13}C\ \{^{1}H\}$ NMR spectrum of **C_5F_{11}-cage** (THF-d_8, 151 MHz).

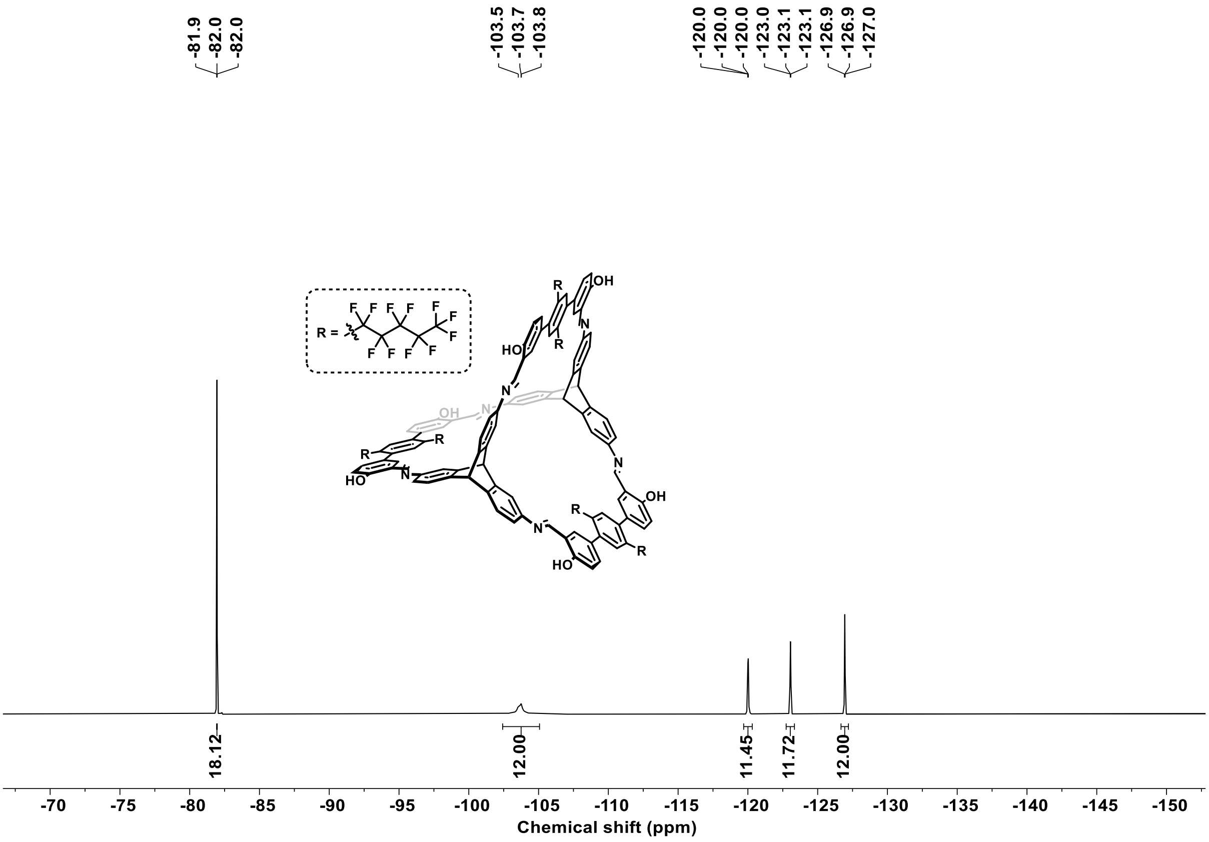

Figure S80. ^{19}F NMR spectrum of **C_5F_{11}-cage** (THF-d_8, 659 MHz).

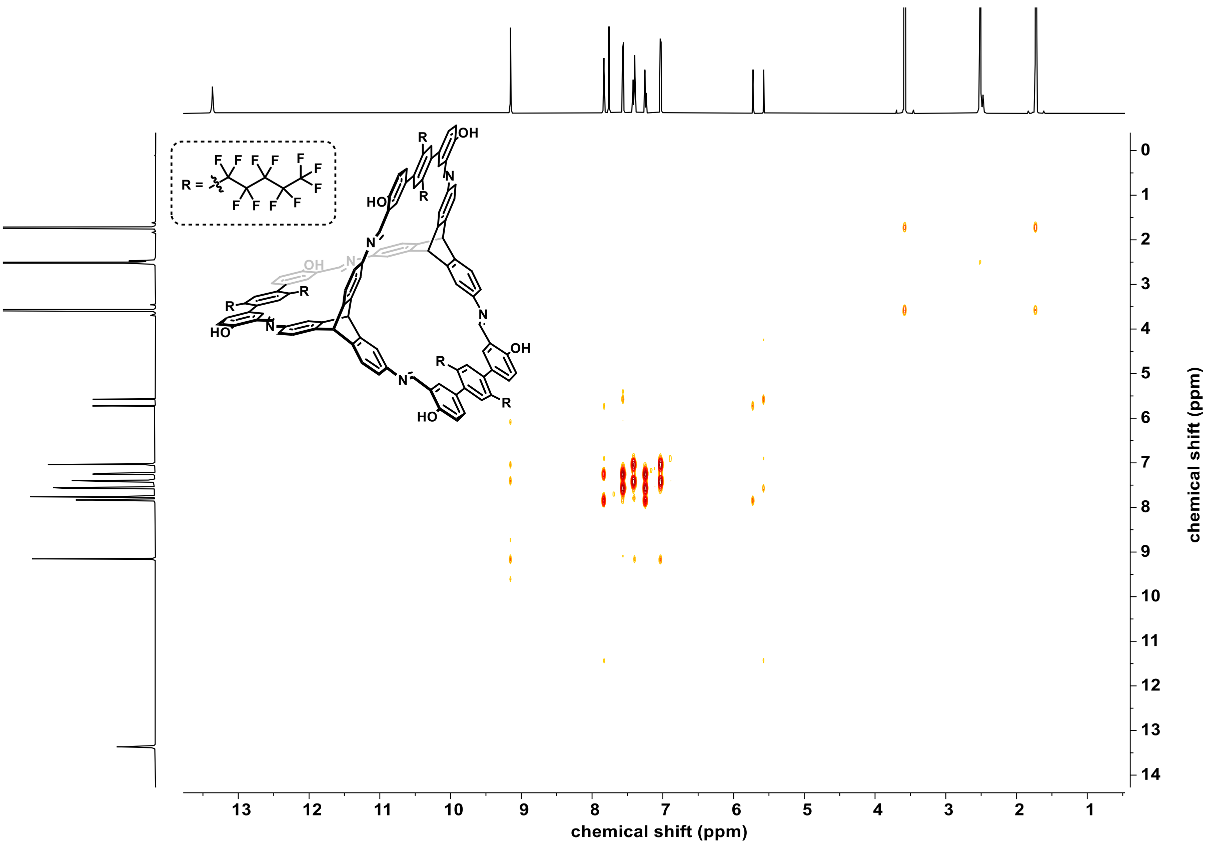

Figure S81. ^{1}H-^{1}H COSY NMR spectrum of **C_5F_{11}-cage** (THF-d_8, 600 MHz, 600 MHz).

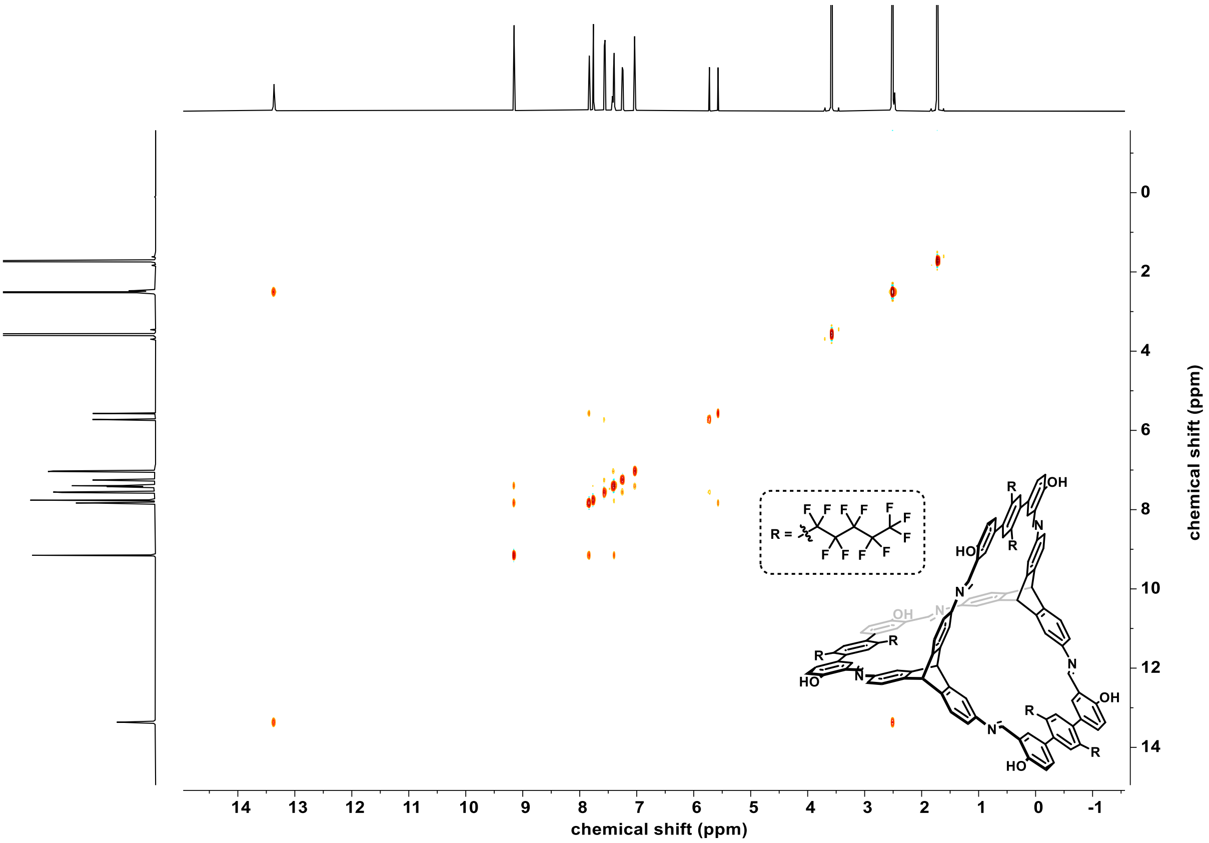

Figure S82. ^{1}H-^{1}H NOESY NMR spectrum of **C_5F_{11}-cage** (THF-d_8, 600 MHz, 600 MHz).

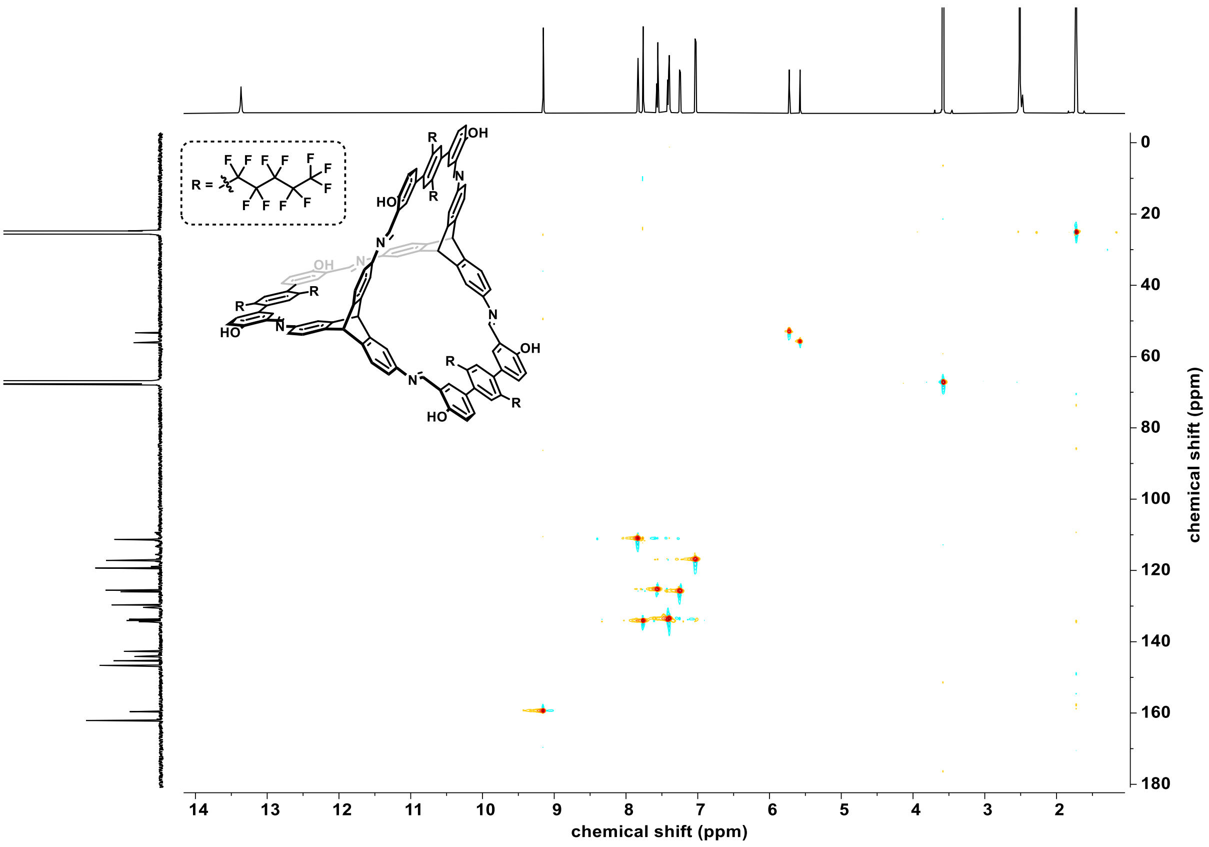

Figure S83. ^{1}H-^{13}C HSQC NMR spectrum of **C_5F_{11}-cage** (THF-d_8, 600 MHz, 151 MHz).

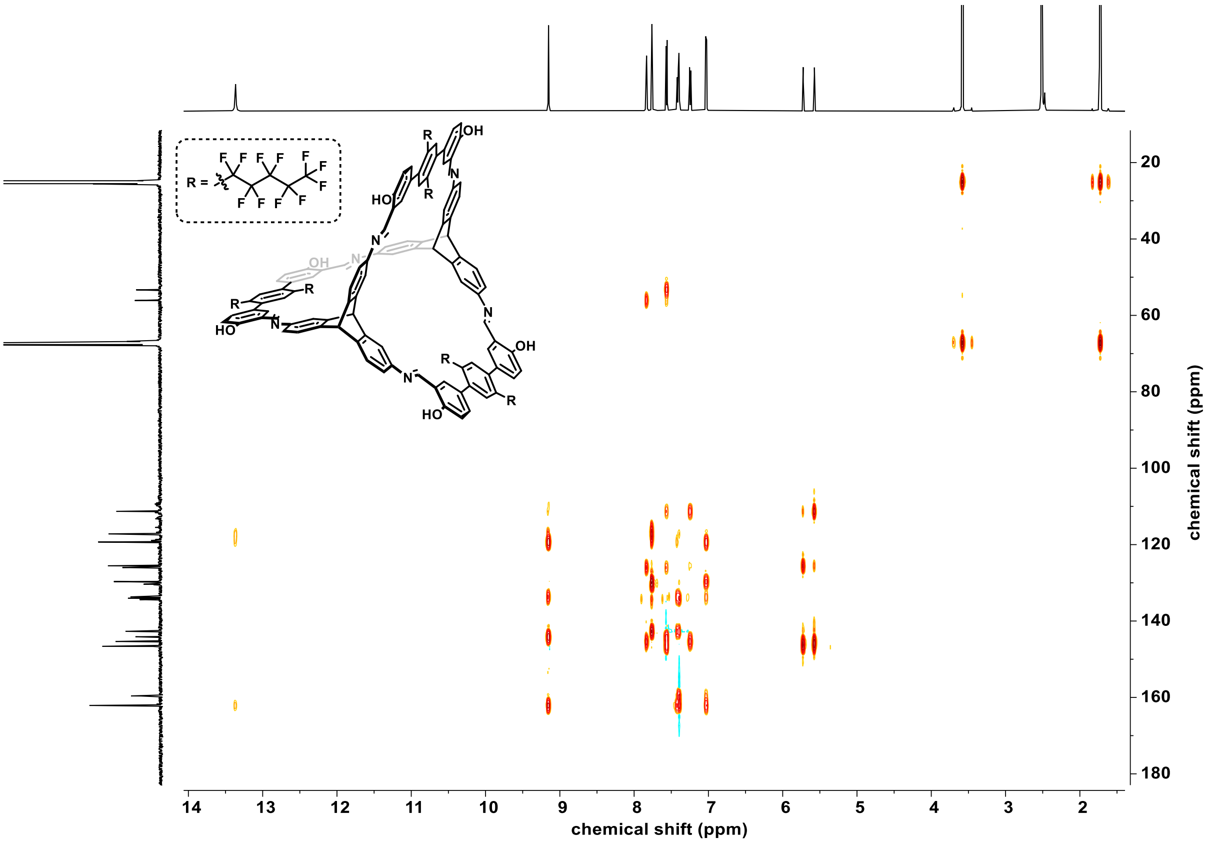

Figure S84. ^{1}H-^{13}C HMBC NMR spectrum of **C_5F_{11}-cage** (THF-d_8, 600 MHz, 151 MHz).

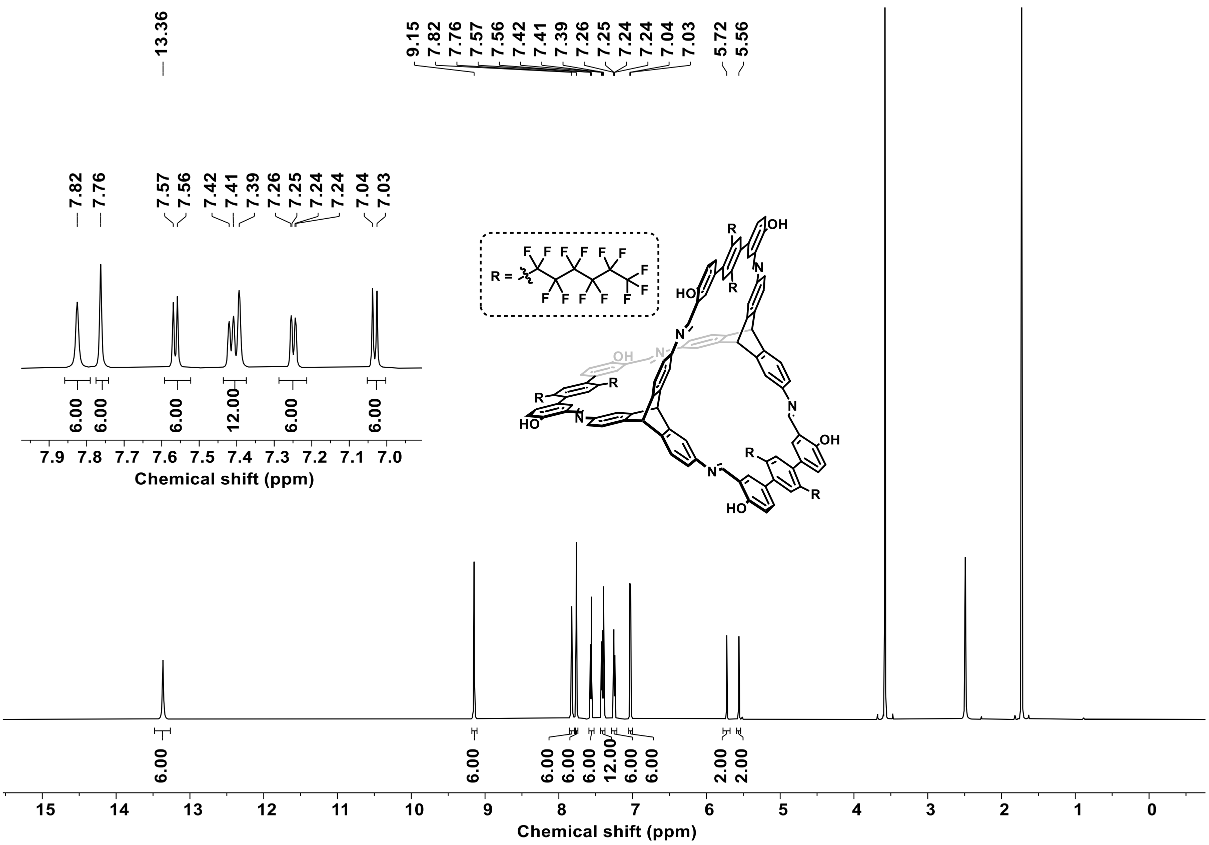

Figure S85. ^{1}H NMR spectrum of **C_6F_{13}-cage** (THF-d_8, 700 MHz).

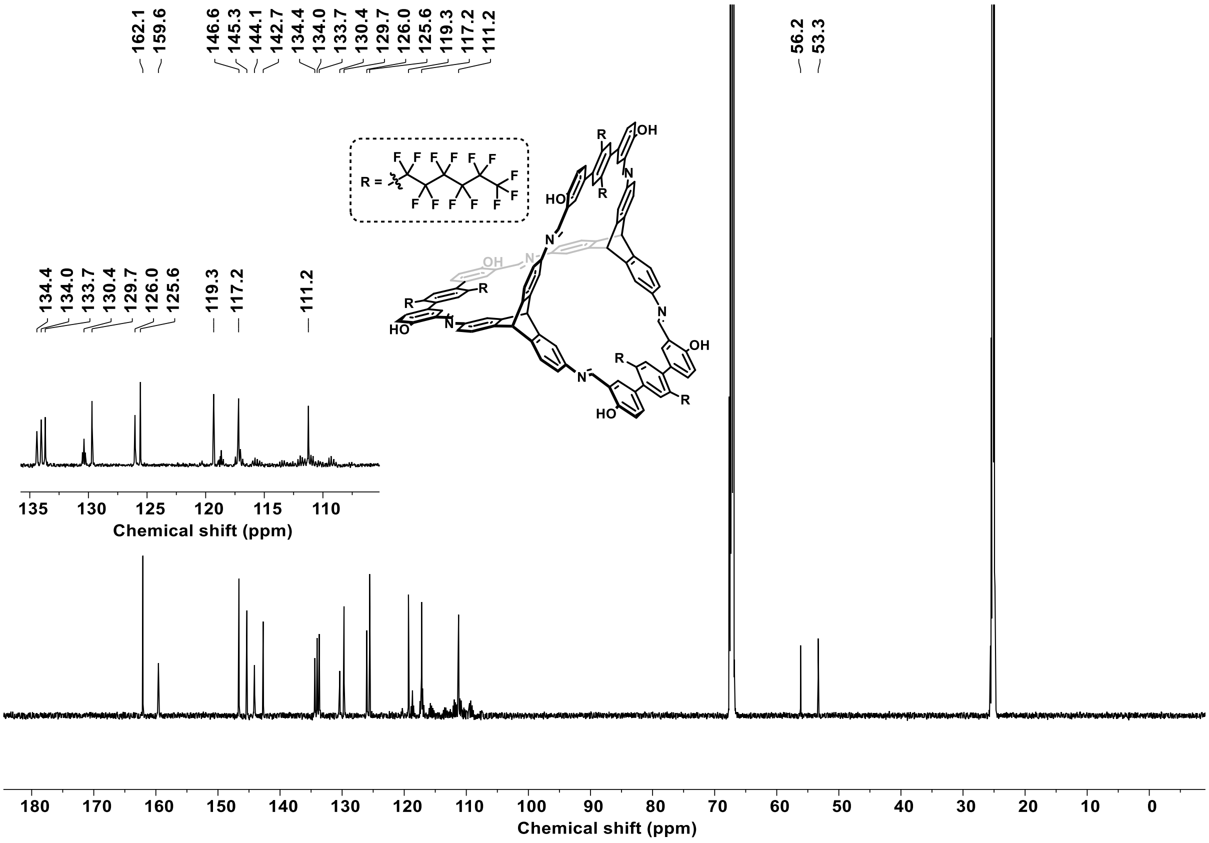

Figure S86. ^{13}C {^{1}H} NMR spectrum of **C_6F_{13}-cage** (THF-d_8, 176 MHz).

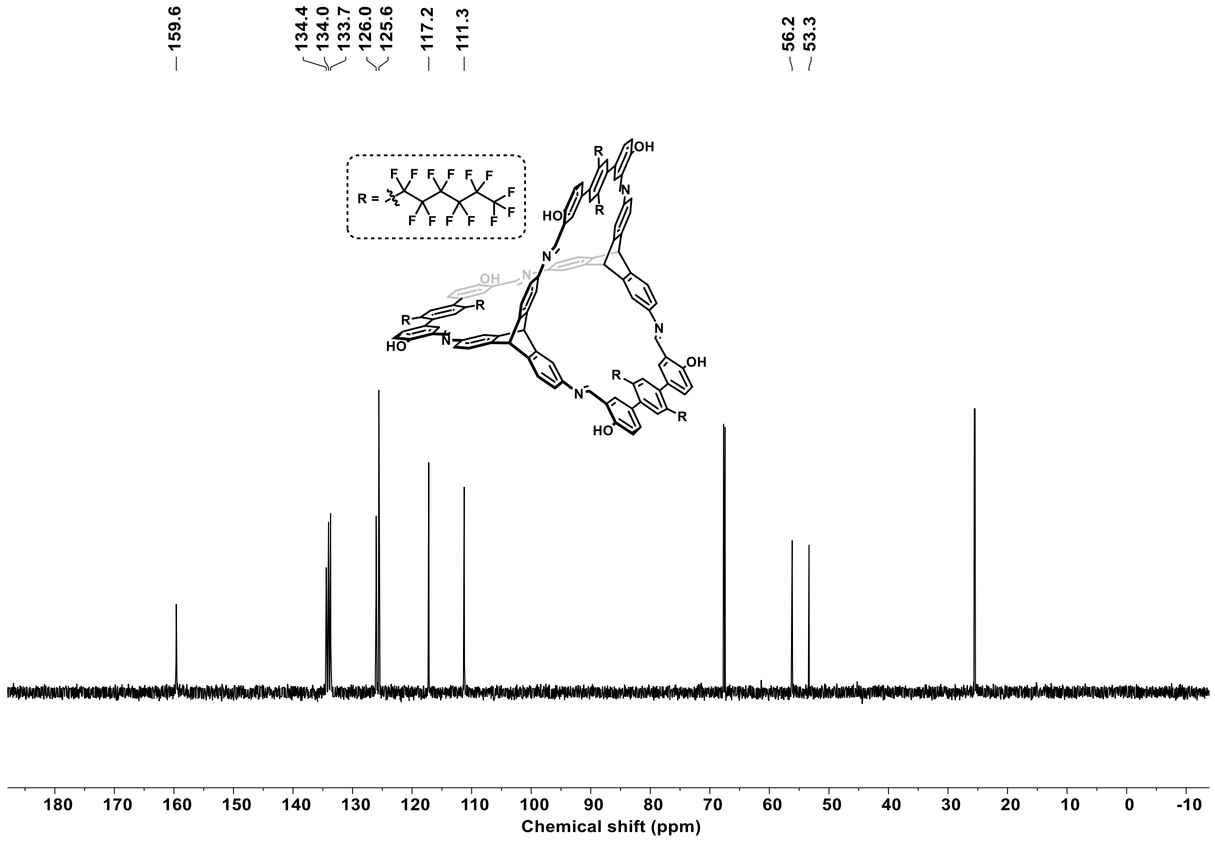

Figure S87. ^{13}C-DEPT135 NMR spectrum of **C_6F_{13}-cage** (THF-d_8, 176 MHz).

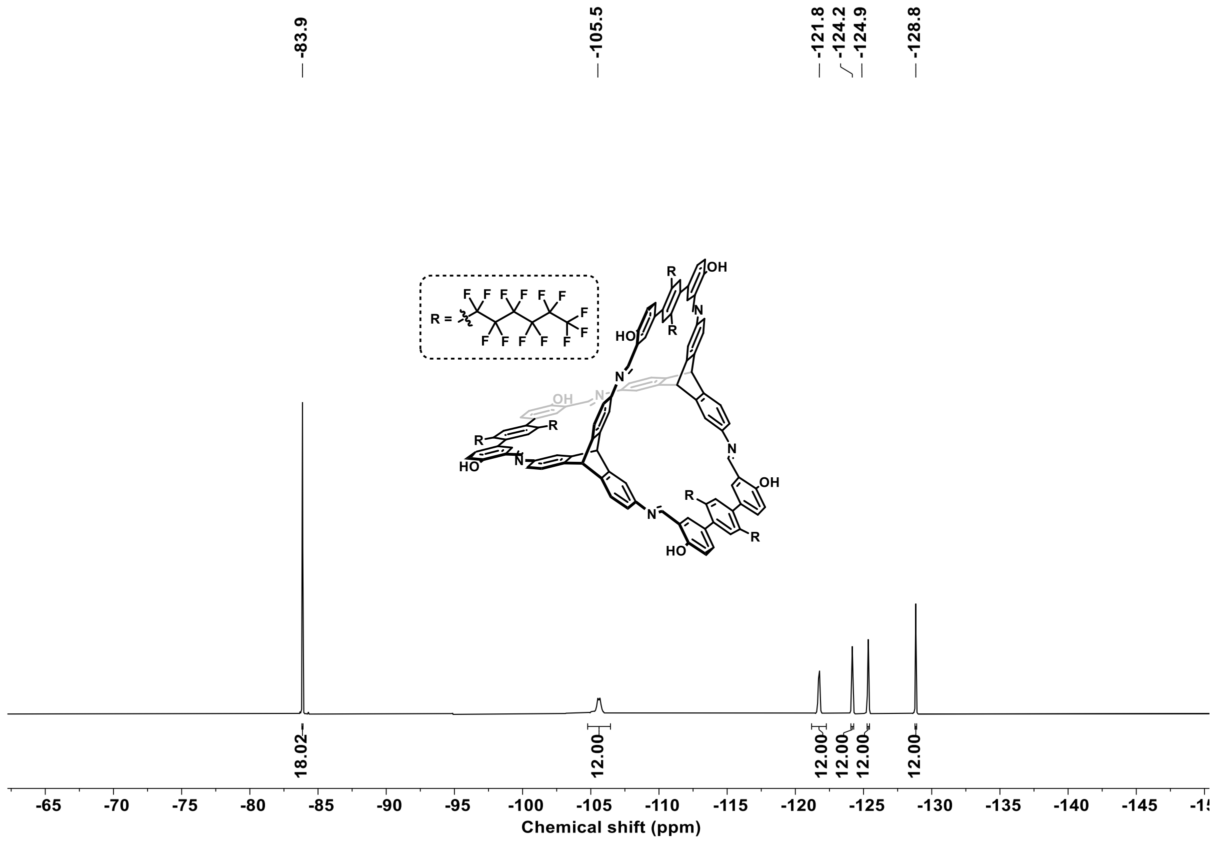

Figure S88. ^{19}F {^1H} NMR spectrum of **C_6F_{13}-cage** (THF-d_8, 471 MHz).

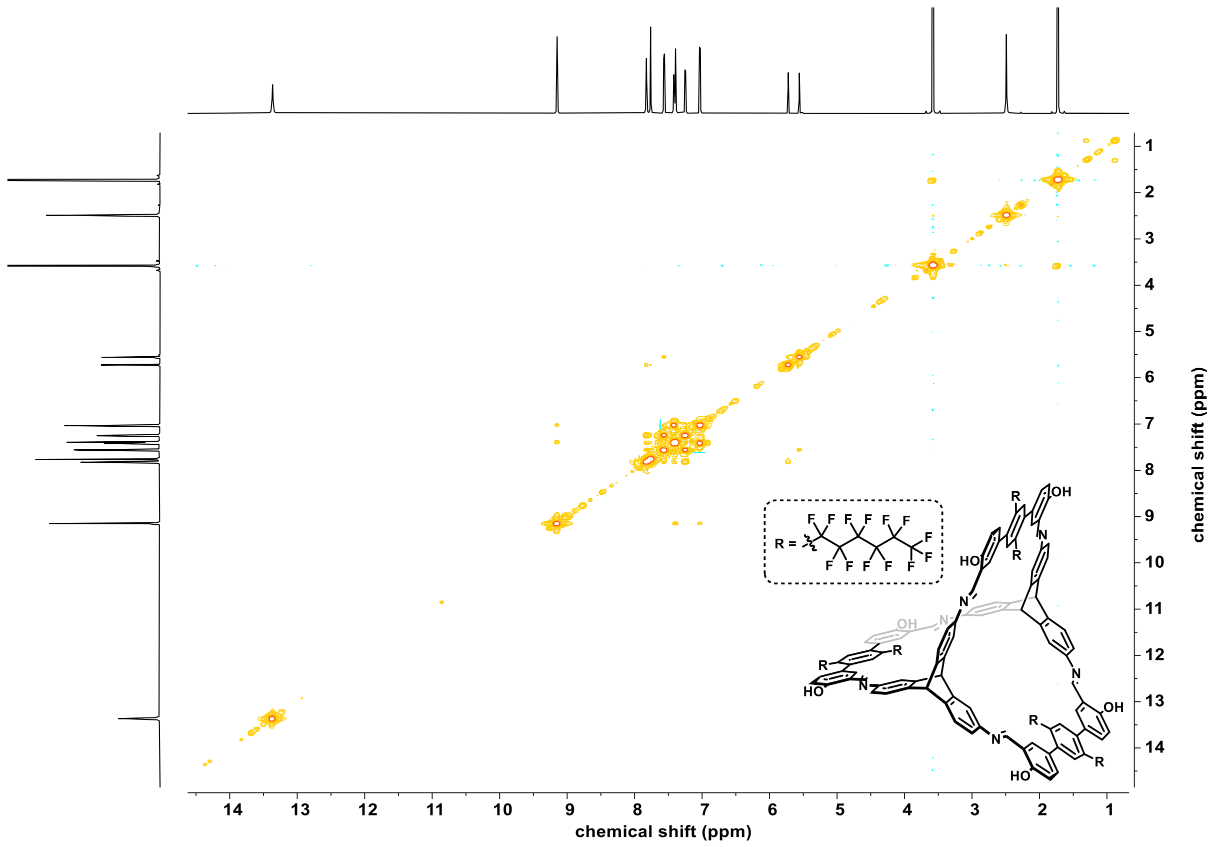

Figure S89. ^{1}H-^{1}H COSY NMR spectrum of **C_6F_{13}-cage** (THF-d_8, 700 MHz, 700 MHz).

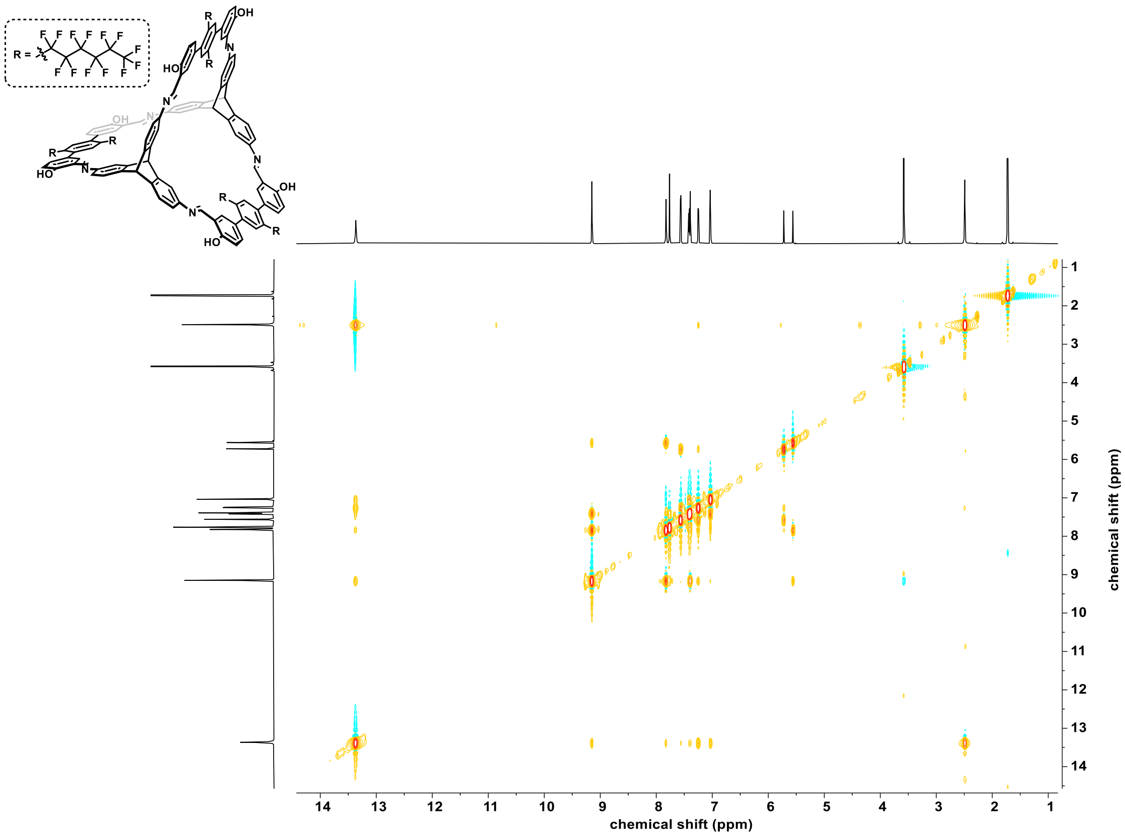

Figure S90. ^{1}H-^{1}H NOESY NMR spectrum of **C_6F_{13}-cage** (THF-d_8, 700 MHz, 700 MHz).

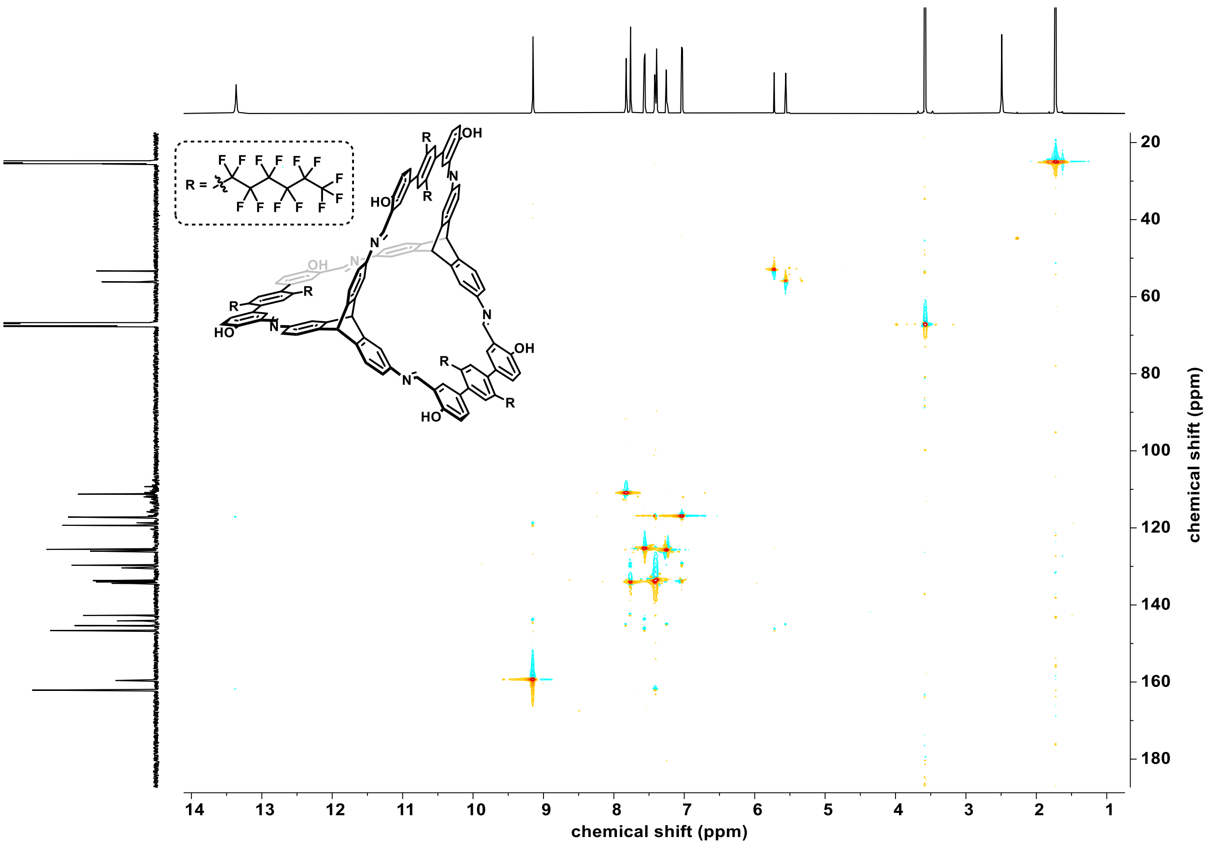

Figure S91. ^{1}H-^{13}C HSQC NMR spectrum of **C_6F_{13}-cage** (THF-d_8, 700 MHz, 176 MHz).

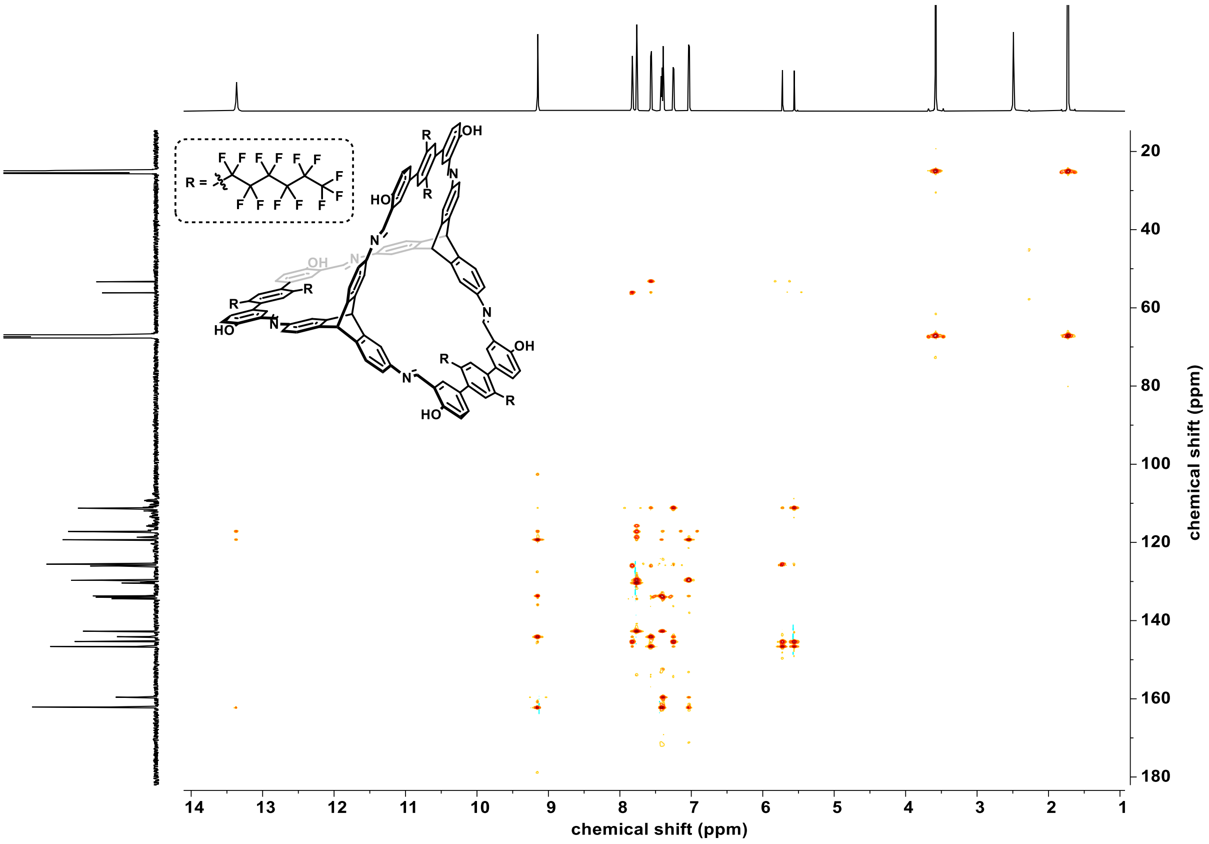

Figure S92. ^{1}H-^{13}C HMBC NMR spectrum of **C_6F_{13}-cage** (THF-d_8, 700 MHz, 176 MHz).

DOSY-NMR-Spectroscopy

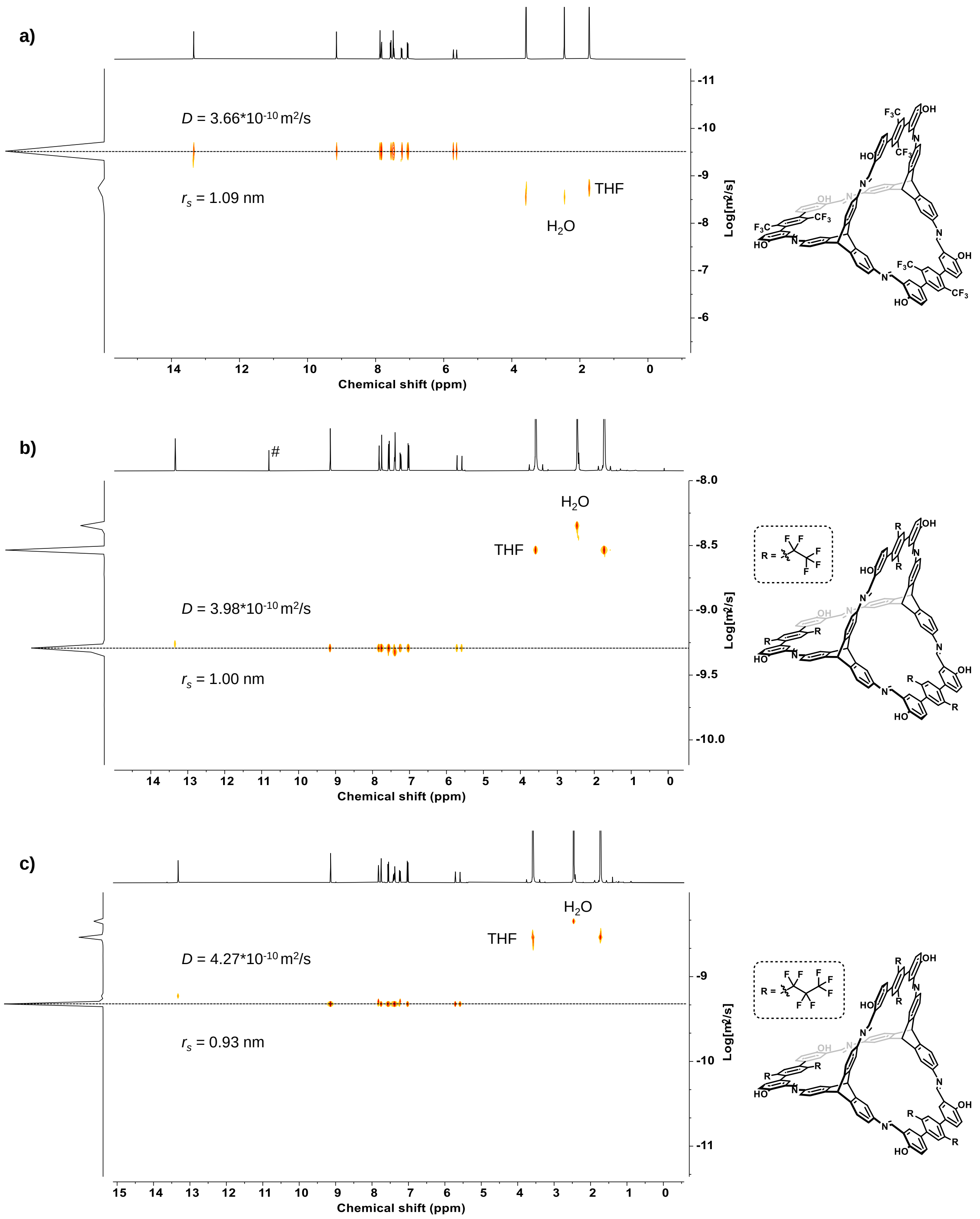

Figure S93. DOSY-NMR spectra (THF-d_8, 400 MHz, 295 K) of **CF3-cage** (a), **C2F5-cage** (b), and **C3F7-cage** (c).

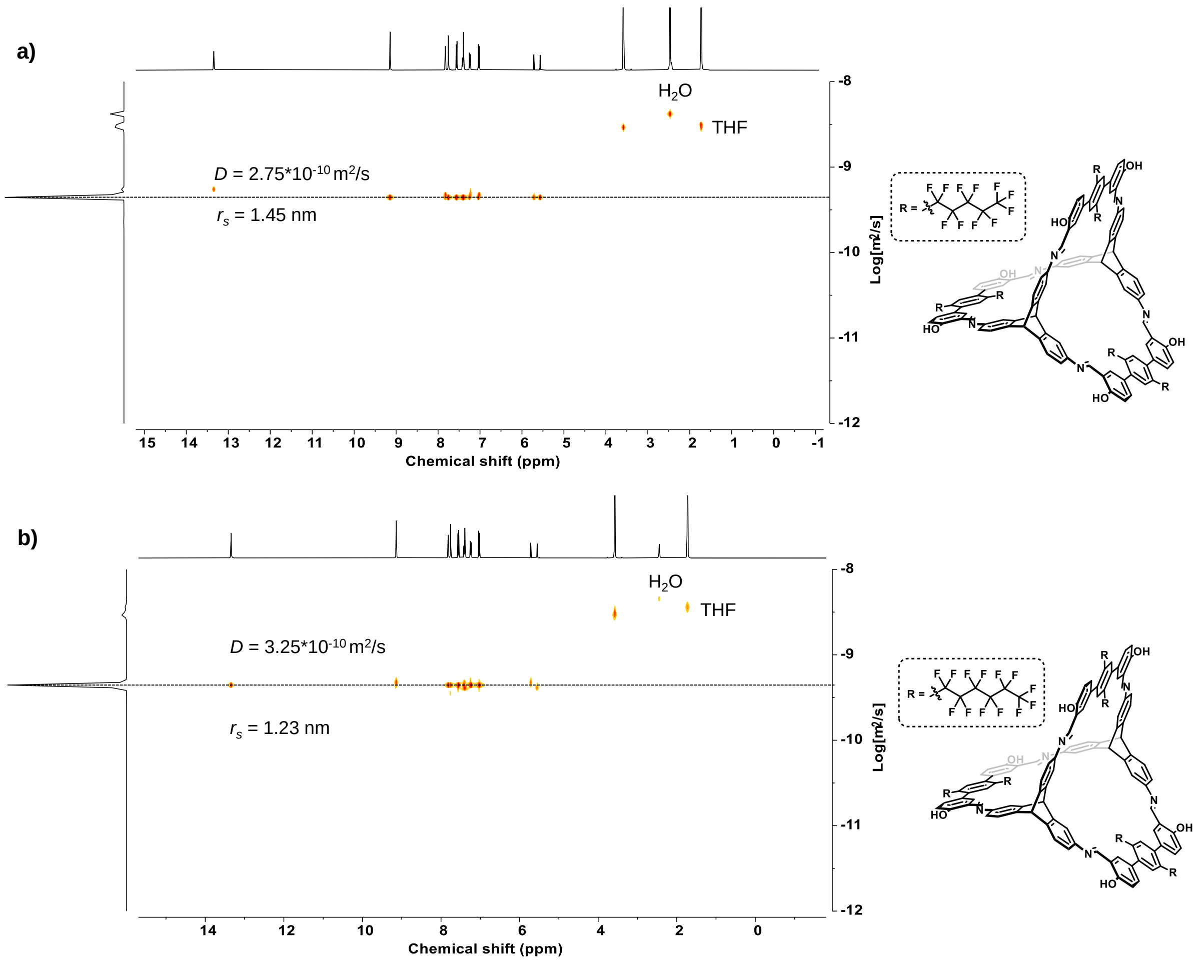

Figure S94. DOSY-NMR spectra (THF-*d*8, 400 MHz, 295 K) of **C_5F_{11}-cage-** (a) and **C_6F_{13}-cage** (b).

4. Mass Spectrometry

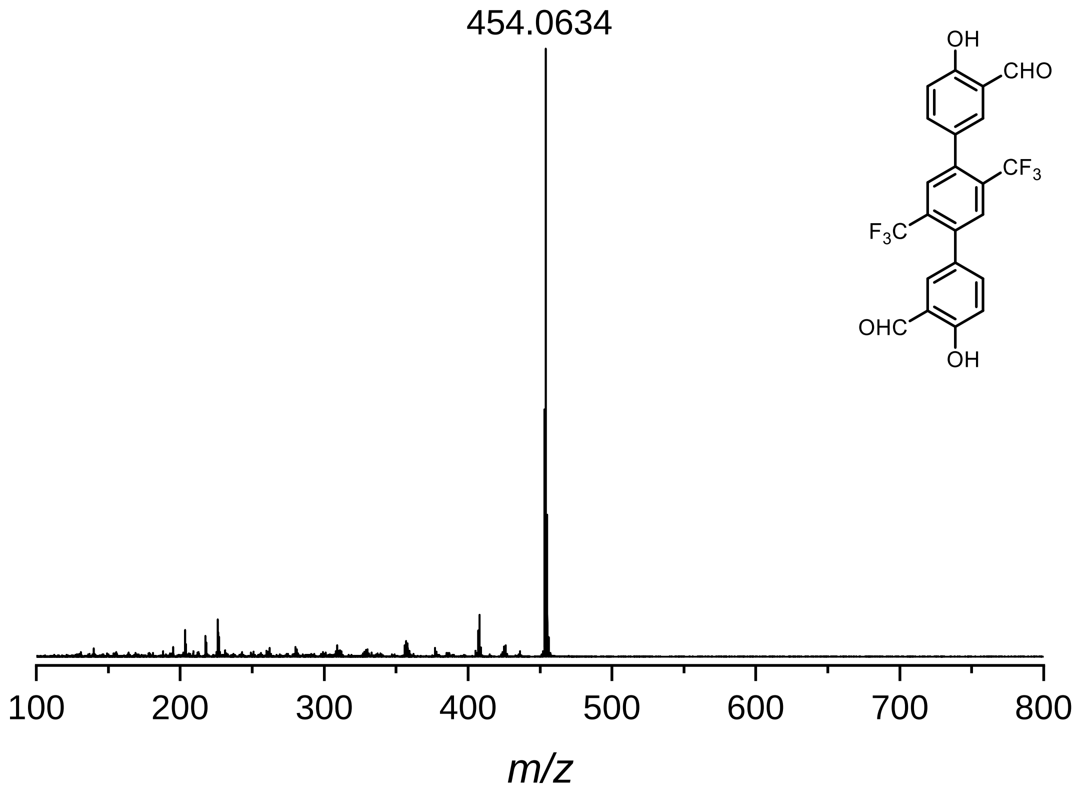

Figure S95. Mass spectrum (EI^+) of CF_3-salicylaldehyde (**2a**).

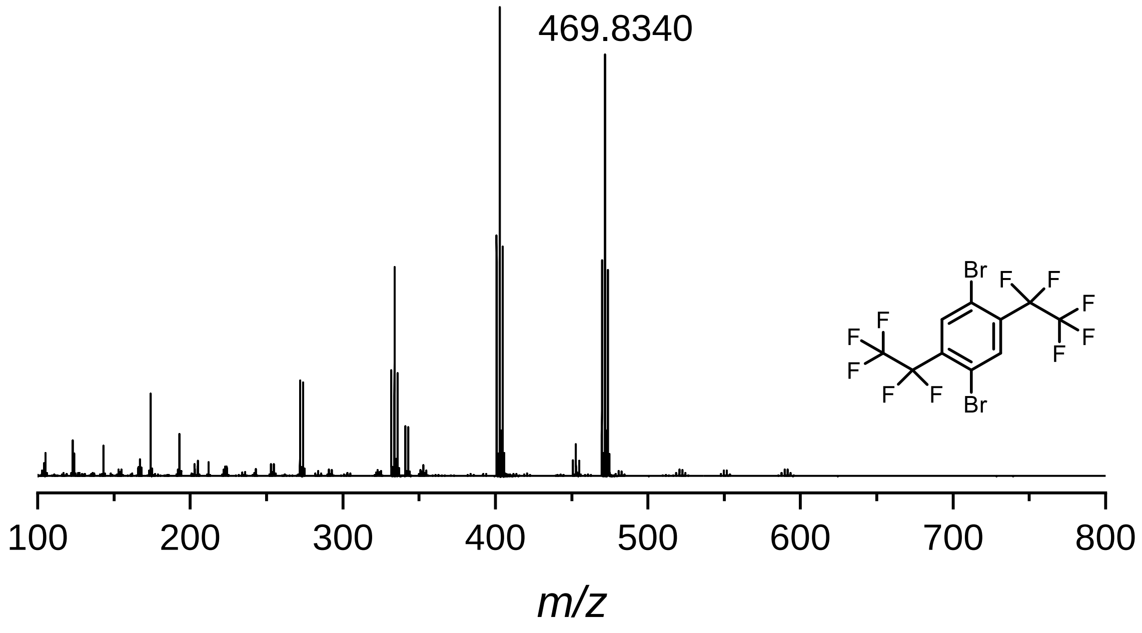

Figure S96. Mass spectrum (EI^+) of 1,4-dibromo-2,5-bis(perfluoroethyl)benzene (**S6**).

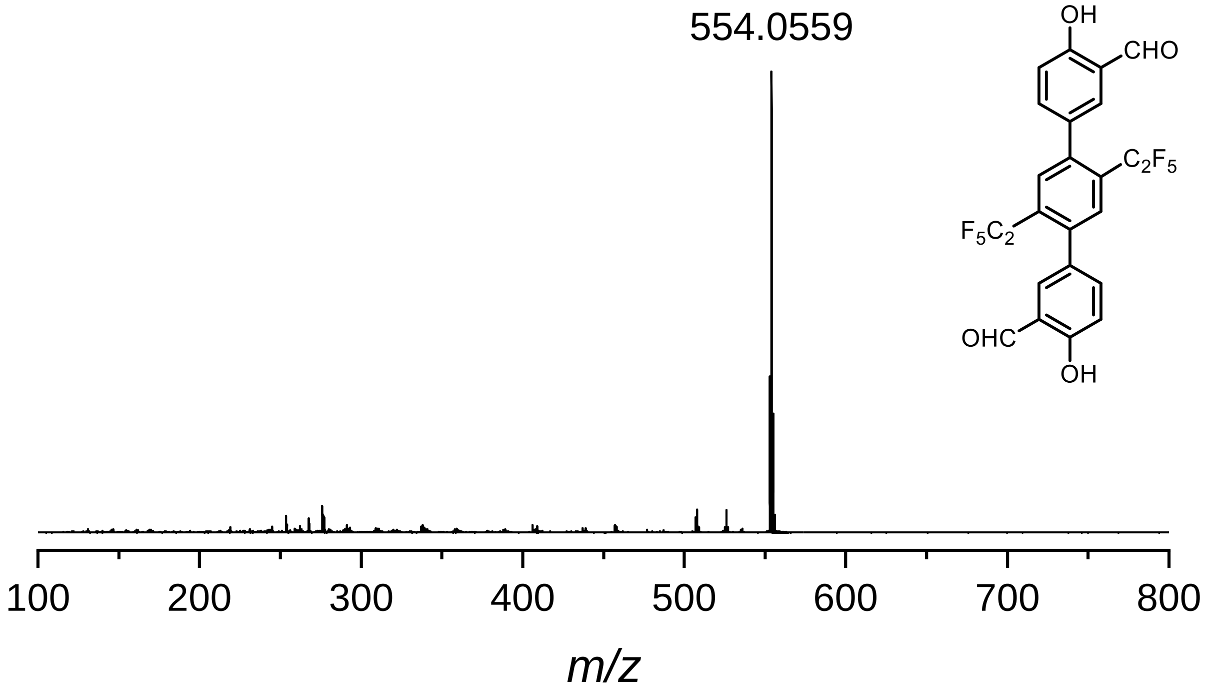

Figure S97. Mass spectrum (EI^+) of 4,4''-dihydroxy-2',5'-bis(perfluoroethyl)-[1,1':4',1''-terphenyl]-3,3''-dicarbaldehyde (**2b**).

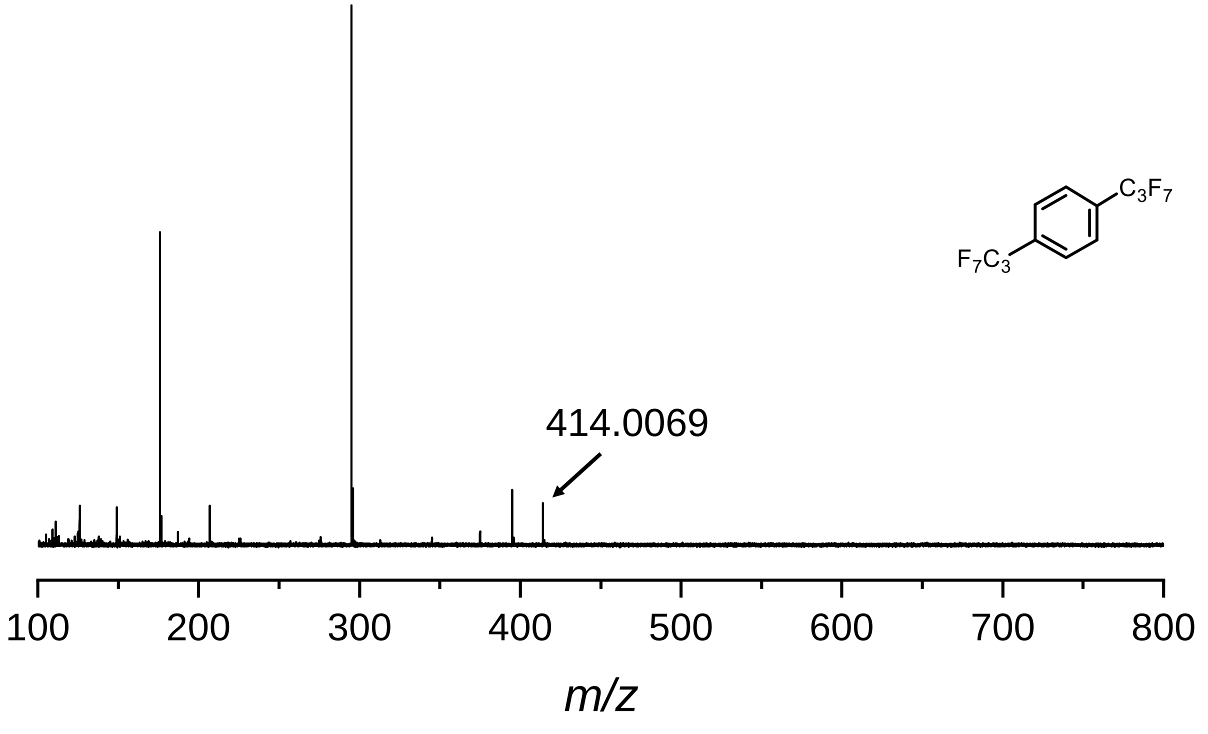

Figure S98. Mass spectrum (EI^+) of 1,4-bis(perfluoropropyl)benzene (**S8**).

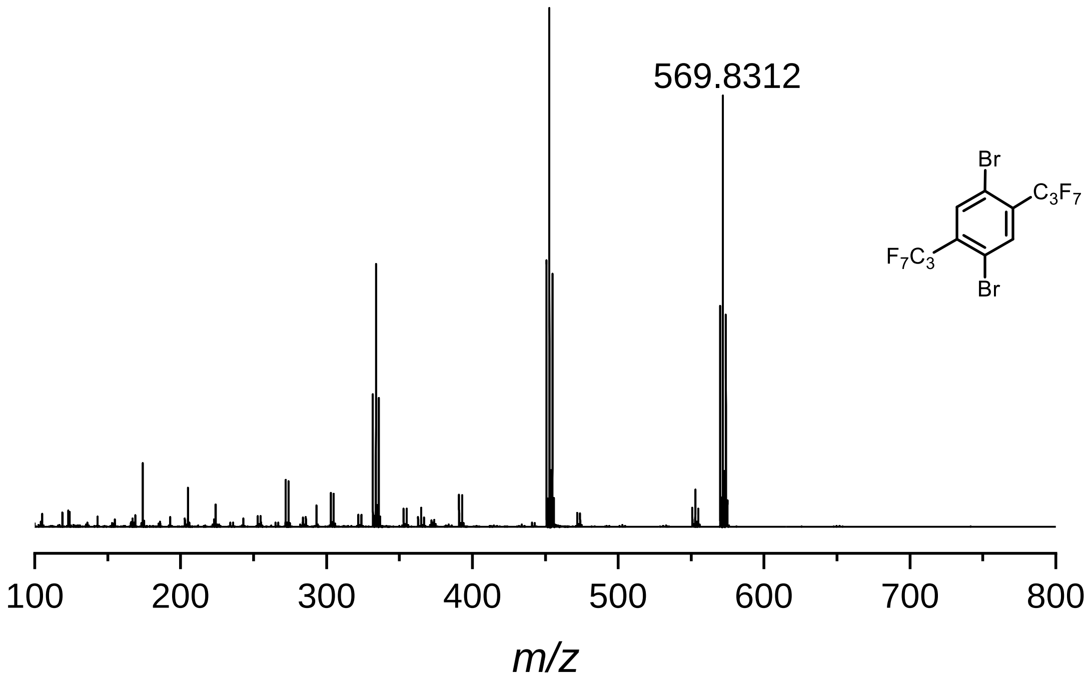

Figure S99. Mass spectrum (EI^+) of 1,4-dibromo-2,5-bis(perfluoropropyl)benzene (**S9**).

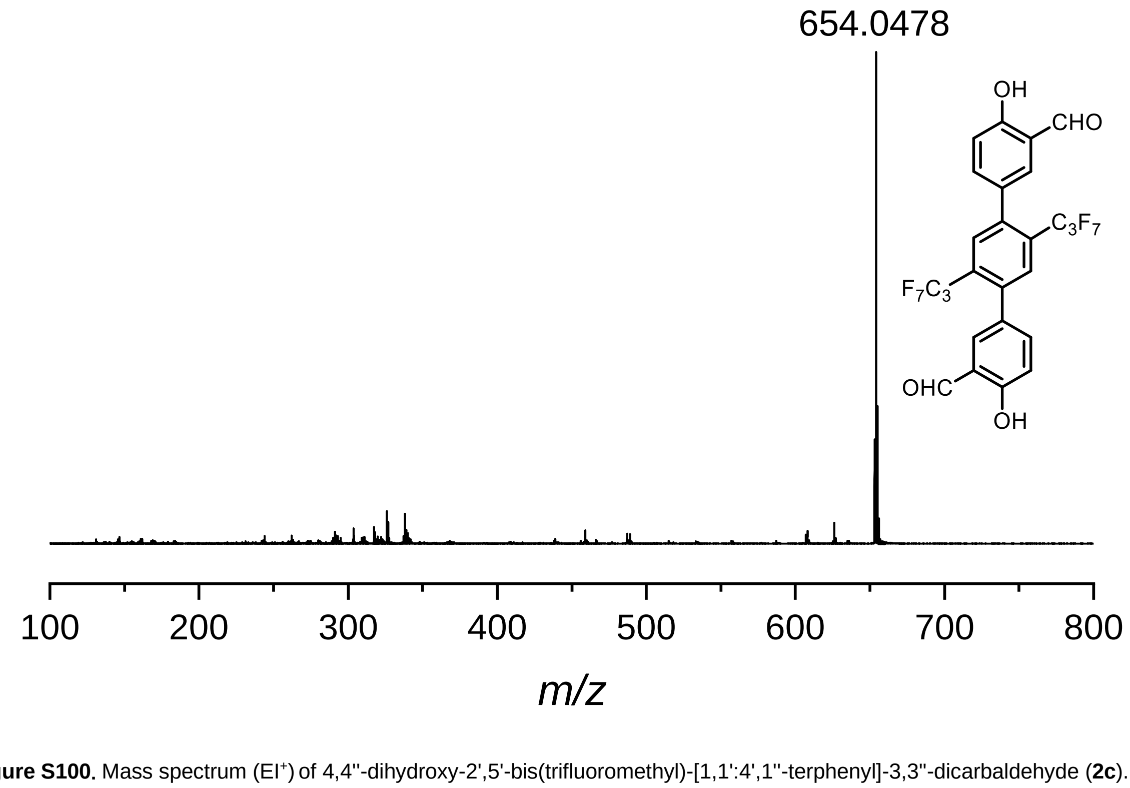

Figure S100. Mass spectrum (EI^+) of 4,4''-dihydroxy-2',5'-bis(trifluoromethyl)-[1,1':4',1''-terphenyl]-3,3''-dicarbaldehyde (**2c**).

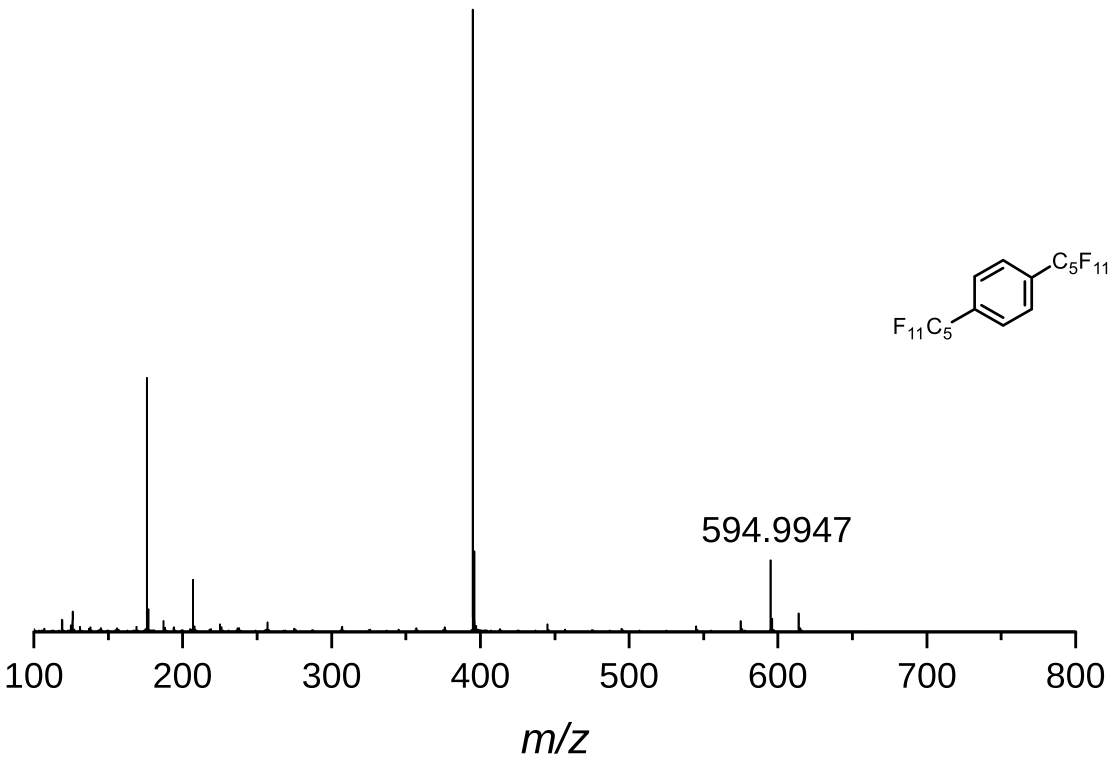

Figure S101. Mass spectrum (EI$^+$) of 1,4-bis(perfluoropentyl)benzene (**S11**).

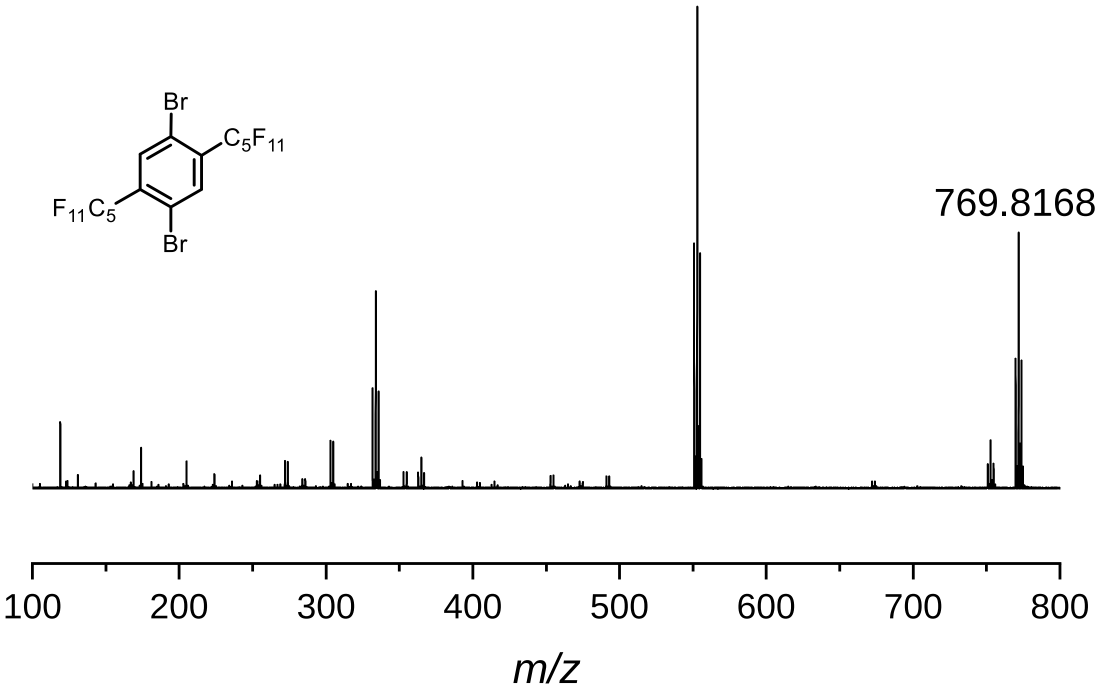

Figure S102. Mass spectrum (EI$^+$) of 1,4-dibromo-2,5-bis(perfluoropentyl) benzene (**S12**).

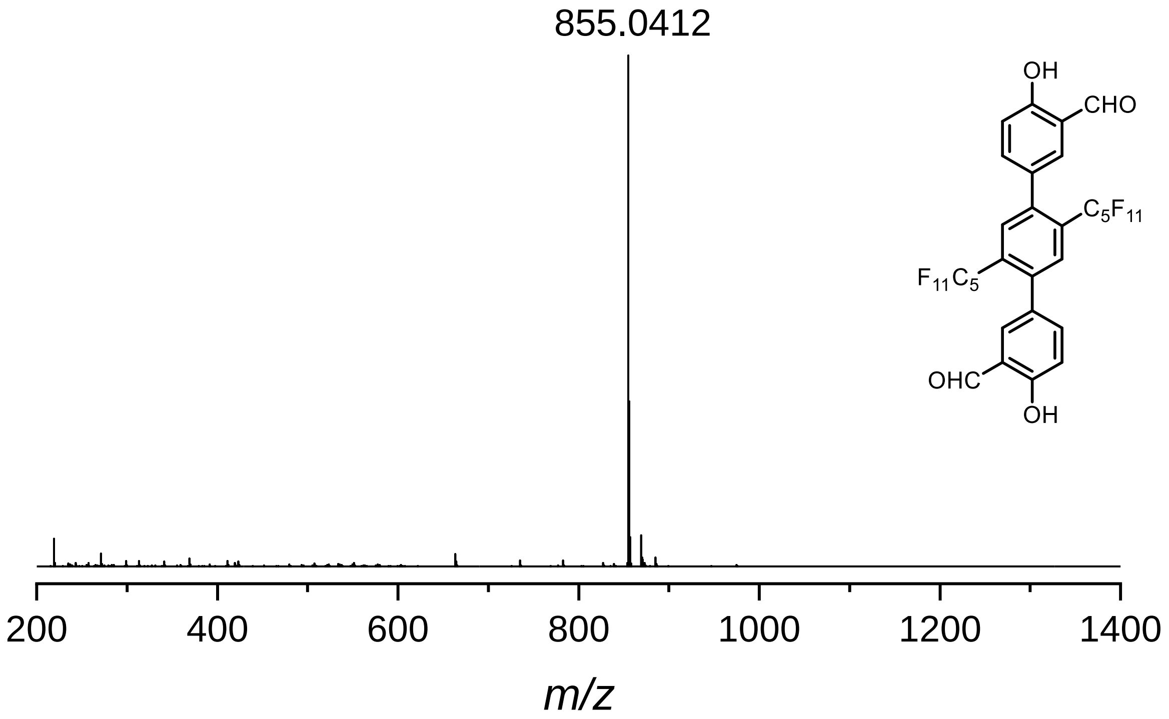

Figure S103. Mass spectrum (APCI$^+$) of 4,4''-dihydroxy-2',5'-bis(perfluoropentyl)-[1,1':4',1''-terphenyl]-3,3''-dicarbaldehyde (**2e**).

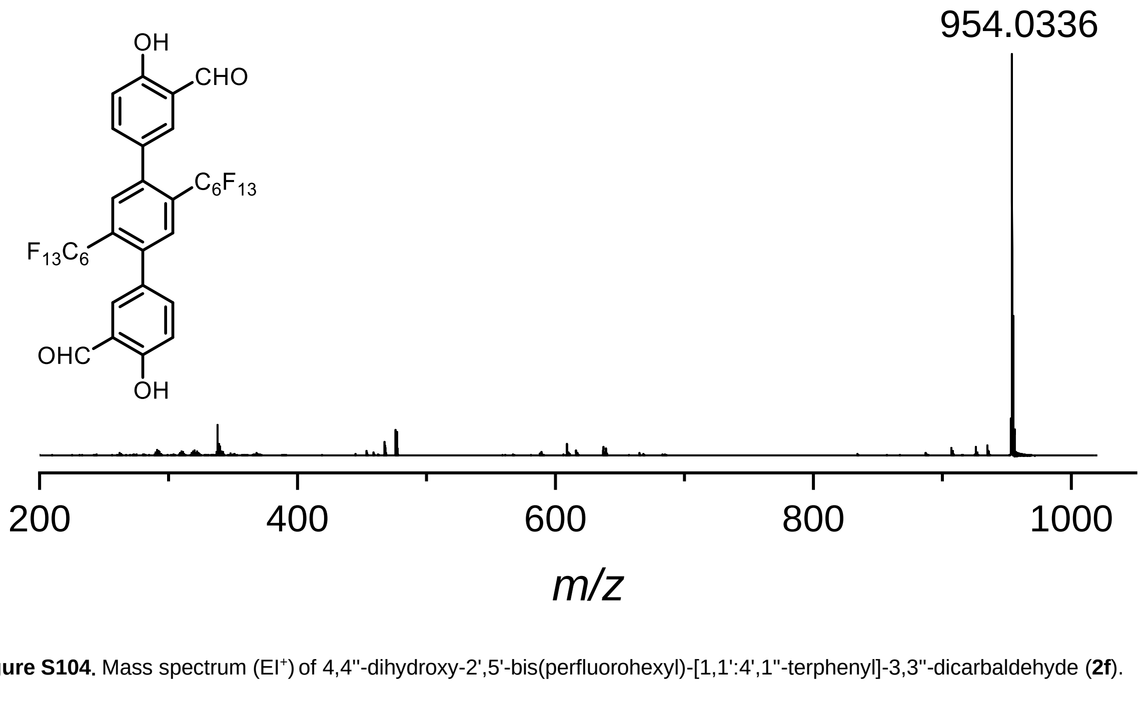

Figure S104. Mass spectrum (EI$^+$) of 4,4''-dihydroxy-2',5'-bis(perfluorohexyl)-[1,1':4',1''-terphenyl]-3,3''-dicarbaldehyde (**2f**).

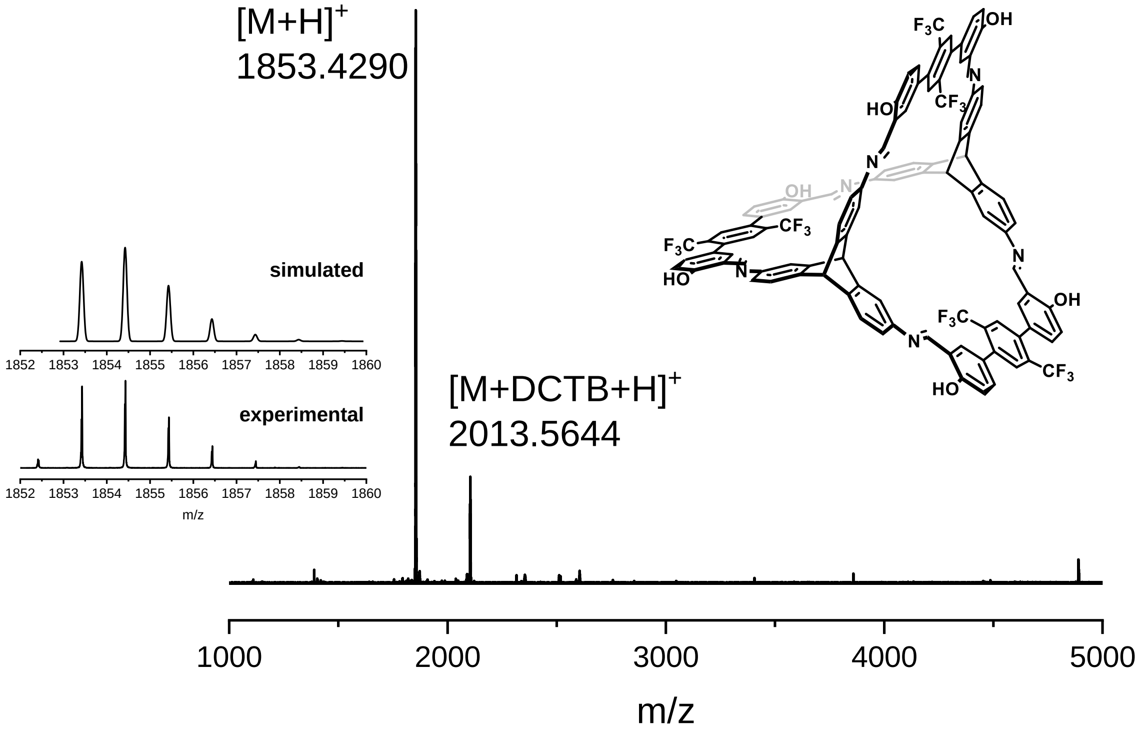

Figure S105. Mass spectrum of CF_3- cage (MALDI-TOF, matrix: DCTB).

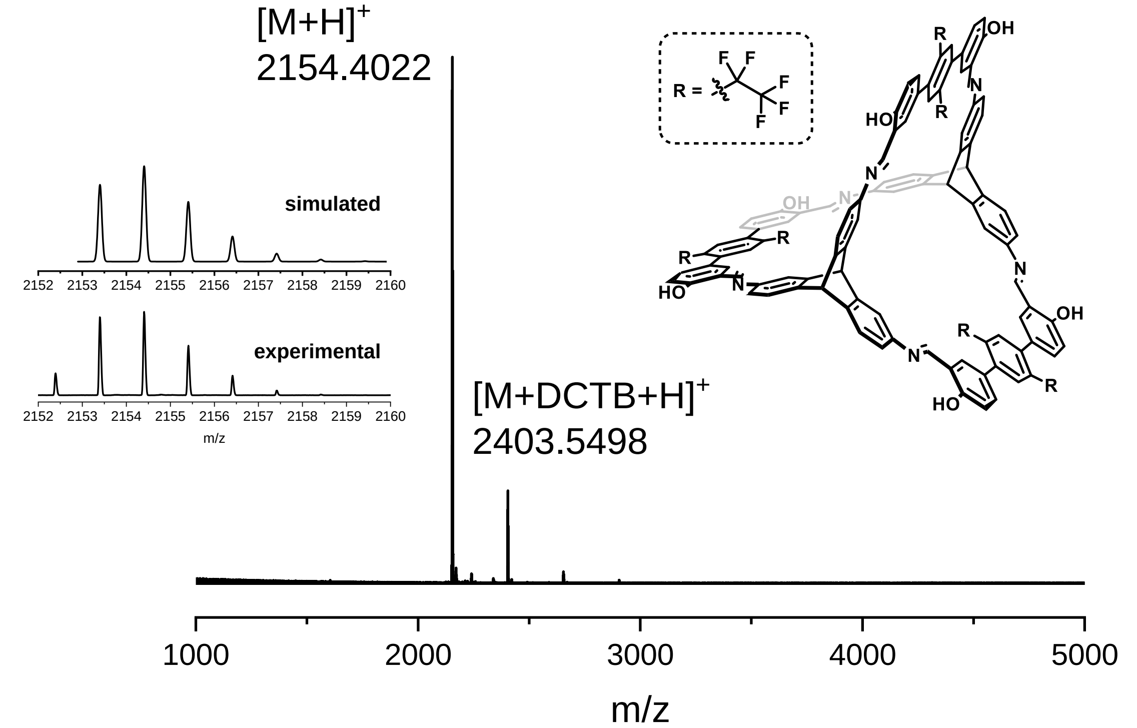

Figure S106. Mass spectrum of C_2F_5-cage (MALDI-TOF, matrix: DCTB).

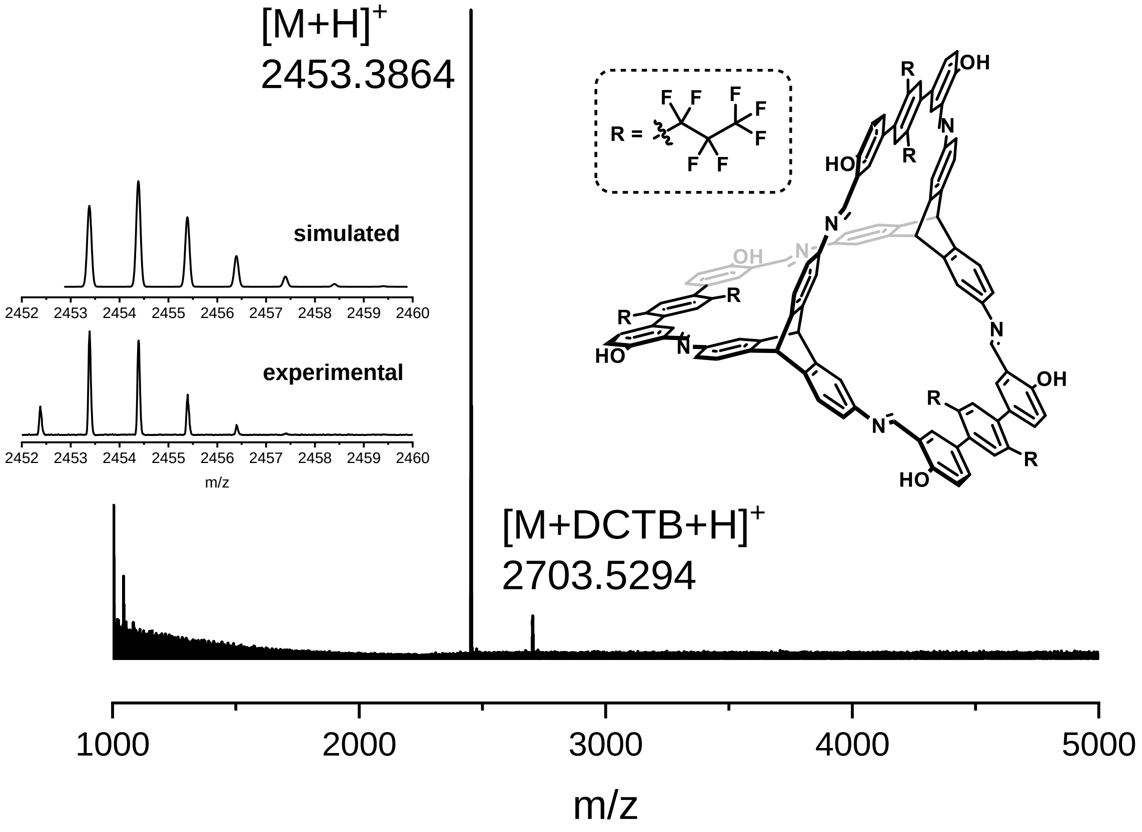

Figure S107. Mass spectrum of C_3F_7-cage- (MALDI-TOF, matrix: DCTB).

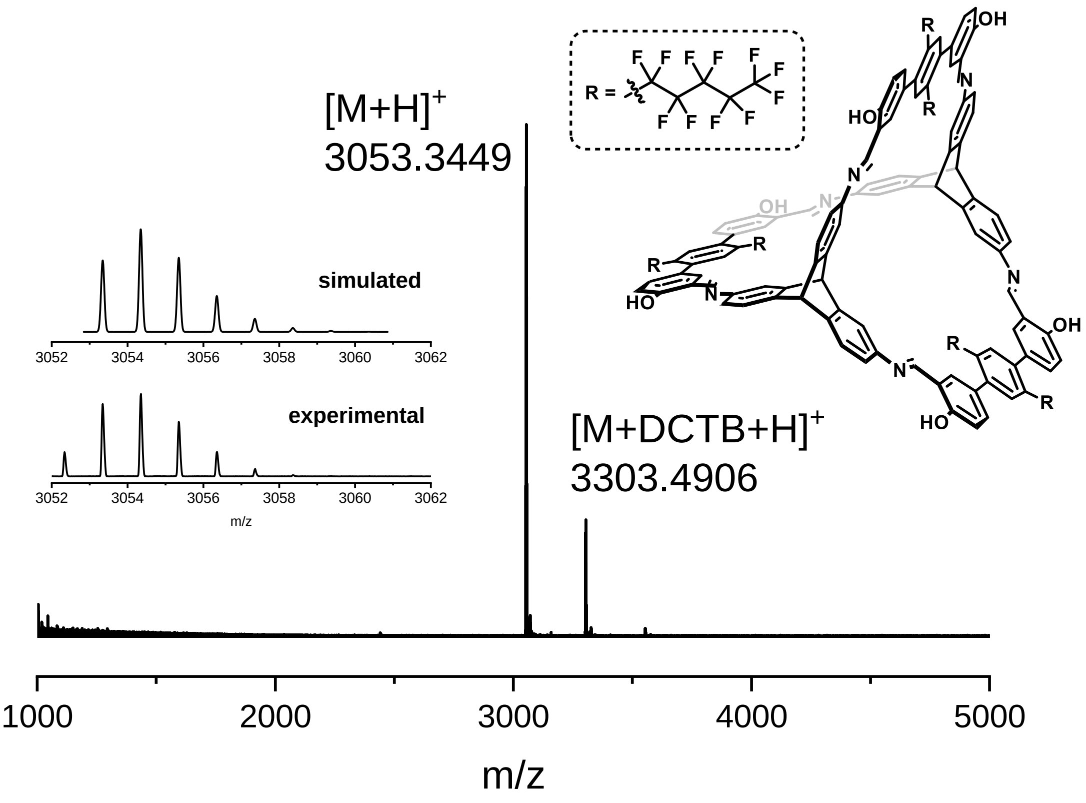

Figure S108. Mass spectrum of C_5F_{11}-cage (MALDI-TOF, matrix: DCTB).

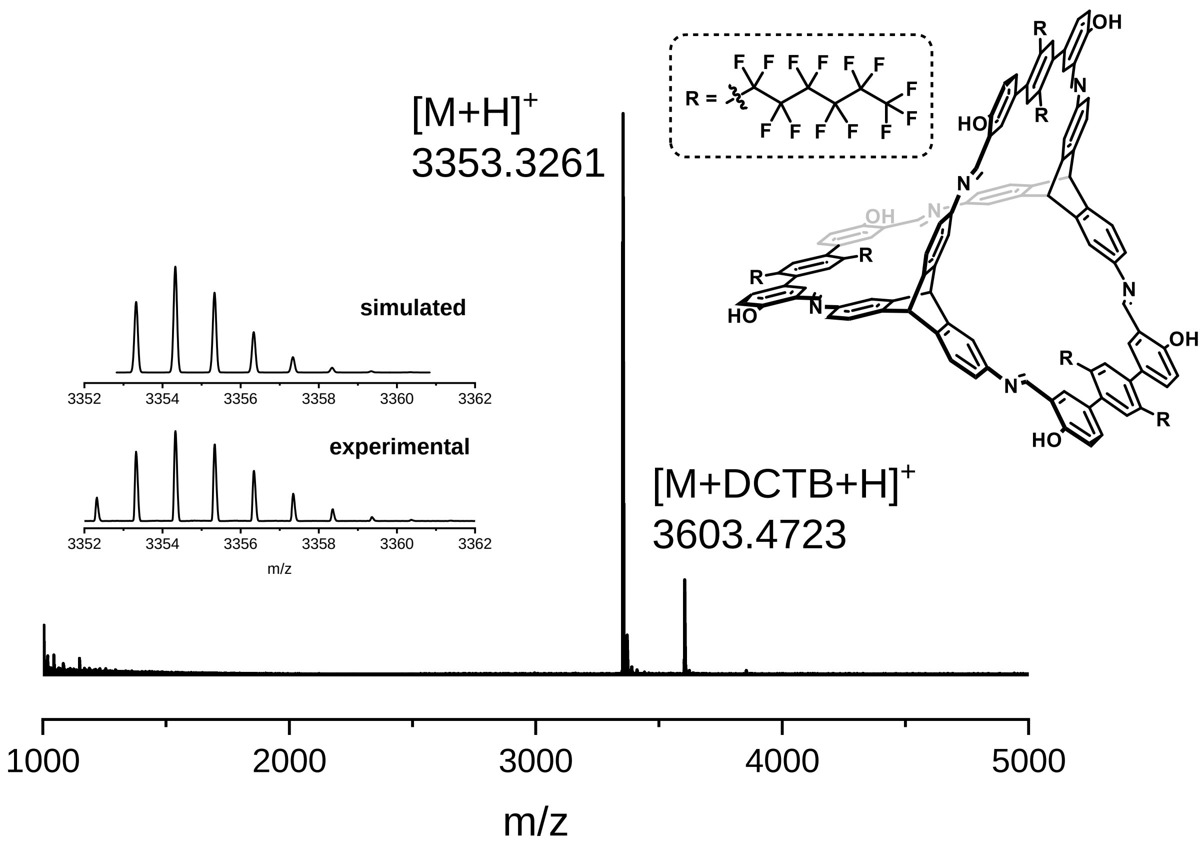

Figure S109. Mass spectrum of C_6F_{13}-cage- (MALDI-TOF, matrix: DCTB).

5. IR Spectroscopy

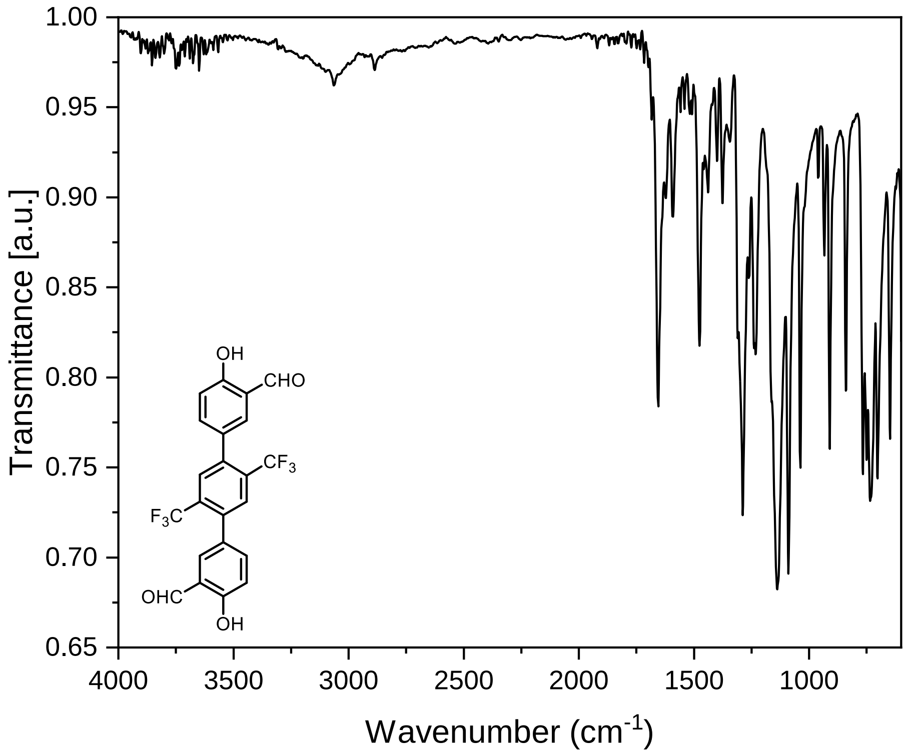

Figure S110. IR spectrum (ATR, ZnSe-crystal) of CF_3-salicylaldehyde (**2a**).

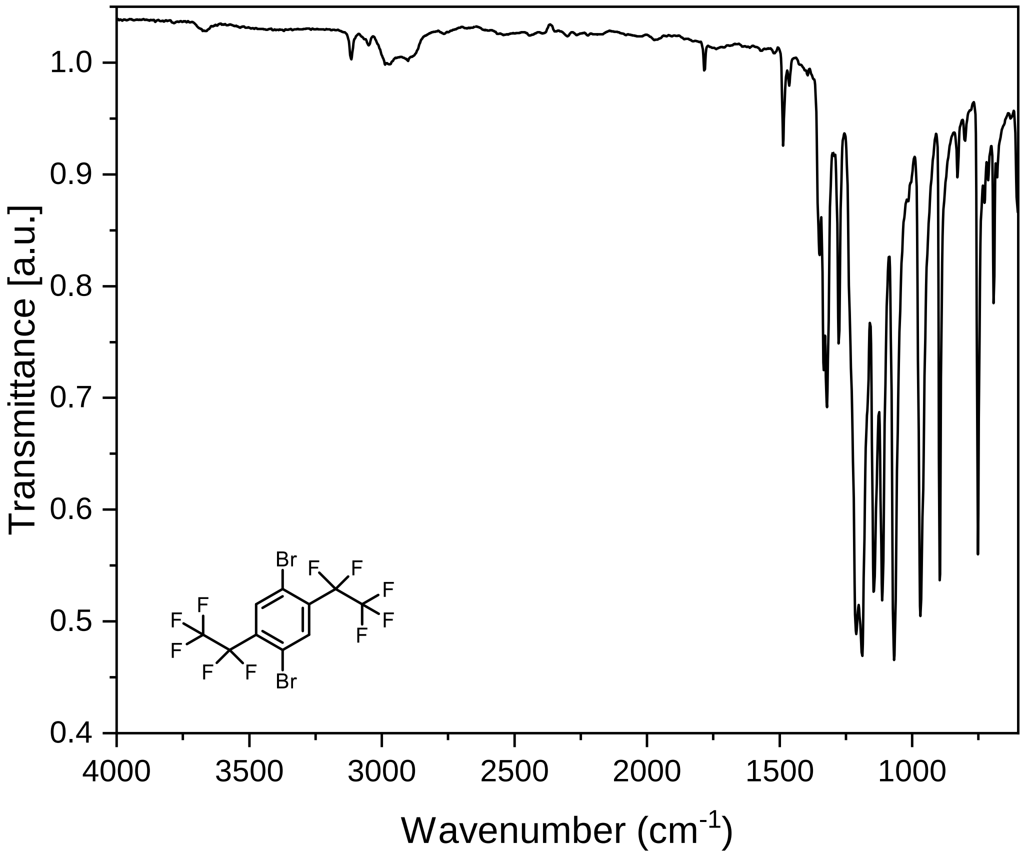

Figure S111. IR spectrum (ATR, ZnSe-crystal) of 1,4-dibromo-2,5-bis(perfluoroethyl)benzene (**S6**).

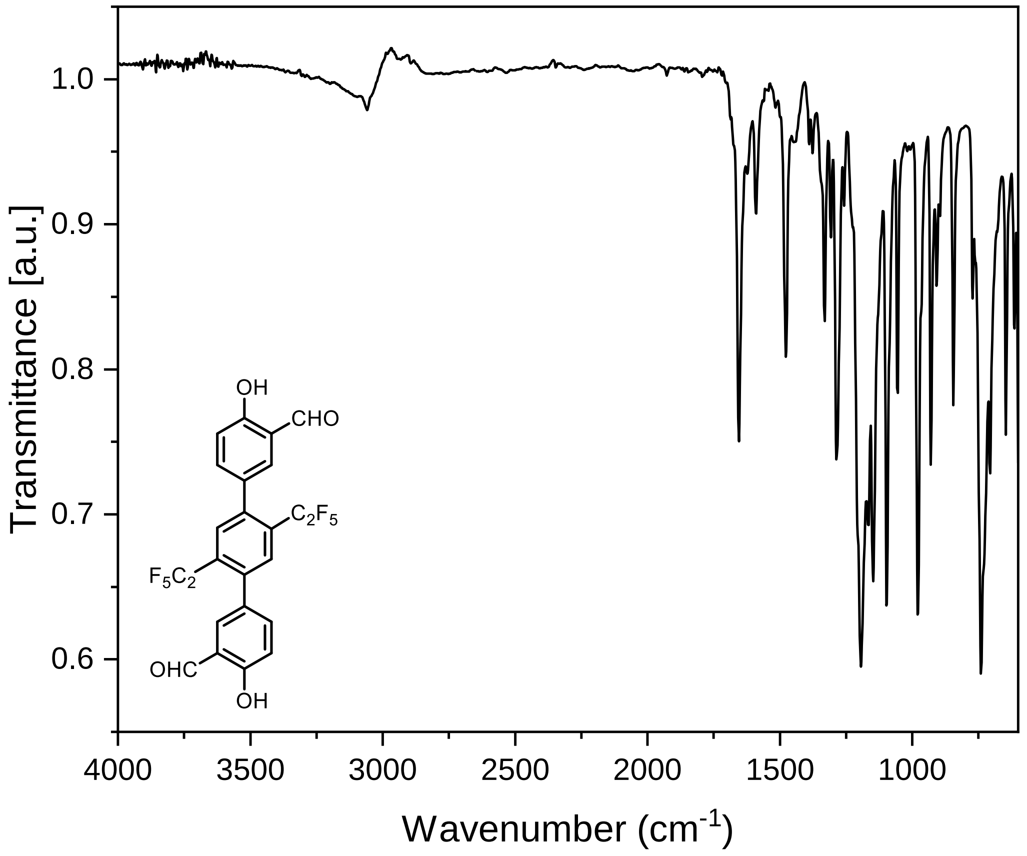

Figure S112. IR spectrum (ATR, ZnSe-crystal) of of 4,4''-dihydroxy-2',5'-bis(perfluoroethyl)-[1,1':4',1''-terphenyl]-3,3''-dicarbaldehyde (**2b**).

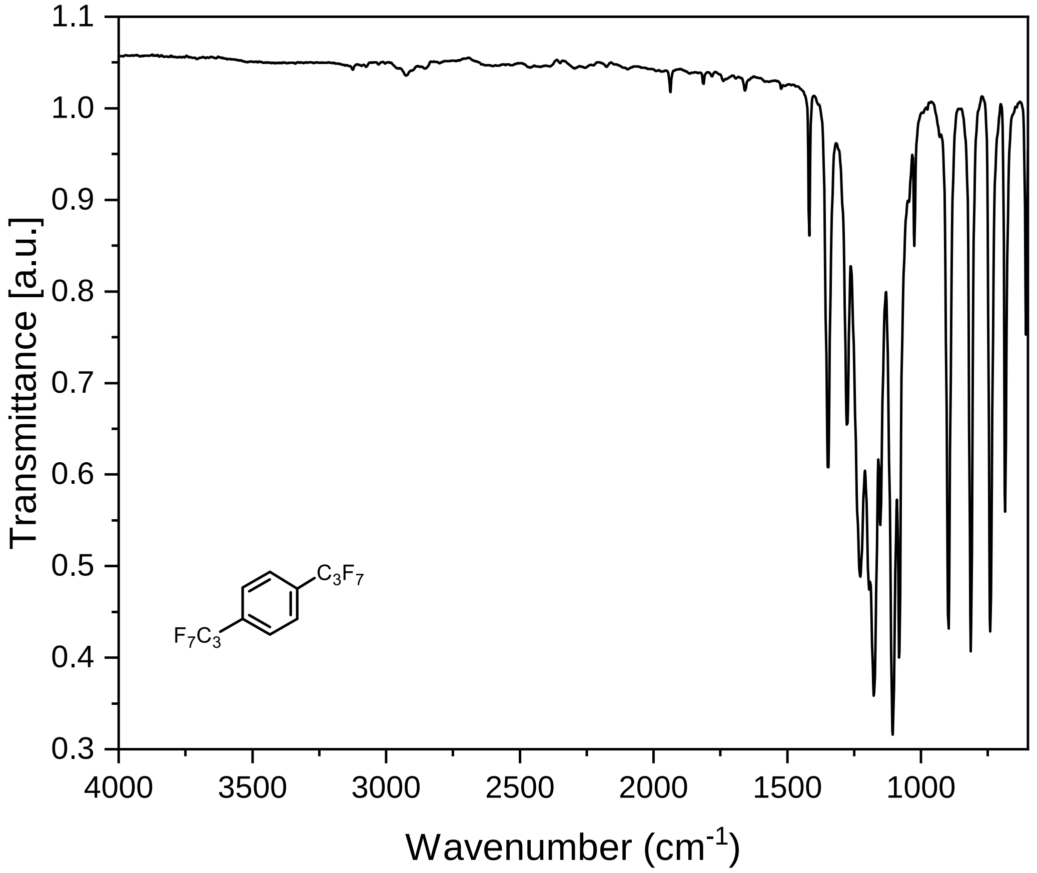

Figure S113. IR spectrum (ATR, ZnSe-crystal) of 1,4-bis(perfluoropropyl)benzene (**S8**).

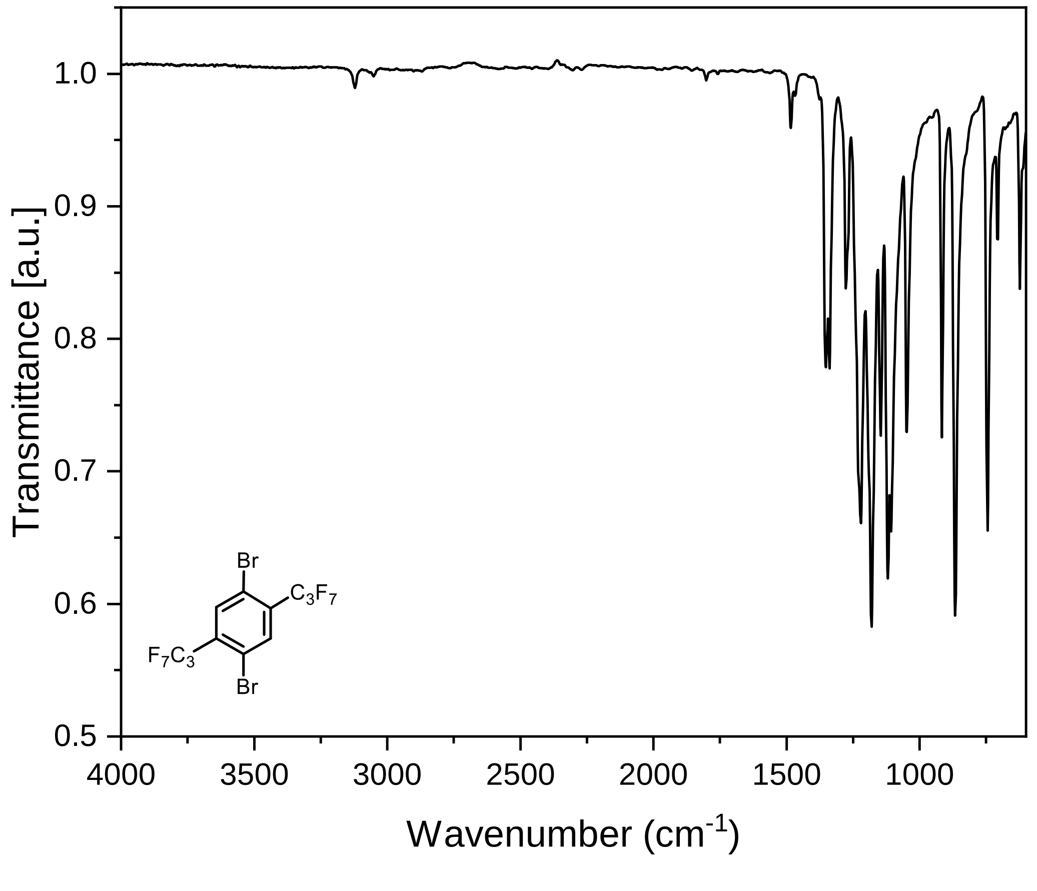

Figure S114. IR spectrum (ATR, ZnSe-crystal) of 1,4-dibromo-2,5-bis(perfluoropropyl)benzene (**S9**).

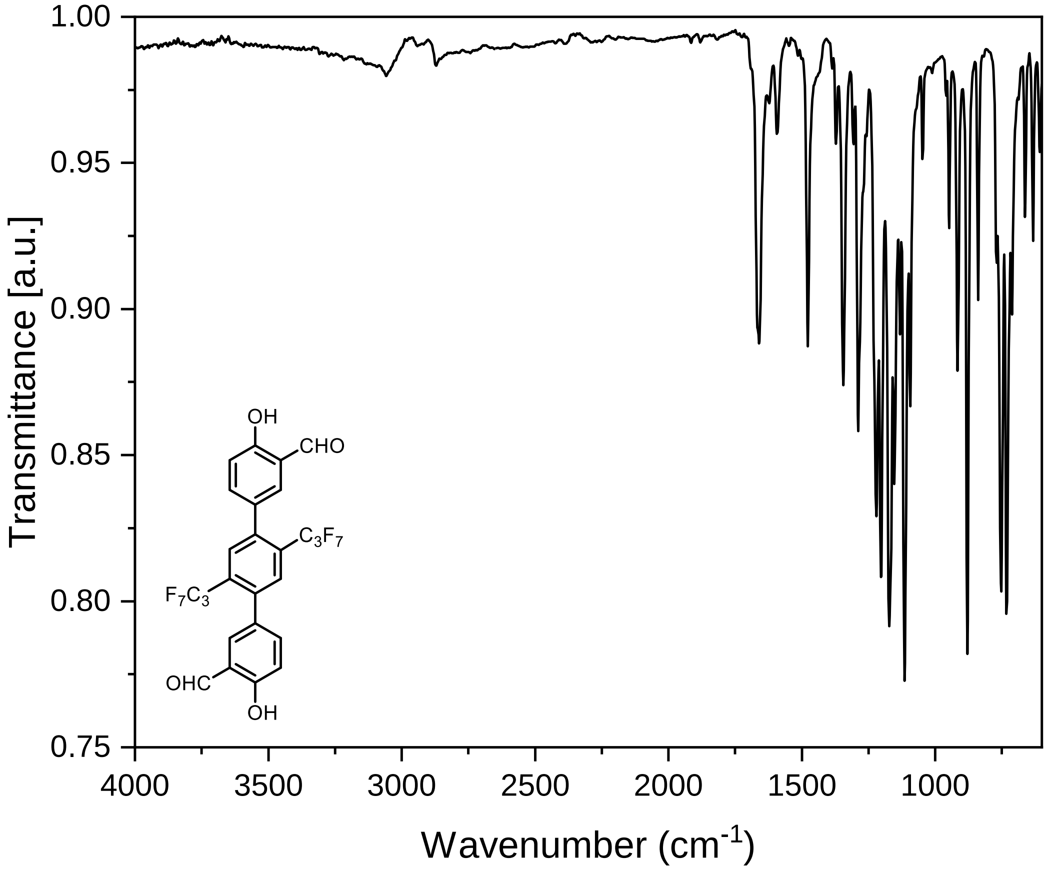

Figure S115. IR spectrum (ATR, ZnSe-crystal) of 4,4''-dihydroxy-2',5'-bis(trifluoromethyl)-[1,1':4',1''-terphenyl]-3,3''-dicarbaldehyde (**2c**).

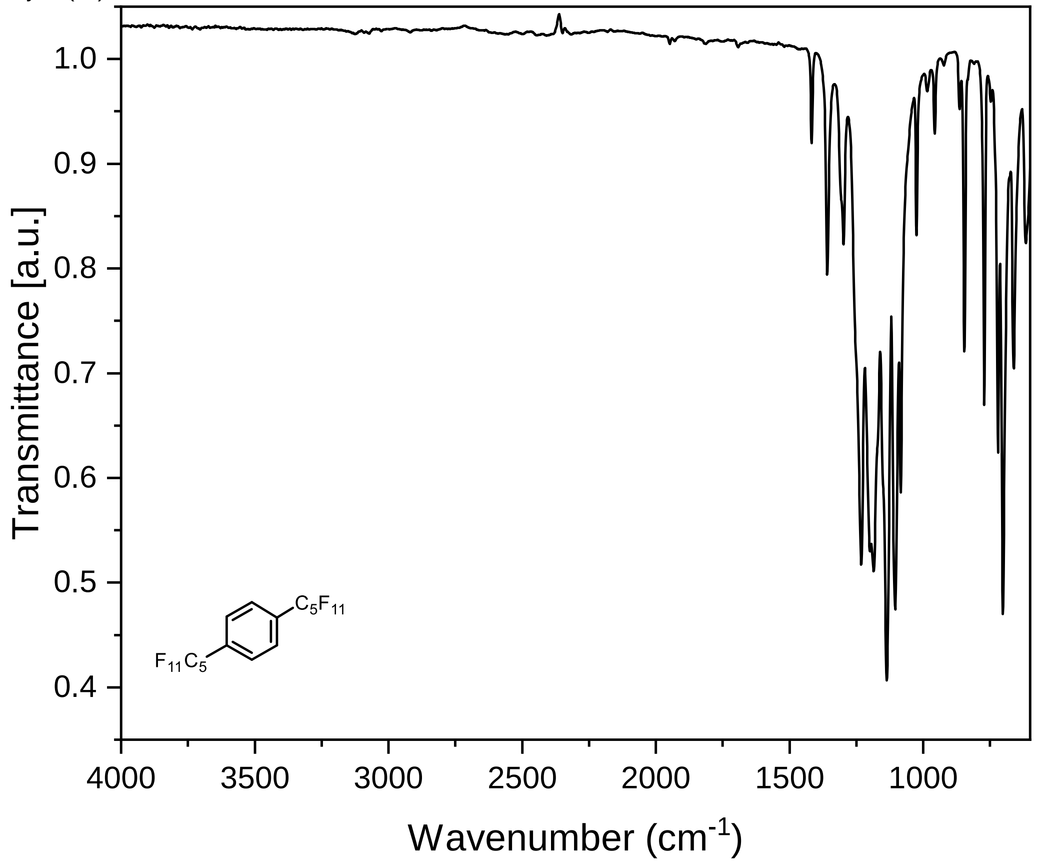

Figure S116. IR spectrum (ATR, ZnSe-crystal) of 1,4-bis(perfluoropentyl)benzene (**S11**).

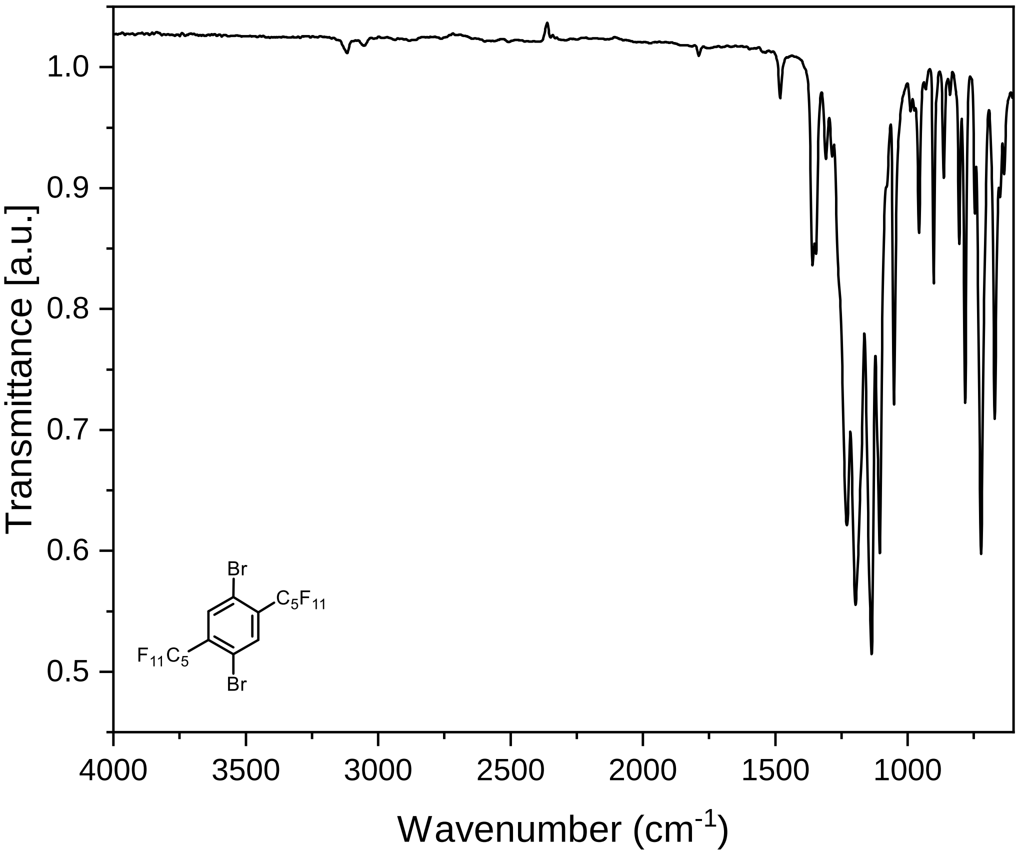

Figure S117. IR spectrum (ATR, ZnSe-crystal) of 1,4-dibromo-2,5-bis(perfluoropentyl) benzene (**S12**).

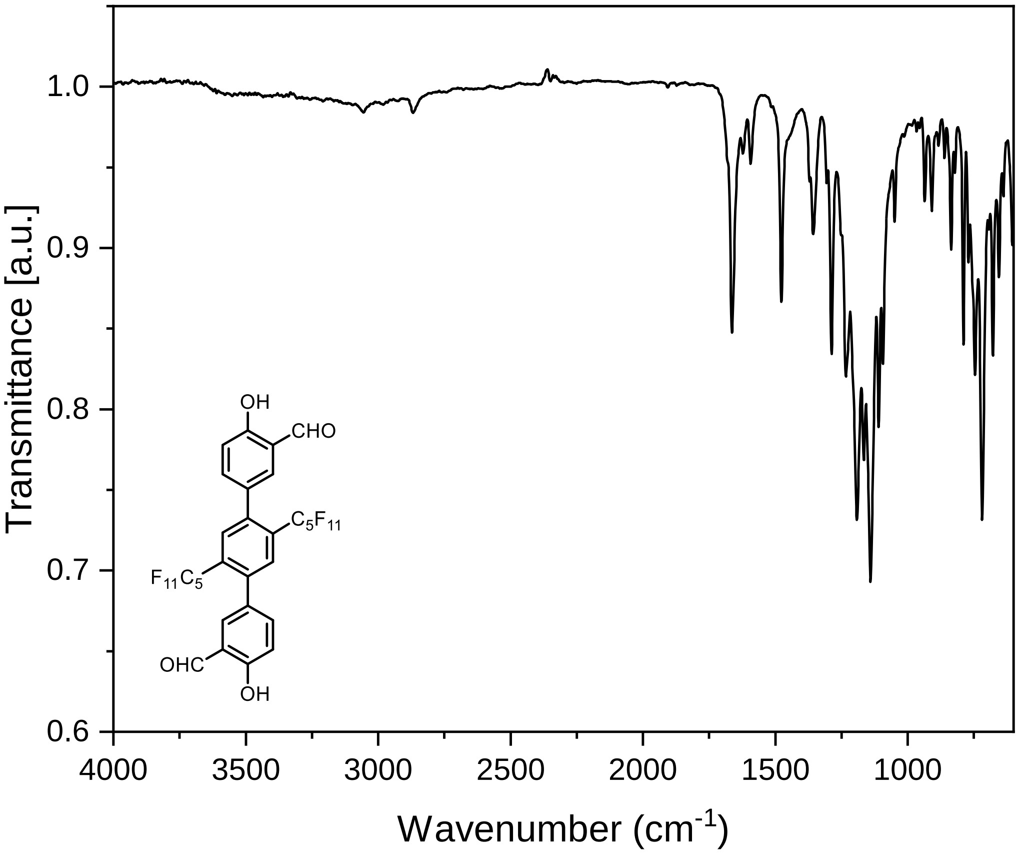

Figure S118. IR spectrum (ATR, ZnSe-crystal) of 4,4''-dihydroxy-2',5'-bis(perfluoropentyl)-[1,1':4',1''-terphenyl]-3,3''-dicarbaldehyde (**2e**).

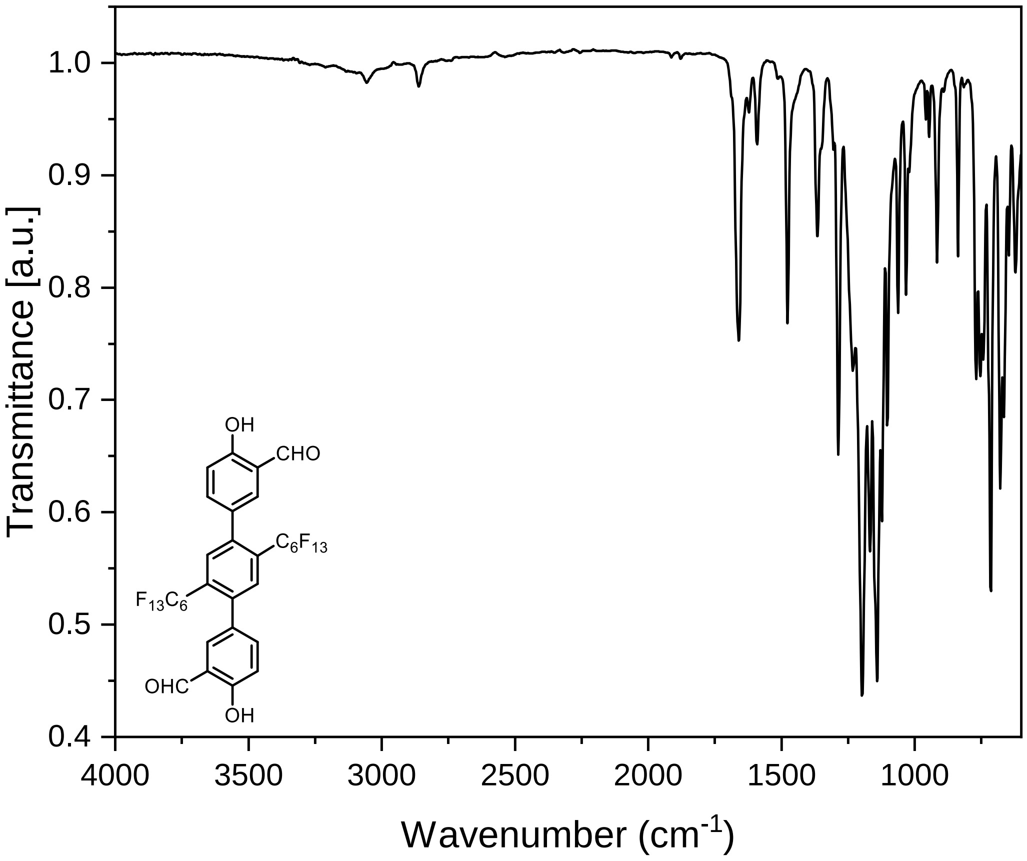

Figure S119. IR spectrum (ATR, ZnSe-crystal) of 4,4''-dihydroxy-2',5'-bis(perfluorohexyl)-[1,1':4',1''-terphenyl]-3,3''-dicarbaldehyde (**2f**).

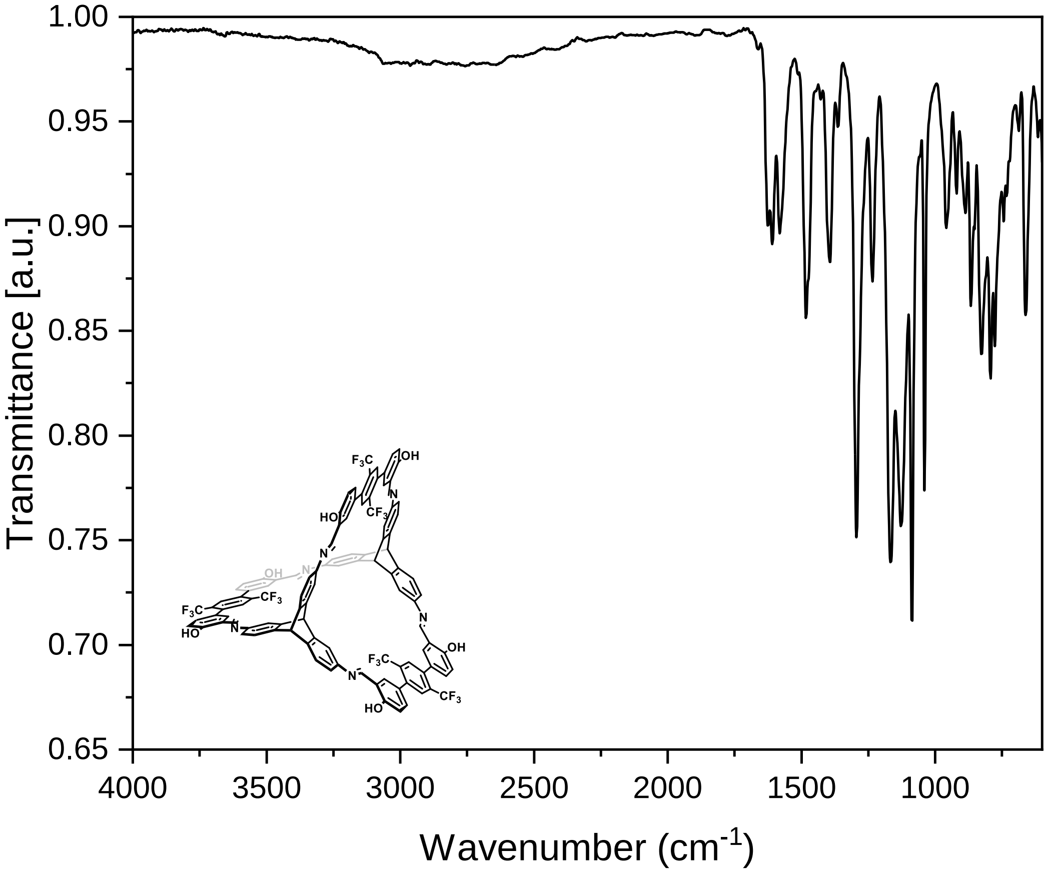

Figure S120. IR spectrum (ATR, ZnSe-crystal) of CF_3-cage.

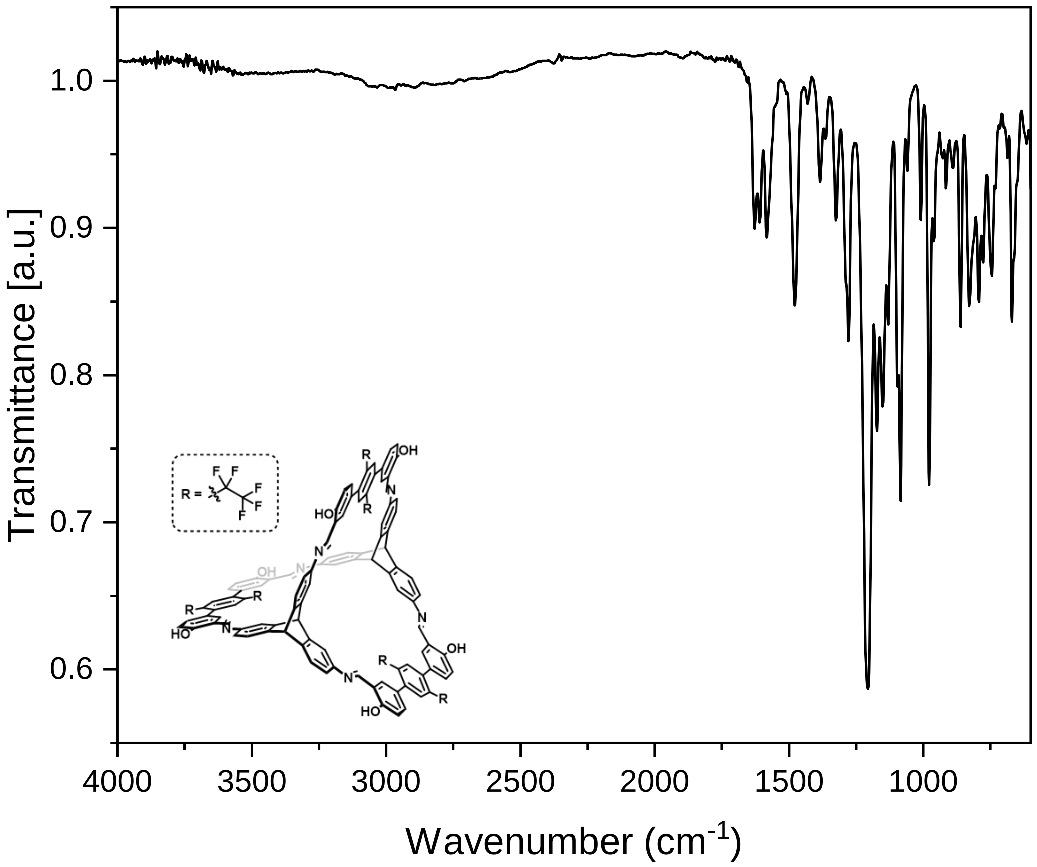

Figure S121. IR spectrum (ATR, ZnSe-crystal) of C_2F_5-cage.

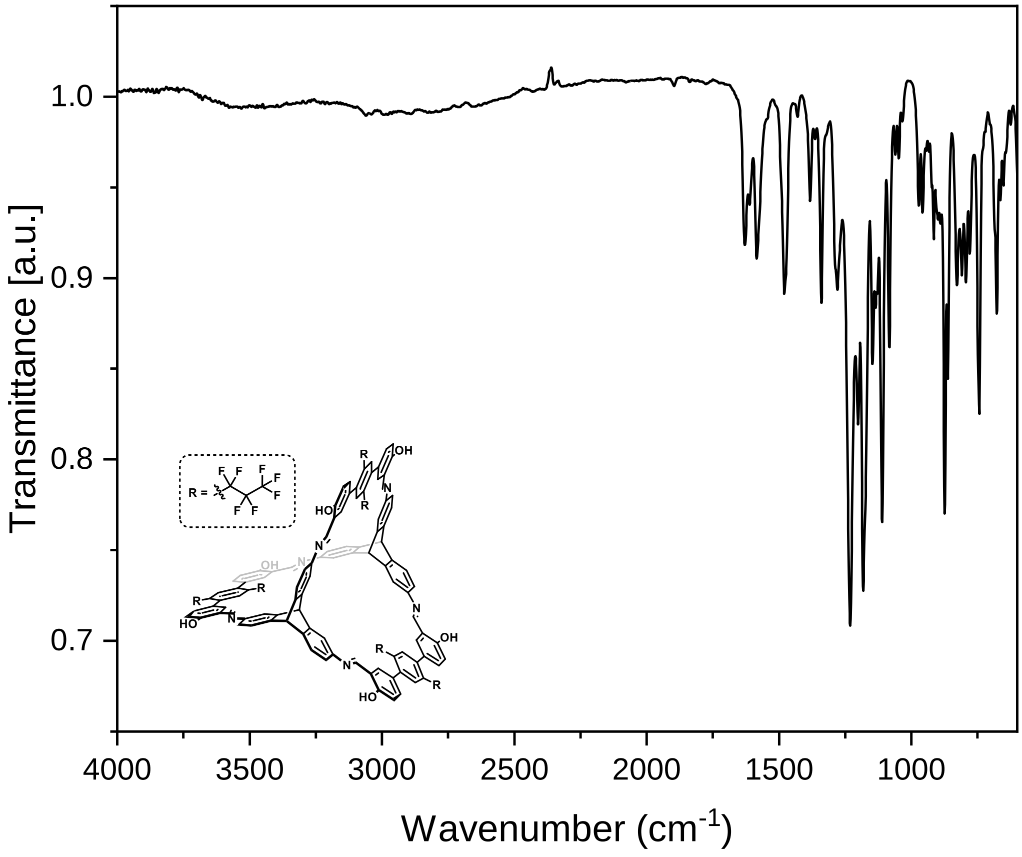

Figure S122. IR spectrum (ATR, ZnSe-crystal) of C_3F_7-cage.

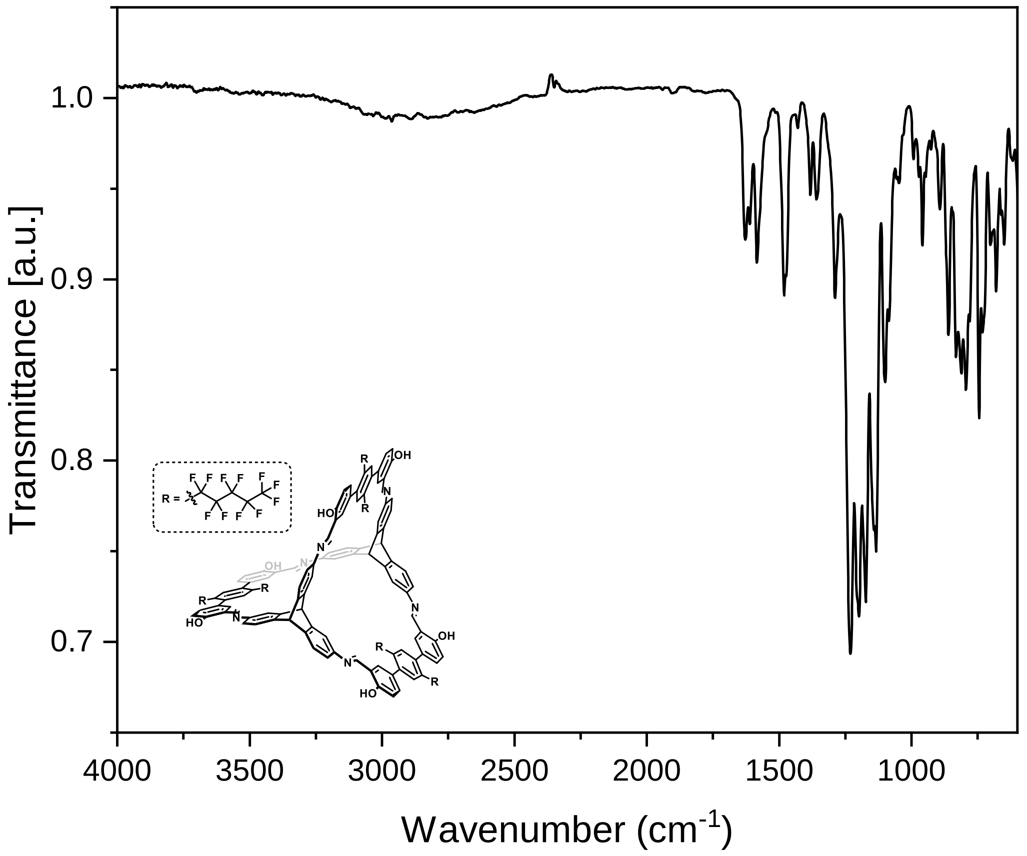

Figure S123. IR spectrum (ATR, ZnSe-crystal) of C_5F_{11}-cage.

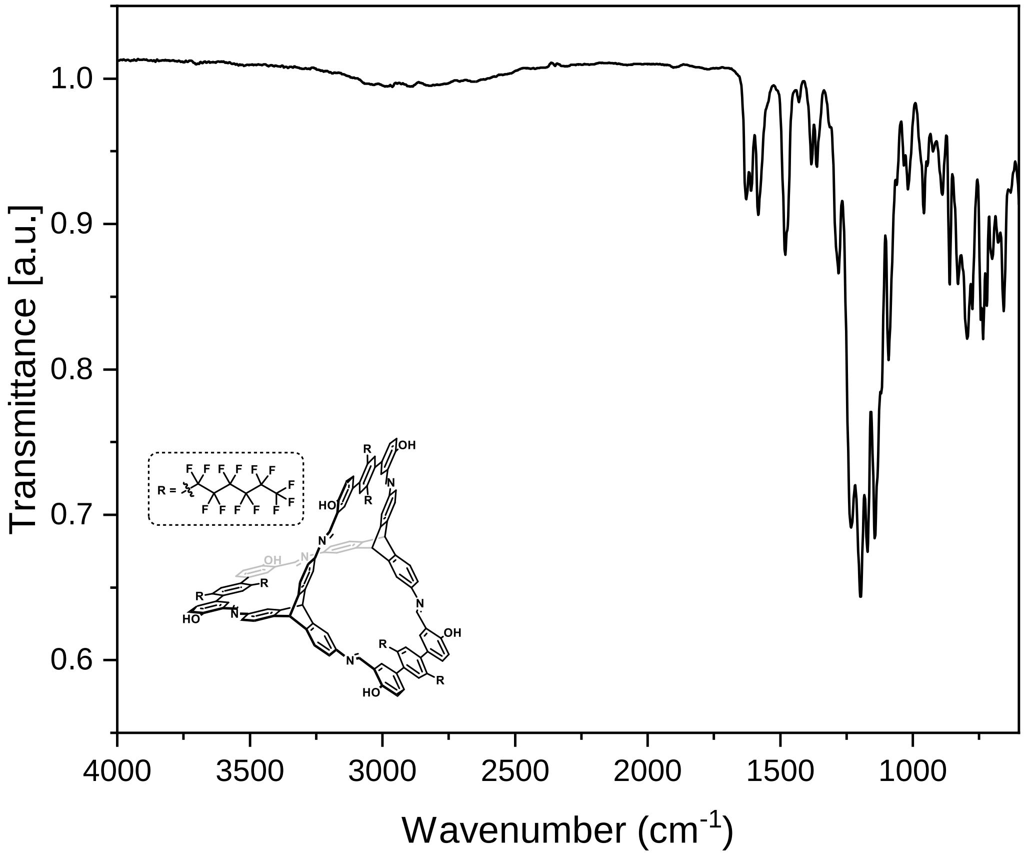

Figure S124. IR spectrum (ATR, ZnSe-crystal) of C_6F_{13}-cage.

6. Single Crystal X-ray Diffraction (SCXRD)

CF_3-cage

Crystals of **CF_3-cage** suitable for single crystal X-ray diffraction have been obtained by vapour diffusion of methanol into a saturated DMF-solution of **CF_3-cage**.

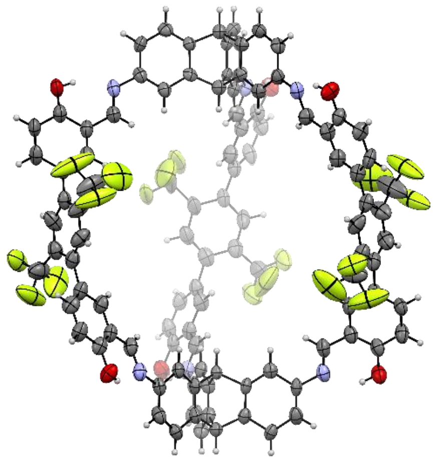

Table S1: Crystal data and structure refinement for **CF_3-cage**.

CCDC-number	2477445	
Empirical formula	$C_{106}H_{58}F_{18}N_6O_6$	
Formula weight	1853.58	
Temperature	200(2) K	
Wavelength	1.54178 Å	
Crystal system	trigonal	
Space group	R3c	
Z	6	
Unit cell dimensions	a = 19.3976(2) Å	α = 90 deg.
	b = 19.3976(2) Å	β = 90 deg.
	c = 60.9238(14) Å	γ = 120 deg.
Volume	19852.4(6) Å^3	
Density (calculated)	0.93 g/cm^3	
Absorption coefficient	0.65 mm^{-1}	
Crystal shape	cuboid	
Crystal size	0.140 x 0.102 x 0.071 mm^3	
Crystal colour	orange	
Theta range for data collection	3.9 to 69.2 deg.	
Index ranges	$-19 \le h \le 20$, $-23 \le k \le 16$, $-51 \le l \le 72$	
Reflections collected	25240	
Independent reflections	5921 (R(int) = 0.0241)	
Observed reflections	4801 ($I > 2\sigma(I)$)	
Absorption correction	Semi-empirical from equivalents	
Max. and min. transmission	1.38 and 0.77	
Refinement method	Full-matrix least-squares on F^2	
Data/restraints/parameters	5921 / 445 / 411	
Goodness-of-fit on F^2	1.06	
Final R indices (I>2sigma(I))	R1 = 0.046, wR2 = 0.123	
Absolute structure parameter	0.51(8)	
Largest diff. peak and hole	0.26 and -0.24 eÅ^{-3}	

C_2F_5-cage

Crystals of **C_2F_5-cage** suitable for single crystal X-ray diffraction have been obtained by vapour diffusion of methanol into a saturated THF-solution of **C_2F_5-cage**.

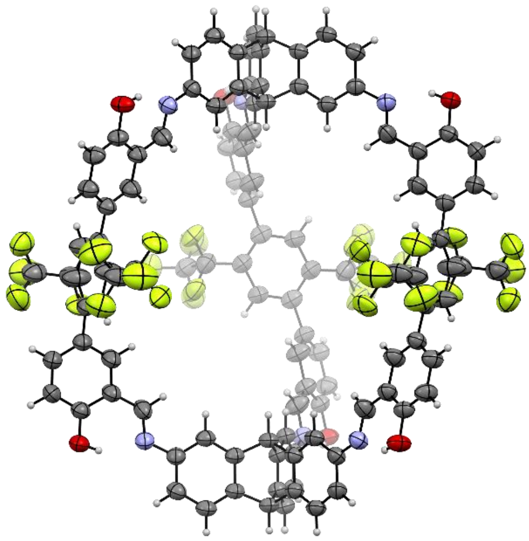

Table S2: Crystal data and structure refinement for **C_2F_5-cage**.

CCDC-number	2477446	
Empirical formula	$C_{112}H_{58}F_{30}N_6O_6$	
Formula weight	2153.64	
Temperature	200(2) K	
Wavelength	1.54178 Å	
Crystal system	trigonal	
Space group	$R\bar{3}c$	
Z	6	
Unit cell dimensions	a = 20.1547(4) Å	α = 90 deg.
	b = 20.1547(4) Å	β = 90 deg.
	c = 77.800(2) Å	γ = 120 deg.
Volume	27369.3(14) $Å^3$	
Density (calculated)	0.78 g/cm^3	
Absorption coefficient	0.61 mm^{-1}	
Crystal shape	brick	
Crystal size	0.083 x 0.078 x 0.068 mm^3	
Crystal colour	orange	
Theta range for data collection	2.8 to 56.0 deg.	
Index ranges	$-21 \le h \le 14$, $-21 \le k \le 19$, $-83 \le l \le 63$	
Reflections collected	29223	
Independent reflections	3965 (R(int) = 0.1002)	
Observed reflections	2276 ($I > 2\sigma(I)$)	
Absorption correction	Semi-empirical from equivalents	
Max. and min. transmission	1.68 and 0.66	
Refinement method	Full-matrix least-squares on F^2	
Data/restraints/parameters	3965 / 335 / 297	
Goodness-of-fit on F^2	0.95	
Final R indices (I>2sigma(I))	R1 = 0.052, wR2 = 0.165	
Largest diff. peak and hole	0.23 and -0.20 $eÅ^{-3}$	

C_3F_7-cage

Crystals of **C_3F_7-cage** suitable for single crystal X-ray diffraction have been obtained by vapour diffusion of methanol into a saturated THF-solution of **C_3F_7-cage**.

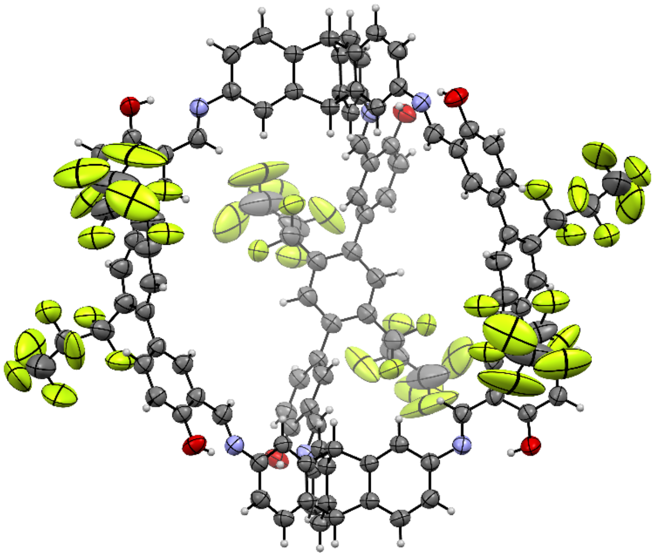

Table S3: Crystal data and structure refinement for **C_3F_7-cage**.

CCDC-number	2477447	
Empirical formula	$C_{118}H_{58}F_{42}N_6O_6$	
Formula weight	2453.70	
Temperature	200(2) K	
Wavelength	1.54178 Å	
Crystal system	trigonal	
Space group	R $\bar{3}$ c	
Z	6	
Unit cell dimensions	a = 19.8267(1) Å	α = 90 deg.
	b = 19.8267(1) Å	β = 90 deg.
	c = 62.2239(11) Å	γ = 120 deg.
Volume	21183.1(5) Å^3	
Density (calculated)	1.15 g/cm^3	
Absorption coefficient	0.98 mm^{-1}	
Crystal shape	cuboid	
Crystal size	0.166 x 0.165 x 0.110 mm^3	
Crystal colour	yellow	
Theta range for data collection	3.8 to 71.7 deg.	
Index ranges	$-20 \le h \le 24$, $-24 \le k \le 20$, $-42 \le l \le 75$	
Reflections collected	33400	
Independent reflections	4536 (R(int) = 0.0425)	
Observed reflections	3394 ($I > 2\sigma(I)$)	
Absorption correction	Semi-empirical from equivalents	
Max. and min. transmission	0.90 and 0.41	
Refinement method	Full-matrix least-squares on F^2	
Data/restraints/parameters	4536 / 591 / 351	
Goodness-of-fit on F^2	1.08	
Final R indices (I>2sigma(I))	R1 = 0.061, wR2 = 0.194	
Largest diff. peak and hole	0.26 and -0.34 eÅ^{-3}	

C_5F_{11}-cage

Crystals of **C_5F_{11}-cage** suitable for single crystal X-ray diffraction have been obtained by vapour diffusion of methanol into a saturated THF-solution of **C_5F_{11}-cage**.

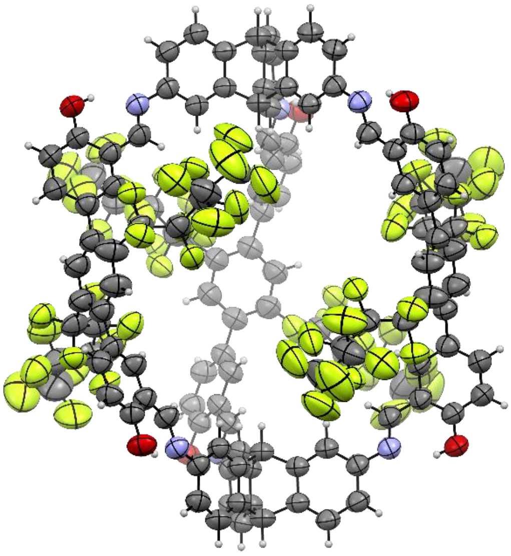

Table S4: Crystal data and structure refinement for **C_5F_{11}-cage**.

CCDC-number	2477448	
Empirical formula	$C_{130}H_{58}F_{66}N_6O_6$	
Formula weight	3053.82	
Temperature	200(2) K	
Wavelength	1.54178 Å	
Crystal system	trigonal	
Space group	R $\bar{3}$ c	
Z	6	
Unit cell dimensions	a = 19.5342(4) Å	α = 90 deg.
	b = 19.5342(4) Å	β = 90 deg.
	c = 66.037(2) Å	γ = 120 deg.
Volume	21822.8(12) $Å^3$	
Density (calculated)	1.39 g/cm^3	
Absorption coefficient	1.30 mm^{-1}	
Crystal shape	cuboid	
Crystal size	0.170 x 0.080 x 0.077 mm^3	
Crystal colour	yellow	
Theta range for data collection	3.7 to 57.9 deg.	
Index ranges	$-20 \le h \le 19$, $-17 \le k \le 21$, $-42 \le l \le 72$	
Reflections collected	16543	
Independent reflections	3375 (R(int) = 0.0314)	
Observed reflections	2361 ($I > 2\sigma(I)$)	
Absorption correction	Semi-empirical from equivalents	
Max. and min. transmission	0.96 and 0.74	
Refinement method	Full-matrix least-squares on F^2	
Data/restraints/parameters	3375 / 2920 / 605	
Goodness-of-fit on F^2	1.03	
Final R indices (I>2sigma(I))	R1 = 0.079, wR2 = 0.227	
Largest diff. peak and hole	0.25 and -0.19 $eÅ^{-3}$	

C_6F_{13}-cage

Crystals of **C_6F_{13}-cage** suitable for single crystal X-ray diffraction have been obtained by vapour diffusion of methanol into a saturated THF-solution of **C_6F_{13}-cage**.

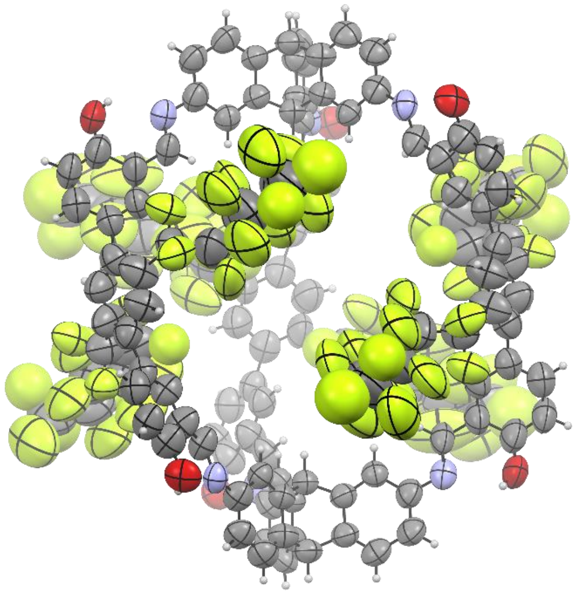

Table S5: Crystal data and structure refinement for **C_6F_{13}-cage**.

CCDC-number	2477449	
Empirical formula	$C_{136}H_{58}F_{78}N_6O_6$	
Formula weight	3353.88	
Temperature	200(2) K	
Wavelength	1.54178 Å	
Crystal system	trigonal	
Space group	R $\bar{3}$ c	
Z	6	
Unit cell dimensions	a = 20.0807(3) Å	α = 90 deg.
	b = 20.0807(3) Å	β = 90 deg.
	c = 66.2180(18) Å	γ = 120 deg.
Volume	23124.1(9) $Å^3$	
Density (calculated)	1.45 g/cm^3	
Absorption coefficient	1.40 mm^{-1}	
Crystal shape	prism	
Crystal size	0.170 x 0.080 x 0.077 mm^3	
Crystal colour	yellow	
Theta range for data collection	2.9 to 46.1 deg.	
Index ranges	$-18 \le h \le 15$, $-17 \le k \le 18$, $-40 \le l \le 61$	
Reflections collected	15069	
Independent reflections	2162 (R(int) = 0.0399)	
Observed reflections	1575 (I > 2σ(I))	
Absorption correction	Semi-empirical from equivalents	
Max. and min. transmission	0.96 and 0.46	
Refinement method	Full-matrix least-squares on F^2	
Data/restraints/parameters	2162 / 1812 / 513	
Goodness-of-fit on F^2	3.61	
Final R indices (I>2sigma(I))	R1 = 0.181, wR2 = 0.437	
Largest diff. peak and hole	0.42 and -0.39 $eÅ^{-3}$	

$(C_3F_8)_3{\subset}C_3F_7$-cage

Crystals of **$(C_3F_8)_3{\subset}C_3F_7$-cage** suitable for single crystal X-ray diffraction have been obtained by filtration of crystals of **C_3F_7-cage** obtained as described above, evaporation of the solvents under reduced pressure and the exposition of the evacuated crystals under 1 atm of C_3F_8 at room temperature overnight.

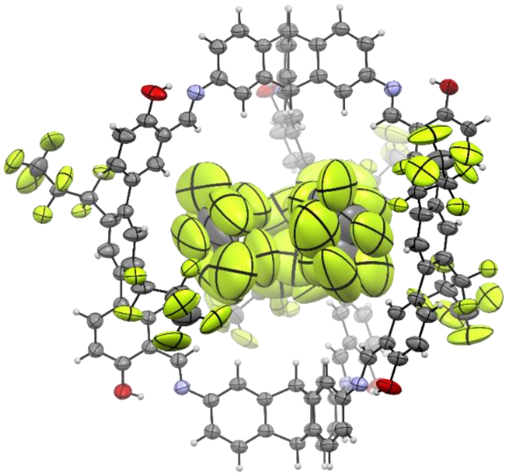

Table S6: Crystal data and structure refinement for $(C_3F_8)_3{\subset}C_3F_7$-cage.

CCDC-number	2477450	
Empirical formula	$C_{127}H_{58}F_{66}N_6O_6$	
Formula weight	3017.79	
Temperature	200(2) K	
Wavelength	1.54178 Å	
Crystal system	trigonal	
Space group	$R\bar{3}$	
Z	6	
Unit cell dimensions	a = 19.2816(3) Å	α = 90 deg.
	b = 19.2816(3) Å	β = 90 deg.
	c = 61.7128(16) Å	γ = 120 deg.
Volume	19869.7(8) $Å^3$	
Density (calculated)	1.51 g/cm^3	
Absorption coefficient	1.42 mm^{-1}	
Crystal shape	cuboid	
Crystal size	0.107 x 0.088 x 0.054 mm^3	
Crystal colour	yellow	
Theta range for data collection	2.7 to 65.1 deg.	
Index ranges	$-22 \le h \le 16$, $-16 \le k \le 22$, $-72 \le l \le 60$	
Reflections collected	24649	
Independent reflections	7401 (R(int) = 0.0443)	
Observed reflections	4490 ($I > 2\sigma(I)$)	
Absorption correction	Semi-empirical from equivalents	
Max. and min. transmission	0.95 and 0.77	
Refinement method	Full-matrix least-squares on F^2	
Data/restraints/parameters	7401 / 3137 / 840	
Goodness-of-fit on F^2	1.04	
Final R indices (I>2sigma(I))	R1 = 0.094, wR2 = 0.254	
Largest diff. peak and hole	0.53 and -0.39 $eÅ^{-3}$	

7. Isomorphism and Structural Details in the Solid State

Table S7. Selected crystallographic parameters proving the isomorphism of the obtained structures.

Parameter	**CF_3-cage**	**C_2F_5-cage**	**C_3F_7-cage**	**C_4F_9-cage[4]**	**C_5F_{11}-cage**	**C_6F_{13}-cage**
CDCC	2477445	2477446	2477447	2150481	2477448	2477449
Temperature [K]	200(2)	200(2)	200(2)	100(2)	200(2)	200(2)
crystal system	trigonal	trigonal	trigonal	trigonal	trigonal	trigonal
space group	$R\bar{3}c$	$R\bar{3}c$	$R\bar{3}c$	$R\bar{3}c$	$R\bar{3}c$	$R\bar{3}c$
Z	6	6	6	6	6	6
a [Å]	19.3976(2)	20.1547(4)	19.8267(1)	19.0156(4)	19.5342(4)	20.0807(3)
b [Å]	19.3976(2)	20.1547(4)	19.8267(1)	19.0156(4)	19.5342(4)	20.0807(3)
c [Å]	60.9238(14)	77.800(2)	62.2239(11)	64.026(2)	66.037(2)	66.2180(18)
α [°]	90	90	90	90	90	90
β [°]	90	90	90	90	90	90
γ [°]	120	120	120	120	120	120
cell volume [$Å^3$]	19852.4(6)	27369.3(14)	21183.1(5)	20049.6(11)	21822.8(12)	23124.1(9)
endo-population	-[a]	46%	-[b]	68%	69%[c]	57%
exo-population	-[a]	54%	100%[b]	32%	31%	43%

[a] Since the first CF_2-unit of the cages is always in-plane with the corresponding phenyl unit, by definition for **CF_3-cage** no *endo* or *exo* conformers can be defined. [b] For **C_3F_7-cage** only *exo*-pointing side-chain have been found with two different main orientations. [c] For **C_5F_{11}-cage** two slightly different *endo*-orientations were found.

The two main orientations of the side-chain are described as *endo*- as well as *exo*-conformers[S4] and their ratio for the corresponding cages is given in Table S7. Due to the crystallographic symmetry, it can not be differentiated whether this *endo*/*exo*-ratio of corresponds to all side-chains of cages being *endo* or *exo* or a mixture of both is actually present. Nevertheless, the alignments have no influence on the corresponding pore structures and thus only the *endo*-side-chain alignments as shown in our initial study[4] with exception of **C_3F_7-cage** where only *exo*-alignments are found. Due to crystallographic reasons, **C_3F_7-cage** is still isomorphous to the other cages of the series. **C_2F_5-cage** shows a slightly elongated crystallographic *c*-axis but PXRD-analyses show, that the crystalline lattice reorganises into a phase isomorphous to the other cages are discussed below.

8. Powder X-ray Diffraction (PXRD)

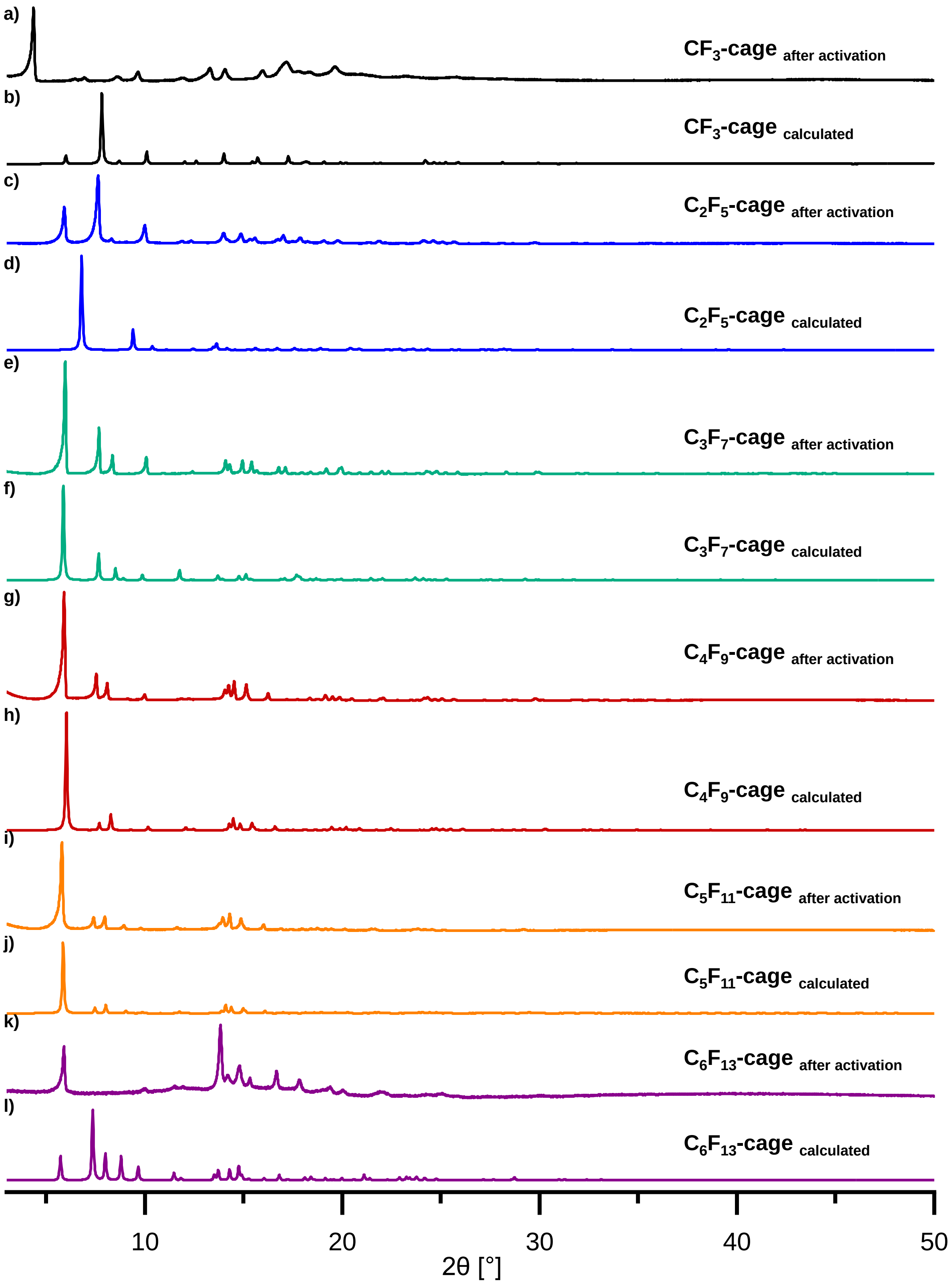

Figure S125. Powder X-ray diffraction patterns of **CF_3-** to **C_6F_{13}-cage** after thermal activation (a, c, e, g, il, k) and calculation from the single crystal X-ray diffraction data (b, d, f, h, j, l).

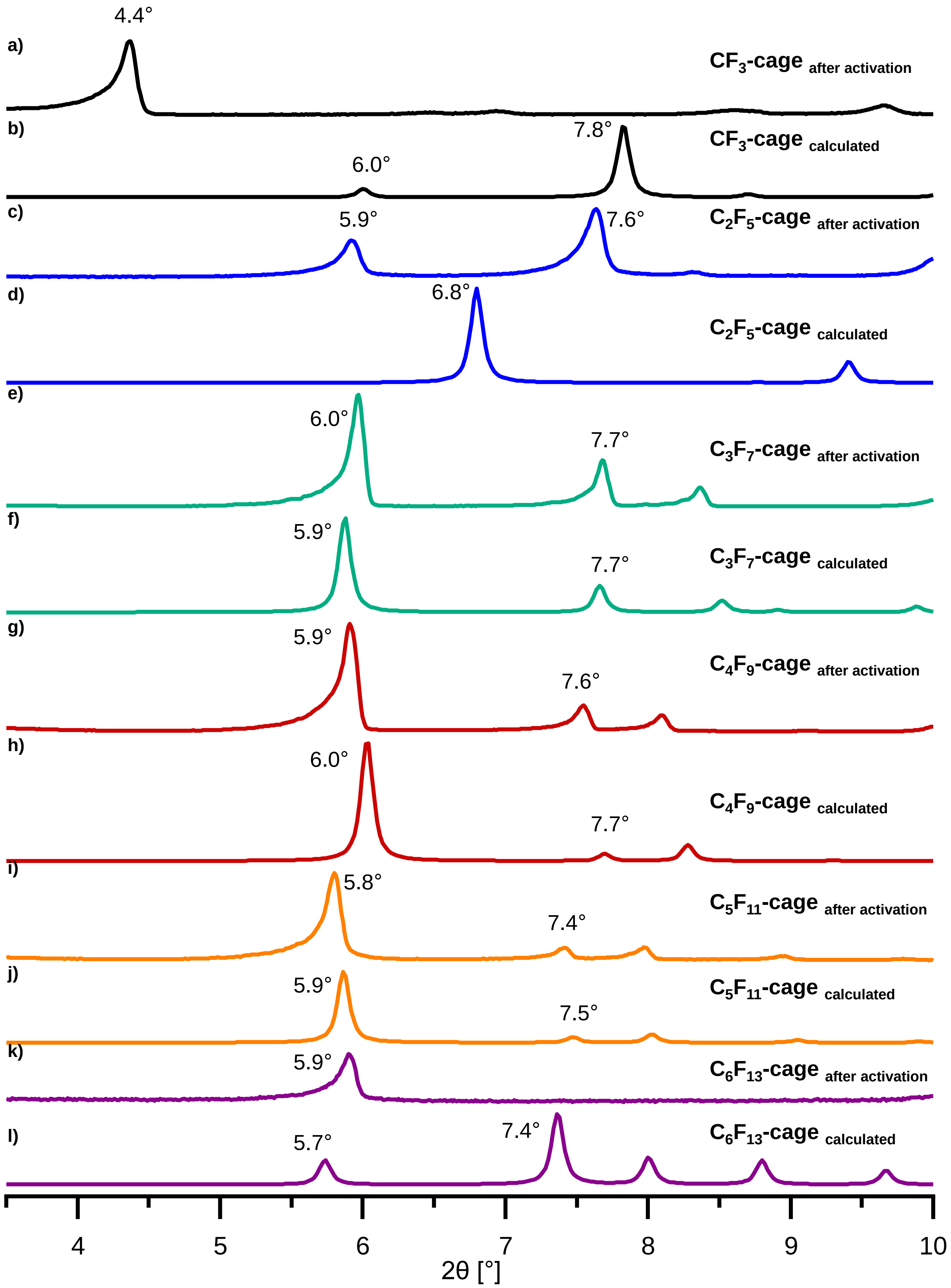

Figure S126. Zoom-ins into the powder X-ray diffraction patterns of **CF_3-** to **C_6F_{13}-cage** after thermal activation (a, c, e, g, il, k) and calculation from the single crystal X-ray diffraction data (b, d, f, h, j, l).

9. Gas Sorption

Gases, Methods, General Properties

Gases

Table S9. Gases used in this study.

Gas	Formula	CAS	Supplier	Quality
Nitrogen	N_2	7727-37-9	Air Liquide	5.0 (99.999%)
Argon	Ar	7440-37-1	Air Liquide	4.8 (99.998%)
Carbon dioxide	CO_2	124-38-9	Air Liquide	4.5 (99.995%)
Oxygen	O_2	7782-44-7	Air Liquide	4.8 (99.998%)
Perfluoromethane	CF_4	75-73-0	Air Liquide	4.5 (99.995%)
Perfluoroethane	C_2F_6	76-16-4	Air Liquide	4.8 (99.998%)
Perfluoropropane	C_3F_8	76-19-7	Air Liquide	3.7 (99.97%)
Perfluorocyclobutane	*c*-C_4F_8	115-25-3	Air Liquide	3.6 (99.96%)
Sulfur hexafluoride	SF_6	2551-62-4	Air Liquide	5.0 (99.999%)
Nitrogen trifluoride	NF_3	7783-54-2	Air Liquide	3.0 (99.9%)

Non-ideality factors

To express the deviation of the behaviour of a real gas from ideality, the non-ideality factor α was calculated and implemented in the parameter files of the operating software of the Quantachrome IQ2 and IQ3 systems. Two methods have been taken into account to calculate the non-ideality factors and the average of both values was used for the corresponding measurements.

<u>Method 1:</u> Berthelot-Equation[S8]

$$pV\,(1 + \alpha p) = n\mathrm{R}T$$

with

$$\alpha = \frac{9}{128}\,\frac{1}{p_c}\,\frac{T_\mathrm{c}}{T}\left(6\left(\frac{T_\mathrm{c}}{T}\right)^2 - 1\right)$$

α: non-ideality factor

p: pressure

p_c: critical pressure

V: volume

R: gas constant

T: temperature

T_c: critical temperature

Method 2: van der Waals Equation[S9]

$$\left(p + \frac{a}{V_m^{\ 2}}\right)(V_m - b) = RT$$

with

$$a = \frac{27\,(RT_c)^2}{64p_c}$$

and

$$b = \frac{RT_c}{8p_c}$$

a: van der Waals parameter (internal pressure)

b: van der Waals parameter (co-volume)

p: pressure

p_c: critical pressure

V_m: molar volume

R: gas constant

T: temperature

T_c: critical temperature

The co-volume **b** defines the deviation for ideal behaviour at high pressures. The van-der-Waals parameter **a** is equivalent to the non-ideality factor and taken into account exclusively.

Values for the critical pressure, critical temperature and boiling points have been extracted from the *NIST Chemistry WebBook, SRD 69* of the National Institute of Standards and Technology.[S10]

Table S10. Selected physical properties and non-ideality factors.

Gas	bp (K)	T (K)[a]	crit. T (K)	crit. P (Torr)	non-ideality ($\times 10^{-5} \cdot Torr^{-1}$)		
					Berthelot	vdW	average
N_2	77.35	77.35	126	25502	6.70	3.59	5.15
N_2	77.35	273.15	126	25502	0.04	0.13	0.08
N_2	77.35	283.15	126	25502	0.02	0.11	0.07
N_2	77.35	298.15	126	25502	0.01	0.09	0.05
N_2	77.35	313.15	126	25502	0.00	0.07	0.03
Ar	87.15	87.3	151	36753	0.27	0.37	0.32
CO_2	194.69[b]	195.15	304.2	55354	2.69	1.50	2.09
CO_2	194.69[b]	273.15	304.2	55354	0.91	0.69	0.80
CF_4	145.1	273.15	227.5	28090	0.66	0.67	0.67
CF_4	145.1	283.15	227.5	28090	0.58	0.61	0.59
CF_4	145.1	298.15	227.5	28090	0.48	0.54	0.51
CF_4	145.1	313.15	227.5	28090	0.39	0.47	0.43
C_2F_6	195.0	273.15	292.8	22817	1.95	1.54	1.74
C_2F_6	195.0	283.15	292.8	22817	1.73	1.41	1.57
C_2F_6	195.0	298.15	292.8	22817	1.45	1.25	1.35
C_2F_6	195.0	313.15	292.8	22817	1.23	1.11	1.17
C_3F_8	234.0	273.15	345.1	20064	3.80	2.57	3.19
C_3F_8	234.0	283.15	345.1	20064	3.38	2.36	2.87
C_3F_8	234.0	298.15	345.1	20064	2.86	2.10	2.48
C_3F_8	234.0	313.15	345.1	20064	2.43	1.87	2.15
c-C_4F_8	267.3	273.15	388.5	20882	5.34	3.24	4.29
c-C_4F_8	267.3	283.15	388.5	20882	4.76	2.98	3.87
c-C_4F_8	267.3	298.15	388.5	20882	4.04	2.65	3.34
c-C_4F_8	267.3	313.15	388.5	20882	3.44	2.37	2.91
SF_6	209.3	273.15	568.8	18677	19.65	8.41	14.03
SF_6	209.3	283.15	568.8	18677	17.58	7.78	12.68
SF_6	209.3	298.15	568.8	18677	14.99	6.95	10.97
SF_6	209.3	313.15	568.8	18677	12.87	6.24	9.56
NF_3	144.1	273.15	233.8	33458	0.61	0.6	0.61
NF_3	144.1	283.15	233.8	33458	0.54	0.55	0.54
NF_3	144.1	298.15	233.8	33458	0.44	0.48	0.46
NF_3	144.1	313.15	233.8	33458	0.37	0.42	0.4

[a] Measurement temperature. [b] Sublimation point.

Selectivity Calculations

The adsorption behaviour of two gases in comparison was investigated using two different selectivity models. The ideal adsorbed solution theory (IAST)[S11] considers the spreading pressure and is thus taking the competing character of two gases for the selective binding sites into account. The IAST is defined by a series of equations which have to be solved numerically. The numerical solution was accomplished using 3P instruments' 3p sim software (version 1.1.0.9).

The experimental isotherms possessing a type I like behaviour have been fitted with the non-linear Tóth equation:

$$q_{eq} = q_{max} \frac{K \cdot p}{(1 + (K \cdot p)^{t})^{\frac{1}{t}}}$$

with: *p*: pressure

q_{eq}: experimental uptake (mmol·g^{-1})

q_{max}: maximum uptake (mmol·g^{-1})

K: affinity constant (1·bar^{-1})

t: heterogeneity (or Tóth) parameter (dimensionless)

Gases with lower adsorption tendencies differ from a typical type I like behaviour, therefore other isotherm models had to be used for these. Isotherms with a non-linear but non-pronounced type I like behaviour have been fitted using the non-linear Tóth equation with $t = 1$, which is known as Langmuir adsorption isotherm:

$$q_{eq} = q_{max} \frac{K \cdot p}{1 + (K \cdot p)}$$

with: *p*: pressure

q_{eq}: experimental uptake (mmol·g^{-1})

q_{max}: maximum uptake (mmol·g^{-1})

K: affinity constant (1·bar^{-1})

Linear isotherms can be thus interpreted as Langmuir isotherms with $q_{max} \rightarrow \infty$, which transfers the Langmuir isotherm to a standard Henry isotherm:

$$q_{eq} = K \cdot p$$

with: *p*: pressure

q_{eq}: experimental uptake ($mmol \cdot g^{-1}$)

K: affinity constant ($1 \cdot bar^{-1}$)

For this purpose, the max. uptake was set to 1000 $mmol \cdot g^{-1}$

The parameters obtained by the described fittings where then applied in the IAST-theory to calculate the selectivity $S_{A/B}$ of gas A over gas B by:

$$S_{A/B} = \frac{x_A/y_A}{x_B/y_B}$$

with: x_i: molar fraction of compound i in the adsorbed phase

Y_i: molar fraction of compound i in the gas phase

For pressures of $p \rightarrow 0$, the IAST-selectivity can be seen as a frontier selectivity which is commonly known as Henry selectivity with the simplified Tóth equation:

$$q_{eq} = q_{max} \cdot K \cdot p$$

By the definition of Henry's law, this means the Henry constant K_H is now defined as:

$$K_H = q_{max} \cdot K$$

And the Henry selectivity S_H of a gas A over gas B can be calculated as the ratio of the corresponding Henry constants.

While the IAST-selectivity is considering the competition of two gases and thus gives insights in the bulk behaviour of an adsorbent, the Henry selectivity is only taking the most selective binding sites into account.

N_2-Sorption at 77 K

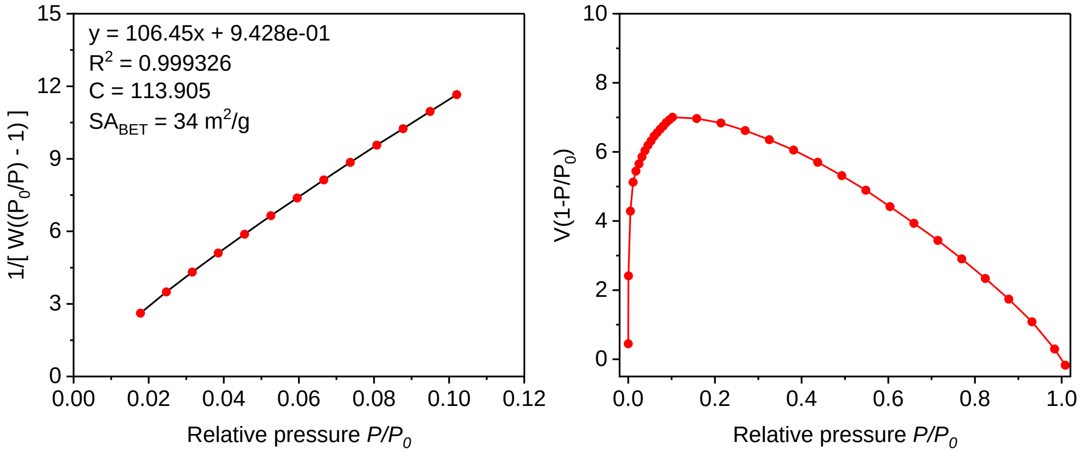

Figure S127. BET-plot (left) and corresponding Rouquerol-plot (right) of **CF_3-cage** derived from a nitrogen sorption isotherm at 77 K.

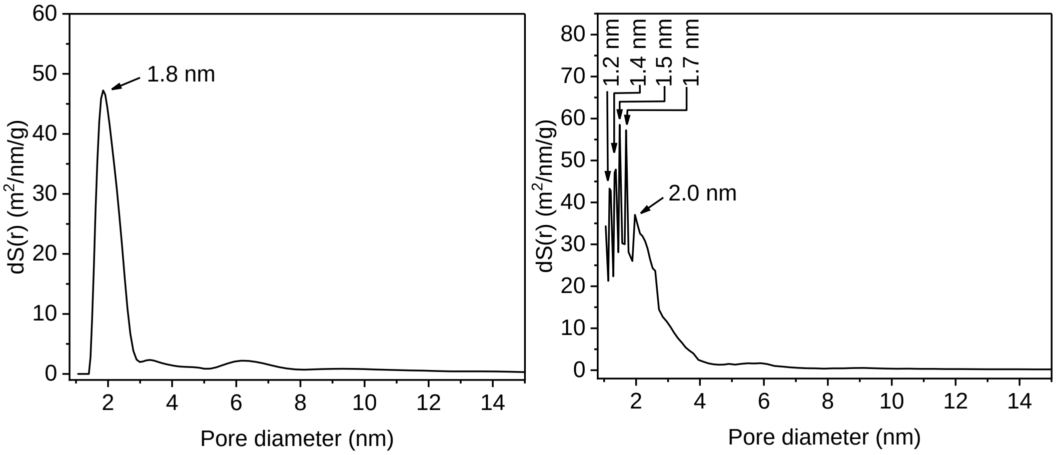

Figure S128. QSDFT pore size distribution (left) (cylindr./spher. pores on carbon, adsorption branch, fitting error: 1.036%) and NLDFT pore size distribution (right) (cylindr. pores on carbon, equilibrium model, fitting error: 4.109%) of **CF_3-cage** derived from a nitrogen sorption isotherm at 77 K.

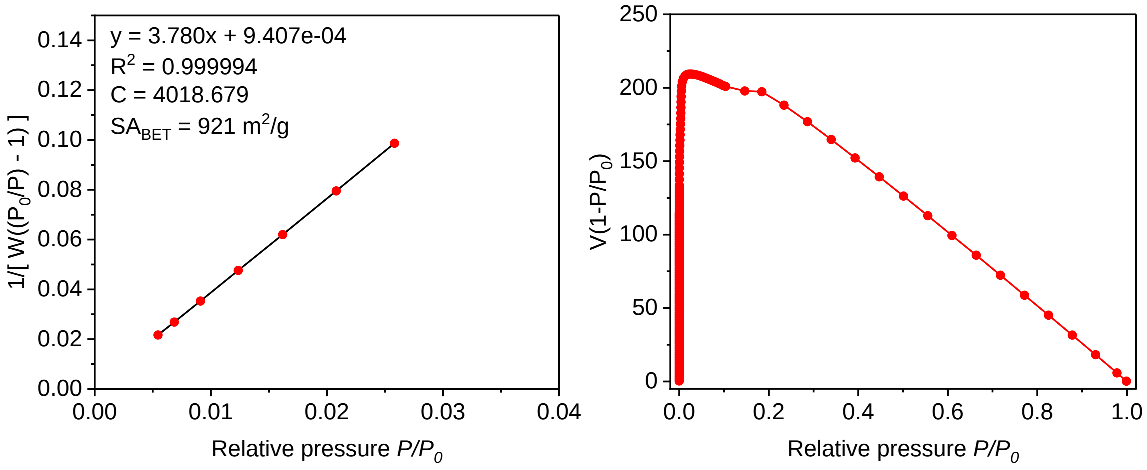

Figure S129. BET-plot (left) and corresponding Rouquerol-plot (right) of **C_2F_5-cage** derived from a nitrogen sorption isotherm at 77 K.

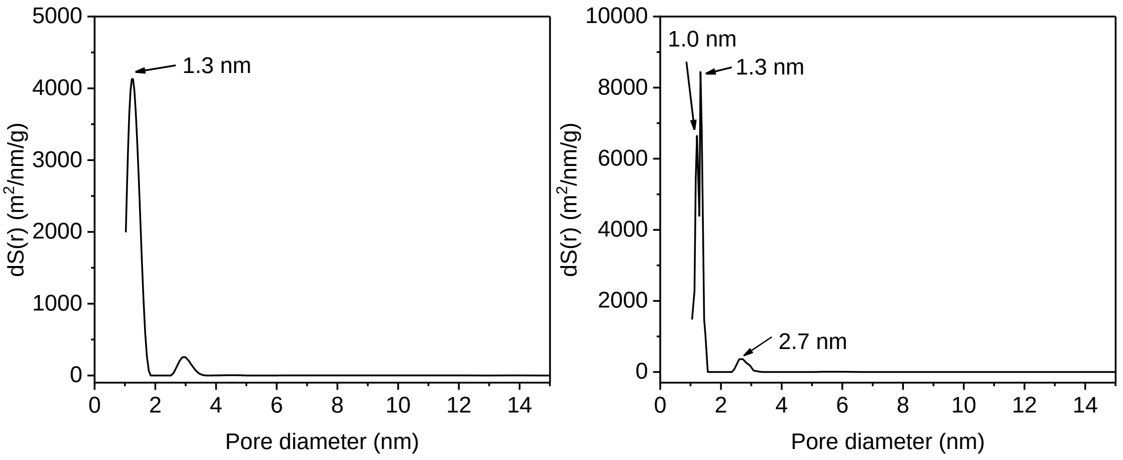

Figure S130. QSDFT pore size distribution (left) (cylindr./spher. pores on carbon, adsorption branch, fitting error: 1.243%) and NLDFT pore size distribution (right) (cylindr. pores on carbon, equilibrium model, fitting error: 0.882%) of **C_2F_5-cage** derived from a nitrogen sorption isotherm at 77 K.

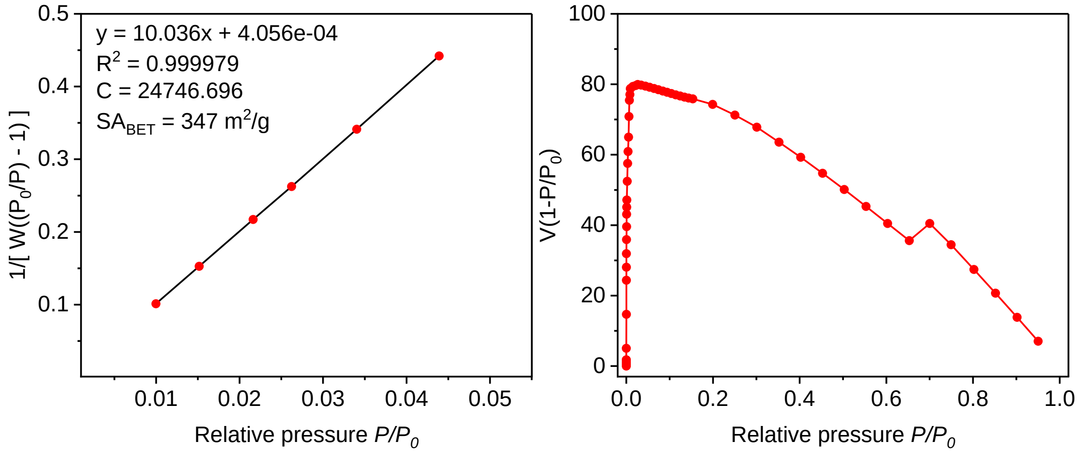

Figure S131. BET-plot (left) and corresponding Rouquerol-plot (right) of **C_3F_7-cage** derived from a nitrogen sorption isotherm at 77 K.

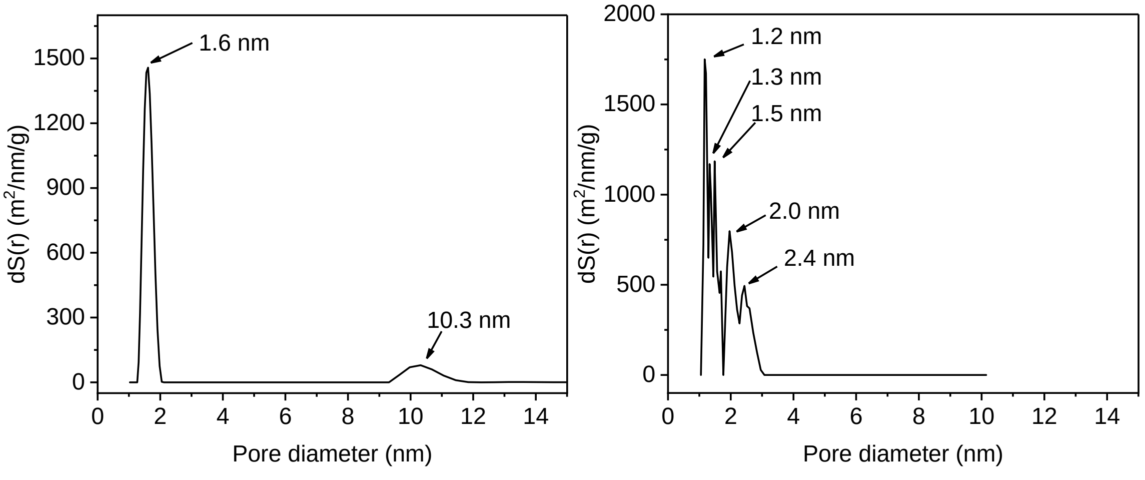

Figure S132. QSDFT pore size distribution (left) (cylindr./spher. pores on carbon, adsorption branch, fitting error: 3.556%) and NLDFT pore size distribution (right) (cylindr. pores on carbon, equilibrium model, fitting error: 6.777%) of **C_3F_7-cage** derived from a nitrogen sorption isotherm at 77 K.

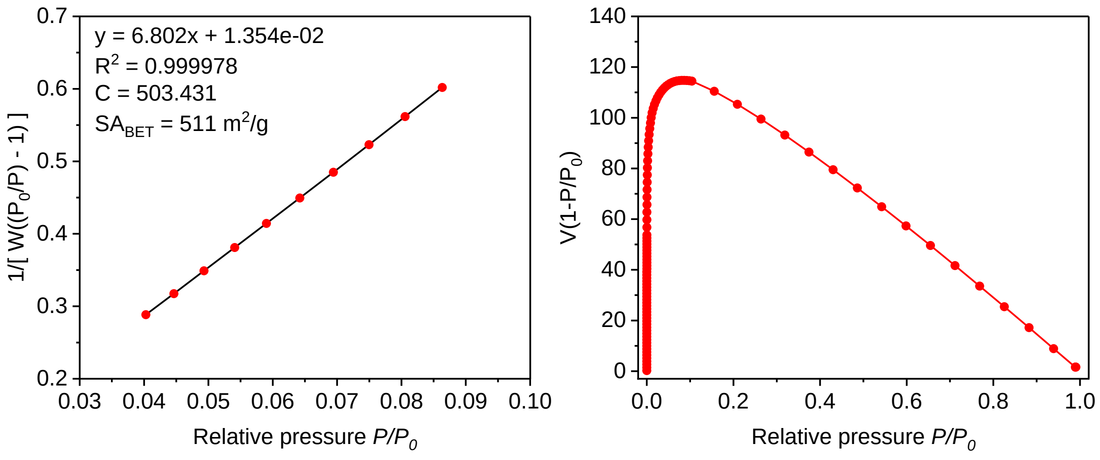

Figure S133. BET-plot (left) and corresponding Rouquerol-plot (right) of **C_5F_{11}-cage** derived from a nitrogen sorption isotherm at 77 K.

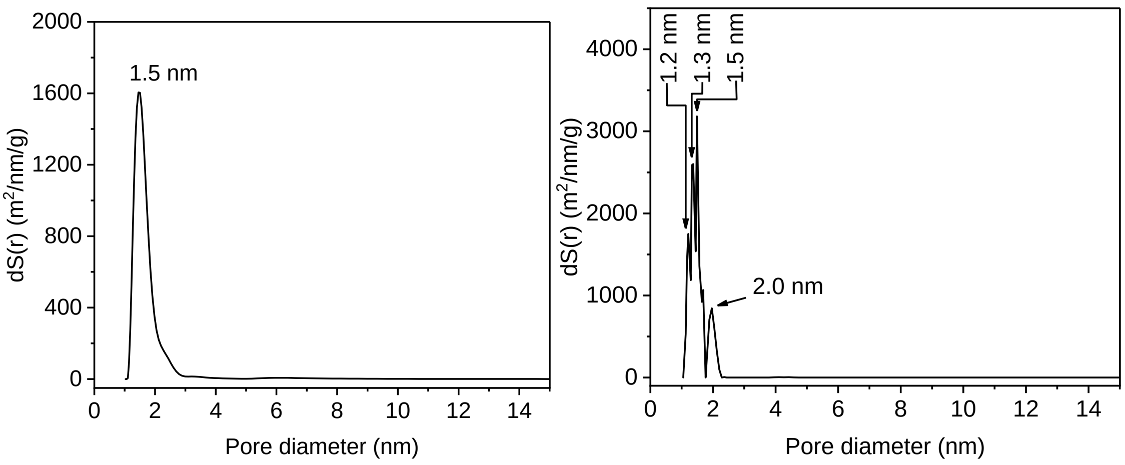

Figure S134. QSDFT pore size distribution (left) (cylindr./spher. pores on carbon, adsorption branch, fitting error: 1.964%) and NLDFT pore size distribution (right) (cylindr. pores on carbon, equilibrium model, fitting error: 1.495%) of **C_5F_{11}-cage** derived from a nitrogen sorption isotherm at 77 K.

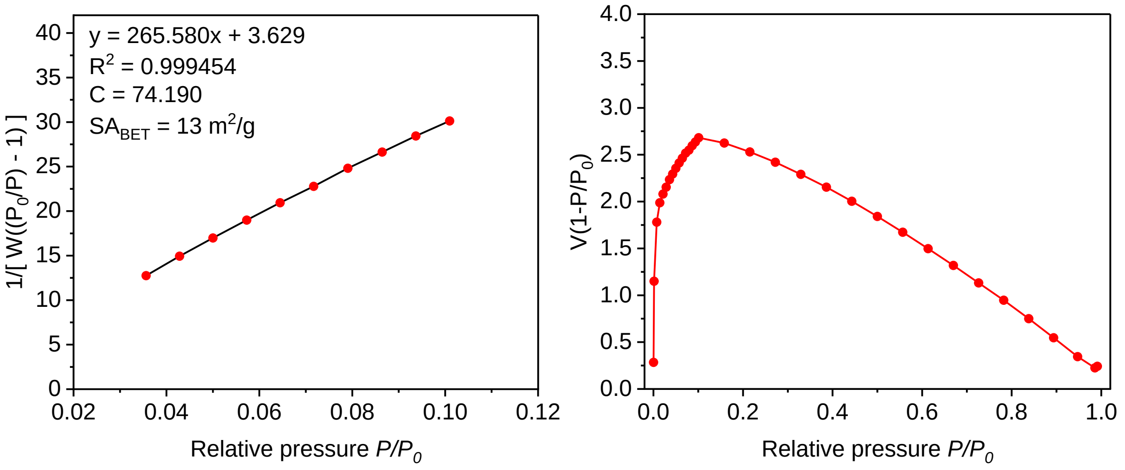

Figure S135. BET-plot (left) and corresponding Rouquerol-plot (right) of **C_6F_{13}-cage** derived from a nitrogen sorption isotherm at 77 K.

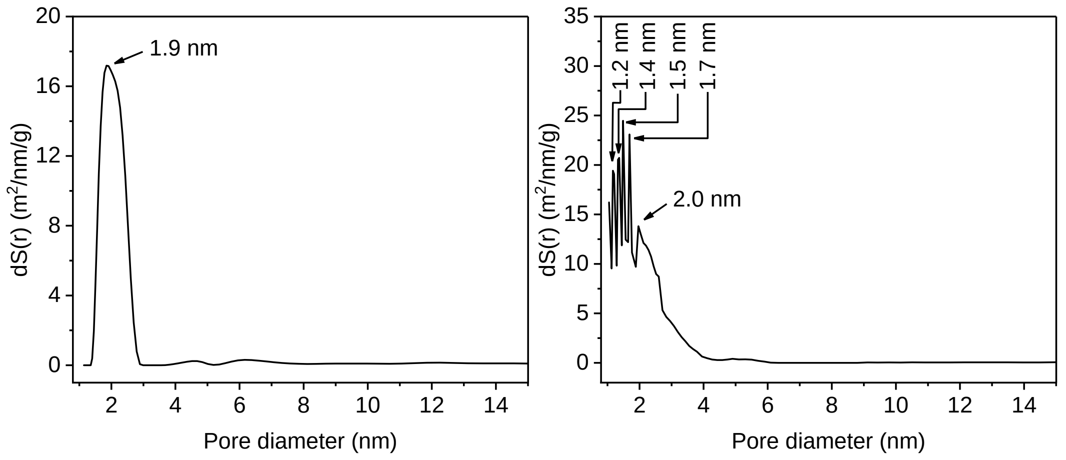

Figure S136. QSDFT pore size distribution (left) (cylindr./spher. pores on carbon, adsorption branch, fitting error: 5.246%) and NLDFT pore size distribution (right) (cylindr. pores on carbon, equilibrium model, fitting error: 6.776%) of **C_3F_7-cage** derived from a nitrogen sorption isotherm at 77 K.

Ar-Sorption at 87 K

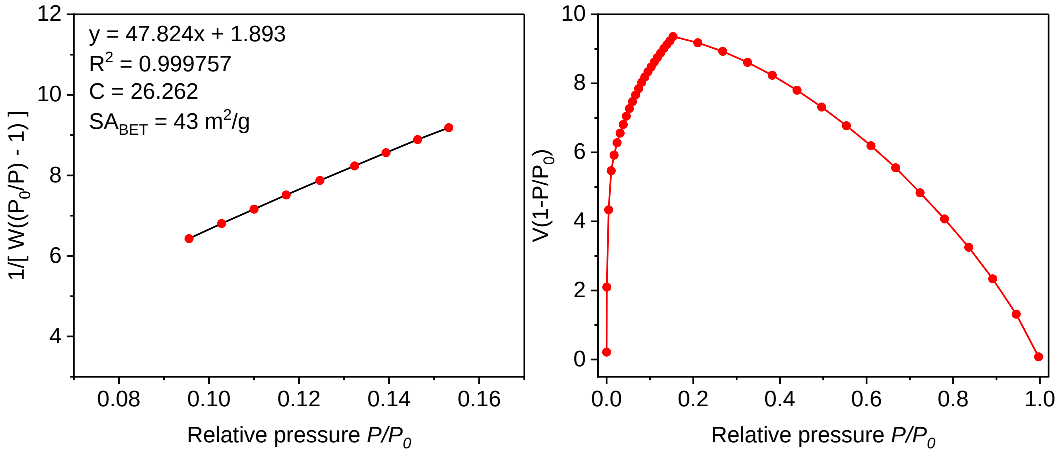

Figure S137. BET-plot (left) and corresponding Rouquerol-plot (right) of **CF_3-cage** derived from an argon sorption isotherm at 87 K.

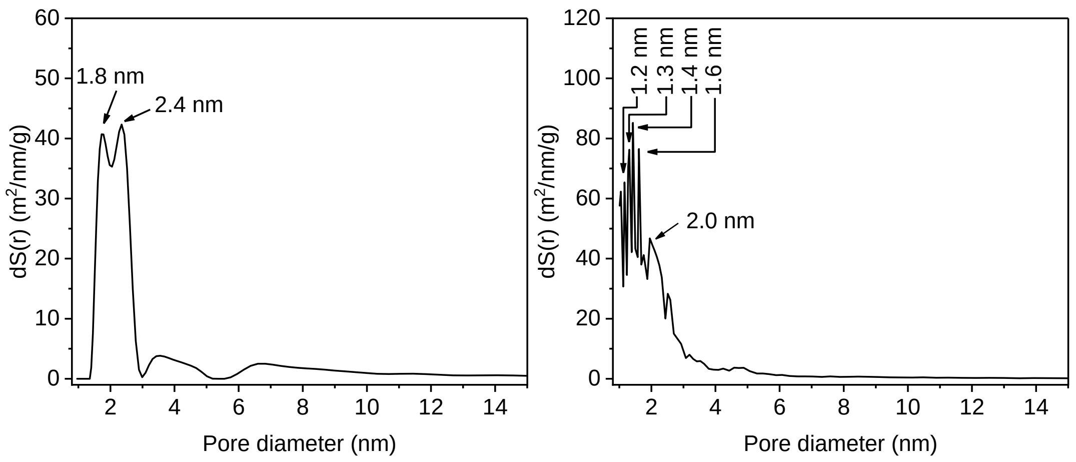

Figure S138. QSDFT pore size distribution (left) (cylindr./spher. pores on carbon, adsorption branch, fitting error: 0.730%) and NLDFT pore size distribution (right) (cylindr. pores on carbon, equilibrium model, fitting error: 3.816%) of **CF_3-cage** derived from an argon sorption isotherm at 87 K.

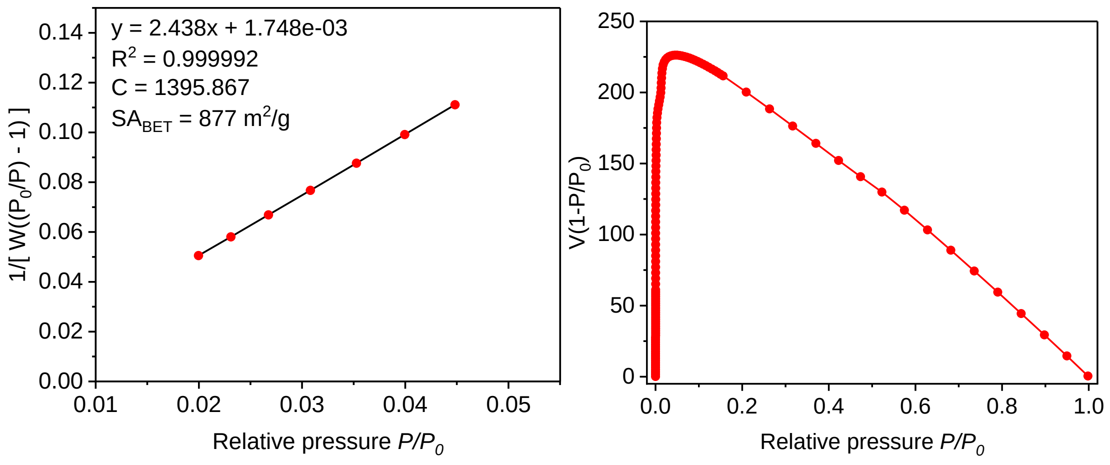

Figure S139. BET-plot (left) and corresponding Rouquerol-plot (right) of **C_2F_5-cage** derived from an argon sorption isotherm at 87 K.

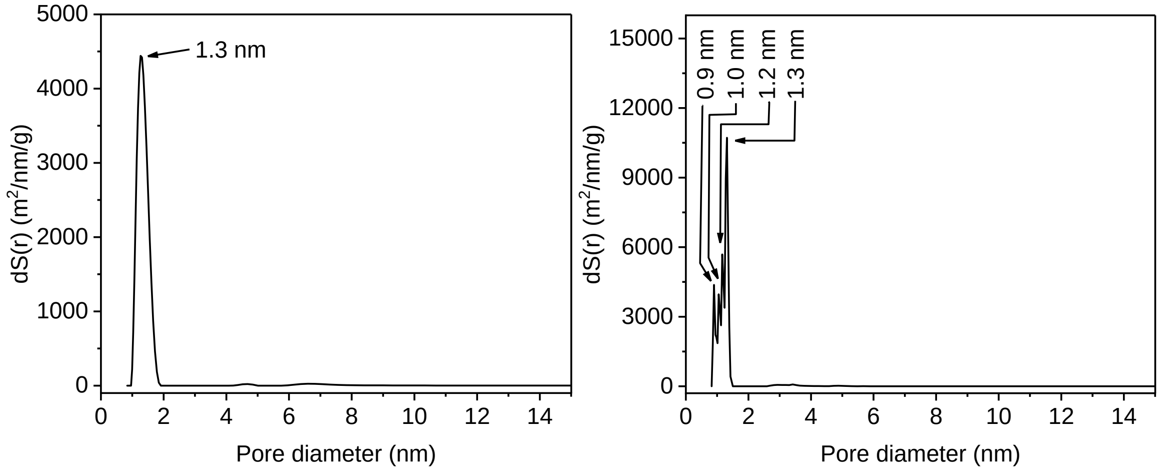

Figure S140. QSDFT pore size distribution (left) (cylindr./spher. pores on carbon, adsorption branch, fitting error: 1.772%) and NLDFT pore size distribution (right) (cylindr. pores on carbon, equilibrium model, fitting error: 0.354%) of **C_2F_5-cage** derived from an argon sorption isotherm at 87 K.

.

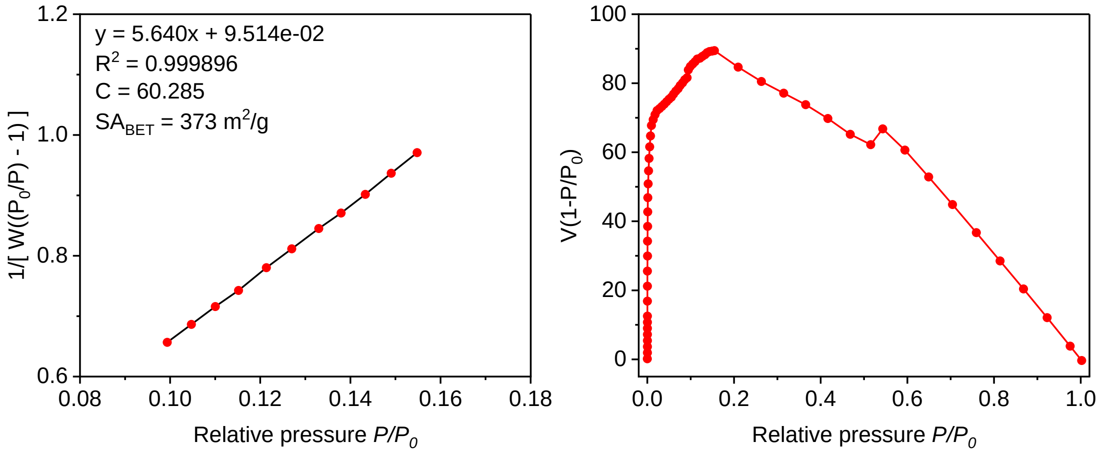

Figure S141. BET-plot (left) and corresponding Rouquerol-plot (right) of **C_3F_7-cage** derived from an argon sorption isotherm at 87 K.

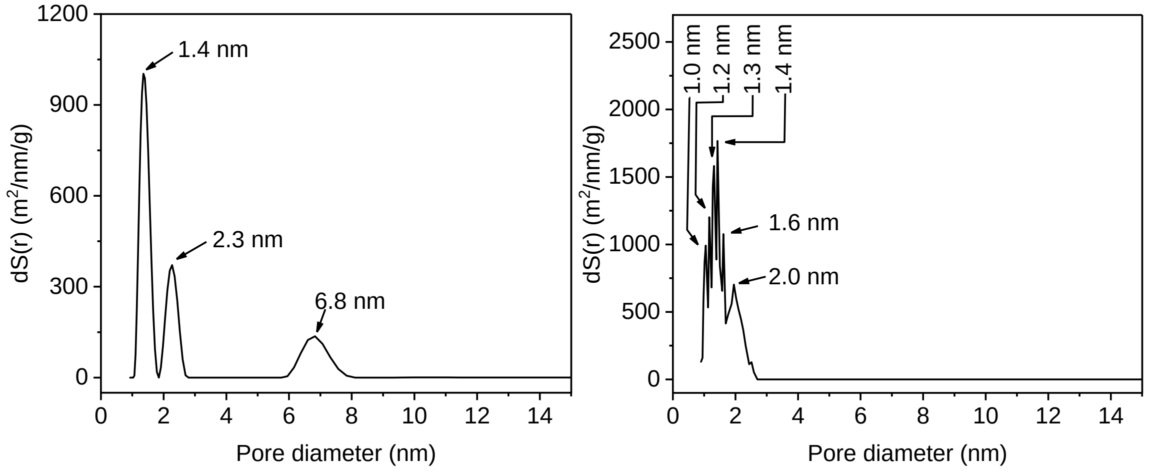

Figure S142. QSDFT pore size distribution (left) (cylindr./spher. pores on carbon, adsorption branch, fitting error: 1.591%) and NLDFT pore size distribution (right) (cylindr. pores on carbon, equilibrium model, fitting error: 3.542%) of **C_3F_7-cage** derived from an argon sorption isotherm at 87 K.

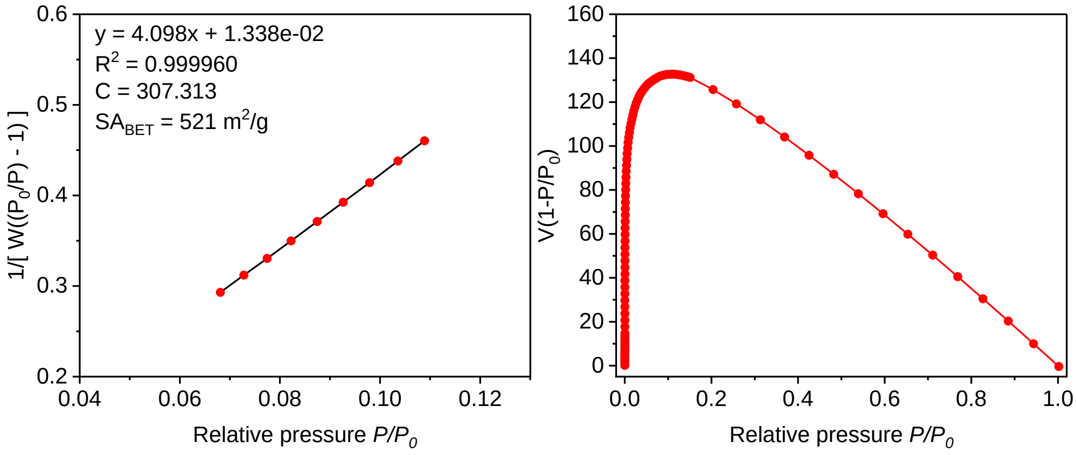

Figure S143. BET-plot (left) and corresponding Rouquerol-plot (right) of **C_5F_{11}-cage** derived from an argon sorption isotherm at 87 K.

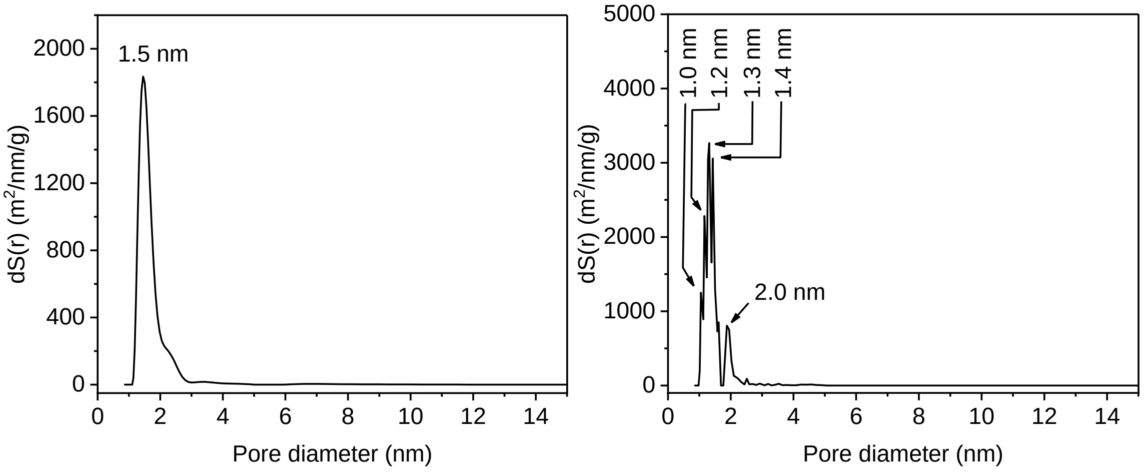

Figure S144. QSDFT pore size distribution (left) (cylindr./spher. pores on carbon, adsorption branch, fitting error: 1.886%) and NLDFT pore size distribution (right) (cylindr. pores on carbon, equilibrium model, fitting error: 1.124%) of **C_5F_{11}-cage** derived from an argon sorption isotherm at 87 K.

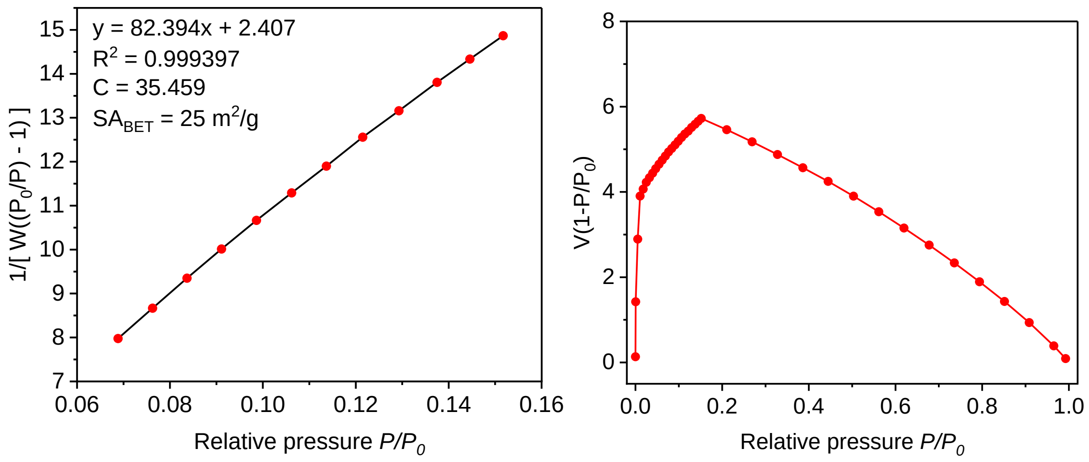

Figure S145. BET-plot (left) and corresponding Rouquerol-plot (right) of **C_6F_{13}-cage** derived from an argon sorption isotherm at 87 K.

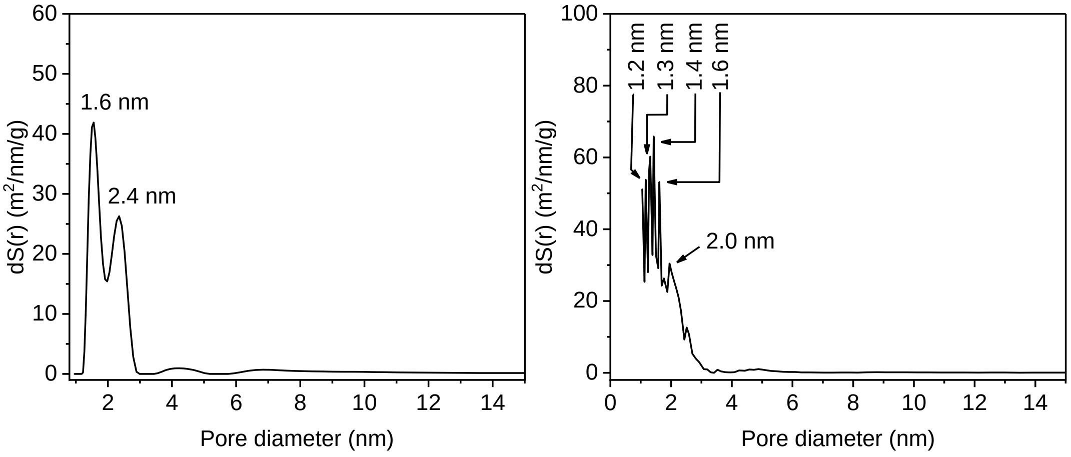

Figure S146. QSDFT pore size distribution (left) (cylindr./spher. pores on carbon, adsorption branch, fitting error: 1.035%) and NLDFT pore size distribution (right) (cylindr. pores on carbon, equilibrium model, fitting error: 6.106%) of **C_6F_{13}-cage** derived from an argon sorption isotherm at 87 K.

CO_2-Sorption at 195 K

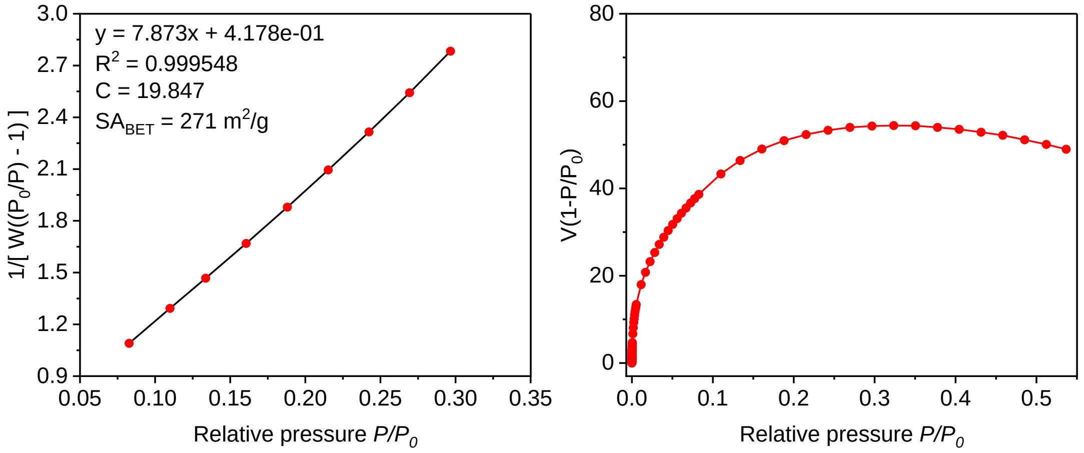

Figure S147. BET-plot (left) and corresponding Rouquerol-plot (right) of **CF_3-cage** derived from a carbon dioxide sorption isotherm at 195 K.

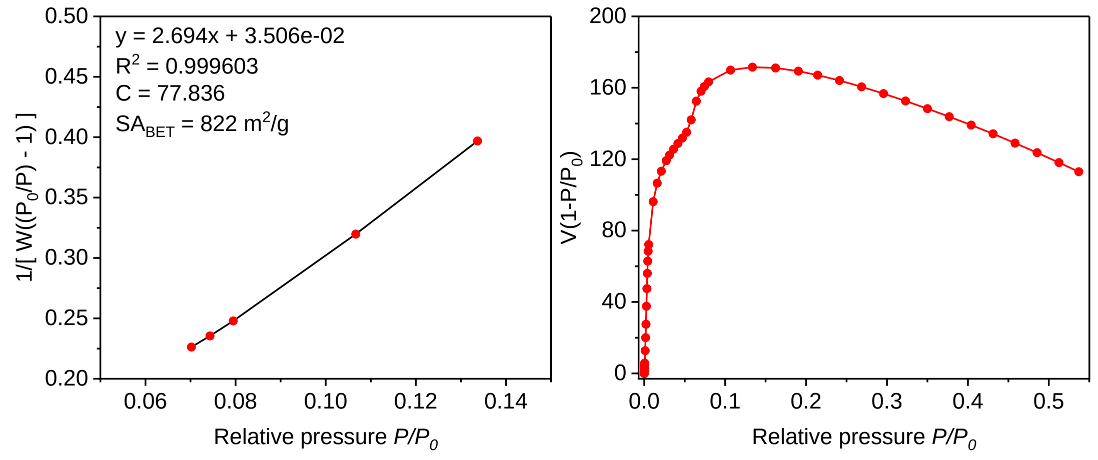

Figure S148. BET-plot (left) and corresponding Rouquerol-plot (right) of **C_2F_5-cage** derived from a carbon dioxide sorption isotherm at 195 K.

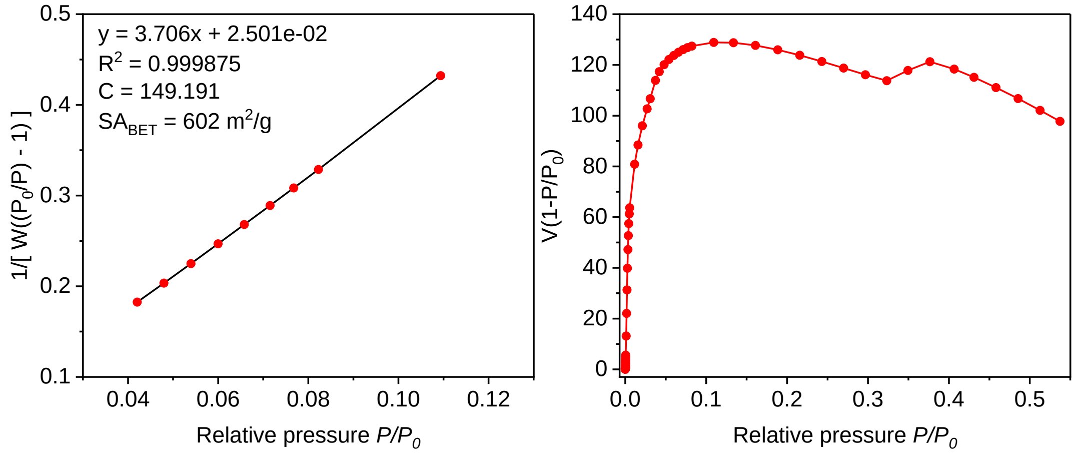

Figure S149. BET-plot (left) and corresponding Rouquerol-plot (right) of **C_3F_7-cage** derived from a carbon dioxide sorption isotherm at 195 K.

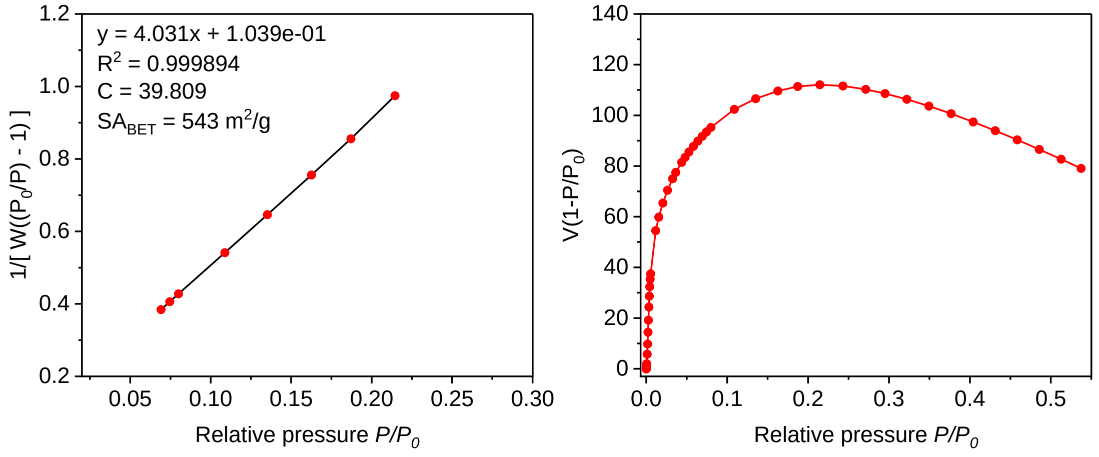

Figure S150. BET-plot (left) and corresponding Rouquerol-plot (right) of **C_5F_{11}-cage** derived from a carbon dioxide sorption isotherm at 195 K.

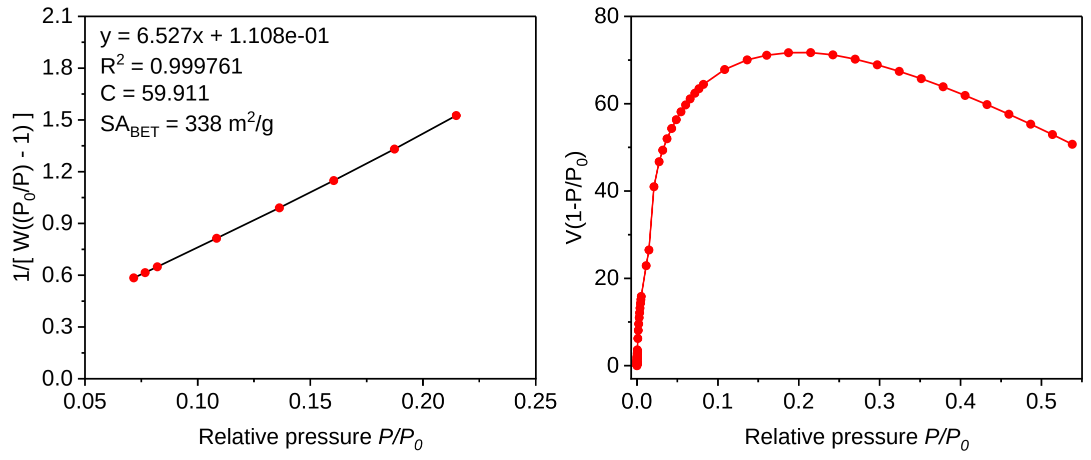

Figure S151. BET-plot (left) and corresponding Rouquerol-plot (right) of **C_6F_{13}-cage** derived from a carbon dioxide sorption isotherm at 195 K.

Gas Sorption at 273 K

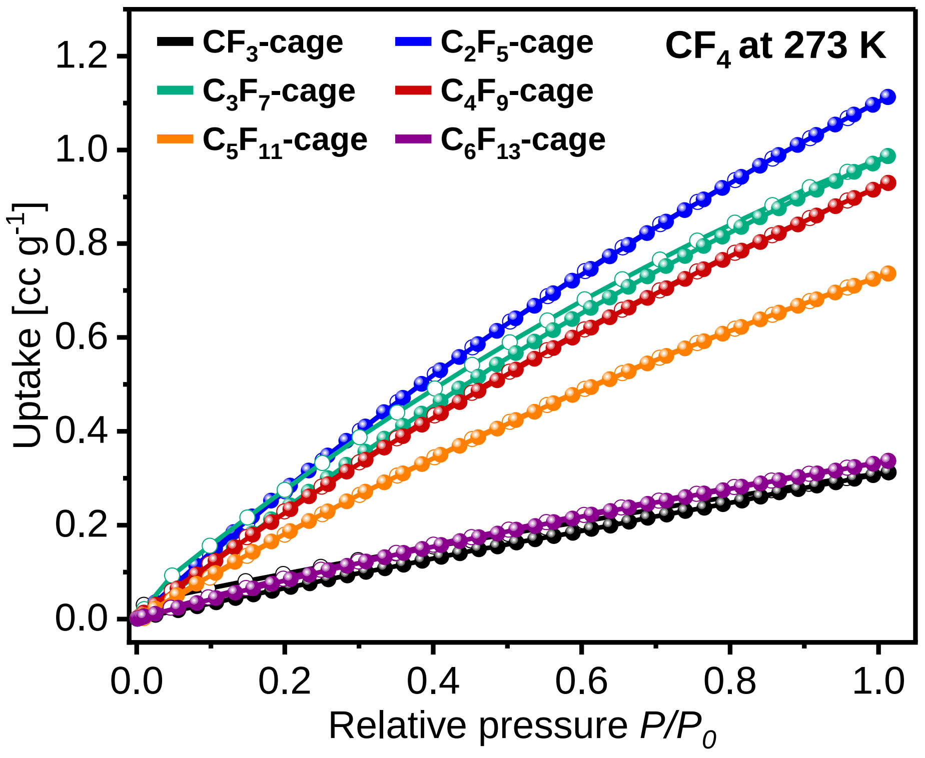

Figure S152. CF_4 sorption isotherms at 273 K. Black: **CF_3-cage**; blue: **C_2F_5-cage**; green: **C_3F_7-cage**; red: **C_4F_8-cage**; orange: **C_5F_{11}-cage**; purple: **C_6F_{13}-cage**. Full circles: adsorption; empty circles: desorption.

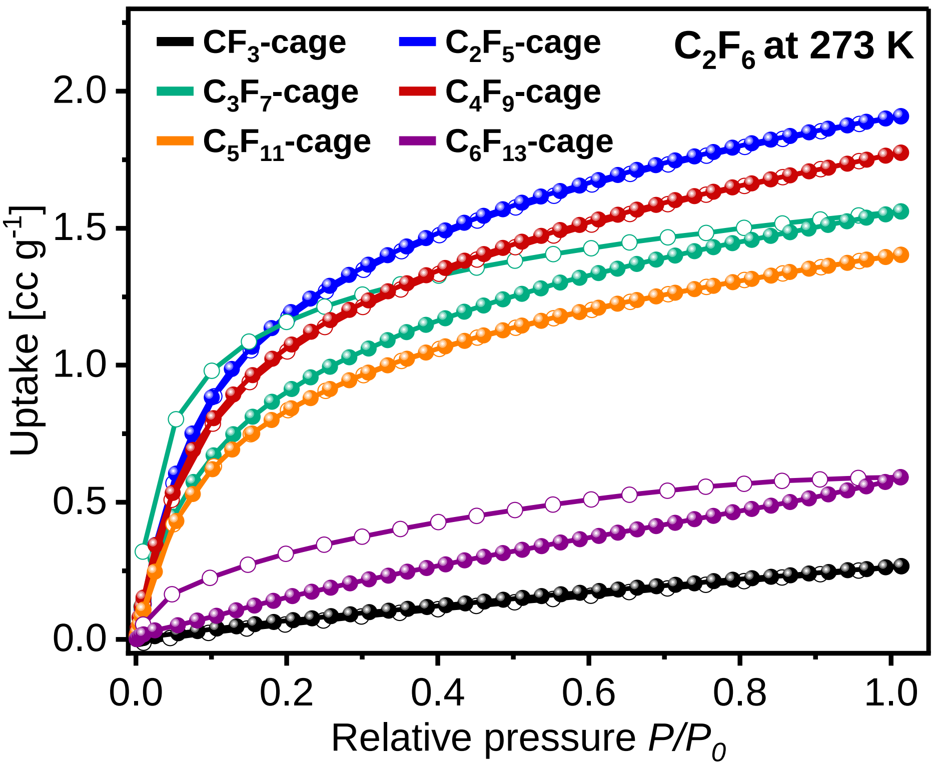

Figure S153. C_2F_6 sorption isotherms at 273 K. Black: **CF_3-cage**; blue: **C_2F_5-cage**; green: **C_3F_7-cage**; red: **C_4F_8-cage**; orange: **C_5F_{11}-cage**; purple: **C_6F_{13}-cage**. Full circles: adsorption; empty circles: desorption.

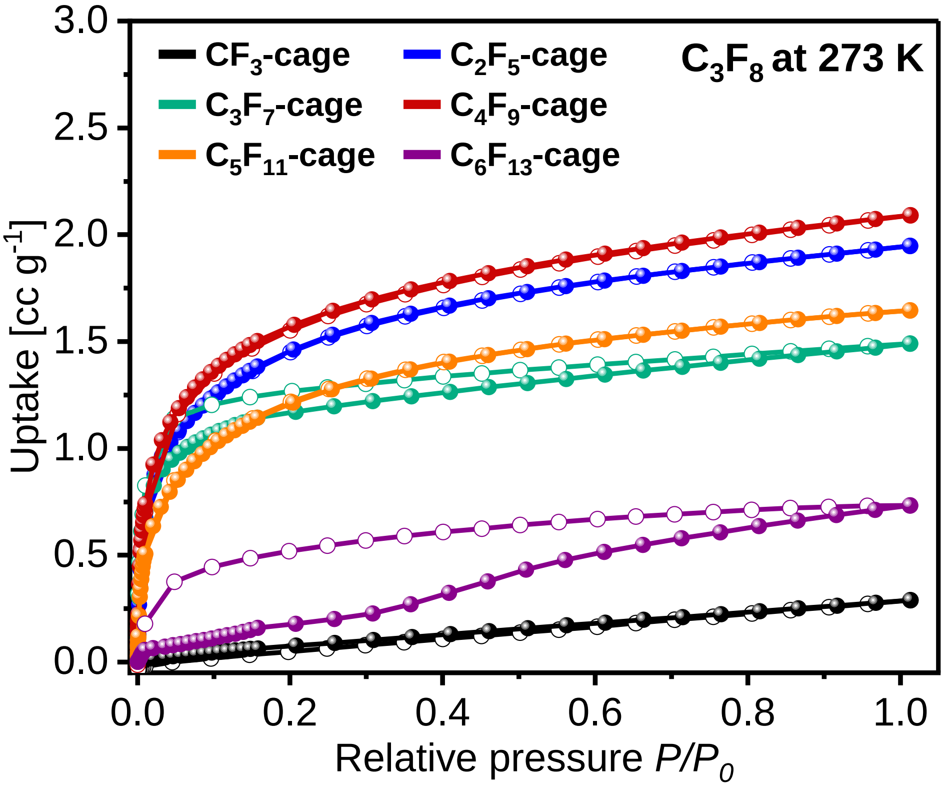

Figure S154. C_3F_8 sorption isotherms at 273 K. Black: **CF_3-cage**; blue: **C_2F_5-cage**; green: **C_3F_7-cage**; red: **C_4F_8-cage**; orange: **C_5F_{11}-cage**; purple: **C_6F_{13}-cage**. Full circles: adsorption; empty circles: desorption.

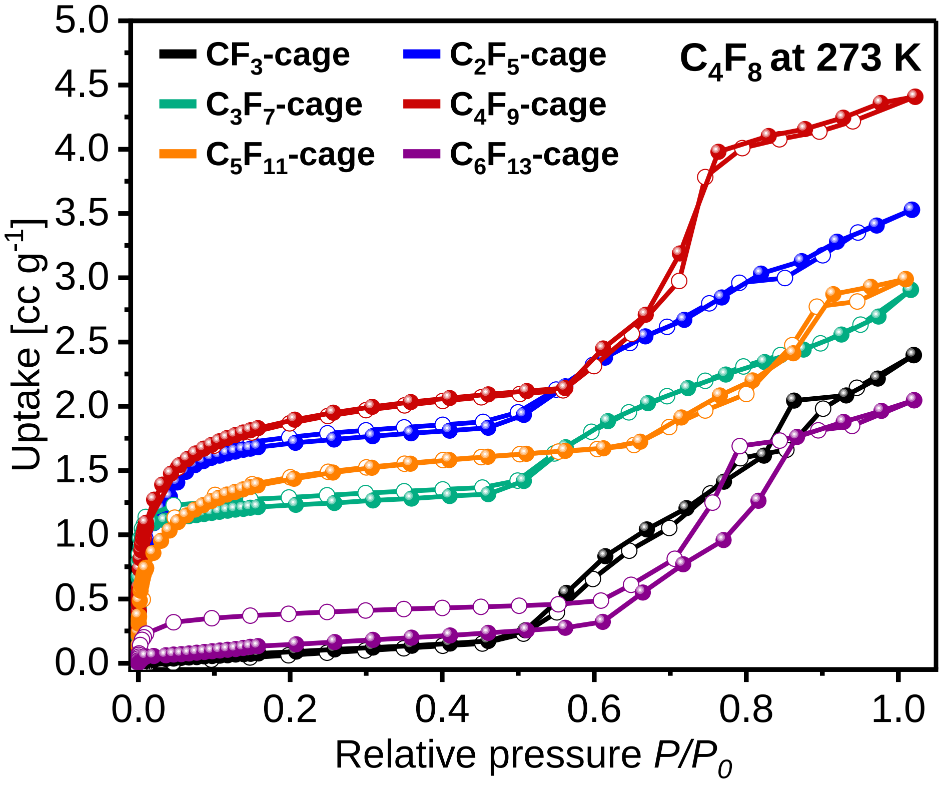

Figure S155. C_4F_8 sorption isotherms at 273 K. Black: **CF_3-cage**; blue: **C_2F_5-cage**; green: **C_3F_7-cage**; red: **C_4F_8-cage**; orange: **C_5F_{11}-cage**; purple: **C_6F_{13}-cage**. Full circles: adsorption; empty circles: desorption.

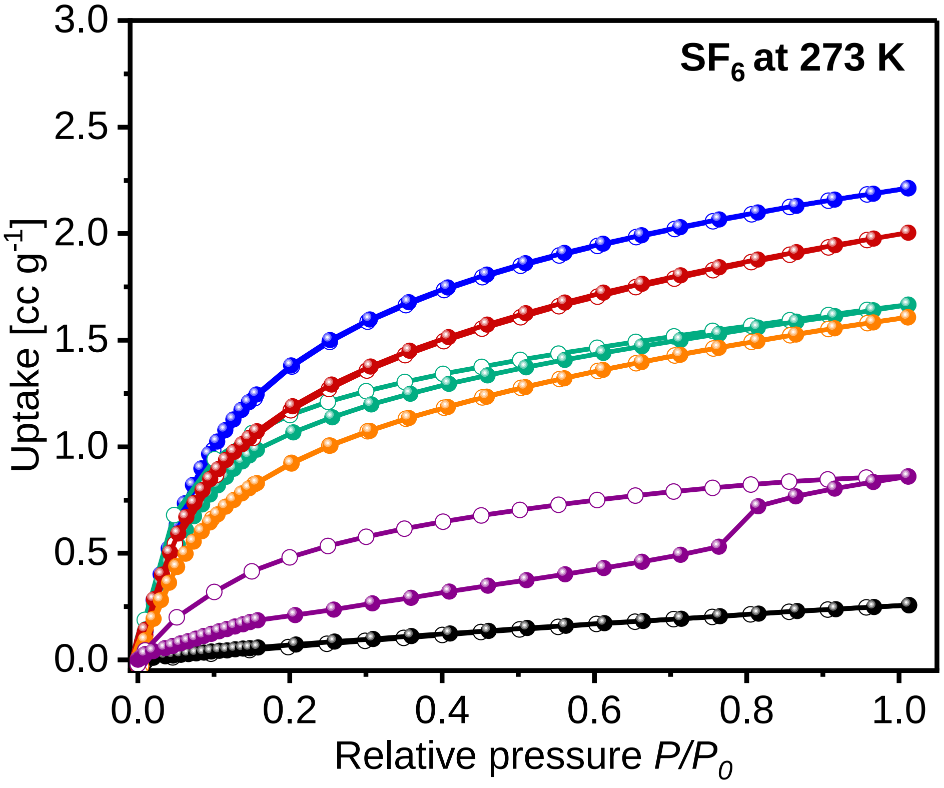

Figure S156. SF_6 sorption isotherms at 273 K. Black: **CF_3-cage**; blue: **C_2F_5-cage**; green: **C_3F_7-cage**; red: **C_4F_8-cage**; orange: **C_5F_{11}-cage**; purple: **C_6F_{13}-cage**. Full circles: adsorption; empty circles: desorption.

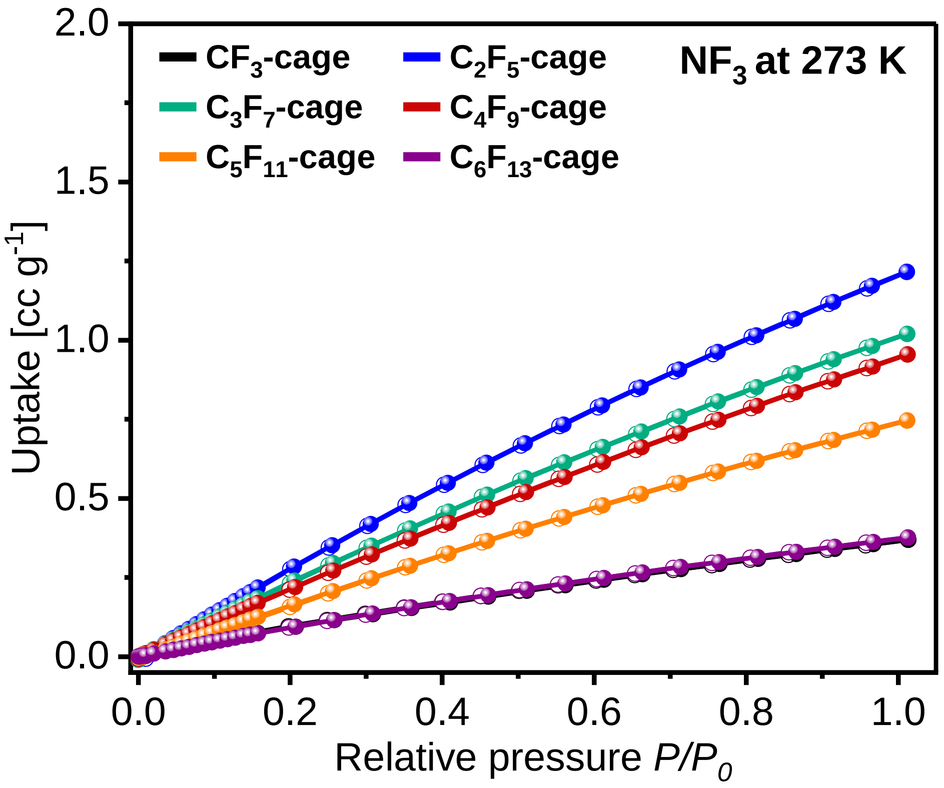

Figure S157. NF_3 sorption isotherms at 273 K. Black: **CF_3-cage**; blue: **C_2F_5-cage**; green: **C_3F_7-cage**; red: **C_4F_8-cage**; orange: **C_5F_{11}-cage**; purple: **C_6F_{13}-cage**. Full circles: adsorption; empty circles: desorption.

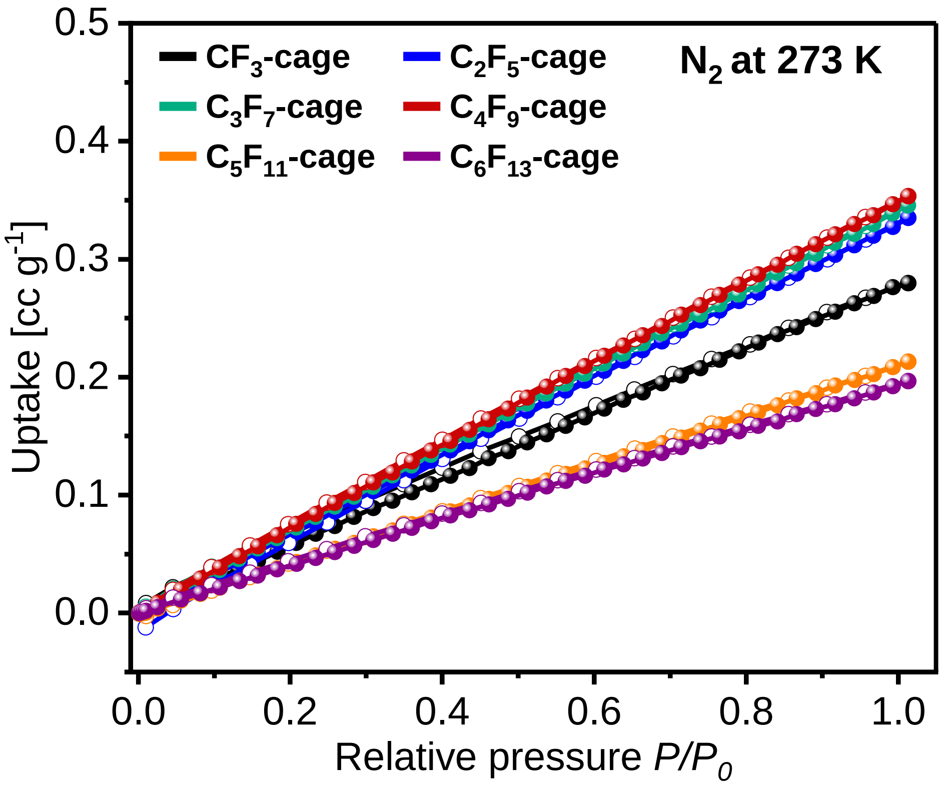

Figure S158. N_2 sorption isotherms at 273 K. Black: **CF_3-cage**; blue: **C_2F_5-cage**; green: **C_3F_7-cage**; red: **C_4F_8-cage**; orange: **C_5F_{11}-cage**; purple: **C_6F_{13}-cage**. Full circles: adsorption; empty circles: desorption.

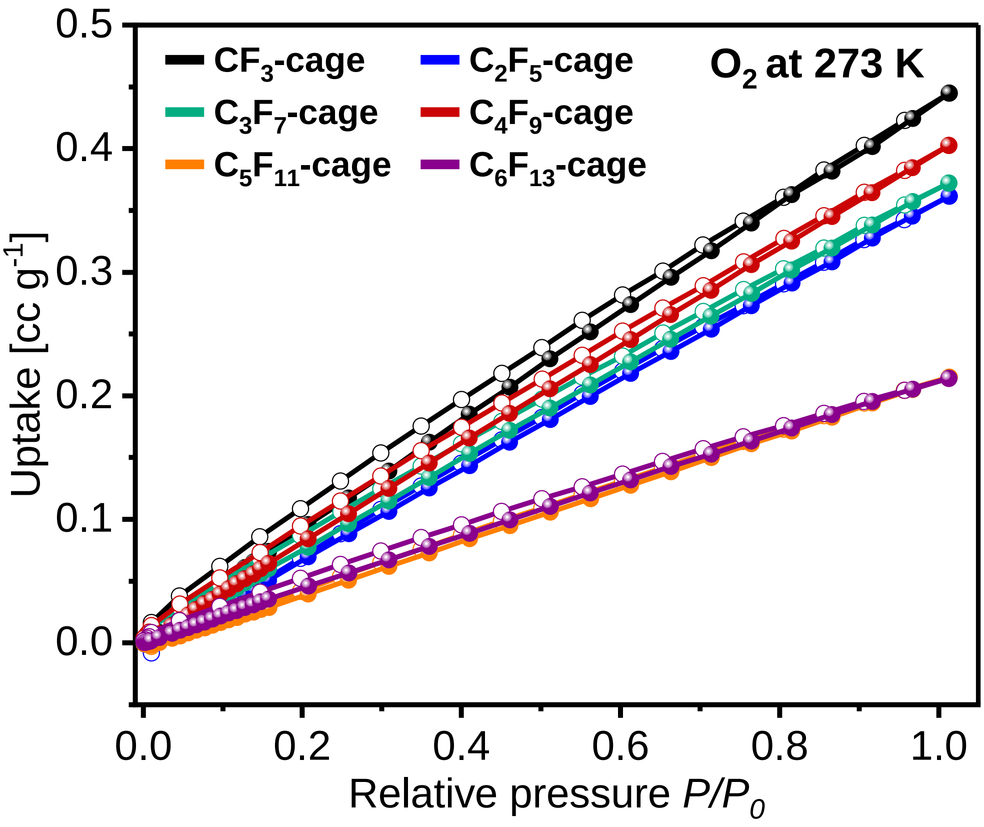

Figure S159. O_2 sorption isotherms at 273 K. Black: **CF_3-cage**; blue: **C_2F_5-cage**; green: **C_3F_7-cage**; red: **C_4F_8-cage**; orange: **C_5F_{11}-cage**; purple: **C_6F_{13}-cage**. Full circles: adsorption; empty circles: desorption.

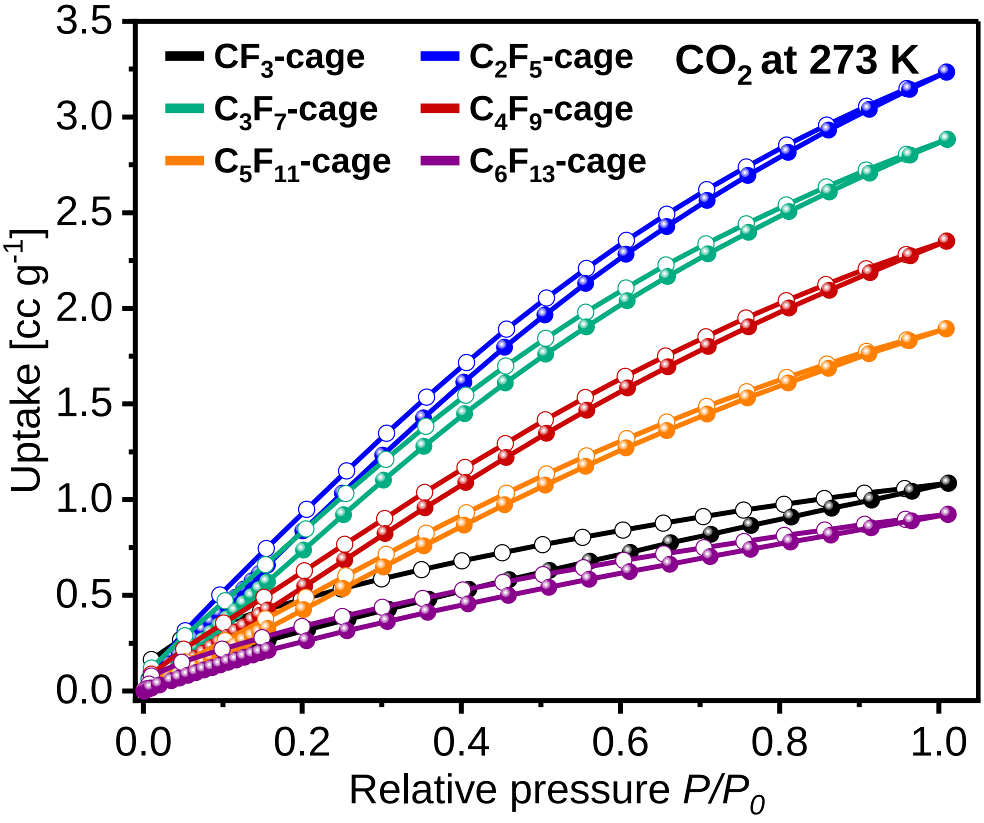

Figure S160. CO_2 sorption isotherms at 273 K. Black: **CF_3-cage**; blue: **C_2F_5-cage**; green: **C_3F_7-cage**; red: **C_4F_8-cage**; orange: **C_5F_{11}-cage**; purple: **C_6F_{13}-cage**. Full circles: adsorption; empty circles: desorption.

Table S11. Fitting and IAST parameters of Tóth and LAI isotherms as well as R^2 -values and Henry constants at 273 K.

Cages	Gas	Affinity const. *K* [1/bar]	Max. uptake *q*max [mmol/g]	Heterogenity Parameter	R^2	*Model*	K_H
**CF_3-cage**	CF_4	0.000311	1000	1	0.999464	LAI	0.311
	C_2F_6	0.001411	423.945005	0.2356	0.999981	Tóth	0.598186402
	C_3F_8	0.000298	1000	1	0.988971	LAI	0.298
	c-C_4F_8	0.000426	1000	1	0.943371	LAI	0.426
	SF_6	0.001766	443.854573	0.2086	0.999846	Tóth	0.783847176
	NF_3	0.050550	10.827757	0.4995	0.999891	Tóth	0.547343116
	N_2	0.412943	0.686558	3.0421	0.999968	Tóth	0.283500932
	O_2	0.022671	20.558043	0.7893	0.999914	Tóth	0.466071393
	CO_2	0.001163	1000	1	0.981509	LAI	1.163
**C_2F_5-cage**	CF_4	0.332331	4.399286	1.0086	0.999989	Tóth	1.462019116
	C_2F_6	9.161702	2.690105	0.6254	0.999205	Tóth	24.64594036
	C_3F_8	273.250874	2.755367	0.3516	0.996523	Tóth	752.9064409
	c-C_4F_8	3640.315942	2.764162	0.2900	0.989907	Tóth	10062.42299
	SF_6	6.944563	2.622415	0.8715	0.999270	Tóth	18.21152618
	NF_3	0.506885	2.730789	1.8048	0.999872	Tóth	1.384195982
	N_2	0.000333	1000	1	0.999799	LAI	0.333
	O_2	0.000354	1000	1	0.998255	LAI	0.354
	CO_2	0.879734	4.793023	2.0790	0.999994	Tóth	4.216585296
**C_3F_7-cage**	CF_4	0.586949	2.024832	1.7041	0.999987	Tóth	1.188473118
	C_2F_6	9.964861	2.972831	0.4563	0.999741	Tóth	29.62384769
	C_3F_8	447.866500	1.624146	0.4379	0.992885	Tóth	727.4005845
	c-C_4F_8	1088.025197	1.204675	0.9150	0.991086	Tóth	1310.716754
	SF_6	9.527190	1.995162	0.7541	0.998833	Tóth	19.00828745
	NF_3	0.298820	3.986254	1.2328	0.999994	Tóth	1.190231231
	N_2	0.086438	4.039532	1.3672	0.999972	Tóth	0.349169067
	O_2	0.000370	1000	1	0.999952	LAI	0.37
	CO_2	0.969902	3.785917	2.5471	0.999918	LAI	3.67196847
**C_4F_9-cage**	SF_6	7.760347	2.793672	0.6516	0.999268	Tóth	21.67986412
	NF_3	0.293659	3.680172	1.3249	0.999993	Tóth	1.080715629
	O_2	0.071192	5.697315	1.3297	0.999976	Tóth	0.405603249
**C_5F_{11}-cage**	CF_4	0.405172	2.324879	1.1619	0.999988	Tóth	0.941975874
	C_2F_6	9.060382	2.231758	0.5458	0.999237	Tóth	20.22058001
	C_3F_8	295.111200	2.736046	0.3084	0.998459	Tóth	807.4378183
	c-C_4F_8	3003.227683	2.678307	0.2657	0.994893	Tóth	8043.565726
	SF_6	6.569322	2.251886	0.6731	0.998764	Tóth	14.79336424
	NF_3	0.650189	1.226555	2.9882	0.999786	Tóth	0.797492569
	N_2	0.000209	1000	1	0.999871	LAI	0.209
	O_2	0.000208	1000	1	0.997428	LAI	0.208
	CO_2	0.917522	2.323443	4.0001	0.999843	Tóth	2.131810068
**C_6F_{13}-cage**	CF_4	0.191404	2.313105	0.8075	0.999981	Tóth	0.442737549
	C_2F_6	0.000601	1000	1	0.987473	LAI	0.601
	C_3F_8	0.000792	1000	1	0.985066	LAI	0.792
	c-C_4F_8	0.000581	1000	1	0.870642	LAI	0.581
	SF_6	0.00081	1000	1	0.971679	LAI	0.81
	NF_3	0.259592	1.852260	0.9454	0.999961	Tóth	0.480831878
	N_2	0.000196	1000	1	0.999485	LAI	0.196
	O_2	0.002186	102.473703	0.5643	0.999966	Tóth	0.224007515
	CO_2	0.065206	30.400168	0.3842	0.999932	Tóth	1.982273355

Gas sorption fitting curves at 273 K

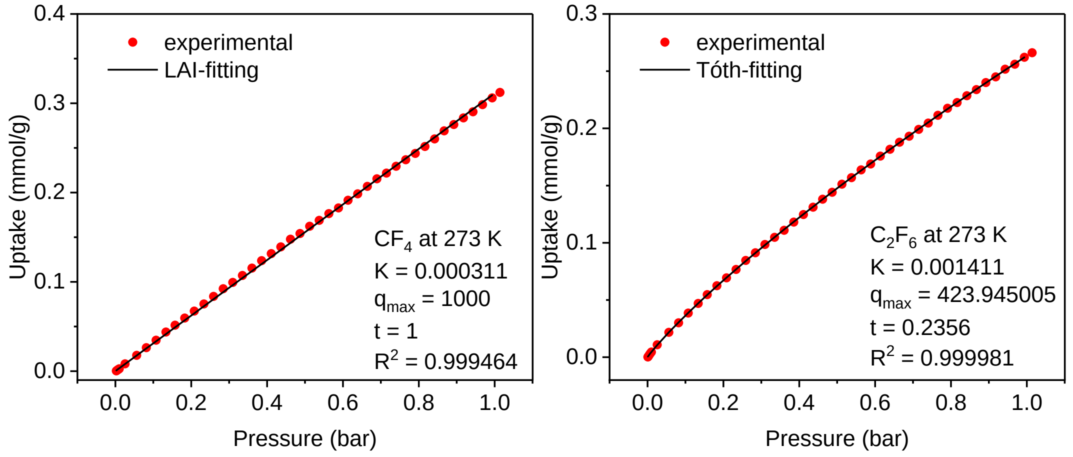

Figure S161. CF_4 (left) and C_2F_6 (right) isotherms and fitting curves of **CF_3-cage** at 273 K.

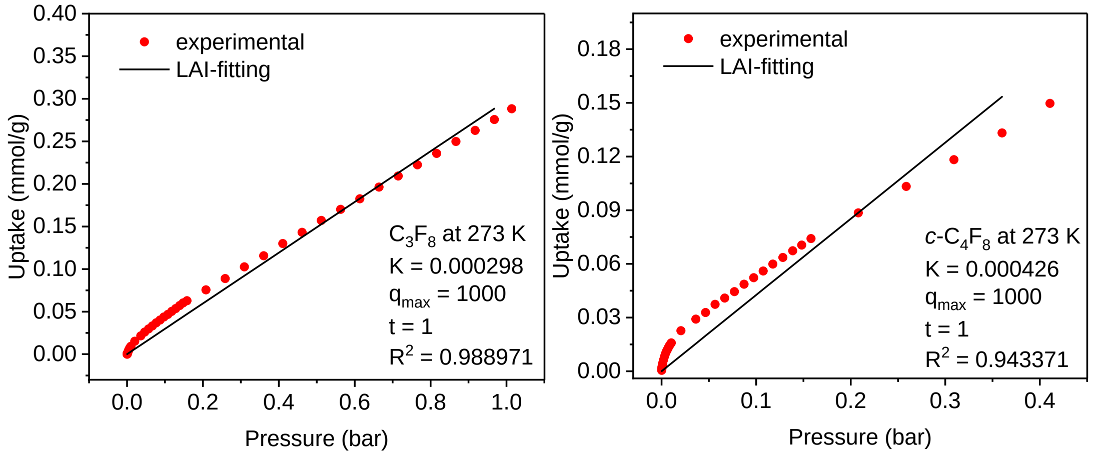

Figure S162. C_3F_8 (left) and *c*-C_4F_8 (right) isotherms and fitting curves of **CF_3-cage** at 273 K. (*Note*: Only the pressure range for *c*-C_4F_8 from 0-0.4 bar was taken into account due to pore condensation >0.4 bar)

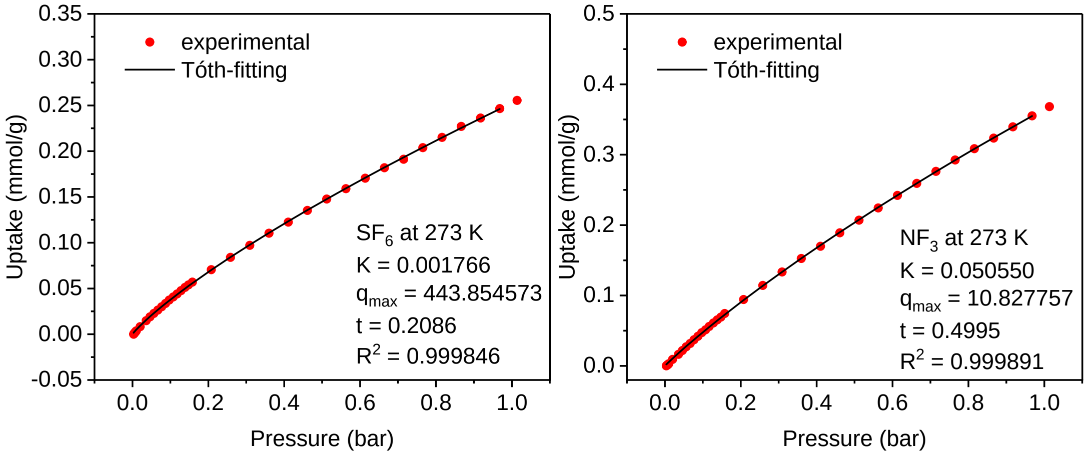

Figure S163. SF_6 (left) and NF_3 (right) isotherms and fitting curves of **CF_3-cage** at 273 K.

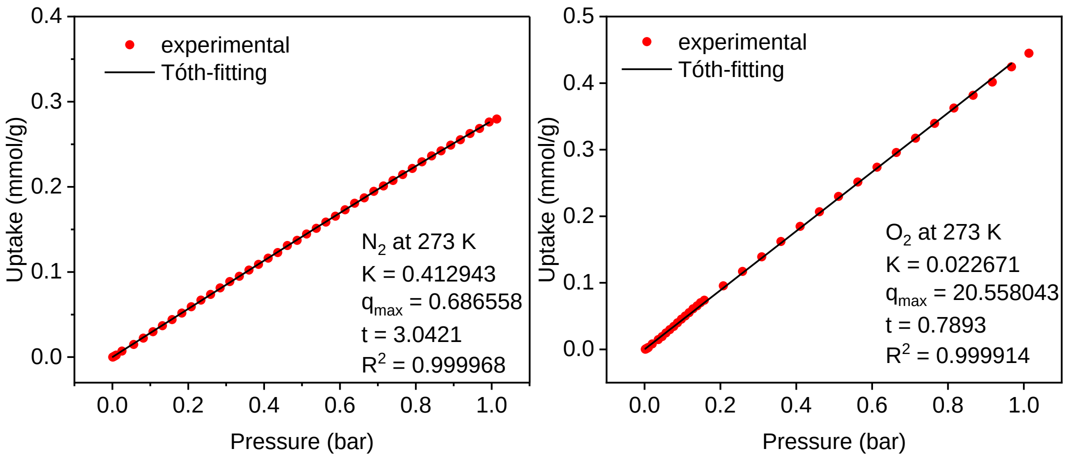

Figure S164. N_2 (left) and O_2 (right) isotherms and fitting curves of **CF_3-cage** at 273 K.

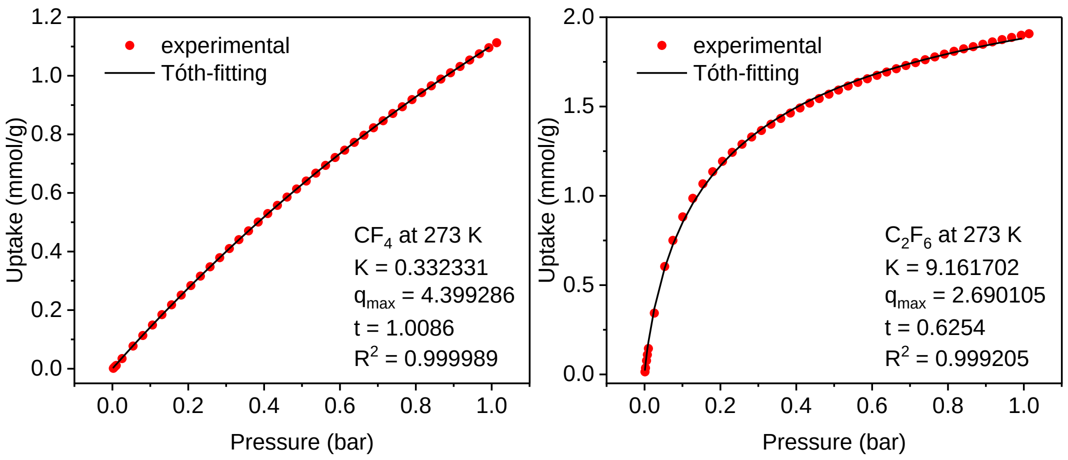

Figure S165. CF_4 (left) and C_2F_6 (right) isotherms and fitting curves of **C_2F_5-cage** at 273 K.

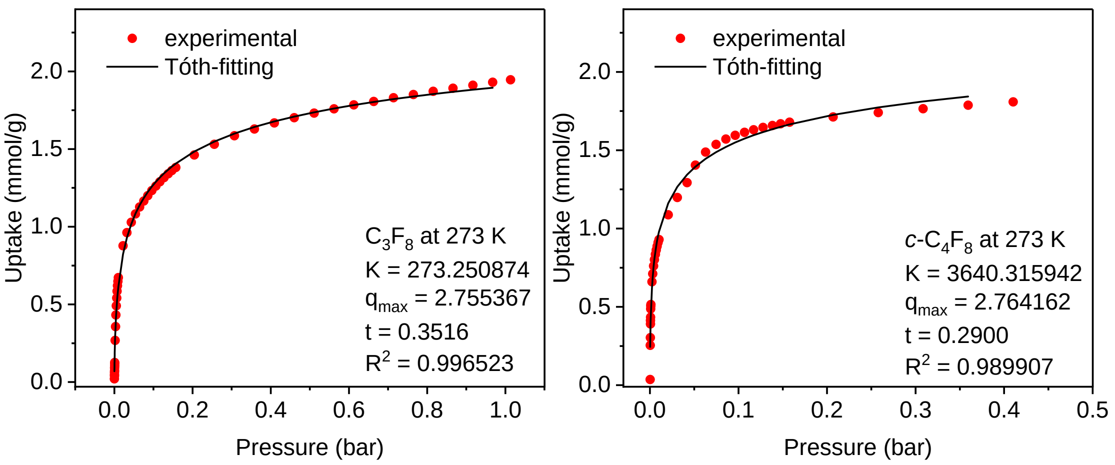

Figure S166. C_3F_8 (left) and *c*-C_4F_8 (right) isotherms and fitting curves of **C_2F_5-cage** at 273 K. (*Note*: Only the pressure range for *c*-C_4F_8 from 0-0.4 bar was taken into account due to pore condensation >0.4 bar)

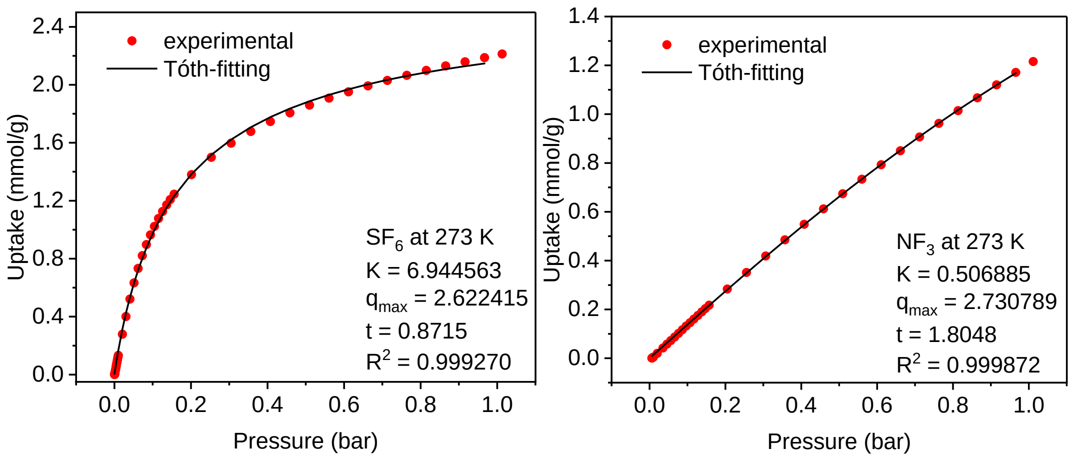

Figure S167. SF_6 (left) and NF_3 (right) isotherms and fitting curves of **C_2F_5-cage** at 273 K.

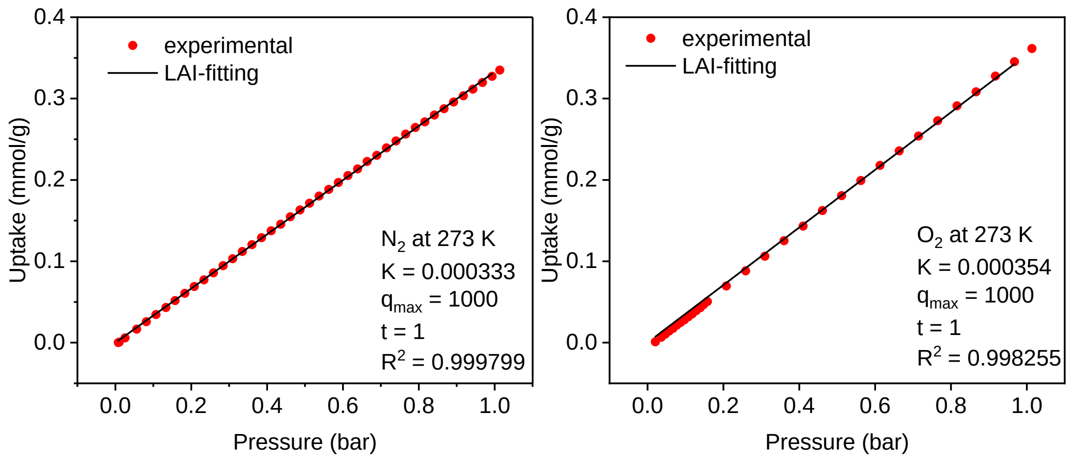

Figure S168. N_2 (left) and O_2 (right) isotherms and fitting curves of **C_2F_5-cage** at 273 K.

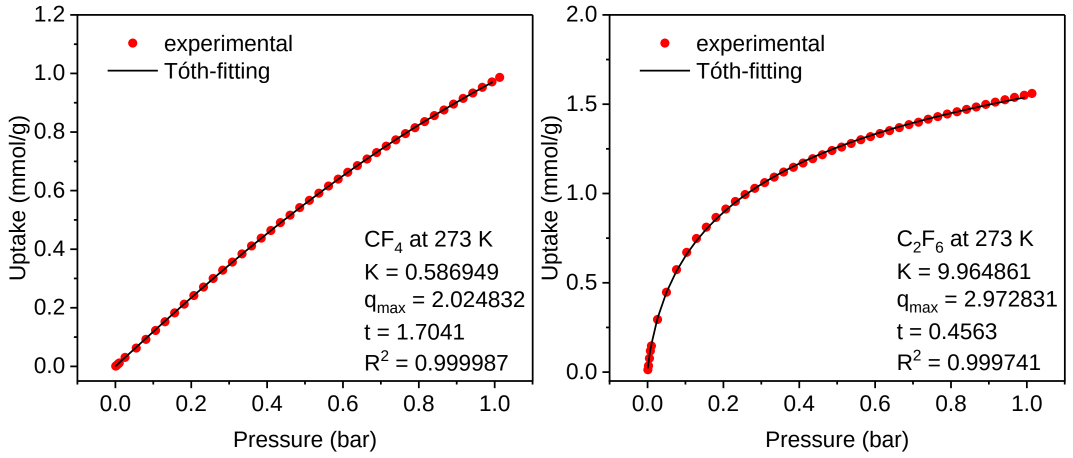

Figure S169. CF_4 (left) and C_2F_6 (right) isotherms and fitting curves of **C_3F_7-cage** at 273 K.

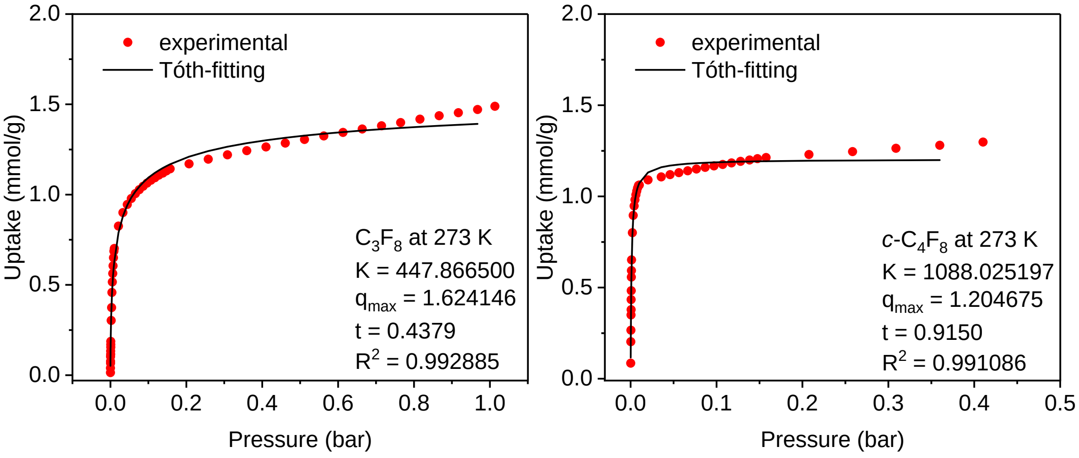

Figure S170. C_3F_8 (left) and *c*-C_4F_8 (right) isotherms and fitting curves of **C_3F_7-cage** at 273 K. (*Note*: Only the pressure range for *c*-C_4F_8 from 0-0.4 bar was taken into account due to pore condensation >0.4 bar)

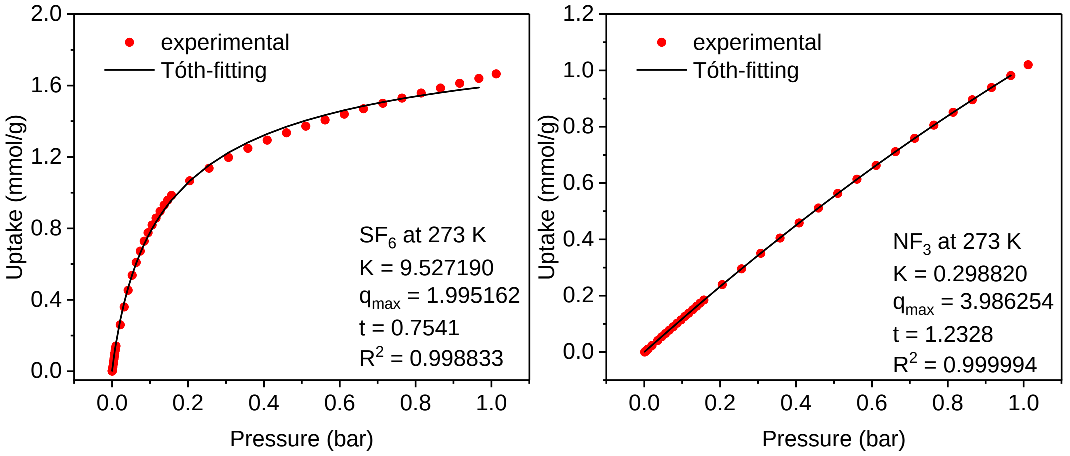

Figure S171. SF_6 (left) and NF_3 (right) isotherms and fitting curves of **C_3F_7-cage** at 273 K.

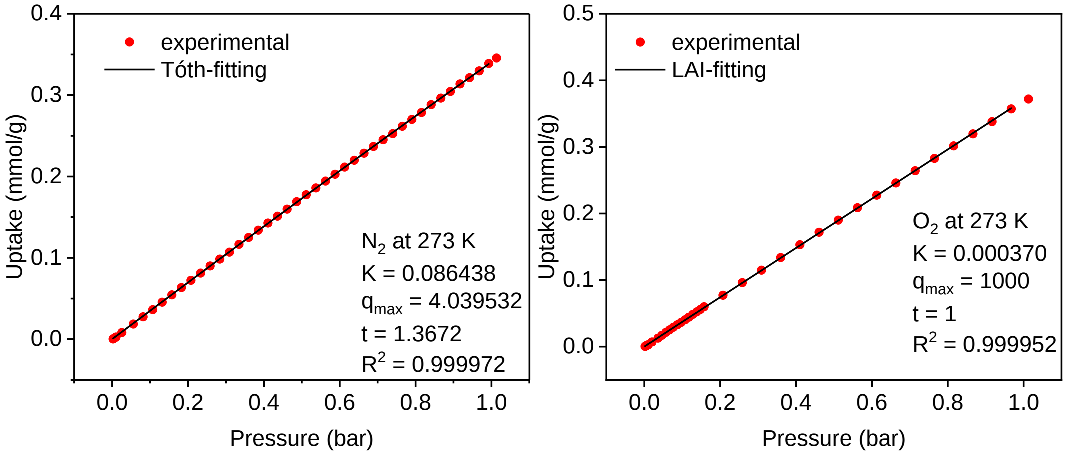

Figure S172. N_2 (left) and O_2 (right) isotherms and fitting curves of **C_3F_7-cage** at 273 K.

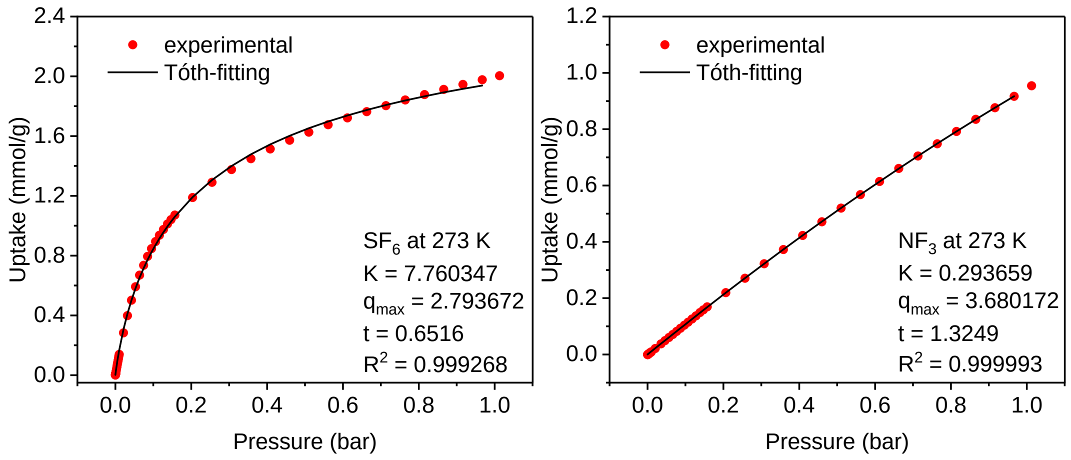

Figure S173. SF_6 (left) and NF_3 (right) isotherms and fitting curves of **C_4F_9-cage** at 273 K.

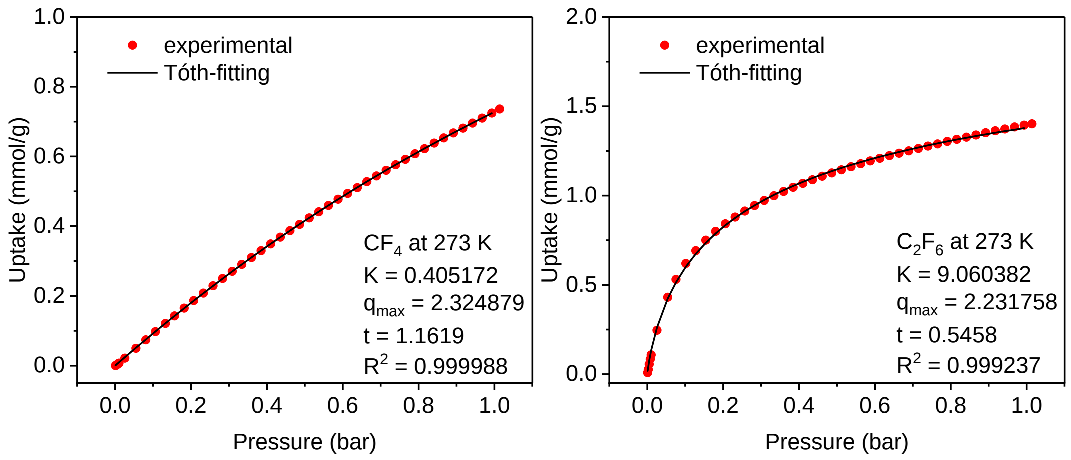

Figure S174. CF_4 (left) and C_2F_6 (right) isotherms and fitting curves of **C_5F_{11}-cage** at 273 K.

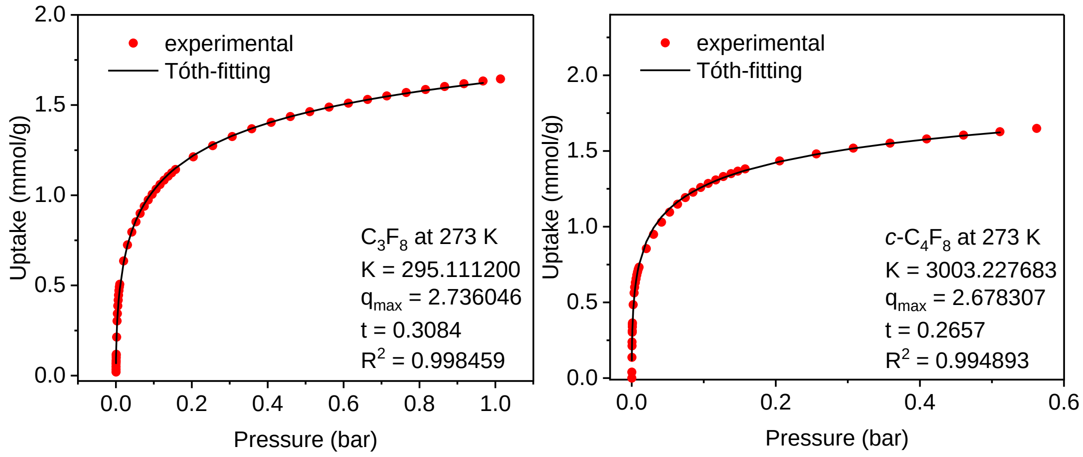

Figure S175. C_3F_8 (left) and *c*-C_4F_8 (right) isotherms and fitting curves of **C_5F_{11}-cage** at 273 K. (*Note*: Only the pressure range for *c*-C_4F_8 from 0-0.55 bar was taken into account due to pore condensation >0.55 bar)

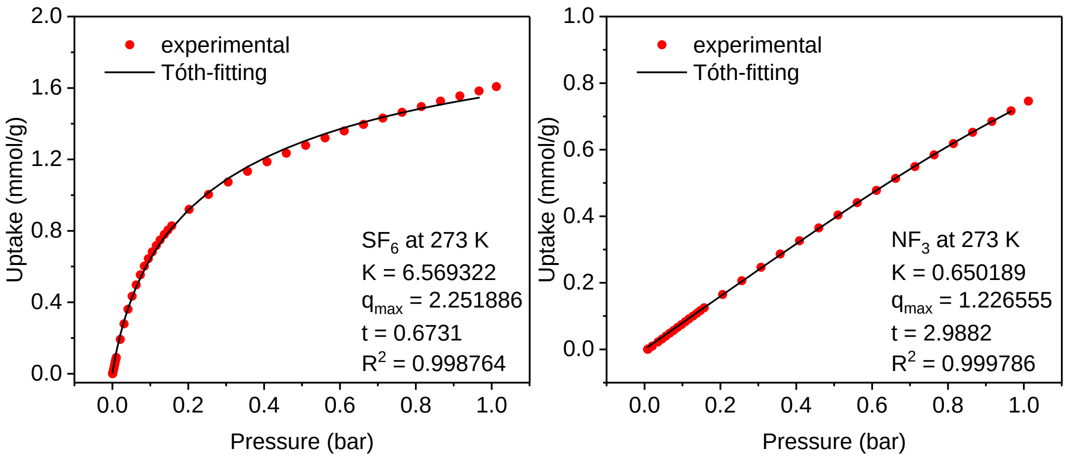

Figure S176. SF_6 (left) and NF_3 (right) isotherms and fitting curves of **C_5F_{11}-cage** at 273 K.

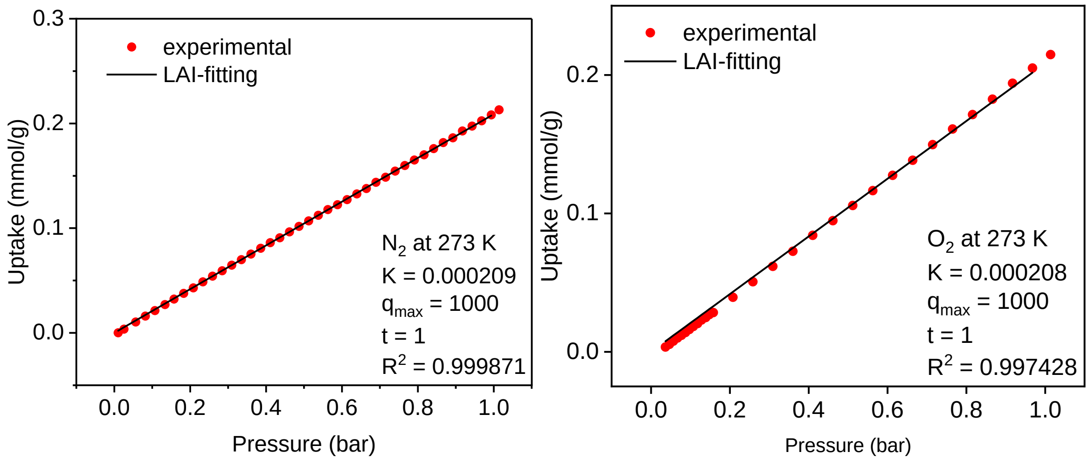

Figure S177. N_2 (left) and O_2 (right) isotherms and fitting curves of **C_5F_{11}-cage** at 273 K.

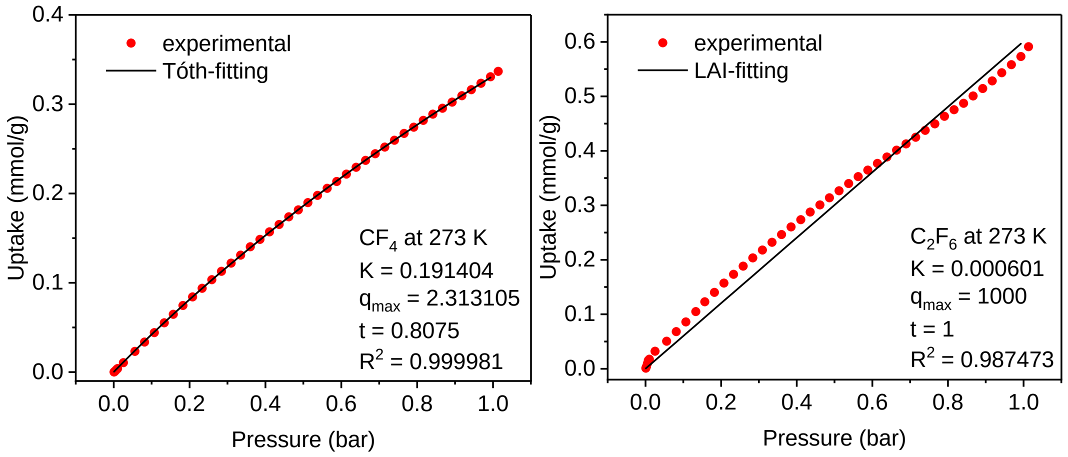

Figure S178. CF_4 (left) and C_2F_6 (right) isotherms and fitting curves of **C_6F_{13}-cage** at 273 K.

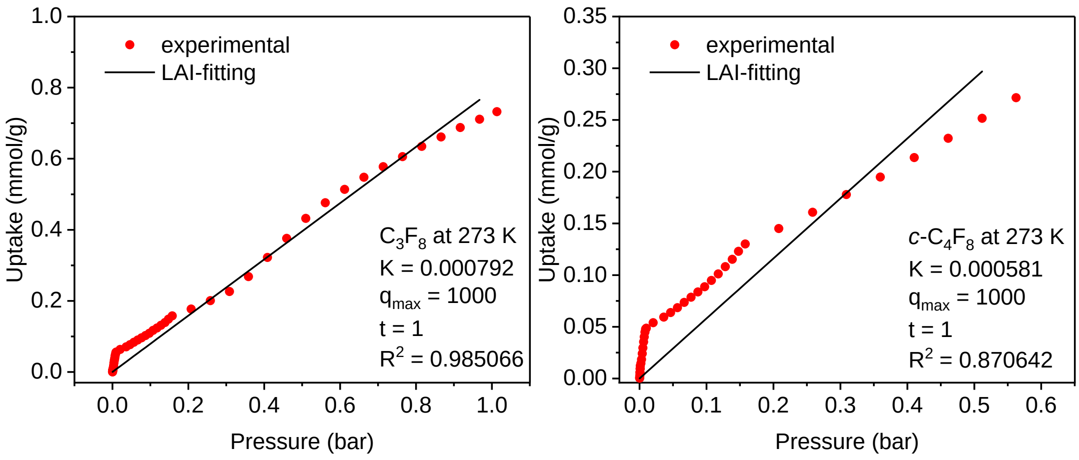

Figure S179. C_3F_8 (left) and *c*-C_4F_8 (right) isotherms and fitting curves of **C_6F_{13}-cage** at 273 K. (*Note*: Only the pressure range for *c*-C_4F_8 from 0-0.55 bar was taken into account due to pore condensation >0.55 bar)

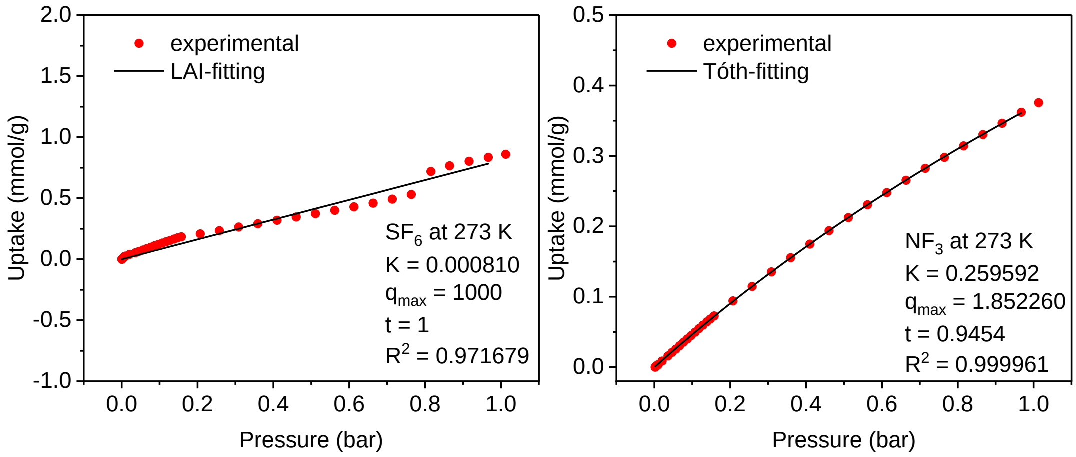

Figure S180. SF_6 (left) and NF_3 (right) isotherms and fitting curves of **C_6F_{13}-cage** at 273 K.

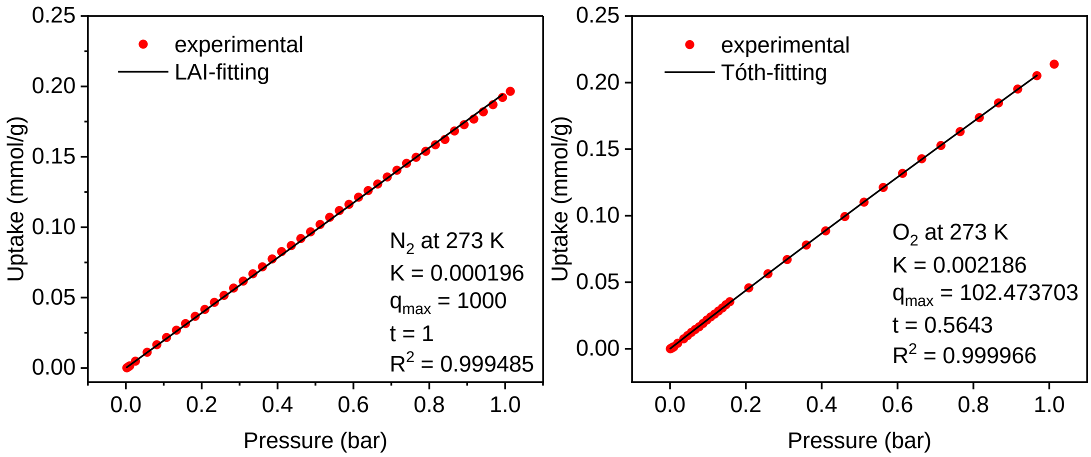

Figure S181. N_2 (left) and O_2 (right) isotherms and fitting curves of **C_6F_{13}-cage** at 273 K.

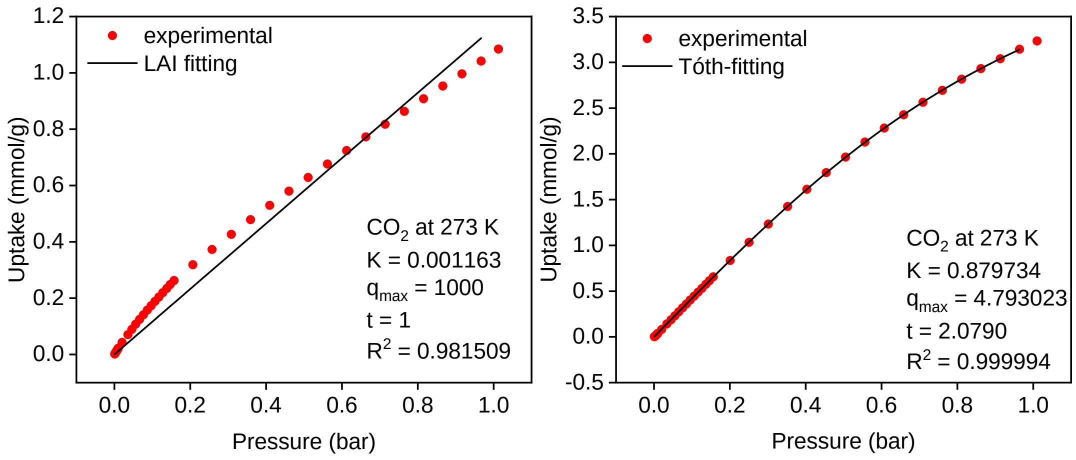

Figure S182. CO_2 isotherms and fitting curves of **CF_3-cage** (left) and **C_2F_5-cage** (right) at 273 K.

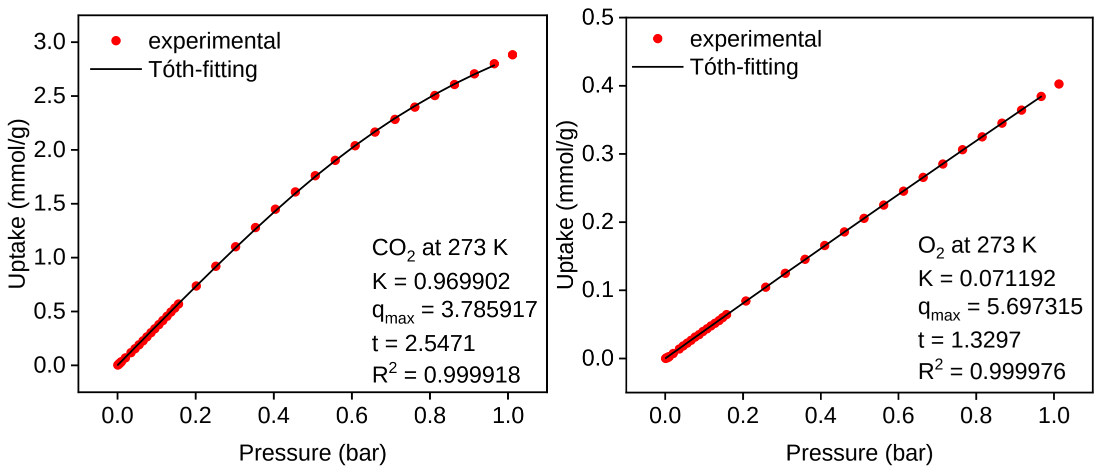

Figure S183. CO_2 isotherm and fitting curve of **C_3F_7-cage** (left) and O_2 isotherm and fitting curve of **C_4F_9-cage** (right) at 273 K.

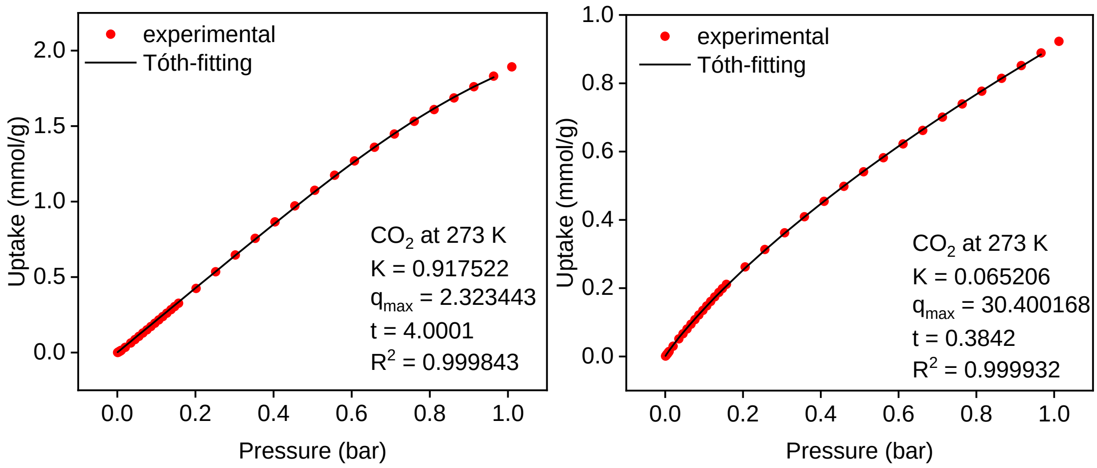

Figure S184. CO_2 isotherms and fitting curves of **C_5F_{11}-cage** (left) and **C_6F_{13}-cage** (right) at 273 K.

Gas Sorption at 283 K

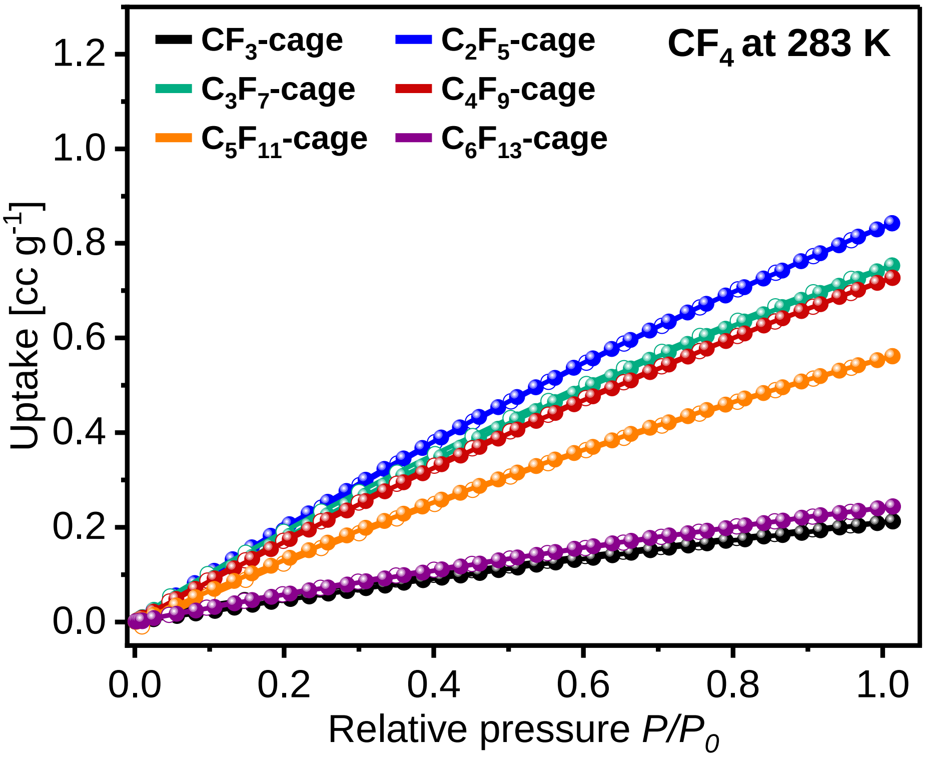

Figure S185. CF_4 sorption isotherms at 283 K. Black: **CF_3-cage**; blue: **C_2F_5-cage**; green: **C_3F_7-cage**; red: **C_4F_8-cage**; orange: **C_5F_{11}-cage**; purple: **C_6F_{13}-cage**. Full circles: adsorption; empty circles: desorption.

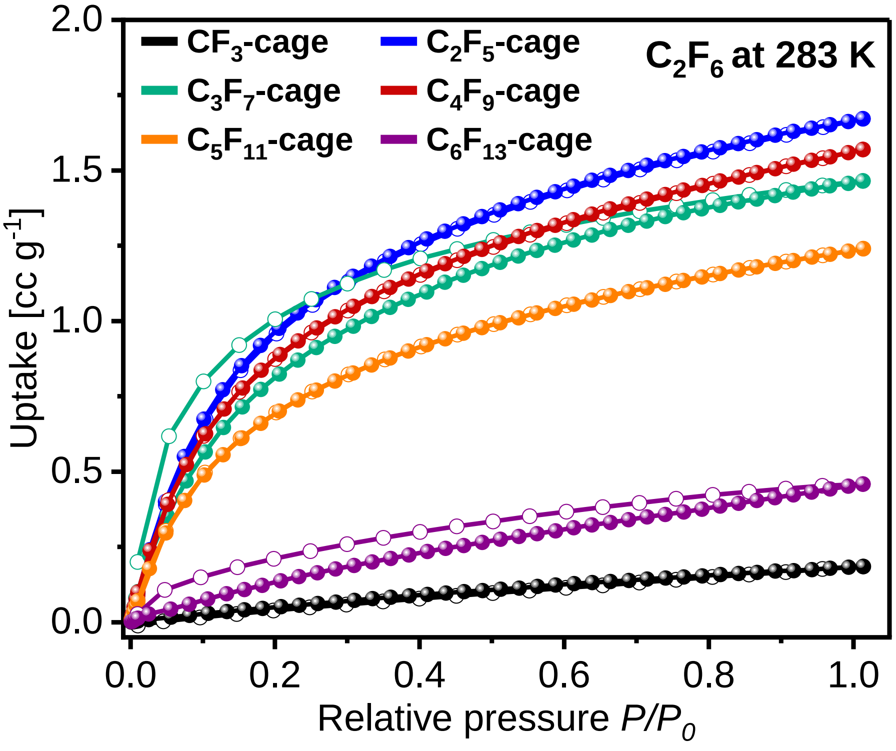

Figure S186. C_2F_6 sorption isotherms at 283 K. Black: **CF_3-cage**; blue: **C_2F_5-cage**; green: **C_3F_7-cage**; red: **C_4F_8-cage**; orange: **C_5F_{11}-cage**; purple: **C_6F_{13}-cage**. Full circles: adsorption; empty circles: desorption.

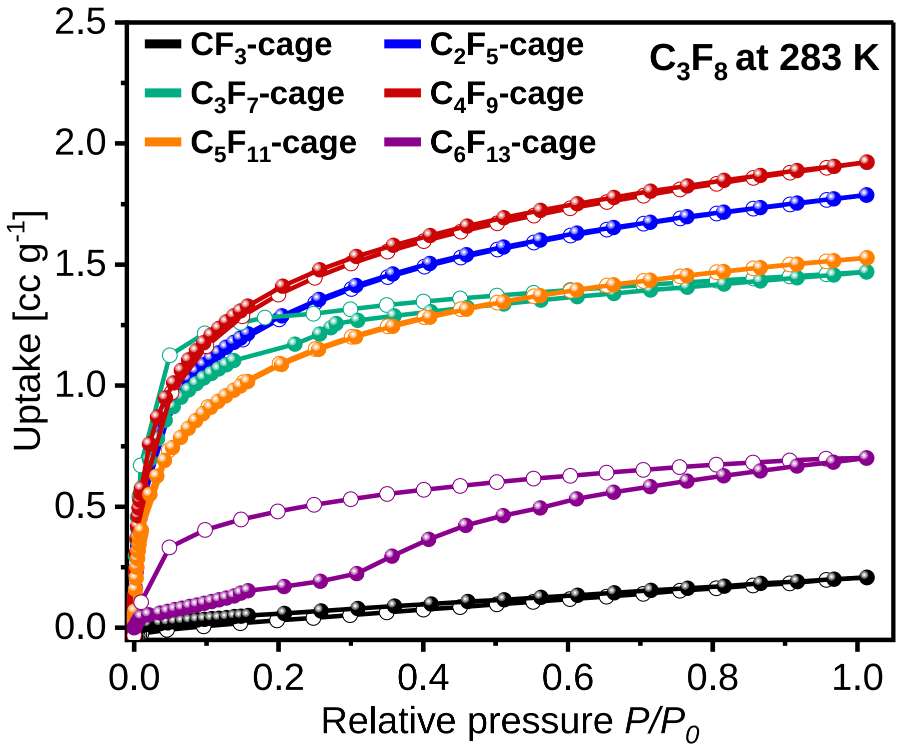

Figure S187. C_3F_8 sorption isotherms at 283 K. Black: **CF_3-cage**; blue: **C_2F_5-cage**; green: **C_3F_7-cage**; red: **C_4F_8-cage**; orange: **C_5F_{11}-cage**; purple: **C_6F_{13}-cage**. Full circles: adsorption; empty circles: desorption.

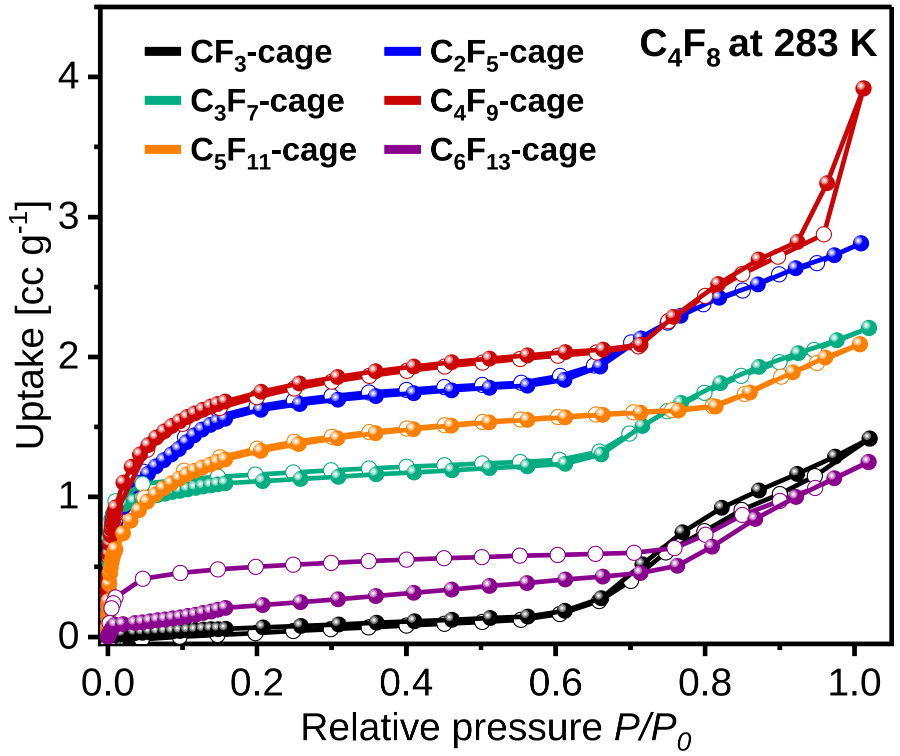

Figure S188. C_4F_8 sorption isotherms at 283 K. Black: **CF_3-cage**; blue: **C_2F_5-cage**; green: **C_3F_7-cage**; red: **C_4F_8-cage**; orange: **C_5F_{11}-cage**; purple: **C_6F_{13}-cage**. Full circles: adsorption; empty circles: desorption.

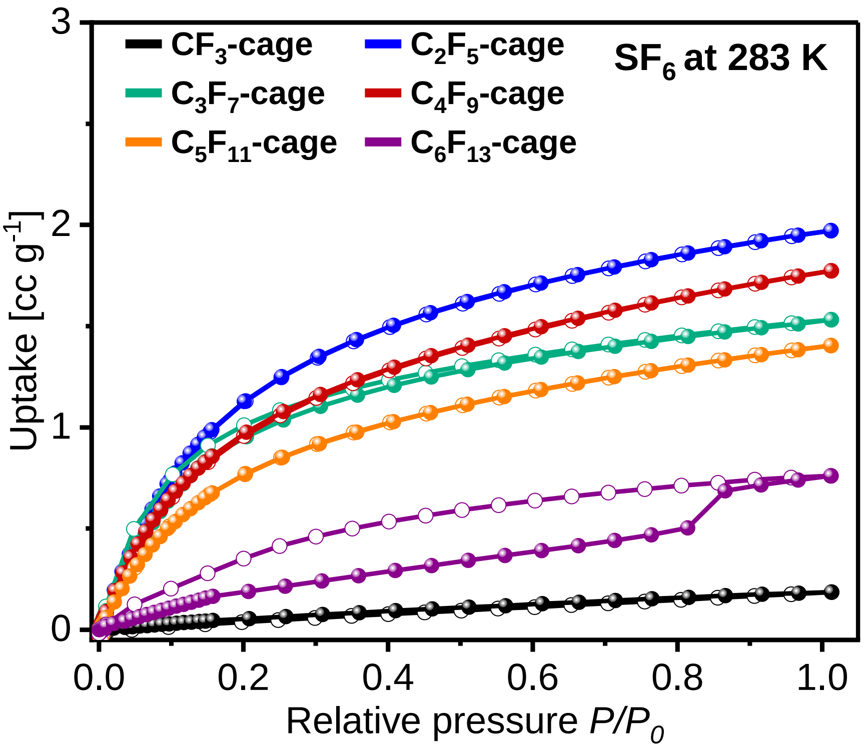

Figure S189. SF_6 sorption isotherms at 283 K. Black: **CF_3-cage**; blue: **C_2F_5-cage**; green: **C_3F_7-cage**; red: **C_4F_8-cage**; orange: **C_5F_{11}-cage**; purple: **C_6F_{13}-cage**. Full circles: adsorption; empty circles: desorption.

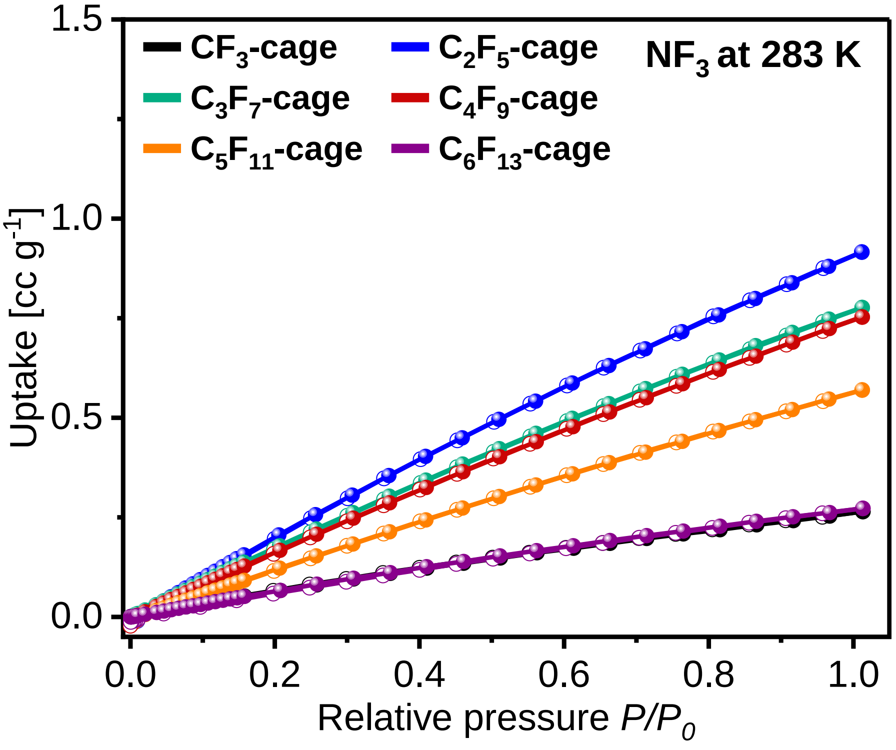

Figure S190. NF_3 sorption isotherms at 283 K. Black: **CF_3-cage**; blue: **C_2F_5-cage**; green: **C_3F_7-cage**; red: **C_4F_8-cage**; orange: **C_5F_{11}-cage**; purple: **C_6F_{13}-cage**. Full circles: adsorption; empty circles: desorption.

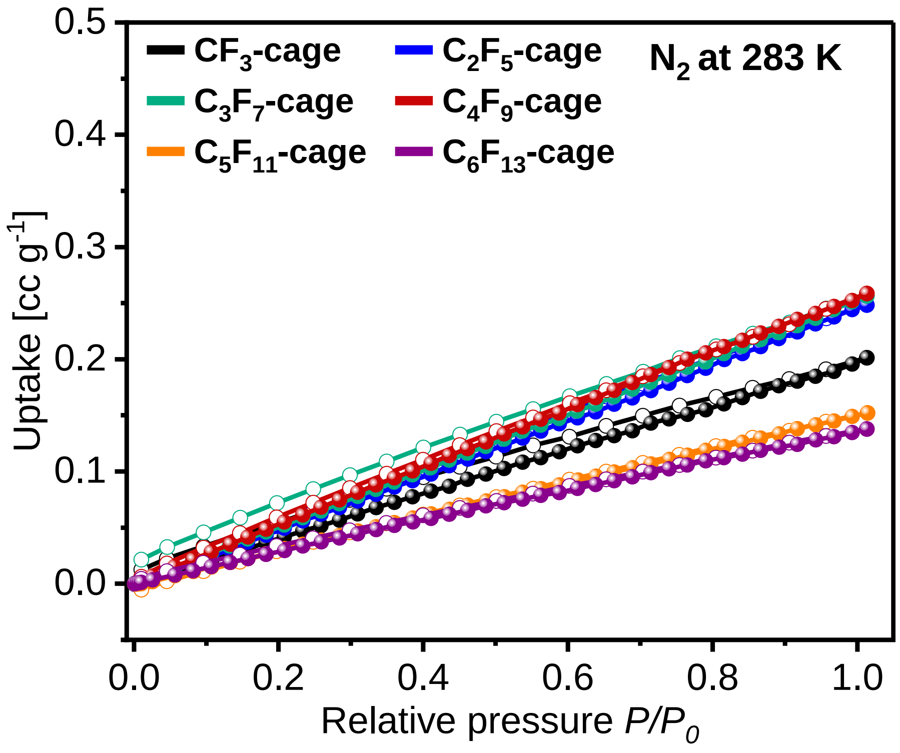

Figure S191. N_2 sorption isotherms at 283 K. Black: **CF_3-cage**; blue: **C_2F_5-cage**; green: **C_3F_7-cage**; red: **C_4F_8-cage**; orange: **C_5F_{11}-cage**; purple: **C_6F_{13}-cage**. Full circles: adsorption; empty circles: desorption.

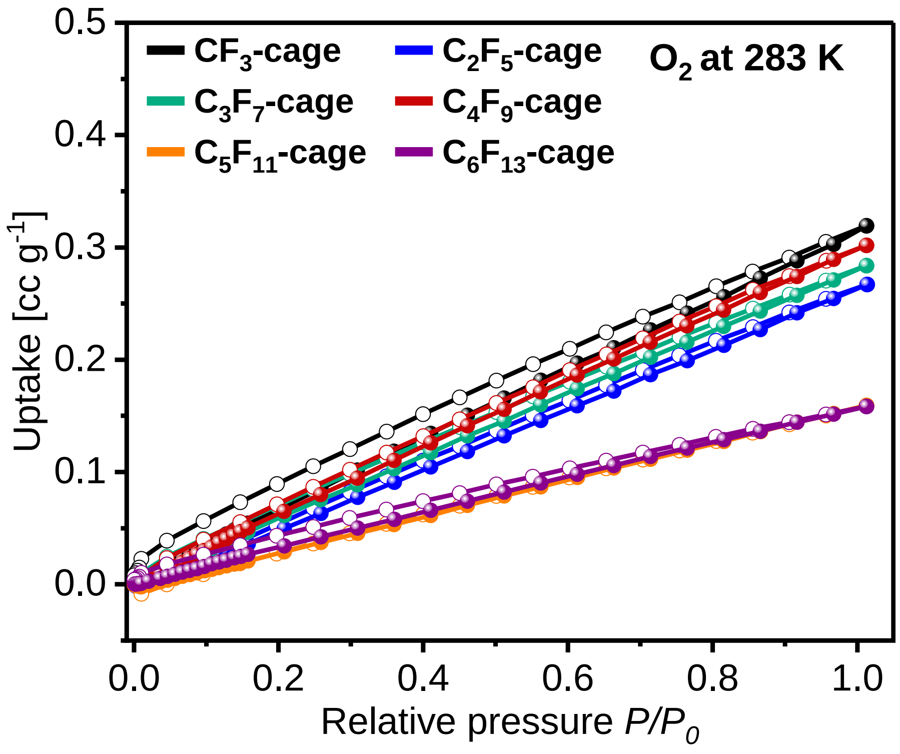

Figure S192. O_2 sorption isotherms at 273 K. Black: **CF_3-cage**; blue: **C_2F_5-cage**; green: **C_3F_7-cage**; red: **C_4F_8-cage**; orange: **C_5F_{11}-cage**; purple: **C_6F_{13}-cage**. Full circles: adsorption; empty circles: desorption.

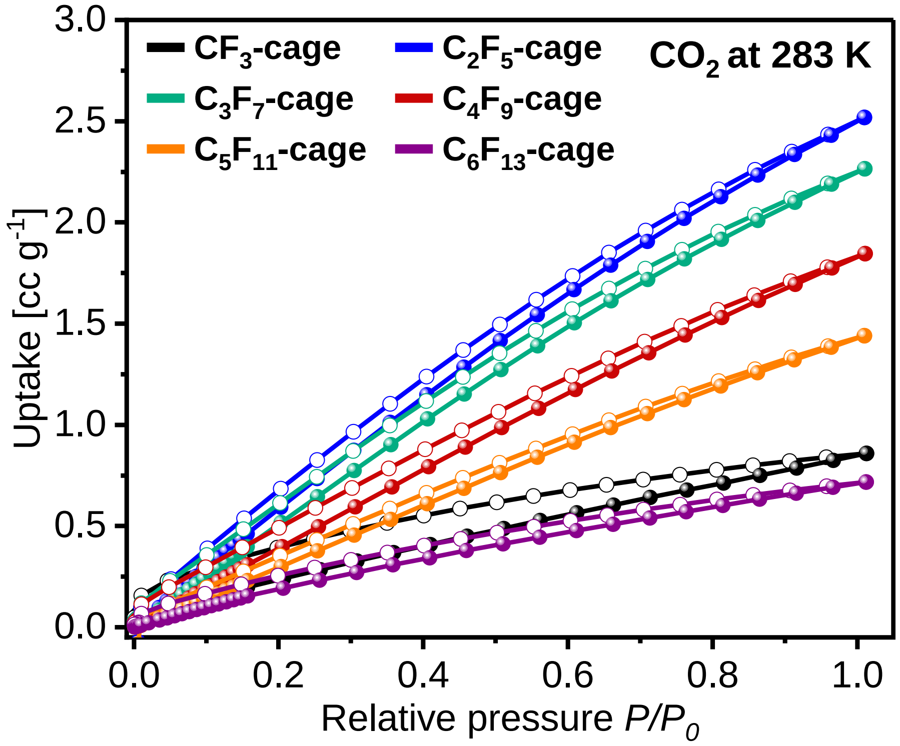

Figure S193. CO_2 sorption isotherms at 273 K. Black: **CF_3-cage**; blue: **C_2F_5-cage**; green: **C_3F_7-cage**; red: **C_4F_8-cage**; orange: **C_5F_{11}-cage**; purple: **C_6F_{13}-cage**. Full circles: adsorption; empty circles: desorption.

Table S12. Fitting and IAST parameters of Tóth and LAI isotherms as well as R^2 -values and Henry constants at 283 K.

Cages	Gas	Affinity const. *K* [1/bar]	Max. uptake *q*max [mmol/g]	Heterogenity Parameter	R^2	*Model*	K_H
**CF_3-cage**	CF_4	0.472838	0.483754	2.0519	0.999872	Tóth	0.228737274
	C_2F_6	0.212376	1.408269	0.6407	0.999931	Tóth	0.299082537
	C_3F_8	0.561981	0.543932	1	0.995350	LAI	0.305679449
	c-C_4F_8	0.000286	1000	1	0.945654	LAI	0.286
	SF_6	0.037641	12.017168	0.3281	0.999697	Tóth	0.452338221
	NF_3	0.163496	2.111046	0.7694	0.999867	Tóth	0.345147577
	N_2	0.000198	1000	1	0.999764	LAI	0.198
	O_2	0.000431	868.848044	0.3493	0.999908	Tóth	0.374473507
	CO_2	0.000906	1000	1	0.987066	LAI	0.906
**C_2F_5-cage**	CF_4	0.330856	3.106560	1.1652	0.999983	Tóth	1.027824015
	C_2F_6	5.723274	2.372526	0.7068	0.999600	Tóth	13.57861637
	C_3F_8	123.440331	2.548337	0.3854	0.997915	Tóth	314.5675628
	c-C_4F_8	2240.091296	3.268557	0.2578	0.993329	Tóth	7321.866086
	SF_6	4.616735	2.440404	0.9317	0.999645	Tóth	11.26669856
	NF_3	0.548072	1.807858	2.3323	0.999804	Toth	0.99083635
	N_2	0.000243	1000	1	0.999447	LAI	0.243
	O_2	0.000259	1000	1	0.997700	LAI	0.259
	CO_2	0.598023	4.890996	1.9680	0.999984	Tóth	2.924928101
**C_3F_7-cage**	CF_4	0.551931	1.606279	1.7280	0.999990	Tóth	0.886555175
	C_2F_6	5.307452	2.311466	0.6417	0.999965	Tóth	12.26799484
	C_3F_8	230.435241	1.751803	0.4388	0.997961	Tóth	403.6771465
	c-C_4F_8	533.711144	1.125576	0.8793	0.985814	Tóth	600.7324546
	SF_6	5.98532	1.768669	0.9618	0.999454	Tóth	10.58604994
	NF_3	0.254510	3.442872	1.2614	0.999990	Tóth	0.876245353
	N_2	0.000251	1000	1	0.999960	LAI	0.251
	O_2	0.015008	19.545120	0.7764	0.999951	Tóth	0.293333161
	CO_2	0.818138	3.102252	3.2278	0.999959	Tóth	2.538070247
**C_4F_9-cage**	SF_6	4.816870	2.561754	0.7176	0.999582	Tóth	12.33963599
	NF_3	0.411658	1.949939	2.0129	0.999910	Tóth	0.802707989
	O_2	0.088627	3.507033	1.2056	0.999914	Tóth	0.310817814
	CO_2	0.700949	2.770941	3.7256	0.999982	Tóth	1.942288323
**C_5F_{11}-cage**	CF_4	0.418420	1.579806	1.4423	0.999967	Tóth	0.661022427
	C_2F_6	5.754379	1.975468	0.6126	0.999543	Tóth	11.36759157
	C_3F_8	134.043627	2.582908	0.3341	0.998884	Tóth	346.2223565
	c-C_4F_8	1427.025306	2.767037	0.2672	0.997638	Tóth	3948.631822
	SF_6	4.527728	1.922501	0.7853	0.999304	Tóth	8.704561608
	NF_3	0.675958	0.872322	3.8768	0.999721	Tóth	0.589653034
	N_2	0.000150	1000	1	0.999728	LAI	0.15
	O_2	0.000155	1000	1	0.996517	LAI	0.155
	CO_2	0.001471	1000	1	0.999458	LAI	1.471
**C_6F_{13}-cage**	CF_4	0.209546	1.411045	0.9697	0.999975	Tóth	0.295678836
	C_2F_6	0.025736	61.985353	0.2585	0.999779	Tóth	1.595255045
	C_3F_8	1.239187	0.717459	5.3626	0.993351	Tóth	0.793189
	c-C_4F_8	0.000872	1000	1	0.896120	LAI	0.872
	SF_6	0.000707	1000	1	0.000707	LAI	0.707
	NF_3	0.357706	0.911527	1.2471	0.999949	Tóth	0.326058677
	N_2	0.147481	0.979210	1.2678	0.999905	Tóth	0.14441487
	O_2	0.026959	6.137539	0.8380	0.999906	Tóth	0.165461914
	CO_2	0.076282	15.623667	0.4839	0.999940	Tóth	1.191804566

Gas sorption fitting curves at 283 K

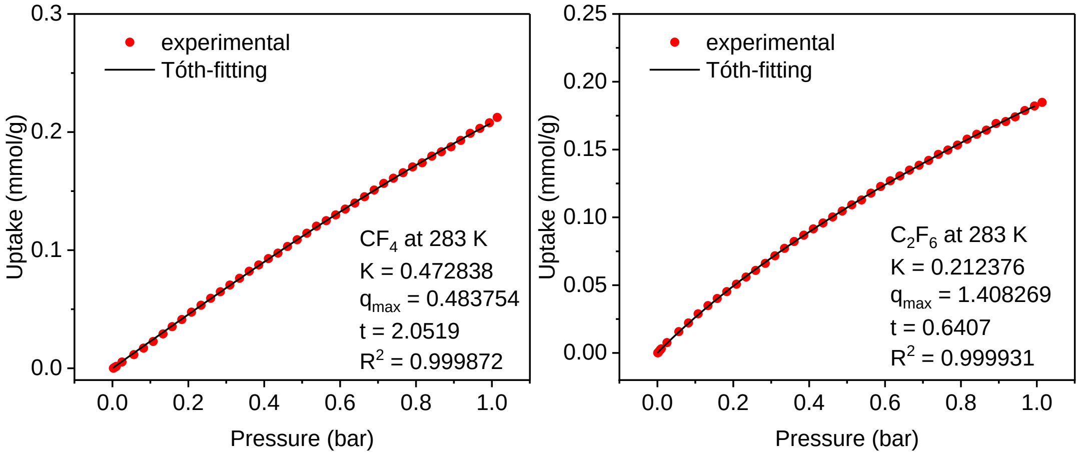

Figure S194. CF_4 (left) and C_2F_6 (right) isotherms and fitting curves of **CF_3-cage** at 283 K.

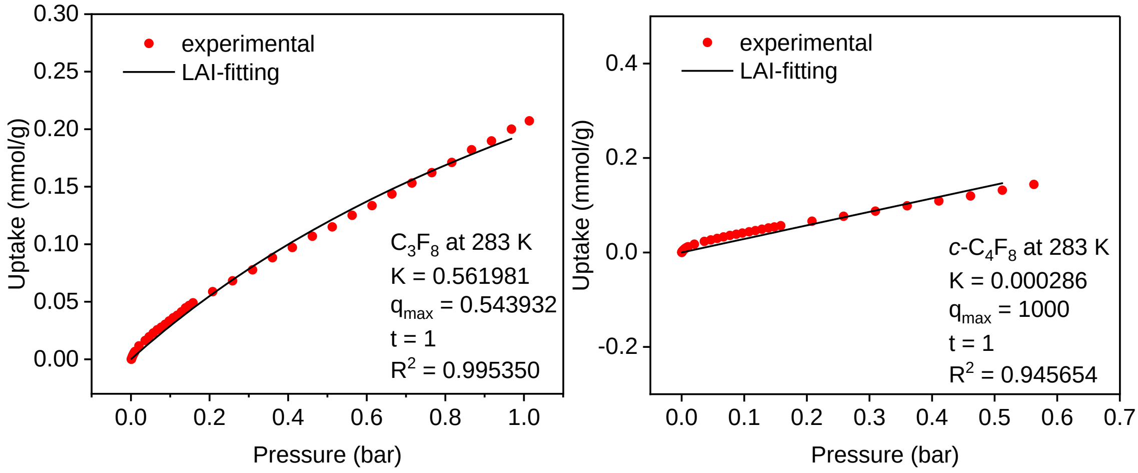

Figure S195. C_3F_8 (left) and *c*-C_4F_8 (right) isotherms and fitting curves of of **CF_3-cage** at 283 K. (Note: Only the pressure range for *c*-C_4F_8 from 0-0.6 bar was taken into account due to pore condensation >0.6 bar)

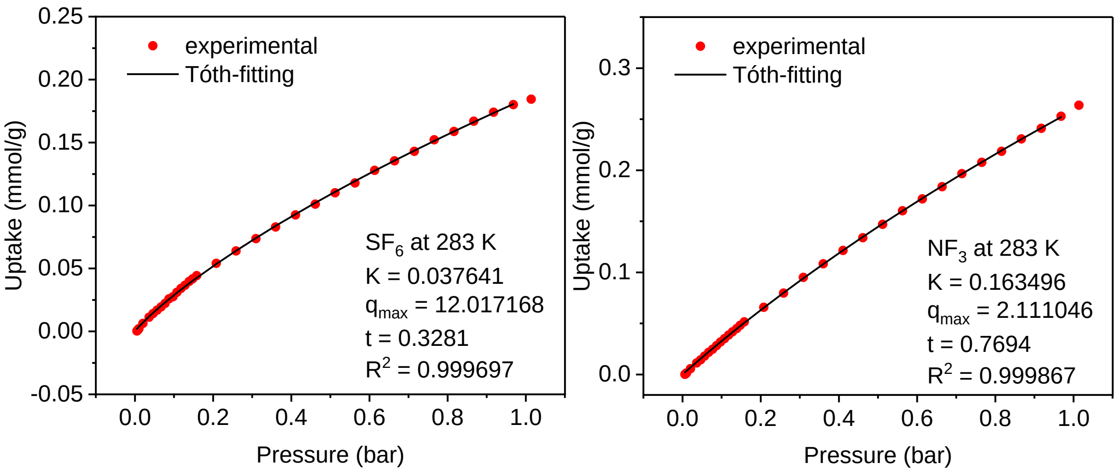

Figure S196. SF_6 (left) and NF_3 (right) isotherms and fitting curves of **CF_3-cage** at 283 K.

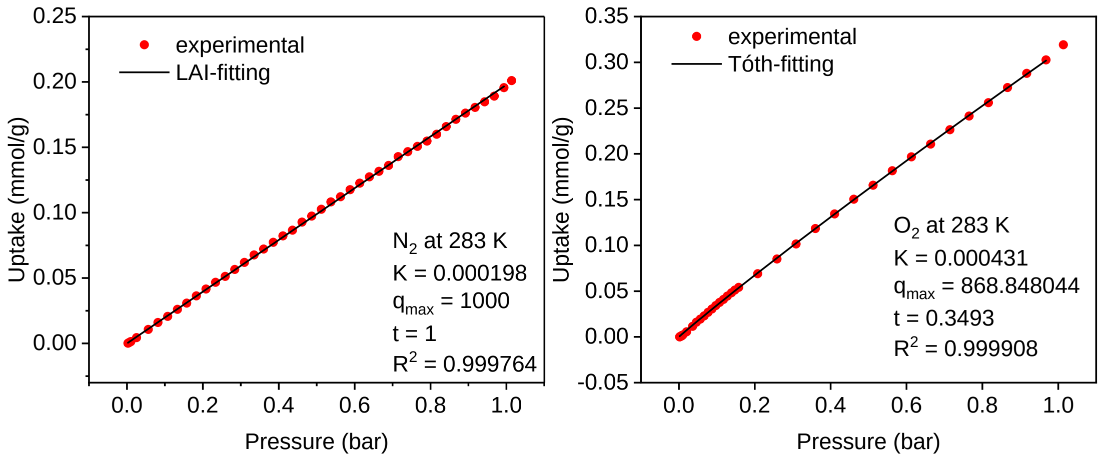

Figure S197. N_2 (left) and O_2 (right) isotherms and fitting curves of **CF_3-cage** at 283 K.

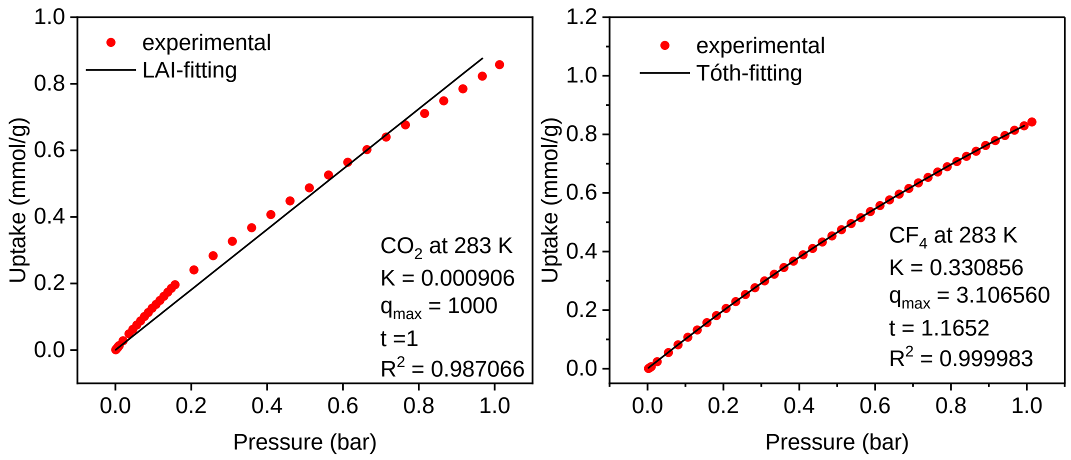

Figure S198. CO_2 isotherm and fitting curve of **CF_3-cage** (left) and CF_4 isotherm and fitting curve of **C_2F_5-cage** (right) at 283 K.

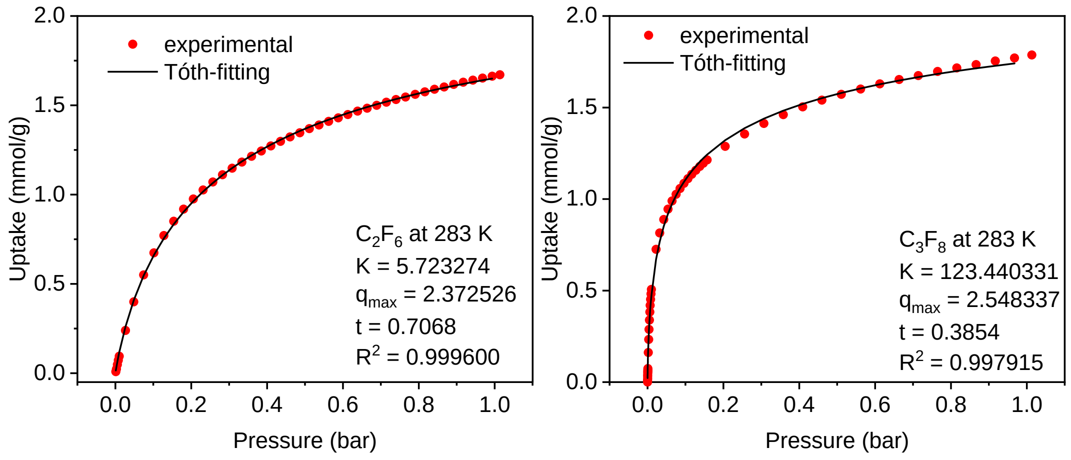

Figure S199. C_2F_6 (left) and C_3F_8 (right) isotherms and fitting curves of **C_2F_5-cage** at 283 K.

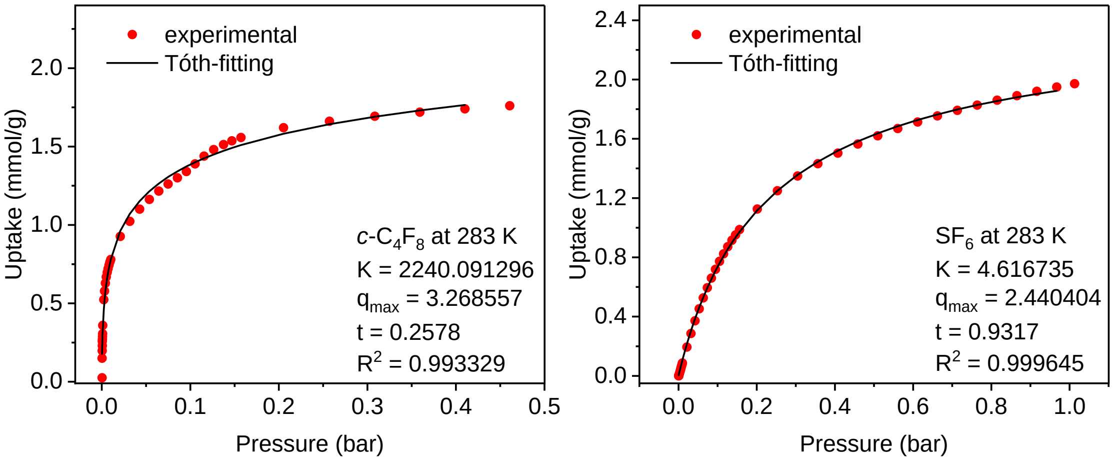

Figure S200. *c*-C_4F_8 (left) and SF_6 (right) isotherms and fitting curves of **C_2F_5-cage** at 283 K. (Note: Only the pressure range for from *c*-C_4F_8 0-0.6 bar was taken into account due to pore condensation >0.6 bar)

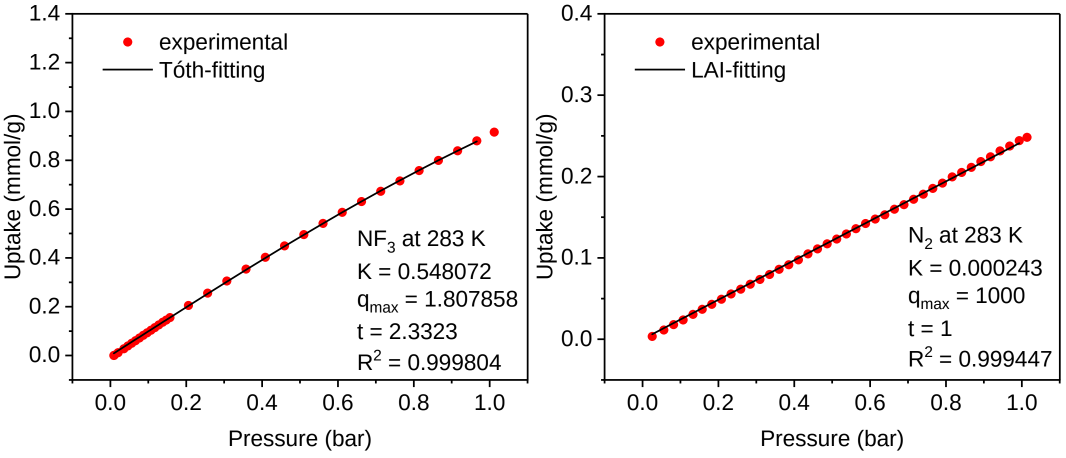

Figure S201. NF_3 (left) and N_2 (right) isotherms and fitting curves of **C_2F_5-cage** at 283 K.

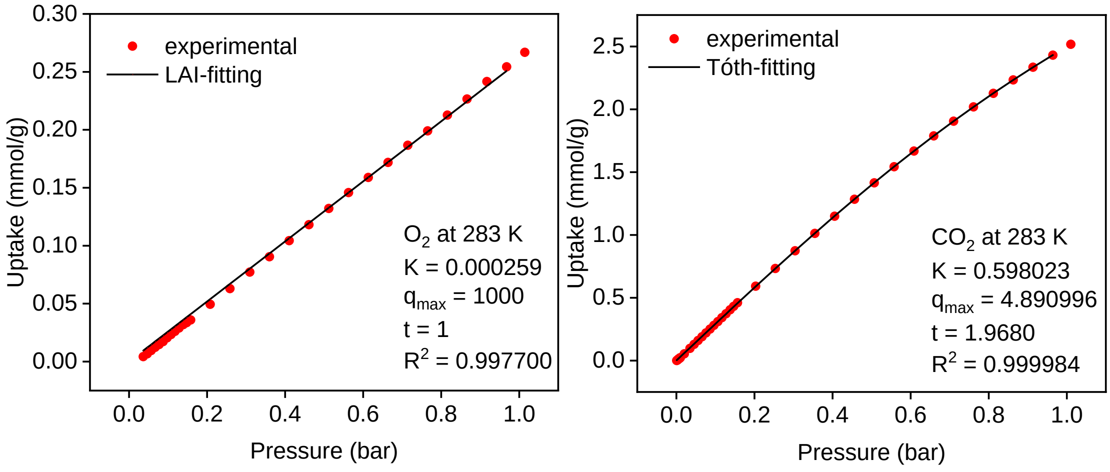

Figure S202. O_2 (left) and CO_2 (right) isotherms and fitting curves of **C_2F_5-cage** at 283 K.

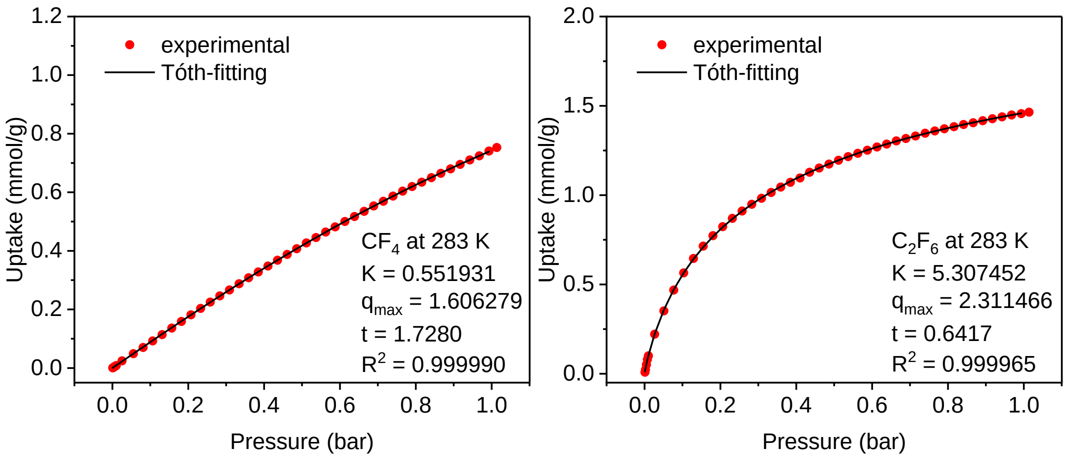

Figure S203. CF_4 (left) and C_2F_6 (right) isotherms and fitting curves of **C_3F_7-cage** at 283 K.

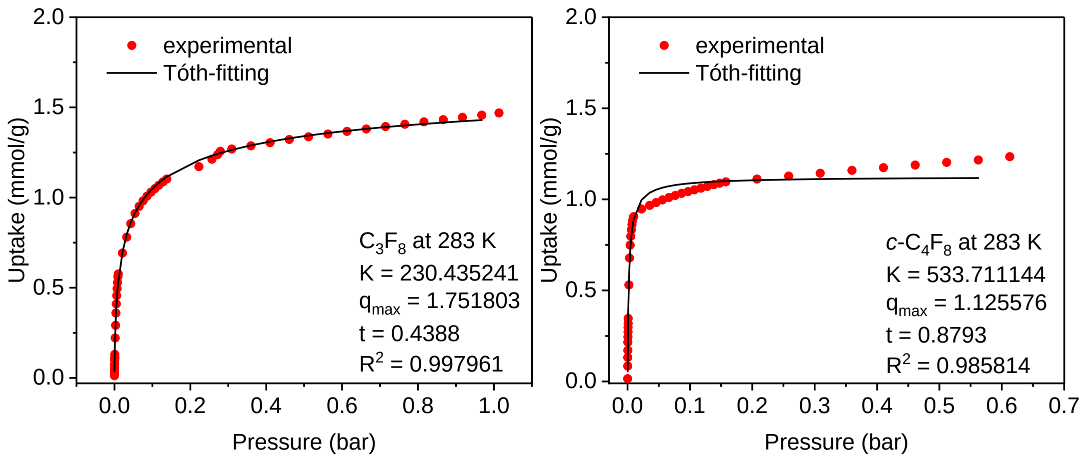

Figure S204. C_3F_8 (left) and *c*-C_4F_8 (right) isotherms and fitting curves of **C_3F_7-cage** at 283 K. (Note: Only the pressure range for from *c*-C_4F_8 0-0.6 bar was taken into account due to pore condensation >0.6 bar)

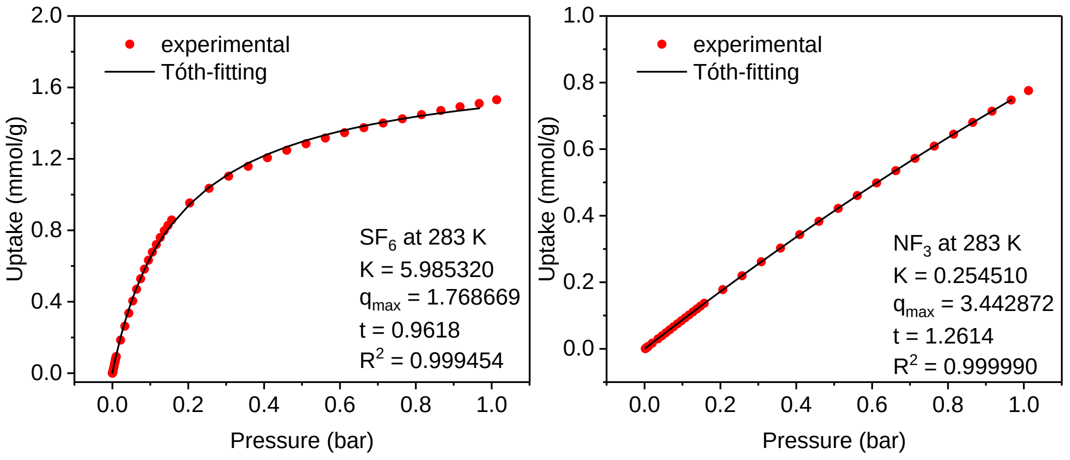

Figure S205. SF_6 (left) and NF_3 (right) isotherms and fitting curves of **C_3F_7-cage** at 283 K.

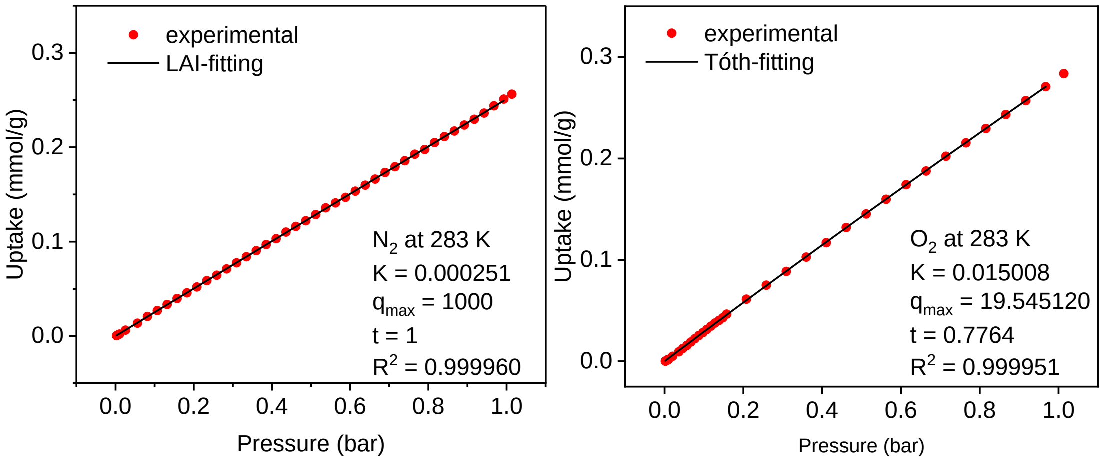

Figure S206. N_2 (left) and O_2 (right) isotherms and fitting curves of **C_3F_7-cage** at 283 K.

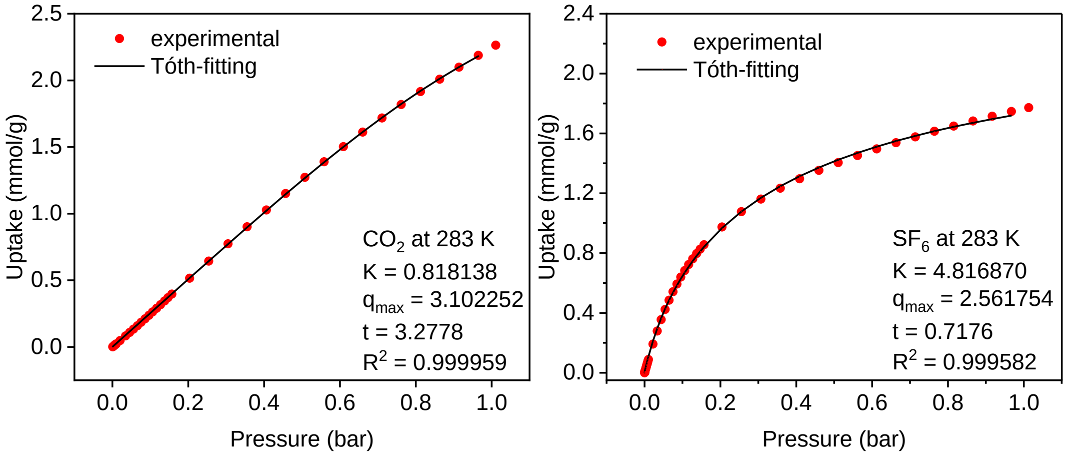

Figure S207. CO_2 isotherm and fitting curve of **C_3F_7-cage** (left) and SF_6 isotherm and fitting curve of **C_4F_9-cage** (right) at 283 K.

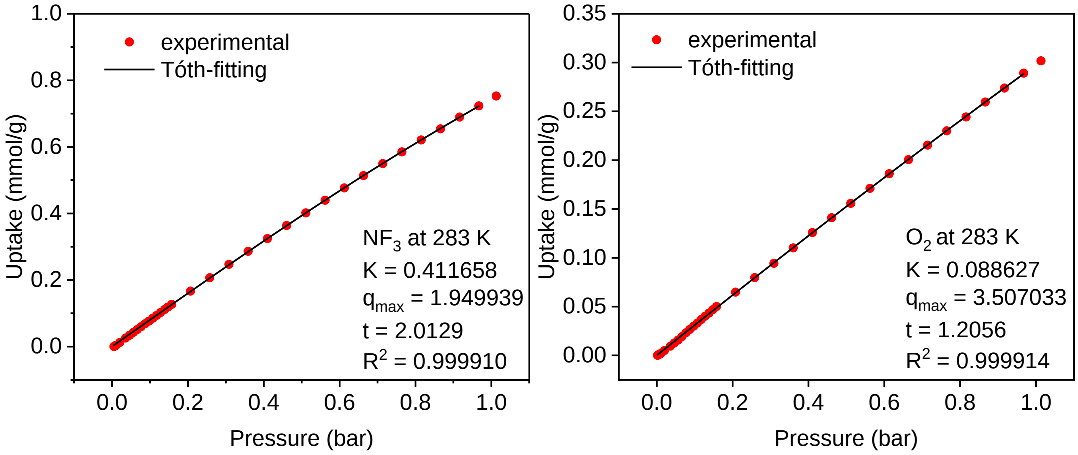

Figure S208. NF_3 (left) and O_2 (right) isotherms and fitting curves of **C_4F_9-cage** at 283 K.

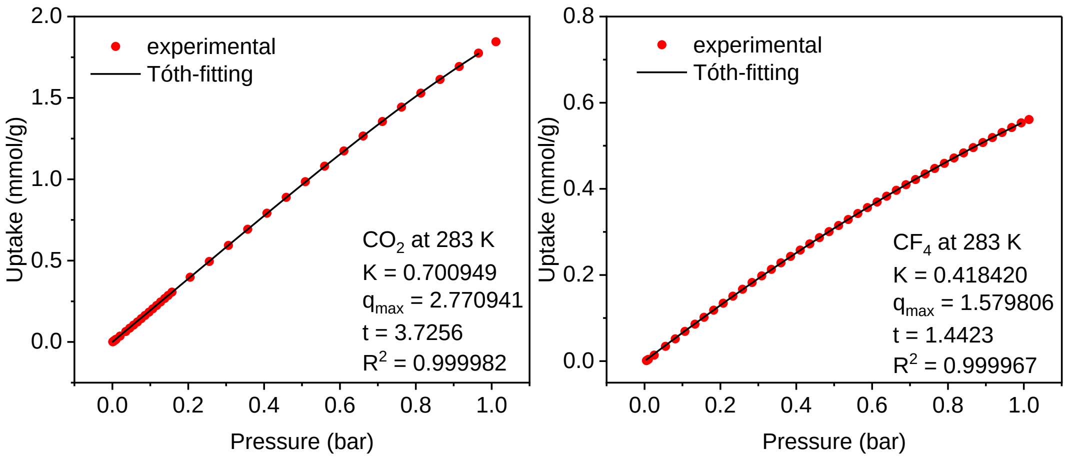

Figure S209. CO_2 isotherm and fitting curve of **C_4F_9-cage** (left) and CF_4 isotherm and fitting curve of **C_5F_{11}-cage** (right) at 283 K.

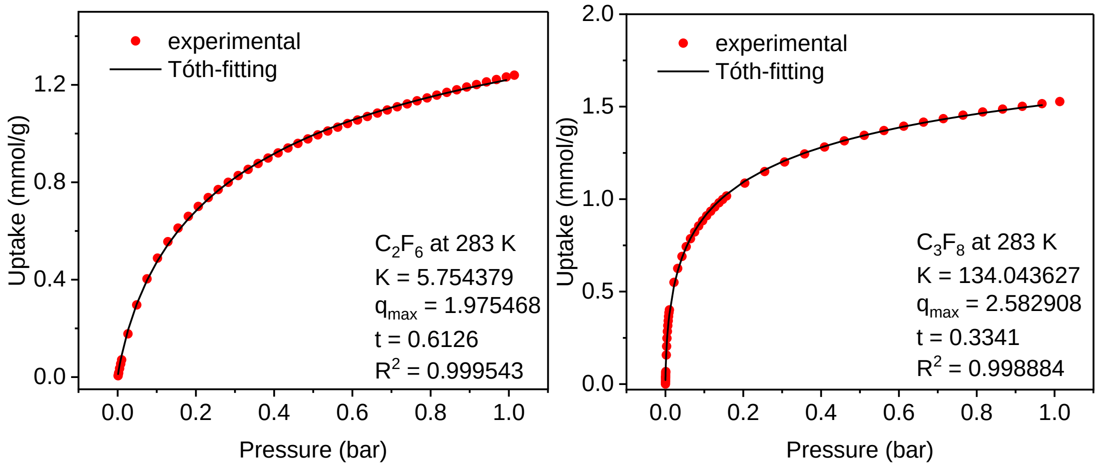

Figure S210. C_2F_6 (left) and C_3F_8 (right) isotherms and fitting curves of **C_5F_{11}-cage** at 283 K.

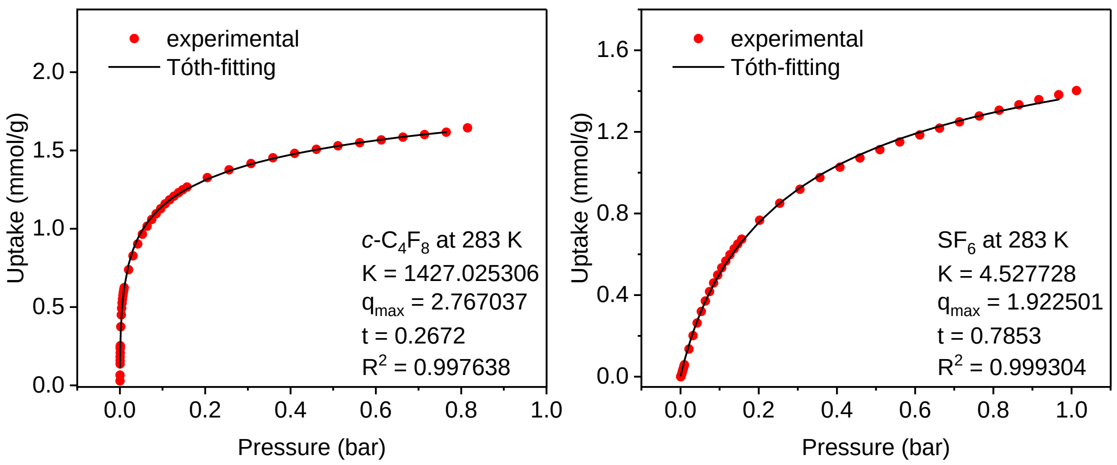

Figure S211. *c*-C_4F_8 (left) and SF_6 (right) isotherms and fitting curves of **C_5F_{11}-cage** at 283 K. (Note: Only the pressure range for from *c*-C_4F_8 0-0.6 bar was taken into account due to pore condensation >0.6 bar)

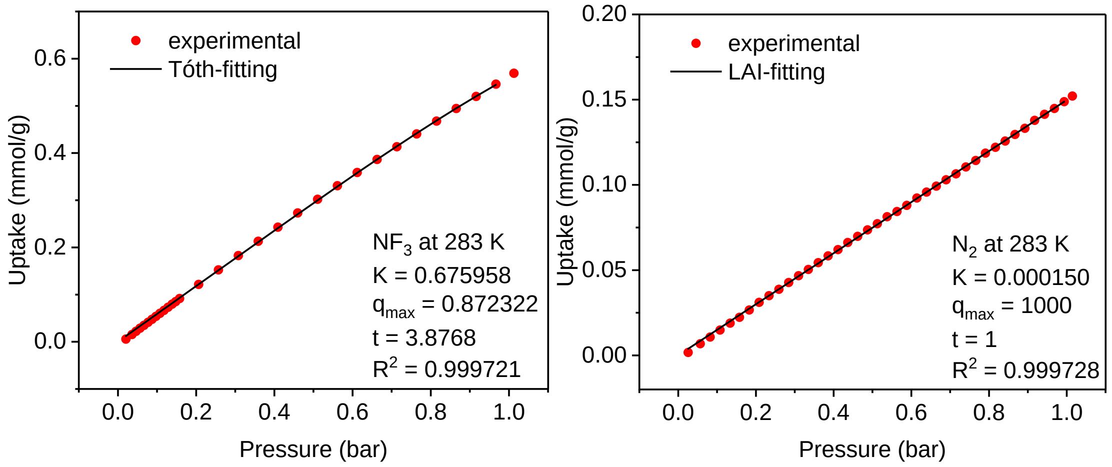

Figure S212. NF_3 (left) and N_2 (right) isotherms and fitting curves of **C_5F_{11}-cage** at 283 K.

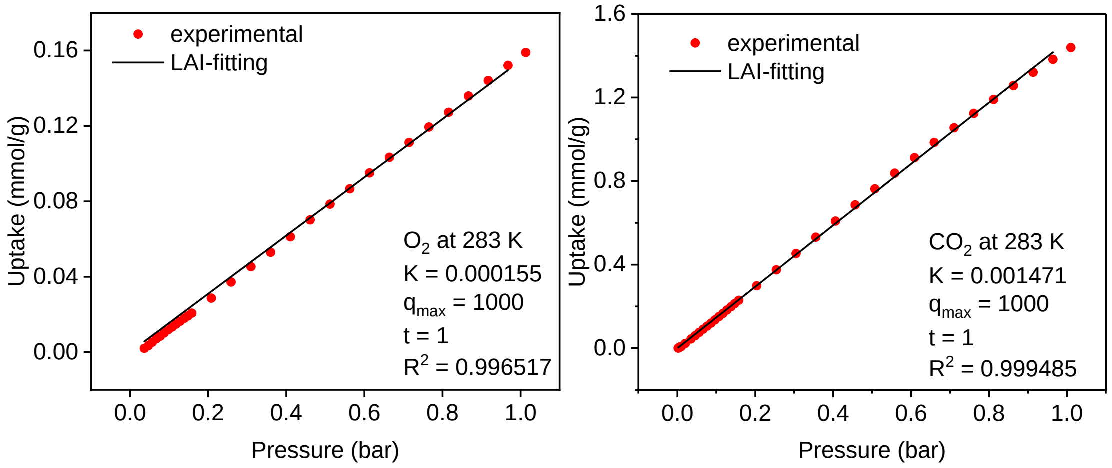

Figure S213. O_2 (left) and CO_2 (right) isotherms and fitting curves of **C_5F_{11}-cage** at 283 K.

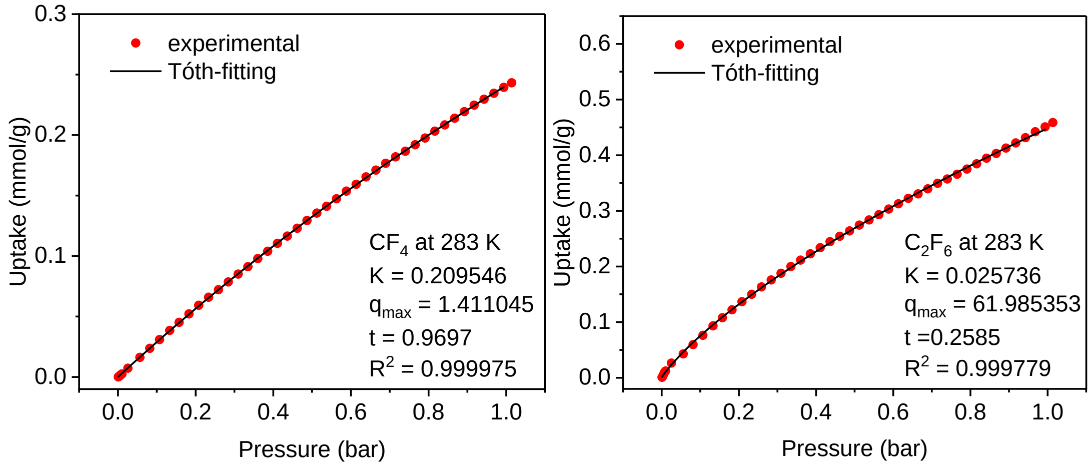

Figure S214. CF_4 (left) and C_2F_6 (right) isotherms and fitting curves of **C_6F_{13}-cage** at 283 K.

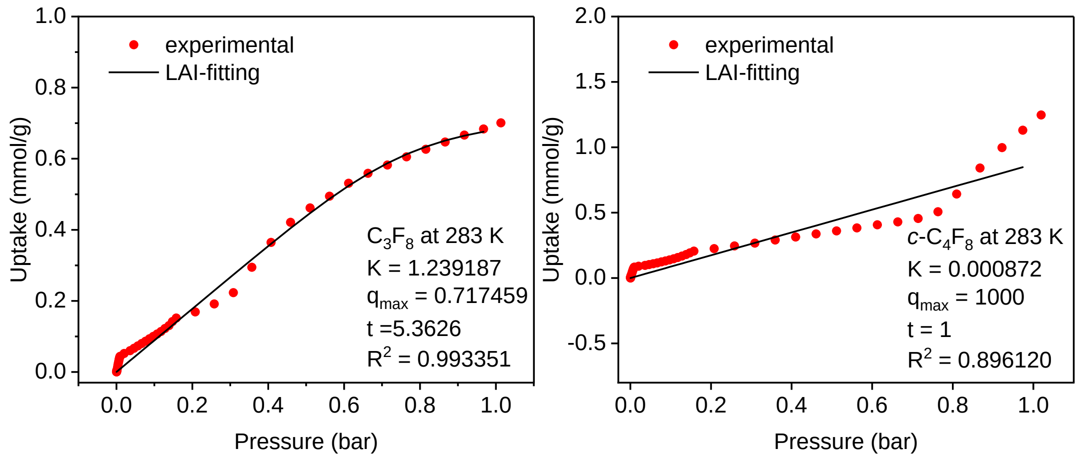

Figure S215. C_3F_8 (left) and c-C_4F_8 (right) isotherms and fitting curves of **C_6F_{13}-cage** at 283 K.

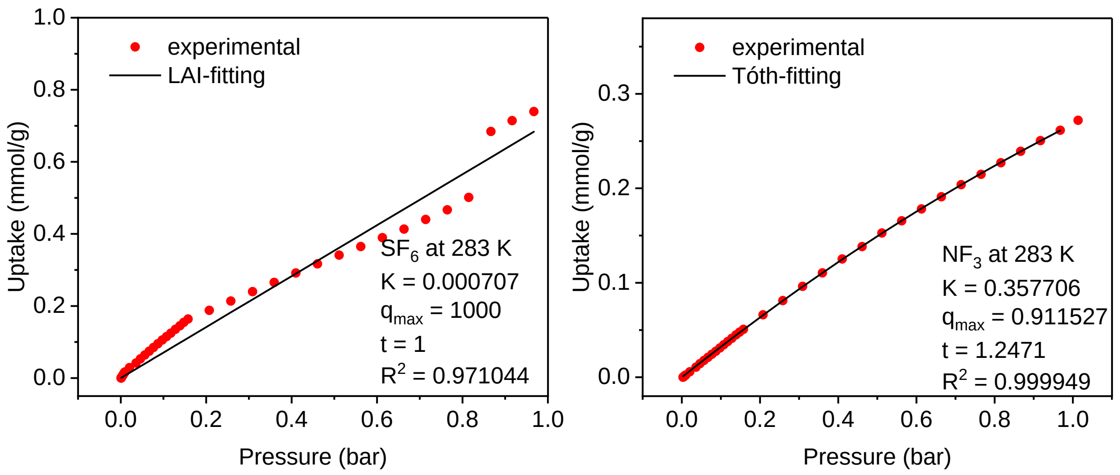

Figure S216. SF_6 (left) and NF_3 (right) isotherms and fitting curves of **C_6F_{13}-cage** at 283 K.

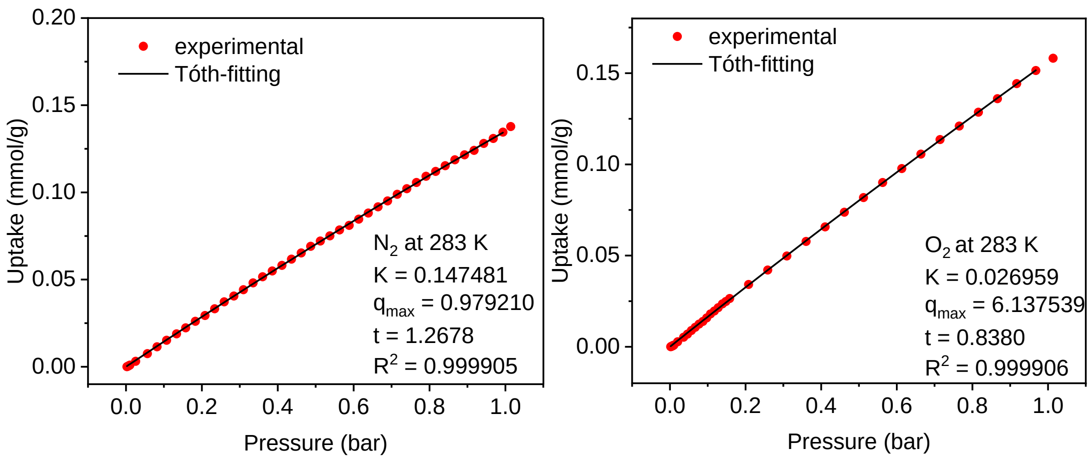

Figure S217. N_2 (left) and O_2 (right) isotherms and fitting curves of **C_6F_{13}-cage** at 283 K.

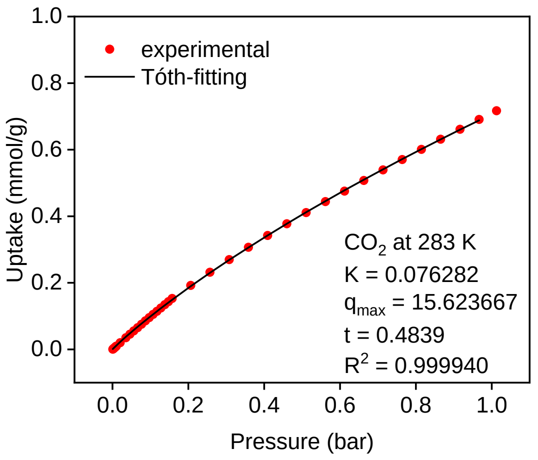

Figure S218. CO_2 isotherm and fitting curve of **C_6F_{13}-cage** at 283 K.

Gas Sorption at 298 K

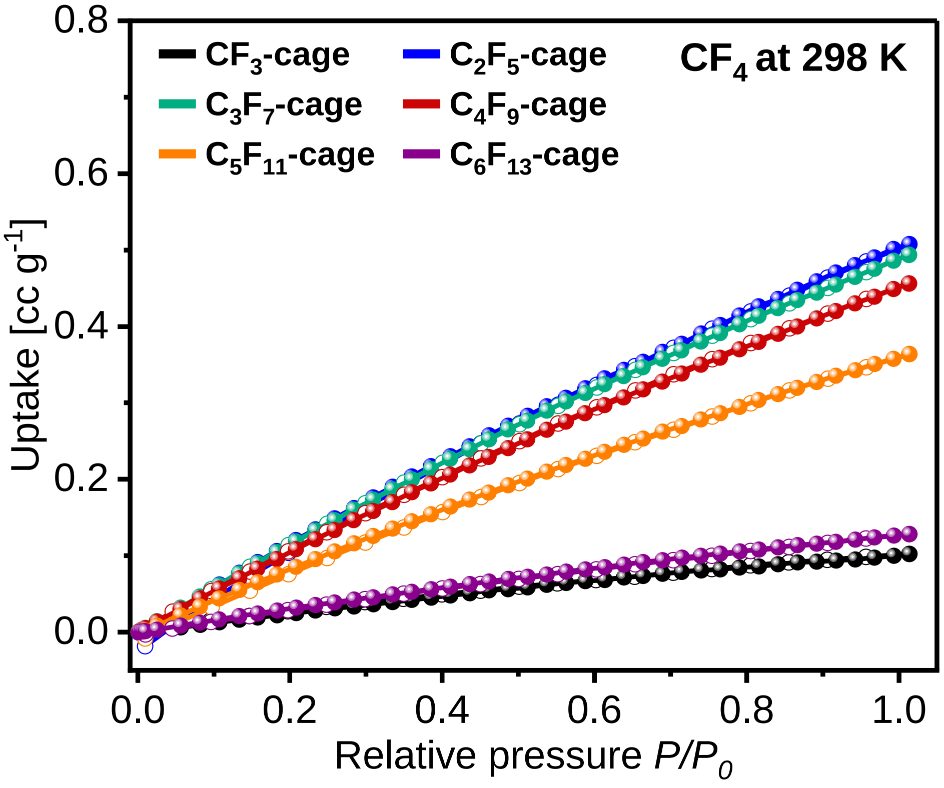

Figure S219. CF_4 sorption isotherms at 298 K. Black: **CF_3-cage**; blue: **C_2F_5-cage**; green: **C_3F_7-cage**; red: **C_4F_8-cage**; orange: **C_5F_{11}-cage**; purple: **C_6F_{13}-cage**. Full circles: adsorption; empty circles: desorption.

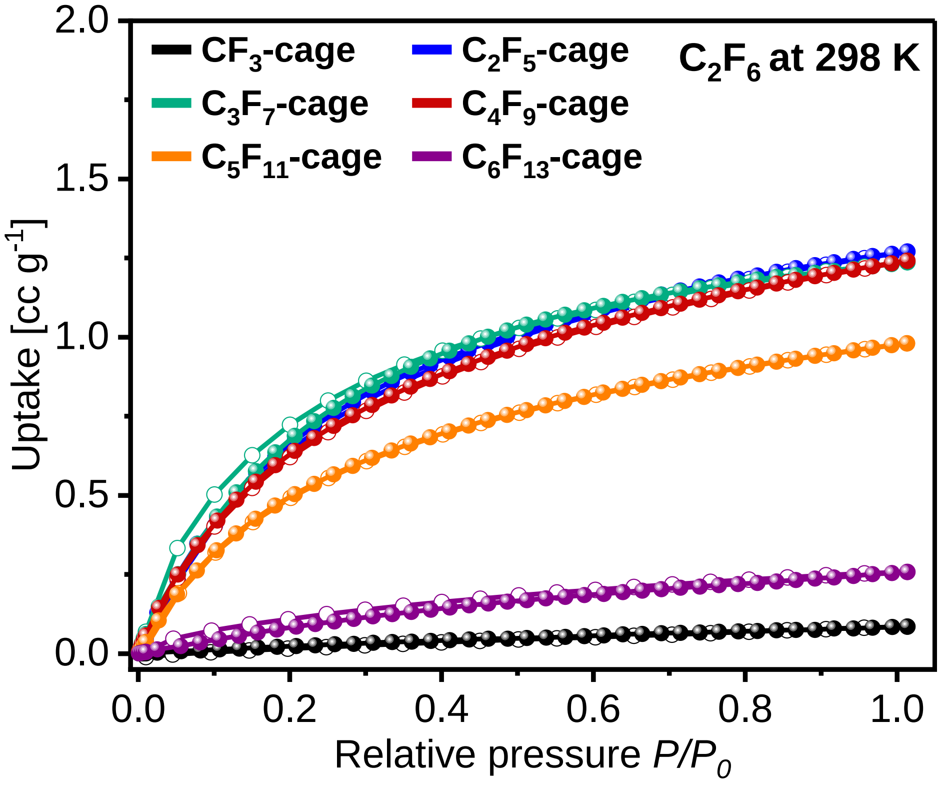

Figure S220. C_2F_6 sorption isotherms at 298 K. Black: **CF_3-cage**; blue: **C_2F_5-cage**; green: **C_3F_7-cage**; red: **C_4F_8-cage**; orange: **C_5F_{11}-cage**; purple: **C_6F_{13}-cage**. Full circles: adsorption; empty circles: desorption.

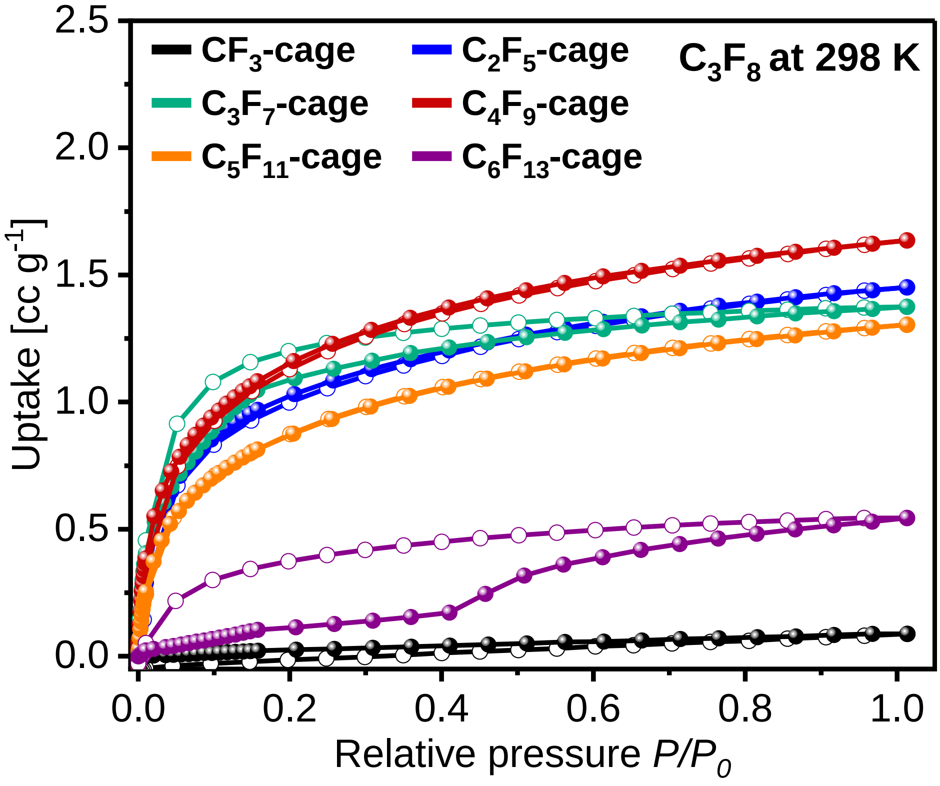

Figure S221. C_3F_8 sorption isotherms at 298 K. Black: **CF_3-cage**; blue: **C_2F_5-cage**; green: **C_3F_7-cage**; red: **C_4F_8-cage**; orange: **C_5F_{11}-cage**; purple: **C_6F_{13}-cage**. Full circles: adsorption; empty circles: desorption.

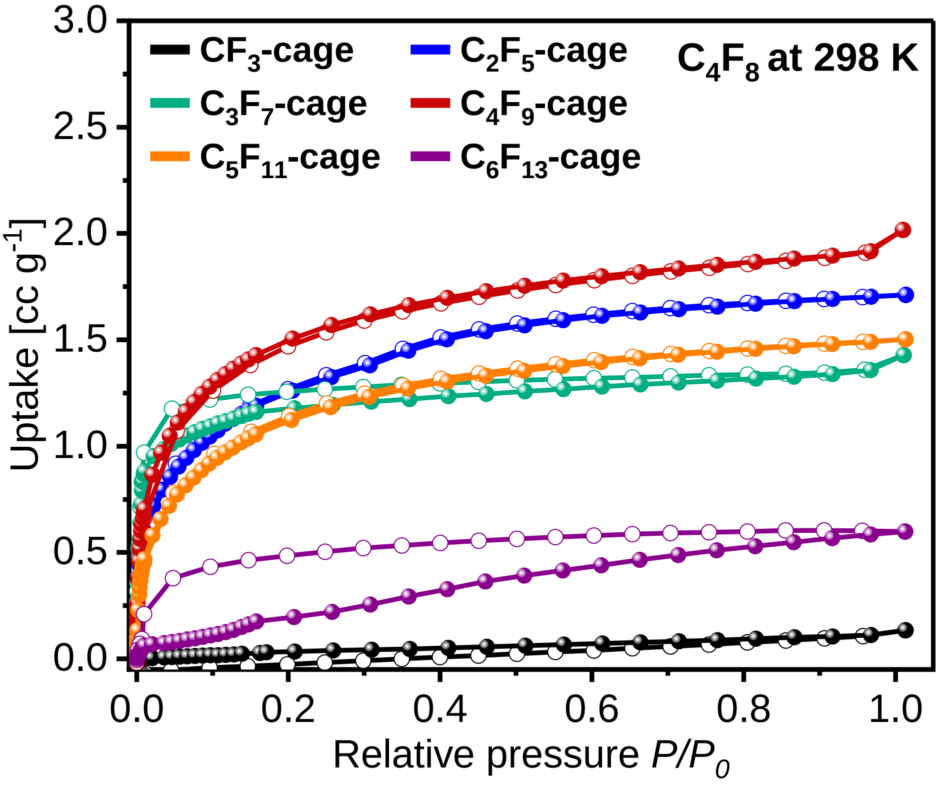

Figure S222. C_4F_8 sorption isotherms at 298 K. Black: **CF_3-cage**; blue: **C_2F_5-cage**; green: **C_3F_7-cage**; red: **C_4F_8-cage**; orange: **C_5F_{11}-cage**; purple: **C_6F_{13}-cage**. Full circles: adsorption; empty circles: desorption.

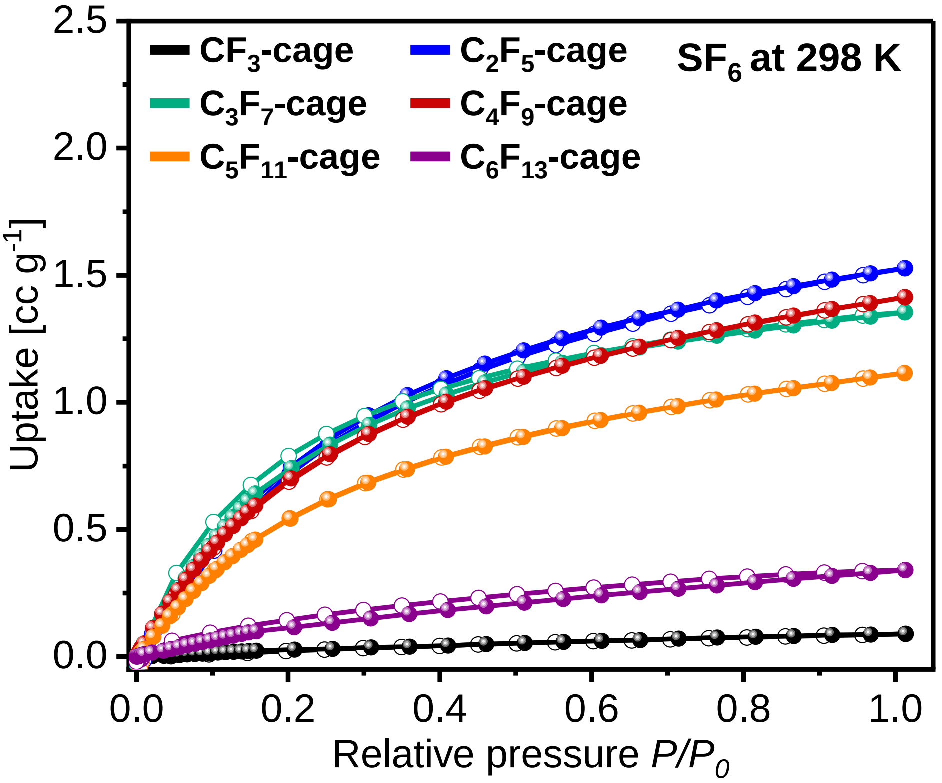

Figure S223. SF_6 sorption isotherms at 298 K. Black: **CF_3-cage**; blue: **C_2F_5-cage**; green: **C_3F_7-cage**; red: **C_4F_8-cage**; orange: **C_5F_{11}-cage**; purple: **C_6F_{13}-cage**. Full circles: adsorption; empty circles: desorption.

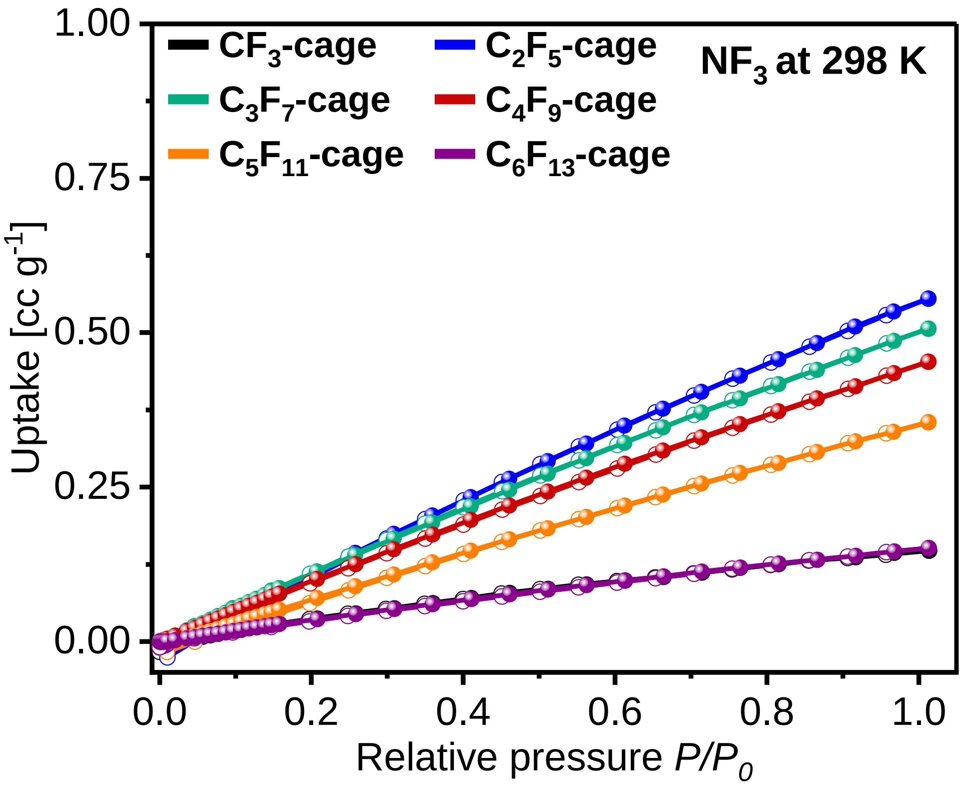

Figure S224. NF_3 sorption isotherms at 298 K. Black: **CF_3-cage**; blue: **C_2F_5-cage**; green: **C_3F_7-cage**; red: **C_4F_8-cage**; orange: **C_5F_{11}-cage**; purple: **C_6F_{13}-cage**. Full circles: adsorption; empty circles: desorption.

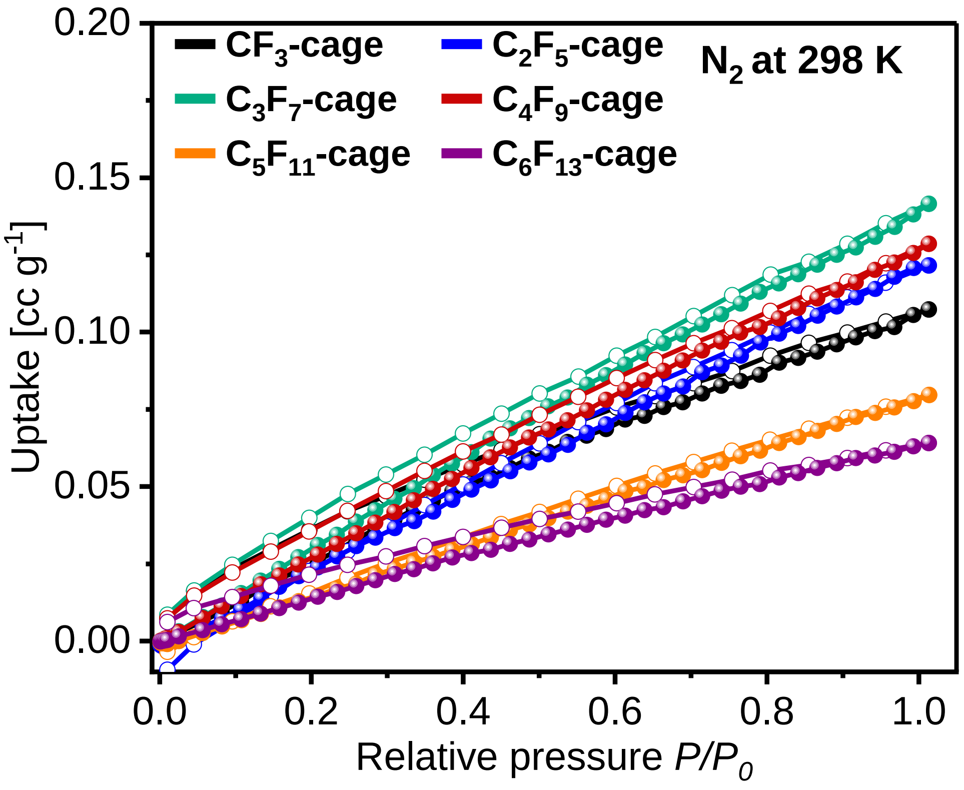

Figure S225. N_2 sorption isotherms at 298 K. Black: **CF_3-cage**; blue: **C_2F_5-cage**; green: **C_3F_7-cage**; red: **C_4F_8-cage**; orange: **C_5F_{11}-cage**; purple: **C_6F_{13}-cage**. Full circles: adsorption; empty circles: desorption.

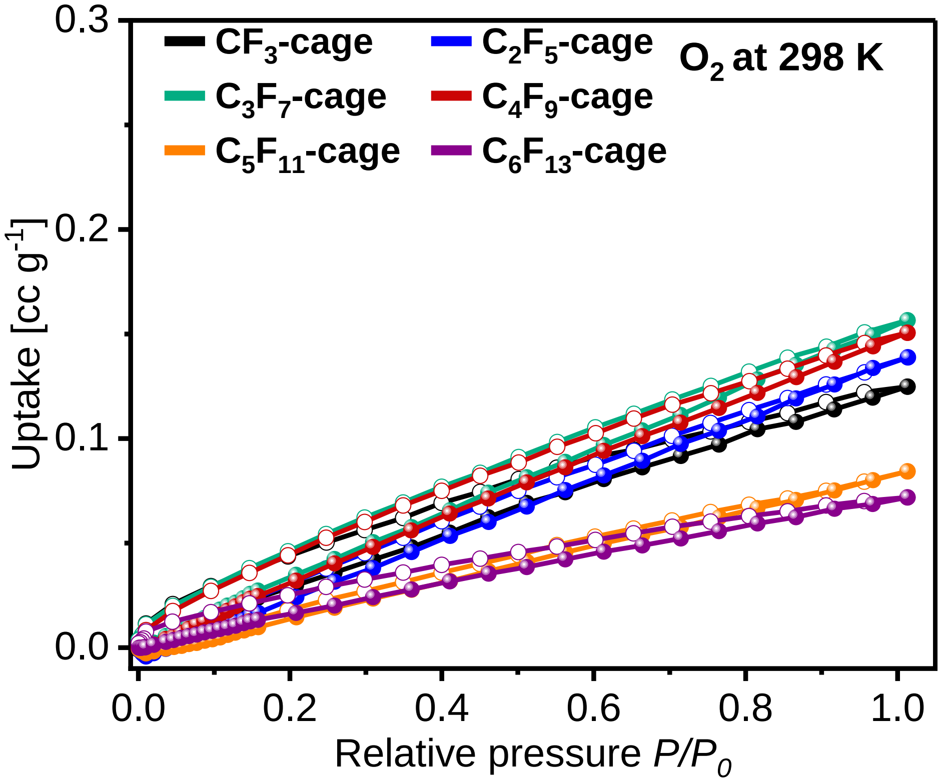

Figure S226. O_2 sorption isotherms at 298 K. Black: **CF_3-cage**; blue: **C_2F_5-cage**; green: **C_3F_7-cage**; red: **C_4F_8-cage**; orange: **C_5F_{11}-cage**; purple: **C_6F_{13}-cage**. Full circles: adsorption; empty circles: desorption.

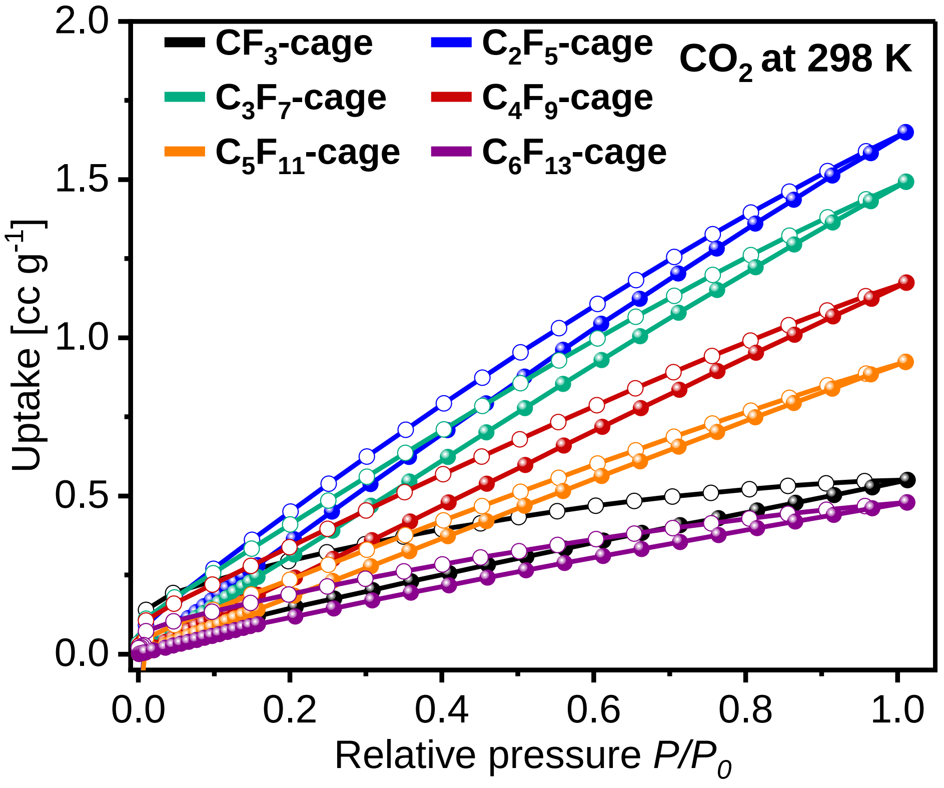

Figure S227. CO_2 sorption isotherms at 298 K. Black: **CF_3-cage**; blue: **C_2F_5-cage**; green: **C_3F_7-cage**; red: **C_4F_8-cage**; orange: **C_5F_{11}-cage**; purple: **C_6F_{13}-cage**. Full circles: adsorption; empty circles: desorption.

Table S13. Fitting and IAST parameters of Tóth and LAI isotherms as well as R^2 -values and Henry constants at 298 K.

Cages	Gas	Affinity const. *K* [1/bar]	Max. uptake *q*max [mmol/g]	Heterogenity Parameter	R^2	*Model*	K_H
**CF_3-cage**	CF_4	0.850296	0.139306	2.7578	0.999778	Tóth	0.118451335
	C_2F_6	0.170242	0.825927	0.5788	0.999380	Tóth	0.140607464
	C_3F_8	0.000094	1000	1	0.986629	LAI	0.094
	c-C_4F_8	0.000117	1000	1	0.987248	LAI	0.117
	SF_6	0.136811	1.146133	0.5269	0.997205	Tóth	0.156803602
	NF_3	0.567979	0.310577	1.6958	0.999089	Tóth	0.176401214
	N_2	0.504992	0.260533	1.4028	0.999439	Tóth	0.131567081
	O_2	0.099299	1.528161	0.7575	0.999573	Tóth	0.151744859
	CO_2	0.005244	225.331802	0.2721	0.999726	Tóth	1.18163997
**C_2F_5-cage**	CF_4	0.326651	1.803608	1.3161	0.999919	Tóth	0.589150357
	C_2F_6	3.499437	1.749462	0.8787	0.999908	Tóth	6.122132053
	C_3F_8	38.957936	1.867406	0.5143	0.998754	Tóth	72.75028343
	c-C_4F_8	1004.438627	3.564064	0.2401	0.994988	Tóth	3579.883551
	SF_6	2.673166	1.92104	1.1516	0.999676	Tóth	5.135258813
	NF_3	0.000560	1000	1	0.998406	LAI	0.56
	N_2	0.000120	1000	1	0.998635	LAI	0.12
	O_2	0.000134	1000	1	0.990129	LAI	0.134
	CO_2	0.357166	4.944781	1.8253	0.999963	Tóth	1.766107651
**C_3F_7-cage**	CF_4	0.358753	1.635699	1.2804	0.999981	Tóth	0.586811923
	C_2F_6	3.654933	1.491902	1.1120	0.999958	Tóth	5.452801853
	C_3F_8	84.307189	1.764594	0.4682	0.998467	Tóth	148.7679599
	c-C_4F_8	493.206605	1.327921	0.6051	0.988462	Tóth	654.9394081
	SF_6	3.499626	1.585550	1.1874	0.999798	Tóth	5.548832004
	NF_3	0.403285	1.344989	1.9266	0.999951	Tóth	0.542413889
	N_2	0.000143	1000	1	0.998066	LAI	0.143
	O_2	0.000157	1000	1	0.999350	LAI	0.157
	CO_2	0.652520	2.339628	4.3115	0.999986	Tóth	1.526654063
**C_4F_9-cage**	SF_6	2.947954	1.999962	0.8929	0.999904	Tóth	5.895795978
	NF_3	0.361158	1.351230	1.7351	0.999976	Tóth	0.488007524
	O_2	0.000151	1000	1	0.999346	LAI	0.151
	CO_2	0.769543	1.522797	8.6377	0.999722	Tóth	1.171857772
**C_5F_{11}-cage**	CF_4	0.323811	1.290617	1.3325	0.999940	Tóth	0.417915981
	C_2F_6	3.374284	1.512821	0.7557	0.999785	Tóth	5.104687695
	C_3F_8	45.461665	2.136778	0.3953	0.999256	Tóth	97.14148562
	c-C_4F_8	406.573776	2.734229	0.2824	0.998325	Tóth	1111.665809
	SF_6	2.774246	1.521278	0.9667	0.999659	Tóth	4.220399406
	NF_3	0.000354	1000	1	0.998138	LAI	0.354
	N_2	0.000078	1000	1	0.998214	LAI	0.078
	O_2	0.000080	1000	1	0.988874	LAI	0.08
	CO_2	0.000917	1000	1	0.999793	LAI	0.917
**C_6F_{13}-cage**	CF_4	0.429452	0.357446	1.3926	0.999912	Tóth	0.1535059
	C_2F_6	0.914142	0.523446	1.0093	0.999731	Tóth	0.478503973
	C_3F_8	0.000580	1000	1	0.984279	LAI	0.58
	c-C_4F_8	0.000686	1000	1	0.960270	LAI	0.686
	SF_6	0.227727	5.474476	0.3522	0.999243	Tóth	1.246685996
	NF_3	0.404280	0.428583	1.4960	0.999768	Tóth	0.173267535
	N_2	0.310284	0.224044	1.5623	0.999702	Tóth	0.069517268
	O_2	0.047015	1.799040	0.6751	0.999757	Tóth	0.084581866
	CO_2	0.046559	14.011624	0.5408	0.999931	Tóth	0.652367202

Gas sorption fitting curves at 298 K

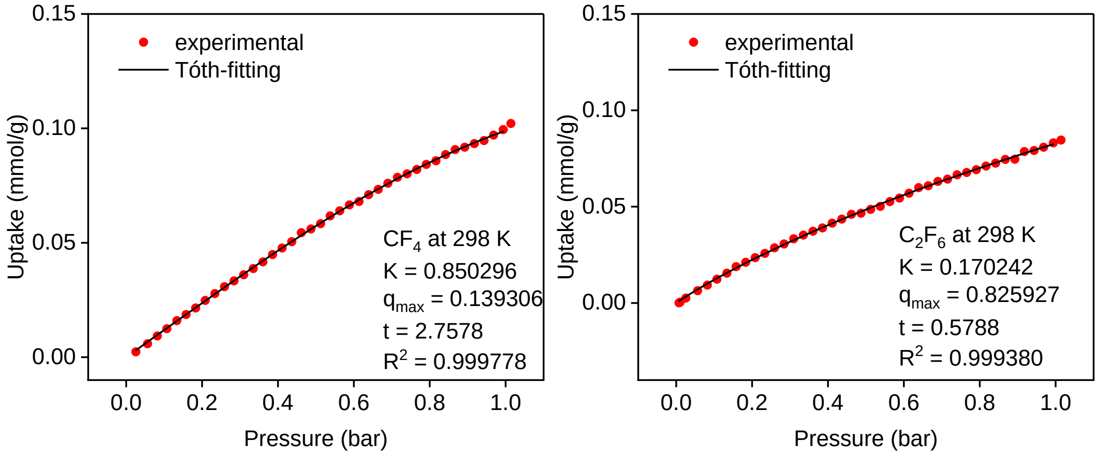

Figure S228. CF_4 (left) and C_2F_6 (right) isotherms and fitting curves of **CF_3-cage** at 298 K.

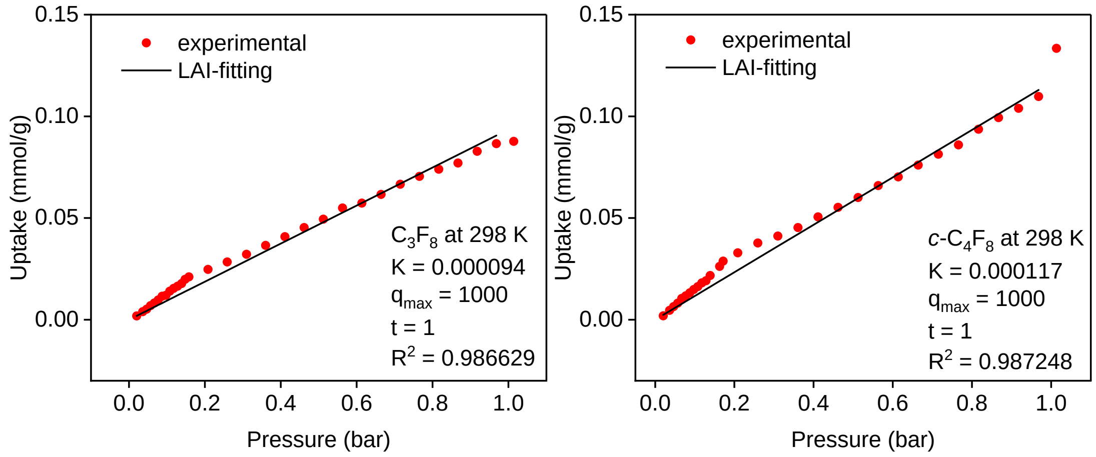

Figure S229. C_3F_8 (left) and *c*-C_4F_8 (right) isotherms and fitting curves of **CF_3-cage** at 298 K.

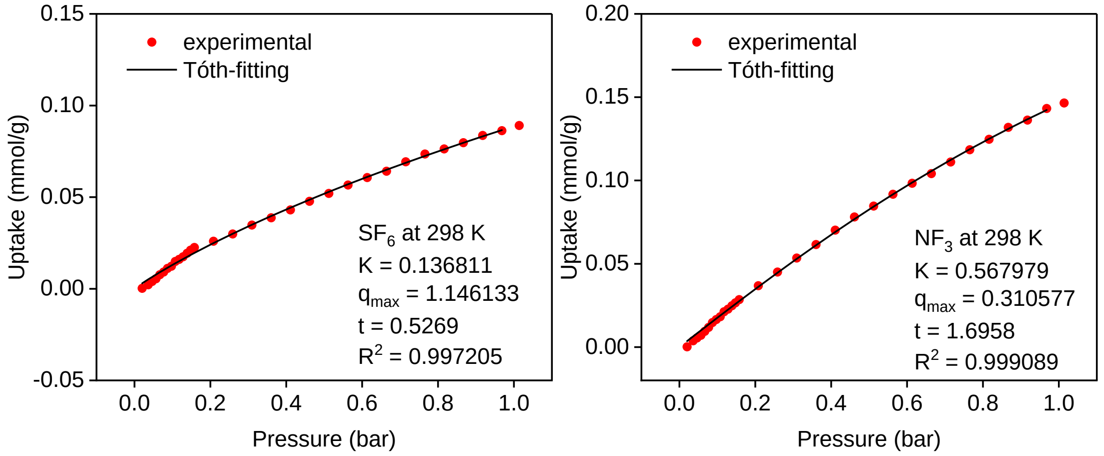

Figure S230. SF_6 (left) and NF_3 (right) isotherms and fitting curves of **CF_3-cage** at 298 K.

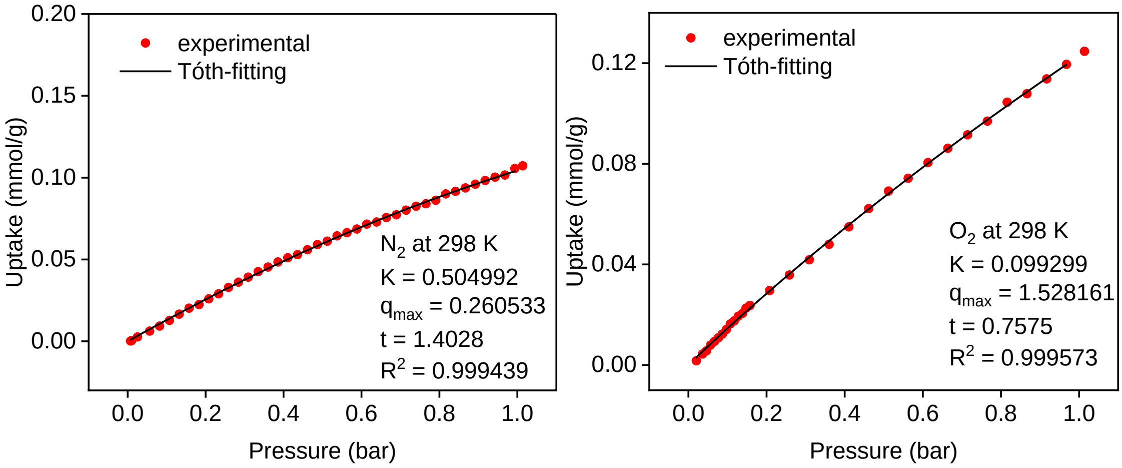

Figure S231. N_2 (left) and O_2 (right) isotherms and fitting curves of **CF_3-cage** at 298 K.

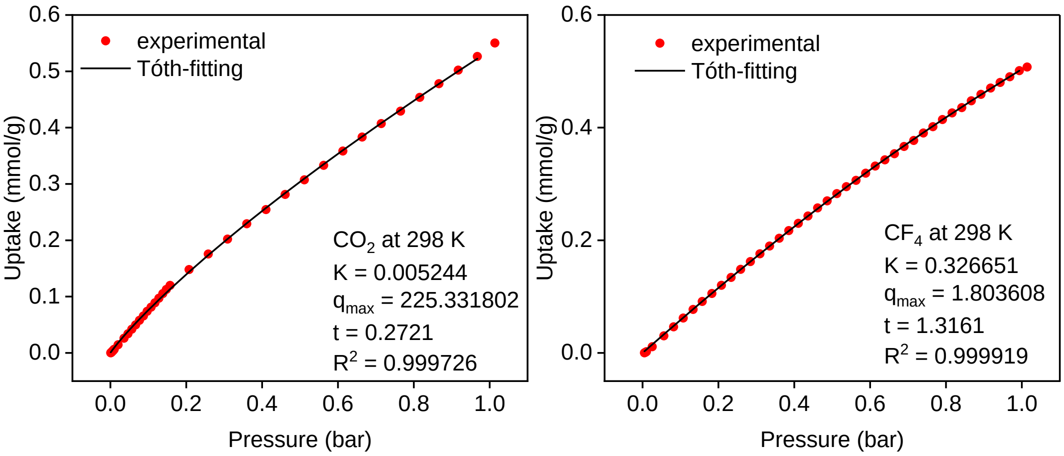

Figure S232. CO_2 isotherm and fitting curve of **CF_3-cage** (left) and CF_4 isotherm and fitting curve of **C_2F_6-cage** (right) at 298 K.

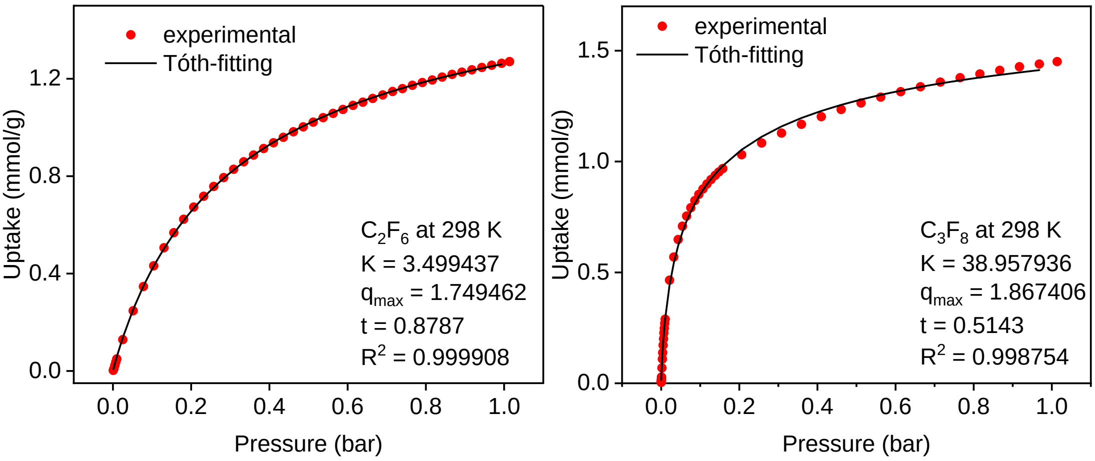

Figure S233. C_2F_6 (left) and C_3F_8 (right) isotherms and fitting curves of **C_2F_6-cage** at 298 K.

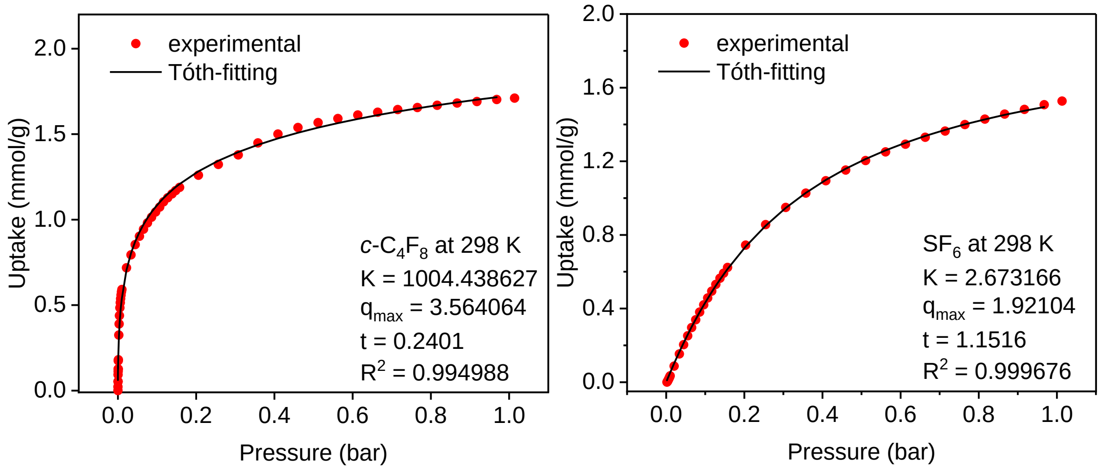

Figure S234. *c*-C_4F_8 (left) and SF_6 (right) isotherms and fitting curves of **C_2F_6-cage** at 298 K.

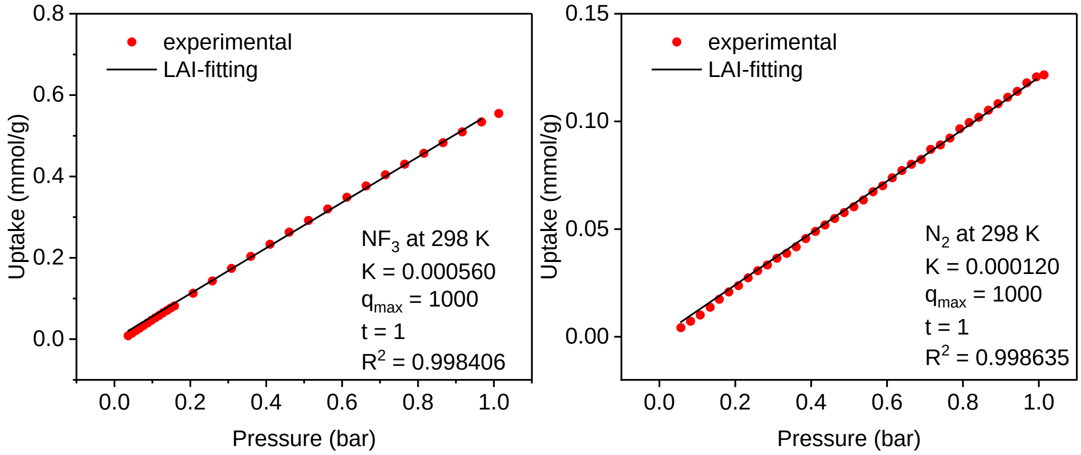

Figure S235. NF_3 (left) and N_2 (right) isotherms and fitting curves of **C_2F_6-cage** at 298 K.

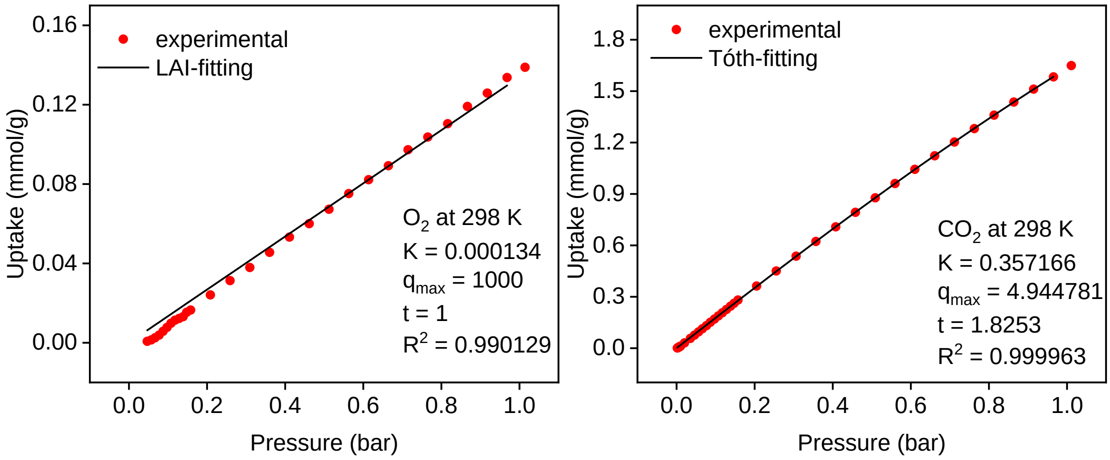

Figure S236. O_2 (left) and CO_2 (right) isotherms and fitting curves of **C_2F_6-cage** at 298 K.

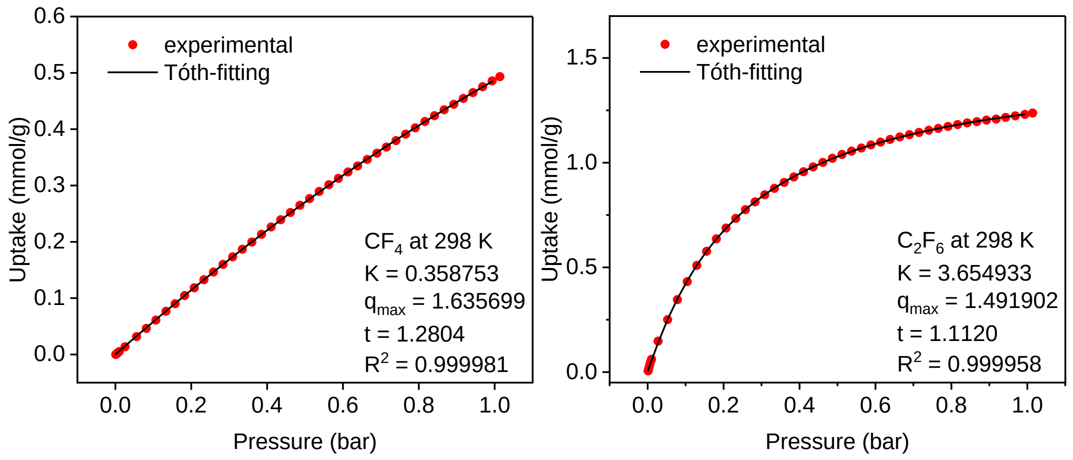

Figure S237. CF_4 (left) and C_2F_6 (right) isotherms and fitting curves of **C_3F_7-cage** at 298 K.

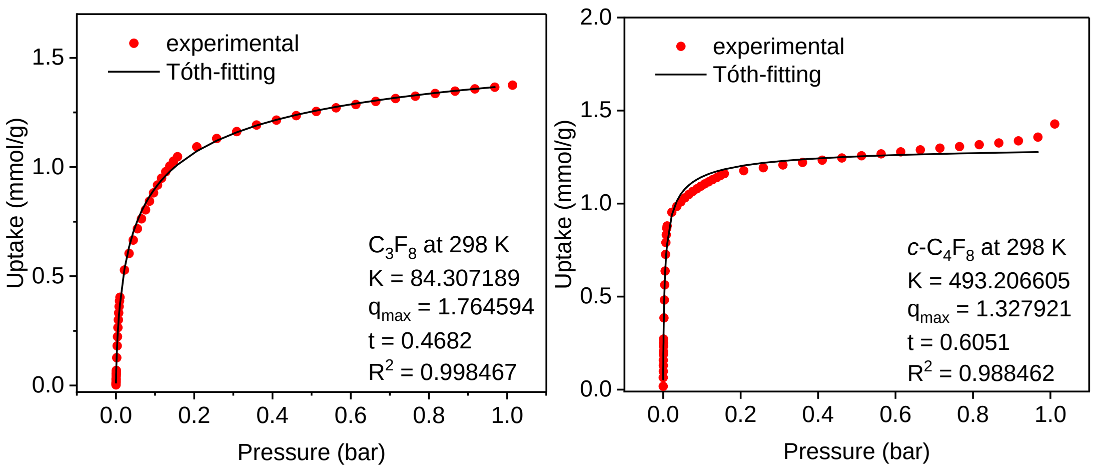

Figure S238. C_3F_8 (left) and *c*-C_4F_8 (right) isotherms and fitting curves of **C_3F_7-cage** at 298 K.

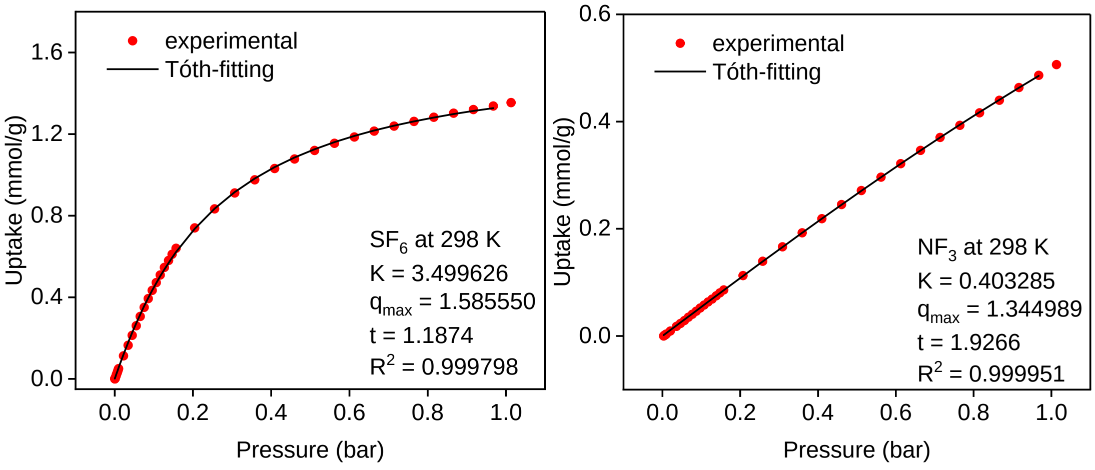

Figure S239. SF_6 (left) and NF_3 (right) isotherms and fitting curves of **C_3F_7-cage** at 298 K.

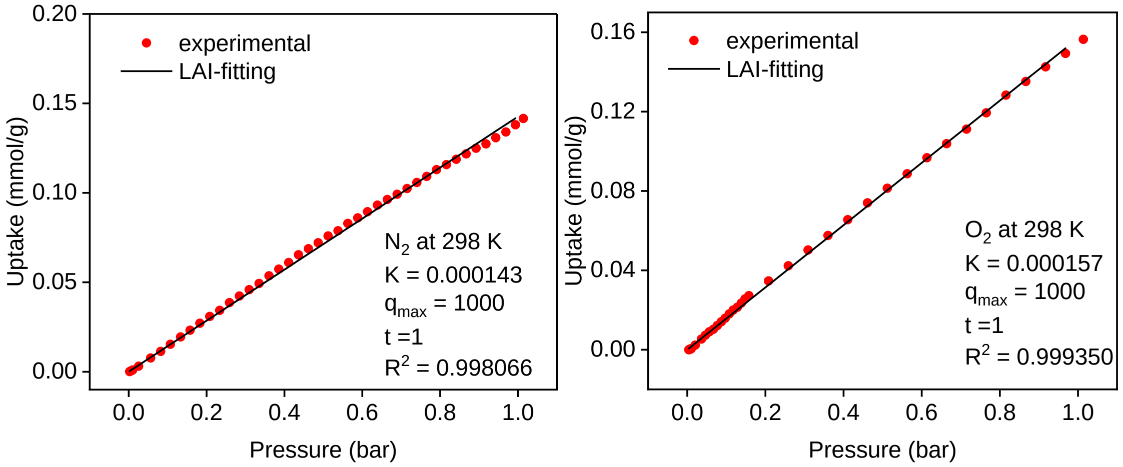

Figure S240. N_2 (left) and O_2 (right) isotherms and fitting curves of **C_3F_7-cage** at 298 K.

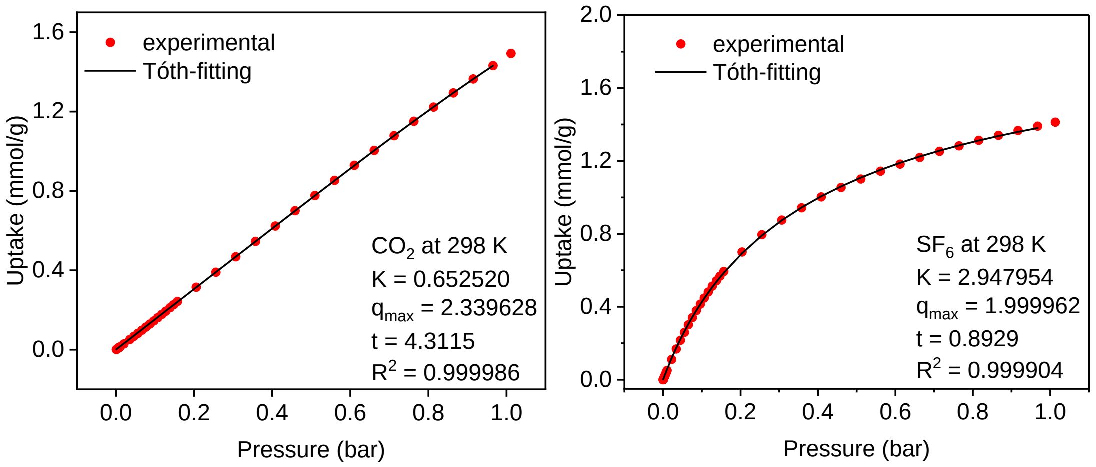

Figure S241. CO_2 isotherm and fitting curve of **C_3F_7-cage** (left) and SF_6 isotherm and fitting curve of **C_4F_9-cage** (right) at 298 K.

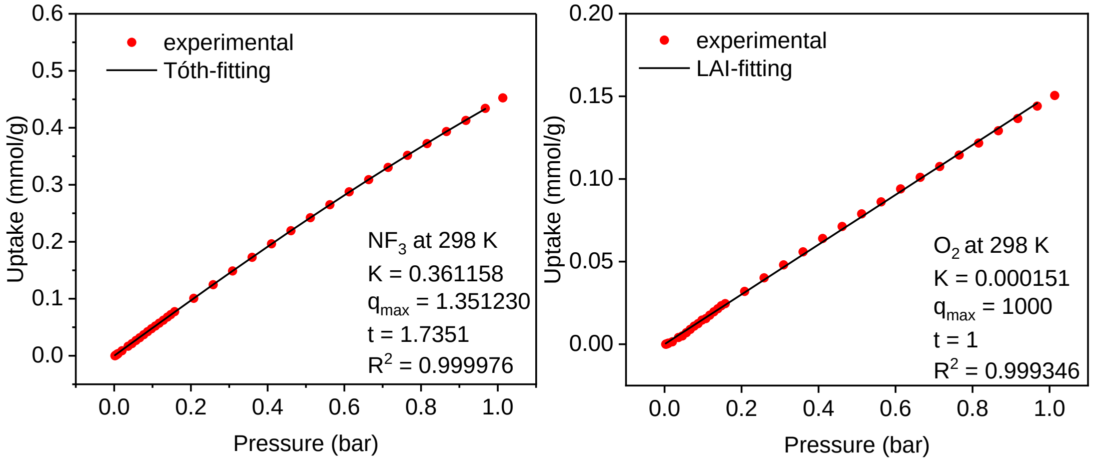

Figure S242. NF_3 (left) and O_2 (right) isotherms and fitting curves of **C_4F_9-cage** at 298 K.

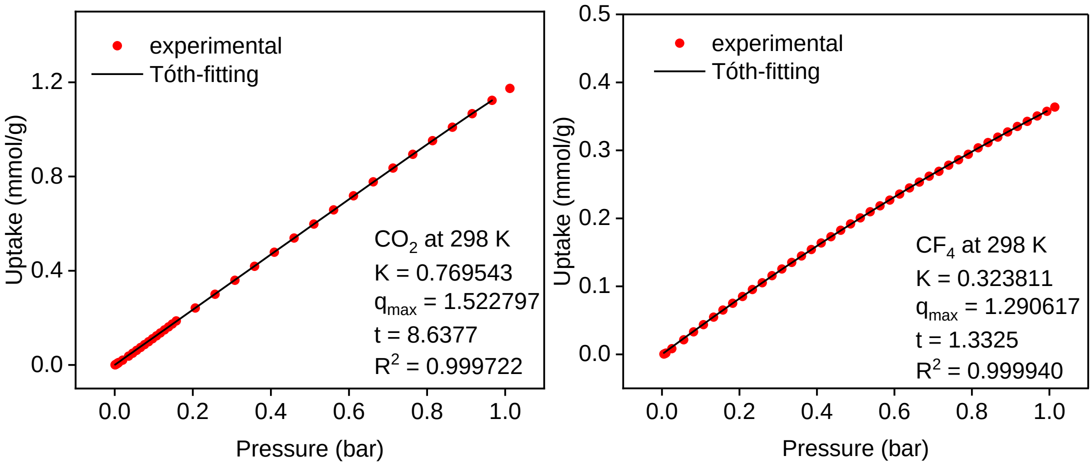

Figure S243. CO_2 isotherm and fitting curve of **C_4F_9-cage** (left) and CF_4 isotherm and fitting curve of **C_5F_{11}-cage** (right) at 298 K.

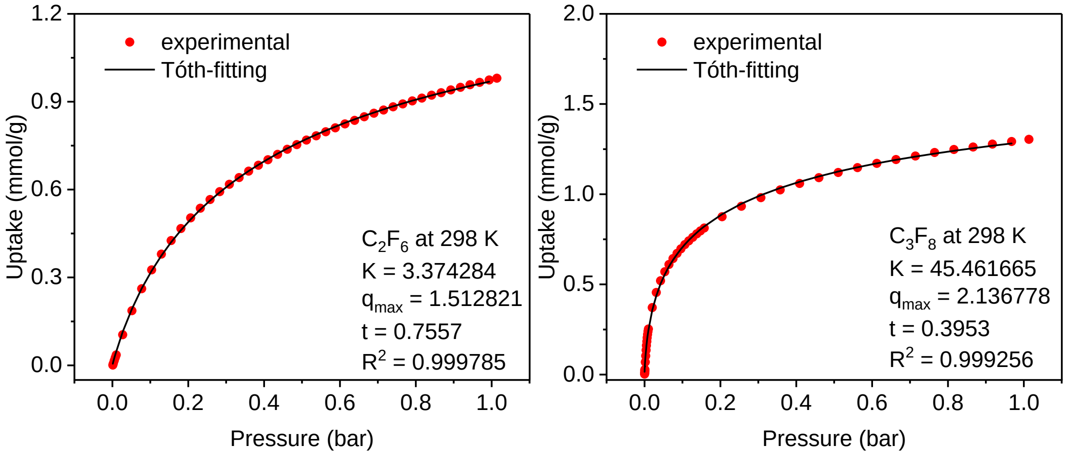

Figure S244. C_2F_6 (left) and C_3F_8 (right) isotherms and fitting curves of **C_5F_{11}-cage** at 298 K.

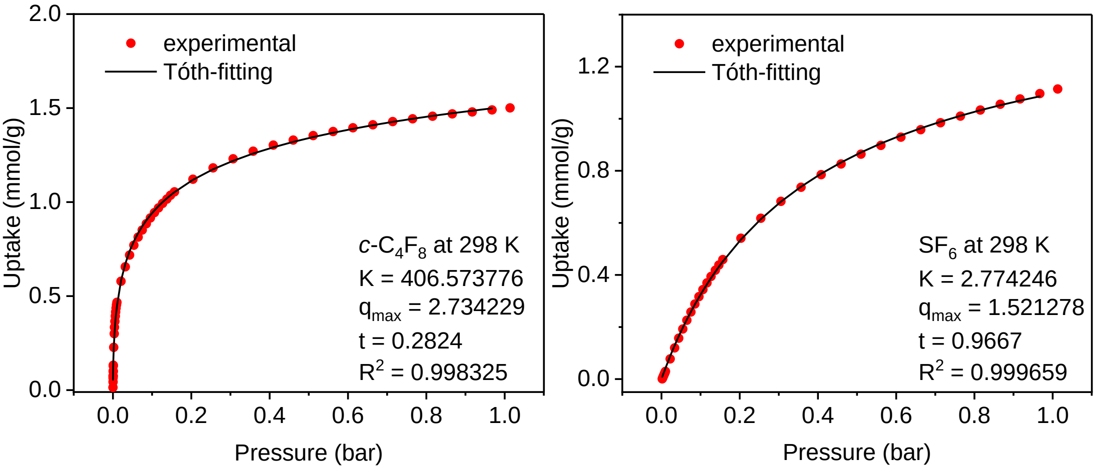

Figure S245. *c*-C_4F_8 (left) and SF_6 (right) isotherms and fitting curves of **C_5F_{11}-cage** at 298 K.

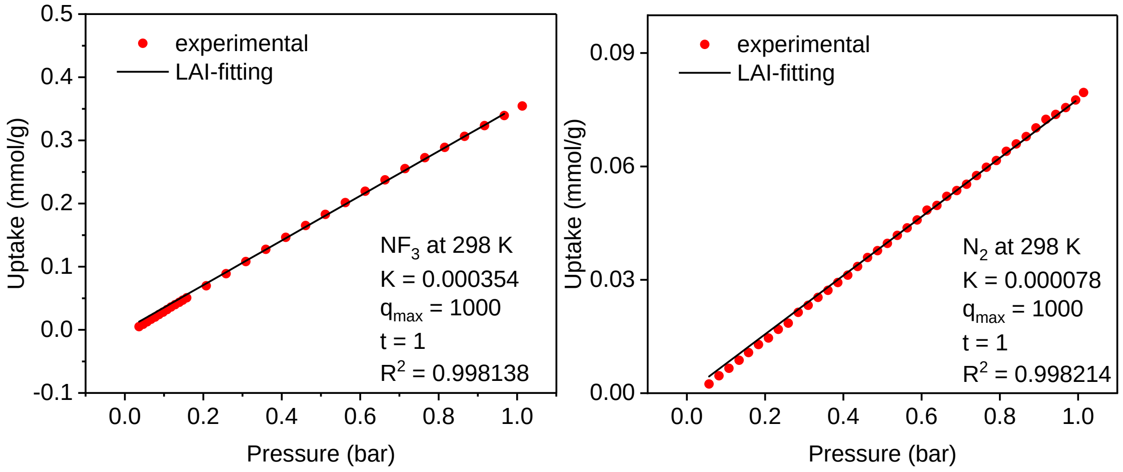

Figure S246. NF_3 (left) and N_2 (right) isotherms and fitting curves of **C_5F_{11}-cage** at 298 K.

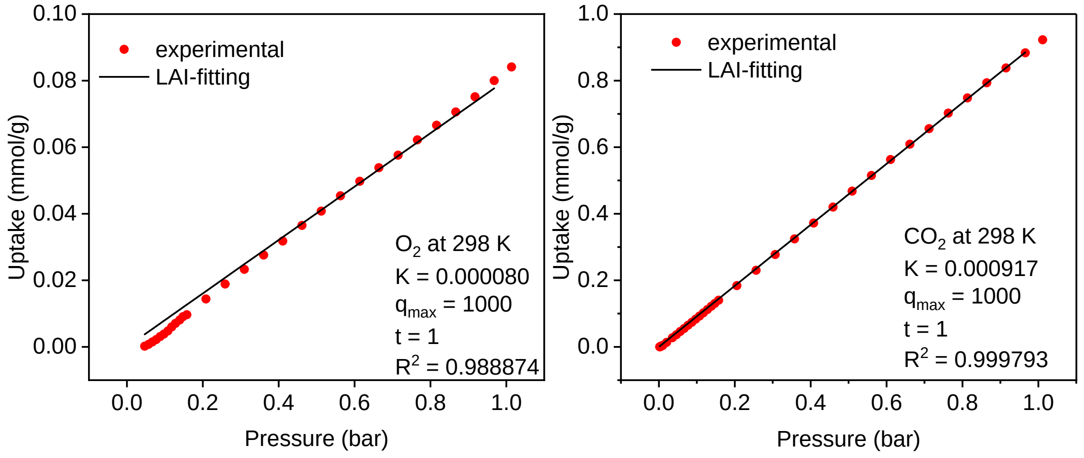

Figure S247. O_2 (left) and CO_2 (right) isotherms and fitting curves of **C_5F_{11}-cage** at 298 K.

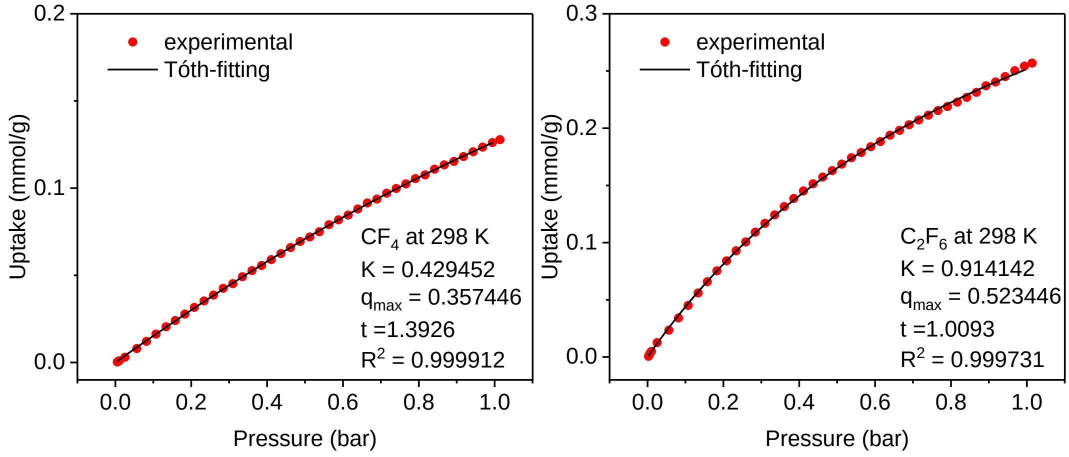

Figure S248. CF_4 (left) and C_2F_6 (right) isotherms and fitting curves of **C_6F_{13}-cage** at 298 K.

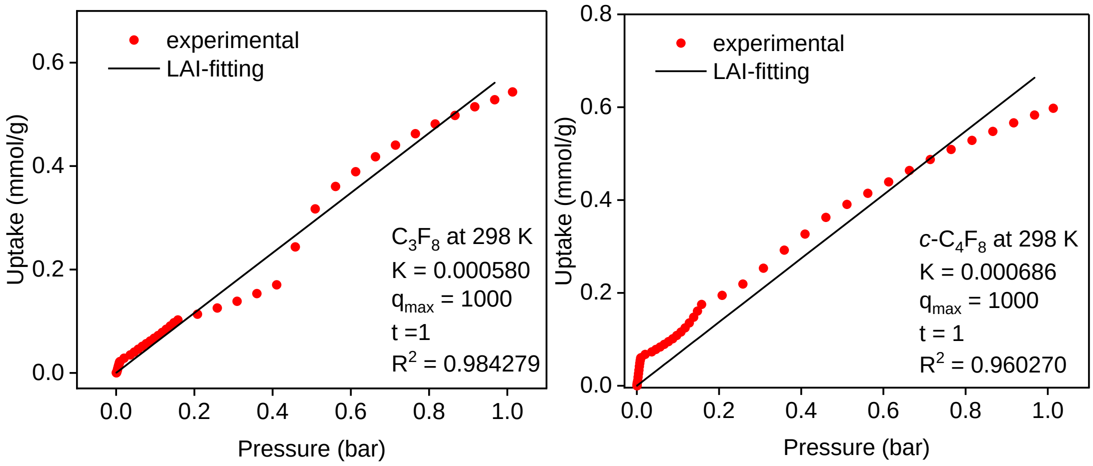

Figure S249. C_3F_8 (left) and *c*-C_4F_8 (right) isotherms and fitting curves of **C_6F_{13}-cage** at 298 K.

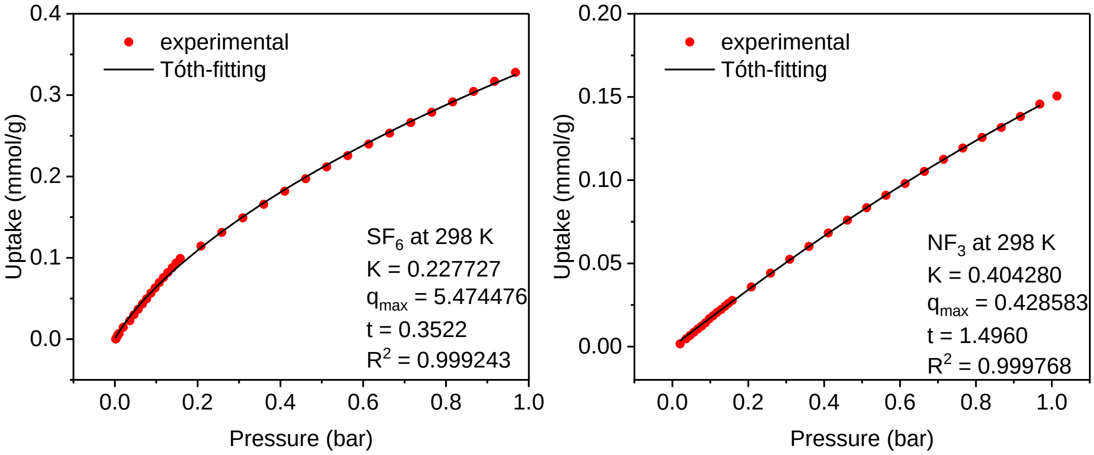

Figure S250. SF_6 (left) and NF_3 (right) isotherms and fitting curves of **C_6F_{13}-cage** at 298 K.

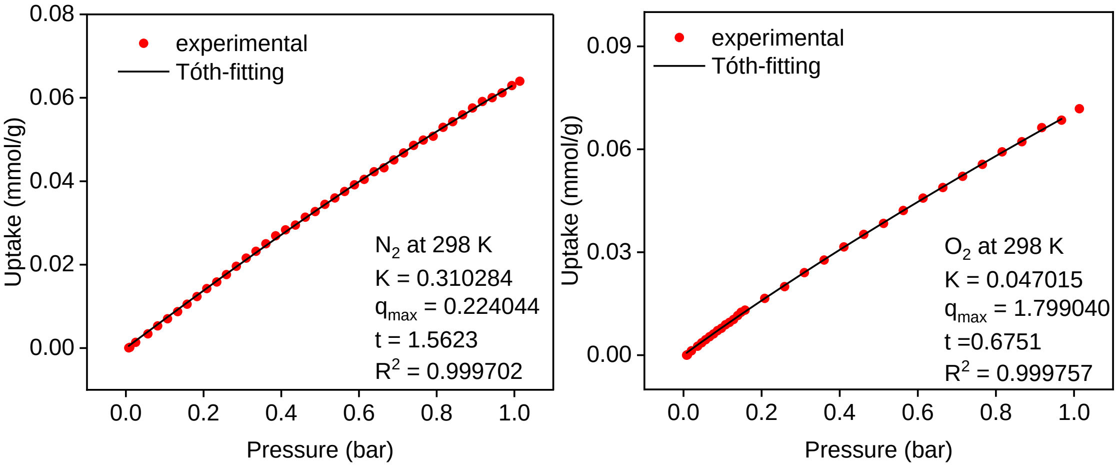

Figure S251. N_2 (left) and O_2 (right) isotherms and fitting curves of **C_6F_{13}-cage** at 298 K.

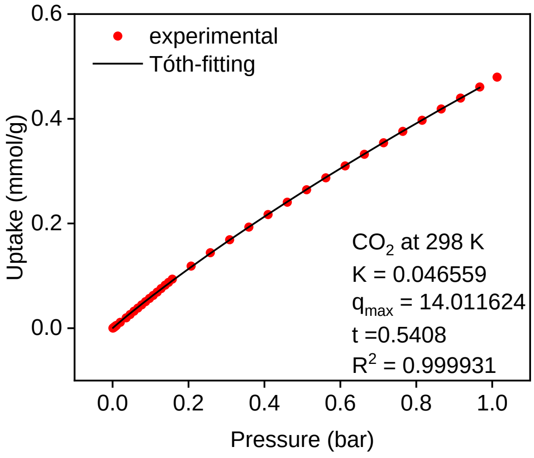

Figure S252. CO_2 isotherm and fitting curve of **C_6F_{13}-cage** at 298 K.

Gas Sorption at 313 K

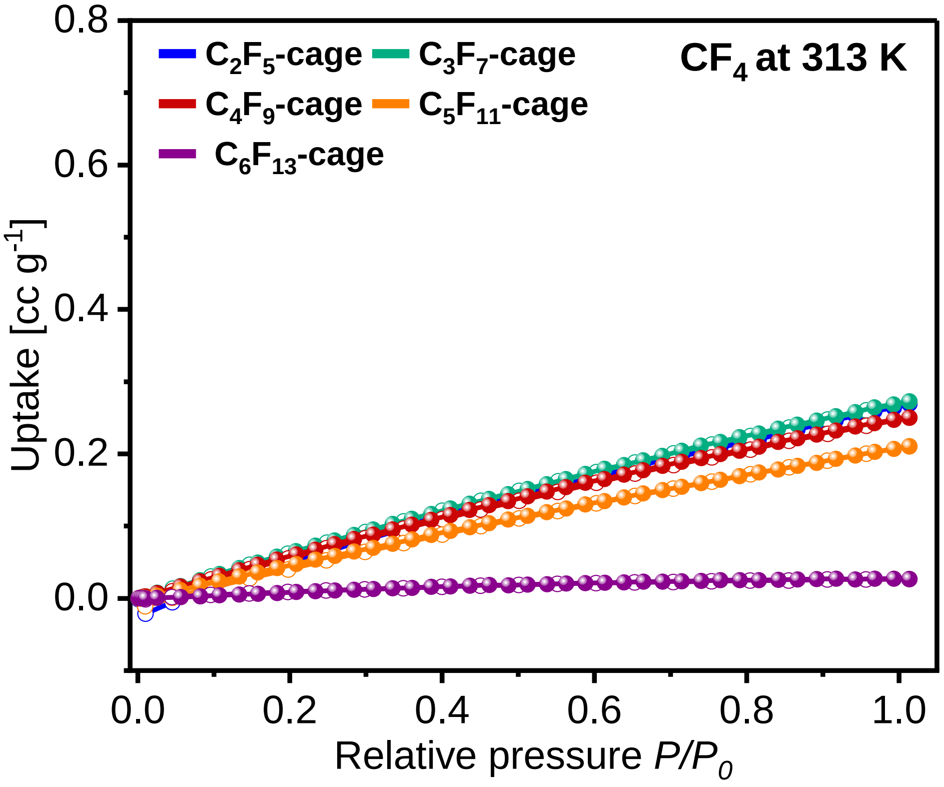

Figure S253. CF_4 sorption isotherms at 313 K. blue: **C_2F_5-cage**; green: **C_3F_7-cage**; red: **C_4F_8-cage**; orange: **C_5F_{11}-cage**; purple: **C_6F_{13}-cage**. Full circles: adsorption; empty circles: desorption. Note that the adsorption of **CF_3-cage** was too low to be measured.

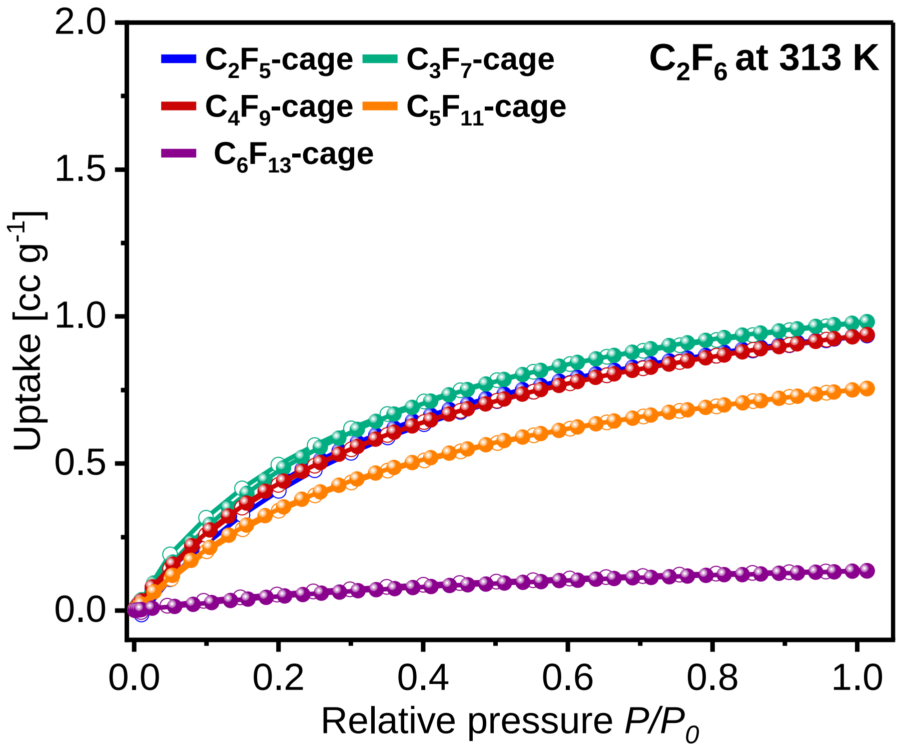

Figure S254. C_2F_6 sorption isotherms at 313 K. blue: **C_2F_5-cage**; green: **C_3F_7-cage**; red: **C_4F_8-cage**; orange: **C_5F_{11}-cage**; purple: **C_6F_{13}-cage**. Full circles: adsorption; empty circles: desorption. Note that the adsorption of **CF_3-cage** was too low to be measured.

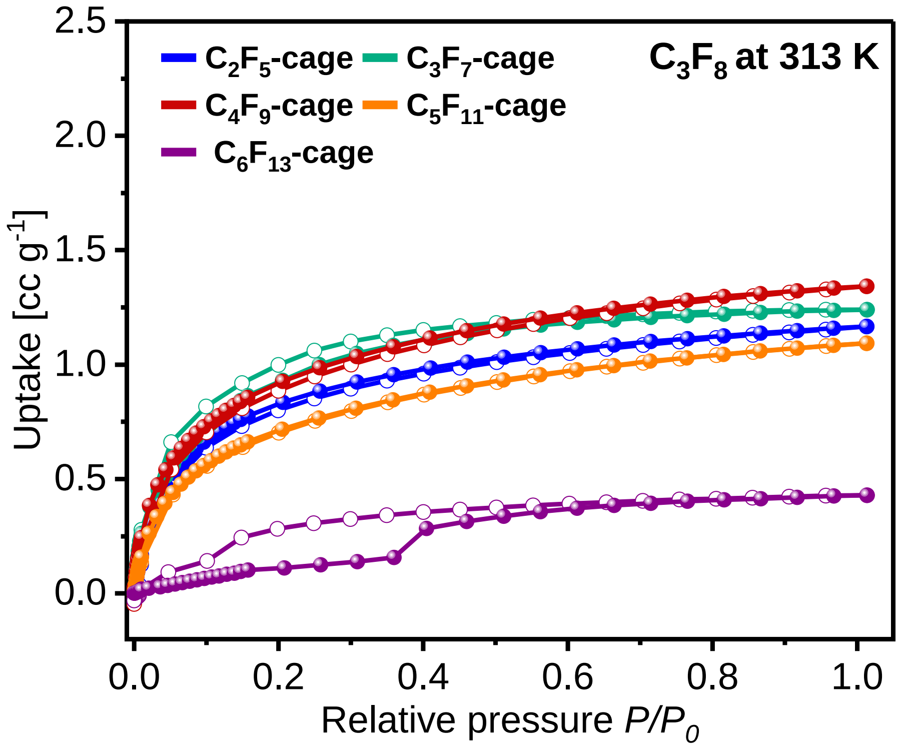

Figure S255. C_3F_8 sorption isotherms at 313 K. blue: **C_2F_5-cage**; green: **C_3F_7-cage**; red: **C_4F_8-cage**; orange: **C_5F_{11}-cage**; purple: **C_6F_{13}-cage**. Full circles: adsorption; empty circles: desorption. Note that the adsorption of **CF_3-cage** was too low to be measured.

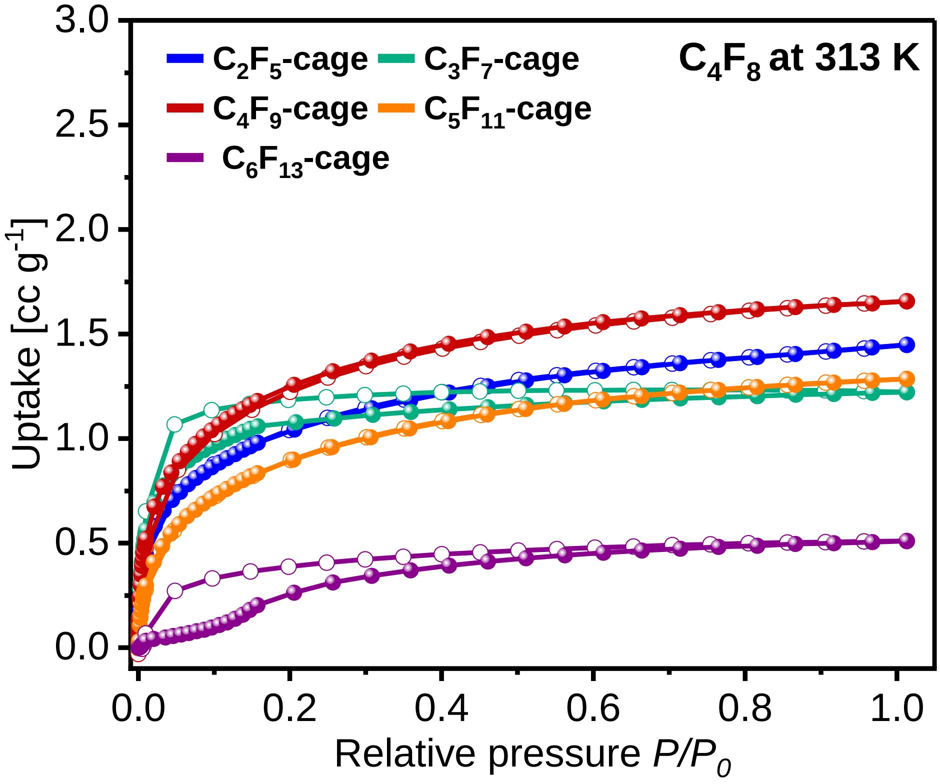

Figure S256. C_4F_8 sorption isotherms at 313 K. blue: **C_2F_5-cage**; green: **C_3F_7-cage**; red: **C_4F_8-cage**; orange: **C_5F_{11}-cage**; purple: **C_6F_{13}-cage**. Full circles: adsorption; empty circles: desorption. Note that the adsorption of **CF_3-cage** was too low to be measured.

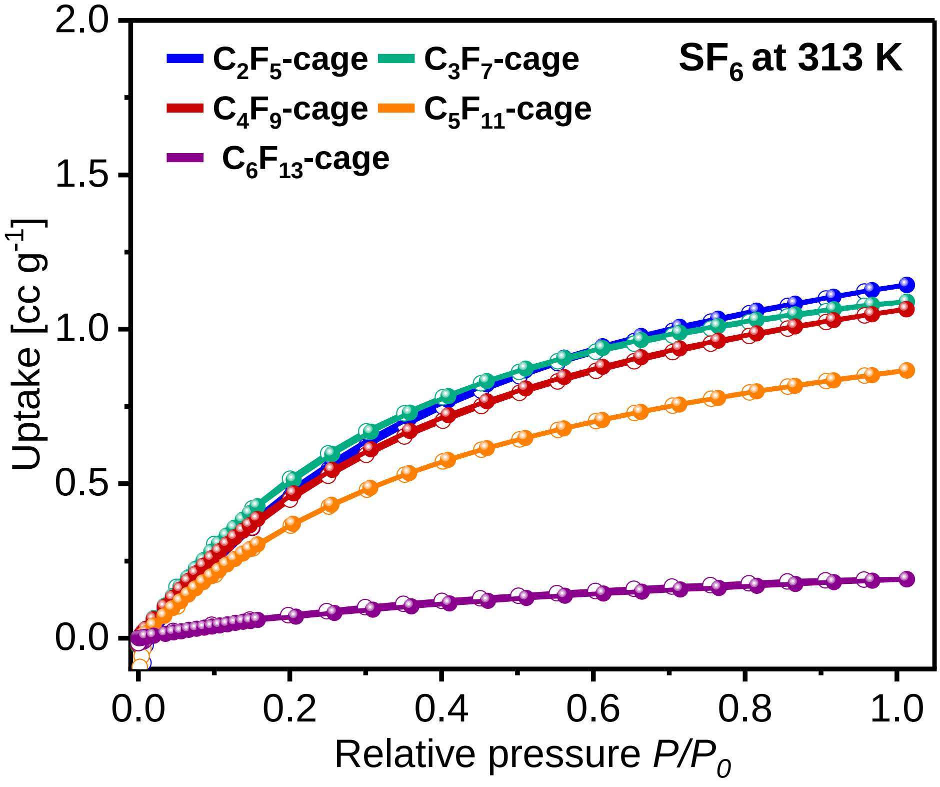

Figure S257. SF_6 sorption isotherms at 313 K. blue: **C_2F_5-cage**; green: **C_3F_7-cage**; red: **C_4F_8-cage**; orange: **C_5F_{11}-cage**; purple: **C_6F_{13}-cage**. Full circles: adsorption; empty circles: desorption. Note that the adsorption of **CF_3-cage** was too low to be measured.

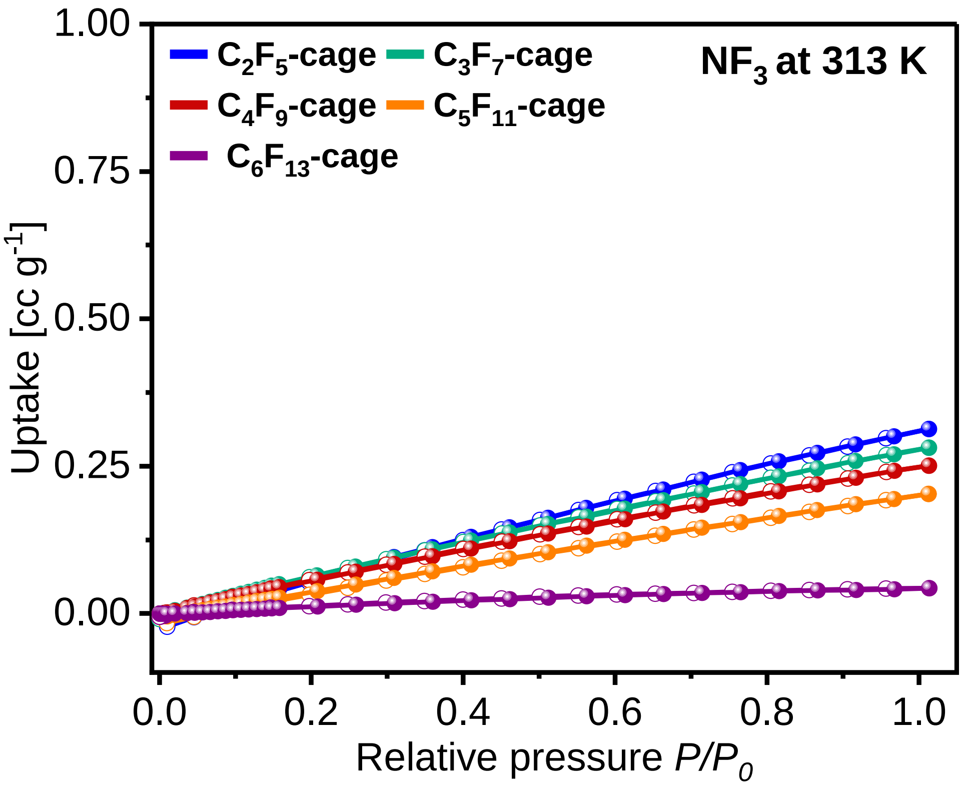

Figure S258. NF_3 sorption isotherms at 313 K. blue: **C_2F_5-cage**; green: **C_3F_7-cage**; red: **C_4F_8-cage**; orange: **C_5F_{11}-cage**; purple: **C_6F_{13}-cage**. Full circles: adsorption; empty circles: desorption. Note that the adsorption of **CF_3-cage** was too low to be measured.

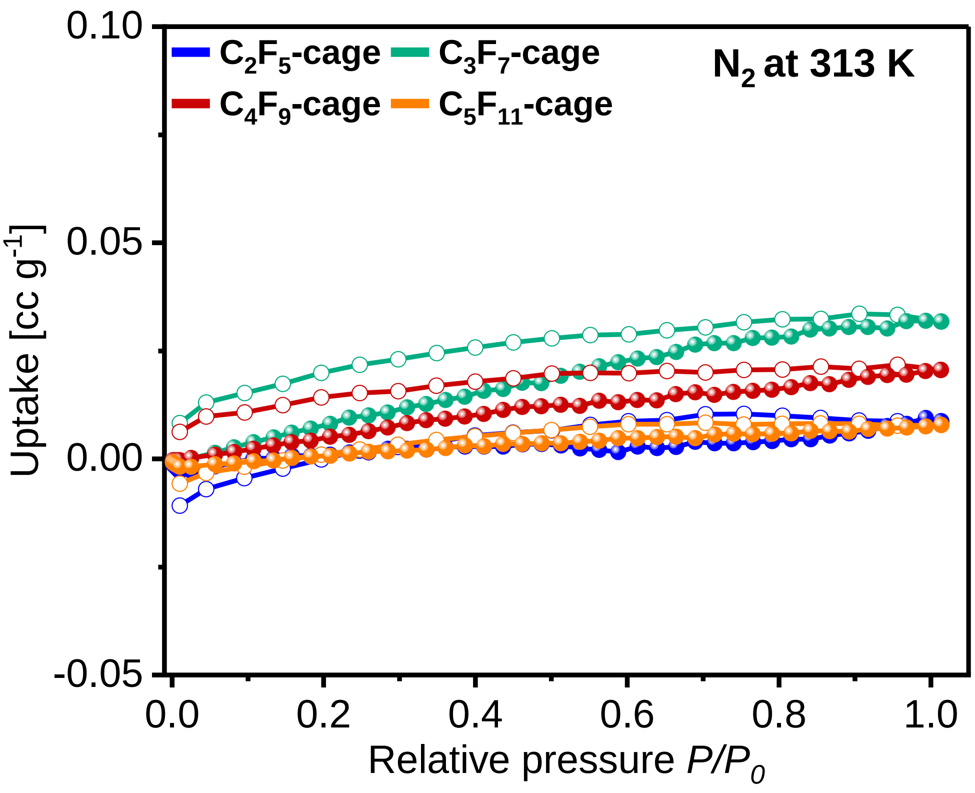

Figure S259. N_2 sorption isotherms at 313 K. Black: **CF_3-cage**; blue: **C_2F_5-cage**; green: **C_3F_7-cage**; red: **C_4F_8-cage**; orange: **C_5F_{11}-cage**. Full circles: adsorption; empty circles: desorption. Note that the adsorption of **CF_3-cage** and **C_6F_{13}-cage** was too low to be measured.

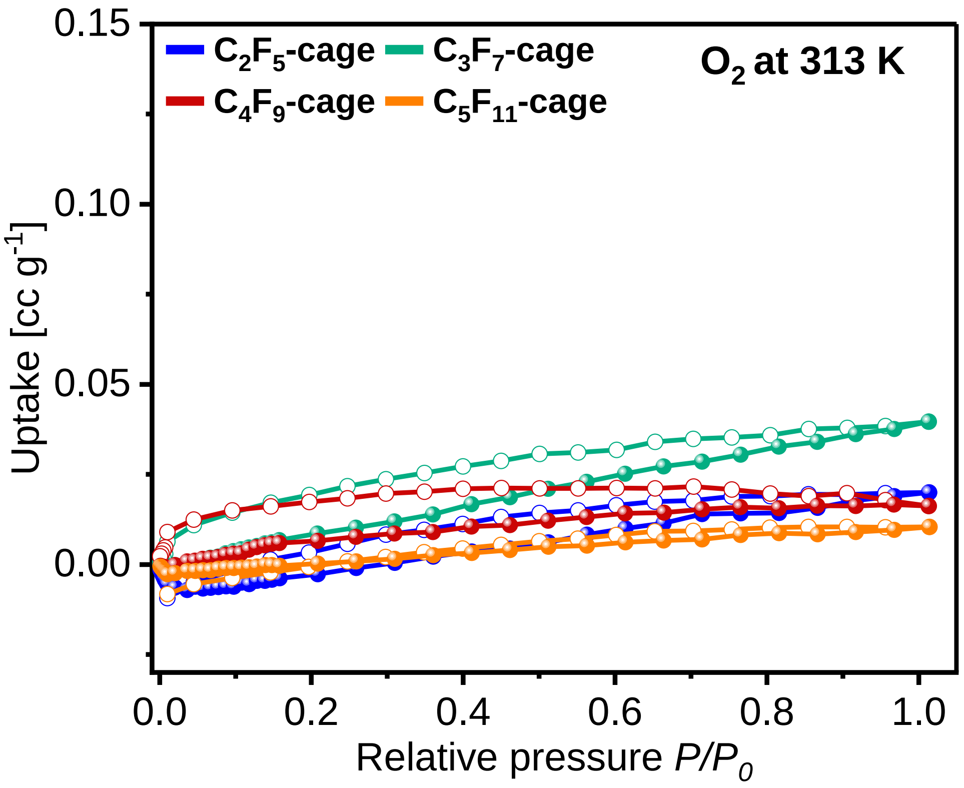

Figure S260. O_2 sorption isotherms at 313 K. blue: **C_2F_5-cage**; green: **C_3F_7-cage**; red: **C_4F_8-cage**; orange: **C_5F_{11}-cage**. Full circles: adsorption; empty circles: desorption. Note that the adsorption of **CF_3-cage** and **C_6F_{13}-cage** was too low to be measured.

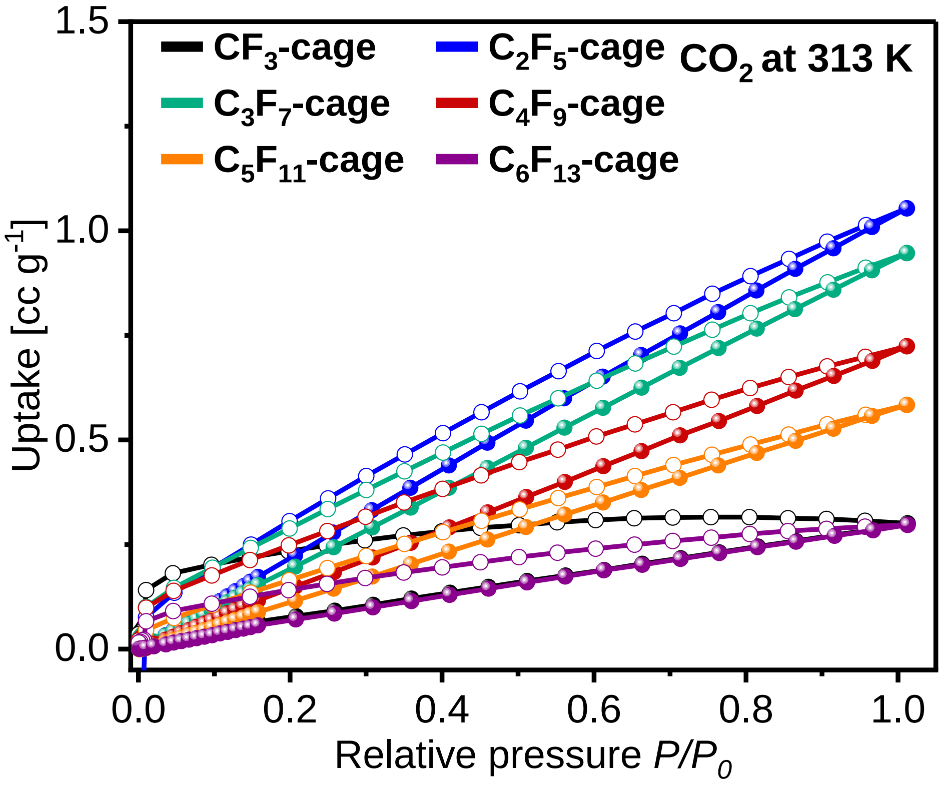

Figure S261. CO_2 sorption isotherms at 313 K. Black: **CF_3-cage**; blue: **C_2F_5-cage**; green: **C_3F_7-cage**; red: **C_4F_8-cage**; orange: **C_5F_{11}-cage**; purple: **C_6F_{13}-cage**. Full circles: adsorption; empty circles: desorption.

Table S14. Fitting and IAST parameters of Tóth and LAI isotherms as well as R^2 -values and Henry constants at 313 K.

**Cages**	**Gas**	**Affinity const. *K* [1/bar]**	**Max. uptake *q*max [mmol/g]**	**Heterogenity Parameter**	**R^2**	***Model***	**K_H**
**C_2F_5-cage**	CF_4	0.400693	0.745486	1.6339	0.999527	Tóth	0.298711022
	C_2F_6	2.447332	1.238452	1.1074	0.999954	Tóth	3.03090321
	C_3F_8	18.755356	1.377738	0.6850	0.999370	Tóth	25.83996666
	c-C_4F_8	281.046362	2.502930	0.3004	0.997555	Tóth	703.4393708
	SF_6	1.941321	1.479070	1.3112	0.999811	Tóth	2.871349651
	NF_3	0.000313	1000	1	0.995711	LAI	0.313
	N_2	0.000006	1000	1	0.595314	LAI	0.006
	O_2	0.000017	1000	1	0.461232	LAI	0.017
	CO_2	0.347510	3.095555	2.3563	0.999905	Tóth	1.075736318
**C_3F_7-cage**	CF_4	0.328627	0.967731	1.2989	0.999959	Tóth	0.318022535
	C_2F_6	2.669151	1.273271	1.1090	0.999988	Tóth	3.398552563
	C_3F_8	32.407683	1.707008	0.5177	0.998952	Tóth	55.32017414
	c-C_4F_8	219.230297	1.319643	0.555	0.998940	Tóth	289.3057268
	SF_6	2.407464	1.329566	1.336	0.999965	Tóth	3.200882281
	NF_3	0.181015	1.721956	1.1523	0.999851	Tóth	0.311699865
	N_2	0.000035	1000	1	0.984478	LAI	0.035
	O_2	0.000040	1000	1	0.997520	LAI	0.04
	CO_2	0.000941	1000	1	0.999966	LAI	0.941
**C_4F_9-cage**	SF_6	2.062049	1.506985	1.0701	0.999987	Tóth	3.107476912
	NF_3	0.198136	1.407462	1.1823	0.999899	Tóth	0.278868891
	O_2	0.000021	1000	1	0.927206	LAI	0.021
	CO_2	0.000713	1000	1	0.999932	LAI	0.713
**C_5F_{11}-cage**	CF_4	0.430785	0.530247	1.8875	0.999833	Tóth	0.228422454
	C_2F_6	2.363392	1.149219	0.8897	0.999905	Tóth	2.716054991
	C_3F_8	21.387619	1.609088	0.4953	0.999439	Tóth	34.41456108
	c-C_4F_8	80.115084	2.274630	0.3481	0.998370	Tóth	182.2321735
	SF_6	1.940461	1.206979	1.1286	0.999797	Tóth	2.342095677
	NF_3	0.0002	1000	1	0.995571	LAI	0.2
	N_2	0.000007	1000	1	0.960242	LAI	0.007
	O_2	0.000010	1000	1	0.851279	LAI	0.01
	CO_2	0.000573	1000	1	0.999597	LAI	0.573
**C_6F_{13}-cage**	CF_4	0.000032	1000	1	0.940966	LAI	0.032
	C_2F_6	1.241528	0.219958	1.1358	0.999815	Tóth	0.273084016
	C_3F_8	0.000525	1000	1	0.960249	LAI	0.525
	c-C_4F_8	2.343624	0.509496	2.9125	0.995371	Tóth	1.194067054
	SF_6	1.048473	0.442444	0.7834	0.999615	Tóth	0.463890588
	NF_3	1.108199	0.048717	3.31	0.997440	Tóth	0.053988131
	CO_2	0.008602	43.893866	0.4428	0.999848	Tóth	0.377575035

Note that the adsorption of **CF_4-cage** and in for selected gases of **C_6F_{13}-cage** was to low to be measured.

Gas sorption fitting curves at 313 K

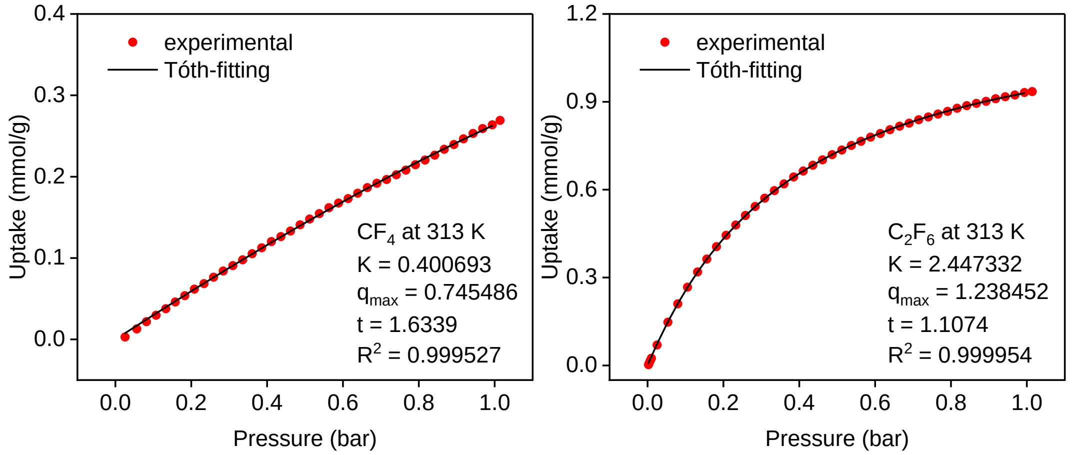

Figure S262. CF_4 (left) and C_2F_6 (right) isotherms and fitting curves of **C_2F_5-cage** at 313 K.

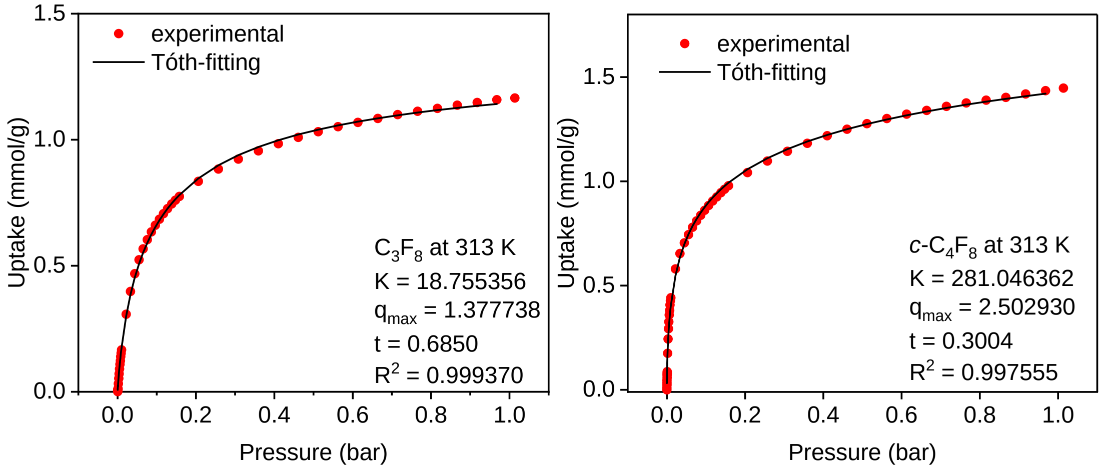

Figure S263. C_3F_8 (left) and *c*-C_4F_8 (right) isotherms and fitting curves of **C_2F_5-cage** at 313 K.

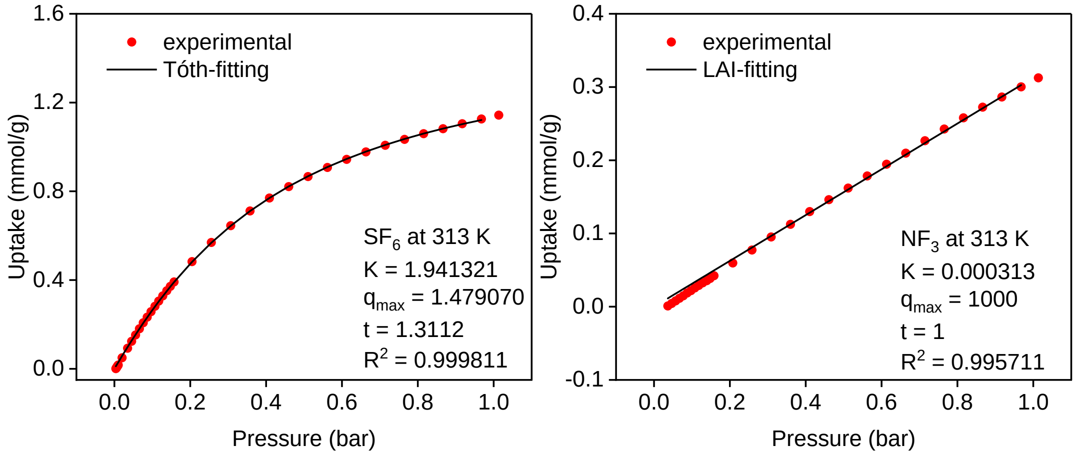

Figure S264. SF_6 (left) and NF_3 (right) isotherms and fitting curves of **C_2F_5-cage** at 313 K.

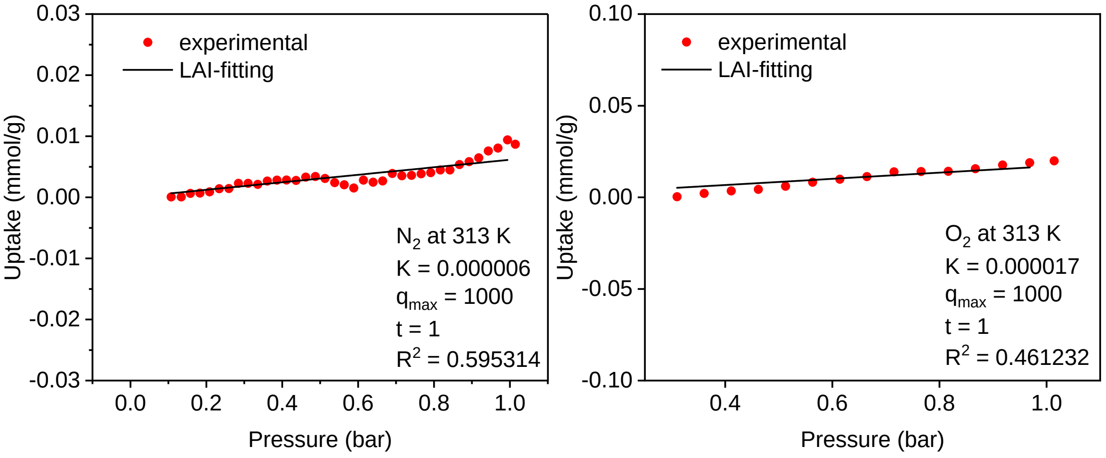

Figure S265. N_2 (left) and O_2 (right) isotherms and fitting curves of **C_2F_5-cage** at 313 K.

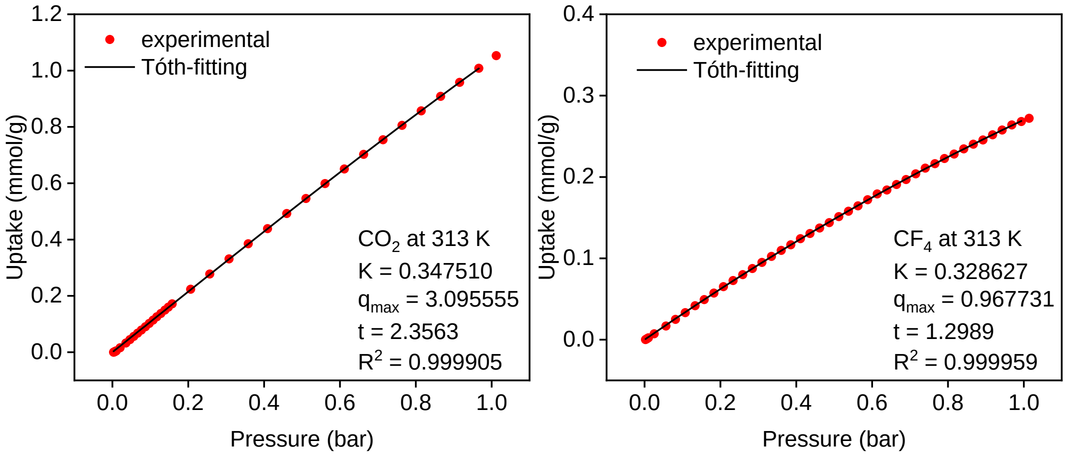

Figure S266. CO_2 isotherm and fitting curve of **C_2F_5-cage** (left) and CF_4 isotherm and fitting curve of **C_3F_7-cage** (right) at 313 K.

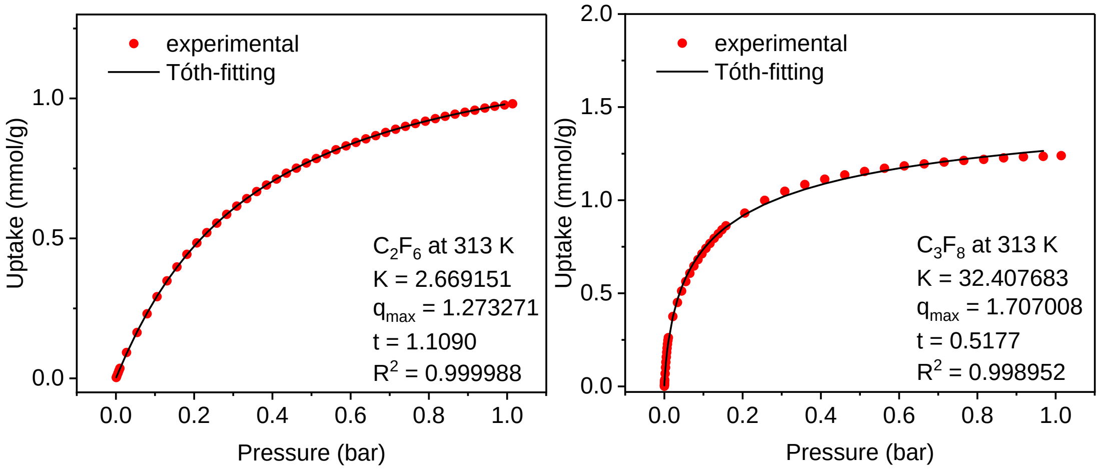

Figure S267. C_2F_6 (left) and C_3F_8 (right) isotherms and fitting curves of **C_3F_7-cage** at 313 K.

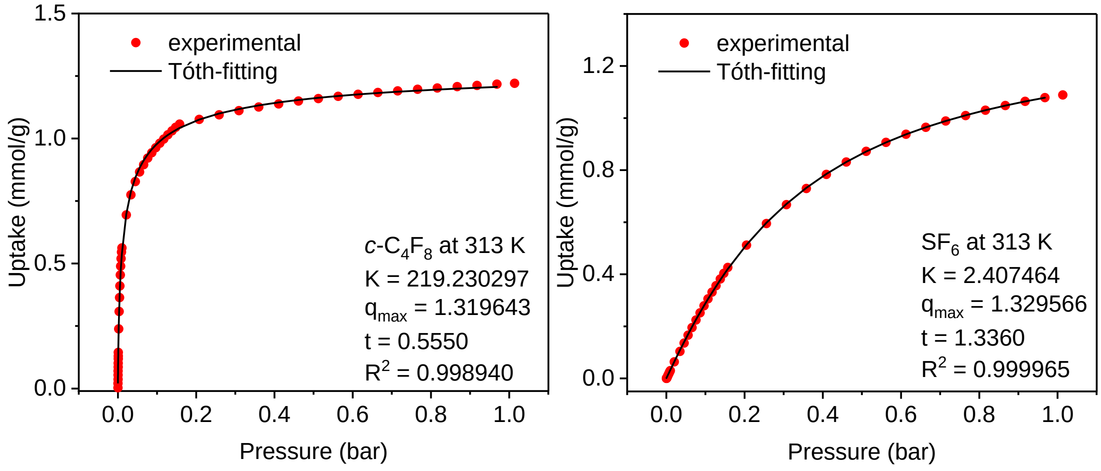

Figure S268. *c*-C_4F_8 (left) and SF_6 (right) isotherms and fitting curves of **C_3F_7-cage** at 313 K.

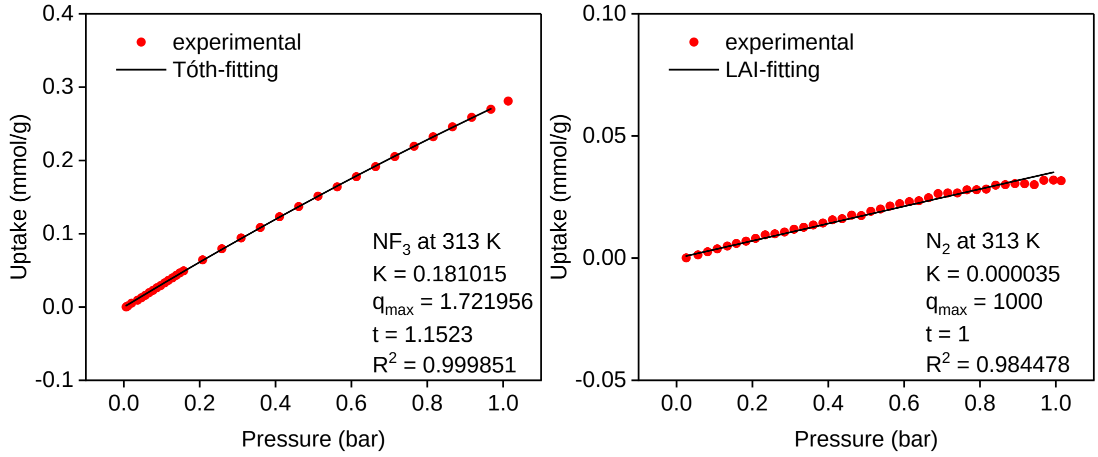

Figure S269. NF_3 (left) and N_2 (right) isotherms and fitting curves of **C_3F_7-cage** at 313 K.

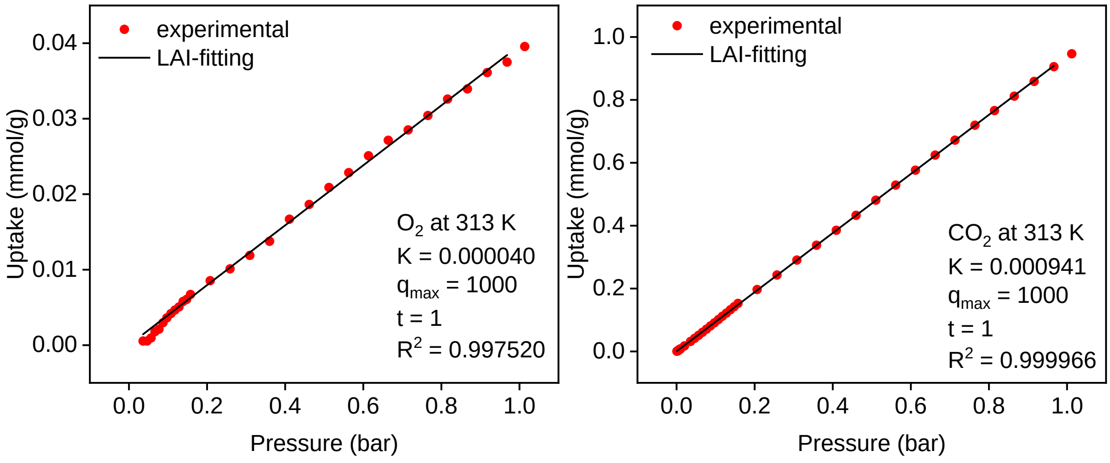

Figure S270. O_2 (left) and CO_2 (right) isotherms and fitting curves of **C_3F_7-cage** at 313 K.

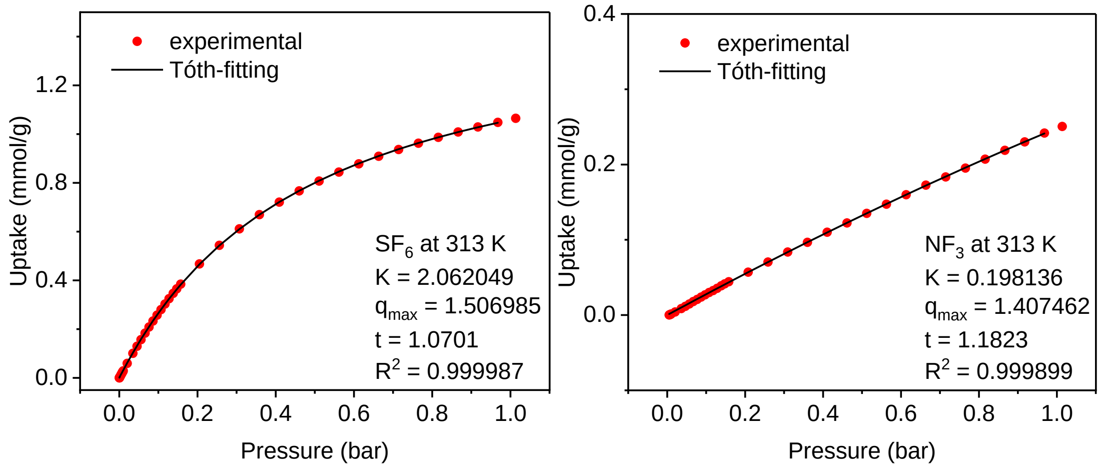

Figure S271. SF_6 (left) and NF_3 (right) isotherms and fitting curves of **C_4F_9-cage** at 313 K.

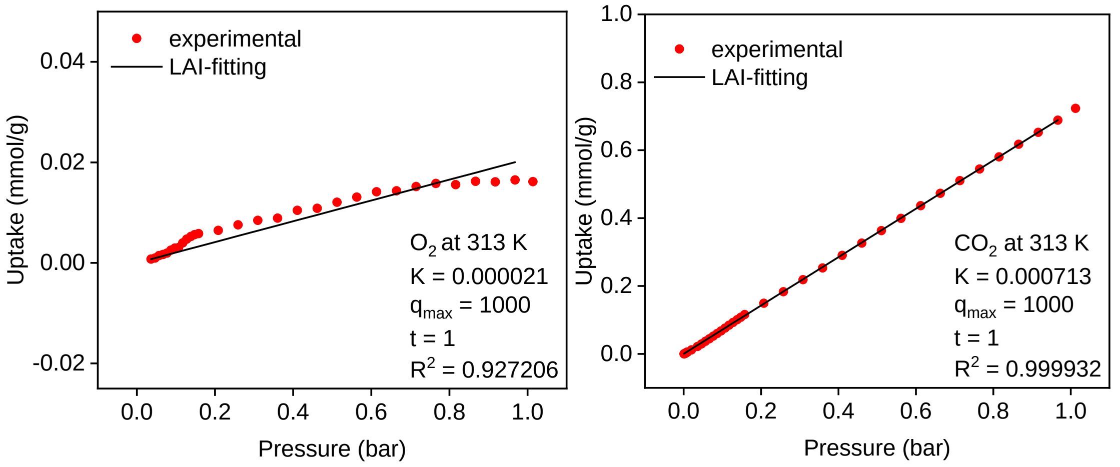

Figure S272. O_2 (left) and CO_2 (right) isotherms and fitting curves of **C_4F_9-cage** at 313 K.

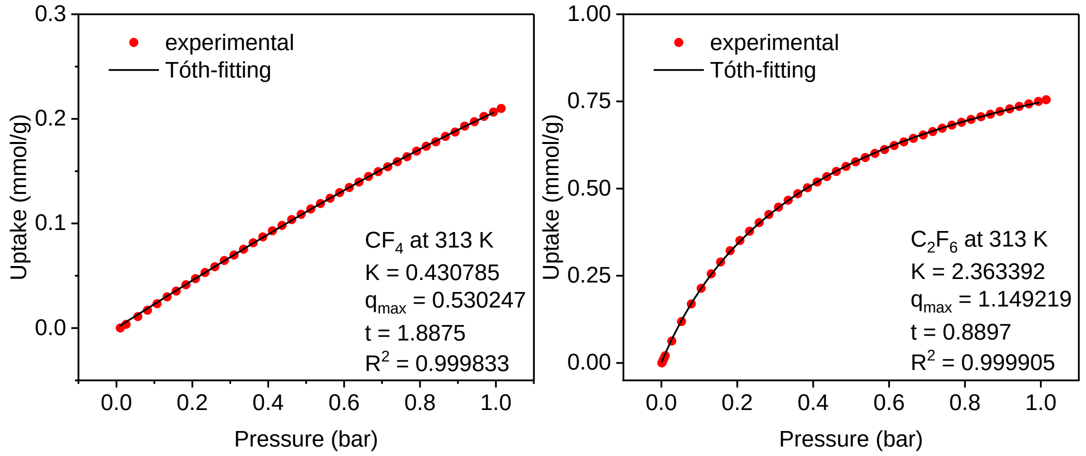

Figure S273. CF_4 (left) and C_2F_6 (right) isotherms and fitting curves of **C_5F_{11}-cage** at 313 K.

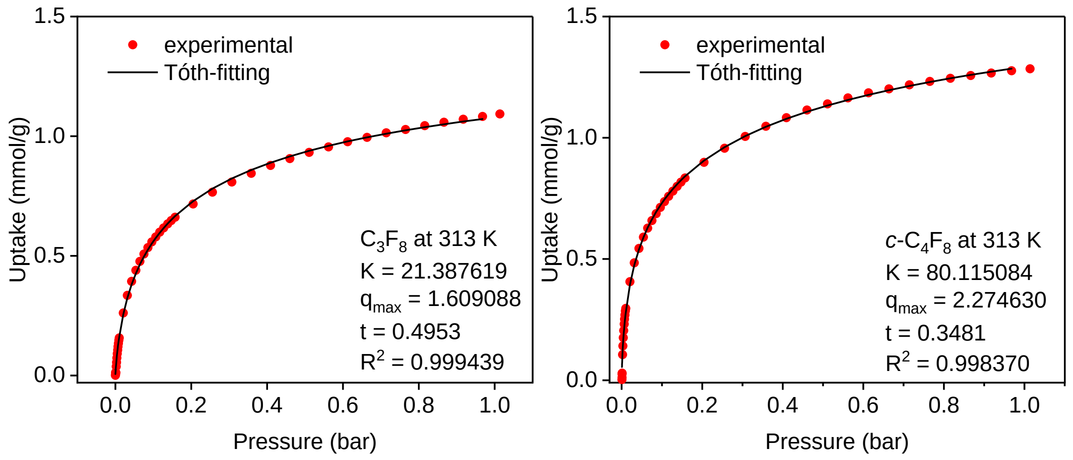

Figure S274. C_3F_8 (left) and c-C_4F_8 (right) isotherms and fitting curves of **C_5F_{11}-cage** at 313 K.

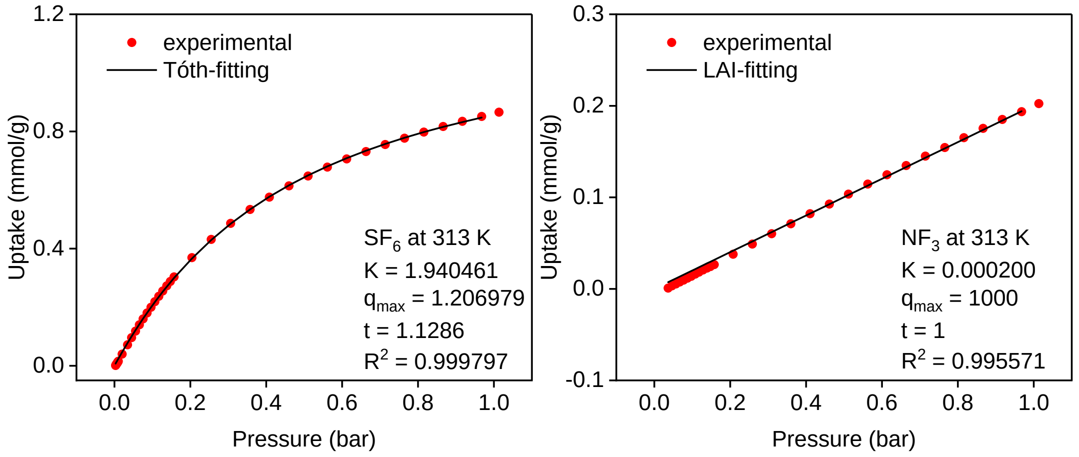

Figure S275. SF_6 (left) and NF_3 (right) isotherms and fitting curves of **C_5F_{11}-cage** at 313 K.

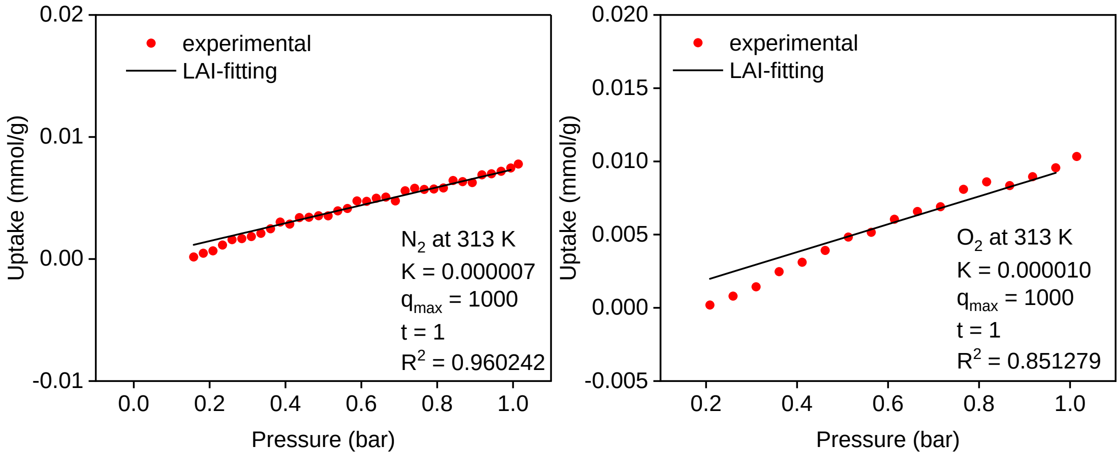

Figure S276. N_2 (left) and O_2 (right) isotherms and fitting curves of **C_5F_{11}-cage** at 313 K.

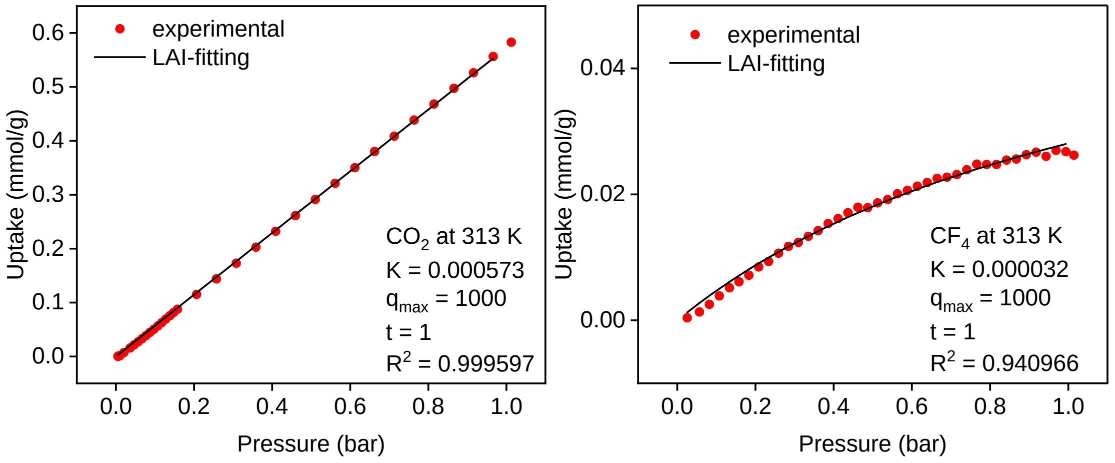

Figure S277. CO_2 isotherm and fitting curve of **C_5F_{11}-cage** (left) and CF_4 isotherm and fitting curve of **C_6F_{13}-cage** (right) at 313 K.

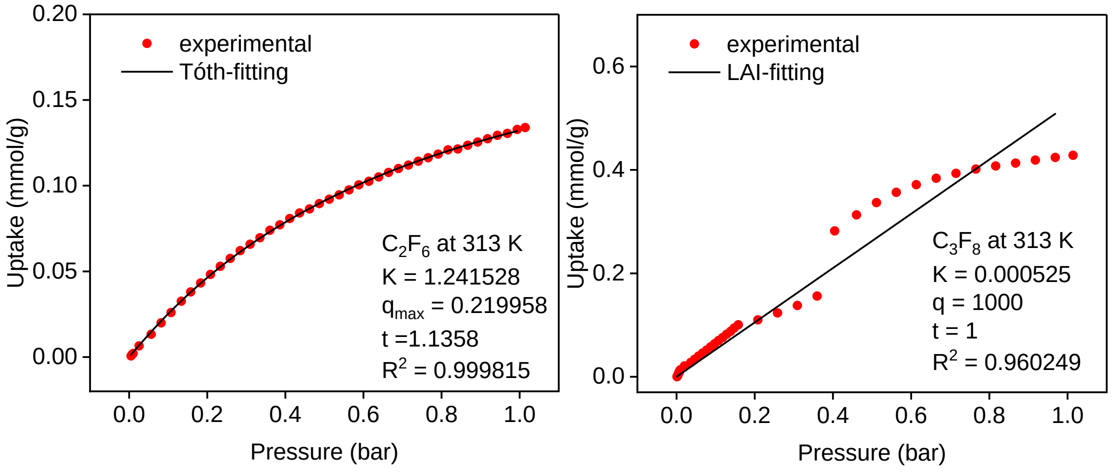

Figure S278. C_2F_6 (left) and C_3F_8 (right) isotherms and fitting curves of **C_6F_{13}-cage** at 313 K.

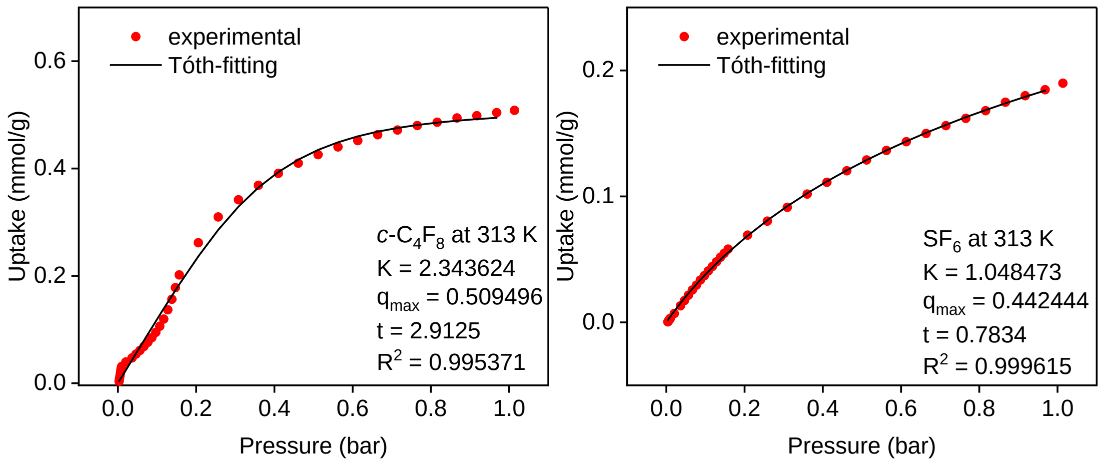

Figure S279. *c*-C_4F_8 (left) and SF_6 (right) isotherms and fitting curves of **C_6F_{13}-cage** at 313 K.

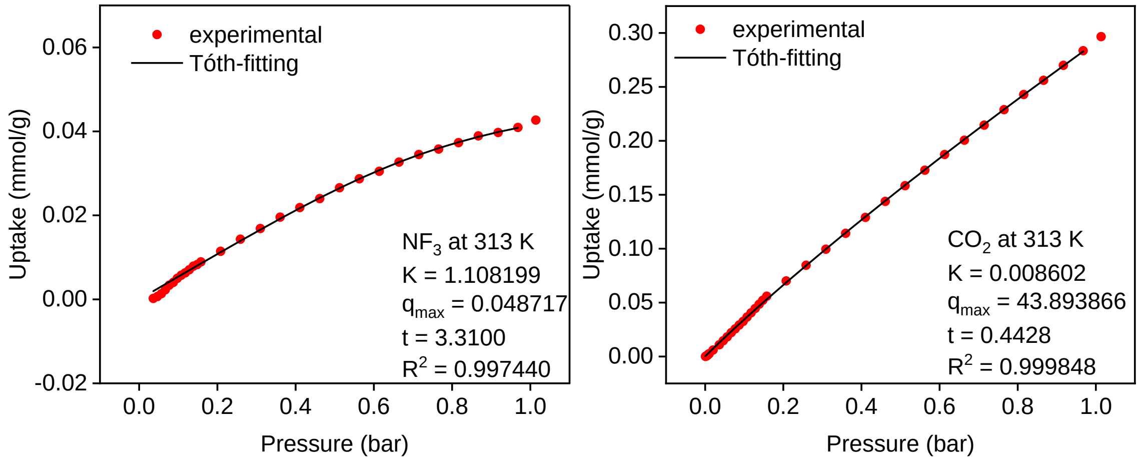

Figure S280. NF_3 (left) and CO_2 (right) isotherms and fitting curves of **C_6F_{13}-cage** at 313 K.

Gas Uptake Summary

Table S15. Summary of gas uptake (mmol g^{-1}) at variable temperature and 1 bar.

	cages	CF_4	C_2F_6	C_3F_8	*c*-C_4F_8	SF_6	NF_3	N_2	O_2	CO_2
273 K	CF_3-cage	0.31	0.27	0.29	2.4	0.26	0.37	0.28	0.44	1.08
	C_2F_5-cage	1.11	1.91	1.95	3.53	2.21	1.22	0.34	0.36	3.23
	C_3F_7-cage	0.99	1.56	1.49	2.9	1.67	1.02	0.35	0.37	2.88
	C_4F_9-cage	0.93	1.78	2.09	4.41	2	0.95	0.35	0.4	2.35
	C_5F_{11}-cage	0.74	1.4	1.65	2.99	1.61	0.75	0.21	0.21	1.89
	C_6F_{13}-cage	0.34	0.59	0.73	2.05	0.86	0.38	0.2	0.21	0.92
283 K	CF_3-cage	0.21	0.18	0.21	1.41	0.18	0.26	0.2	0.32	0.86
	C_2F_5-cage	0.84	1.67	1.79	2.81	1.97	0.92	0.25	0.27	2.52
	C_3F_7-cage	0.75	1.46	1.47	2.2	1.53	0.78	0.26	0.28	2.26
	C_4F_9-cage	0.73	1.57	1.92	3.92	1.77	0.75	0.26	0.3	1.85
	C_5F_{11}-cage	0.56	1.24	1.53	2.09	1.4	0.57	0.15	0.16	1.44
	C_6F_{13}-cage	0.24	0.46	0.7	1.25	0.76	0.27	0.14	0.16	0.72
298 K	CF_3-cage	0.1	0.08	0.09	0.13	0.09	0.15	0.11	0.12	0.55
	C_2F_5-cage	0.51	1.27	1.45	1.71	1.53	0.55	0.12	0.14	1.65
	C_3F_7-cage	0.49	1.24	1.37	1.43	1.35	0.51	0.14	0.16	1.49
	C_4F_9-cage	0.46	1.24	1.64	2.02	1.41	0.45	0.13	0.15	1.17
	C_5F_{11}-cage	0.36	0.98	1.3	1.5	1.11	0.35	0.08	0.08	0.92
	C_6F_{13}-cage	0.13	0.26	0.54	0.6	0.34	0.15	0.06	0.07	0.48
313 K	CF_3-cage	-	-	-	-	-	-	-	-	0.3
	C_2F_5-cage	0.27	0.94	1.17	1.45	1.14	0.31	0.01	0.02	1.05
	C_3F_7-cage	0.27	0.98	1.24	1.22	1.09	0.28	0.03	0.04	0.95
	C_4F_9-cage	0.25	0.94	1.34	1.66	1.06	0.25	0.02	0.02	0.72
	C_5F_{11}-cage	0.21	0.75	1.09	1.28	0.87	0.2	0.01	0.01	0.58
	C_6F_{13}-cage	0.03	0.13	0.43	0.51	0.19	0.04	-	-	0.3

-: no uptake; the red data represents the highest uptake for each gas at each respective temperature.

Gas Selectivity Summary

Table S16. Selected IAST and Henry selectivity (F-gas over nitrogen) at variable temperature and 1 bar.[a]

	cages	CF_4/N_2	C_2F_6/N_2	C_3F_8/N_2	*c*-C_4F_8/N_2	SF_6/N_2	NF_3/N_2
273 K	**CF_3-cage**	1.10/1.10/1.10/1.10	1.10/1.10/1.11/2.11	1.06/1.06/1.06/1.05	1.52/1.51/1.51/1.50	1.10/1.10/1.10/2.76	1.52/1.54/1.55/1.93
	**C_2F_5-cage**	4.00/4.18/4.23/4.39	18.8/34.8/51.4/74.0	32.8/91.7/292/2261	45.2/151/680/30217	21.2/36.5/47.3/54.7	4.06/4.13/4.14/4.16
	**C_3F_7-cage**	3.37/3.40/3.40/3.40	16.7/27.8/41.7/84.8	43.9/111/334/2083	77.5/247/937/3754	19.7/31.6/42.2/54.4	3.36/3.38/3.39/3.41
	**C_4F_9-cage**	3.10/3.21/3.24/3.37	16.3/30.8/48.1/85.6	34.4/100/350/4977	47.5/163/801/150374	17.7/31.5/44.5/61.4	2.95/3.00/3.02/3.06
	**C_5F_{11}-cage**	4.19/4.36/4.40/4.51	21.4/40.0/61.6/96.8	42.3/117/379/3863	57.3/185/804/38486	22.9/39.9/55.1/70.8	3.79/3.81/3.81/3.82
	**C_6F_{13}-cage**	1.98/2.04/2.05/2.26	3.07/3.07/3.07/3.07	1.32/1.32/1.32/1.32	2.96/2.96/2.96/2.96	4.13/4.13/4.13/4.13	2.21/2.28/2.30/2.45
283 K	**CF_3-cage**	1.12/1.13/1.13/1.16	1.12/1.13/1.14/1.51	1.25/1.27/1.28/1.28	1.44/1.44/1.44/1.44	1.18/1.20/1.20/2.28	1.51/1.54/1.54/1.74
	**C_2F_5-cage**	3.99/4.11/4.14/4.23	20.1/33.8/44.8/55.9	37.7/99.5/280/1295	52.8/166/687/30131	22.6/35.4/42.4/46.4	4.04/4.07/4.07/4.10
	**C_3F_7-cage**	3.41/3.49/3.50/3.53	16.5/27.5/36.5/48.9	36.1/104/332/1608	46.1/163/714/2393	18.6/30.6/37.7/42.2	3.37/3.43/3.45/3.49
	**C_4F_9-cage**	3.18/3.27 /3.29/3.36	17.5/30.0/41.5/58.9	39.5/108/338/2767	57.3/187/836/59278	18.8/30.5/39.0/47.8	3.08/3.10/3.10/3.11
	**C_5F_{11}-cage**	4.24/4.34/4.36/4.41	23.8/40.9/56.9/75.8	50.3/132/383/2308	70.0/216/864/26324	25.3/40.5/50.7/58.0	3.92/3.93/3.93/3.93
	**C_6F_{13}-cage**	1.98/1.99/1.99/2.05	4.98/5.46/5.72/11.1	5.01/5.20/5.26/5.49	7.01/6.34/6.23/6.04	5.52/5.12/5.05/4.90	2.24/2.24/2.25/2.26
298 K	**CF_3-cage**	0.94/0.94/0.94/0.90	0.81/0.81/0.81/1.07	0.78/0.79/0.79/0.71	0.98/0.98/0.98/0.89	0.89/0.88/0.88/1.19	1.44/1.42/1.42/1.34
	**C_2F_5-cage**	4.73/4.84/4.86/4.91	26.9/40.1/47.04/51.0	55.6/133/299/606	80.8/238/896/29832	28.9/38.7/41.8/42.8	4.67/4.67/4.67/4.67
	**C_3F_7-cage**	3.91/4.02/4.04/4.10	22.6/32.6/36.5/38.1	51.5/135/360/1040	78.3/263/1064/4580	24.2/34.2/37.6/38.8	3.75/3.78/3.78/3.79
	**C_4F_9-cage**	3.93/4.04/4.07/4.15	24.1/37.2/46.0/53.8	59.9/152/404/1537	90.9/277/1079/24459	25.7/36.9/42.3/45.4	3.70/3.73/3.74/3.75
	**C_5F_{11}-cage**	5.18/5.29/5.31/5.36	31.3/47.8/58.3/65.4	72.5/174/422/1245	106/301/1048/14252	33.0/46.2/51.8/54.1	4.54/4.54/4.54/4.54
	**C_6F_{13}-cage**	2.26/2.23/2.23/2.21	7.10/6.73/6.71/6.88	12.7/9.12/8.70/8.34	16.8/10.9/10.3/9.87	11.5/10.9/11.5/17.9	2.62/2.56/2.55/2.49
313 K	**CF_3-cage**	-	-	-	-	-	-
	**C_2F_5-cage**	49.0/49.7/49.8/49.8	348/464/501/505	829/1836/3364/4307	1282/3622/12345/117240	377/461/477/479	52.2/52.2/52.2/52.2
	**C_3F_7-cage**	8.77/9/9.04/9.09	64.9/87.9/95.5/97.1	165/390/866/1581	250/767/2627/8266	68.4/86.9/90.9/91.5	8.66/8.83/8.86/8.91
	**C_4F_9-cage**	13.6/14.0/14.0/14.1	101/143/164/171	278/658/1522/3243	443/1268/4385/41475	105/136/146/148	12.9/13.2/13.2/13.3
	**C_5F_{11}-cage**	32.3/32.6/32.6/32.6	239/334/378/388	622/1392/2888/4916	864/2216/6249/26033	248/314/332/335	28.6/28.6/28.57/28.6
	**C_6F_{13}-cage**	-	-	-	-	-	-

[a] $S_{IAST, 50:50}/S_{IAST, 10:90}/S_{IAST, 1:99}/S_{Henry}$; the red and green data represents the highest selectivity of $S_{IAST, 50:50}/S_{IAST, 10:90}/S_{IAST, 1:99}$ and S_{Henry}, respectively, for each gas at each respective temperature. - : The selectivity of F-gas over nitrogen for **CF_3-cage** and **C_6F_{13}-cage** cannot be calculated because there is no valid nitrogen uptake data available at 313 K.

Table S17. Selected IAST and Henry selectivity (F-gas over oxygen) at variable temperature and 1 bar.[a]

	cages	CF_4/O_2	C_2F_6/O_2	C_3F_8/O_2	c-C_4F_8/O_2	SF_6/O_2	NF_3/O_2
273 K	CF_3-cage	0.69/0.69/0.69/0.67	0.66/0.64/0.64/1.28	0.66/0.66/0.66/0.64	0.95/0.95/0.95/0.91	0.65/0.63/0.62/1.68	0.92/0.92/0.91/1.17
	C_2F_5-cage	3.76/3.93/3.97/4.13	17.7/32.6/47.9/69.6	30.8/86.0/272/2127	42.4/141/636/28425	19.9/34.2/44.3/51.4	3.82/3.88/3.89/3.91
	C_3F_7-cage	3.09/3.16/3.17/3.21	13.2/24.4/38.0/80.1	26.6/80.9/287/1966	36.8/141/708/3542	15.1/27.6/38.7/51.4	3.07/3.14/3.16/3.22
	C_4F_9-cage	2.75/2.81/2.83/2.93	16.4/27.9/41.4/74.5	43.6/106/320/4331	73.8/204/809/130872	18.0/28.7/38.6/53.5	2.62/2.64/2.64/2.66
	C_5F_{11}-cage	4.21/4.38/4.42/4.53	21.5/40.2/61.9/97.2	42.5/118/381/3882	57.6/186/808/38671	23.0/40.1/55.4/71.1	3.81/3.83/3.83/3.83
	C_6F_{13}-cage	1.80/1.84/1.85/1.98	2.82/2.79/2.78/2.68	3.74/3.68/3.66/3.54	2.73/2.69/2.69/2.59	3.83/3.76/3.75/3.62	2.02/2.06/2.07/2.15
283 K	CF_3-cage	0.66/0.66/0.65/0.61	0.64/0.62/0.62/0.80	0.71/0.69/0.68/0.82	0.87/0.88/0.88/0.76	0.67/0.65/0.64/1.21	0.89/0.88/0.88/0.92
	C_2F_5-cage	3.74/3.85/3.88/3.97	18.8/31.6/41.7/52.4	35.4/93.0/261/1215	49.5/155/641/28270	21.2/33.1/39.6/43.5	3.79/3.82/3.82/3.83
	C_3F_7-cage	3.06/3.07/3.07/3.02	16.2/25.0/31.8/41.8	39.6/105/304/1376	53.2/176/692/2048	18.5/28.1/33.1/36.1	3.02/3.02/3.02/2.99
	C_4F_9-cage	2.75/2.76/2.77/2.79	18.5/26.9/34.6/48.9	62.9/125/306/2297	134/292/889/49205	20.3/27.4/32.7/39.7	2.65/2.63/2.62/2.58
	C_5F_{11}-cage	4.10/4.20/4.22/4.26	23.0/39.5/54.8/73.3	48.7/128/369/2234	67.7/209/834/25475	24.5/39.2/49.0/56.2	3.80/3.80/3.80/3.80
	C_6F_{13}-cage	1.71/1.72/1.73/1.79	4.14/4.65/4.87/9.64	5.81/5.6/5.55/5.37	5.72/5.49/5.44/5.27	4.59/4.44/4.41/4.27	1.93/1.95/1.95/1.97
298 K	CF_3-cage	0.82/0.81/0.81/0.78	0.71/0.70/0.70/0.93	0.69/0.70/0.70/0.62	0.87/0.87/0.87/0.77	0.77/0.76/0.76/1.03	1.25/1.25/1.25/1.16
	C_2F_5-cage	4.23/4.33/4.35/4.40	24.0/35.7/41.9/45.7	49.7/119/265/543	72.3/212/797/26716	25.9/34.6/37.3/38.3	0.42/0.42/0.42/0.42
	C_3F_7-cage	3.56/3.65/3.67/3.74	20.6/29.6/33.1/34.7	46.9/122/325/948	71.2/239/965/4172	22.0/31.0/34.1/35.3	3.41/3.44/3.44/3.45
	C_4F_9-cage	3.38/3.47/3.49/3.58	20.7/31.8/39.1/46.3	51.5/130/343/1323	78.2/238/922/21057	22.1/31.6/36.1/39.1	3.18/3.21/3.22/3.23
	C_5F_{11}-cage	5.05/5.16/5.18/5.22	30.5/46.6/56.8/63.8	70.7/170/411/1214	103/294/1021/13896	32.1/45.0/50.4/52.8	4.43/4.43/4.43/4.43
	C_6F_{13}-cage	1.96/1.95/1.95/1.81	5.70/5.85/5.86/5.66	9.17/7.96/7.66/6.86	11.2/9.49/9.07/8.11	8.42/9.36/9.90/14.7	2.28/2.24/2.23/2.05
313 K	CF_3-cage	-	-	-	-	-	-
	C_2F_5-cage	17.3/17.5/17.6/17.6	123/163/176/178	292/646/1178/1520	452/1275/4327/41379	133/162/168/169	18.4/18.4/18.4/18.4
	C_3F_7-cage	7.67/7.87/7.90/7.95	56.7/76.8/83.5/85.0	144/341/754/1383	219/671/2294/7233	59.8/76.0/79.5/80.0	7.58/7.72/7.75/7.79
	C_4F_9-cage	13.6/14.0/14.0/14.1	101/143/164/171	278/658/1522/3243	443/1268/4385/41475	105/136/146/148	12.9/13.2/13.2/13.3
	C_5F_{11}-cage	22.6/22.8/22.8/22.8	167/233/264/272	435/973/2015/3441	605/1550/4363/18223	174/220/233/234	20.0/20.0/20.0/20.0
	C_6F_{13}-cage	-	-	-	-	-	-

[a] $S_{IAST,\ 50:50}/S_{IAST,\ 10:90}/S_{IAST,\ 1:99}/S_{Henry}$; the red and green data represents the highest selectivity of $S_{IAST,\ 50:50}/S_{IAST,\ 10:90}/S_{IAST,\ 1:99}$ and S_{Henry}, respectively, for each gas at each respective temperature. - : The selectivity of F-gas over oxygen for **CF_3-cage** and **C_6F_{13}-cage** can't be calculated because there is no valid oxygen uptake data available at 313 K.

Table S18. Selected IAST and Henry selectivity (F-gas over carbon dioxide) at variable temperature and 1 bar.[a]

	cages	CF_4/CO_2	C_2F_6/CO_2	C_3F_8/CO_2	*c*-C_4F_8/CO_2	SF_6/CO_2	NF_3/CO_2
273 K	**CF_3-cage**	0.27/0.27/ 0.27/0.27	0.23/0.21/ 0.20/0.51	0.26/0.26/ 0.26/0.26	0.37/0.37/ 0.37/0.37	0.22/0.19/ 0.19/0.67	0.32/0.30/ 0.29/0.47
	**C_2F_5-cage**	0.27/0.25/ 0.24/0.35	1.25/1.35/ 1.38/5.84	2.68/4.28/ 5.92/179	4.63/9.75/ 21.1/2386	1.51/1.69/ 1.76/4.32	0.29/0.26/ 0.25/0.33
	**C_3F_7-cage**	0.26/0.22/ 0.21/0.32	1.05/1.07/ 1.07/8.07	2.75/4.85/ 7.60/198	4.93/13.2/ 36.4/357	1.26/1.36/ 1.40/5.18	0.28/0.26/ 0.26/0.32
	**C_4F_9-cage**	0.35/0.33/ 0.32/0.44	1.96/2.37/ 2.58/11.2	6.47/11.4/ 20.8/650	13.8/27.9/ 72.2/19631	2.20/2.70/ 2.90/8.02	0.36/0.34/ 0.34/0.40
	**C_5F_{11}-cage**	0.35/0.32/ 0.44/0.44	1.88/2.23/ 2.41/9.49	5.64/9.07/ 14.6/379	11.4/20.5/ 44.0/3773	2.10/2.48/ 2.66/6.94	0.34/0.30/ 0.29/0.37
	**C_6F_{13}-cage**	0.27/0.26/ 0.26/0.22	0.51/0.53/ 0.54/0.30	0.68/0.70/ 0.71/0.40	0.49/0.51/ 0.52/0.29	0.70/0.72/ 0.72/0.41	0.31/0.30/ 0.3/0.24
283 K	**CF_3-cage**	0.21/0.16/ 0.15/0.25	0.19/0.15/ 0.15/0.33	0.20/0.14/ 0.13/0.34	0.32/0.32/ 0.32/0.32	0.20/0.17/ 0.16/0.50	0.29/0.26/ 0.25/0.38
	**C_2F_5-cage**	0.29/0.26/ 0.26/0.35	1.42/1.58/ 1.64/4.64	3.15/5.26/ 7.56/108	5.29/11.2/ 24.5/2503	1.70/1.94/ 2.02/3.85	0.31/0.27/ 0.26/0.34
	**C_3F_7-cage**	0.29/0.24/ 0.24/0.35	1.36/1.48/ 1.53/4.83	3.92/7.05/ 11.7/159	6.07/14.5/ 36.6/237	1.58/1.80/ 1.89/4.17	0.30/0.29/ 0.28/0.35
	**C_4F_9-cage**	0.38/0.36/ 0.35/0.45	2.09/2.55/ 2.77/7.83	7.09/12.1/ 21.8/368	16.7/30.9/ 75.7/7874	2.32/2.81/ 3.04/6.35	0.38/0.36/ 0.36/0.41
	**C_5F_{11}-cage**	0.39/0.36/ 0.35/0.45	2.04/2.60/ 2.86/7.73	4.55/9.44/ 17.5/235	6.48/16.9/ 48.9/2684	2.23/2.82/ 3.08/5.92	0.37/0.33/ 0.31/0.40
	**C_6F_{13}-cage**	0.27/0.26/ 0.25/0.25	0.63/0.62/ 0.62/1.34	1.01/1.01/ 1.01/0.75	1.05/1.04/ 1.04/0.73	0.84/0.84/ 0.85/0.59	0.30/0.28/ 0.28/0.27
298 K	**CF_3-cage**	0.07/0.03/ 0.02/0.10	0.09/0.08/ 0.07/0.12	0.14/0.15/ 0.15/0.08	0.17/0.18/ 0.18/0.10	0.10/0.09/ 0.09/0.13	0.18/0.15/ 0.14/0.15
	**C_2F_5-cage**	0.28/0.26/ 0.25/0.33	1.57/1.76/ 1.83/3.47	3.65/6.15/ 8.65/41.2	6.05/12.9/ 28.6/2027	1.78/1.99/ 2.05/2.91	0.32/0.32/ 0.33/0.32
	**C_3F_7-cage**	0.32/0.29/ 0.28/0.38	1.85/2.14/ 2.25/3.57	5.51/9.48/ 15.6/97.5	13.0/27.0/ 65.0/429	2.02/2.35/ 2.48/3.63	0.32/0.30/ 0.29/0.36
	**C_4F_9-cage**	0.40/0.37/ 0.37/0.46	2.48/2.95/ 3.18/5.97	12.8/16.0/ 24.9/170	48.9/52.9/ 88.0/2713	2.75/3.17/ 3.38/5.03	0.38/0.36/ 0.35/0.42
	**C_5F_{11}-cage**	0.40/0.38/ 0.37/0.46	2.33/2.90/ 3.13/2.33	5.62/11.2/ 19.1/106	8.36/20.9/ 54.7/1212	2.51/3.05/ 3.25/4.60	0.39/0.39/ 0.39/0.39
	**C_6F_{13}-cage**	0.21/0.18/ 0.17/0.24	0.58/0.54/ 0.53/0.73	1.10/1.10/ 1.10/0.89	1.32/1.30/ 1.30/1.05	0.85/0.83/ 0.83/1.91	0.26/0.23/ 0.23/0.27
313 K	**CF_3-cage**	-	-	-	-	-	-
	**C_2F_5-cage**	0.23/0.20/ 0.19/0.28	1.69/1.87/ 1.94/2.82	4.48/7.41/ 10.0/24.0	8.12/16.8/ 36.4/654	1.91/2.10/ 2.16/2.67	0.29/0.29/ 0.29/0.29
	**C_3F_7-cage**	0.28/0.25/ 0.24/0.34	2.12/2.48/ 2.61/3.61	5.55/10.9/ 17.6/58.8	8.60/23.2/ 63.7/307	2.28/2.64/ 2.76/3.40	0.29/0.27/ 0.27/0.33
	**C_4F_9-cage**	0.35/0.32/ 0.31/0.41	2.66/3.23/ 3.45/5.04	7.61/15.6/ 27.3/95.5	12.4/32.2/ 90.7/1222	2.83/3.35/ 3.52/4.36	0.35/0.33/ 0.33/0.39
	**C_5F_{11}-cage**	0.36/0.33/ 0.33/0.40	2.62/3.16/ 3.35/4.74	7.05/13.6/ 21.5/60.1	9.94/22.6/ 48.8/318	2.78/3.24/ 3.38/4.09	0.35/0.35/ 0.35/0.35
	**C_6F_{13}-cage**	0.10/0.10/ 0.10/0.08	0.52/0.46/ 0.44/0.72	1.71/1.67/ 1.67/1.39	3.47/3.71/ 3.71/3.16	0.83/0.81/ 0.80/1.23	0.04/0.01/ 0.004/0.14

[a] $S_{IAST,\ 50:50}/S_{IAST,\ 10:90}/S_{IAST,\ 1:99}/S_{Henry}$; the red and green data represents the highest selectivity of $S_{IAST,\ 50:50}/S_{IAST,\ 10:90}/S_{IAST,\ 1:99}$ and S_{Henry}, respectively, for each gas at each respective temperature. - : The selectivity of F-gas over carbon dioxide for **CF_3-cage** can't be calculated because there is no valid F-gas uptake data available at 313 K.

IAST Selectivity at 273 K

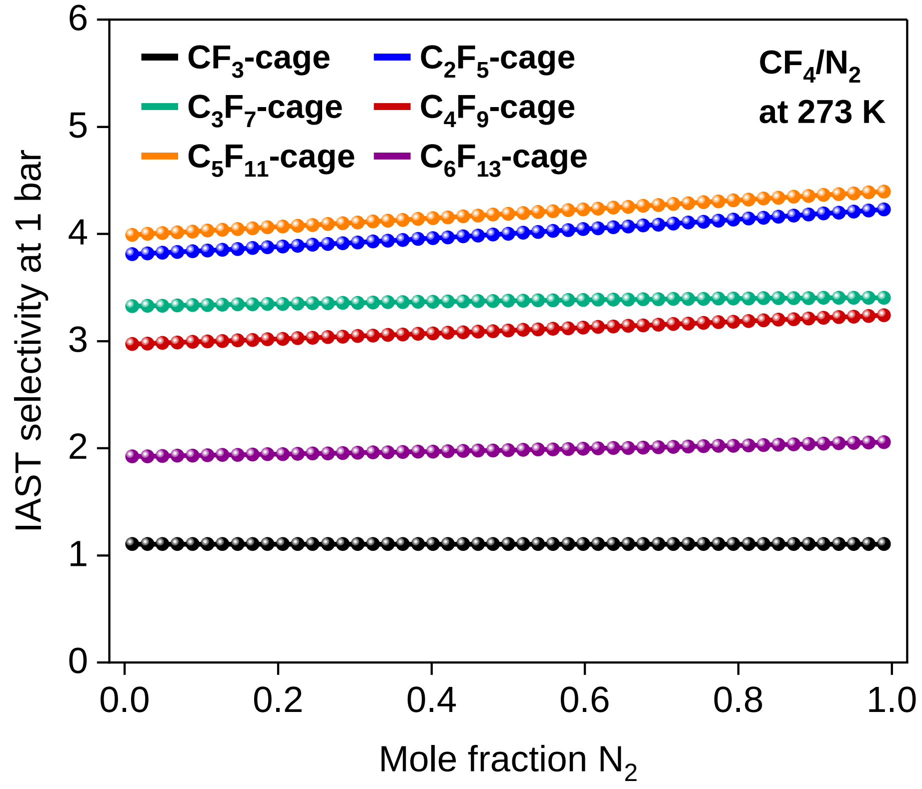

Figure S281. Composition dependent IAST selectivities for CF_4 over nitrogen at 273 K and one bar. Black: **CF_3-cage**; blue: **C_2F_5-cage**; green: **C_3F_7-cage**; red: **C_4F_8-cage**; orange: **C_5F_{11}-cage**; purple: **C_6F_{13}-cage**.

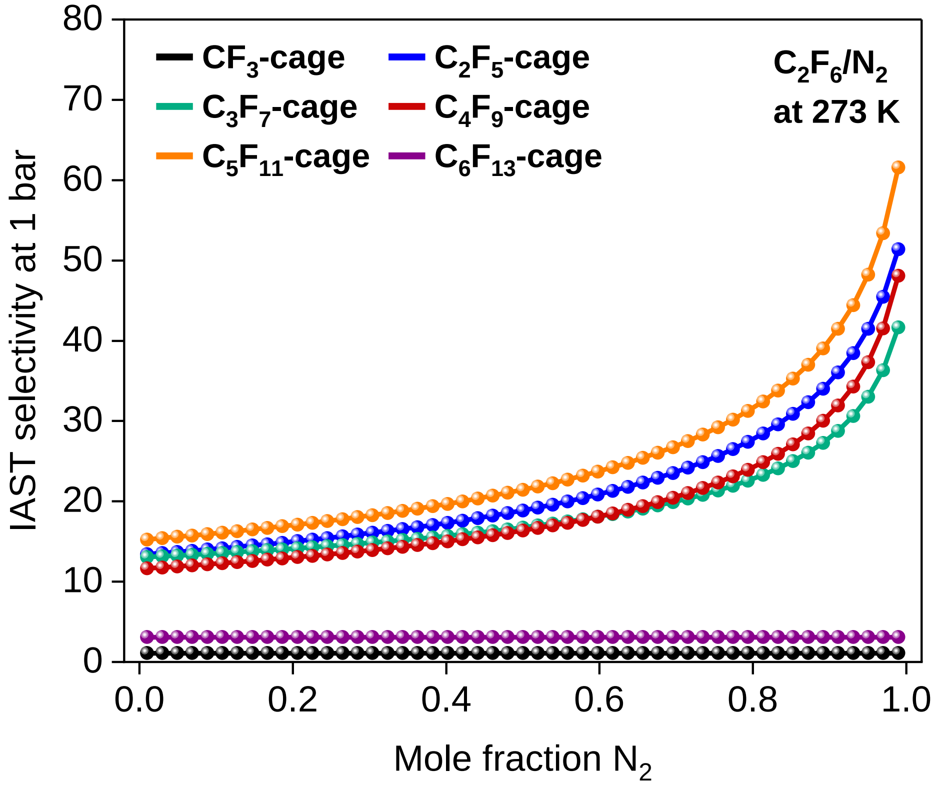

Figure S282. Composition dependent IAST selectivities for C_2F_6 over nitrogen at 273 K and one bar. Black: **CF_3-cage**; blue: **C_2F_5-cage**; green: **C_3F_7-cage**; red: **C_4F_8-cage**; orange: **C_5F_{11}-cage**; purple: **C_6F_{13}-cage**.

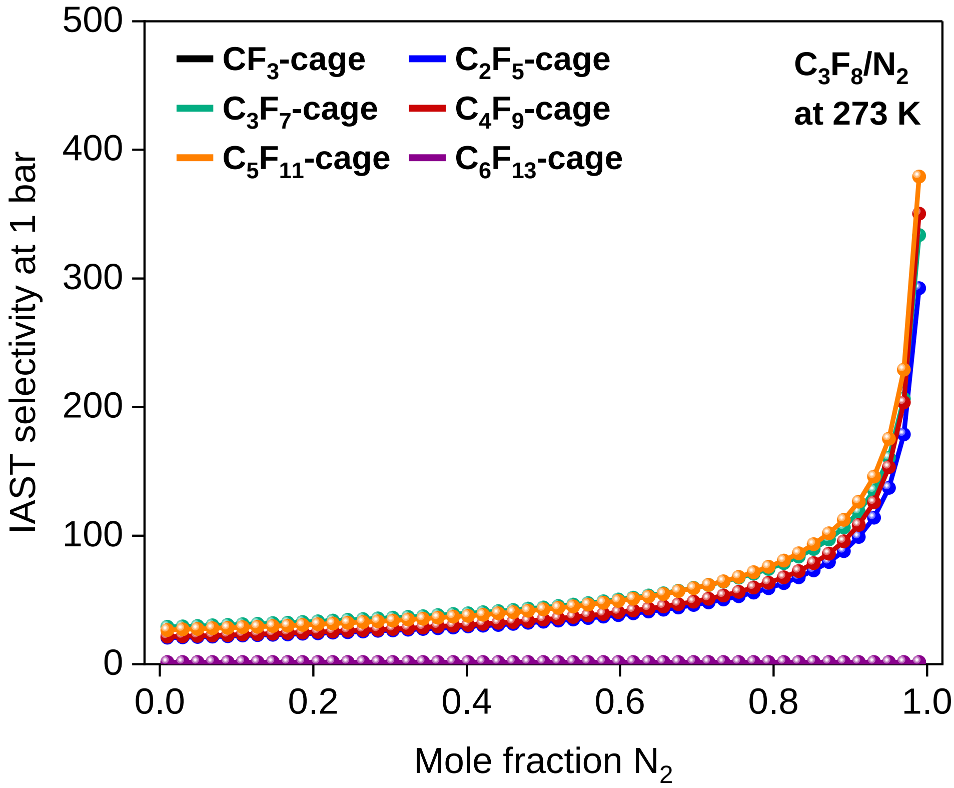

Figure S283. Composition dependent IAST selectivities for C_3F_8 over nitrogen at 273 K and one bar. Black: **CF_3-cage**; blue: **C_2F_5-cage**; green: **C_3F_7-cage**; red: **C_4F_8-cage**; orange: **C_5F_{11}-cage**; purple: **C_6F_{13}-cage**.

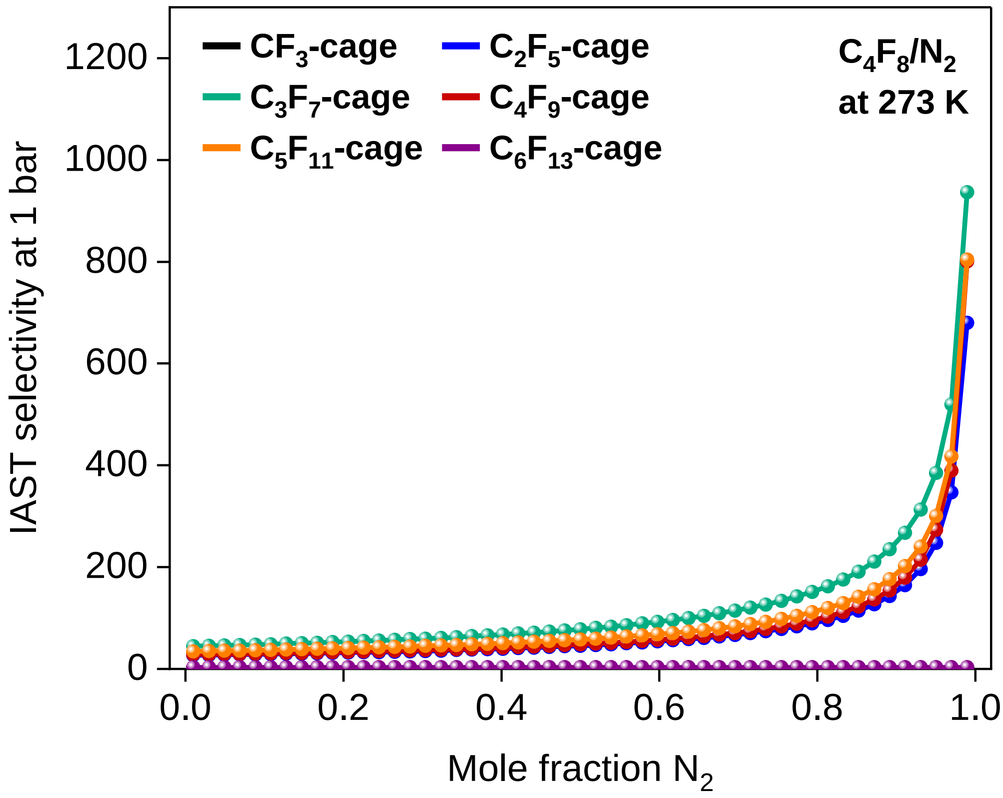

Figure S284. Composition dependent IAST selectivities for C_4F_8 over nitrogen at 273 K and one bar. Black: **CF_3-cage**; blue: **C_2F_5-cage**; green: **C_3F_7-cage**; red: **C_4F_8-cage**; orange: **C_5F_{11}-cage**; purple: **C_6F_{13}-cage**.

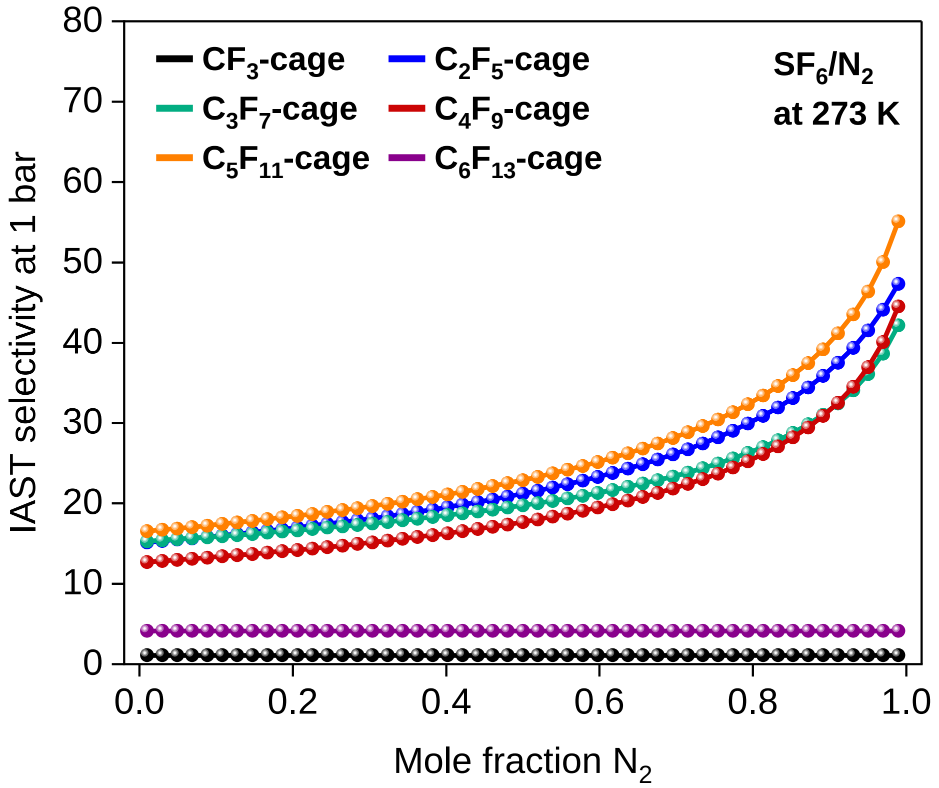

Figure S285. Composition dependent IAST selectivities for SF_6 over nitrogen at 273 K and one bar. Black: **CF_3-cage**; blue: **C_2F_5-cage**; green: **C_3F_7-cage**; red: **C_4F_8-cage**; orange: **C_5F_{11}-cage**; purple: **C_6F_{13}-cage**.

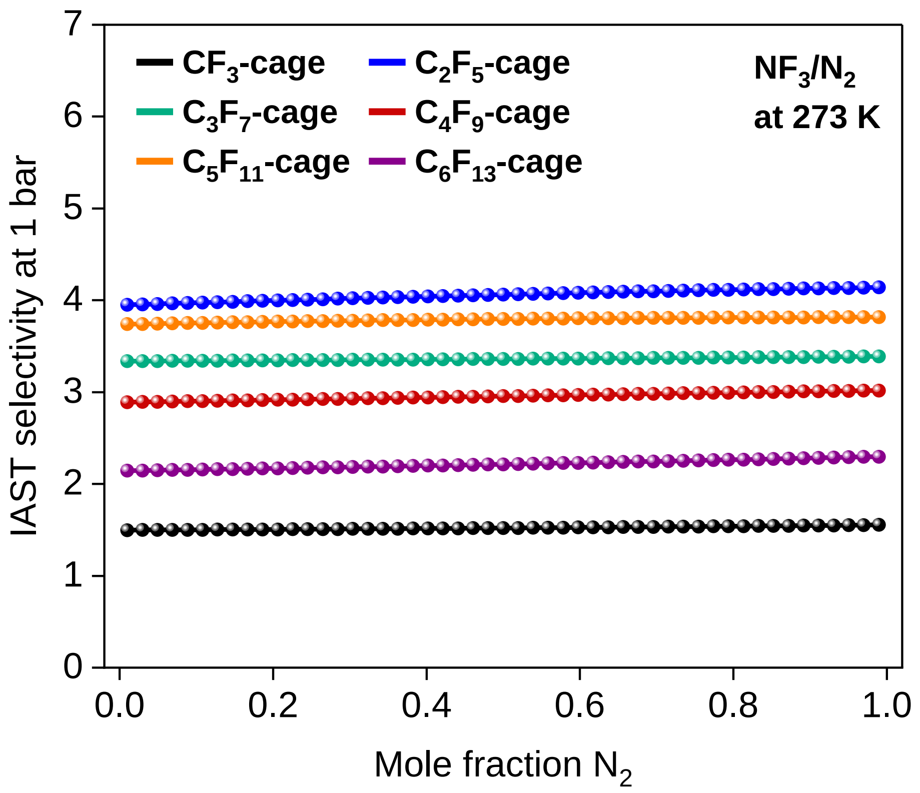

Figure S286. Composition dependent IAST selectivities for SF_6 over nitrogen at 273 K and one bar. Black: **CF_3-cage**; blue: **C_2F_5-cage**; green: **C_3F_7-cage**; red: **C_4F_8-cage**; orange: **C_5F_{11}-cage**; purple: **C_6F_{13}-cage**.

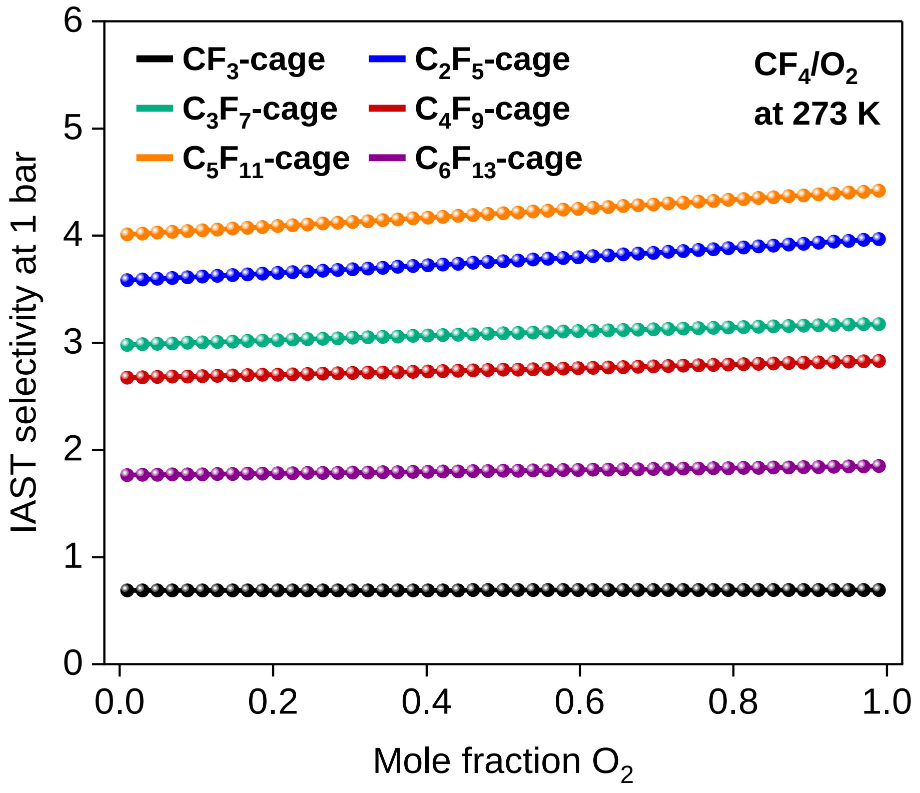

Figure S287. Composition dependent IAST selectivities for CF_4 over oxygen at 273 K and one bar. Black: **CF_3-cage**; blue: **C_2F_5-cage**; green: **C_3F_7-cage**; red: **C_4F_8-cage**; orange: **C_5F_{11}-cage**; purple: **C_6F_{13}-cage**.

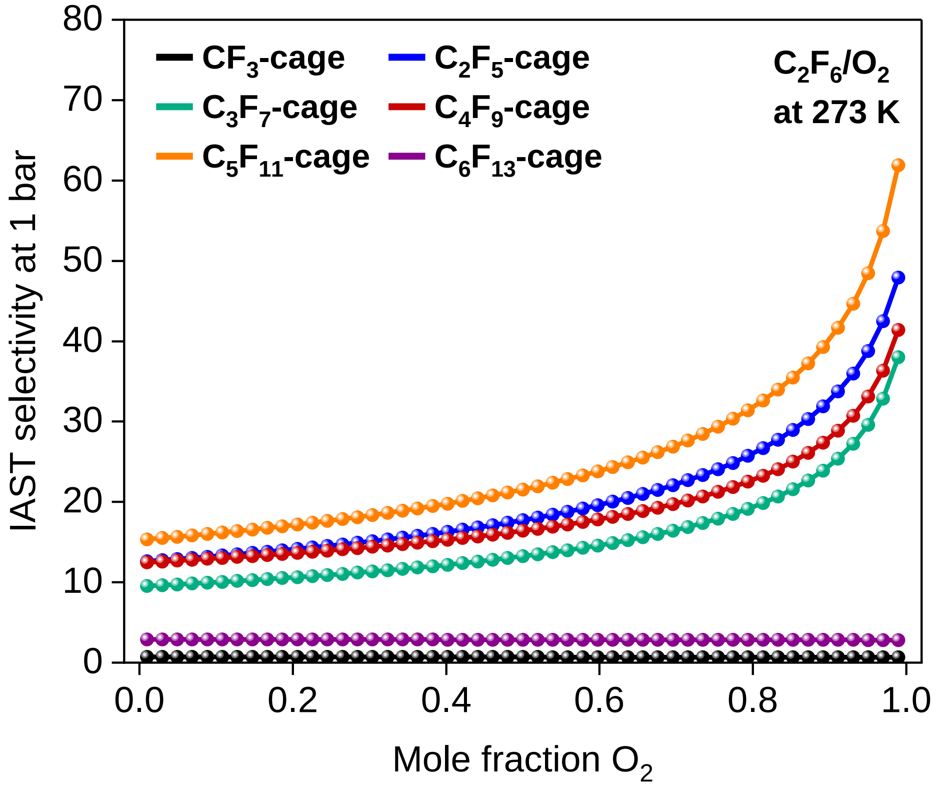

Figure S288. Composition dependent IAST selectivities for C_2F_6 over oxygen at 273 K and one bar. Black: **CF_3-cage**; blue: **C_2F_5-cage**; green: **C_3F_7-cage**; red: **C_4F_8-cage**; orange: **C_5F_{11}-cage**; purple: **C_6F_{13}-cage**.

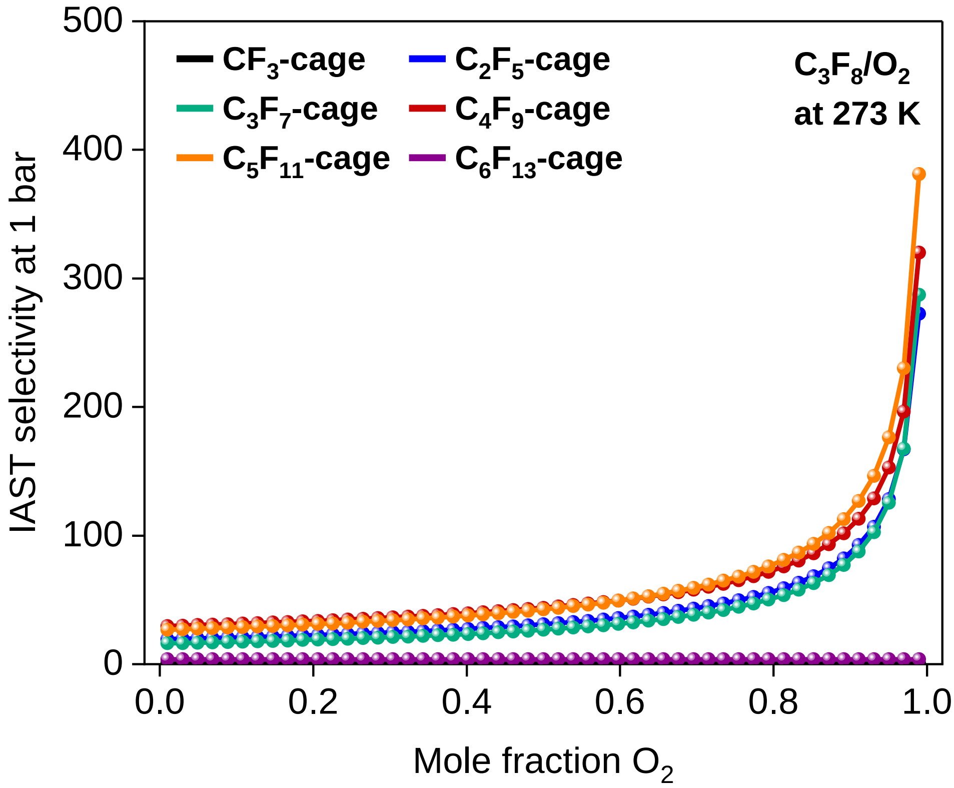

Figure S289. Composition dependent IAST selectivities for C_3F_8 over oxygen at 273 K and one bar. Black: **CF_3-cage**; blue: **C_2F_5-cage**; green: **C_3F_7-cage**; red: **C_4F_8-cage**; orange: **C_5F_{11}-cage**; purple: **C_6F_{13}-cage**.

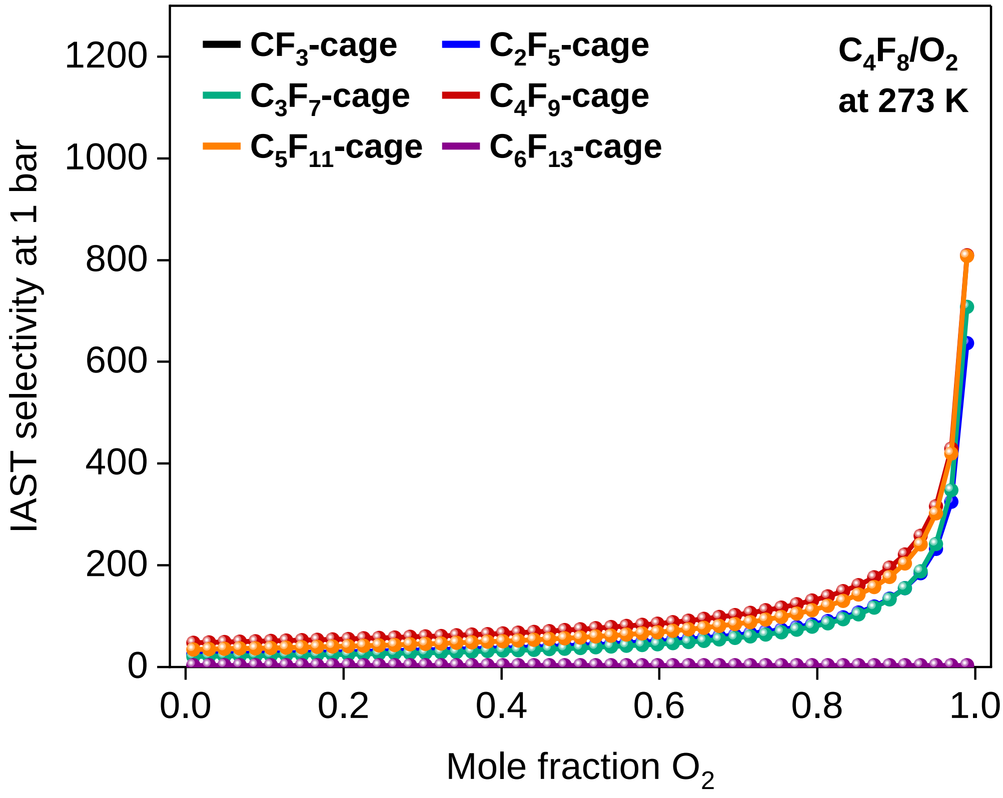

Figure S290. Composition dependent IAST selectivities for C_4F_8 over oxygen at 273 K and one bar. Black: **CF_3-cage**; blue: **C_2F_5-cage**; green: **C_3F_7-cage**; red: **C_4F_8-cage**; orange: **C_5F_{11}-cage**; purple: **C_6F_{13}-cage**.

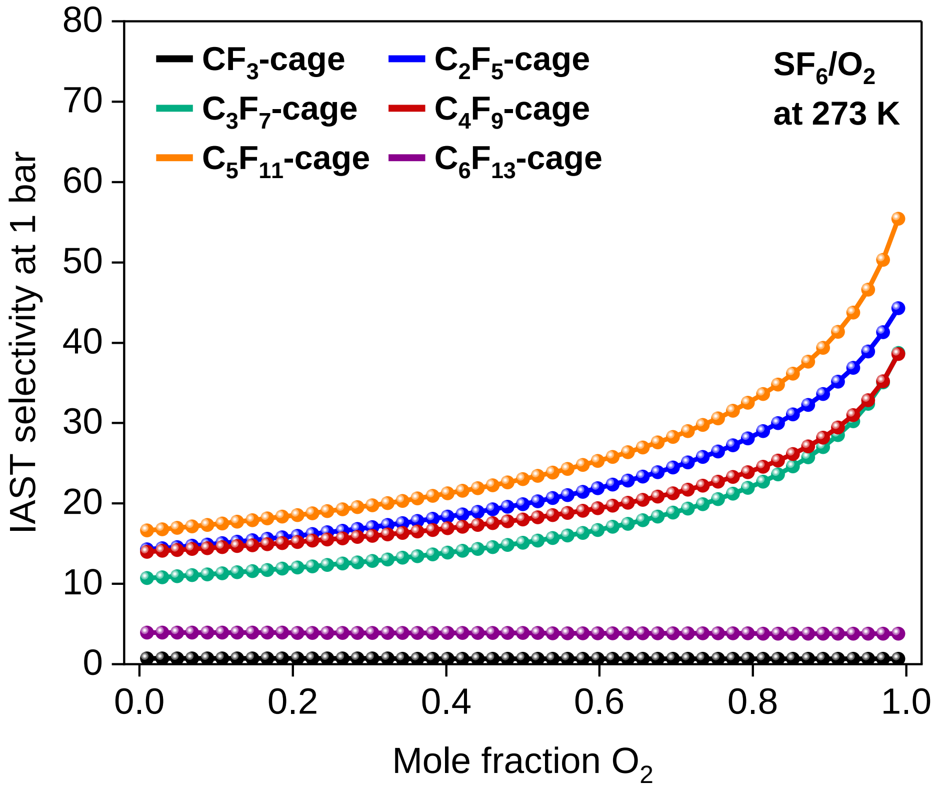

Figure S291. Composition dependent IAST selectivities for SF_6 over oxygen at 273 K and one bar. Black: **CF_3-cage**; blue: **C_2F_5-cage**; green: **C_3F_7-cage**; red: **C_4F_8-cage**; orange: **C_5F_{11}-cage**; purple: **C_6F_{13}-cage**.

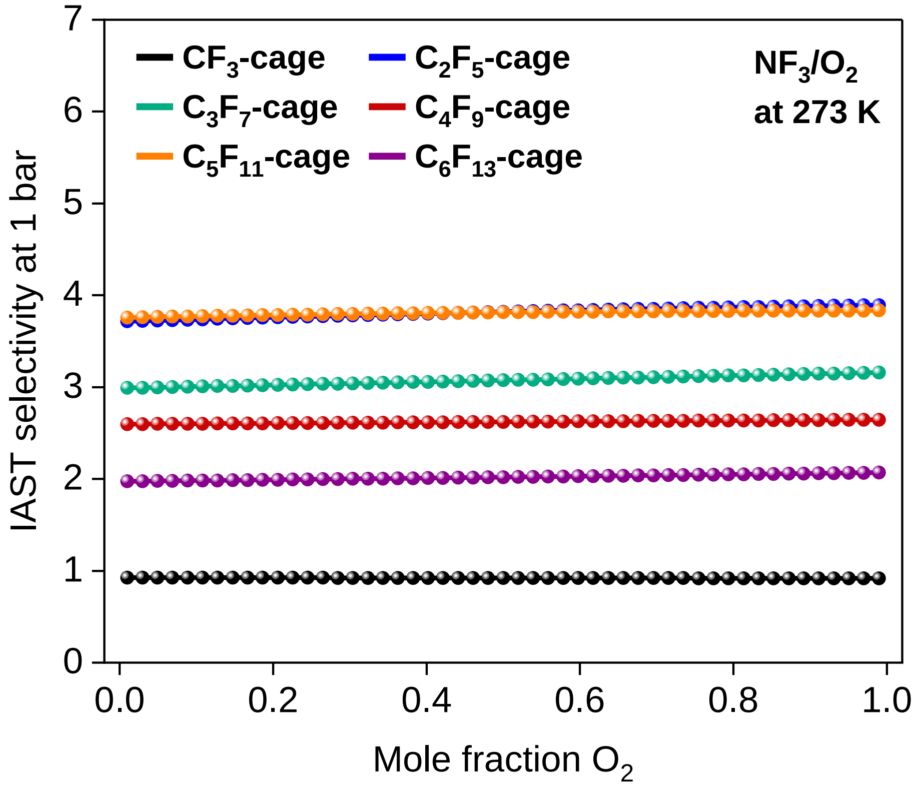

Figure S292. Composition dependent IAST selectivities for NF_3 over oxygen at 273 K and one bar. Black: **CF_3-cage**; blue: **C_2F_5-cage**; green: **C_3F_7-cage**; red: **C_4F_8-cage**; orange: **C_5F_{11}-cage**; purple: **C_6F_{13}-cage**.

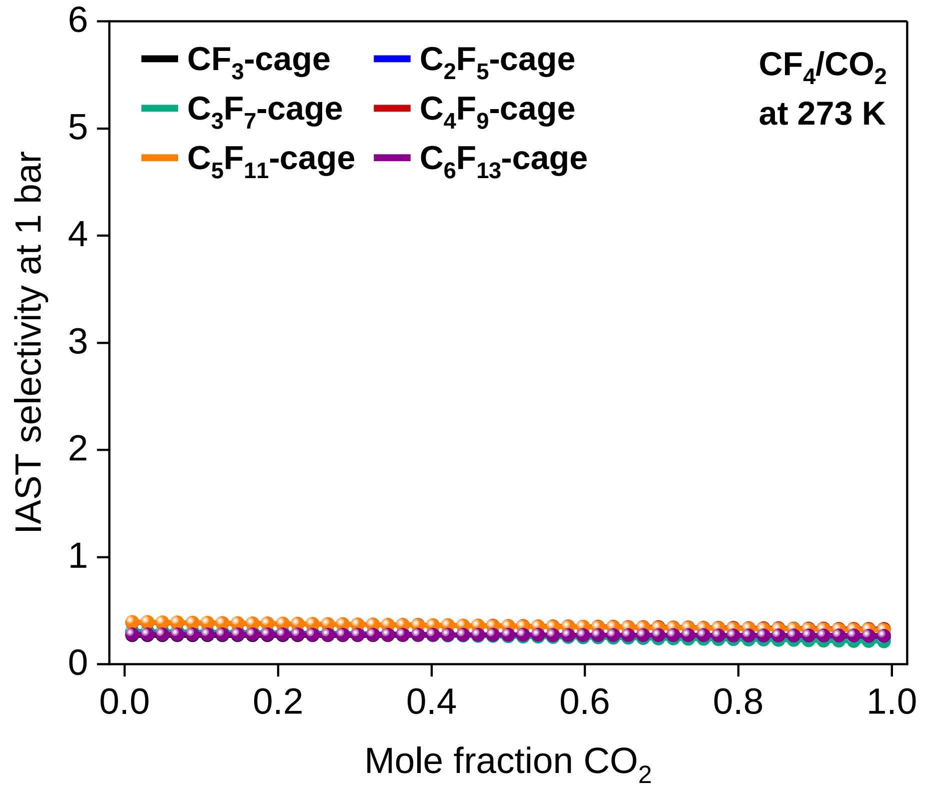

Figure S293. Composition dependent IAST selectivities for CF_4 over carbon dioxide at 273 K and one bar. Black: **CF_3-cage**; blue: **C_2F_5-cage**; green: **C_3F_7-cage**; red: **C_4F_8-cage**; orange: **C_5F_{11}-cage**; purple: **C_6F_{13}-cage**.

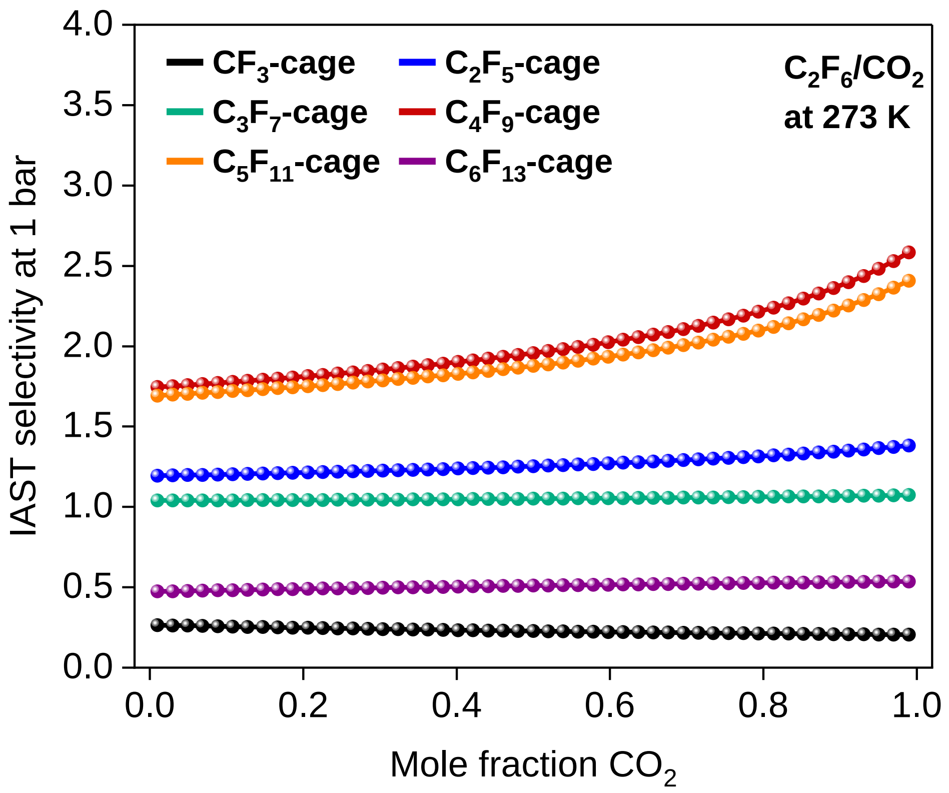

Figure S294. Composition dependent IAST selectivities for C_2F_6 over carbon dioxide at 273 K and one bar. Black: **CF_3-cage**; blue: **C_2F_5-cage**; green: **C_3F_7-cage**; red: **C_4F_8-cage**; orange: **C_5F_{11}-cage**; purple: **C_6F_{13}-cage**.

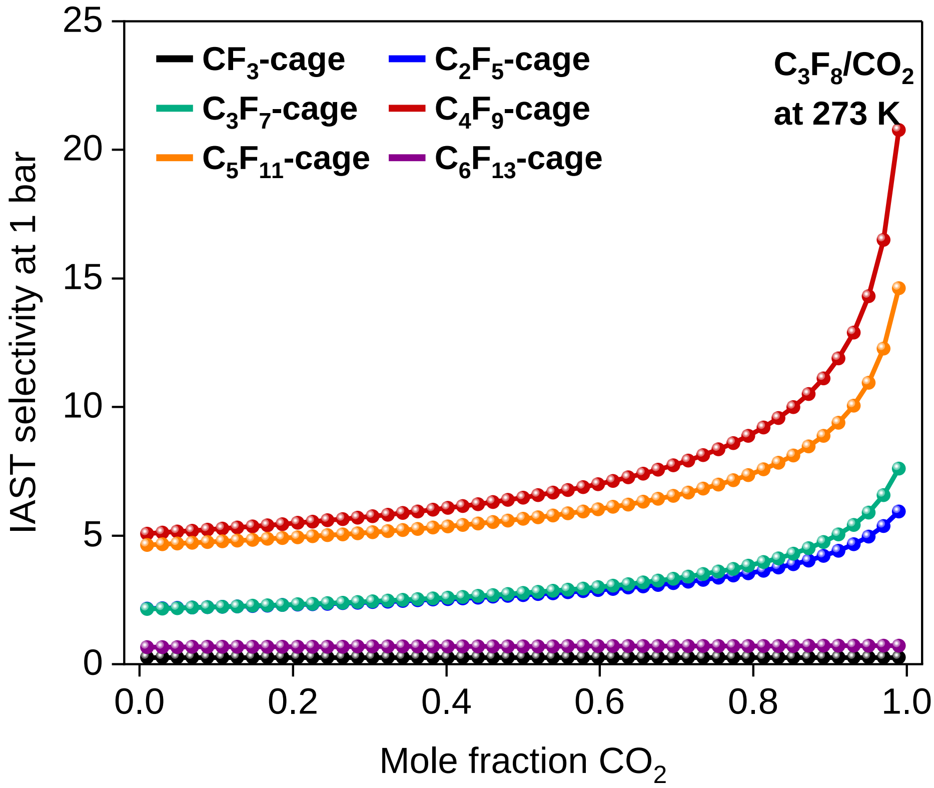

Figure S295. Composition dependent IAST selectivities for C_3F_8 over carbon dioxide at 273 K and one bar. Black: **CF_3-cage**; blue: **C_2F_5-cage**; green: **C_3F_7-cage**; red: **C_4F_8-cage**; orange: **C_5F_{11}-cage**; purple: **C_6F_{13}-cage**.

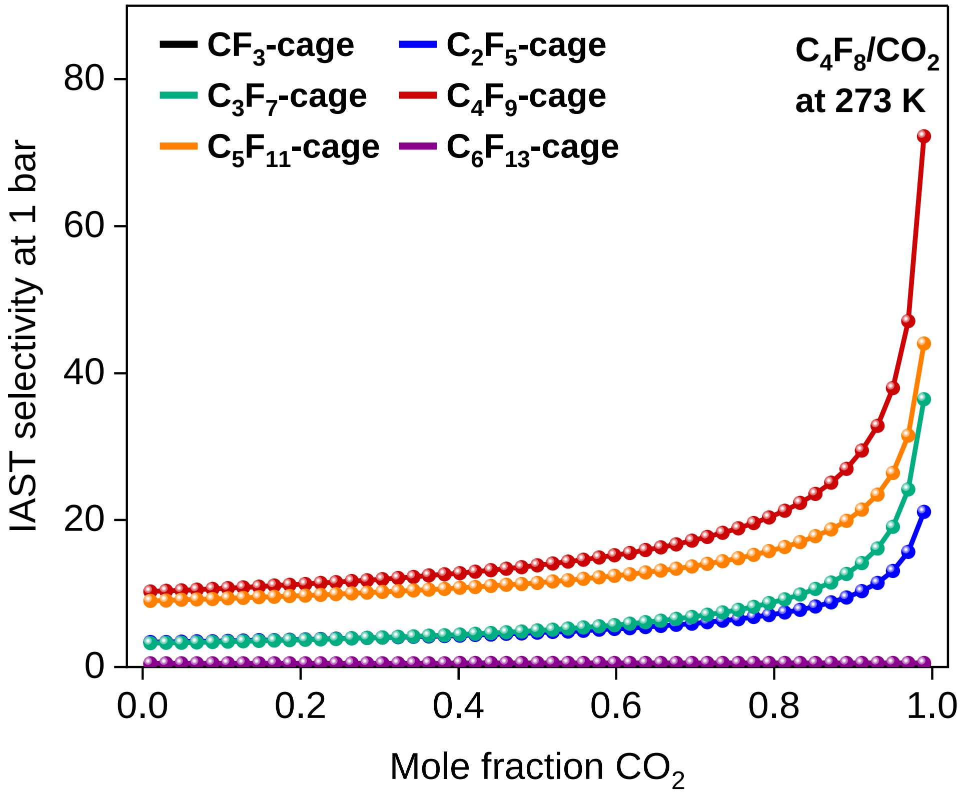

Figure S296. Composition dependent IAST selectivities for C_4F_8 over carbon dioxide at 273 K and one bar. Black: **CF_3-cage**; blue: **C_2F_5-cage**; green: **C_3F_7-cage**; red: **C_4F_8-cage**; orange: **C_5F_{11}-cage**; purple: **C_6F_{13}-cage**.

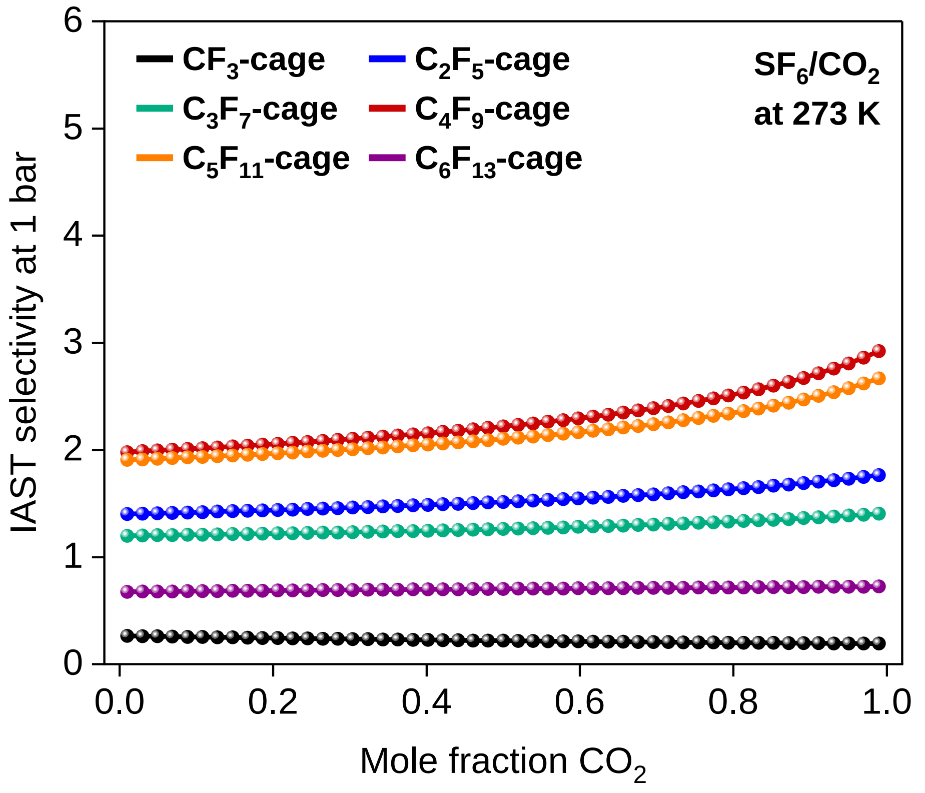

Figure S297. Composition dependent IAST selectivities for SF_6 over carbon dioxide at 273 K and one bar. Black: **CF_3-cage**; blue: **C_2F_5-cage**; green: **C_3F_7-cage**; red: **C_4F_8-cage**; orange: **C_5F_{11}-cage**; purple: **C_6F_{13}-cage**.

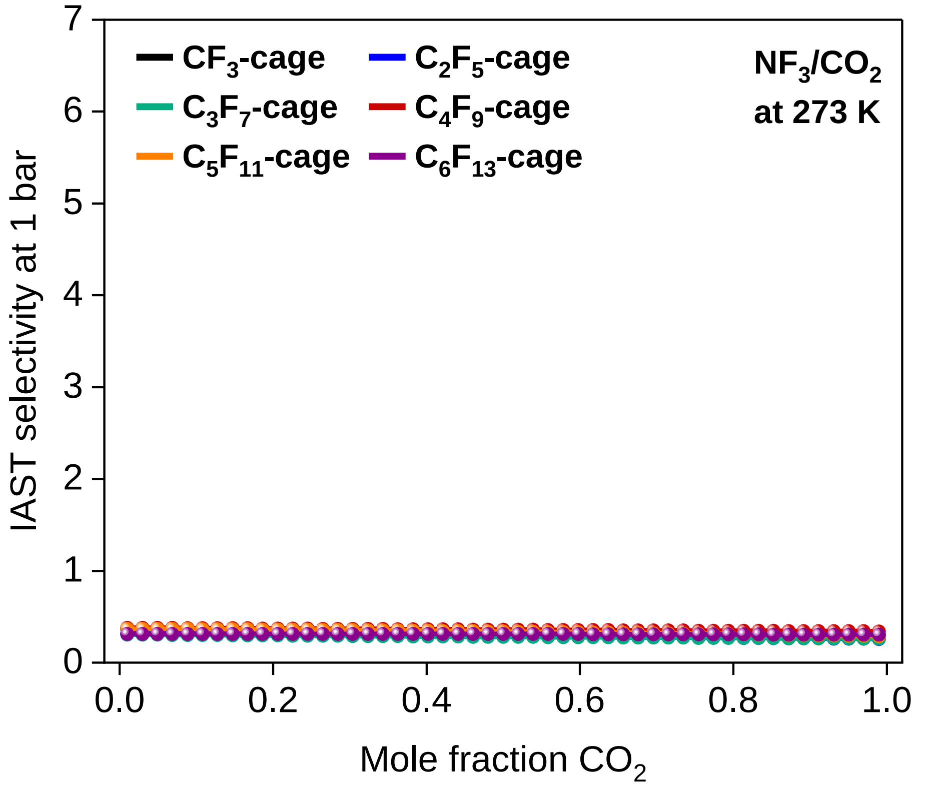

Figure S298. Composition dependent IAST selectivities for SF_6 over carbon dioxide at 273 K and one bar. Black: **CF_3-cage**; blue: **C_2F_5-cage**; green: **C_3F_7-cage**; red: **C_4F_8-cage**; orange: **C_5F_{11}-cage**; purple: **C_6F_{13}-cage**.

IAST Selectivity Curves at 283 K

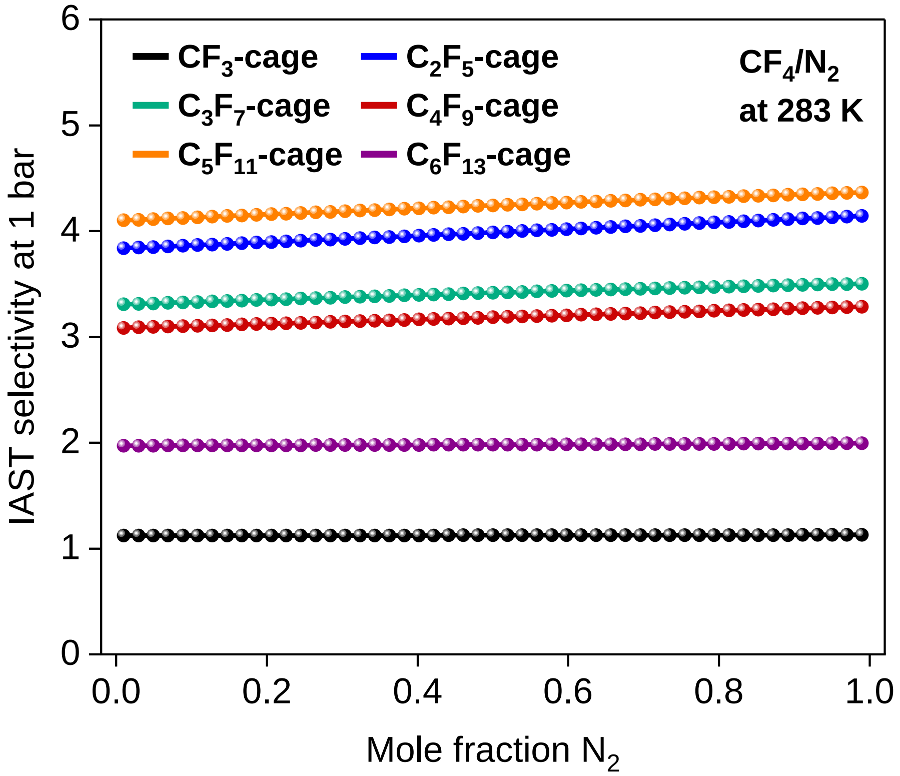

Figure S299. Composition dependent IAST selectivities for CF_4 over nitrogen at 283 K and one bar. Black: **CF_3-cage**; blue: **C_2F_5-cage**; green: **C_3F_7-cage**; red: **C_4F_8-cage**; orange: **C_5F_{11}-cage**; purple: **C_6F_{13}-cage**.

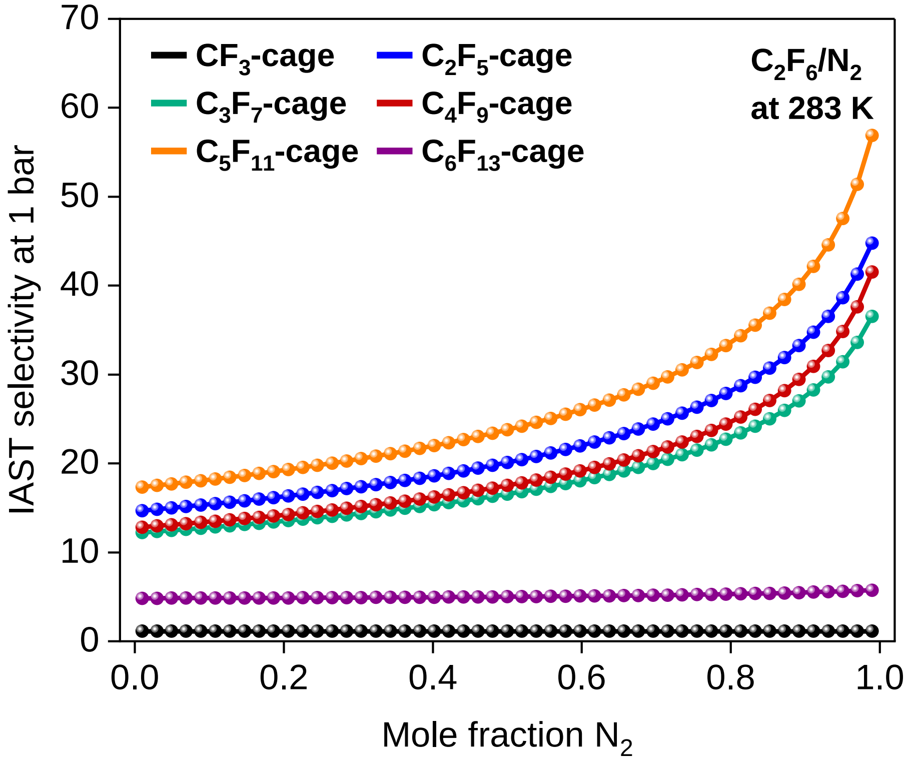

Figure S300. Composition dependent IAST selectivities for C_2F_6 over nitrogen at 283 K and one bar. Black: **CF_3-cage**; blue: **C_2F_5-cage**; green: **C_3F_7-cage**; red: **C_4F_8-cage**; orange: **C_5F_{11}-cage**; purple: **C_6F_{13}-cage**.

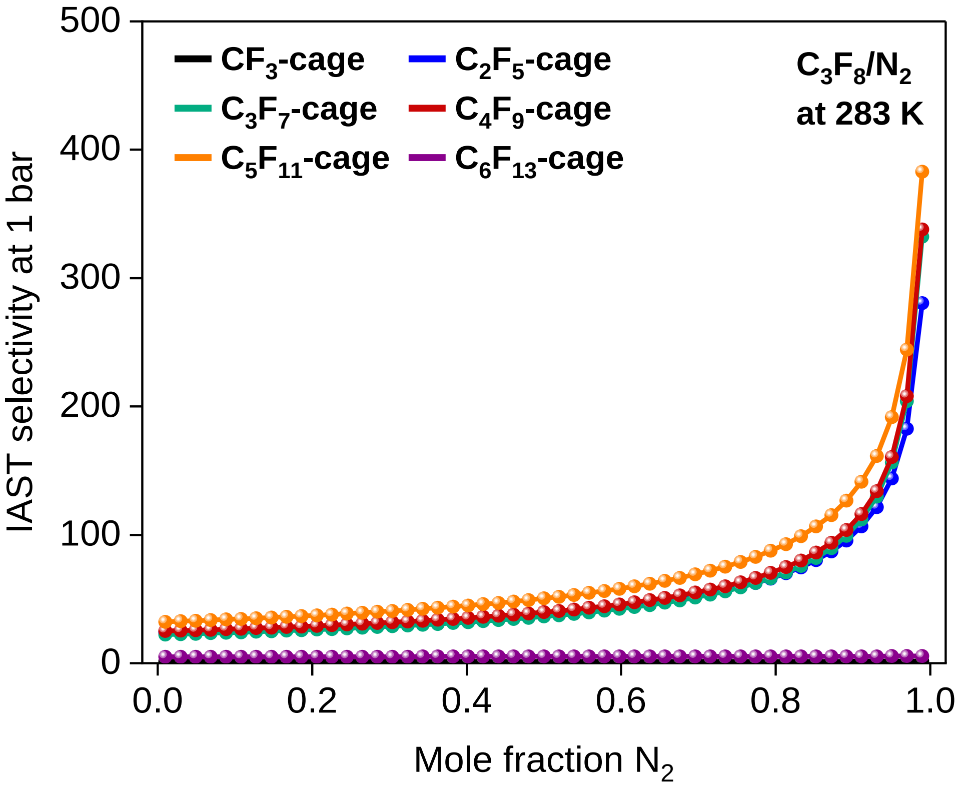

Figure S301. Composition dependent IAST selectivities for C_3F_8 over nitrogen at 283 K and one bar. Black: **CF_3-cage**; blue: **C_2F_5-cage**; green: **C_3F_7-cage**; red: **C_4F_8-cage**; orange: **C_5F_{11}-cage**; purple: **C_6F_{13}-cage**.

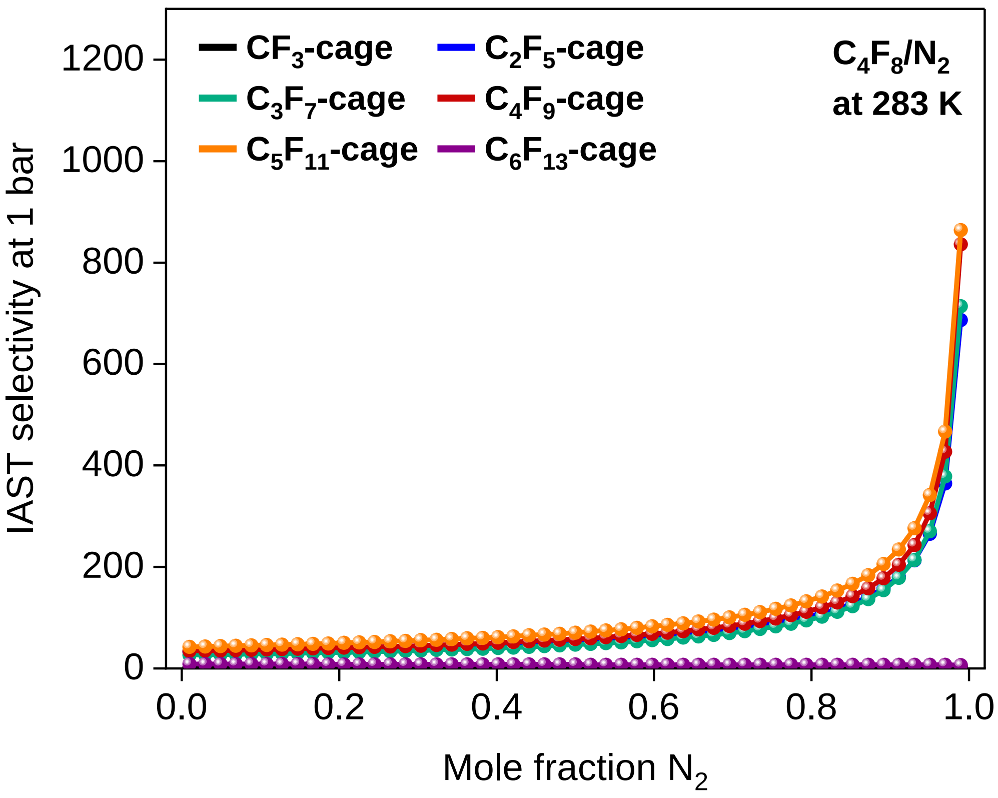

Figure S302. Composition dependent IAST selectivities for C_4F_8 over nitrogen at 283 K and one bar. Black: **CF_3-cage**; blue: **C_2F_5-cage**; green: **C_3F_7-cage**; red: **C_4F_8-cage**; orange: **C_5F_{11}-cage**; purple: **C_6F_{13}-cage**.

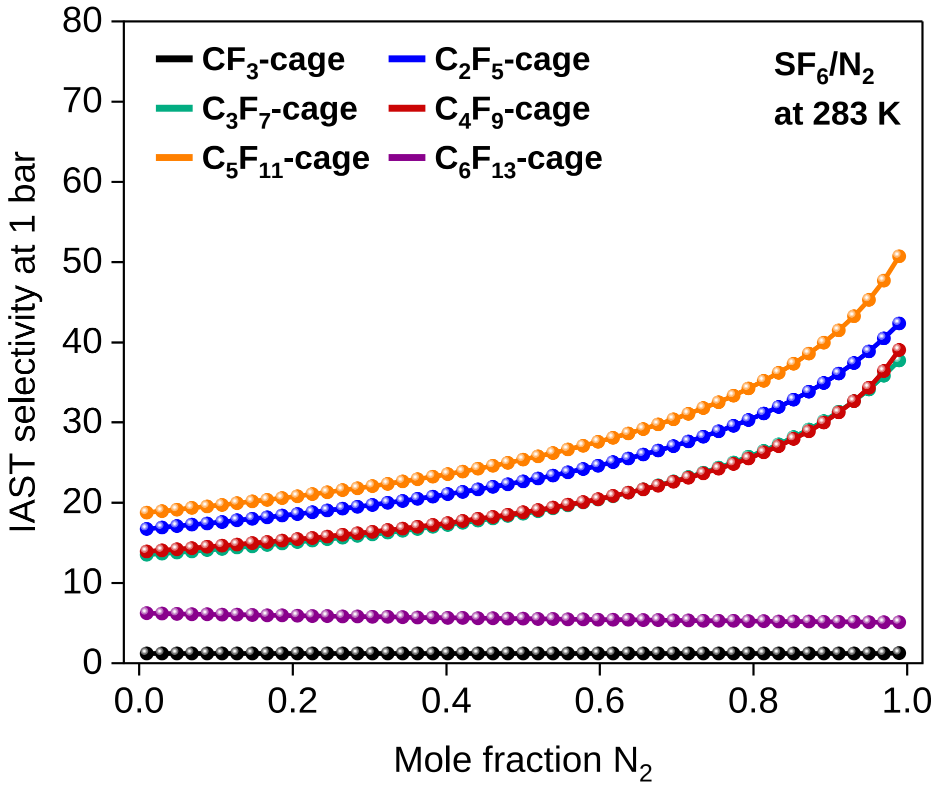

Figure S303. Composition dependent IAST selectivities for SF_6 over nitrogen at 283 K and one bar. Black: **CF_3-cage**; blue: **C_2F_5-cage**; green: **C_3F_7-cage**; red: **C_4F_8-cage**; orange: **C_5F_{11}-cage**; purple: **C_6F_{13}-cage**.

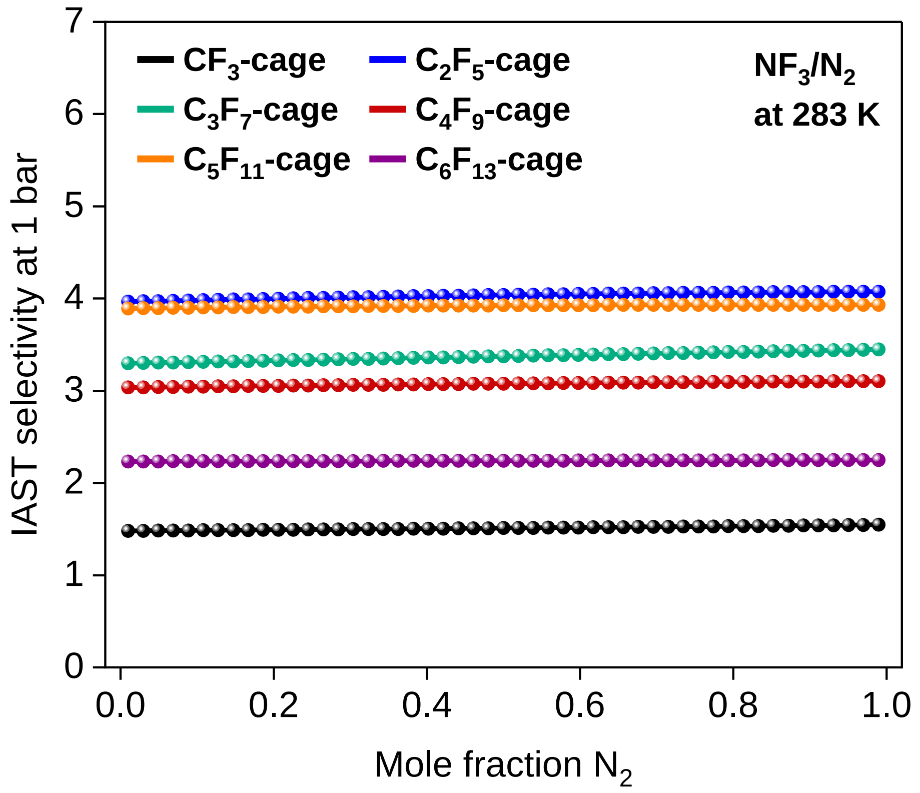

Figure S304. Composition dependent IAST selectivities for NF_3 over nitrogen at 283 K and one bar. Black: **CF_3-cage**; blue: **C_2F_5-cage**; green: **C_3F_7-cage**; red: **C_4F_8-cage**; orange: **C_5F_{11}-cage**; purple: **C_6F_{13}-cage**.

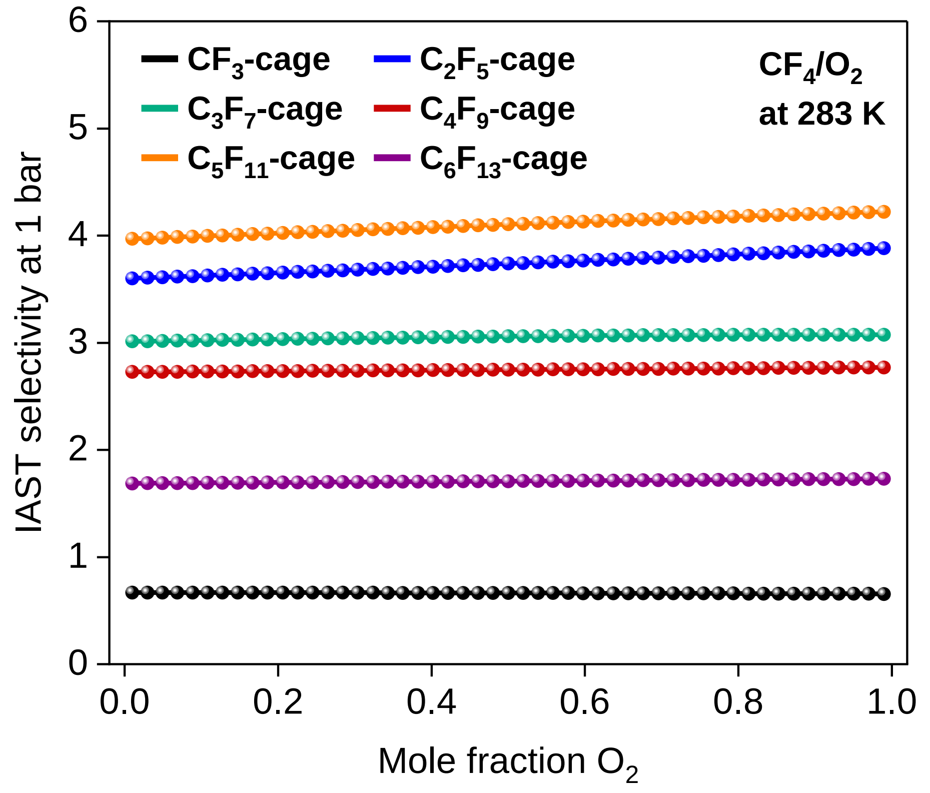

Figure S305. Composition dependent IAST selectivities for CF_4 over oxygen at 283 K and one bar. Black: **CF_3-cage**; blue: **C_2F_5-cage**; green: **C_3F_7-cage**; red: **C_4F_8-cage**; orange: **C_5F_{11}-cage**; purple: **C_6F_{13}-cage**.

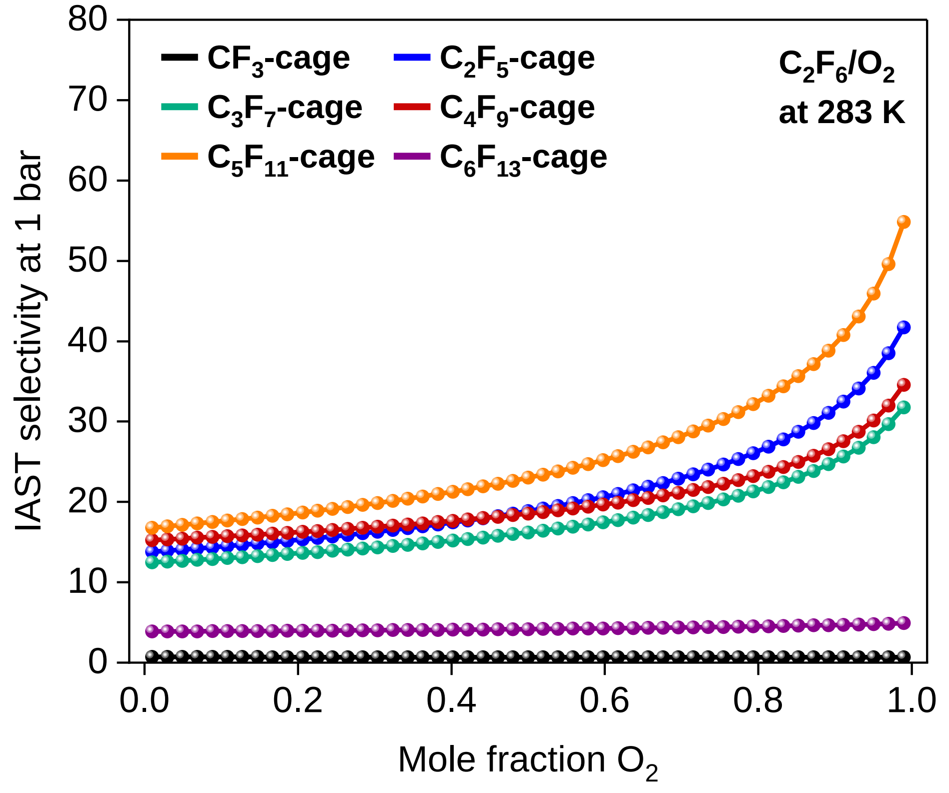

Figure S306. Composition dependent IAST selectivities for C_2F_6 over oxygen at 283 K and one bar. Black: **CF_3-cage**; blue: **C_2F_5-cage**; green: **C_3F_7-cage**; red: **C_4F_8-cage**; orange: **C_5F_{11}-cage**; purple: **C_6F_{13}-cage**.

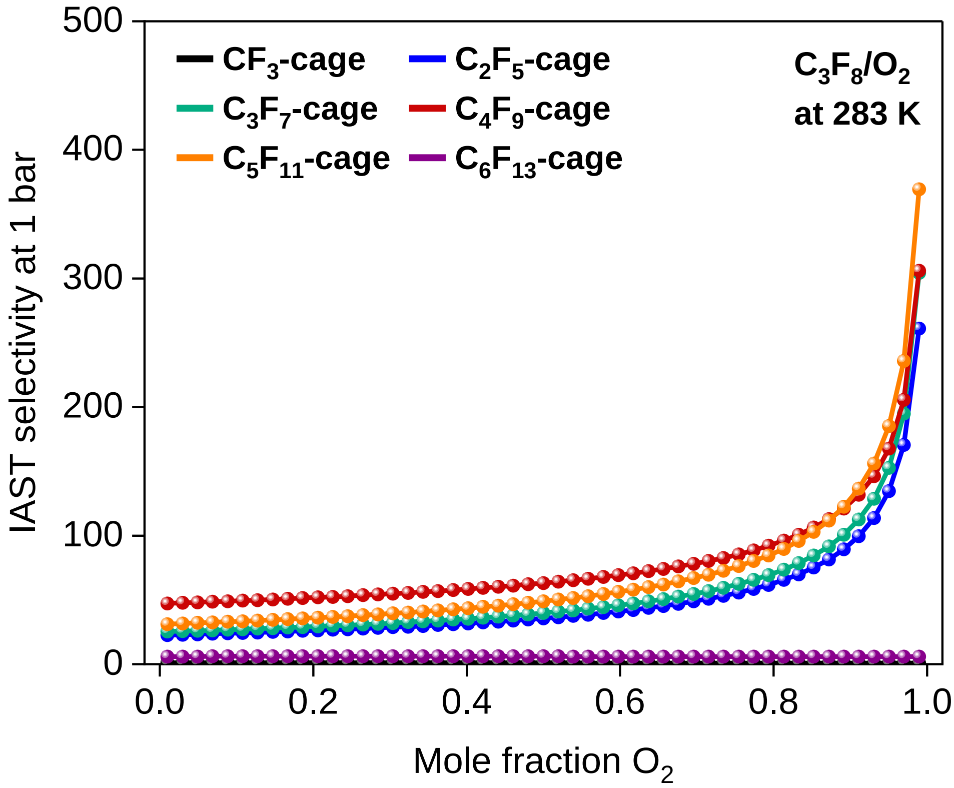

Figure S307. Composition dependent IAST selectivities for C_3F_8 over oxygen at 283 K and one bar. Black: **CF_3-cage**; blue: **C_2F_5-cage**; green: **C_3F_7-cage**; red: **C_4F_8-cage**; orange: **C_5F_{11}-cage**; purple: **C_6F_{13}-cage**.

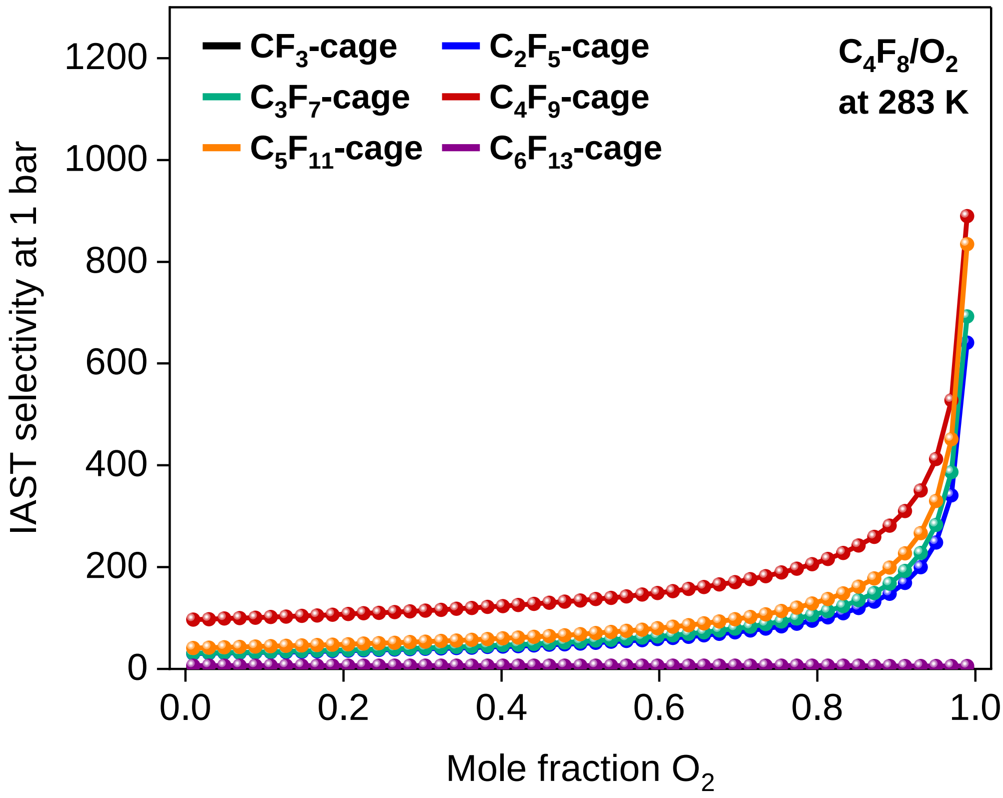

Figure S308. Composition dependent IAST selectivities for C_4F_8 over oxygen at 283 K and one bar. Black: **CF_3-cage**; blue: **C_2F_5-cage**; green: **C_3F_7-cage**; red: **C_4F_8-cage**; orange: **C_5F_{11}-cage**; purple: **C_6F_{13}-cage**.

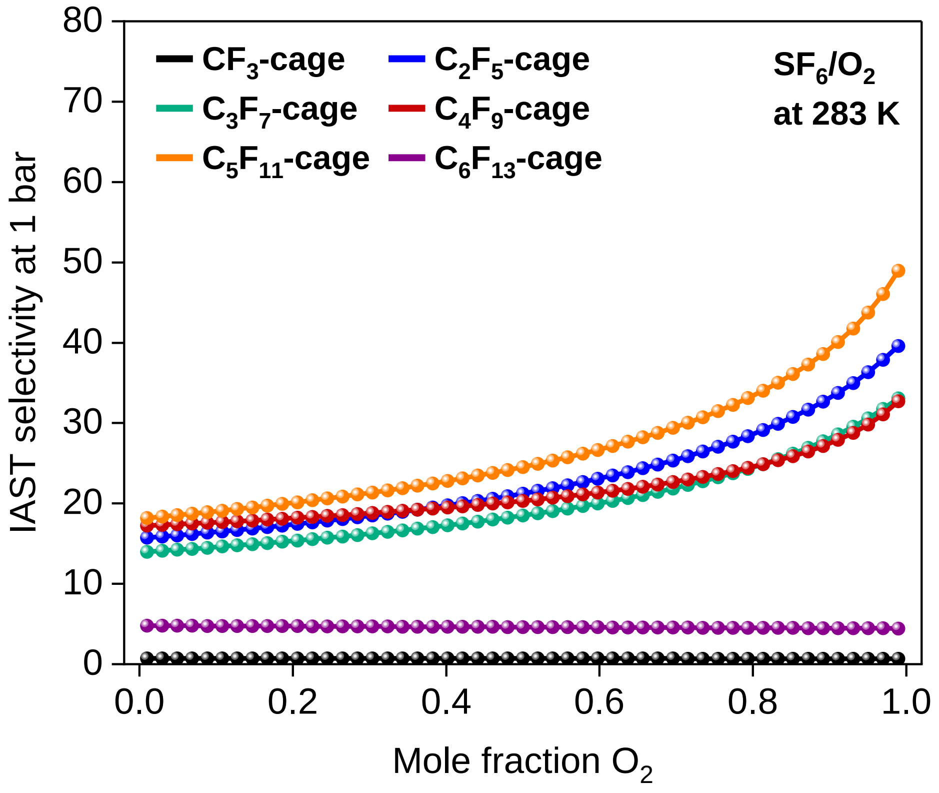

Figure S309. Composition dependent IAST selectivities for SF_6 over oxygen at 283 K and one bar. Black: **CF_3-cage**; blue: **C_2F_5-cage**; green: **C_3F_7-cage**; red: **C_4F_8-cage**; orange: **C_5F_{11}-cage**; purple: **C_6F_{13}-cage**.

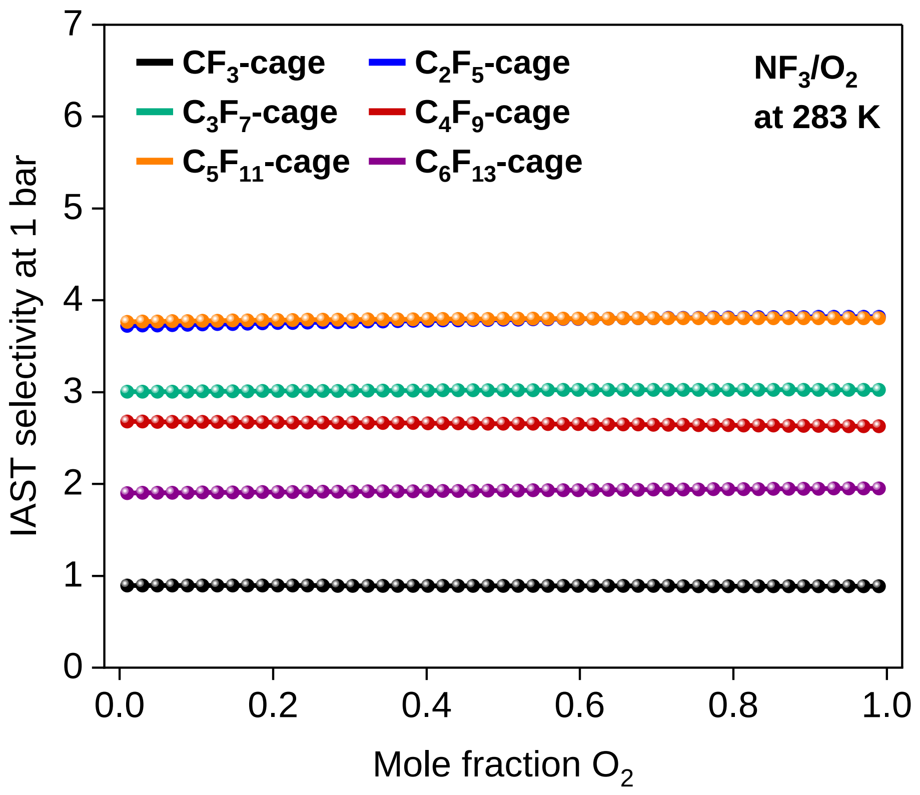

Figure S310. Composition dependent IAST selectivities for NF_3 over oxygen at 283 K and one bar. Black: **CF_3-cage**; blue: **C_2F_5-cage**; green: **C_3F_7-cage**; red: **C_4F_8-cage**; orange: **C_5F_{11}-cage**; purple: **C_6F_{13}-cage**.

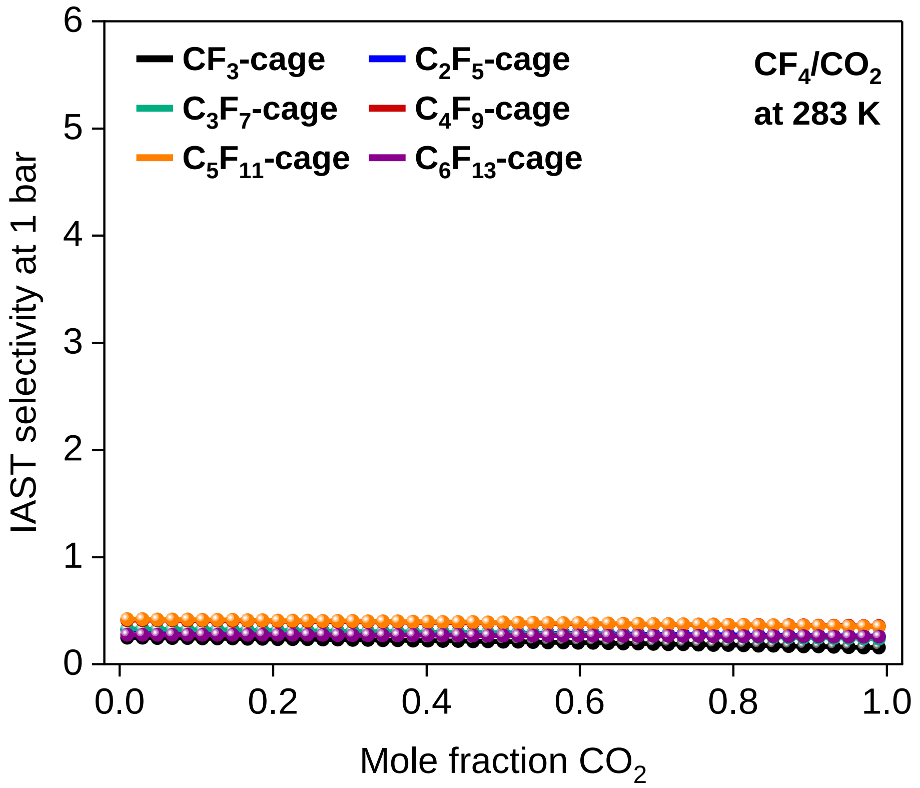

Figure S311. Composition dependent IAST selectivities for CF_4 over carbon dioxide at 283 K and one bar. Black: **CF_3-cage**; blue: **C_2F_5-cage**; green: **C_3F_7-cage**; red: **C_4F_8-cage**; orange: **C_5F_{11}-cage**; purple: **C_6F_{13}-cage**.

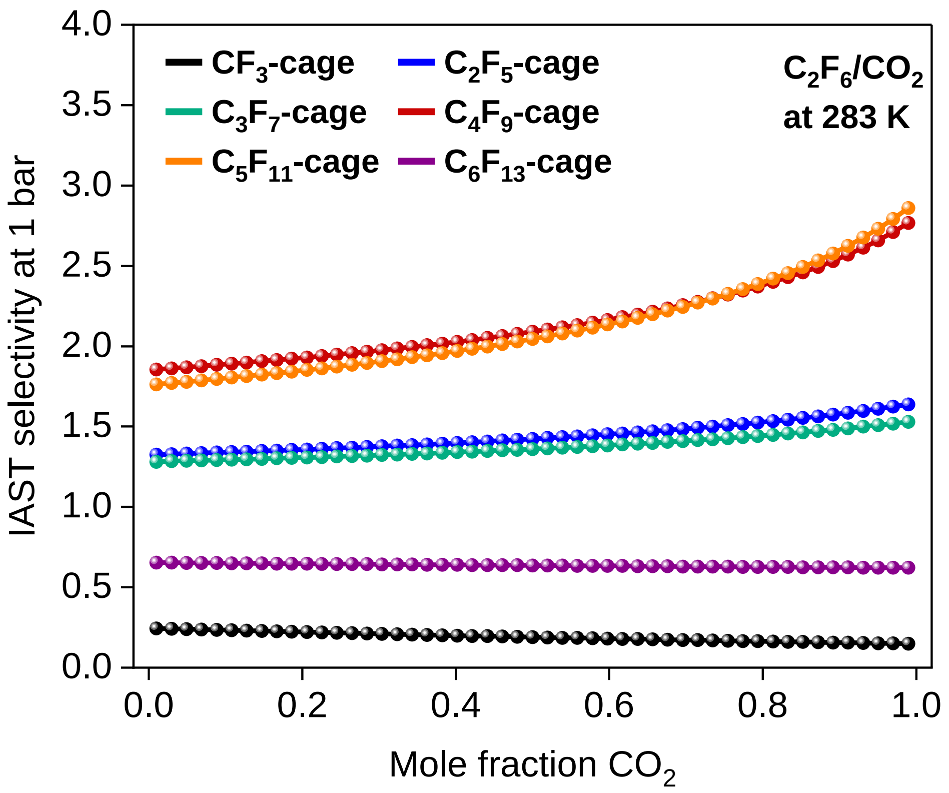

Figure S312. Composition dependent IAST selectivities for C_2F_6 over carbon dioxide at 283 K and one bar. Black: **CF_3-cage**; blue: **C_2F_5-cage**; green: **C_3F_7-cage**; red: **C_4F_8-cage**; orange: **C_5F_{11}-cage**; purple: **C_6F_{13}-cage**.

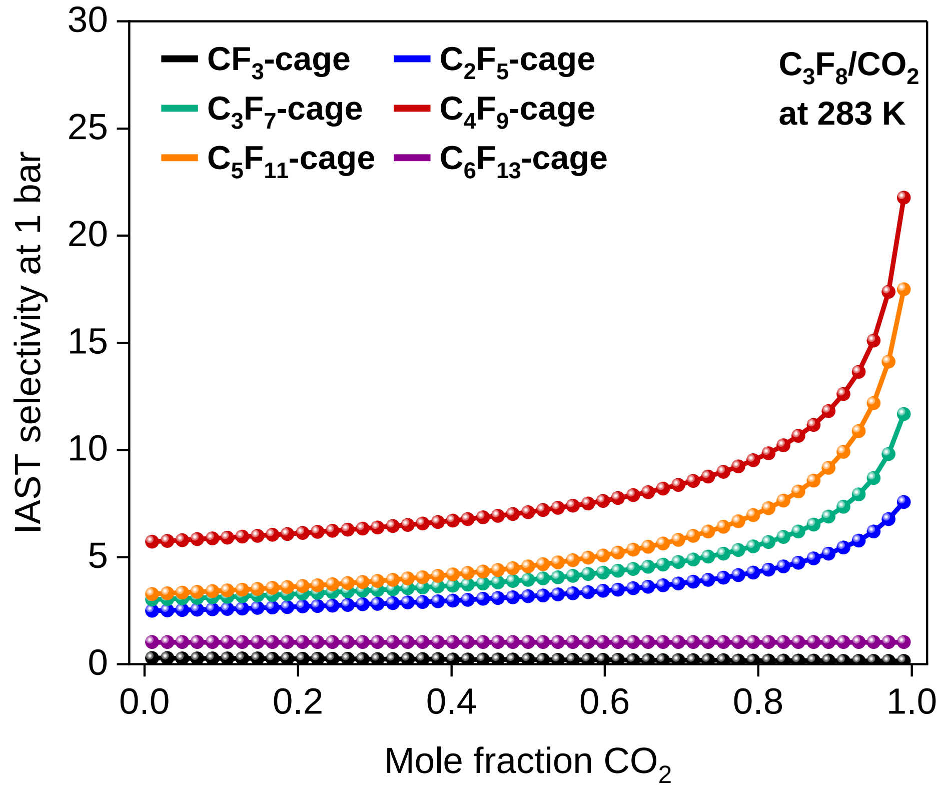

Figure S313. Composition dependent IAST selectivities for C_3F_8 over carbon dioxide at 283 K and one bar. Black: **CF_3-cage**; blue: **C_2F_5-cage**; green: **C_3F_7-cage**; red: **C_4F_8-cage**; orange: **C_5F_{11}-cage**; purple: **C_6F_{13}-cage**.

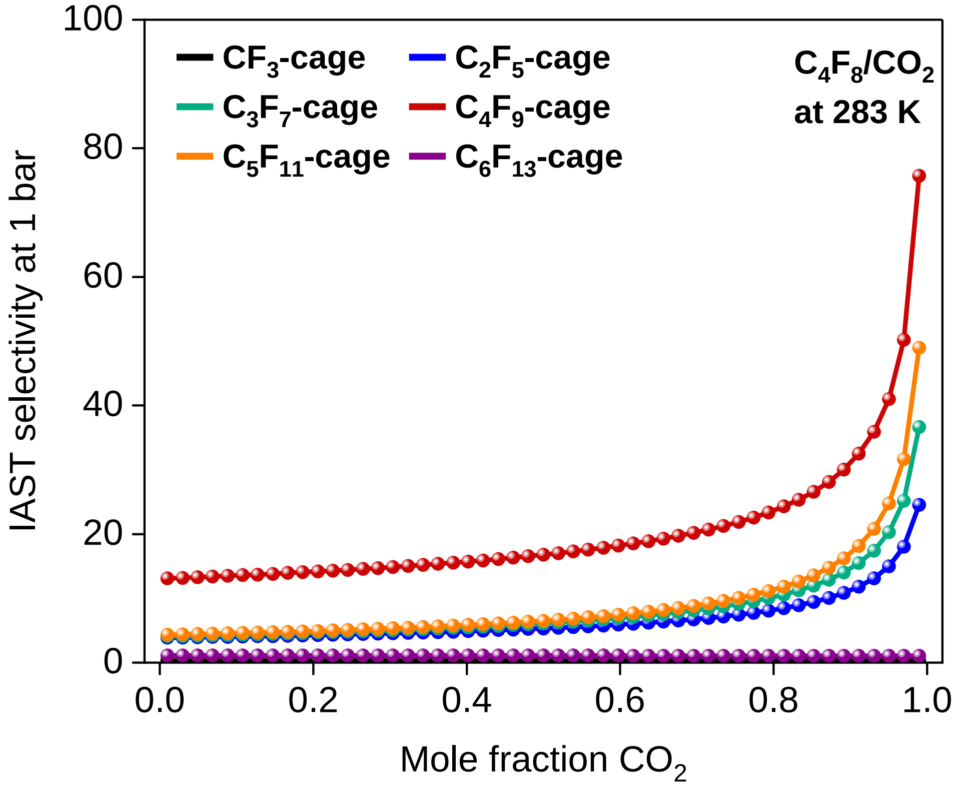

Figure S314. Composition dependent IAST selectivities for C_4F_8 over carbon dioxide at 283 K and one bar. Black: **CF_3-cage**; blue: **C_2F_5-cage**; green: **C_3F_7-cage**; red: **C_4F_8-cage**; orange: **C_5F_{11}-cage**; purple: **C_6F_{13}-cage**.

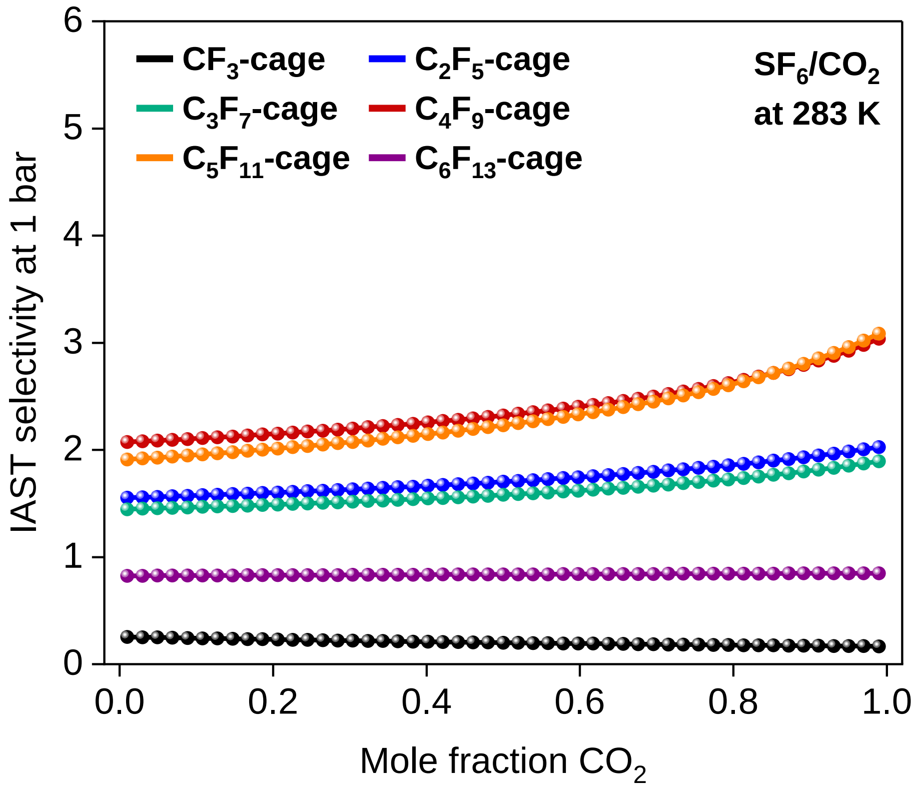

Figure S315. Composition dependent IAST selectivities for SF_6 over carbon dioxide at 283 K and one bar. Black: **CF_3-cage**; blue: **C_2F_5-cage**; green: **C_3F_7-cage**; red: **C_4F_8-cage**; orange: **C_5F_{11}-cage**; purple: **C_6F_{13}-cage**.

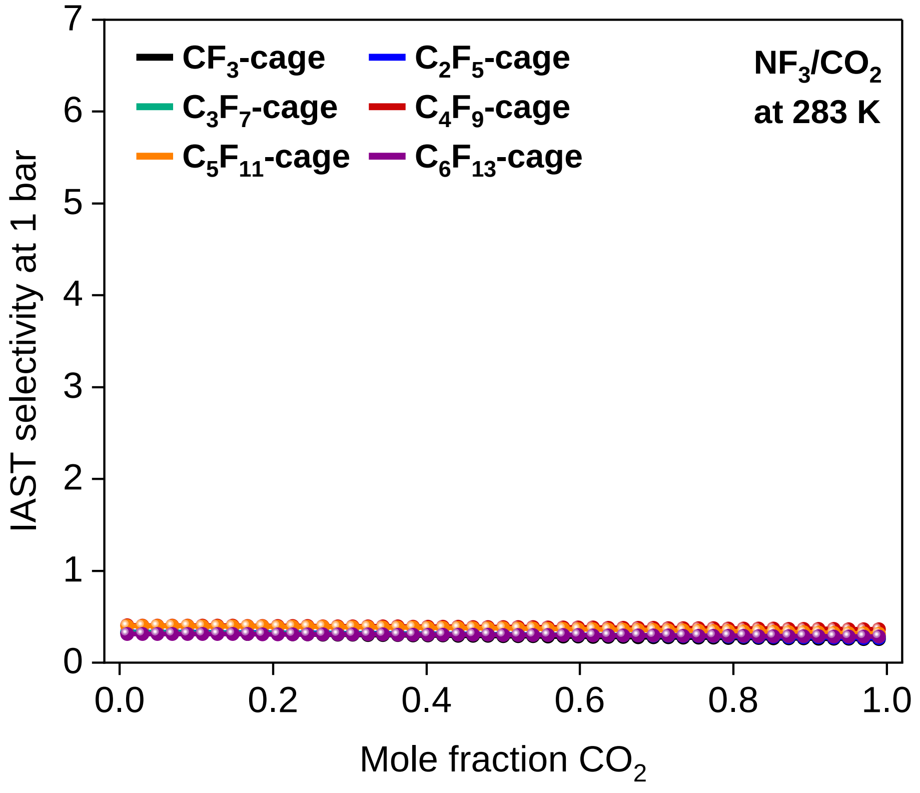

Figure S316. Composition dependent IAST selectivities for SF_6 over carbon dioxide at 283 K and one bar. Black: **CF_3-cage**; blue: **C_2F_5-cage**; green: **C_3F_7-cage**; red: **C_4F_8-cage**; orange: **C_5F_{11}-cage**; purple: **C_6F_{13}-cage**.

IAST Selectivity Curves at 298 K

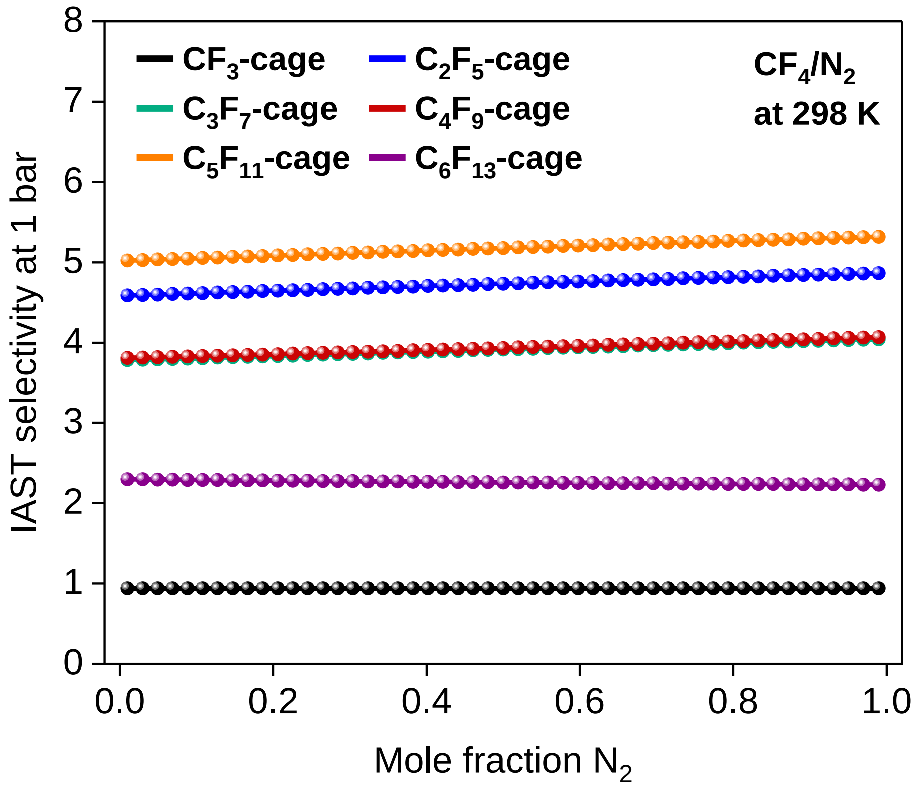

Figure S317. Composition dependent IAST selectivities for CF_4 over nitrogen at 298 K and one bar. Black: **CF_3-cage**; blue: **C_2F_5-cage**; green: **C_3F_7-cage**; red: **C_4F_8-cage**; orange: **C_5F_{11}-cage**; purple: **C_6F_{13}-cage**.

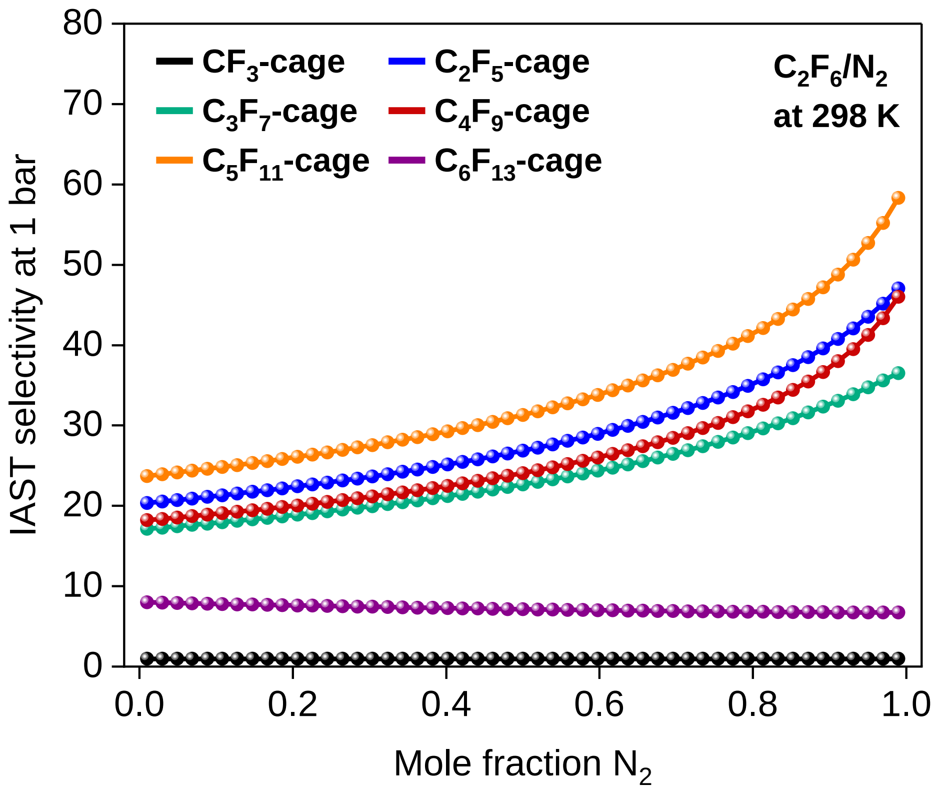

Figure S318. Composition dependent IAST selectivities for C_2F_6 over nitrogen at 298 K and one bar. Black: **CF_3-cage**; blue: **C_2F_5-cage**; green: **C_3F_7-cage**; red: **C_4F_8-cage**; orange: **C_5F_{11}-cage**; purple: **C_6F_{13}-cage**.

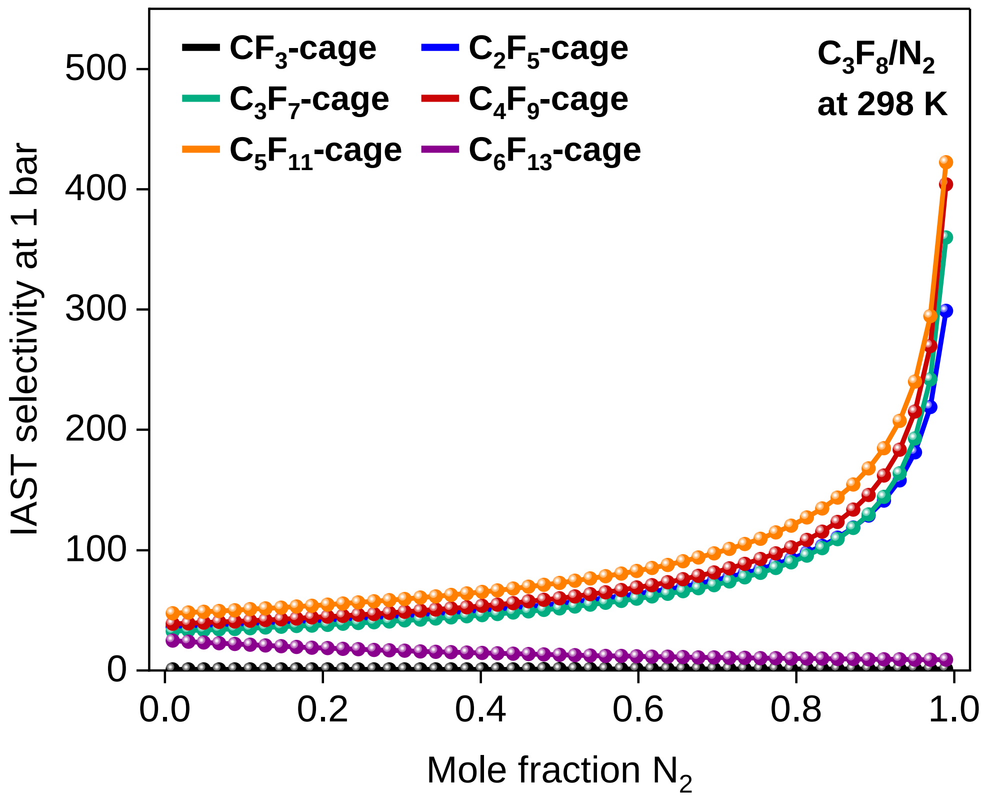

Figure S319. Composition dependent IAST selectivities for C_3F_8 over nitrogen at 298 K and one bar. Black: **CF_3-cage**; blue: **C_2F_5-cage**; green: **C_3F_7-cage**; red: **C_4F_8-cage**; orange: **C_5F_{11}-cage**; purple: **C_6F_{13}-cage**.

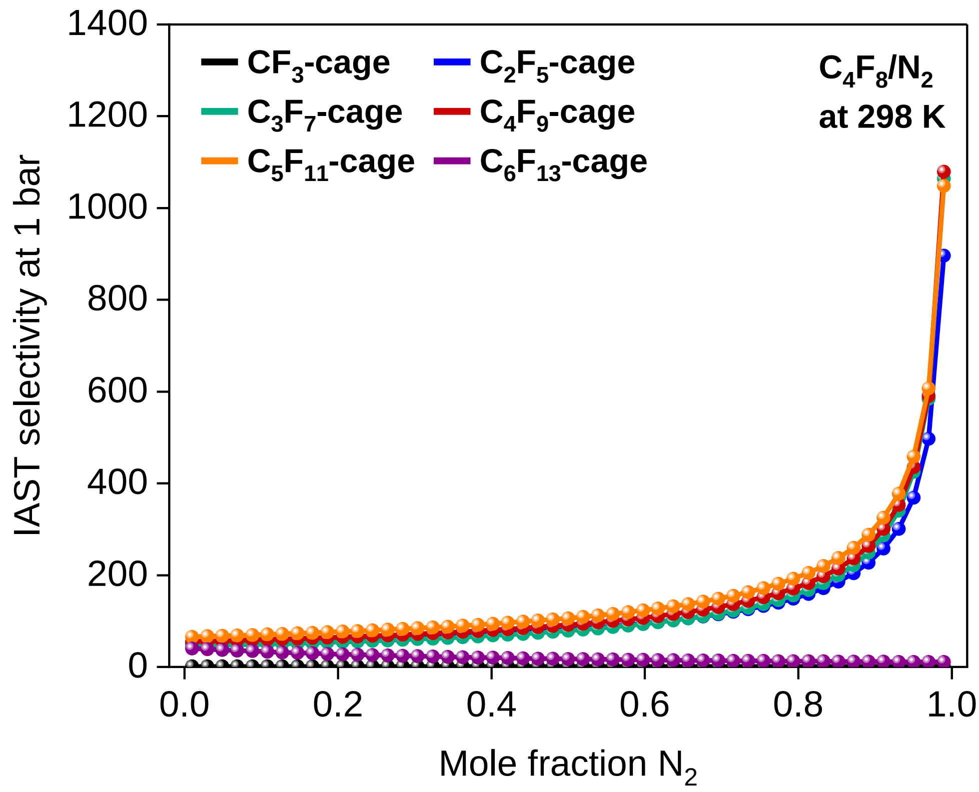

Figure S320. Composition dependent IAST selectivities for C_4F_8 over nitrogen at 298 K and one bar. Black: **CF_3-cage**; blue: **C_2F_5-cage**; green: **C_3F_7-cage**; red: **C_4F_8-cage**; orange: **C_5F_{11}-cage**; purple: **C_6F_{13}-cage**.

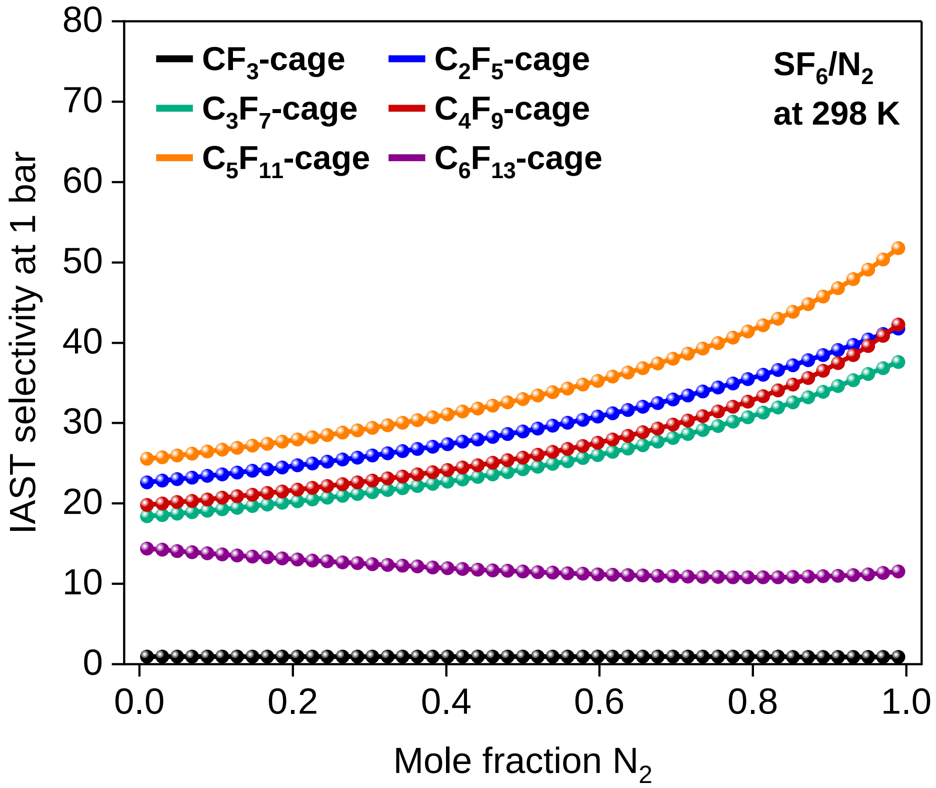

Figure S321. Composition dependent IAST selectivities for SF_6 over nitrogen at 298 K and one bar. Black: **CF_3-cage**; blue: **C_2F_5-cage**; green: **C_3F_7-cage**; red: **C_4F_8-cage**; orange: **C_5F_{11}-cage**; purple: **C_6F_{13}-cage**.

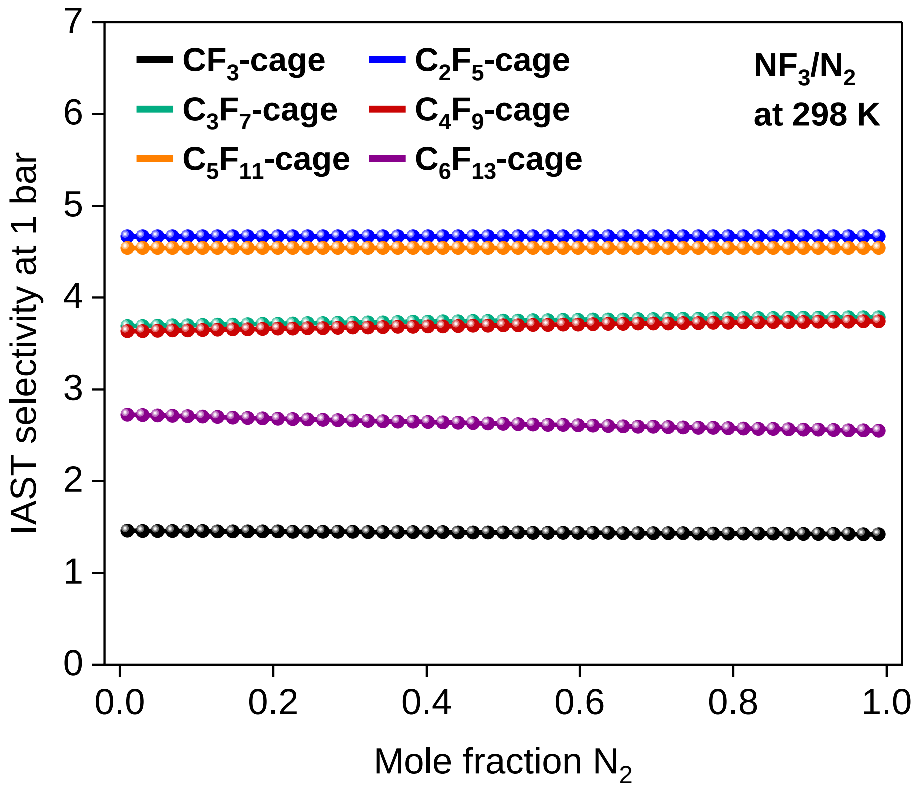

Figure S322. Composition dependent IAST selectivities for NF_3 over nitrogen at 298 K and one bar. Black: **CF_3-cage**; blue: **C_2F_5-cage**; green: **C_3F_7-cage**; red: **C_4F_8-cage**; orange: **C_5F_{11}-cage**; purple: **C_6F_{13}-cage**.

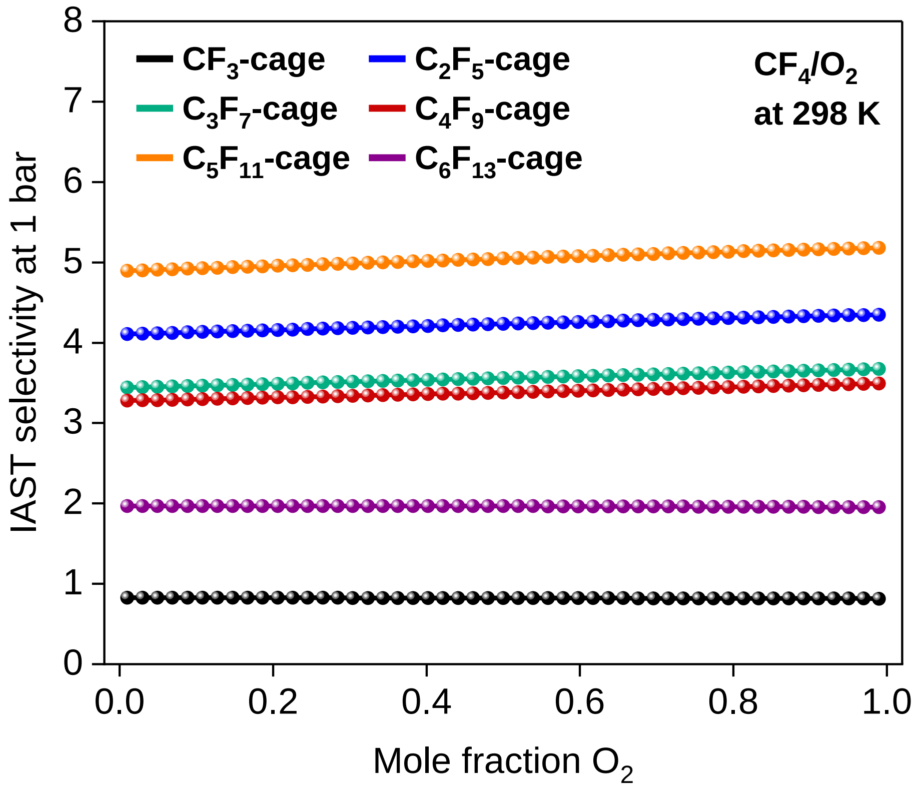

Figure S323. Composition dependent IAST selectivities for CF_4 over oxygen at 298 K and one bar. Black: **CF_3-cage**; blue: **C_2F_5-cage**; green: **C_3F_7-cage**; red: **C_4F_8-cage**; orange: **C_5F_{11}-cage**; purple: **C_6F_{13}-cage**.

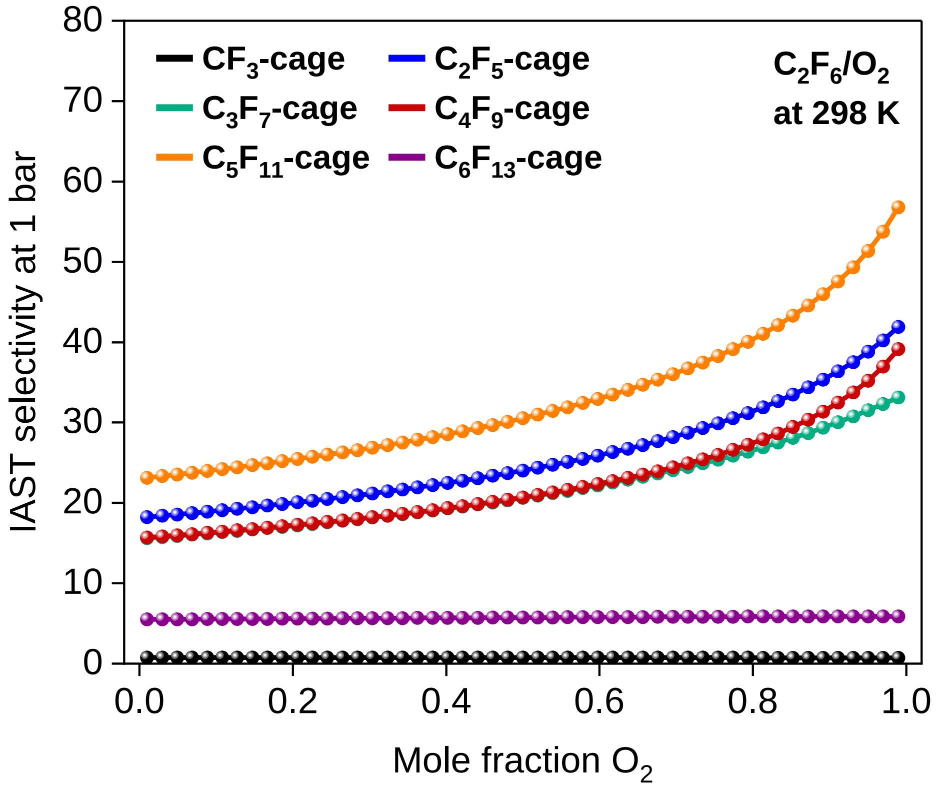

Figure S324. Composition dependent IAST selectivities for C_2F_6 over oxygen at 298 K and one bar. Black: **CF_3-cage**; blue: **C_2F_5-cage**; green: **C_3F_7-cage**; red: **C_4F_8-cage**; orange: **C_5F_{11}-cage**; purple: **C_6F_{13}-cage**.

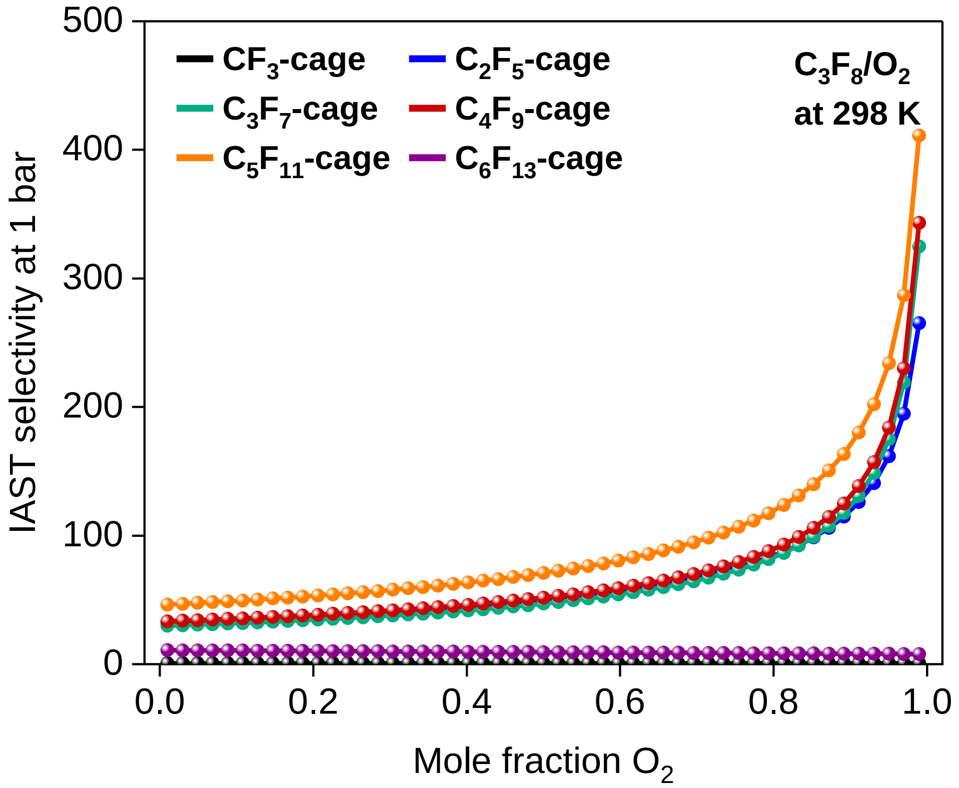

Figure S325. Composition dependent IAST selectivities for C_3F_8 over oxygen at 298 K and one bar. Black: **CF_3-cage**; blue: **C_2F_5-cage**; green: **C_3F_7-cage**; red: **C_4F_8-cage**; orange: **C_5F_{11}-cage**; purple: **C_6F_{13}-cage**.

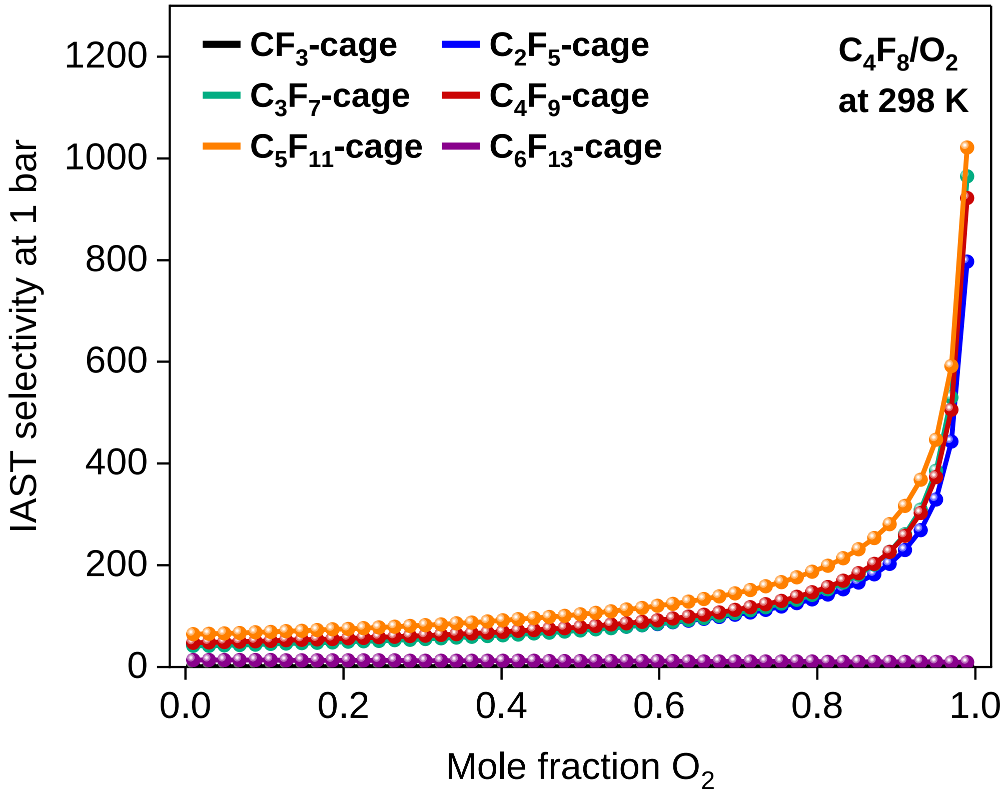

Figure S326. Composition dependent IAST selectivities for C_4F_8 over oxygen at 298 K and one bar. Black: **CF_3-cage**; blue: **C_2F_5-cage**; green: **C_3F_7-cage**; red: **C_4F_8-cage**; orange: **C_5F_{11}-cage**; purple: **C_6F_{13}-cage**.

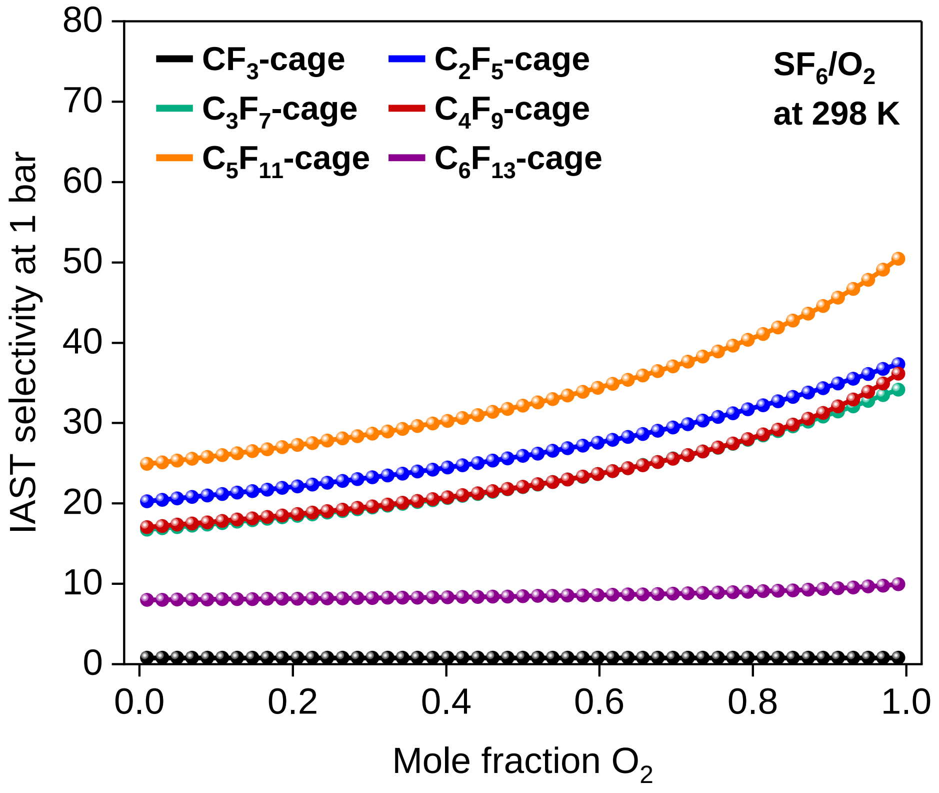

Figure S327. Composition dependent IAST selectivities for SF_6 over oxygen at 298 K and one bar. Black: **CF_3-cage**; blue: **C_2F_5-cage**; green: **C_3F_7-cage**; red: **C_4F_8-cage**; orange: **C_5F_{11}-cage**; purple: **C_6F_{13}-cage**.

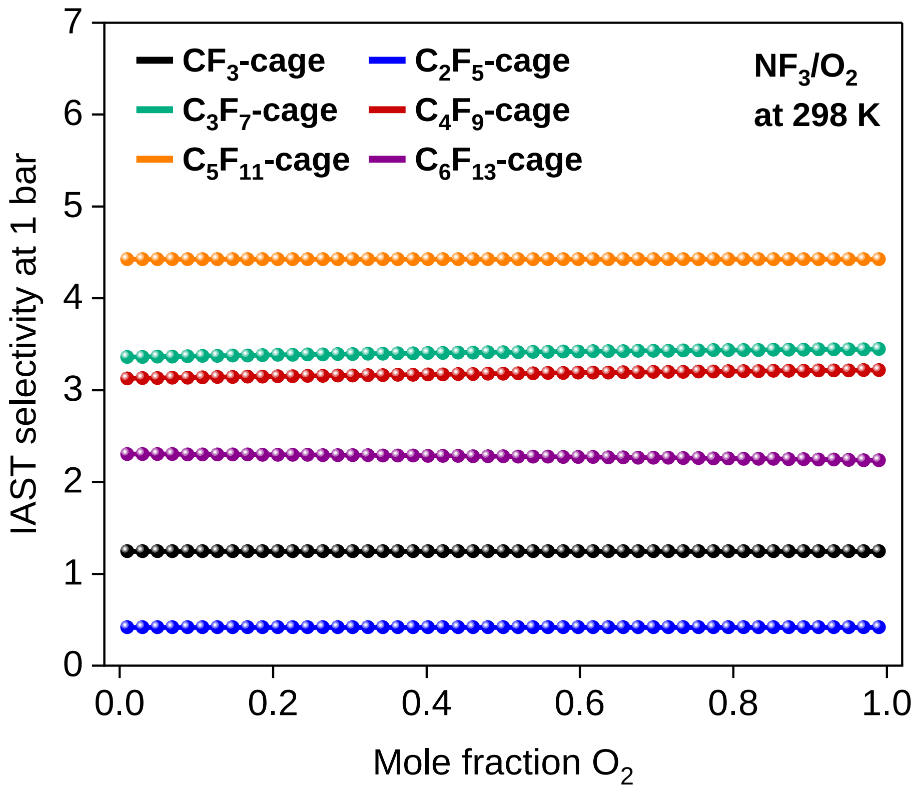

Figure S328. Composition dependent IAST selectivities for NF_3 over oxygen at 298 K and one bar. Black: **CF_3-cage**; blue: **C_2F_5-cage**; green: **C_3F_7-cage**; red: **C_4F_8-cage**; orange: **C_5F_{11}-cage**; purple: **C_6F_{13}-cage**.

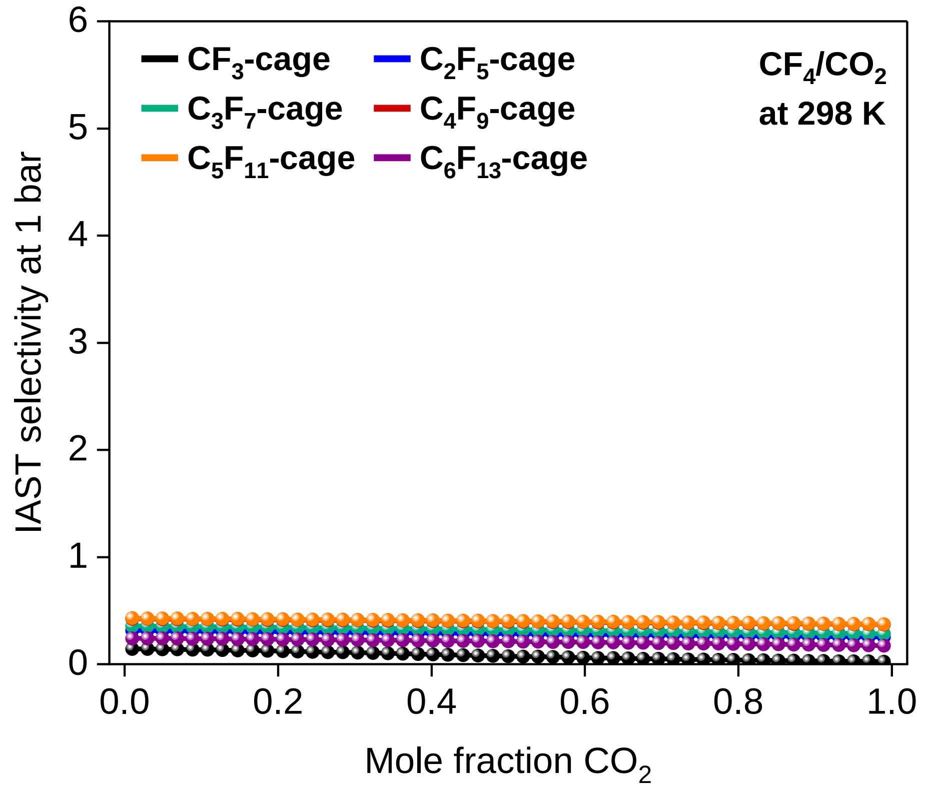

Figure S329. Composition dependent IAST selectivities for CF_4 over carbon dioxide at 298 K and one bar. Black: **CF_3-cage**; blue: **C_2F_5-cage**; green: **C_3F_7-cage**; red: **C_4F_8-cage**; orange: **C_5F_{11}-cage**; purple: **C_6F_{13}-cage**.

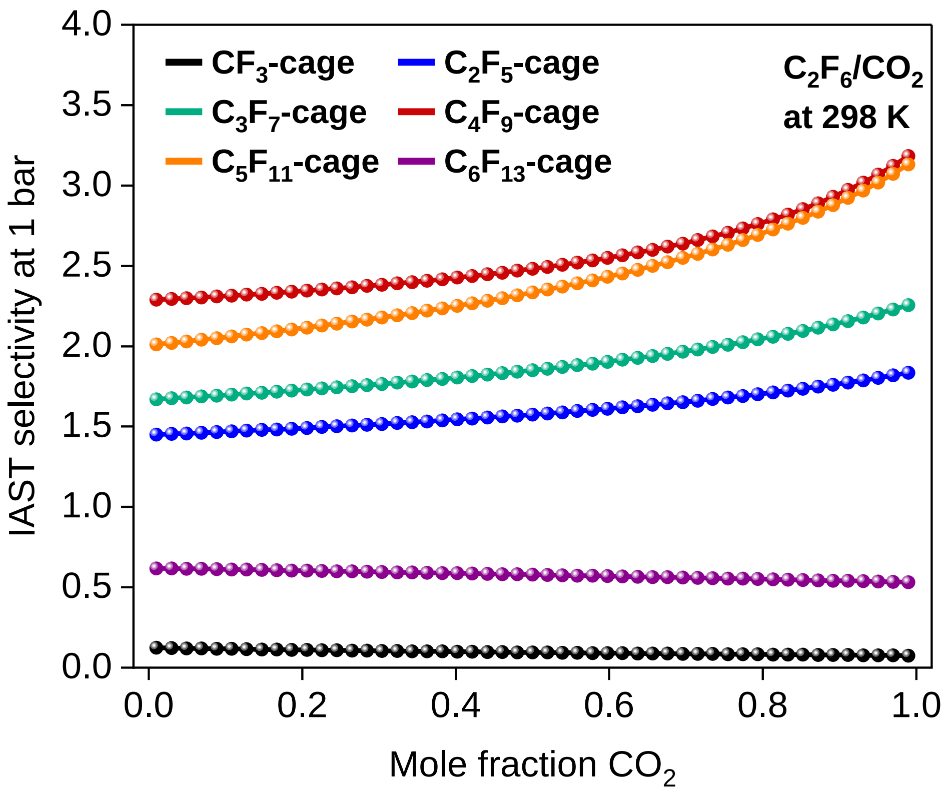

Figure S330. Composition dependent IAST selectivities for C_2F_6 over carbon dioxide at 298 K and one bar. Black: **CF_3-cage**; blue: **C_2F_5-cage**; green: **C_3F_7-cage**; red: **C_4F_8-cage**; orange: **C_5F_{11}-cage**; purple: **C_6F_{13}-cage**.

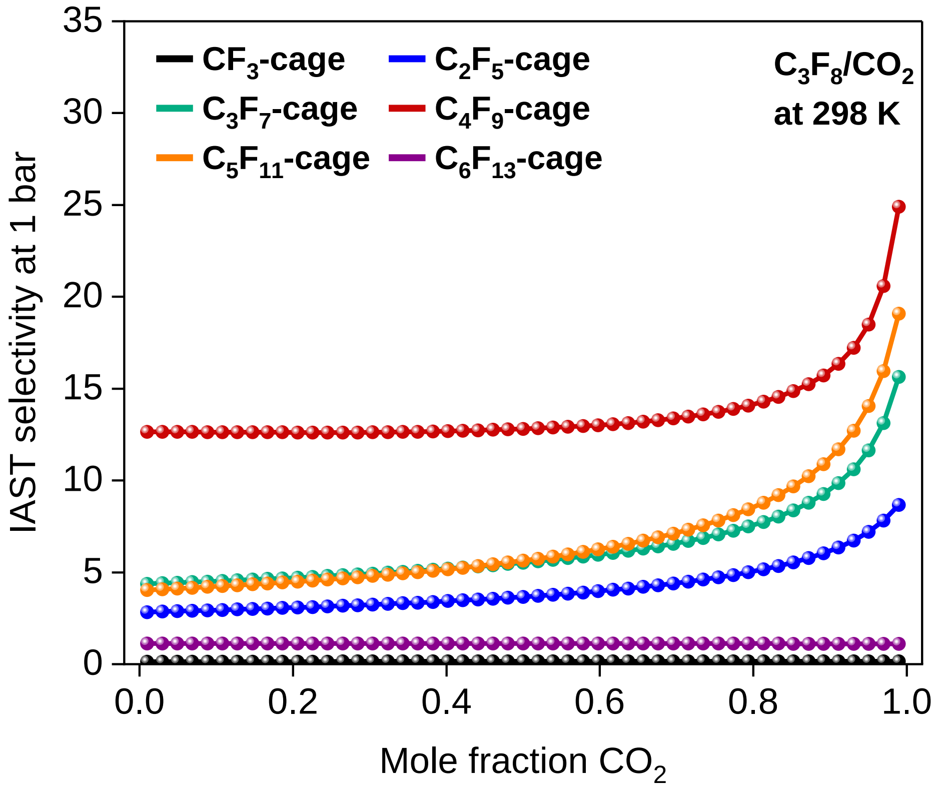

Figure S331. Composition dependent IAST selectivities for C_3F_8 over carbon dioxide at 298 K and one bar. Black: **CF_3-cage**; blue: **C_2F_5-cage**; green: **C_3F_7-cage**; red: **C_4F_8-cage**; orange: **C_5F_{11}-cage**; purple: **C_6F_{13}-cage**.

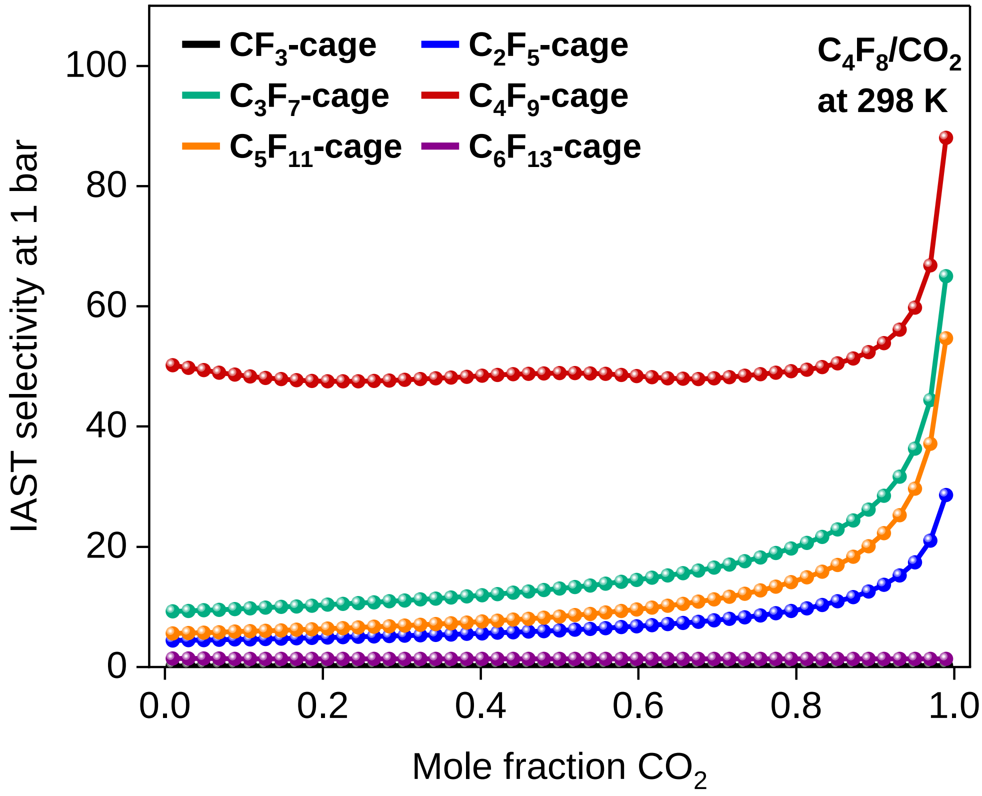

Figure S332. Composition dependent IAST selectivities for C_4F_8 over carbon dioxide at 298 K and one bar. Black: **CF_3-cage**; blue: **C_2F_5-cage**; green: **C_3F_7-cage**; red: **C_4F_8-cage**; orange: **C_5F_{11}-cage**; purple: **C_6F_{13}-cage**.

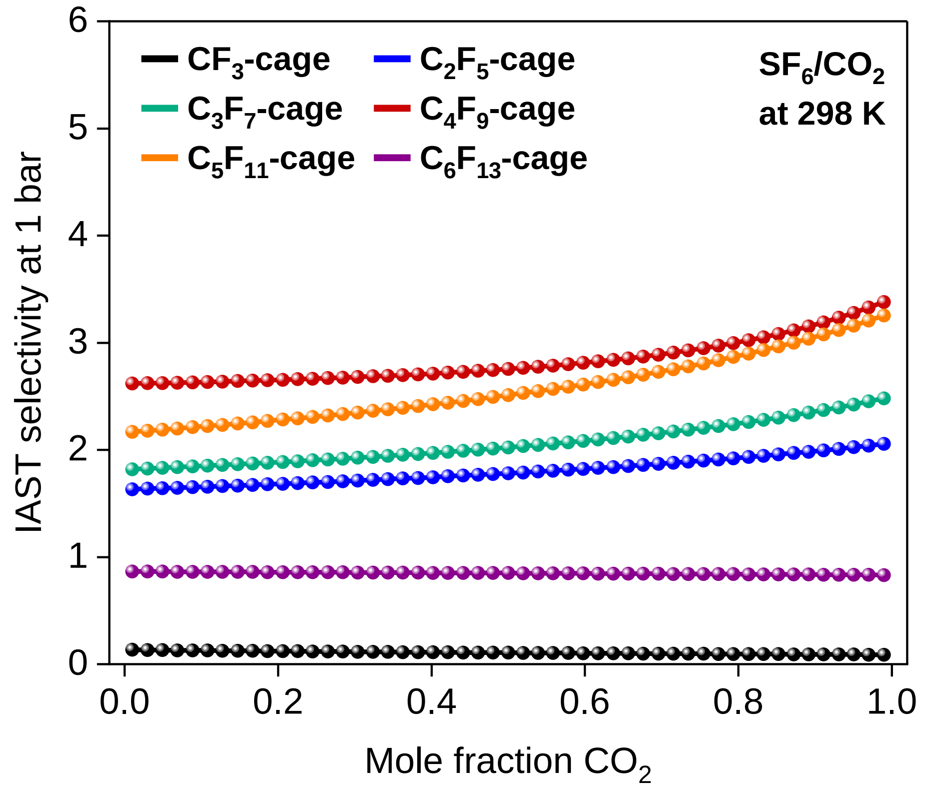

Figure S333. Composition dependent IAST selectivities for SF_6 over carbon dioxide at 298 K and one bar. Black: **CF_3-cage**; blue: **C_2F_5-cage**; green: **C_3F_7-cage**; red: **C_4F_8-cage**; orange: **C_5F_{11}-cage**; purple: **C_6F_{13}-cage**.

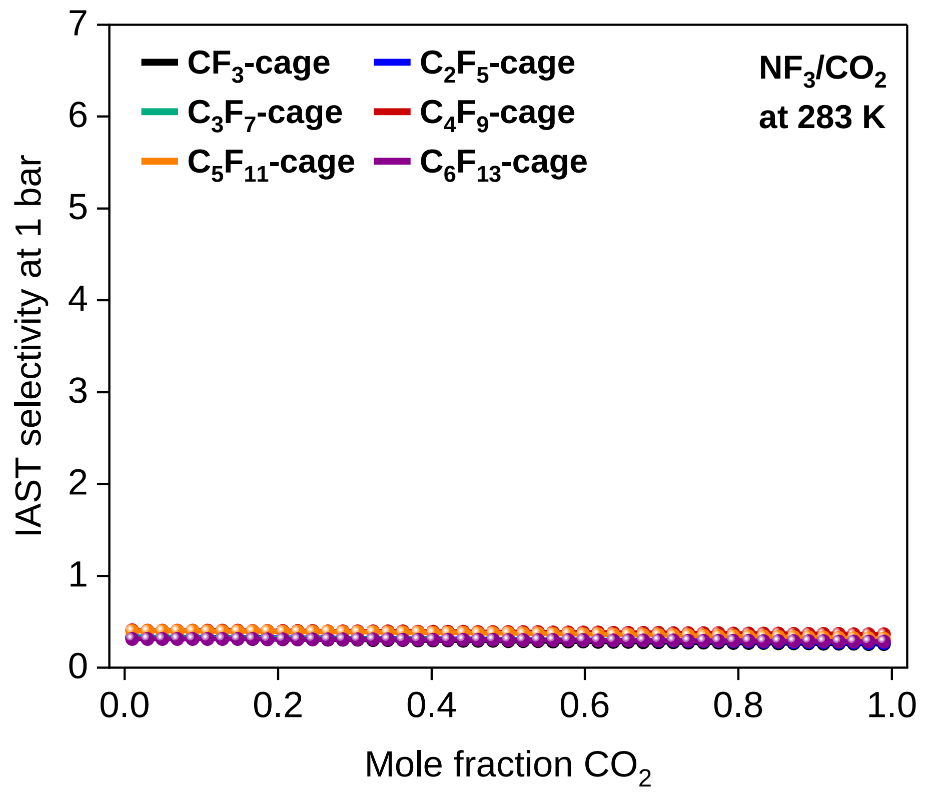

Figure S334. Composition dependent IAST selectivities for NF_3 over carbon dioxide at 298 K and one bar. Black: **CF_3-cage**; blue: **C_2F_5-cage**; green: **C_3F_7-cage**; red: **C_4F_8-cage**; orange: **C_5F_{11}-cage**; purple: **C_6F_{13}-cage**.

IAST Selectivity Curves at 313 K

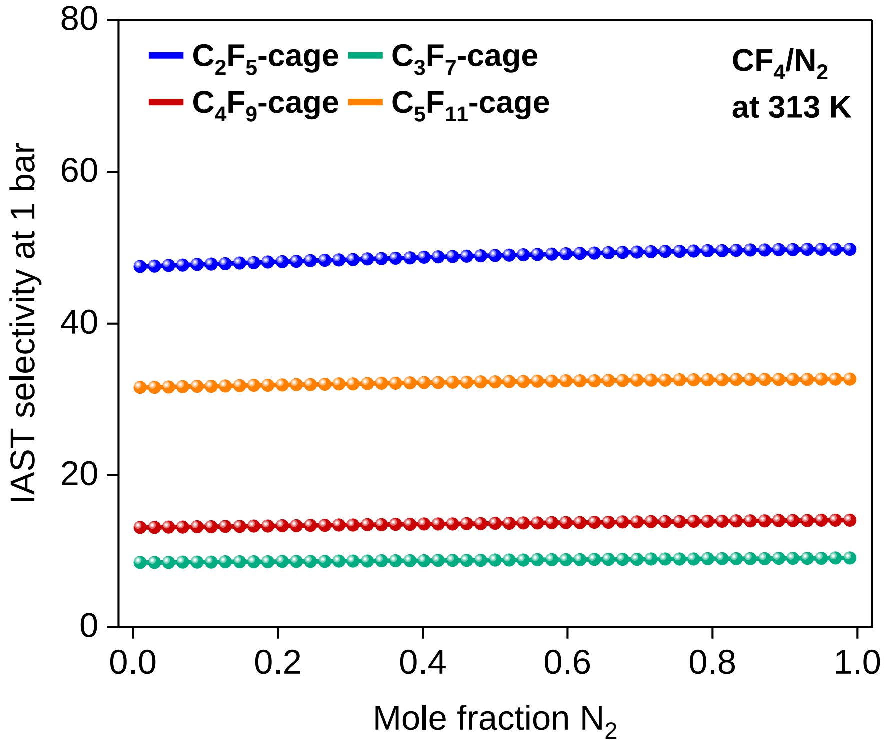

Figure S335. Composition dependent IAST selectivities for CF_4 over nitrogen at 313 K and one bar. Black: **CF_3-cage**; blue: **C_2F_5-cage**; green: **C_3F_7-cage**; red: **C_4F_8-cage**; orange: **C_5F_{11}-cage**; purple: **C_6F_{13}-cage**.

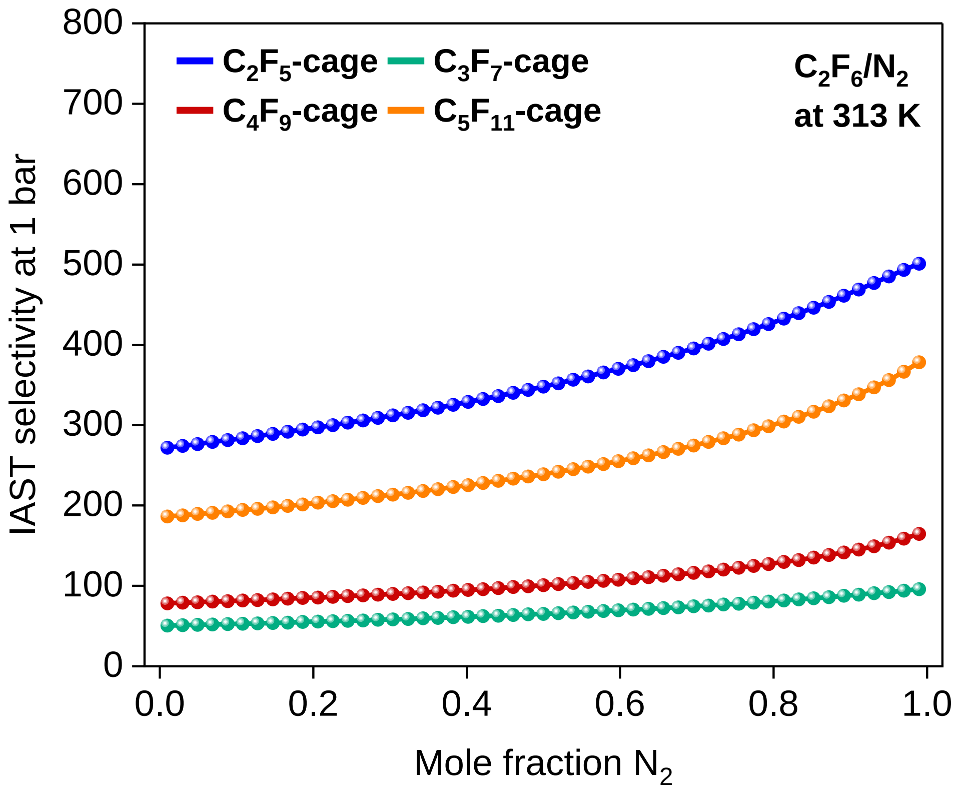

Figure S336. Composition dependent IAST selectivities for C_2F_6 over nitrogen at 313 K and one bar. Black: **CF_3-cage**; blue: **C_2F_5-cage**; green: **C_3F_7-cage**; red: **C_4F_8-cage**; orange: **C_5F_{11}-cage**; purple: **C_6F_{13}-cage**.

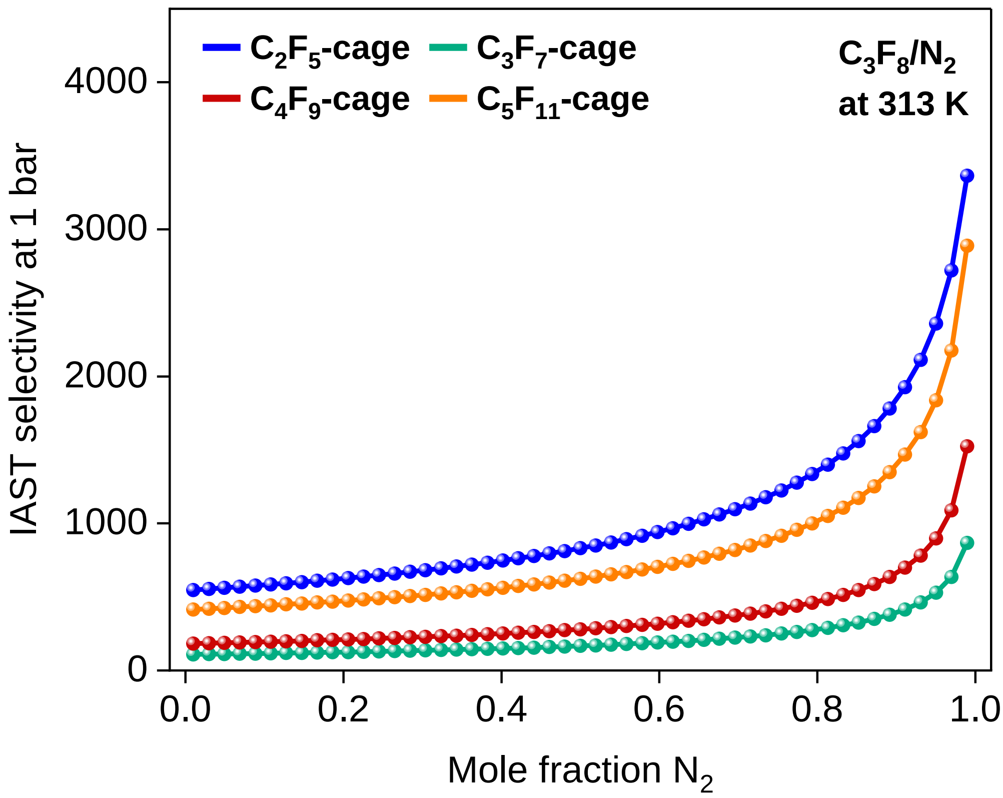

Figure S337. Composition dependent IAST selectivities for C_3F_8 over nitrogen at 313 K and one bar. Black: **CF_3-cage**; blue: **C_2F_5-cage**; green: **C_3F_7-cage**; red: **C_4F_8-cage**; orange: **C_5F_{11}-cage**; purple: **C_6F_{13}-cage**.

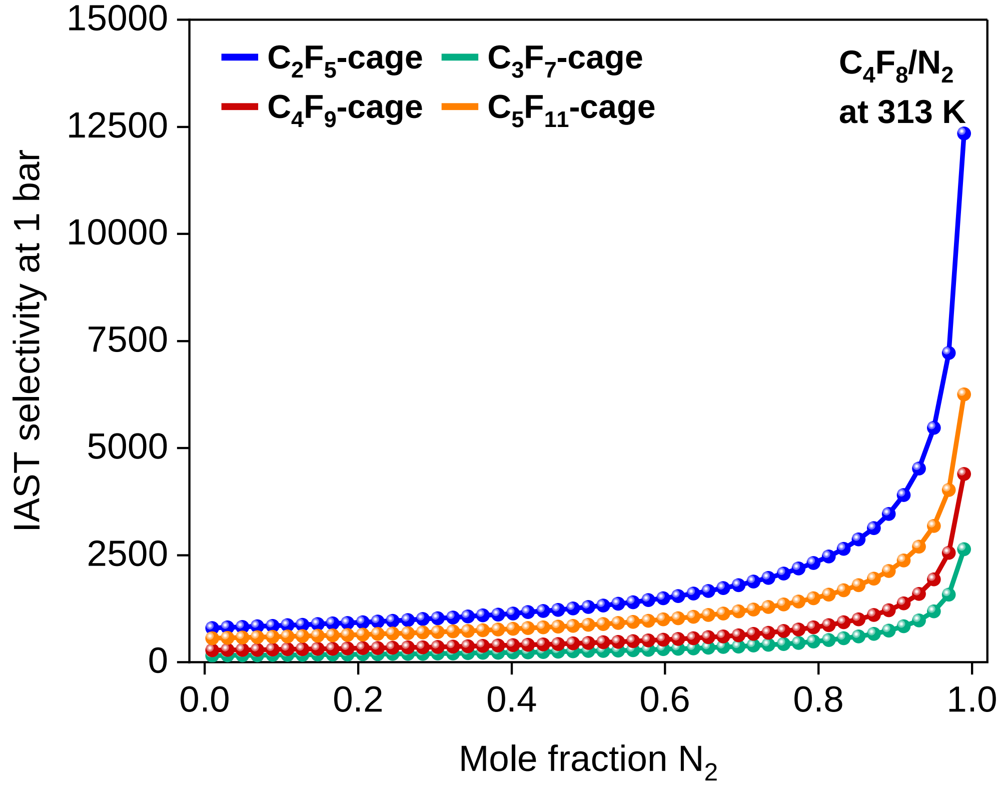

Figure S338. Composition dependent IAST selectivities for C_4F_8 over nitrogen at 313 K and one bar. Black: **CF_3-cage**; blue: **C_2F_5-cage**; green: **C_3F_7-cage**; red: **C_4F_8-cage**; orange: **C_5F_{11}-cage**; purple: **C_6F_{13}-cage**.

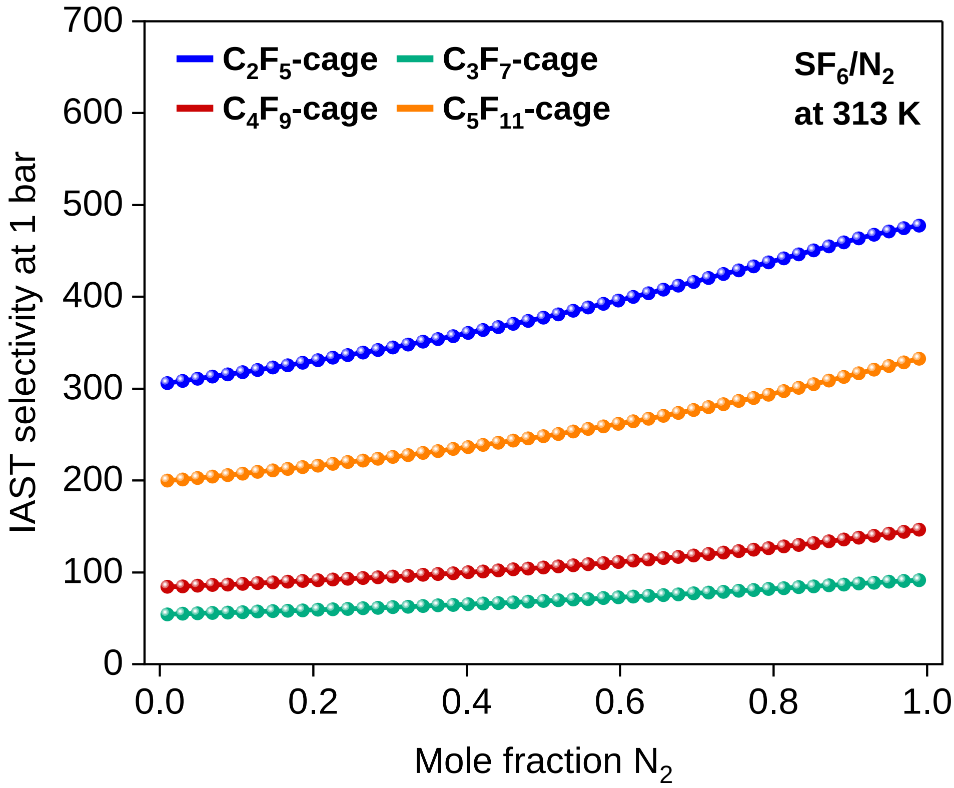

Figure S339. Composition dependent IAST selectivities for SF_6 over nitrogen at 313 K and one bar. Black: **CF_3-cage**; blue: **C_2F_5-cage**; green: **C_3F_7-cage**; red: **C_4F_8-cage**; orange: **C_5F_{11}-cage**; purple: **C_6F_{13}-cage**.

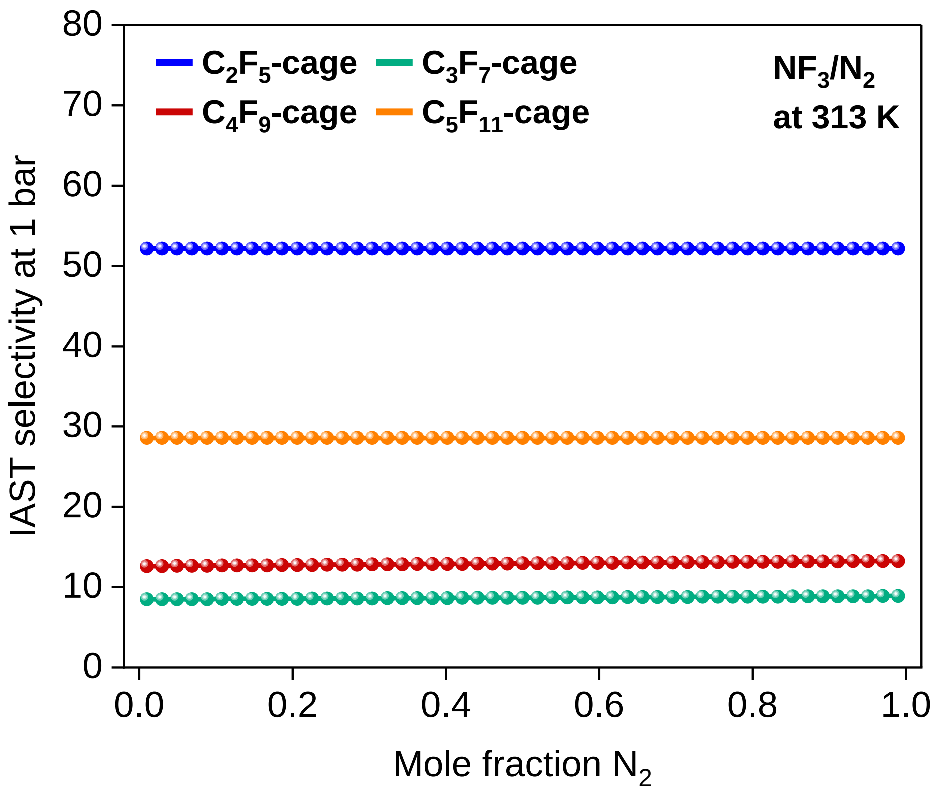

Figure S340. Composition dependent IAST selectivities for NF_3 over nitrogen at 313 K and one bar. Black: **CF_3-cage**; blue: **C_2F_5-cage**; green: **C_3F_7-cage**; red: **C_4F_8-cage**; orange: **C_5F_{11}-cage**; purple: **C_6F_{13}-cage**.

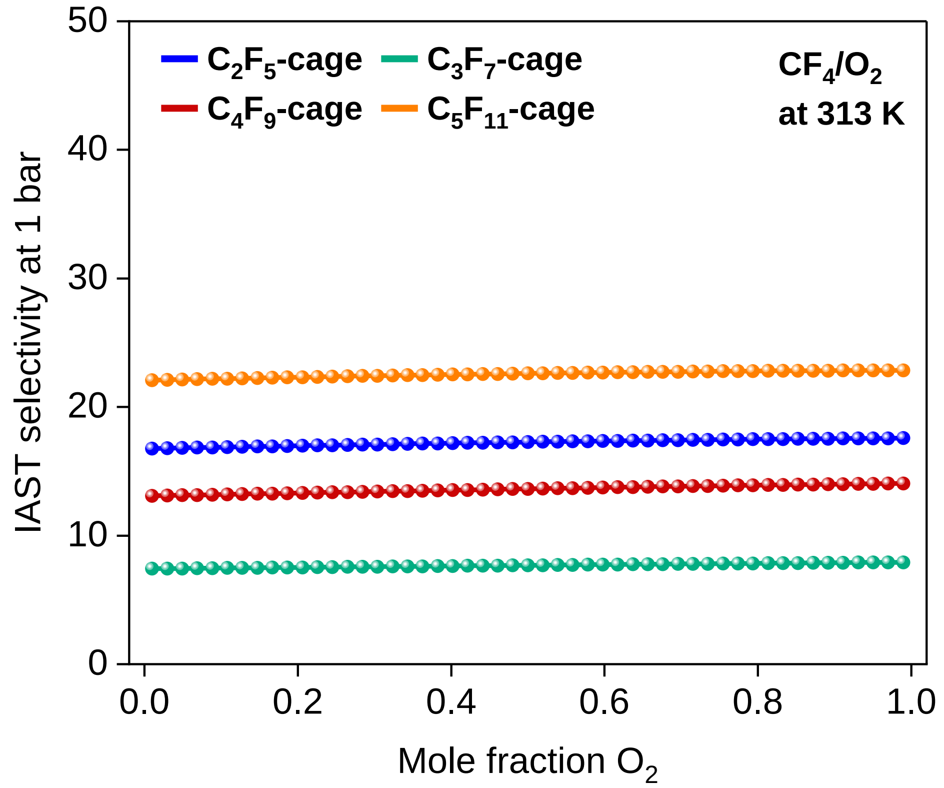

Figure S341. Composition dependent IAST selectivities for CF_4 over oxygen at 313 K and one bar. Black: **CF_3-cage**; blue: **C_2F_5-cage**; green: **C_3F_7-cage**; red: **C_4F_8-cage**; orange: **C_5F_{11}-cage**; purple: **C_6F_{13}-cage**.

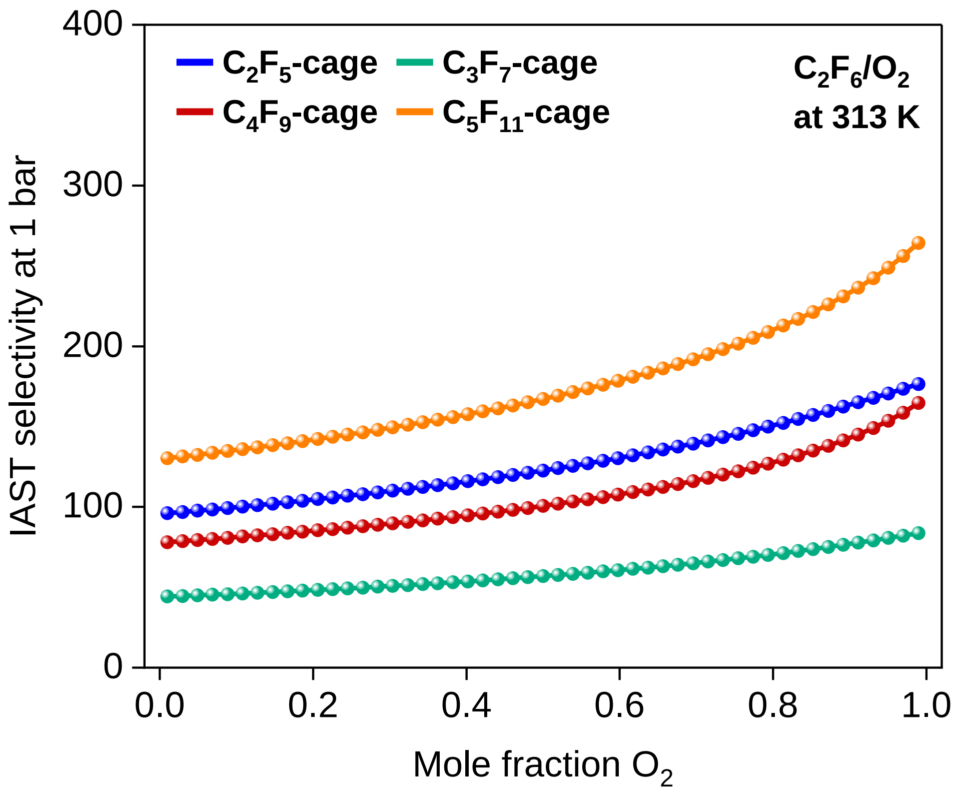

Figure S342. Composition dependent IAST selectivities for C_2F_6 over oxygen at 313 K and one bar. Black: **CF_3-cage**; blue: **C_2F_5-cage**; green: **C_3F_7-cage**; red: **C_4F_8-cage**; orange: **C_5F_{11}-cage**; purple: **C_6F_{13}-cage**.

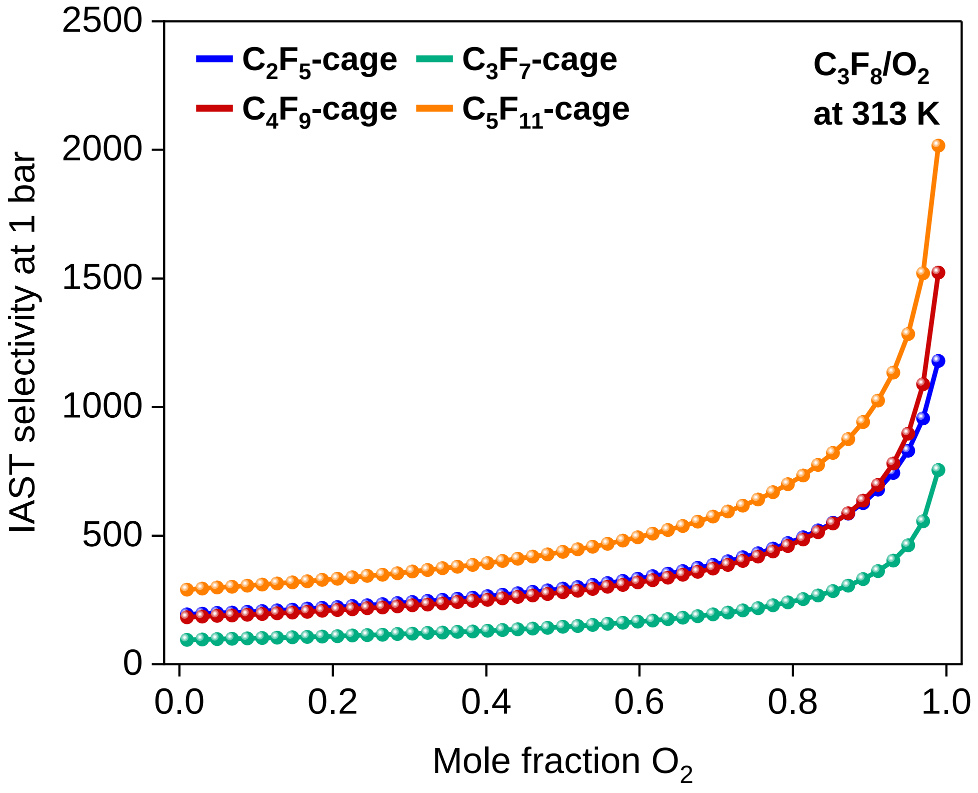

Figure S343. Composition dependent IAST selectivities for C_3F_8 over oxygen at 313 K and one bar. Black: **CF_3-cage**; blue: **C_2F_5-cage**; green: **C_3F_7-cage**; red: **C_4F_8-cage**; orange: **C_5F_{11}-cage**; purple: **C_6F_{13}-cage**.

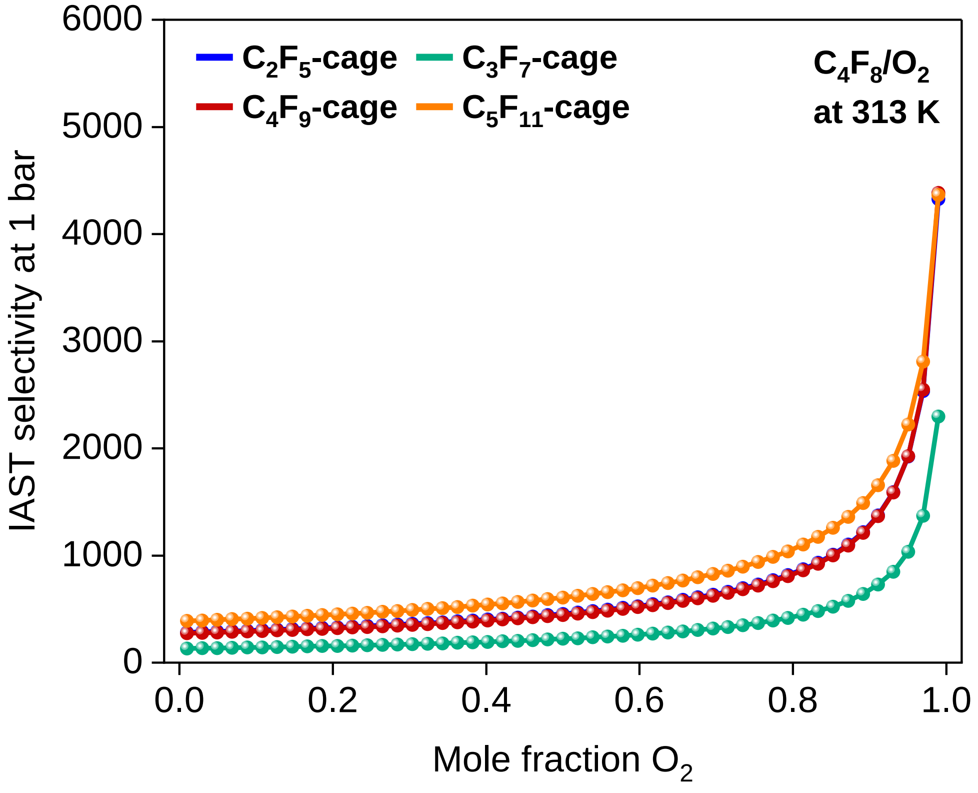

Figure S344. Composition dependent IAST selectivities for C_4F_8 over oxygen at 313 K and one bar. Black: **CF_3-cage**; blue: **C_2F_5-cage**; green: **C_3F_7-cage**; red: **C_4F_8-cage**; orange: **C_5F_{11}-cage**; purple: **C_6F_{13}-cage**.

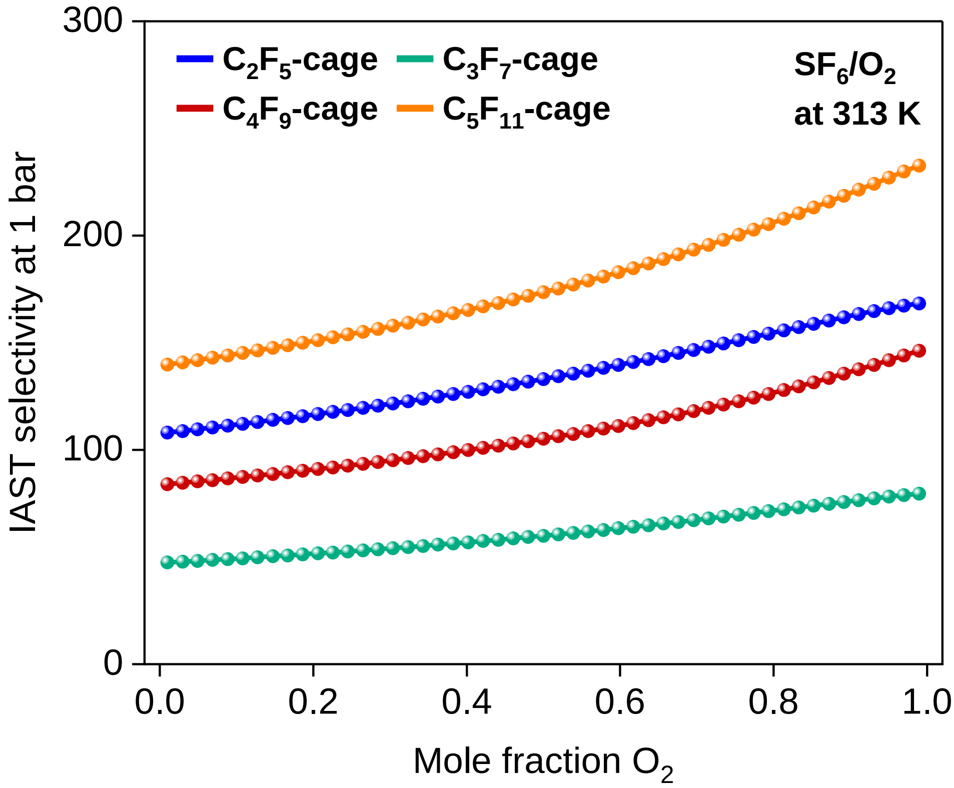

Figure S345. Composition dependent IAST selectivities for SF_6 over oxygen at 313 K and one bar. Black: **CF_3-cage**; blue: **C_2F_5-cage**; green: **C_3F_7-cage**; red: **C_4F_8-cage**; orange: **C_5F_{11}-cage**; purple: **C_6F_{13}-cage**.

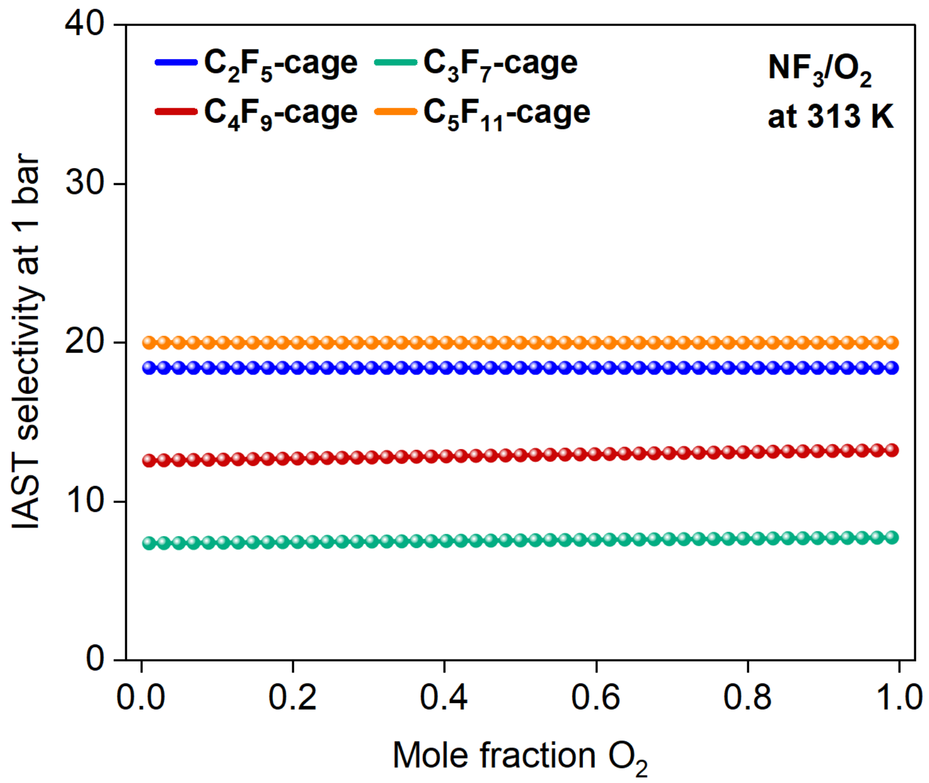

Figure S346. Composition dependent IAST selectivities for NF_3 over oxygen at 313 K and one bar. Black: **CF_3-cage**; blue: **C_2F_5-cage**; green: **C_3F_7-cage**; red: **C_4F_8-cage**; orange: **C_5F_{11}-cage**; purple: **C_6F_{13}-cage**.

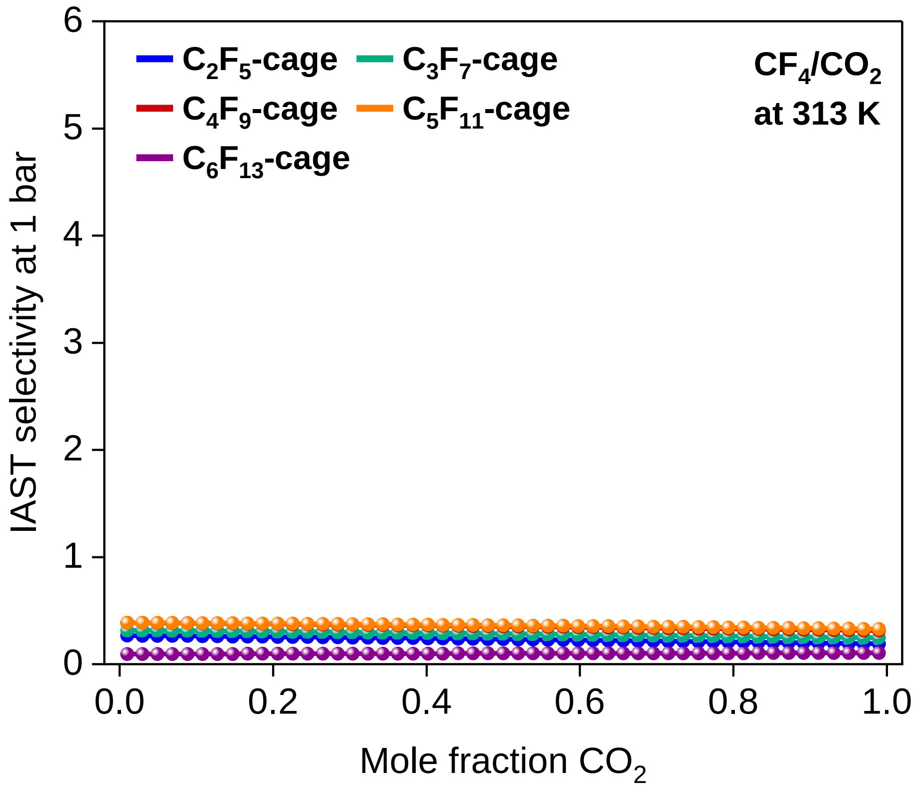

Figure S347. Composition dependent IAST selectivities for CF_4 over carbon dioxide at 313 K and one bar. Black: **CF_3-cage**; blue: **C_2F_5-cage**; green: **C_3F_7-cage**; red: **C_4F_8-cage**; orange: **C_5F_{11}-cage**; purple: **C_6F_{13}-cage**.

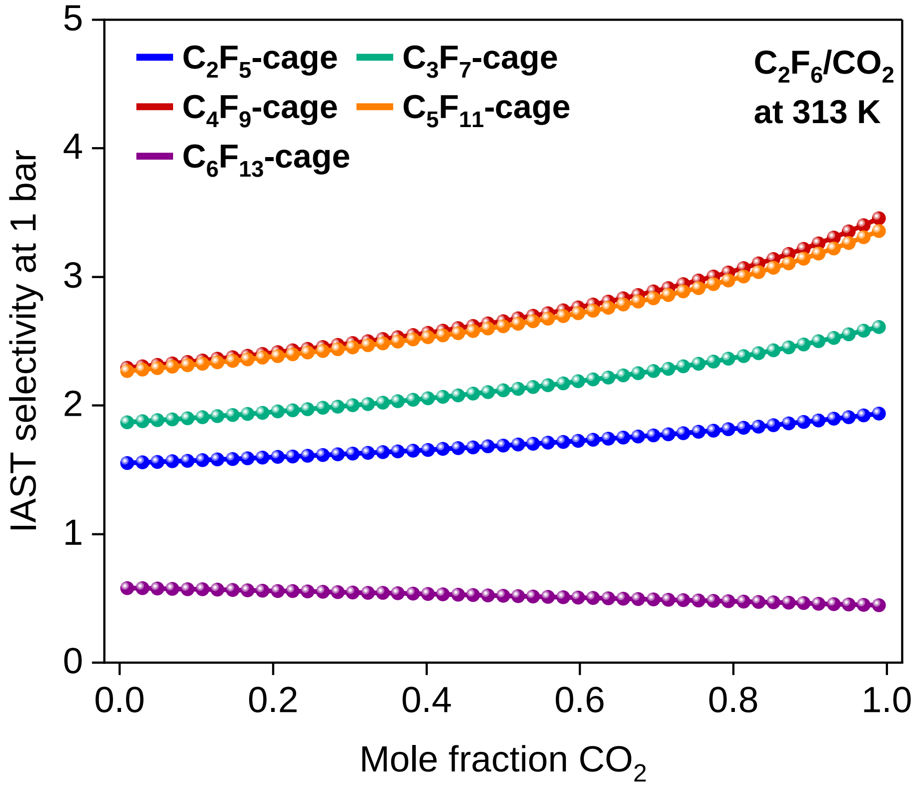

Figure S348. Composition dependent IAST selectivities for C_2F_6 over carbon dioxide at 313 K and one bar. Black: **CF_3-cage**; blue: **C_2F_5-cage**; green: **C_3F_7-cage**; red: **C_4F_8-cage**; orange: **C_5F_{11}-cage**; purple: **C_6F_{13}-cage**.

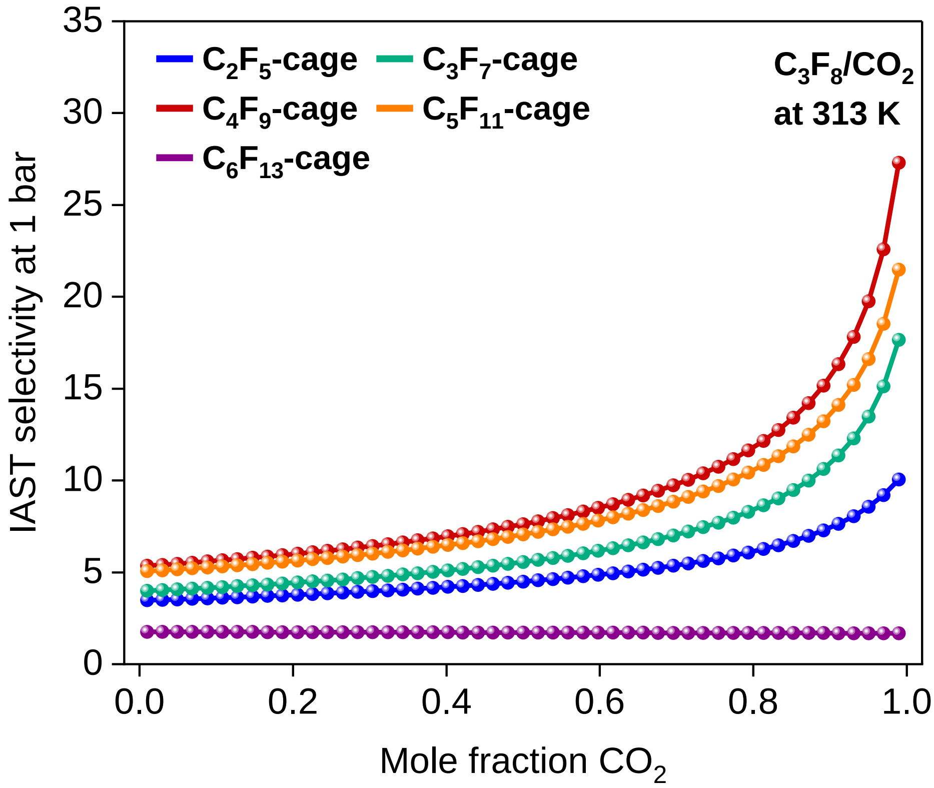

Figure S349. Composition dependent IAST selectivities for C_3F_8 over carbon dioxide at 313 K and one bar. Black: **CF_3-cage**; blue: **C_2F_5-cage**; green: **C_3F_7-cage**; red: **C_4F_8-cage**; orange: **C_5F_{11}-cage**; purple: **C_6F_{13}-cage**.

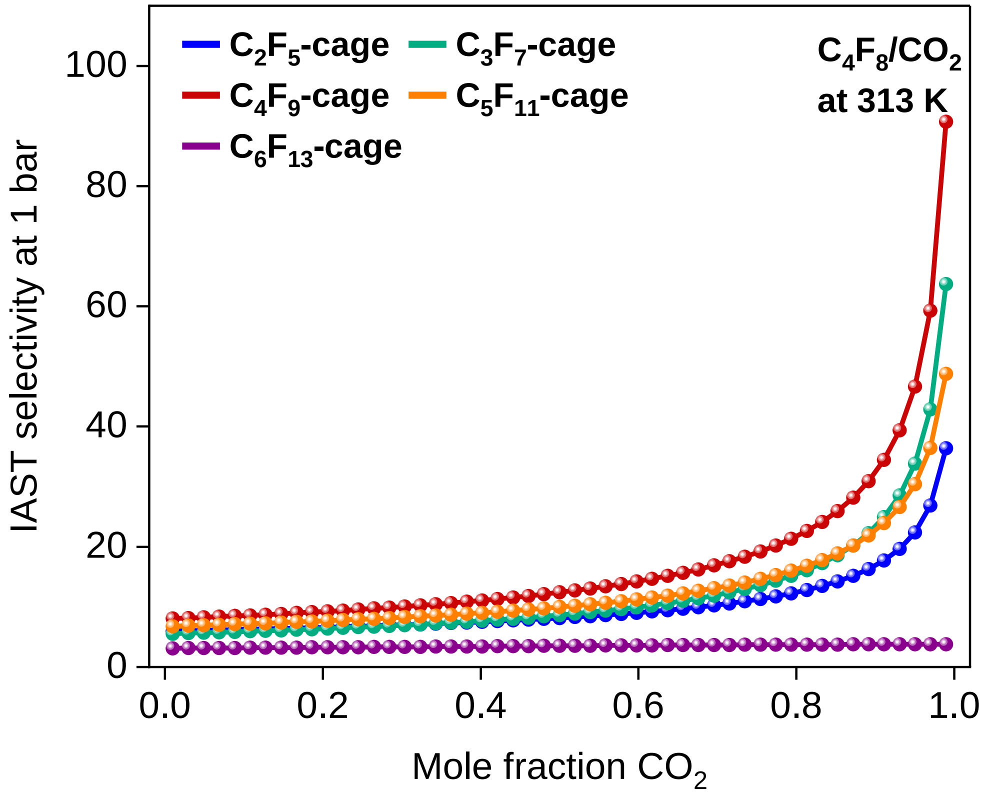

Figure S350. Composition dependent IAST selectivities for C_4F_8 over carbon dioxide at 313 K and one bar. Black: **CF_3-cage**; blue: **C_2F_5-cage**; green: **C_3F_7-cage**; red: **C_4F_8-cage**; orange: **C_5F_{11}-cage**; purple: **C_6F_{13}-cage**.

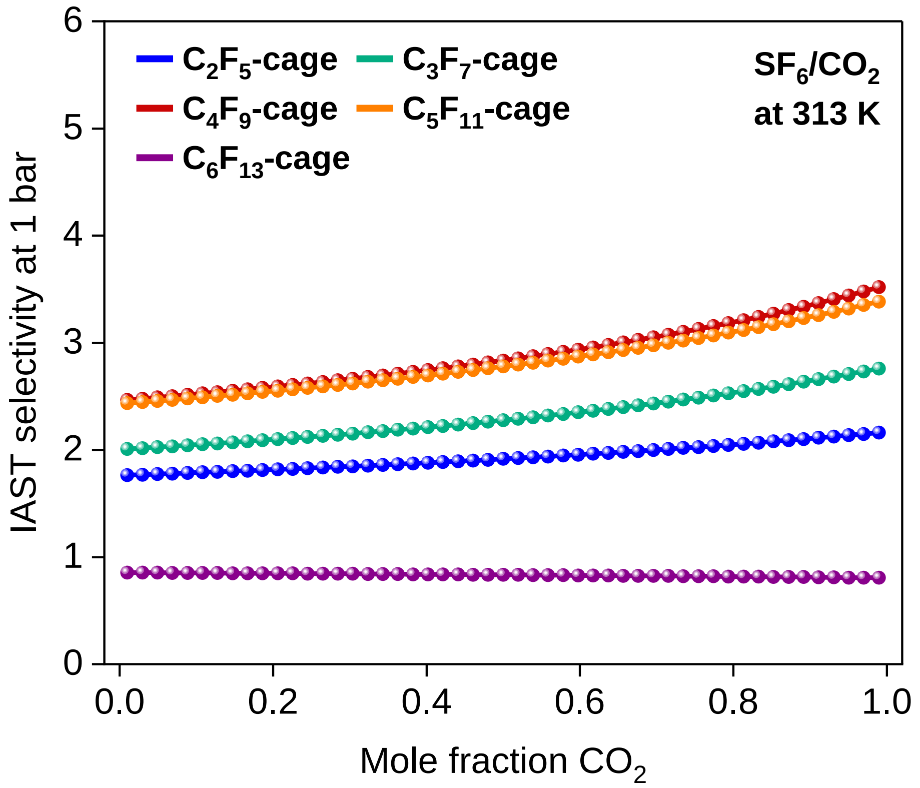

Figure S351. Composition dependent IAST selectivities for SF_6 over carbon dioxide at 313 K and one bar. Black: **CF_3-cage**; blue: **C_2F_5-cage**; green: **C_3F_7-cage**; red: **C_4F_8-cage**; orange: **C_5F_{11}-cage**; purple: **C_6F_{13}-cage**.

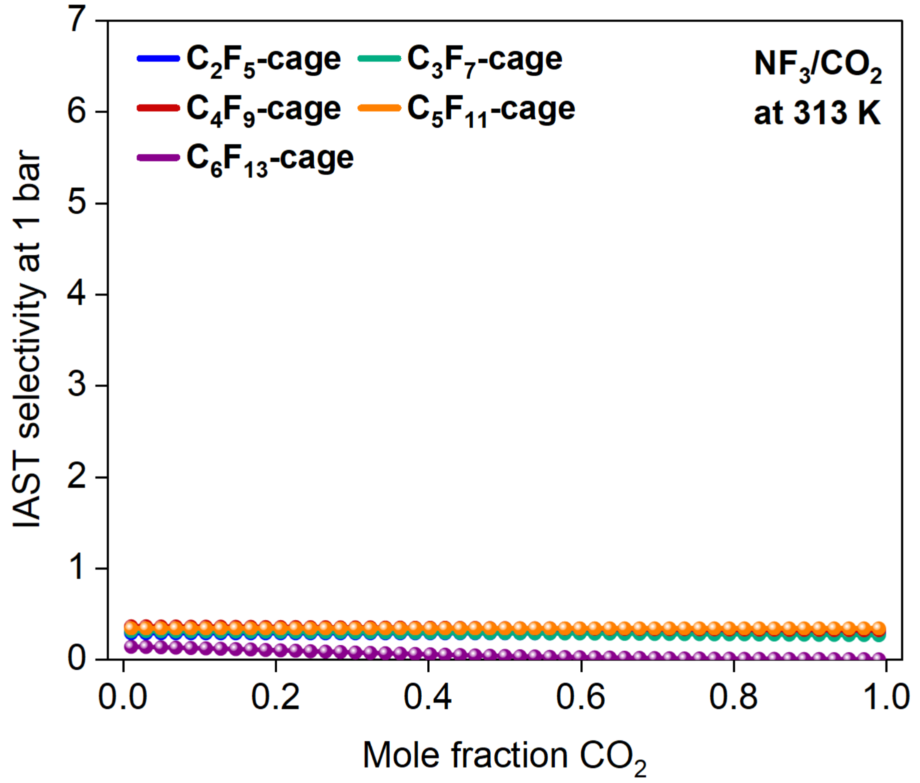

Figure S352. Composition dependent IAST selectivities for NF_3 over carbon dioxide at 313 K and one bar. Black: **CF_3-cage**; blue: **C_2F_5-cage**; green: **C_3F_7-cage**; red: **C_4F_8-cage**; orange: **C_5F_{11}-cage**; purple: **C_6F_{13}-cage**.

Isosteric Enthalpies of Adsorption

For the calculation of the isosteric enthalpies of adsorption[S12] of the corresponding gas the temperature independent virial coefficients a_i and b_j were calculated by fitting the following virial-type expression to the experimental data at 273 K and 283 K:

$$\ln p = \ln n + \left(\frac{1}{T}\right)\sum_{i=0}^{m} a_i\, q^i + \sum_{j=0}^{m} b_j\, q^j$$

with: p: pressure (kpa)

n: amount of gas adsorbed (mmol·g^{-1})

T: temperature (K)

$a_i + b_j$: virial coefficients

$n + m$: number of coefficients to describe the isotherms adequately

For n = 7 and m =1 the overall fit and the average value of the squared deviations from the experimental values was minimized. The values of the virial coefficients a_0 to a_m were then used to calculate the isosteric enthalpy of adsorption using the following expression:

$$\Delta H_{\mathrm{ads}}(\mathrm{n}) = \mathrm{R}\sum_{\mathrm{i}=0}^{\mathrm{m}} \mathrm{a_i\, n^i}$$

with: $\Delta H_{ads}(n)_t$: coverage-dependent isosteric enthalpie of adsorption (J·mol^{-1})

R: universal gas constant (J·K·mol^{-1})

n: amount of the gas adsorbed (mol·kg^{-1})

a_i: virial coefficient

m: number of the coefficients required to adequately describe the isotherms

The error of the ΔH_{ads} calculations is strongly influencing the meaningfulness of the values themselves. As shown by Janiak and co-workers[S12] not only the R^2 value of the fitting has to be taken into account but also the standard deviation of the virial coefficients.

The error of the zero (or low) coverage heat of adsorptions for $n \rightarrow 0$ can be expressed as the product of the standard deviation of virial coefficient a_0 and the gas constant:

$$\Delta H_{ads,\,0} = R \cdot \Delta a_0$$

Fittings with insufficient fitting parameters or non-converging fits are not further discussed or depicted (Table S19)

For a comparison with reported isosteric heat of adsorption data, the general assumption

$$\Delta H_{ads} = -Q_{st}$$

was considered.S[12]

Table S19. ΔH_{ads} data obtained in this study.

gas / cmp.	PFC-14	PFC-116	PFC-218	PFC-318[a]	SF_6	NF_3
**CF_3-cage**	-31.4±4.8	-23.8±3.8	-[b]	-[b]	-[b]	-[b]
**C_2F_5-cage**	-27.1±3.8	-28.1±0.6	-32.0±1.1	-54.6±2.2	-22.9±3.9	-19.5±0.4
**C_3F_7-cage**	-17.6±1.5	-26.1±0.7	-22.9±2.4	-80.1±16.7[c]	-30.7±4.2	-22.9±2.4
**C_4F_9-cage**	-24.1±2.0	-31.6±0.3	-40.1±0.8	-46.2±0.8	-33.2±1.7	-
**C_5F_{11}-cage**	-26.4±1.6	-27.2±0.9	-32.5±1.3	-32.7±2.0	-25.1±5.3	-17.6±1.1
**C_6F_{13}-cage**	-24.1±4.6	-17.4±2.7	-[b]	-[b]	-[b]	-[b]

[a] Due to the pore condensation of PFC-318 at pressures above 0.5 bar only pressure points >0.5 bar have been considered. [b] not determined due to insufficient fitting parameters or non-converging fits. [c] Non-reliable value due to insufficient fitting parameters.

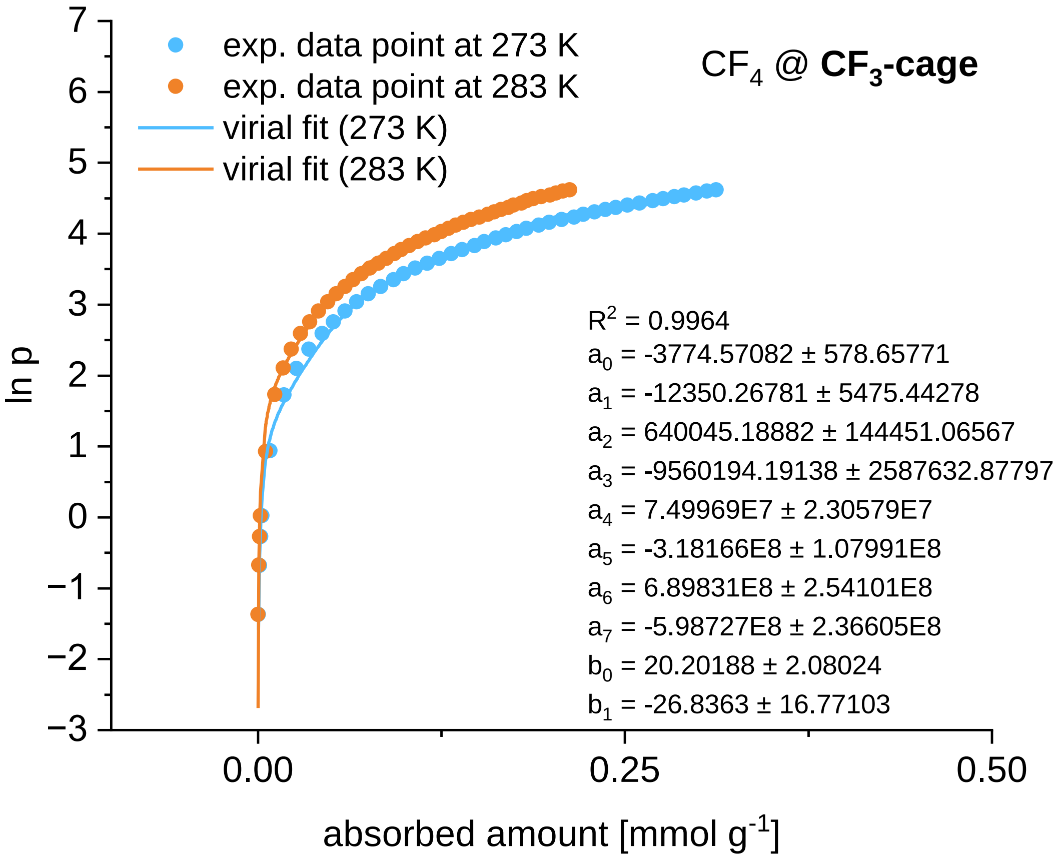

Figure S353. CF_4 isotherms at 273 K and 283 K and virial fitting curves and parameters of **CF_3-cage**.

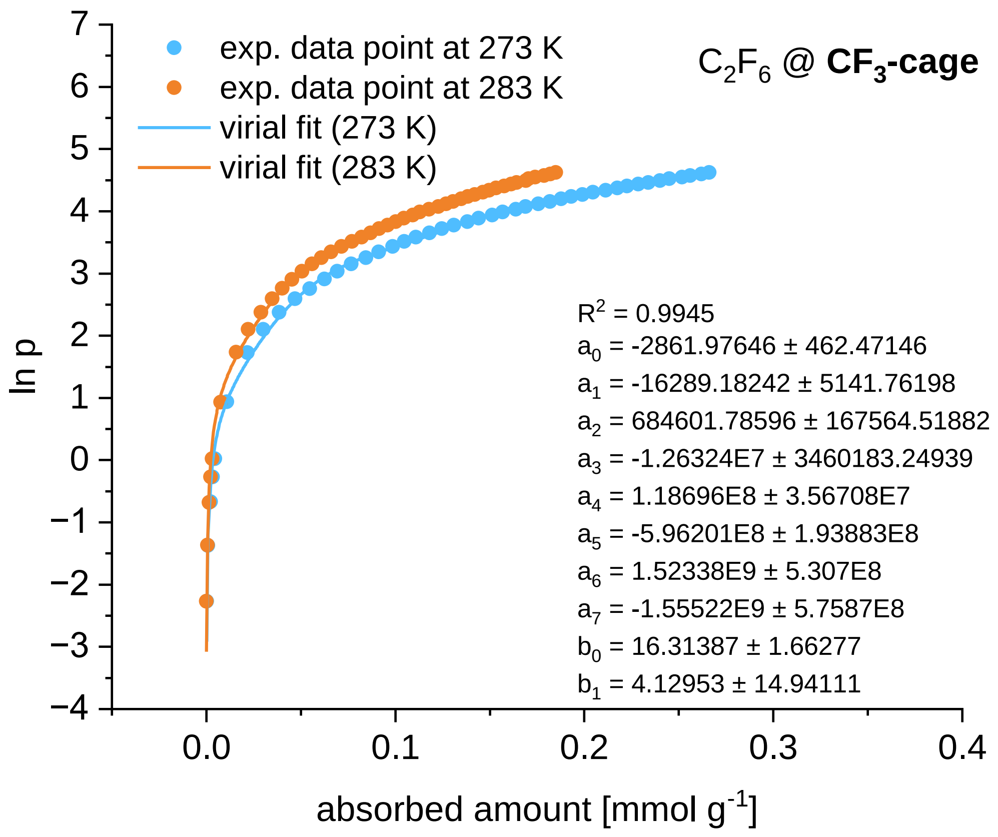

Figure S354. C_2F_6 isotherms at 273 K and 283 K and virial fitting curves and parameters of **CF_3-cage**.

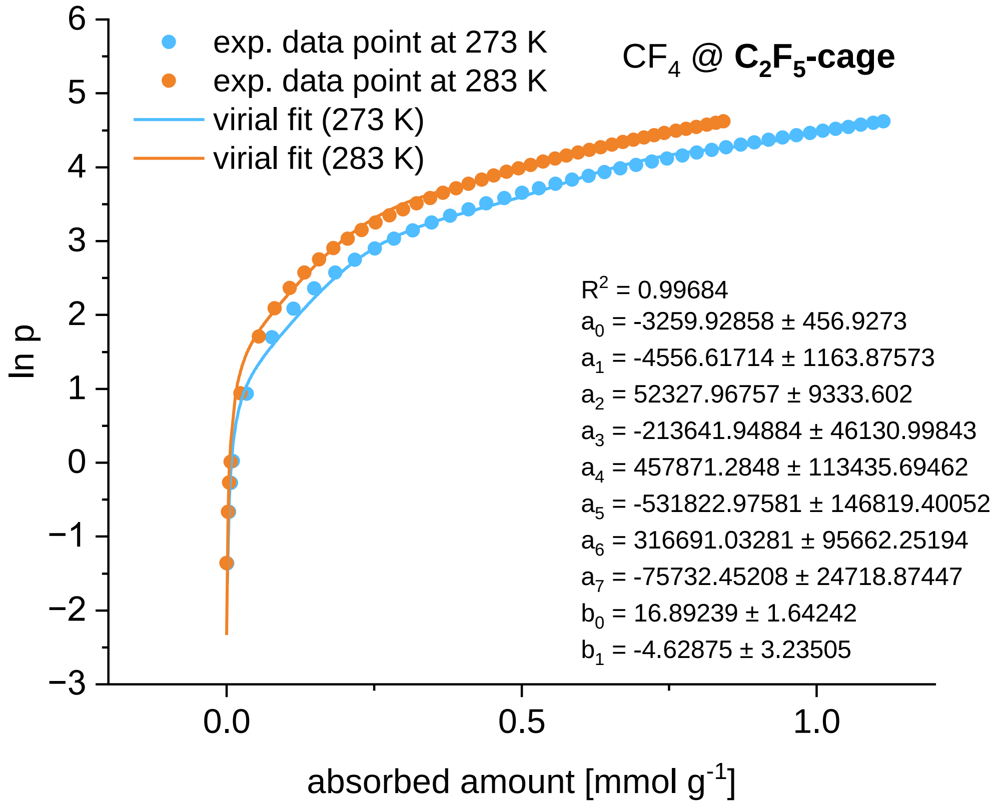

Figure S355. CF_4 isotherms at 273 K and 283 K and virial fitting curves and parameters of **C_2F_5-cage**.

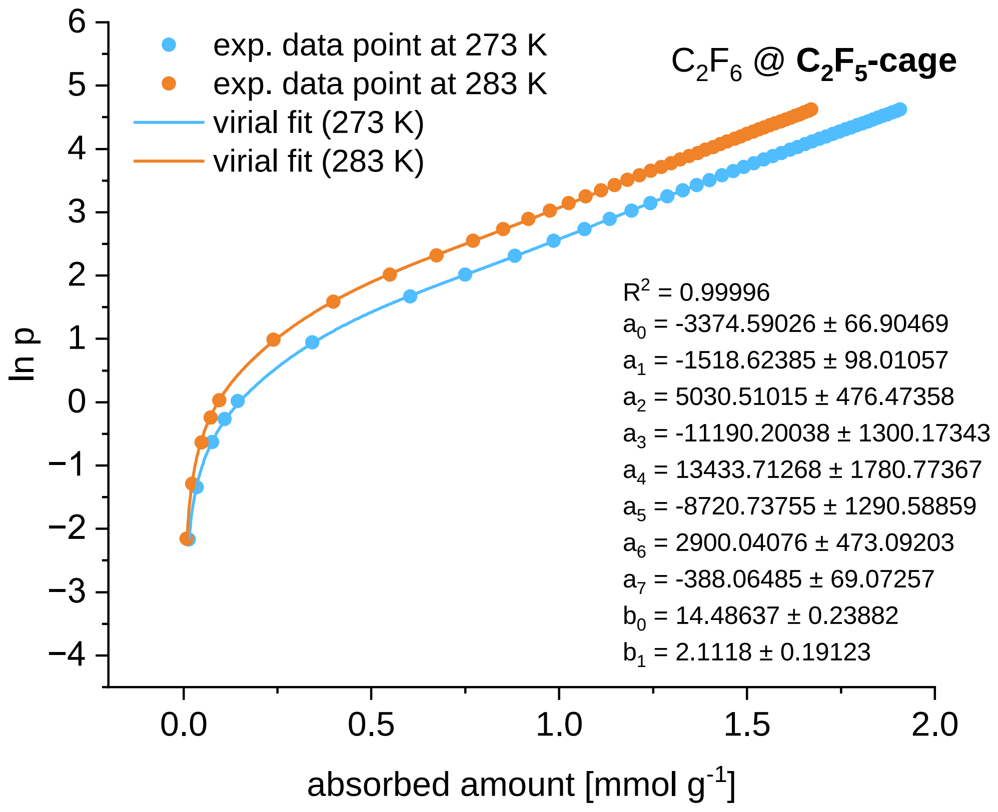

Figure S356. C_2F_6 isotherms at 273 K and 283 K and virial fitting curves and parameters of **C_2F_5-cage**.

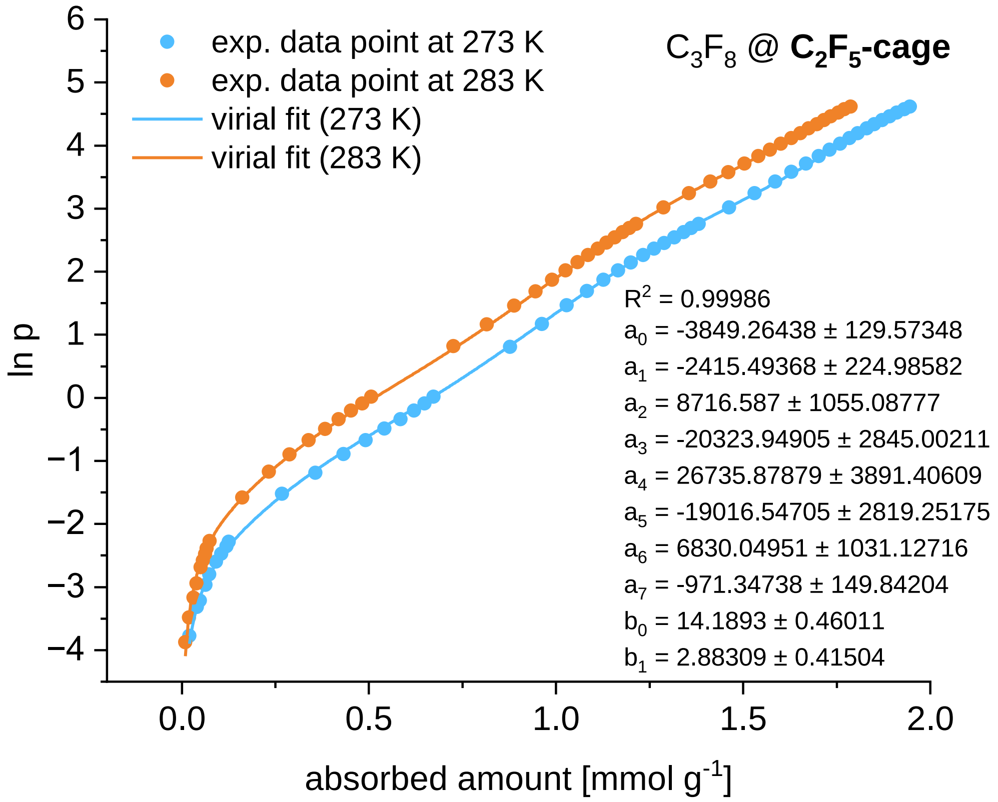

Figure S357. C_3F_8 isotherms at 273 K and 283 K and virial fitting curves and parameters of **C_2F_5-cage**.

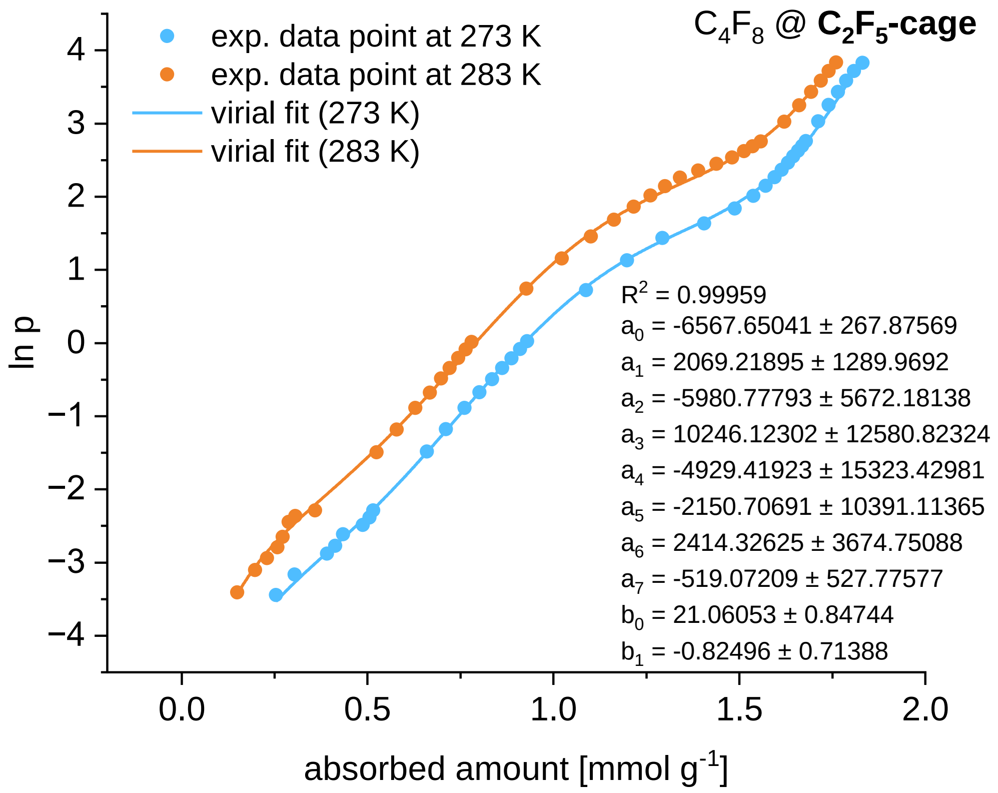

Figure S358. C_4F_8 isotherms at 273 K and 283 K and virial fitting curves and parameters of **C_2F_5-cage**.

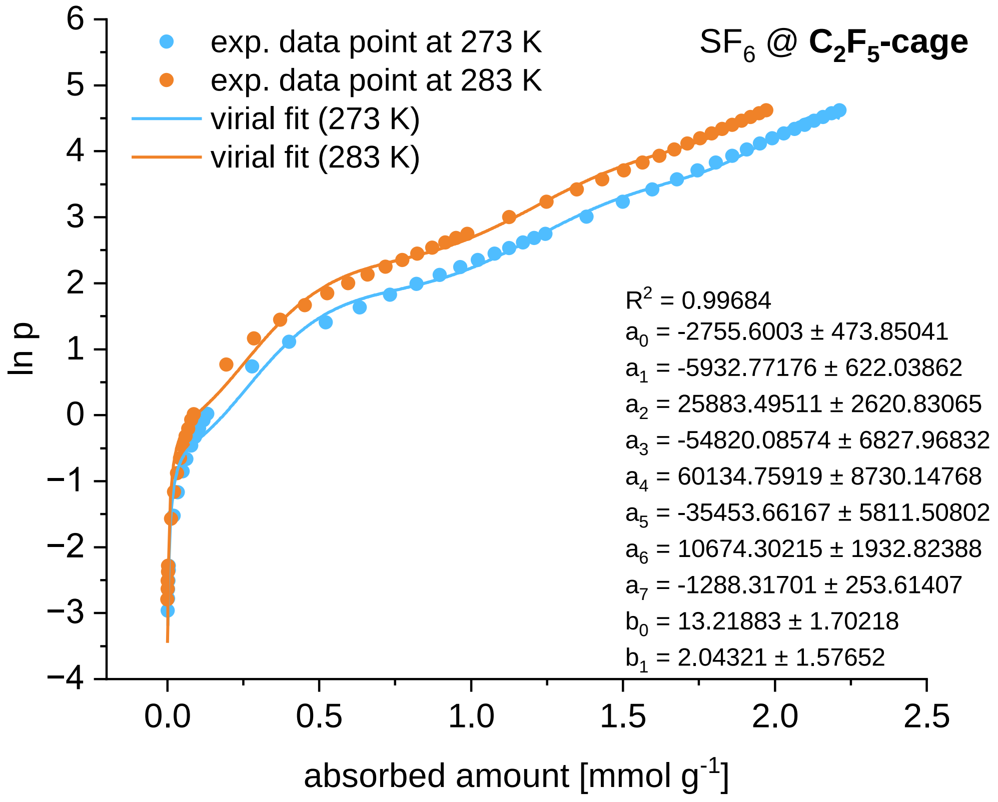

Figure S359. SF_6 isotherms at 273 K and 283 K and virial fitting curves and parameters of **C_2F_5-cage**.

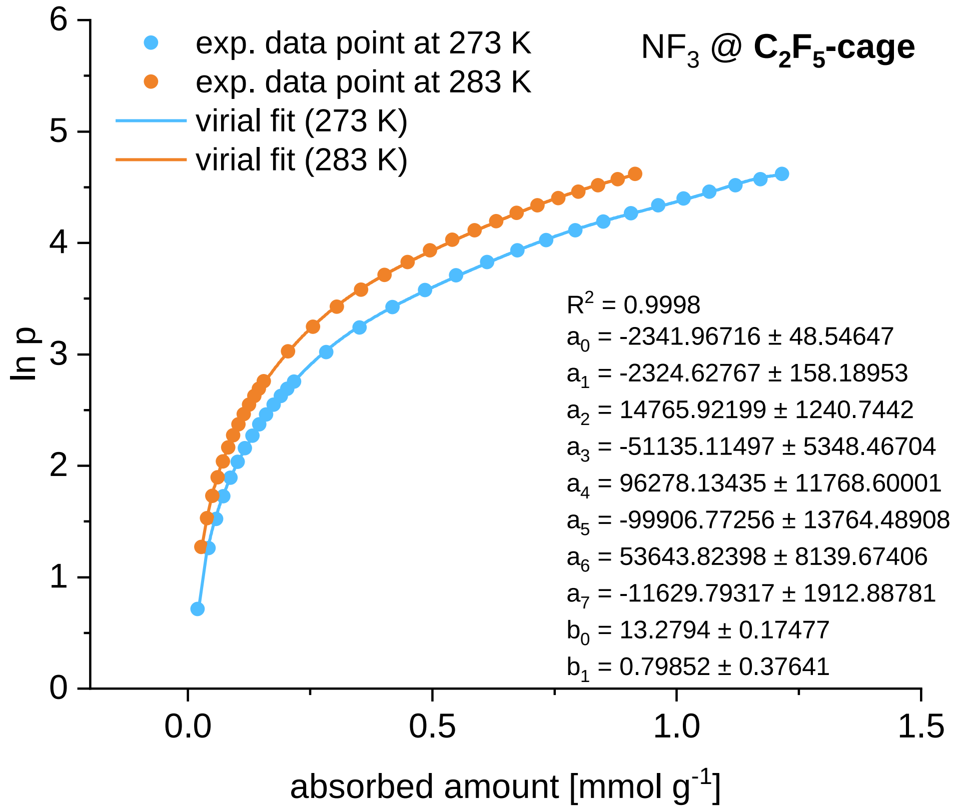

Figure S360. NF_3 isotherms at 273 K and 283 K and virial fitting curves and parameters of **C_2F_5-cage**.

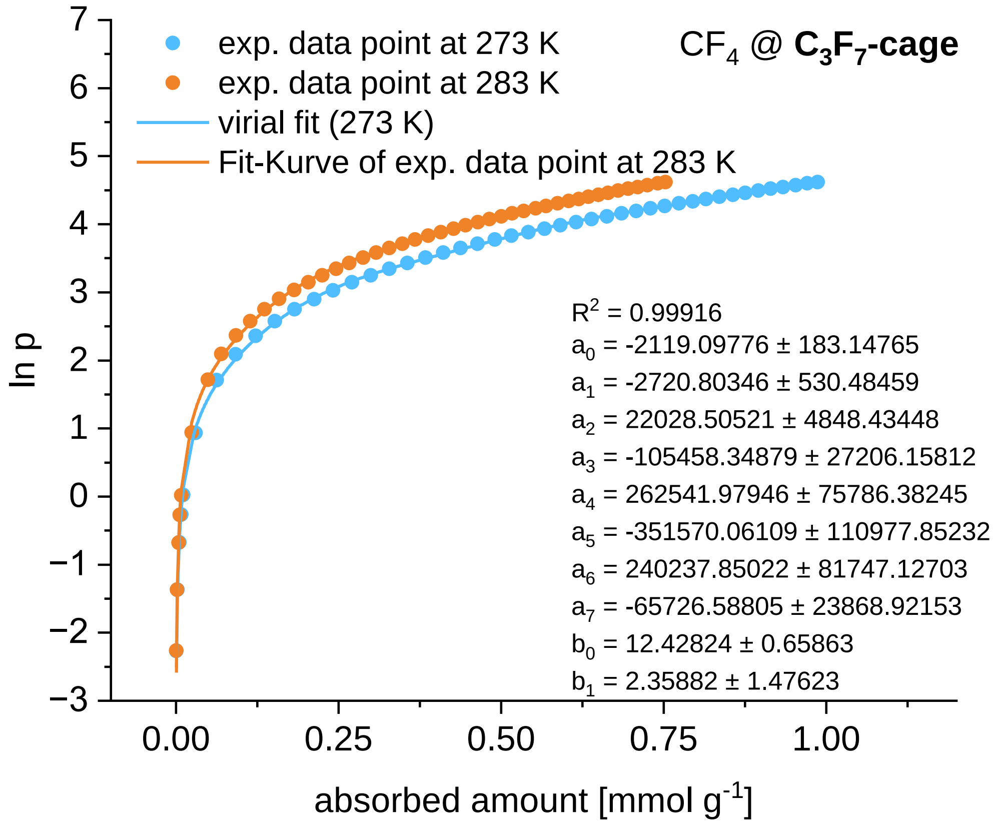

Figure S361. CF_4 isotherms at 273 K and 283 K and virial fitting curves and parameters of **C_3F_7-cage**.

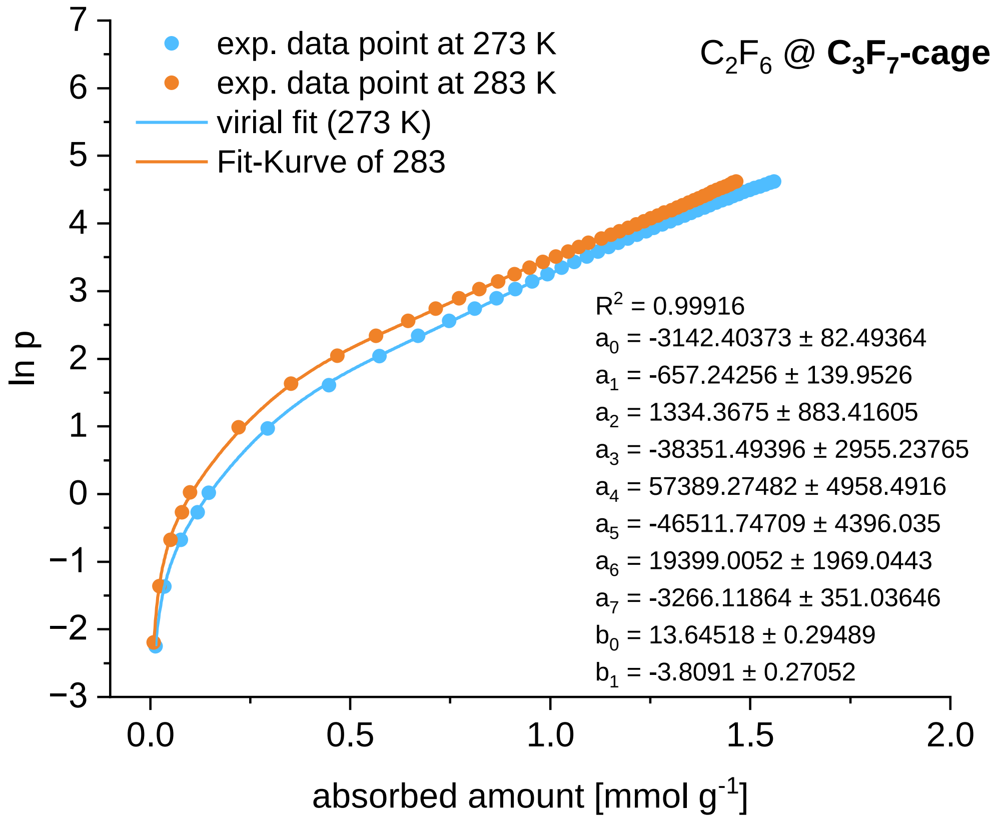

Figure S362. C_2F_6 isotherms at 273 K and 283 K and virial fitting curves and parameters of **C_3F_7-cage**.

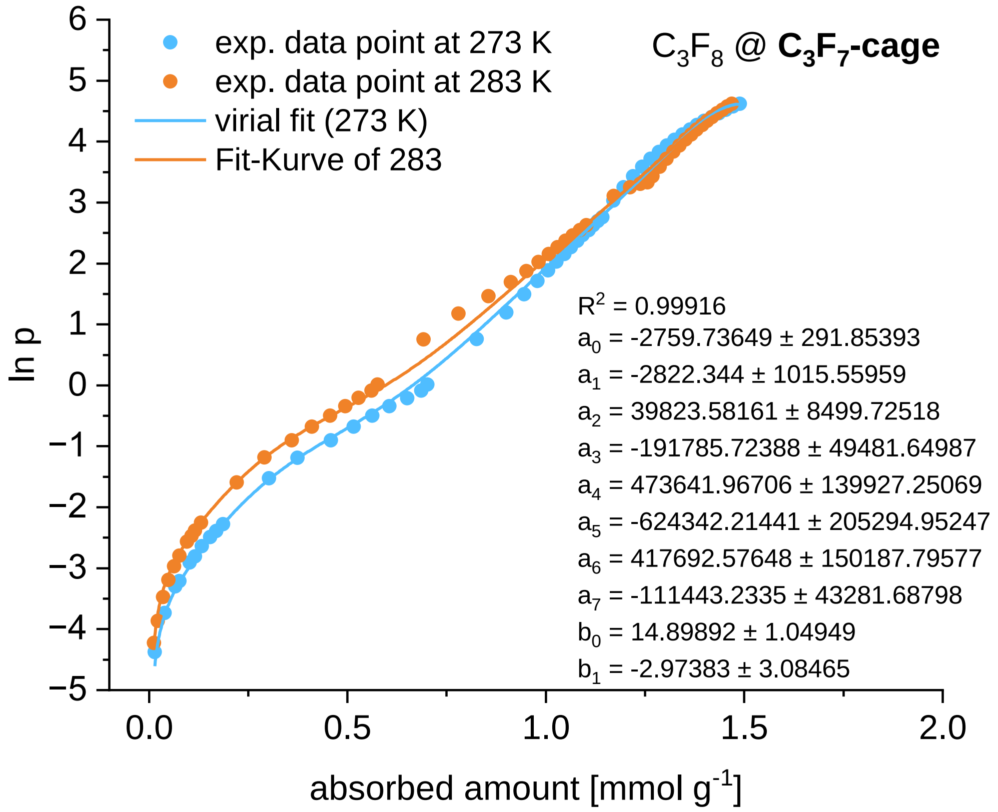

Figure S363. C_3F_8 isotherms at 273 K and 283 K and virial fitting curves and parameters of **C_3F_7-cage**.

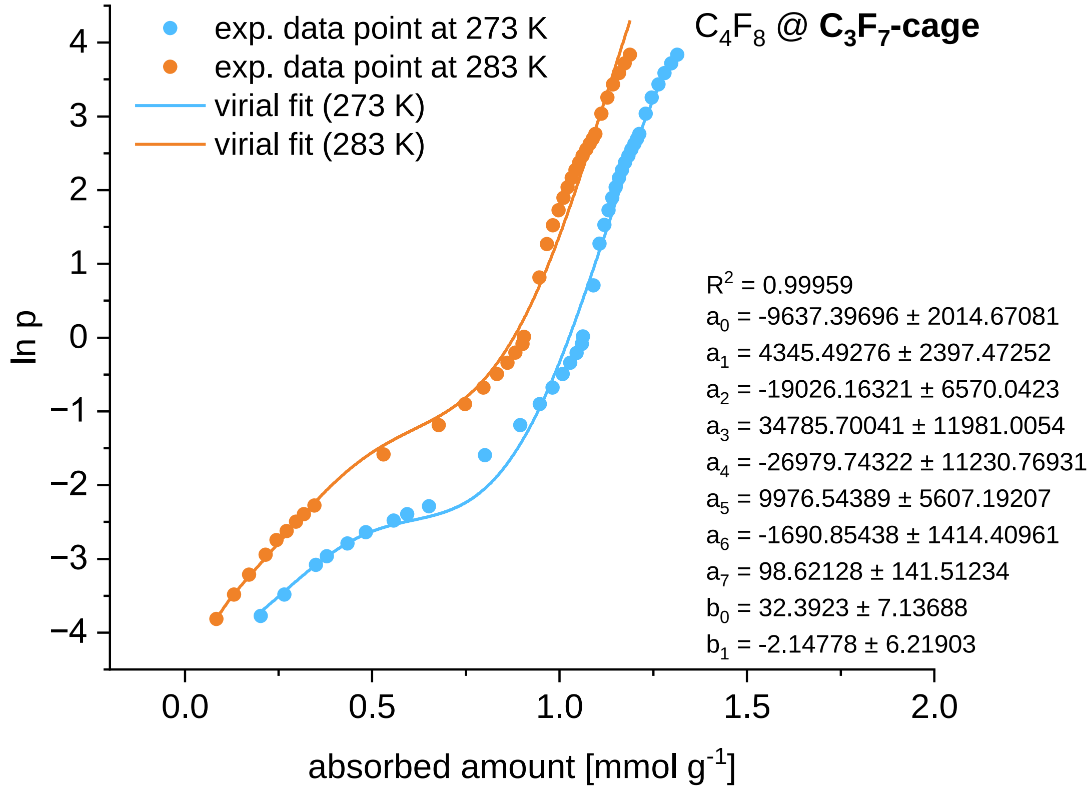

Figure S364. C_4F_8 isotherms at 273 K and 283 K and virial fitting curves and parameters of **C_3F_7-cage**.

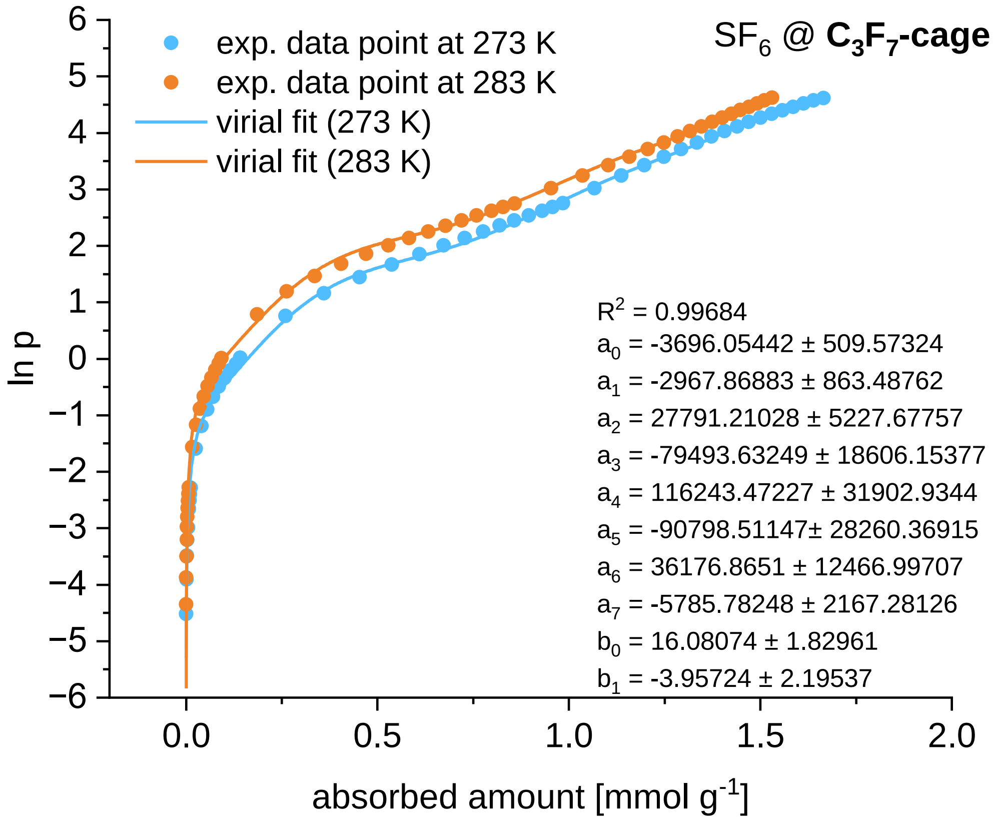

Figure S365. SF_6 isotherms at 273 K and 283 K and virial fitting curves and parameters of **C_3F_7-cage**.

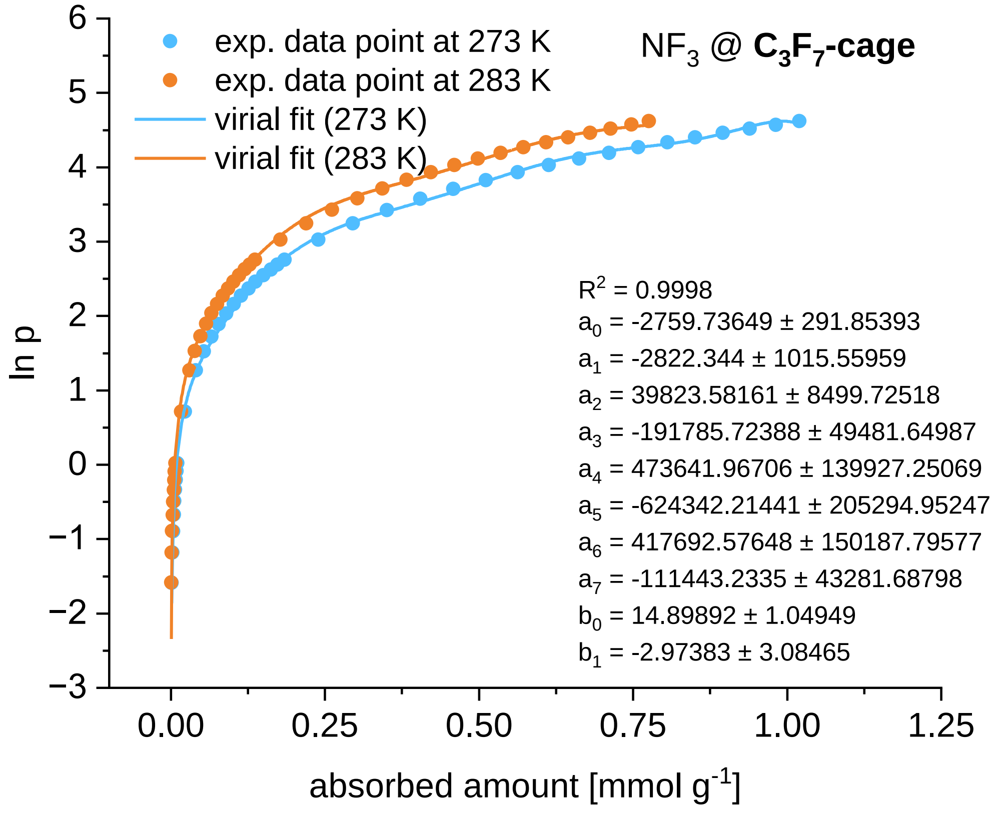

Figure S366. NF_3 isotherms at 273 K and 283 K and virial fitting curves and parameters of **C_3F_7-cage**.

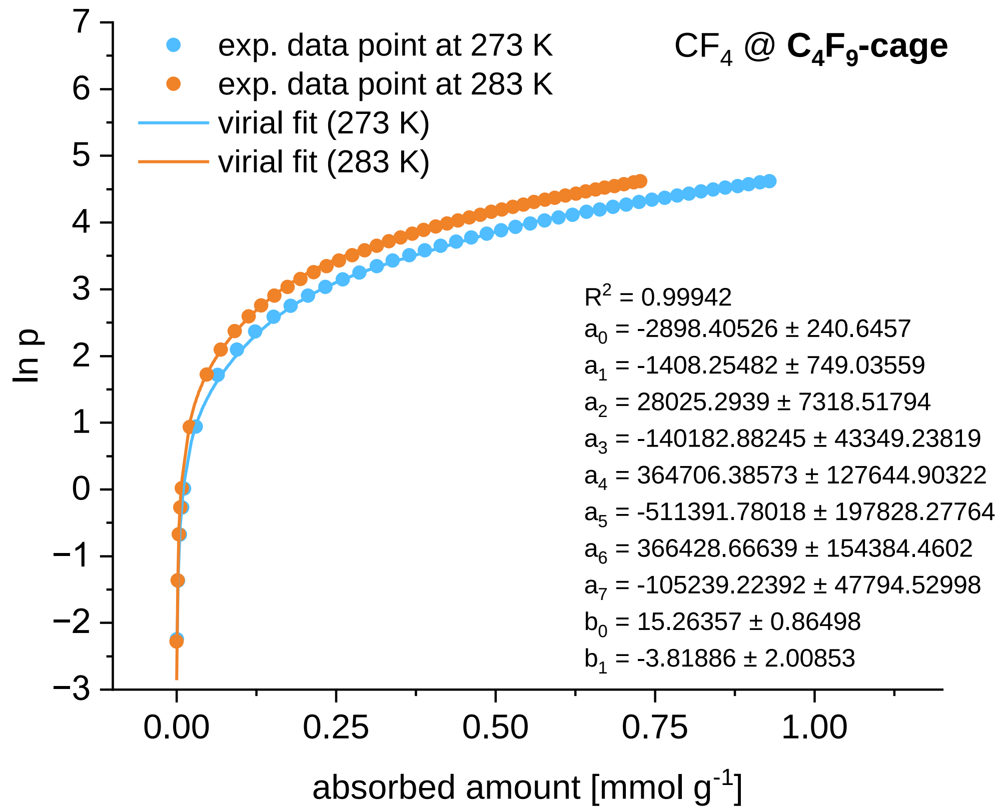

Figure S367. CF_4 isotherms at 273 K and 283 K and virial fitting curves and parameters of **C_4F_9-cage**.

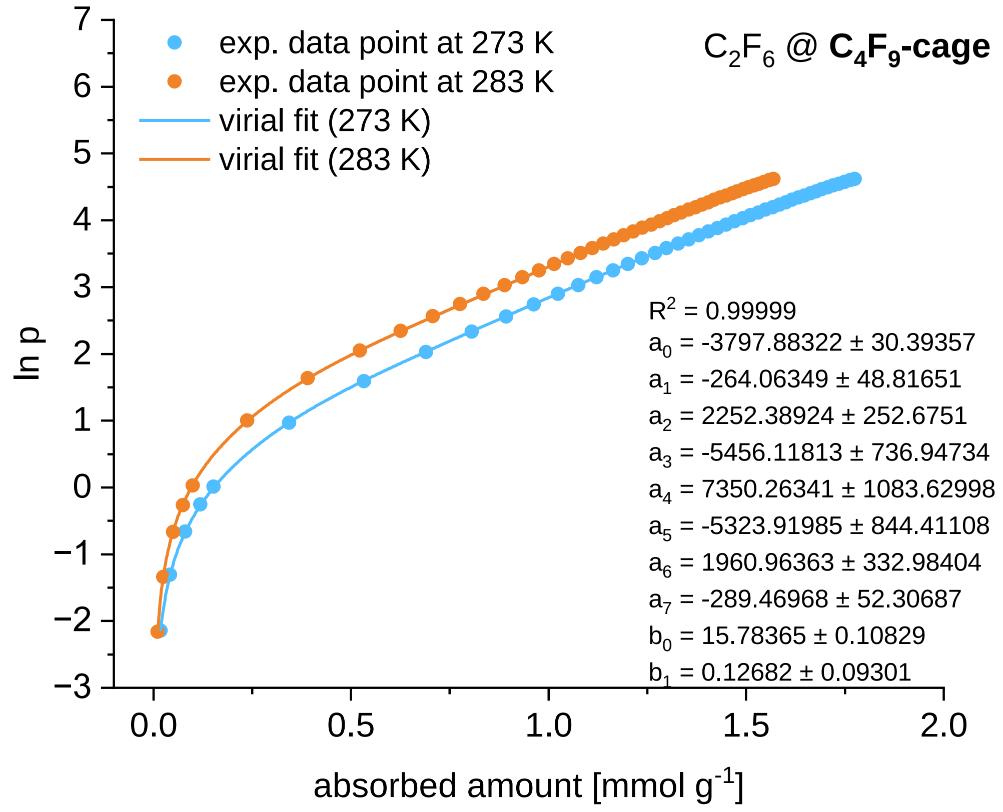

Figure S368. C_2F_6 isotherms at 273 K and 283 K and virial fitting curves and parameters of **C_4F_9-cage**.

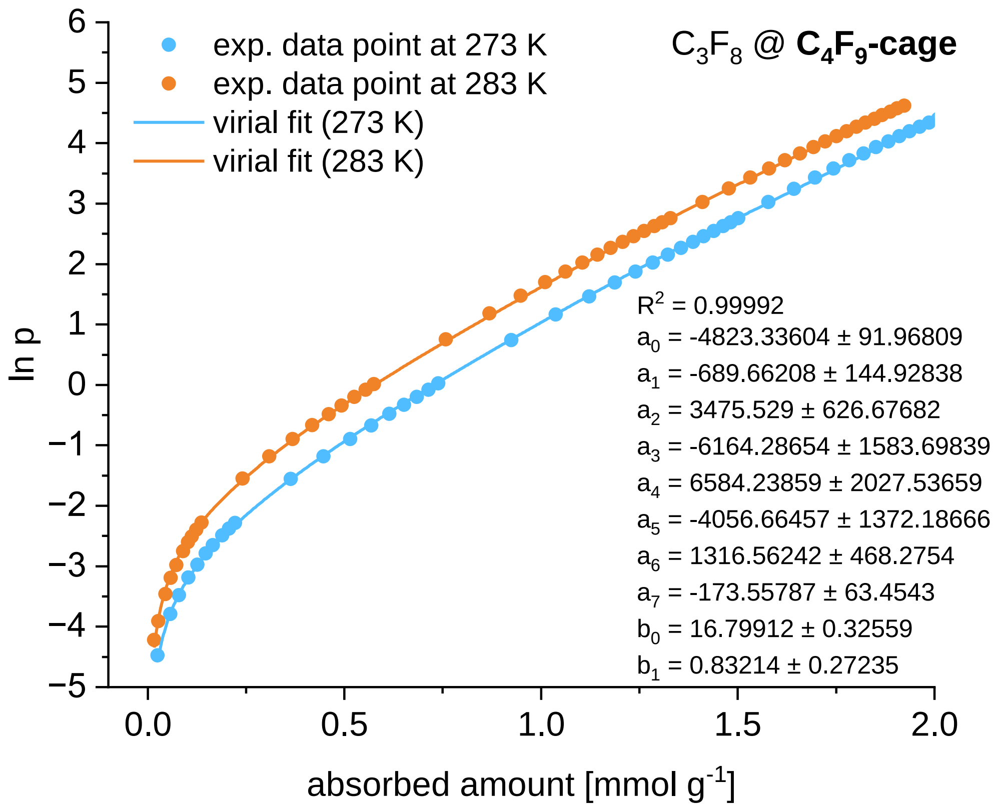

Figure S369. C_3F_8 isotherms at 273 K and 283 K and virial fitting curves and parameters of **C_4F_9-cage**.

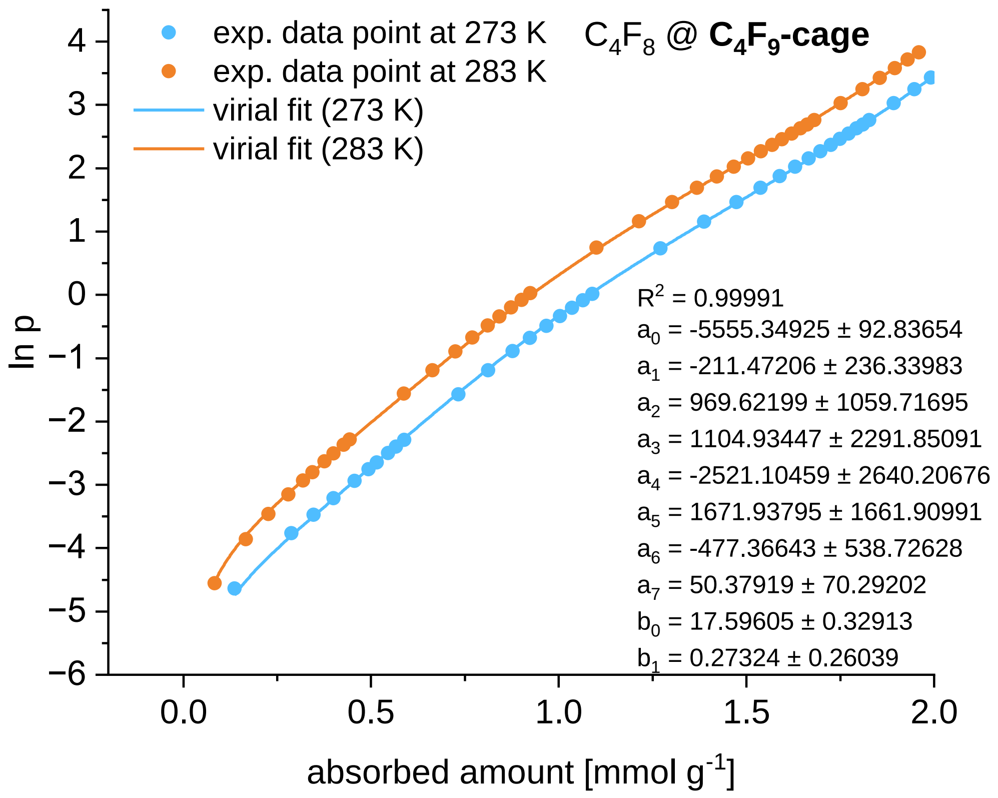

Figure S370. C_4F_8 isotherms at 273 K and 283 K and virial fitting curves and parameters of **C_4F_9-cage**.

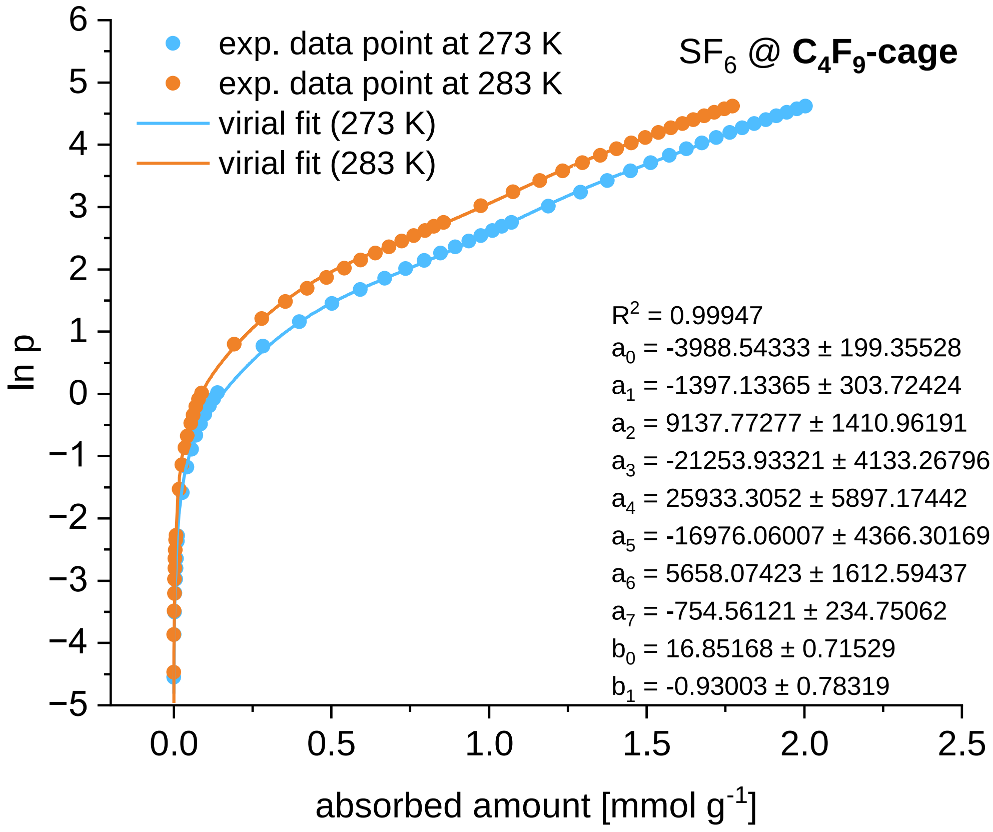

Figure S371. SF_6 isotherms at 273 K and 283 K and virial fitting curves and parameters of **C_4F_9-cage**.

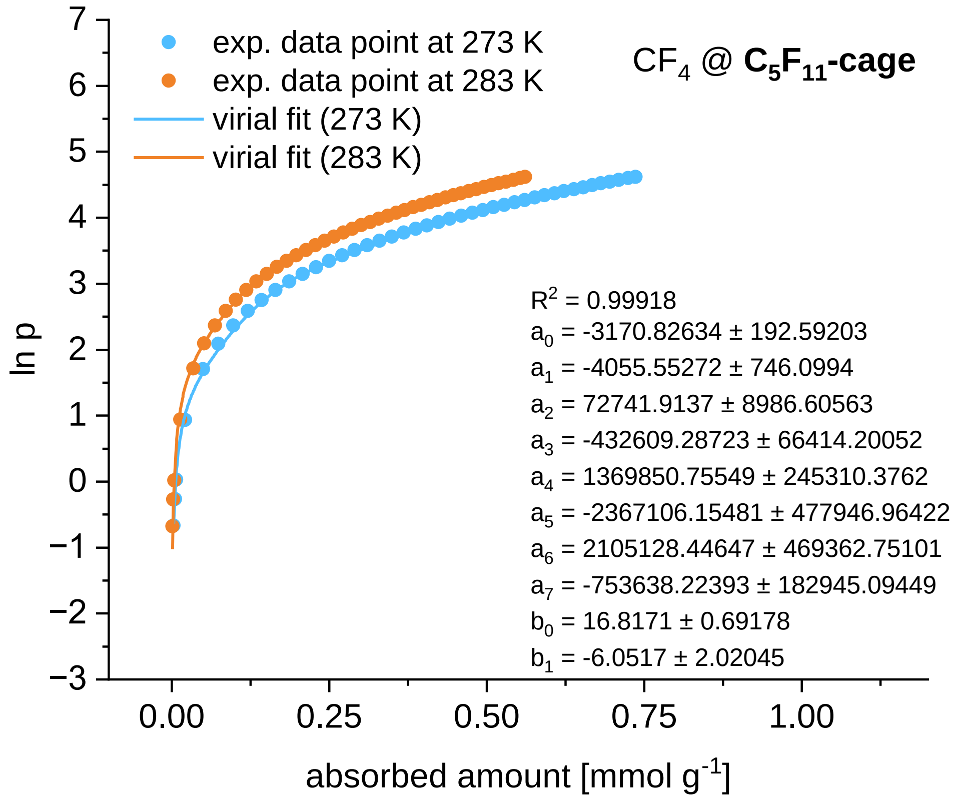

Figure S372. CF_4 isotherms at 273 K and 283 K and virial fitting curves and parameters of **C_5F_{11}-cage**.

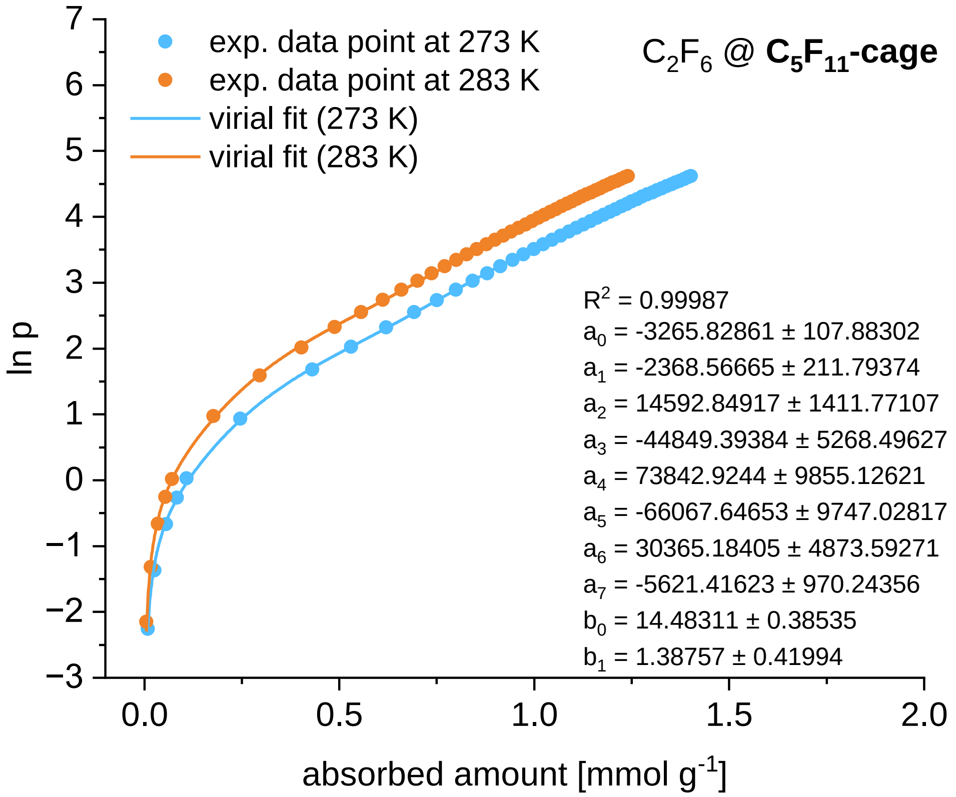

Figure S373. C_2F_6 isotherms at 273 K and 283 K and virial fitting curves and parameters of **C_5F_{11}-cage**.

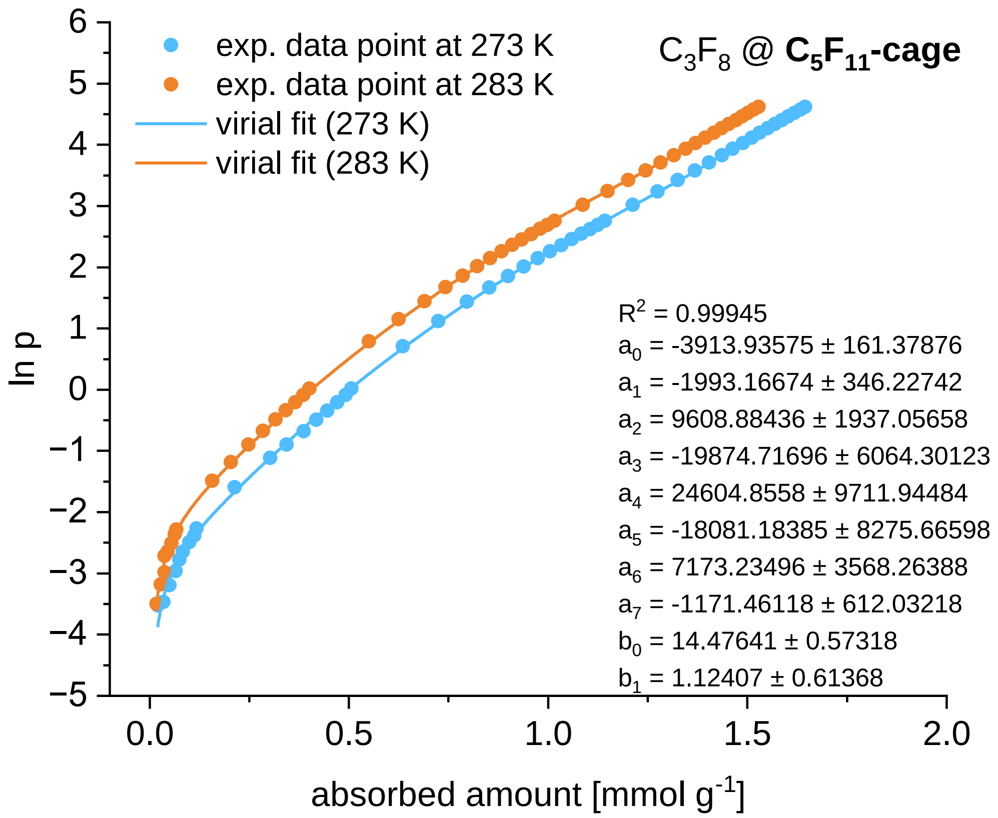

Figure S374. C_3F_8 isotherms at 273 K and 283 K and virial fitting curves and parameters of **C_5F_{11}-cage**.

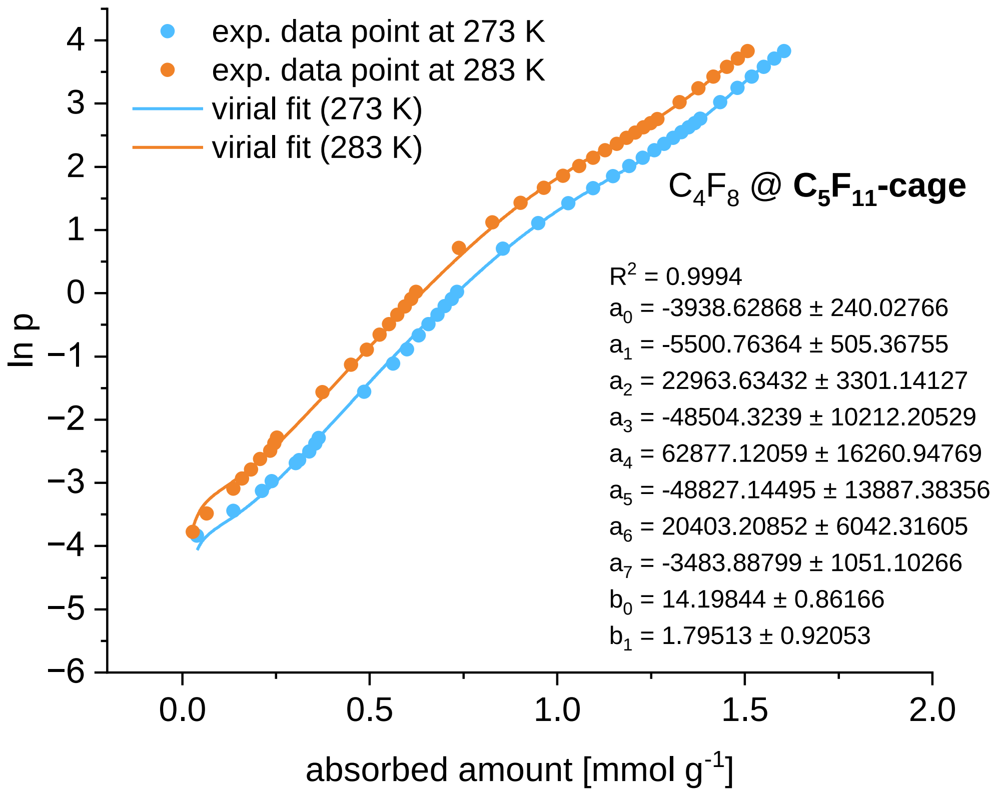

Figure S375. C_4F_8 isotherms at 273 K and 283 K and virial fitting curves and parameters of **C_5F_{11}-cage**.

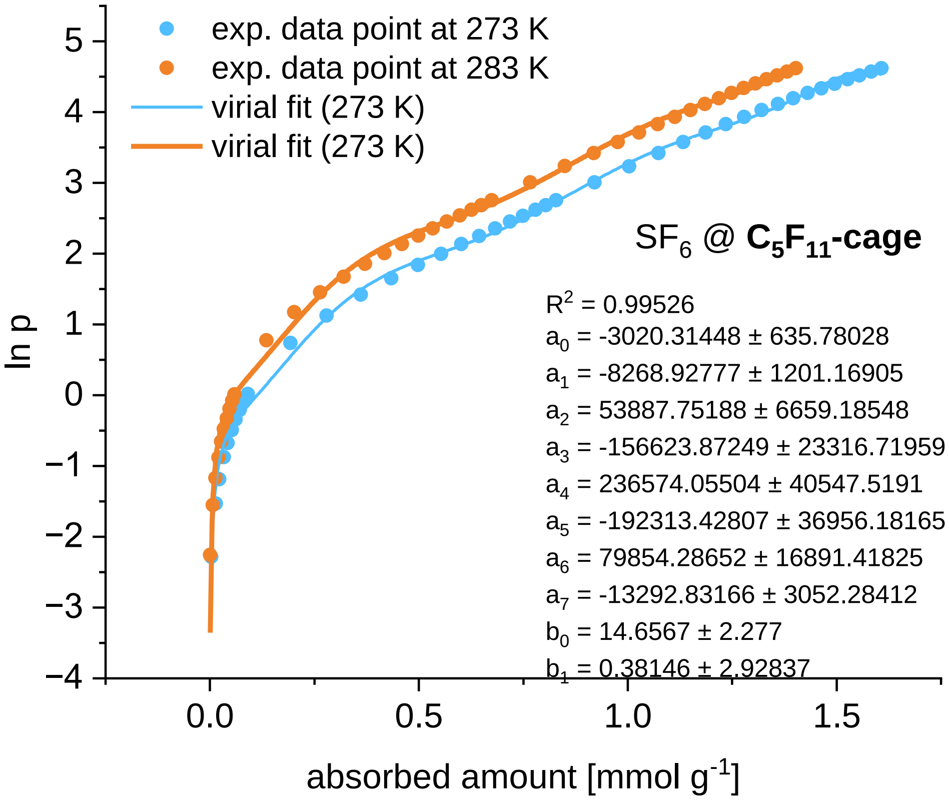

Figure S376. SF_6 isotherms at 273 K and 283 K and virial fitting curves and parameters of **C_5F_{11}-cage**.

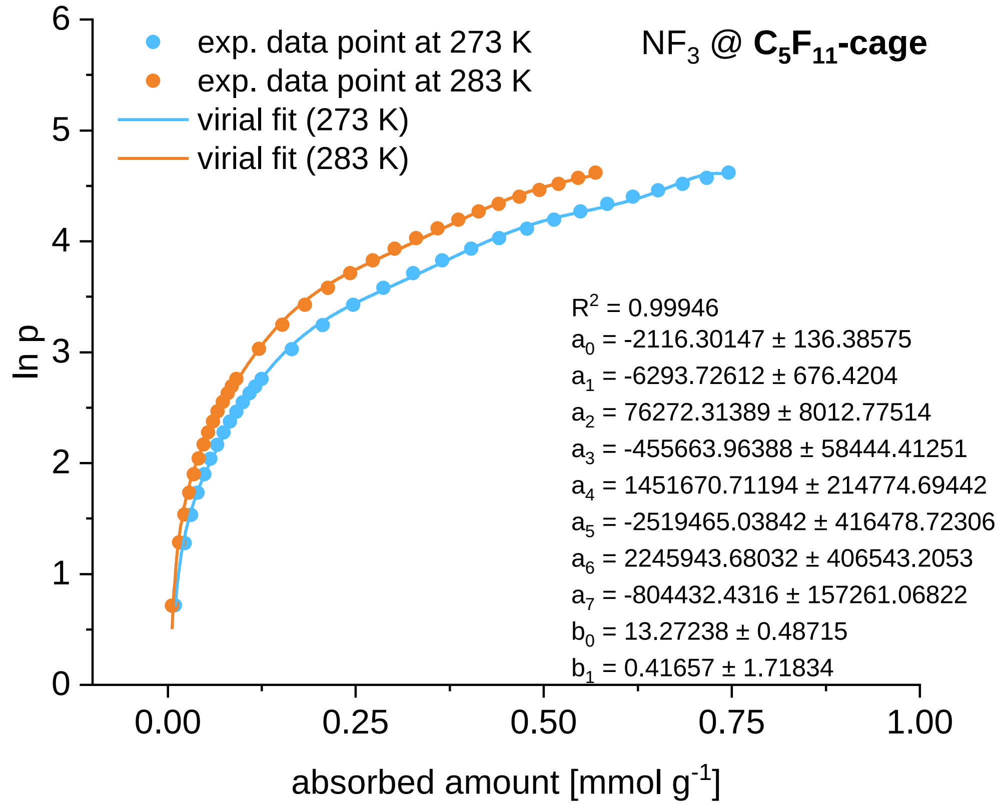

Figure S377. NF_3 isotherms at 273 K and 283 K and virial fitting curves and parameters of **C_5F_{11}-cage**.

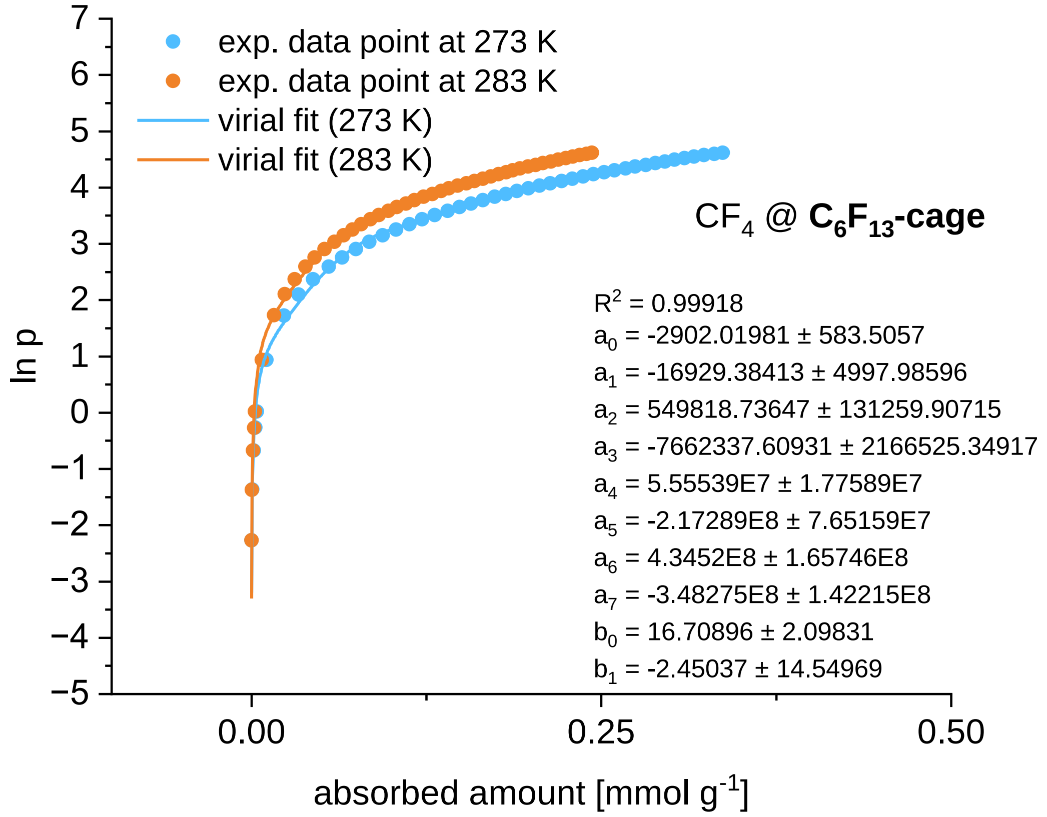

Figure S378. CF_4 isotherms at 273 K and 283 K and virial fitting curves and parameters of **C_6F_{13}-cage**.

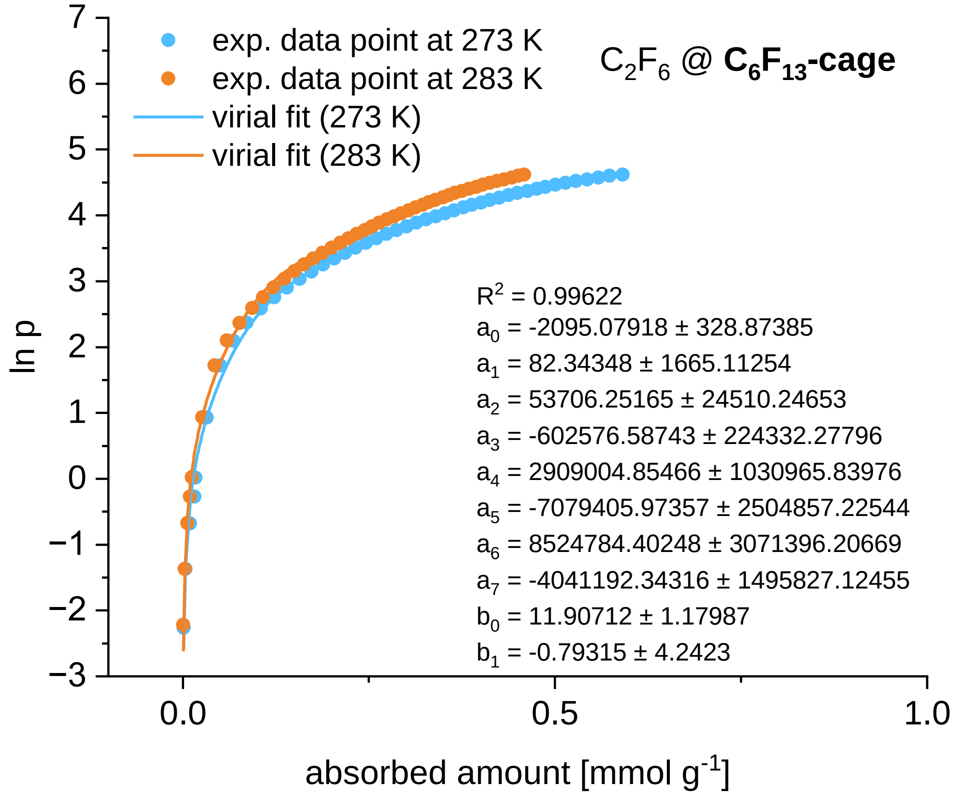

Figure S379. C_2F_6 isotherms at 273 K and 283 K and virial fitting curves and parameters of **C_6F_{13}-cage**.

Breakthrough Experiments

The experimental breakthroughs depicted in Figure 6 of the manuscript have been conducted using a 3P instruments MixSorb S system coupled with an MKS Cirrus-3 mass spectrometer.

C_2F_5-cage (0.169 g) was packed in an adsorber with 4.5 mm inner diameter, sealed and flushed with nitrogen. The sample was initially pre-treated in a flow of helium at 50 °C for 2 h and for an additional hour at 50 °C after each measurement. After measurements with relative humidity, the sample was pre-treated in a flow of helium at 100 °C to ensure complete removal of excess water. The progress was monitored by MS in selected cases.

The gas mixtures were realized in a stream of 50% helium as carrier gas.

Table S20. Breakthrough experiments of **C_2F_5-cage** conducted in this study.

Exp.	gas mixture	ratio	temp. [K]	conditions	flow rate [mL min]
1	PFC-318/N_2	10:90	298 K	dry	10
2	PFC-318/N_2	10:90	313 K	dry	10
3	PFC-318/N_2	1:99	298 K	dry	10
4	PFC-318/N_2	1:99	313 K	dry	10
5	PFC-318/N_2	10:90	298 K	12% RH	10
6	PFC-318/N_2	10:90	298 K	25% RH	10
7	PFC-318/N_2	10:90	298 K	50% RH	10
8	PFC-318/Air[1]	10:90	298 K	dry	10
9	PFC-318/Air [2]	10:90	298 K	dry	20[3]
10[4]	PFC-318/Air	10:90	298 K	dry	10

[1] synthetic air (79.5% nitrogen + 20.5% oxygen). [2] synthetic air (79.5% nitrogen + 20.5% oxygen) + 400 ppm CO_2 in helium. [3] Measurement was performed with a flow rate of 20 mL min^{-1} due to the flow rate range of the CO_2 mass flow controller. [4] Three repetitive cycles have been performed under similar conditions as in experiment 8 to prove the recyclability of the material.

For the determination of the uptake capacities:

1. the breakthrough curves have been integrated up to the point where steps derived from artefacts of the detector were observed (Figure S379),
2. a blank measurement was treated accordingly,
3. the blank uptakes were subtracted from the experimental uptake.

It is worth mentioning, that the determination of the nitrogen uptake capacities has larger error margins due the immediate breakthroughs within the blank time. This potentially leads to an underestimation of the obtained selectivities because a nitrogen capacity close to zero is to be expected.

Table S21. Gas sorption parameters obtained from the breakthrough experiments of **C_2F_5-cage**.

Exp.	N_2 capacity [mmol g^{-1}]	C_4F_8 capacity [mmol g^{-1}]	*y* N_2	*y* C_4F_8	Selectivity
1	0.16	0.85	0.45	0.05	47.8
2	0.06	0.69	0.45	0.05	103.5
3	0.21	0.54	0.45	0.005	231.4
4	0.19	0.4	0.45	0.005	189.5
5	0.46	0.83	0.446	0.05	16.1
6	0.43	0.88	0.442	0.05	18.1
7	0.32	0.88	0.435	0.05	23.9
8	0.2	0.87	0.358	0.05	31.1
9	0.1	0.86	0.358	0.05	61.6

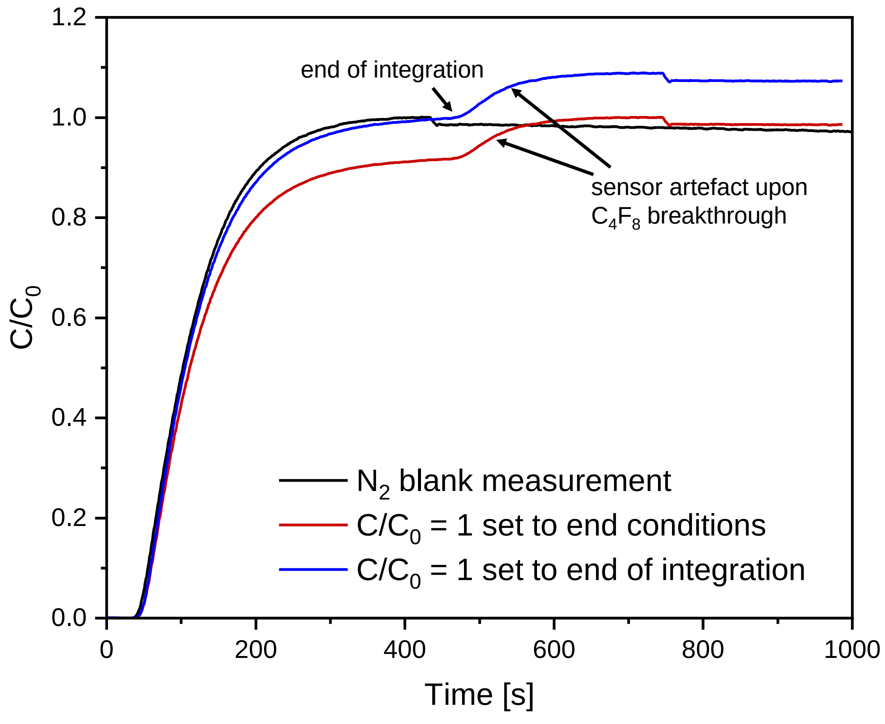

Figure S380. Exemplary procedure for the determination of the nitrogen capacities.

10. Stability Investigations

To test the long-term stability of the porous materials, **C_2F_5-cage** was investigated exemplarily. The authentic sample was first investigated directly after crystallization (for details see synthetic procedures) and then again after being stored for ~3.5 years(!) under ambient conditions without air conditioning (meaning temperature fluctuations between ~18 °C and 35 °C and different levels of humidity). For the gas adsorption study the sample was activated at 50 °C for 3 h in dynamic vacuum.

By PXRD investigations, nearly identical spectra were obtained indicating the stability of the crystalline lattice even after being stored for 3.5 year (Figure S381). This assumption is further underlined by near identical nitrogen sorption isotherms (Figure S382) and corresponding BET surface areas of 921 $m^2\ g^{-1}$ (initial measurement) and 904 $m^2\ g^{-1}$ (after 3.5 years).

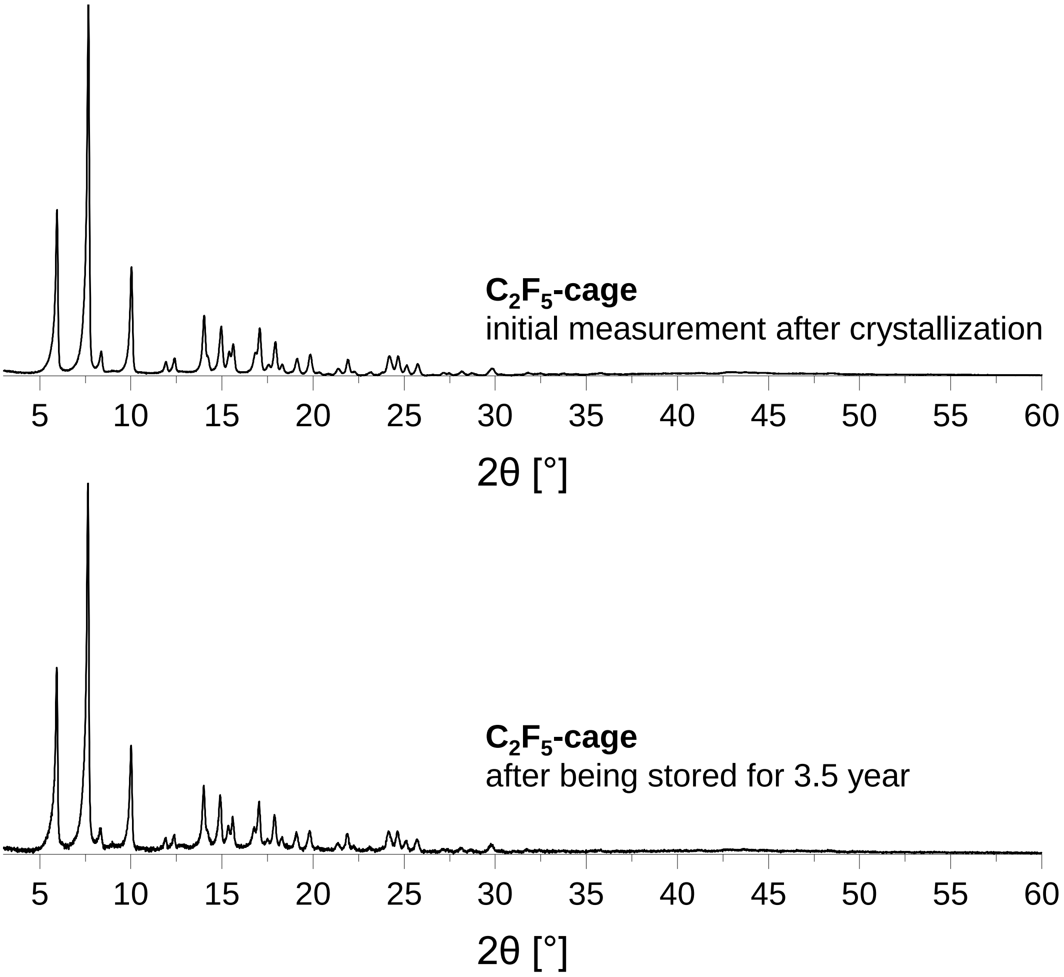

Figure S381. PXRD measurements of initial material (top) and a repeated measurement after storage for 3.5 years (bottom).

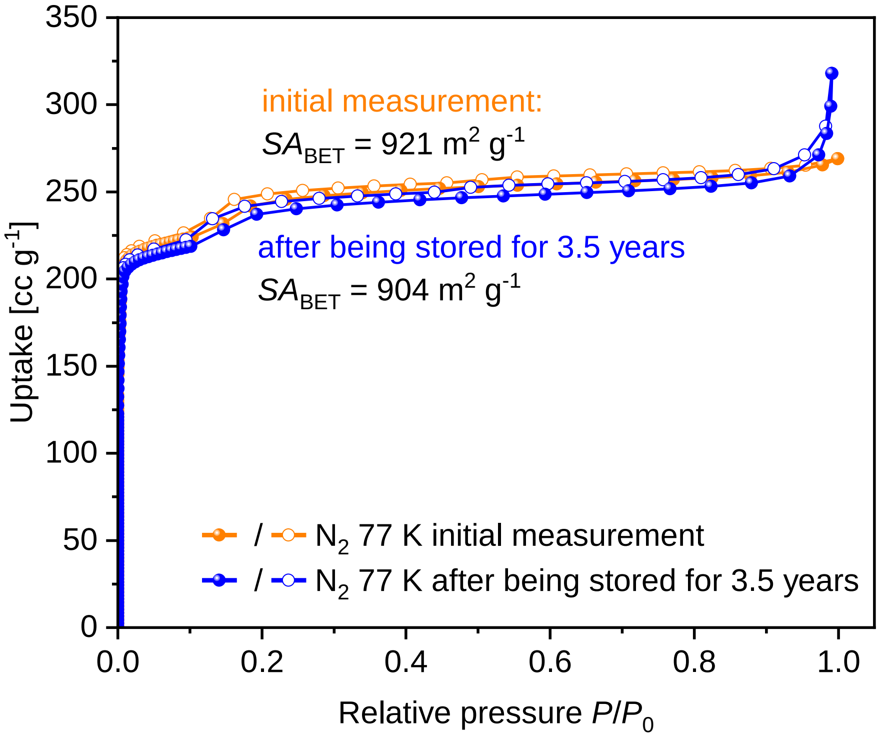

Figure S382. Nitrogen sorption isotherms of the initial material (orange) and a repeated measurement after storage for 3.5 years (blue). Full circles: adsorption; empty circles: desorption.

The chemical stability of cages was furthermore proven by identical ^{1}H-NMR spectra of the freshly synthesized as well as after 3.5 years redissolved material (Figure S383).

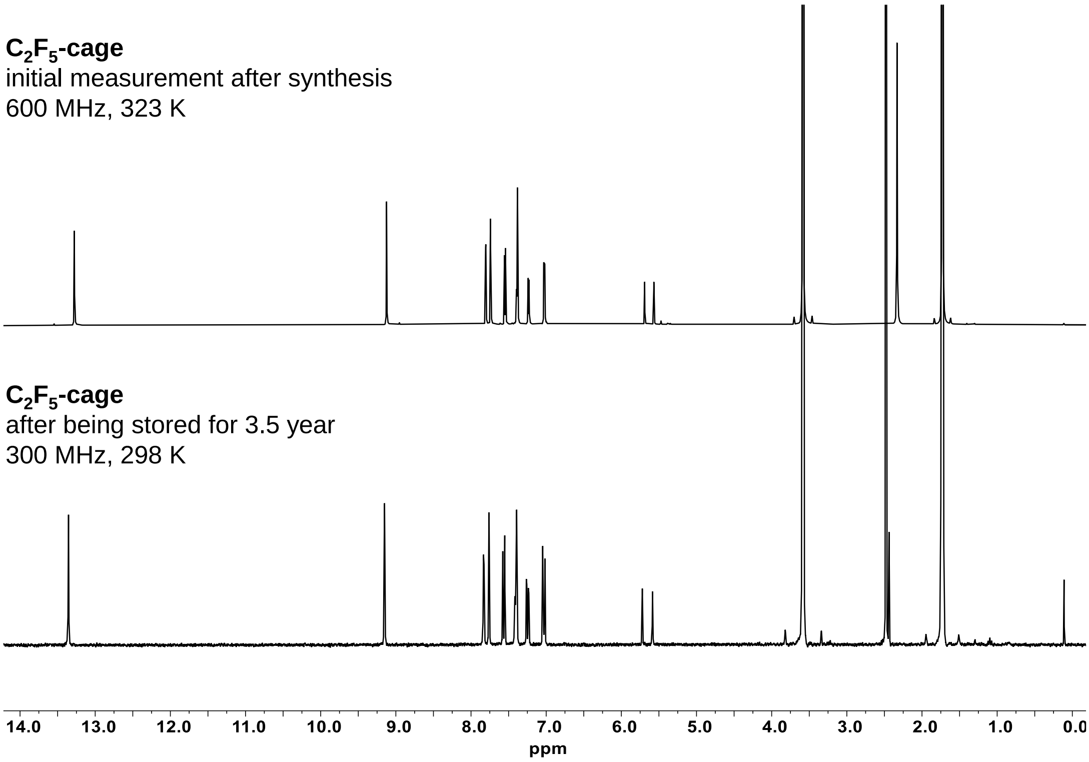

Figure S383. ^{1}H-NMR spectra of the initial material (top, 600 MHz, 323 K) and a repeated measurement after storage for 3.5 years (bottom, 300 MHz) in THF-d_6.

11. Quantumchemical Calculations

All quantum chemical calculations were performed by employing the Gaussian16 program package.[S13] The theoretical approach is based on Kohn-Sham density functional methodologies[S14] using the B3LYP[S15] functional. As basis set the augmented, polarised triple-ζ-basis (aug-cc-pVDZ[S16]) was used. For the calculations the quadratic convergence criteria, as implemented in Gaussian16, were applied. The dispersion energies of the corresponding fragments were calculated applying the exchange-hole dipole moment (XDM) model using the postg program package developed by the Johnson group.[S17] The corresponding geometries were cut outs from the single-crystal X-ray structures. To obtain the geometries, all excess atoms were selected and deleted using GaussView6 (v6.1.1) and the "missing" H atoms added via the "Add valence" function. The wfx-files resulting from the cacluations were post-processed using postg, applying the damping parameters listed for the B3LYP/aug-cc-pVDZ combination (a1 = 0.6224, a2 = 1.7068). The quantum chemical calculations of **C_4F_9-cage** already have been reported.[S4]

For the calculation of the IGMH surfaces,[S18] the geometry from the single crystal X-ray structure of $(C_3F_8)_3 \subset$**C_3F_7-cage** was used and a single point calculation performed at B3LYP/aug-cc-pVDZ level of theory.[S15-16] The resulting Fchk-file was analyzed with Multiwfn 3.7[S19] and a medium quality grid. The results were plotted using Origin 2025b and VMD 1.9.3.[S20] The atomic pair contributions were calculated using the high quality grid option of Multiwfn.

CF_3-cage hexamer cutout

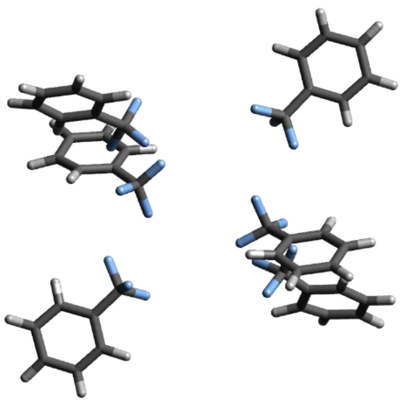

C	5.998	23.944	31.779
C	6.78	24.454	30.795
H	7.586	24.9	31.029
C	6.435	24.342	29.451
C	5.329	23.563	29.058
C	4.485	23.209	30.045
H	3.626	22.879	29.808
C	4.809	23.303	31.409
C	3.889	22.735	32.478
F	4.262	21.555	32.917
F	2.701	22.548	31.924
F	3.707	23.614	33.422
C	1.661	27.63	41.933
C	1.61	26.698	40.949
H	1.627	25.777	41.183
C	1.534	27.053	39.605
C	1.309	25.966	38.645
F	0.804	24.839	39.098
F	2.437	25.611	38.12
F	0.717	26.245	37.508
C	1.656	28.4	39.212
C	1.541	29.308	40.199
H	1.397	30.217	39.961
C	1.621	28.981	41.563
C	-1.661	16.431	31.779
C	-1.61	15.499	30.795
H	-1.627	14.578	31.029
C	-1.534	15.854	29.451
C	-1.656	17.201	29.058
C	-1.541	18.109	30.045
H	-1.397	19.018	29.808
C	-1.621	17.782	31.409
C	-1.653	18.862	32.478
F	-2.861	19.129	32.917
F	-1.221	19.985	31.924
F	-0.801	18.581	33.422
C	-4.338	26.82	31.779
C	-5.17	27.243	30.795
H	-5.959	27.717	31.029
C	-4.901	26.999	29.451
C	-3.673	26.431	29.058
C	-2.944	25.877	30.045
H	-2.229	25.299	29.808
C	-3.188	26.111	31.409
C	-2.236	25.598	32.478
F	-1.401	26.511	32.917
F	-1.48	24.663	31.924
F	-2.906	25.001	33.422
C	-5.361	21.221	41.933
C	-4.528	21.643	40.949
H	-3.74	22.118	41.183
C	-4.798	21.4	39.605
C	-3.744	21.748	38.645
F	-2.516	21.874	39.098
F	-4.001	22.903	38.12
F	-3.69	21.096	37.508
C	-6.026	20.832	39.212
C	-6.755	20.278	40.199
H	-7.47	19.699	39.961
C	-6.511	20.511	41.563
C	3.7	18.345	41.933
C	2.918	18.855	40.949
H	2.113	19.3	41.183
C	3.264	18.743	39.605
C	2.436	19.481	38.645
F	1.712	20.482	39.098
F	1.564	18.682	38.12
F	2.973	19.854	37.508

C	4.37	17.963	39.212
C	5.214	17.609	40.199
H	6.072	17.279	39.961
C	4.89	17.703	41.563
H	-4.55366	27.02187	32.80742
H	-3.34121	26.4394	28.04078
H	-5.63299	27.2449	28.71031
H	1.65086	29.75009	42.30631
H	1.72974	27.34225	42.96129
H	1.8289	28.68319	38.19474
H	-1.72974	16.14325	32.80729
H	-1.3816	15.09706	28.71025
H	-1.8289	17.48419	28.04074
H	5.54035	17.29165	42.30646
H	3.41691	18.43021	42.96135
H	4.52855	17.67109	38.19487
H	-5.14624	21.4243	42.96132
H	-7.19177	20.15237	42.30653
H	-6.35779	20.8404	38.19478
H	6.28111	24.02889	32.80737
H	7.01477	24.8519	28.71021
H	5.17014	23.27159	28.04078

C_2F_5-cage hexamer cutout

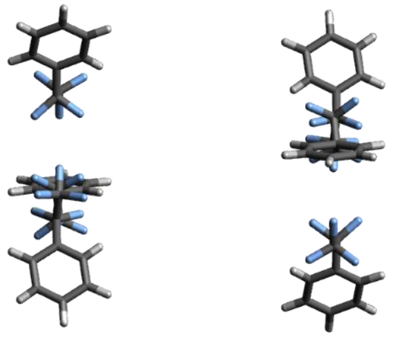

C	4.4213	18.0902	31.124
C	3.2687	18.7699	31.3169
H	2.7912	19.0523	30.5451
C	2.7312	19.0854	32.5539
C	3.3785	18.6924	33.7093
C	4.5436	18.0338	33.5171
H	5.0269	17.7615	34.2881
C	5.0856	17.7261	32.2793
C	6.6055	17.4482	32.263
F	7.4626	17.7413	31.2391
F	7.337	17.8048	33.3701
C	6.6997	15.9249	32.3913
F	7.9713	15.4736	32.4085
F	6.1071	15.582	33.549
F	6.0598	15.3381	31.3869
C	7.7999	23.8747	44.0907
C	7.8122	22.5366	44.2836
H	7.818	21.9819	43.5126
C	7.8166	21.9132	45.5206
C	8.336	20.4582	45.537
F	9.0182	19.8623	46.5616
F	9.0108	20.003	44.4291
C	7.0638	19.6148	45.4094
F	7.3088	18.2881	45.3915
F	6.4705	19.9565	44.2509
F	6.236	19.8757	46.413
C	7.8001	22.6707	46.676
C	7.8121	24.0086	46.4838
H	7.818	24.5635	47.2548
C	7.8167	24.6321	45.246
C	5.656	12.272	44.0907
C	6.8087	12.9517	44.2836
H	7.2861	13.2341	43.5126
C	7.3463	13.2674	45.5206
C	8.3468	14.4446	45.537
F	8.5217	15.3334	46.5616
F	8.4037	15.2564	44.4291
C	9.7133	13.7645	45.4094
F	10.7397	14.6401	45.3915
F	9.714	13.0798	44.2509
F	9.9012	12.9171	46.413
C	6.6987	12.874	46.676
C	5.5339	12.2158	46.4838
H	5.0505	11.9433	47.2548
C	4.9917	11.9079	45.246
C	12.3548	11.0343	33.7095

C	12.3425	12.3723	33.5166
H	12.3367	12.927	34.2876
C	12.3379	12.9958	32.2796
C	11.8187	14.4508	32.2632
F	11.1365	15.0467	31.2386
F	11.1441	14.906	33.3711
C	13.0909	15.2942	32.3908
F	12.8459	16.6209	32.4087
F	13.6842	14.9524	33.5493
F	13.9187	15.0333	31.3872
C	12.3548	12.2383	31.1242
C	12.3424	10.9004	31.3164
H	12.3367	10.3455	30.5454
C	12.338	10.2769	32.5542
C	16.7762	16.2167	44.0907
C	15.6112	16.8751	44.2836
H	15.1279	17.1474	43.5126
C	15.069	17.1828	45.5206
C	13.5493	17.4607	45.537
F	12.6921	17.1678	46.5616
F	12.8178	17.1041	44.4291
C	13.455	18.9841	45.4094
F	12.1835	19.4353	45.3915
F	14.0476	19.3271	44.2509
F	14.0949	19.5706	46.413
C	15.7335	16.8187	46.676
C	16.8859	16.1391	46.4838
H	17.3636	15.8566	47.2548
C	17.4236	15.8235	45.246
C	14.4987	22.637	33.7095
C	13.346	21.9573	33.5166
H	12.8686	21.6749	34.2876
C	12.8084	21.6415	32.2796
C	11.8079	20.4644	32.2632
F	11.633	19.5756	31.2386
F	11.751	19.6526	33.3711
C	10.4414	21.1444	32.3908
F	9.415	20.2689	32.4087
F	10.4407	21.8292	33.5493
F	10.2535	21.9918	31.3872
C	13.456	22.0349	31.1242
C	14.6208	22.6932	31.3164
H	15.1042	22.9656	30.5454
C	15.163	23.0011	32.5542
H	16.09628	23.5209	32.61502
H	14.86901	22.87527	34.68469
H	13.06429	21.83329	30.1491
H	4.79159	17.8519	30.14881
H	2.98677	18.89429	34.68433
H	1.81435	19.63368	32.61457
H	7.77872	24.31452	43.1155
H	7.77935	22.23065	47.6511
H	7.83327	25.70024	45.18516
H	15.36308	17.05718	47.6511
H	18.3404	15.27515	45.18519
H	17.16773	16.01513	43.11552
H	12.37598	10.59448	34.6847
H	12.32159	9.20876	32.61504
H	12.37613	12.67835	30.14911
H	7.09044	13.07556	47.6511
H	4.05842	11.3881	45.18518
H	5.28569	12.03373	43.11551

C_3F_7-cage hexamer cutout

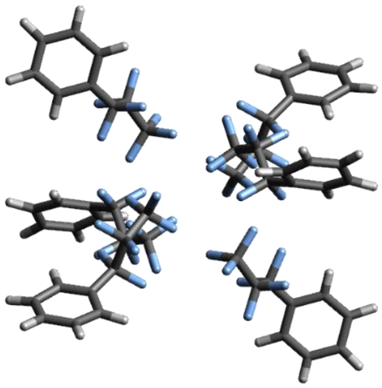

C	3.7419	18.7401	-3.8261
C	3.0175	19.2705	-4.8572
H	2.2457	19.7815	-4.6425
C	3.3382	19.11	-6.195

C	2.7569	20.0242	-7.1682
F	1.6176	20.5563	-6.6293
F	2.4437	19.3547	-8.3106
C	3.5478	21.3456	-7.6442
F	3.793	22.006	-6.5012
F	4.7716	20.9972	-8.0151
C	2.7054	22.3487	-8.5041
F	1.638	22.8042	-7.8072
F	3.657	23.1887	-8.9049
F	2.2418	21.689	-9.5756
C	4.4451	18.3339	-6.5447
C	5.2668	17.9719	-5.5137
H	6.0949	17.5592	-5.7283
C	4.9672	18.1694	-4.1758
C	6.1716	24.4634	-14.1968
C	6.8959	24.9942	-15.2278
H	7.6677	25.505	-15.0132
C	6.5751	24.8335	-16.5657
C	5.4682	24.0574	-16.9154
C	4.6466	23.6954	-15.8843
H	3.8183	23.2825	-16.099
C	4.9461	23.8927	-14.5465
C	3.8638	23.8465	-13.5727
F	2.8332	23.1257	-14.1122
F	4.2868	23.2406	-12.4303
C	3.115	25.1923	-13.0973
F	2.6657	25.7349	-14.2404
F	4.0286	26.078	-12.7258
C	1.8248	24.9643	-12.2374
F	0.8966	24.2675	-12.9349
F	1.5732	26.2085	-11.836
F	2.1645	24.2329	-11.1652
C	-4.445	27.4539	-14.1968
C	-5.2669	27.8158	-15.2278
H	-6.0951	28.2288	-15.0132
C	-4.9673	27.6183	-16.5657
C	-3.7417	27.0478	-16.9154
C	-3.0174	26.5172	-15.8843
H	-2.2457	26.0064	-16.099
C	-3.338	26.6779	-14.5465

C	-2.7569	25.7638	-13.5727
F	-1.6174	25.2317	-14.1122
F	-2.4436	26.4331	-12.4303
C	-3.548	24.4423	-13.0973
F	-3.7932	23.782	-14.2404
F	-4.7718	24.7907	-12.7258
C	-2.7055	23.4391	-12.2374
F	-1.6379	22.9835	-12.9349
F	-3.6571	22.5991	-11.836
F	-2.2418	24.0989	-11.1652
C	-1.7265	16.7644	-14.1968
C	-1.6291	15.8717	-15.2278
H	-1.5726	14.9479	-15.0132
C	-1.6078	16.2299	-16.5657
C	-1.7265	17.5766	-16.9154
C	-1.6292	18.4691	-15.8843
H	-1.5727	19.3929	-16.099
C	-1.608	18.1111	-14.5465
C	-1.1069	19.0714	-13.5727
F	-1.2159	20.3243	-14.1122
F	-1.8432	19.0081	-12.4303
C	0.433	19.047	-13.0973
F	1.1275	19.1648	-14.2404
F	0.7432	17.813	-12.7258
C	0.8806	20.2783	-12.2374
F	0.7413	21.4306	-12.9349
F	2.0839	19.8741	-11.836
F	0.0773	20.3499	-11.1652
C	1.7265	28.2114	-3.8261
C	1.6291	27.3187	-4.8572
H	1.5726	26.3949	-4.6425
C	1.6078	27.6768	-6.195
C	1.1068	26.7163	-7.1688
F	1.2158	25.4634	-6.6293
F	1.8432	26.7799	-8.3106
C	-0.4329	26.7407	-7.6442
F	-1.1276	26.6229	-6.5012
F	-0.7431	27.9747	-8.0151
C	-0.8805	25.5094	-8.5041
F	-0.7412	24.3571	-7.8066

F	-2.0838	25.9136	-8.9055
F	-0.0774	25.4378	-9.5756
C	1.7265	29.0235	-6.5447
C	1.6292	29.916	-5.5137
H	1.5727	30.8398	-5.7283
C	1.608	29.558	-4.1758
C	-5.4683	21.7304	-3.8261
C	-4.6465	22.0923	-4.8572
H	-3.8182	22.5053	-4.6425
C	-4.9461	21.8949	-6.195
C	-3.8637	21.9412	-7.1688
F	-2.8331	22.662	-6.6293
F	-4.2869	22.5472	-8.3106
C	-3.115	20.5956	-7.6442
F	-2.6656	20.0528	-6.5012
F	-4.0286	19.7099	-8.0151
C	-1.8248	20.8236	-8.5041
F	-0.8966	21.5204	-7.8066
F	-1.5732	19.5794	-8.9055
F	-2.1644	21.5549	-9.5756
C	-6.1717	21.3243	-6.5447
C	-6.8959	20.7937	-5.5137
H	-7.6677	20.2829	-5.7283
C	-6.5753	20.9544	-4.1758
H	-3.39157	27.0272	-17.92628
H	-4.63786	27.74679	-13.1859
H	-5.66802	27.90009	-17.32365
H	1.88366	29.33702	-7.55559
H	1.50189	30.30582	-3.41791
H	1.88359	27.89791	-2.81519
H	-6.52183	21.30367	-7.55558
H	-7.16989	20.48862	-3.4179
H	-5.27537	22.02327	-2.81521
H	5.27524	23.76455	-17.92629
H	7.16954	25.29941	-17.32364
H	6.52169	24.484	-13.1859
H	-1.88365	17.89014	-17.92629
H	-1.8836	16.45092	-13.18588
H	-1.50146	15.48216	-17.32364
H	4.638	18.04104	-7.5556
H	3.39196	18.76104	-2.81516
H	5.66784	17.8874	-3.41786

C_5F_{11}-cage hexamer cutout

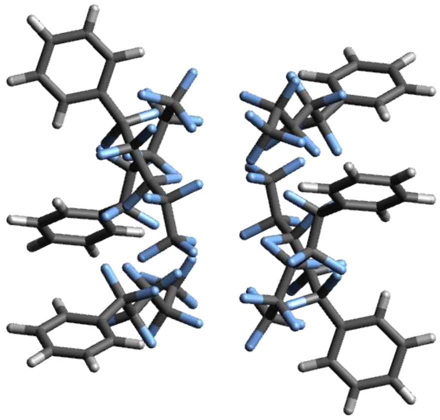

C	-7.7633	-0.3136	17.8842
C	-7.7809	-1.2833	16.9293
H	-7.8025	-2.1925	17.204
C	-7.7685	-0.999	15.5676
C	-7.7633	0.3136	15.1344
C	-7.7809	1.2833	16.0893
H	-7.8025	2.1925	15.8146
C	-7.7685	0.999	17.451
C	-7.956	2.1828	18.3022
F	-8.5698	1.8521	19.477
F	-8.8155	3.0361	17.6412
C	-6.7568	3.0097	18.6416
F	-5.9004	2.2447	19.37
F	-6.0768	3.2598	17.4734
C	-6.8134	4.3509	19.3317
F	-7.7325	4.2636	20.3394
F	-7.3124	5.3011	18.4607
C	-5.5708	4.9686	19.8943
F	-4.4031	4.787	19.2234
F	-5.384	4.3463	21.1339
C	-5.6082	6.452	20.1974
F	-5.62	7.162	19.0326
F	-6.767	6.6552	20.8849

F	-4.5593	6.8809	20.9338
C	-13.3771	1.241	28.8904
C	-12.5461	1.7411	27.9355
H	-11.7696	2.2144	28.2102
C	-12.7862	1.5881	26.5738
C	-11.8547	2.3425	25.7226
F	-12.4481	2.7087	24.5478
F	-11.5455	3.5135	26.3836
C	-10.539	1.7174	25.3831
F	-10.7733	0.5932	24.6547
F	-9.9825	1.2535	26.5513
C	-9.4058	2.437	24.693
F	-9.941	3.1893	23.6853
F	-8.8324	3.3443	25.5641
C	-8.2496	1.6697	24.1304
F	-7.823	0.5677	24.8013
F	-8.6951	1.1968	22.8909
C	-6.9836	2.4438	23.8273
F	-6.3746	2.809	24.9922
F	-7.3871	3.549	23.1398
F	-6.0877	1.7498	23.091
C	-13.9204	0.9273	26.1406
C	-14.7689	0.4577	27.0955
H	-15.5671	0.0219	26.8207
C	-14.5165	0.5892	28.4571
C	-15.3809	6.5664	15.1344
C	-14.5324	6.0968	16.0893
H	-13.7342	5.661	15.8146
C	-14.7848	6.2282	17.451
C	-13.6658	5.7987	18.3022
F	-13.6453	6.4957	19.477
F	-12.4971	6.1164	17.6412
C	-13.5493	4.3467	18.6416
F	-14.64	3.9875	19.37
F	-13.6728	3.6328	17.4734
C	-12.3595	3.7251	19.3317
F	-11.9755	4.5647	20.3394
F	-11.2871	3.6822	18.4607
C	-12.4459	2.3401	19.8943
F	-13.187	1.4197	19.2234

F	-13.0781	2.4895	21.1339
C	-11.1425	1.6308	20.1974
F	-10.5217	1.286	19.0326
F	-10.3871	2.5328	20.8849
F	-11.2956	0.508	20.9338
C	-15.9242	6.88	17.8842
C	-16.7552	7.3801	16.9293
H	-17.5317	7.8534	17.204
C	-16.5151	7.2272	15.5676
C	-6.1571	10.0371	15.1344
C	-6.9881	9.537	16.0893
H	-7.7646	9.0637	15.8146
C	-6.748	9.69	17.451
C	-7.6795	8.9356	18.3022
F	-7.0861	8.5694	19.477
F	-7.9887	7.7646	17.6412
C	-8.9952	9.5607	18.6416
F	-8.7609	10.6848	19.37
F	-9.5517	10.0246	17.4734
C	-10.1284	8.8411	19.3317
F	-9.5932	8.0887	20.3394
F	-10.7018	7.9338	18.4607
C	-11.2846	9.6084	19.8943
F	-11.7112	10.7104	19.2234
F	-10.8391	10.0812	21.1339
C	-12.5506	8.8343	20.1974
F	-13.1596	8.469	19.0326
F	-12.1471	7.7291	20.8849
F	-13.4465	9.5282	20.9338
C	-5.6138	10.3507	17.8842
C	-4.7653	10.8204	16.9293
H	-3.9671	11.2561	17.204
C	-5.0177	10.6889	15.5676
C	-4.1533	4.7117	28.8904
C	-5.0018	5.1813	27.9355
H	-5.8	5.6171	28.2102
C	-4.7494	5.0499	26.5738
C	-5.8684	5.4794	25.7226
F	-5.8889	4.7824	24.5478
F	-7.0371	5.1617	26.3836

C	-5.9849	6.9314	25.3831
F	-4.8942	7.2905	24.6547
F	-5.8614	7.6453	26.5513
C	-7.1747	7.5529	24.693
F	-7.5587	6.7133	23.6853
F	-8.2471	7.5959	25.5641
C	-7.0883	8.9379	24.1304
F	-6.3472	9.8584	24.8013
F	-6.4561	8.7886	22.8909
C	-8.3917	9.6473	23.8273
F	-9.0125	9.992	24.9922
F	-9.1471	8.7452	23.1398
F	-8.2386	10.7701	23.091
C	-3.61	4.3981	26.1406
C	-2.779	3.898	27.0955
H	-2.0025	3.4246	26.8207
C	-3.0191	4.0509	28.4571
C	-11.7709	10.9644	28.8904
C	-11.7533	9.9947	27.9355
H	-11.7317	9.0856	28.2102
C	-11.7657	10.2791	26.5738
C	-11.5782	9.0953	25.7226
F	-10.9644	9.426	24.5478
F	-10.7187	8.242	26.3836
C	-12.7774	8.2684	25.3831
F	-13.6338	9.0333	24.6547
F	-13.4574	8.0183	26.5513
C	-12.7208	6.9272	24.693
F	-11.8017	7.0145	23.6853
F	-12.2218	5.9769	25.5641
C	-13.9634	6.3095	24.1304
F	-15.1311	6.491	24.8013
F	-14.1502	6.9317	22.8909
C	-13.926	4.8261	23.8273
F	-13.9142	4.116	24.9922
F	-12.7672	4.6229	23.1398
F	-14.9749	4.3972	23.091
C	-11.7709	11.5917	26.1406
C	-11.7533	12.5614	27.0955
H	-11.7317	13.4705	26.8207
C	-11.7657	12.277	28.4571
H	-3.39062	4.29125	25.0988
H	-2.32516	3.65662	29.16977
H	-4.35555	4.8482	29.93221
H	-5.41147	10.48709	18.92601
H	-4.32924	11.09266	14.85493
H	-6.37648	9.93025	14.0926
H	-11.78803	10.72102	29.93221
H	-11.77122	13.07511	29.16977
H	-11.78803	11.83511	25.0988
H	-7.76298	-1.79711	14.85494
H	-7.74617	0.55701	14.0926
H	-7.74617	-0.55701	18.926
H	-15.17862	6.42988	14.0926
H	-17.20902	7.62147	14.85491
H	-16.14358	6.98685	18.926
H	-13.15775	1.34784	29.93221
H	-15.20497	0.18543	29.16975
H	-14.12268	0.79078	25.0988

C_6F_{13}-cage hexamer cutout

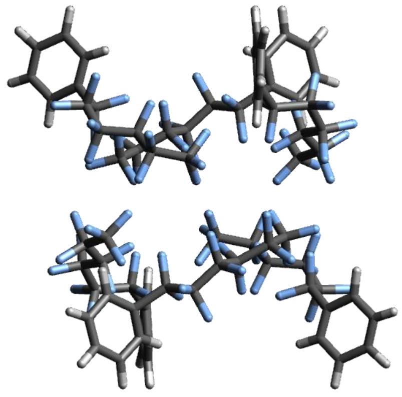

C	3.7208	7.088	17.8974
C	2.8681	7.5464	16.9796
H	2.0863	8.0206	17.2372
C	3.1278	7.3321	15.7413
C	4.278	6.7663	15.2116
C	5.1014	6.2569	16.1294
H	5.9029	5.8171	15.8718
C	4.7859	6.3748	17.3677

C	5.4506	5.1665	18.2775
F	5.4745	5.3933	19.5946
F	6.7325	5.2237	17.906
C	4.9915	3.6836	18.1623
F	3.6639	3.5003	18.1954
F	5.3778	3.147	16.9862
C	5.5369	2.6774	19.2257
F	5.0851	2.8811	20.464
F	5.1061	1.426	18.9635
C	7.0867	2.6499	19.3449
F	7.611	3.7104	19.9932
F	7.6776	2.727	18.1523
C	7.6928	1.4345	20.0972
F	7.1163	1.2572	21.3043
F	7.4722	0.2887	19.433
C	9.2105	1.4749	20.3985
F	9.703	0.2779	20.7216
F	9.9765	1.7376	19.3555
F	9.5963	2.3169	21.3573
C	12.082	0.3217	15.2116
C	12.1116	1.2892	16.1294
H	12.0915	2.2035	15.8718
C	12.167	0.9573	17.3677
C	12.8811	2.1371	18.2775
F	12.6726	2.0446	19.5946
F	12.1907	3.2184	17.906
C	14.3948	2.4809	18.1623
F	15.2174	1.4229	18.1954
F	14.6665	3.0837	16.9862
C	14.9936	3.4563	19.2257
F	15.0432	2.9631	20.464
F	16.2926	3.7092	18.9635
C	14.2424	4.8123	19.3449
F	13.0619	4.7361	19.9932
F	13.8804	5.2853	18.1523
C	14.9919	5.9449	20.0972
F	15.4337	5.5345	21.3043
F	16.0946	6.3268	19.433
C	14.1982	7.2391	20.3985
F	14.9884	8.2643	20.7216
F	13.5876	7.7711	19.3555
F	13.276	7.1523	21.3573
C	12.082	-0.3217	17.8974
C	12.1112	-1.2892	16.9796
H	12.0915	-2.2035	17.2372
C	12.167	-0.9573	15.7413
C	15.8027	4.8273	28.9337
C	14.9795	5.3364	28.016
H	14.1778	5.7765	28.2735
C	15.2948	5.2188	26.7777
C	14.6301	6.4271	25.8678
F	14.6061	6.2005	24.5508
F	13.3483	6.3697	26.2393
C	15.0892	7.91	25.9831
F	16.4168	8.0933	25.95
F	14.703	8.4465	27.1591
C	14.5438	8.9162	24.9196
F	14.9957	8.7124	23.6813
F	14.9745	10.1678	25.1818
C	12.994	8.9436	24.8004
F	12.4697	7.8832	24.1521
F	12.4032	8.8664	25.993
C	12.3879	10.1591	24.0482
F	12.9643	10.3366	22.841
F	12.6085	11.3049	24.7123
C	10.8702	10.1187	23.7469
F	10.3776	11.3159	23.4237
F	10.1042	9.8559	24.7898
F	10.4843	9.2768	22.788
C	16.3599	4.5056	26.2479
C	17.2126	4.0476	27.1657
H	17.9944	3.573	26.9081
C	16.9529	4.2615	28.404
C	14.3183	10.6241	17.8974
C	15.1416	11.1332	16.9796
H	15.9433	11.5733	17.2372
C	14.8263	11.0156	15.7413
C	13.7611	10.3024	15.2116
C	12.9085	9.8444	16.1294
H	12.1266	9.3698	15.8718

C	13.1681	10.0583	17.3677
C	11.7894	10.0868	18.2775
F	11.9737	9.9525	19.5946
F	11.1981	8.9482	17.906
C	10.7347	11.2258	18.1623
F	11.2398	12.4672	18.1954
F	10.0769	11.1598	16.9862
C	9.5906	11.2566	19.2257
F	9.993	11.5462	20.464
F	8.7222	12.2552	18.9635
C	8.7919	9.9282	19.3449
F	9.4482	8.9439	19.9932
F	8.5633	9.3781	18.1523
C	7.4363	10.011	20.0972
F	7.5708	10.5988	21.3043
F	6.5542	10.7749	19.433
C	6.7124	8.6764	20.3985
F	5.4294	8.8482	20.7216
F	6.557	7.8817	19.3555
F	7.2486	7.9212	21.3573
C	7.9987	11.2719	28.9337
C	7.9695	10.3044	28.016
H	7.9892	9.3901	28.2735
C	7.9137	10.6363	26.7777
C	7.1996	9.4565	25.8678
F	7.4079	9.549	24.5508
F	7.8902	8.3752	26.2393
C	5.6859	9.1127	25.9831
F	4.8633	10.1707	25.95
F	5.4144	8.5099	27.1591
C	5.0871	8.1373	24.9196
F	5.0377	8.6304	23.6813
F	3.7879	7.8844	25.1818
C	5.8383	6.7813	24.8004
F	7.0188	6.8575	24.1521
F	6.2005	6.3083	25.993
C	5.0888	5.6487	24.0482
F	4.6468	6.0591	22.841
F	3.9861	5.2668	24.7123
C	5.8825	4.3545	23.7469

F	5.0921	3.3293	23.4237
F	6.4931	3.8225	24.7898
F	6.8045	4.4413	22.788
C	7.9987	11.9153	26.2479
C	7.9691	12.8827	27.1657
H	7.9892	13.7971	26.9081
C	7.9137	12.5509	28.404
C	6.3196	1.2912	28.9337
C	7.172	1.7496	28.016
H	7.9541	2.2238	28.2735
C	6.9126	1.5353	26.7777
C	8.2913	1.5068	25.8678
F	8.1071	1.6409	24.5508
F	8.8825	2.6455	26.2393
C	9.346	0.3677	25.9831
F	8.8409	-0.8736	25.95
F	10.0037	0.434	27.1591
C	10.4901	0.337	24.9196
F	10.0876	0.0476	23.6813
F	11.3586	-0.6618	25.1818
C	11.2888	1.6654	24.8004
F	10.6325	2.6497	24.1521
F	11.5173	2.2157	25.993
C	12.6444	1.5826	24.0482
F	12.51	0.9947	22.841
F	13.5265	0.8187	24.7123
C	13.3683	2.9172	23.7469
F	14.6514	2.7452	23.4237
F	13.5237	3.7119	24.7898
F	12.8322	3.6723	22.788
C	5.7624	0.9695	26.2479
C	4.9394	0.4601	27.1657
H	4.1374	0.0203	26.9081
C	5.2544	0.578	28.404
H	16.48605	4.33808	25.19865
H	15.59469	4.85304	29.98297
H	17.70702	3.9713	29.10542
H	12.00014	0.51473	14.16234
H	12.00043	-0.51473	18.94668
H	12.29303	-1.75546	15.0399

H	14.52629	10.64988	18.94667	H	6.44597	1.4584	29.98297
H	15.45449	11.52385	15.03991	H	4.62597	0.07005	29.1054
H	13.63485	10.1349	14.16236	H	5.55426	0.94398	25.19865
H	3.59455	7.2553	18.94667				
H	4.48602	6.74069	14.16233				
H	2.37358	7.62197	15.03985				
H	8.08027	11.0789	29.98298				
H	8.08055	12.10833	25.19864				
H	7.78805	13.34911	29.10541				

12. Literature Comparison

Table S22. Comparison of literature known PFC-218/N_2 and PFC-318/N_2 selectivities with those of **C_5F_{11}-cage**.

Material	Gas mixture	ratio	Temp. [K]	Selectivity	Method	Reference
**C_5F_{11}-cage**	C_3F_8/N_2	10:90	298K	174	IAST	*this work*
**C_5F_{11}-cage**	C_3F_8/N_2	1:99	298K	422	IAST	*this work*
MOF A520	C_3F_8/N_2	10:90	298K	6034	IAST	[S21]
CPOF-6	C_3F_8/N_2	10:90	298K	148	IAST	[S22]
Co0.2Cr-MIL-101	C_3F_8/N_2	1:999	298K	146.7	IAST	[S23]
**C_5F_{11}-cage**	C_4F_8/N_2	10:90	298K	301	IAST	*this work*
CPOF-6	C_4F_8/N_2	10:90	298K	418	IAST	[S22]

13. References

[S1] C. Zhang, C. F. Chen, *J. Org. Chem.* **2006**, 71, 6626.

[S2] E. F. DiMauro, J. R. Vitullo, *J. Org. Chem.* **2006**, 71, 3959.

[S3] J. Pang, Z. Di, J.-S. Qin, S. Yuan, C. T. Lollar, J. Li, P. Zhang, M. Wu, D. Yuan, M. Hong, H.-C. Zhou, *J. Am. Chem. Soc.* **2020**, 142, 15020.

[S4] K. Tian, S. M. Elbert, X. Y. Hu, T. Kirschbaum, W. S. Zhang, F. Rominger, R. R. Schroder, M. Mastalerz, *Adv. Mater.* **2022**, 34, e2202290.

[S5] J. Lim, T. M. Swager, *Angew. Chem. Int. Ed.* **2010**, 49, 7486.

[S6] G. E. Carr, R. D. Chambers, T. F. Holmes, D. G. Parker, *J. Chem. Soc., Perkin Trans. 1* **1988**, 921.

[S7] S. Kusaka, Y. Itoh, A. Hori, J. Usuba, J. Pirillo, Y. Hijikata, Y. Ma, R. Matsuda, *Nat. Commun.* **2024**, 15, 10117.

[S8] A. F. Saturno, *J. Chem. Educ.* **1962**, 39, 464.

[S9] G. M. Kontogeorgis, R. Privat, J.-N. Jaubert, *J. Chem. Eng. Data* **2019**, 64, 4619.

[S10] P. J. Linstrom, W. G. Mallard, *NIST Chemistry WebBook, NIST Standard Reference Database Number 69, National Institute of Standards and Technology, Gaithersburg MD, 20899, https://doi.org/10.18434/T4D303, (retrieved February 8, 2022).* .

[S11] A. L. Myers, J. M. Prausnitz, *AIChE J.* **1965**, 11, 121.

[S12] A. Nuhnen, C. Janiak, *Dalton Trans.* **2020**, 49, 10295.

[S13] M. J. Frisch, G. W. Trucks, H. B. Schlegel, G. E. Scuseria, M. A. Robb, J. R. Cheeseman, G. Scalmani, V. Barone, G. A. Petersson, H. Nakatsuji, X. Li, M. Caricato, A. V. Marenich, J. Bloino, B. G. Janesko, R. Gomperts, B. Mennucci, H. P. Hratchian, J. V. Ortiz, A. F. Izmaylov, J. L. Sonnenberg, Williams, F. Ding, F. Lipparini, F. Egidi, J. Goings, B. Peng, A. Petrone, T. Henderson, D. Ranasinghe, V. G. Zakrzewski, J. Gao, N. Rega, G. Zheng, W. Liang, M. Hada, M. Ehara, K. Toyota, R. Fukuda, J. Hasegawa, M. Ishida, T. Nakajima, Y. Honda, O. Kitao, H. Nakai, T. Vreven, K. Throssell, J. A. Montgomery Jr., J. E. Peralta, F. Ogliaro, M. J. Bearpark, J. J. Heyd, E. N. Brothers, K. N. Kudin, V. N. Staroverov, T. A. Keith, R. Kobayashi, J. Normand, K. Raghavachari, A. P. Rendell, J. C. Burant, S. S. Iyengar, J. Tomasi, M. Cossi, J. M. Millam, M. Klene, C. Adamo, R. Cammi, J. W. Ochterski, R. L. Martin, K. Morokuma,

O. Farkas, J. B. Foresman, D. J. Fox, *Gaussian 16 Rev. C.01* **Gaussian Inc., Wallingford, CT, 2016**.

[S14] a) W. Koch, *A Chemist's Guide to Density Functional Theory*, Wiley-VCH, Weinheim **2001**; b) R. G. Parr, W. Yang, *Density-functional theory of atoms and molecules*, Oxford Univ. Press, New York, NY **1994**; c) P. Hohenberg, W. Kohn, *Phys. Rev.* **1964**, 136, B864; d)W. Kohn, L. J. Sham, *Phys. Rev.* **1965**, 140, A1133.

[S15] a) C. Lee, W. Yang, R. G. Parr, *Phys. Rev. B* **1988**, 37, 785; b) P. J. Stephens, F. J. Devlin, C. F. Chabalowski, M. J. Frisch, *J. Phys. Chem.* **1994**, 98, 11623; c) S. H. Vosko, L. Wilk, M. Nusair, *Can. J. Phys.* **1980**, 58, 1200.

[S16] a) T. H. Dunning, *J. Chem. Phys.* **1989**, 90, 1007; b)R. A. Kendall, T. H. Dunning, R. J. Harrison, *J. Chem. Phys.* **1992**, 96, 6796.

[S17] a) F. O. Kannemann, A. D. Becke, *J. Chem. Theory Comput.* **2010**, 6, 1081; b) A. Otero-de-la-Roza, E. R. Johnson, *J. Chem. Phys.* **2013**, 138, 204109.

[S18] T. Lu, Q. Chen, *J. Comput. Chem.* **2022**, 43, 539.

[S19] a) T. Lu, F. Chen, *J. Comput. Chem.* **2012**, 33, 580; b)T. Lu, *J. Chem. Phys.* **2024**, 161, 082503.

[S20] W. Humphrey, A. Dalke, K. Schulten, *J. Mol. Graph.* **1996**, 14, 33.

[S21] Z. Fang, H. Huang, L. Sun, M. Zheng, Z. Lv, T. Yan, C. Zhong, *Chem. Eng. J.* **2025**, 516, 163987.

[S22] M. Chen, Y. Zhao, D. Wen, W. Gong, Y. Chen, Y. Gao, Y. Yu, G. Xing, Y. Zhang, W. Zhu, T. Ben, *Nat. Commun.* **2025**, 16, 5499.

[S23] Y. Zhaochun, Y. JiaCheng, Z. Wen, Y. Wenhui, G. Jianyu, L. Li, X. Hong, *Chem. Eng. Sci.* **2023**, 282, 119302
